# ESICM LIVES 2016: part two: Milan, Italy. 1–5 October 2016

**DOI:** 10.1186/s40635-016-0099-9

**Published:** 2016-09-29

**Authors:** S. Sivakumar, F. S. Taccone, K. A. Desai, C. Lazaridis, M. Skarzynski, M. Sekhon, W. Henderson, D. Griesdale, L. Chapple, A. Deane, L. Williams, R. Strickland, K. Lange, D. Heyland, M. Chapman, M. J. Rowland, P. Garry, J. Westbrook, R. Corkill, C. A. Antoniades, K. T. Pattinson, G. Fatania, A. J. Strong, R. B. Myers, C. Lazaridis, C. M. Jermaine, C. S. Robertson, C. G. Rusin, J. Hofmeijer, L. Sondag, M. C. Tjepkema-Cloostermans, A. Beishuizen, F. H. Bosch, M. J. A. M. van Putten, L. Carteron, C. Patet, D. Solari, M. Oddo, M. A. Ali, C. Dias, R. Almeida, A. Vaz-Ferreira, J. Silva, E. Monteiro, A. Cerejo, A. P. Rocha, A. A. Elsayed, A. M. Abougabal, B. N. Beshey, K. M. Alzahaby, S. Pozzebon, A. Blandino Ortiz, S. Cristallini, O. Lheureux, A. Brasseur, J. L. Vincent, J. Creteur, F. S. Taccone, M. Hravnak, K. Yousef, Y. Chang, E. Crago, R. M. Friedlander, S. A. Abdelmonem, S. A. Tahon, T. A. Helmy, H. S. Meligy, F. Puig, I. Dunn-Siegrist, J. Pugin, S. Gupta, D. Govil, S. Srinivasan, S. J. Patel, J. K. N, A. Gupta, D. S. Tomar, M. Shafi, R. Harne, D. P. Arora, N. Talwar, S. Mazumdar, E. E. Papakrivou, D. Makris, E. Manoulakas, B. Tsolaki, B. Karadodas, E. Zakynthinos, I. Palacios Garcia, A. Diaz Martin, V. Sanchez Encinares, M. Pachón Ibañez, J. Garnacho Montero, G. Labrador, T. Cebrero Cangueiro, V. Poulose, J. Koh, J. W. Kam, H. Yeter, A. Kara, O. Aktepe, A. Topeli, I. Tsolakoglou, G. Intas, P. Stergiannis, A. A. Kolaros, E. Chalari, E. Athanasiadou, A. Martika, G. Fildisis, V. Faivre, C. Mengelle, B. Favier, D. Payen, A. Poppe, M. S. Winkler, E. Mudersbach, J. Schreiber, M. L. Wruck, E. Schwedhelm, S. Kluge, C. Zöllner, T. Tavladaki, A. M. Spanaki, H. Dimitriou, E. Kondili, C. Choulaki, E. Meleti, D. Kafetzopoulos, D. Georgopoulos, G. Briassoulis, A. García-de la Torre, M. V. de la Torre-Prados, T. Tsvetanova-Spasova, P. Nuevo-Ortega, C. Rueda-Molina, A. Fernández-Porcel, E. Camara-Sola, L. Salido-Díaz, A. García-Alcántara, T. Tavladaki, A. M. Spanaki, H. Dimitriou, E. Kondili, C. Choulaki, D. E. Meleti, D. Kafetzopoulos, D. Georgopoulos, G. Briassoulis, B. Suberviola, J. Riera, L. Rellan, M. Sanchez, J. C. Robles, E. Lopez, R. Vicente, E. Miñambres, M. Santibañez, M. Le Guen, J. Moore, N. Mason, M. Windpassinger, O. Plattner, E. Mascha, D. I. Sessler, Outcomes Research, U. Melia, J. Fontanet, J. P. van den Berg, M. M. R. F. Struys, H. E. M. Vereecke, E. W. Jensen, P. J. T. Rood, F. van de Schoor, K. van Tertholen, P. Pickkers, M. van den Boogaard, Z. J. Beardow, H. Redhead, K. Paramasivam, T. Numan, M. van den Boogaard, A. M. Kamper, P. Rood, L. M. Peelen, P. M. Zeman, A. J. Slooter, C. E. van Ewijk, G. E. Jacobs, A. R. J. Girbes, S. N. Myatra, M. M. Harish, N. R. Prabu, S. Siddiqui, A. P. Kulkarni, J. V. Divatia, L. D. Murbach, M. A. Leite, E. F. Osaku, C. R. L. M. Costa, M. Pelenz, N. M. Neitzke, M. M. Moraes, J. L. Jaskowiak, M. M. M. Silva, R. S. Zaponi, L. R. L. Abentroth, S. M. Ogasawara, A. C. Jorge, P. A. D. Duarte, N. Hernández-Sánchez, L. A. Sánchez-Hurtado, F. J. García-Guillen, S. A. Ñamendys-Silva, B. Maghsoudi, M. Emami, M. B. Khosravi, F. Zand, H. R. Tabatabaie, M. Masjedi, G. Sabetiyan, A. Mokri, Nurses of the Central and General ICUs of Shiraz Namazi Hospital, J. Troubleyn, M. Diltoer, R. Jacobs, D. N. Nguyen, E. De Waele, J. De Regt, P. M. Honoré, V. Van Gorp, H. D. Spapen, R. S. Contreras, N. D. Toapanta, G. Moreno, J. Sabater, H. Torrado, M. Gonzalez, M. Marin, E. Farigola, A. Gonzalez, J. Fernandez, A. Vera, X. Gisbert, C. Juliá, J. Uya, L. Corral, Sedation an Delirium Group Hospital Universitari de Bellvitge, I. Elias-Jones, L. Gemmell, A. MacKay, D. Randall, A. Adwaney, M. Blunden, J. R. Prowle, C. J. Kirwan, N. Thomas, A. Martin, H. Owen, L. Darwin, D. Conway, D. Atkinson, M. Sharman, J. Moore, C. Barbanti, J. Amour, P. Gaudard, B. Rozec, P. Mauriat, M. M’rini, P. L. Leger, G. Cambonie, J. M. Liet, C. Girard, S. Laroche, P. Damas, Z. Assaf, G. Loron, L. Lecourt, P. Pouard, D. Randall, A. Adwaney, M. Blunden, J.R. Prowle, C. J. Kirwan, S. H. Kim, S. Na, J. Kim, S. Y. Oh, C. W. Jung, S. H. Yoo, S. H. Min, E. J. Chung, H. Lee, N. J. Lee, K. W. Lee, K. S. Suh, H. G. Ryu, D. C. Marshall, R. J. Goodson, J. D. Salciccioli, J. Shalhoub, E. K. Potter, J. Kirk-Bayley, N. D. Karanjia, L. G. Forni, B. C. Creagh-Brown, M. Bossy, M. Nyman, A. Tailor, B. Creagh-Brown, SPACeR group (Surrey Peri-operative, Anaesthesia and Critical Care Collaborative Research Group), D. D’Antini, S. Spadaro, F. Valentino, F. Sollitto, G. Cinnella, L. Mirabella, F. J. Redondo Calvo, N. Bejarano, D. Padilla, V. Baladron, P. Villajero, R. Villazala, J. Redondo, A. S. Yuste, J. Liu, F. Shen, J. L. Teboul, N. Anguel, A. Beurton, N. Bezaz, C. Richard, X. Monnet, T. Fossali, R. Colombo, D. Ottolina, M. Rossetti, C. Mazzucco, A. Marchi, A. Porta, E. Catena, K. H. Tollisen, G. Ø. Andersen, F. Heyerdahl, D. Jacobsen, M. C. de Waard, A. R. J. Girbes, M. C. O. van IJzendoorn, H. Buter, W. P. Kingma, G. J. Navis, E. C. Boerma, J. Rulisek, M. Balik, S. Zacharov, H. S. Kim, S. J. Jeon, H. Namgung, E. Lee, E. Lee, Y. J. Cho, Y. J. Lee, A. Huang, L. Cioccari, N. Luethi, J. Mårtensson, R. Bellomo, M. Forsberg, G. Edman, J. Höjer, S. Forsberg, M. T. Chiquito Freile, F. N. Hidalgo, J. A. Martinez Molina, R. Lecumberri, A. Figuerola Rosselló, P. Medrano Travieso, G. Tuero Leon, J. Gonzalez Sanchez, L. Sahuquillo Frias, D. Balsells Rosello, J. A. Garcia Verdejo, J. A. Noria Serrano, D. Winterwerp, T. van Galen, A. Vazin, I. Karimzade, A. Zand, E. Ozen, S. Ekemen, A. Akcan, E. Sen, B. Buyukkidan Yelken, N. Kureshi, L. Fenerty, G. Thibault-Halman, M. Erdogan, S. Walling, R. S. Green, D. B. Clarke, P. Briassoulis, K. Kalimeris, A. Ntzouvani, T. Nomikos, K. Papaparaskeva, E. Politi, G. Kostopanagiotou, K. Crewdson, M. Rehn, A. Weaver, K. Brohi, D. Lockey, S. Wright, K. Thomas, C. Baker, L. Mansfield, V. Stafford, C. Wade, G. Watson, A. Bryant, T. Chadwick, J. Shen, J. Wilkinson, J. Furneval, A. Henderson, K. Hugill, P. Howard, A. Roy, S. Bonner, S. Baudouin, C. Sánchez Ramírez, S. Hípola Escalada, M. A. Hernández Viera, M. Cabrera Santana, L. Caipe Balcázar, N. Sangil Monroy, F. Artiles Campelo, C. F. Lübbe Vázquez, P. Saavedra Santana, S. Ruiz Santana, L. Carteron, C. Patet, H. Quintard, D. Solari, P. Bouzat, M. Oddo, T. Wollersheim, J. Malleike, K. Haas, N. Carbon, J. Schneider, C. Birchmeier, J. Fielitz, S. Spuler, S. Weber-Carstens, L. Enseñat, A. Pérez-Madrigal, P. Saludes, L. Proença, G. Gruartmoner, C. Espinal, J. Mesquida, W. Huber, M. Eckmann, F. Elkmann, A. Gruber, T. Lahmer, U. Mayr, A. Herner, R. Schellnegger, J. Schneider, R. M. Schmid, W. Ayoub, W. Samy, A. Esmat, A. Battah, S. Mukhtar, W. Mongkolpun, D. Orbegozo Cortés, C. P. R. Cordeiro, J. L. Vincent, J. Creteur, S. Funcke, H. Groesdonk, B. Saugel, G. Wagenpfeil, S. Wagenpfeil, D. A. Reuter, M. M. Fernandez, R. Fernandez, M. Magret, A. González-Castro, M. T. Bouza, M. Ibañez, C. García, B. Balerdi, A. Mas, V. Arauzo, J. M. Añón, F. Ruiz, J. Ferreres, R. Tomás, M. Alabert, A. I. Tizón, S. Altaba, N. Llamas, E C. Goligher, E. Fan, M. Herridge, S. Vorona, M. Sklar, M. Dres, N. Rittayamai, A. Lanys, C. Urrea, G. Tomlinson, W. D. Reid, G. D. Rubenfeld, B. P. Kavanagh, L. J. Brochard, N. D. Ferguson, A. Serpa Neto, M. Gama de Abreu, P. Pelosi, M. J. Schultz, for the PRoVENT investigators and the PROVE Network, C. Guérin, L. Papazian, J. Reignier, L. Ayzac, A. Loundou, J. M. Forel, C. Rolland-Debord, C. Bureau, T. Poitou, M. Clavel, S. Perbet, N. Terzi, A. Kouatchet, T. Similowski, A. Demoule, N. Hunfeld, Z. Trogrlic, S. Ladage, R. J. Osse, B. Koch, W. Rietdijk, J. Devlin, M. van der Jagt, E. Picetti, P. Ceccarelli, F. Mensi, L. Malchiodi, S. Risolo, I. Rossi, M. V. Antonini, F. Servadei, M. L. Caspani, A. Roquilly, S. Lasocki, P. Seguin, T. Geeraerts, P. F. Perrigault, C. Dahyot-Fizelier, C. Paugam-Burtz, F. Cook, R. Cinotti, D. Demeure dit Latte, P. J. Mahe, C. Fortuit, F. Feuillet, K. Asehnoune, C. Marzorati, S. Spina, V. Scaravilli, A. Vargiolu, M. Riva, C. Giussani, E. Sganzerla, G. Citerio, S. Barbadillo, F. J. González de Molina, F. Álvarez-Lerma, A. Rodríguez, SEMICYUC/GETGAG Working Group, T. Zakharkina, I. Martin-Loeches, S. Matamoros, P. Povoa, A. Torres, J. Kastelijn, J. J. Hofstra, M. de Jong, M. Schultz, P. Sterk, A. Artigas, L. J. Bos, A. S. Moreau, I. Martin-Loeches, P. Povoa, J. Salluh, A. Rodriguez, S. Nseir, TAVeM study group, E. de Jong, J. A. van Oers, A. Beishuizen, A. R. J. Girbes, M. W. N. Nijsten, D. W. de Lange, D. Bonvicini, D. Labate, L. Benacchio, A. Olivieri, E. Pizzirani, J. C. Lopez-Delgado, M. Gonzalez-Romero, V. Fuentes-Mila, D. Berbel-Franco, I. Romera-Peregrina, A. Martinez-Pascual, J. Perez-Sanchez, R. Abellan-Lencina, R. E. Ávila-Espinoza, G. Moreno-Gonzalez, F. Sbraga, S. Griffiths, M. P. W. Grocott, B. Creagh-Brown, POPC-CB investigators, J. Doyle, P. Wilkerson, Y. Soon, S. Huddart, M. Dickinson, A. Riga, A. Zuleika, K. Miyamoto, Y. Kawazoe, T. Morimoto, T. Yamamoto, A. Fuke, A. Hashimoto, H. Koami, S. Beppu, Y. Katayama, M. Ito, Y. Ohta, H. Yamamura, DESIRE (DExmedetomidine for Sepsis in ICU Randomized Evaluation) Trial Investigators, S. L. Rygård, L B. Holst, J. Wetterslev, P. I. Johansson, A. Perner, I. W. Soliman, D. W. de Lange, D. van Dijk, J. J. M. van Delden, O. L. Cremer, A. J. C. Slooter, L. M. Peelen, D. McWilliams, C. Snelson, A. Das Neves, C. I. Loudet, M. Busico, D. Vazquez, D. Villalba, M. Veronesi, A. Lischinsky, F. J. L. López, L. Benito Mori, G. Plotnikow, A. Díaz, S. Giannasi, R. Hernandez, L. Krzisnik, C. Cecotti, L. Viola, R. Lopez, J. P. Sottile, G. Benavent, E. Estenssoro, C. M. Chen, C. C. Lai, K. C. Cheng, W. Chou, K. S. Chan, L. E. Roeker, C. M. Horkan, F. K. Gibbons, K. B. Christopher, P. J. M. Weijs, K. M. Mogensen, J. D. Rawn, M. K. Robinson, K. B. Christopher, Z. Tang, C. Qiu, B. Ouyang, C. Cai, X. Guan, T. Regueira, L. Cea, S. Juan Carlos, B. Elisa, C. Puebla, A. Vargas, M. K. Poulsen, L. P. Thomsen, S. Kjærgaard, S. E. Rees, D. S. Karbing, T. Wollersheim, S. Frank, M. C. Müller, N. M. Carbon, V. Skrypnikov, P. A. Pickerodt, R. Falk, A. Mahlau, S. Weber-Carstens, A. Lee, R. Inglis, R. Morgan, G. Barker, K. Kamata, T. Abe, D. Saitoh, Y. Tokuda, R. S. Green, M. B. Butler, M. Erdogan, H. Tae Hwa, L. Jae Gil, R. Hernández Vaquero, E. Rodriguez-Ruiz, A. Lopez Lago, J. L. Garcia Allut, A. Estany Gestal, M. A. Garcia Gonzalez, D. O. Thomas-Rüddel, D. Schwarzkopf, C. Fleischmann, K. Reinhart, S. Suwanpasu, Y. Sattayasomboon, N. M. Filgueiras Filho, J. C. A. Oliveira, C. S. Ballalai, C. V. De Lucia, G. P. Araponga, L. N. Veiga, C. S. Silva, M. E. Garrido, B. B. Ramos, E. F. Ricaldi, S. S. Gomes, GEMINI, L. Gemmell, A. MacKay, C. Wright, R. I. Docking, P. Doherty, E. Black, P. Stenhouse, M. P. Plummer, M. E. Finnis, L. K. Phillips, P. Kar, S. Bihari, V. Biradar, S. Moodie, M. Horowitz, J. E. Shaw, A. M. Deane, T. Yatabe, S. Inoue, M. Sakaguchi, M. Egi, Y. Ali Abdelhamid, M. P. Plummer, M. E. Finnis, L. K. Phillips, P. Kar, S. Bihari, V. Biradar, S. Moodie, M. Horowitz, J. E. Shaw, A. M. Deane, M. Hokka, M. Egi, S. Mizobuchi, P. Kar, M. Plummer, Y. Ali Abdelhamid, E. Giersch, M. Summers, S. Hatzinikolas, S. Heller, M. Chapman, K. Jones, M. Horowitz, A. Deane, R. Schweizer, M. Jacquet-Lagreze, P. Portran, S. Junot, B. Allaouchiche, J. L. Fellahi, P. Guerci, B. Ergin, A. Kapucu, C. Ince, L. Cioccari, N. Luethi, M. Crisman, R. Bellomo, J. Mårtensson, C. Righy Shinotsuka, D. Fagnoul, A. Brasseur, D. Orbegozo, J. L. Vincent, J. C. Preiser, J. C. Preiser, O. Lheureux, A. Thooft, S. Brimioulle, J. L. Vincent, H. Iwasaka, S. Tahara, M. Nagamine, A. Ichigatani, A. Rugerio Cabrera, E. Monares Zepeda, J. Franco Granillo, J. S. Aguirre Sánchez, A. A. Tanaka Montoya, A. Pedraza Montenegro, G. A. Gálvez Blanco, C. M. Coronado Robles, A. Drolz, T. Horvatits, K. Roedl, K. Rutter, S. Kluge, G. C. Funk, B. Schneeweiss, V. Fuhrmann, G. Sabetian, F. Pooresmaeel, F. Zand, S. Ghaffaripour, A. Farbod, H. Tabei, L. Taheri, R. Anandanadesan, V. Metaxa, C. Teixeira, S. M. Pereira, P. Hernández-Marrero, A. S. Carvalho, M. Beckmann, C. S. Hartog, D. Schwarzkopf, A. Raadts, A. Robertsen, R. Førde, N. O. Skaga, E. Helseth, S. Honeybul, K. Ho, P. Martinez Lopez, M. Nieto Gonzalez, P. Nuevo Ortega, E. Camara Sola, T. Spasova, M. V. de la Torre-Prados, O. Kopecky, K. Rusinova, P. Waldauf, Z. Cepeplikova, M. Balik, J. Palamidessi Domínguez, P. Matia Almudevar, S. Alcántara Carmona, J. J. Rubio Muñoz, D. Palacios Castañeda, A. Naharro Abellán, P. Rodríguez Villamizar, J. Veganzones Ramos, L. Pérez Pérez, A. Pérez Lucendo, M. Camós Ejarque, A. Estella, V. Lopez Camps, M. C. Martín, N. Masnou, Bioethics work group of SEMICYUC, S. Barbosa, A. Varela, I. Palma, L. Cristina, E. Nunes, I. Pereira, G. Campello, C. Granja, R. Pande, M. Pandey, S. Varghese, M. Chanu, M. J. Van Dam, E. W. M. T. Ter Braak, A. Estella, M. Gracia, R. Viciana, M. Recuerda, L. Perez Fontaiña, B. Tharmalingam, F. Kovari, L. Rose, M. Mcginlay, R. Amin, K. Burns, B. Connolly, N. Hart, P. Jouvet, S. Katz, D. Leasa, C. Mawdsley, D. Mcauley, M. Schultz, B. Blackwood, S. Denham, R. Worrall, M. Arshad, P. Isherwood, A. Khadjibaev, D. Sabirov, A. Rosstalnaya, F. Parpibaev, V. Sharipova, G. A. Galvez Blanco, C. I. Olvera Guzman, J. S. Aguirre Sánchez, J. Franco Granillo, S. Gupta, D. Govil, S. Srinivasan, S. J. Patel, J. K. N, A. Gupta, M. Shafi, D. S. Tomar, R. Harne, D. P. Arora, N. Talwar, S. Mazumdar, Y. S. Cha, S. J. Lee, N. Tyagi, R. K. Rajput, S. Taneja, V. K. Singh, S. C. Sharma, S. Mittal, B. K. Rao, J. Ayachi, N. Fraj, S. Romdhani, A. Khedher, K. Meddeb, N. Sma, A. Azouzi, R. Bouneb, I. Chouchene, M. El Ghardallou, M. Boussarsar, R. Jennings, E. Walter, J. M. Ribeiro, I. Moniz, R. Marçal, A. C. Santos, C. Candeias, Z. Costa e Silva, S. E. Zamora Gomez, O. R. Perez Nieto, J. A. Castanon Gonzalez, A. I. Vasquez Cuellar, H. Mildh, V. Pettilä, A. M. Korhonen, S. Karlsson, T. Ala-Kokko, M. Reinikainen, S. T. Vaara, The FINNAKI Study Group, M. Zaleska-Kociecka, M. Grabowski, M. Dąbrowski, S. Wozniak, K. Piotrowska, M. Banaszewski, J. Imiela, J. Stepinska, A. González Pérez, P. Florez Ordoñez, A. Giribet, M. A. Alonso Cuervo, R. Alonso Cuervo, M. A. Rodriguez Esteban, L. Iglesias Fraile, C. Ponte Mittelbrum, G. Muñiz Albaiceta, J. Koeze, F. Keus, W. Dieperink, I. C. C. van der Horst, M. van Meurs, J. G. Zijlstra, S. Roberts, C. Hernandez Caballero, G. Isgro, D. Hall, S. Beitland, A.-M. S. Trøseid, B. S. Brusletto, B. E. Waldum-Grevbo, J. P. Berg, K. Sunde, D. García Huertas, F. Manzano, M. M. Jiménez- Quintana, A. Osuna, F. Santiago-Ruiz, C. Rodríguez-Mejías, R. Wangensteen, H. R. Jamaati, M. Masjedi, F. Zand, S. M. R. Hashemian, G. Sabetian, G. Abbasi, V. Khaloo, S. H.a. Tabei, A. Kafilzadeh, H. Haddad Bakhodaei, J. A. Diaz, R. Silva, D. J. Garcia, E. Luis, M. N. Gomez, R. Soriano, P. L. Gonzalez, I. A. Ibrahim, M. M. Rafik, A. M. Al-Ansary, M. A. Algendi, A. A. Ali, V. Fuhrmann, K. Roedl, T. Horvatits, A. Drolz, K. Rutter, D. Benten, J. Kluwe, S. Siedler, S. Kluge, I. Adedugbe, G. T. Bird, Queen Square Neuroanaesthesia and Neurocritical Care Resreach Group, R. M. Kennedy, S. Sharma, M. B. Butler, G. Yugi, B. A. Haroon, T. Witter, W. Khaliq, M. Singer, A. A. Havaldar, B. Krishna, S. Sriram, E. D. Valenzuela Espinoza, M. O. Pozo, V. S. Kanoore Edul, M. Furche, M. F. Motta, A. Risso Vazquez, P. N. Rubatto Birri, C. Ince, A. Dubin, A. Dogliotti, A. Ramos, C. Lovesio, E. Delile, R. Nevière, P.-A. Thiébaut, J. Maupoint, P. Mulder, D. Coquerel, S. Renet, J. C. do Rego, J. Rieusset, V. Richard, F. Tamion, W. Khaliq, D. T. Andreis, M. Singer, B. Smit, Y. M. Smulders, M. C. de Waard, H. M. Oudemans van Straaten, A. R. J. Girbes, E. C. Eringa, A. M. E. Spoelstra-de Man, L. Alegría, D. Soto, C. Luengo, J. Gomez, N. Jarufe, A. Bruhn, R. Castro, E. Kattan, P. Tapia, R. Rebolledo, P. Achurra, G. Ospina-Tascón, J. Bakker, G. Hernández, P. Bertini, F. Guarracino, R. Baldassarri, M. R. Pinsky, L. Alegría, M. Vera, J. Dreyse, D. Carpio, C. Henriquez, D. Gajardo, S. Bravo, R. Castro, G. Ospina-Tascón, J. Bakker, G. Hernández, S. Kim, M. Lee, S. Y. Park, S. So, H. Lee, M. B. Kačar, S. M. Kačar, I. Uddin, A. M. Belhaj, M. A. Aydın, D. Avsec, A. Kapuağası, Ç. Kaymak, L. Kovach, İ. Şencan, B. Meço, M. Özçelik, N. Ünal, C. Lazaridis, B. Jenni-Moser, M.-M. Jeitziner, M. S. Galassi, F. L. Sales, K. C. L. de Moraes, C. L. Batista, J. A. de Souza Júnior, T. B. Marcari, R. Lobato, C. S. A. A. Castro, L. M. de Souza, F. F. P. Rodrigues, N. G. Correa, A. M. Pelegrini, R. A. C. Eid, K. T. Timenetsky, D. Cazati, M. Lobato, P. S. Diniz, L. L. Rocha, A. M. Cavalheiro, N. M. Lucinio, E. R. Santos, M. Norrenberg, A. Gleize, J. C. Preiser, I. Fernández Simón, S. Alcántara Carmona, I. Lipperheide Valhonrat, J. Palamidessi Domínguez, A. Naharro Abellán, P. Matía Almudévar, F. Dávila, J. J. Rubio, A. J. Ramos, Á. J. Roldán Reina, N. Palomo López, M. Adriaensens Pérez, D. X. Cuenca Apolo, L. Martín Villén, F. M. Porras López, I. Palacios García, J. R. Naranjo Izurieta, J. J. Egea Guerrero, Renal Transplantation HUVR, S. Calvert, M. Quint, K. Adeniji, R. Young, D. D. Shevill, E. Robertson, P. Garside, E. Walter, P. Isotti, M. M. De Vecchi, A. E. Perduca, A. Negro, G. Villa, D. F. Manara, L. Cabrini, A. Zangrillo, J. F. Frencken, L. van Baal, L. M. Peelen, D. W. Donker, J. Horn, T. van der Poll, W. A. van Klei, M. J. M. Bonten, O. L. Cremer, C. E. Menard, A. Kumar, E. Rimmer, S. Doucette, A. F. Turgeon, B. L. Houston, D. S. Houston, R. Zarychanski, B. Bollen Pinto, M. Carrara, M. Ferrario, K. Bendjelid, J. Nunes, P. Diaz, G. Silva, S. Escórcio, S. Chaves, M. Jardim, N. Fernandes, M. Câmara, R. Duarte, C. A. Pereira, J. Vieira, J. J. Nóbrega, C. M. Coronado Robles, M. A. Montes de Oca-Sandoval, A. Sánchez-Rodríguez, J. G. Joya-Galeana, A. Correa-Morales, G. Camarena-Alejo, J. Aguirre-Sánchez, J. Franco-Granillo, M. Soliman, A. Al Azab, R. El Hossainy, H. Nagy, M. Nirmalan, I. A. Crippa, F. Zama Cavicchi, J. L. Vincent, J. Creteur, F. S. Taccone, A. Chaari, K. Abdel Hakim, H. Hassanein, M. Etman, M. El Bahr, K. Bousselmi, E. S. Khalil, V. Kauts, W. F. Casey, H. Imahase, Y. Sakamoto, S. Inoue, K. C. Yamada, H. Koami, T. Miike, F. Nagashima, T. Iwamura, A. Boscolo, V. Lucchetta, E. Piasentini, D. Bertini, L. Manesso, L. Spiezia, P. Simioni, C. Ori, R. B. Souza, A. M. Martins, A. M. A. Liberatore, Y. R. Kang, M. N. Nakamae, J. C. F. Vieira, I. H. J. Koh, K. Hanslin, F. Wilske, P. Skorup, J. Sjölin, M. Lipcsey, W. Jin Long, C. Er Zhen, A. Vakalos, V. Avramidis, S. H. Wu, L. J. Shyu, C. H. Li, C. H. Yu, H. C. Chen, C. H. Wang, K. H. Lin, Z. E. Aray, C. F. Gómez, A. P. Tejero, D. D. Monge, V. M. Losada, C. M. Tarancón, S. D. Cortés, A. M. Gutiérrez, T. P. Álvarez, A. Rouze, K. Jaffal, S. Six, K. Stolz, V. Cattoen, S. Nseir, J. M. Arnal, M. Saoli, D. Novotni, A. Garnero, T. Becher, V. Buchholz, D. Schädler, I. Frerichs, N. Weiler, N. Eronia, T. Mauri, S. Gatti, E. Maffezzini, A. Bronco, L. Alban, T. Sasso, C. Marenghi, G. Grasselli, A. Pesenti, G. Bellani, A. Al-Fares, L. Del Sorbo, S. Anwar, F. Facchin, S. Azad, R. Zamel, N. Ferguson, M. Cypel, S. Keshavjee, E. Fan, E. Durlinger, A. Spoelstra-de Man, B. Smit, H. J. de Grooth, A. Girbes, H. Oudemans-van Straaten, Y. Smulders, M. A. Alfaro, F. Parrilla, A. Meli, M. Pellegrini, N. Rodriguez, J. M. Goyeneche, I. Morán, H. Aguirre, J. Mancebo, S. J. H. Heines, U. Strauch, D. C. J. J. Bergmans, P. Blankman, A. Shono, D. Hasan, D. Gommers, W. Y. Chung, K. S. Lee, Y. J. Jung, J. H. Park, S. S. Sheen, K. J. Park, R. Worral, S. Denham, P. Isherwood, S. E. Rees, S. Larraza, N. Dey, S. Spadaro, J. B. Brohus, R. W. Winding, C. A. Volta, D. S. Karbing, F. Ampatzidou, A. Vlachou, G. Kehagioglou, T. Karaiskos, A. Madesis, C. Mauromanolis, N. Michail, G. Drossos, N. Saraj, S. Rijkenberg, H. M. Feijen, H. Endeman, A. A. J. Donnelly, E. Morgan, H. Garrard, H. Buckley, L. Russell, N. Haase, A. Perner, C. Goh, K. Mouyis, C. L. N. Woodward, J. Halliday, G. B. Encina, J. Ros, L. Lagunes, J. Tabernero, F. Bosch, J. Rello, D. García Huertas, F. Manzano, E. Morente-Constantin, B. Rivera-Ginés, M. Colmenero-Ruiz, A. Naharro Abellán, L. Pérez Pérez, A. Pérez Lucendo, P. Matía Almudévar, J. Palamidessi Domínguez, P. Rodríguez Villamizar, J. García Sanz, I. Fernandez Simon, B. Lobo Valbuena, S. Alcantara Carmona, M. Pais, S. Ramalingam, C. Díaz, L. Fox, M. Santafe, P. Barba, M. García, S. Leal, M. Pérez, M. L. Pérez Pérez, A. Naharro Abellán, A. Pérez Lucendo, P. Matia Almudevar, J. Palamidessi Domínguez, P. Rodríguez Villamizar, J. Veganzones, I. Fernandez Simón, B. Lobo Valbuena, N. Martínez, S. Alcántara Carmona, I. Moors, D. Mokart, F. Pène, J. Lambert, A. Kouatchet, J. Mayaux, F. Vincent, M. Nyunga, F. Bruneel, L. Laisne, A. Rabbat, C. Lebert, P. Perez, M. Chaize, A. Renault, A. P. Meert, R. Hamidfar, M. Jourdain, M. Darmon, B. Schlemmer, S. Chevret, V. Lemiale, E. Azoulay, D. Benoit, D. Martins-Branco, M. Sousa, S. Marum, M. J. Bouw, G. Galstyan, P. Makarova, E. Parovichnikova, L. Kuzmina, V. Troitskaya, N. Drize, E. Gemdzhian, V. Savchenko, H. C. Chao, E. Kılıc, B. Demiriz, M. L. Uygur, M. Sürücü, K. Cınar, A. E. Yıldırım, K. Kiss, B. Köves, V. Csernus, Z. Molnár, A. Ntantana, D. Matamis, S. Savvidou, M. Giannakou, M. Gouva, G. Nakos, V. Koulouras, S. Gaffney, E. Black, R. Docking, C. Judge, T. Drew, H. Misran, R. Munshi, L. McGovern, M. Coyle, L. Dunne, E. Deasy, P. Lavin, A. Fahy, D. M. Darcy, M. Donnelly, N. H. Ismail, T. Hall, K. Wykes, J. Jack, W. C. Ngu, P. Morgan, J. Ruiz-Ramos, P. Ramirez, M. Gordon, E. Villarreal, J. Frasquet, J. L. Poveda-Andrés, A. Castellanos, C. E. Ijssennagger, S. ten Hoorn, A. van Wijk, J. M. van den Broek, P. R. Tuinman, A. M. Elmenshawy, B. D. Hammond, G. Gibbon, T. Belcham, K. Burton, L. U. Taniguchi, F. J. S. Ramos, A. K. Momma, A. P. R. Martins-Filho, J. J. Bartocci, M. F. D. Lopes, M. H. Sad, C. M. Rodrigues, E. M. C. Pires, J. M. Vieira, M. A. Leite, L. D. Murbach, E. F. Osaku, J. Barreto, S. T. Duarte, S. Taba, D. Miglioranza, D. P. Gund, C. F. Lordani, C. R. L. M. Costa, S. M. Ogasawara, A. C. Jorge, P. A. D. Duarte, S. Spadaro, M. Capuzzo, F. Dalla Corte, S. Terranova, G. Scaramuzzo, A. Fogagnolo, S. Bertacchini, A. Bellonzi, R. Ragazzi, C. A. Volta, C. Cruz, A. Nunes, F. Seabra Pereira, I. Aragão, A. F. Cardoso, C. Santos, M. J. Malheiro, H. Castro, T. Cardoso, J. Paratz, J. Kenardy, T. Comans, F. Coyer, P. Thomas, R. Boots, N. Pereira, A. Vilas-Boas, E. Gomes, C. Dias, J. Torres, D. Carvalho, E. Molinos, C. Vales, R. Araújo, C. Cruz, A. Nunes, F. Seabra Pereira, A. F. Cardoso, C. Santos, M. J. Malheiro, H. Castro, T. Cardoso, L. Karnatovskaia, K. Philbrick, G. Ognjen, M. Clark, R. Molina Montero, J. Luján Varas, L. Alcázar Sánchez-Elvira, C. Pintado Delgado, P. Villa Díaz, B. Llorente Ruiz, A. Pardo Guerrero, J. A. Cambronero Galache, R. Jiménez, S. Rebollo, O. Alejandro, A. Fernández, S. Moreno, L. Herrera, A. Ojados, M. Galindo, J. Murcia, M. Contreras, S. Sánchez-Argente, Y. Bonilla, M. D. Rodríguez, J. M. Allegue, Ö. Cakin, H. Parlak, H. Kirca, F. Mutlu, B. Aydınlı, M. Cengiz, A. Ramazanoglu, E.-J. Jung, S.-Y. Oh, H. Lee, N. M. Filgueiras Filho, E. F. Ricaldi, S. S. Gomes, B. B. Ramos, C. V. De Lucia, C. S. Ballalai, J. C. A. Oliveira, G. P. Araponga, L. N. Veiga, C. S. Silva, M. E. Garrido, GEMINI, J. Cebrián Domenech, A. Pinos Montalvo, T. Ciges Chornet, P. Concha Martinez, M. Piñol Ribas, R. Gimeno Costa, A. Castellanos Ortega, C. Forbes, H. Prescott, A. Lal, F. A. Khan, E. G. Dela Pena, J. S. Dizon, P. P. P. Perez, C. M. J. Wong, M. Muñoz Garach, O. Moreno Romero, R. Ramirez Puerta, F. Acosta Diaz, A. M. Perez Bailon, A. Carranza Pinel, L. Peñas Maldonado, M. S. Kalaiselvan, R. L. Siva kumar, M. K. Renuka, A. S. Arun Kumar, S. De Rosa, F. Ferrari, S. Carboni Checcacci, A. Rigobello, M. Joannidis, F. Politi, A. Pellizzari, R. Bonato, A. Fernandez-Carmona, I. Macias-Guarasa, R. Gutierrez-Rodriguez, P. Martinez-Lopez, M. A. Diaz-Castellanos, EDISVAL Group, A. Fernandez-Carmona, M. Arias-Diaz, E. Aguilar-Alonso, I. Macias-Guarasa, P. Martinez-Lopez, M. A. Diaz-Castellanos, EDISVAL Group, R. N. Nikandish, V. Artemenko, A. Budnyuk, G. Li Bassi, T. Senussi, F. Idone, E. Aguilera Xiol, C. Travierso, C. Chiurazzi, A. Motos, R. Amaro, Y. Hua, L. Fernández-Barat, O. T. Ranzani, Q. Bobi, M. Rigol, A. Torres, A. Youn, J. Gyung Hwang, M. Muñoz Garach, O. Moreno Romero, M. E. Yuste Ossorio, F. Acosta Diaz, A. M. Perez Bailon, A. Carranza Pinel, L. Peñas Maldonado, C. Teixeira, H. Figueira, R. Oliveira, A. Mota, I. Aragão, O. Kamp, O. Cruciger, M. Aach, C. Kaczmarek, C. Waydhas, T. A. Schildhauer, U. Hamsen, M. Camprubí-Rimblas, L. Chimenti, R. Guillamat-Prats, T. Lebouvier, J. Bringué, J. Tijero, M. N. Gómez, L. Blanch, A. Artigas, G. Tagliabue, M. Ji, J. V. Suneby Jagers, P. A. Easton, R. B. Souza, A. M. A. Liberatore, A. M. C. R. P. F. Martins, J. C. F. Vieira, Y. R. Kang, M. N. Nakamae, I. H. J. Koh, J. Y. Hong, M. H. Shin, M. S. Park, A. Pomprapa, P. A. Pickerodt, M. B. T. Hofferberth, M. Russ, W. Braun, M. Walter, R. Francis, B. Lachmann, S. Leonhardt, I. H. J. Koh, R. B. Souza, A. M. C. R. P. F. Martins, J. C. F. Vieira, A. M. A. Liberatore, A. Landaverde-López, N. A. Canedo-Castillo, A. Esquivel-Chávez, P. C. Arvizu-Tachiquín, L. A. Sánchez-Hurtado, J. A. Baltazar-Torres, V. Cardoso, A. Krystopchuk, S. Castro, L. Melão, S. Firmino, A. Marreiros, C. Granja, S. Almaziad, A. Kubbara, W. Barnett, R. Nakity, W. Alamoudi, R. Altook, T. Tarazi, M. Fida, F. Safi, R. Assaly, A. Santini, M. Milesi, T. Maraffi, P. Pugni, D. T. Andreis, M. Cavenago, L. Gattinoni, A. Protti, G. Perchiazzi, J. B. Borges, S. Bayat, L. Porra, L. Broche, M. Pellegrini, G. Scaramuzzo, G. Hedenstierna, A. Larsson, M. Pellegrini, G. Hedenstierna, A. Roneus, M. Segelsjö, M. C. Vestito, A. Larsson, G. Perchiazzi, E. Gremo, A. Nyberg, M. Castegren, A. Pikwer, T. Yoshida, D. Engelberts, G. Otulakowski, B. Katira, M. Post, N. D. Ferguson, L. Brochard, M. B. P. Amato, B. P. Kavanagh, PLUG Working group, N. Koch, W. Huber, J. Hoellthaler, S. Mair, V. Phillip, R. M. Schmid, A. Beitz, V. Baladrón, F. J. Redondo Calvo, D. Padilla, P. Villarejo, R. Villazala, A. S. Yuste, N. Bejarano, R. J. Steenstra, H. Banierink, J. Hof, I. C. van der Horst, M. W. Nijsten, M. Hoekstra, K. Roedl, F. Sterz, T. Horvatits, K. Horvatits, A. Drolz, H. Herkner, V. Fuhrmann, M. Kott, K. Zitta, B. Brandt, C. Schildhauer, G. Elke, L. Hummitzsch, I. Frerichs, N. Weiler, M. Albrecht, L. Rey González, D. Cabestrero Alonso, A. Blandino Ortiz, R. de Pablo Sánchez, J. Higuera Lucas, K. Roedl, F. Sterz, A. Drolz, K. Horvatits, T. Horvatits, H. Herkner, V. Fuhrmann, T. Horvatits, A. Drolz, K. Roedl, K. Rutter, A. Ferlitsch, G. Fauler, M. Trauner, V. Fuhrmann, T. Horvatits, S. Pischke, L. Fischer, F. Thaiss, M. Koch, K. Bangert, V. Fuhrmann, S. Kluge, A. W. Lohse, B. Nashan, M. Sterneck, S. Faenza, A. Siniscalchi, E. Pierucci, E. Mancini, D. Ricci, C. Gemelli, A. Cuoghi, S. Magnani, M. Atti, F. Sotos, J. Cánovas, A. López, A. Burruezo, D. Torres, M. E. Herrera-Gutierrez, J. Barrueco-Francioni, D. Arias-Verdú, R. Lozano-Saez, G. Quesada-Garcia, G. Seller-Pérez, A. Figueiredo, Y. Anzola, R. Pereira, L. Bento, D. Arias-Verdú, M. Lai, M. Deiana, J. Barrueco-Francioni, M. E. Herrera-Gutierrez, G. Seller-Perez, K. Vardas, S. Ilia, A. Sertedaki, E. Charmadari, C. A. Stratakis, E. Briassouli, D. Goukos, K. Psarra, E. Botoula, S. Tsagarakis, E. Mageira, C. Routsi, S. Nanas, G. Briassoulis, A. Boscolo, D. Bertini, E. Campello, V. Lucchetta, E. Piasentini, C. M. Radu, L. Manesso, P. Simioni, C. Ori, H. Su, Y. M. Lam, K. Willis, V. Pullar, R. P. Hubner, J. L. Tsang, L. García de Guadiana-Romualdo, S. Rebollo-Acebes, P. Esteban-Torrella, R. Jiménez-Sánchez, E. Jiménez-Santos, A. Ortín-Freire, A. Hernando-Holgado, M. D. Albaladejo-Otón, L. Coelho, L. Rabello, J. Salluh, I. Martin-Loeches, A. Rodriguez, S. Nseir, P. Póvoa, TAVeM study Group, E. Varis, V. Pettilä, M. Poukkanen, S. Jacob, S. Karlsson, A. Perner, J. Takala, E. Wilkman, The FINNAKI Study Group, O. H. M. Lundberg, L. Bergenzaun, J. Rydén, M. Rosenqvist, O. Melander, M. S. Chew, E. Rodriguez-Ruiz, R. Hernández Vaquero, A. Lopez Lago, J. L. Garcia Allut, A. Estany Gestal, M. A. Garcia Gonzalez, Y. Kishihara, H. Yasuda, S. Rebollo, L. García de Guadiana-Romualdo, R. Jimenez, P. Esteban Torrella, A. Fernandez, S. Sanchez, A. Ortin, G. Li Bassi, R. Guillamat Prats, A. Artigas, E. Aguilera, D. Marti, O. T. Ranzani, M. Rigol, L. Fernandez, M. Ferrer, I. Martin-Loeches, A. Torres, V. S. Lanziotti, P. Póvoa, L. Pulcheri, M. O. Ribeiro, A. P. Barbosa, J. R. Lapa e Silva, M. Soares, J. I. F. Salluh, I. Palacios Garcia, A. Diaz Martin, M. Gil Marqués, A. Puppo Moreno, A. Gutierrez Pizarraya, J. Pachón Diaz, M. Pachón Ibañez, Y. Smani, M. Mc Connell, L. A. Zhang, R. S. Parker, I. Banerjee, G. Clermont, E. Norberg, J. Oras, A. Cuisinier, C. Maufrais, J. F. Payen, S. Nottin, G. Walther, P. Bouzat, S. Arib, F. Bilotta, R. Badenes, F. Rubulotta, S. Mirek, I. A. Crippa, B. Monfort, E. Stazi, A. Lozano Roig, J. Creteur, F. S. Taccone, S. Magnoni, M. Marando, S. Pifferi, V. Conte, F. Ortolano, M. Carbonara, G. Bertani, E. Scola, M. Cadioli, F. Triulzi, A. Colombo, N. Stocchetti, H. B. Rotzel, A. Serrano Lázaro, D. Aguillón Prada, M. Rodriguez Guimillo, C. Sanchís Piqueras, J. Romero Guia, M. García Simon, A. Mesejo Arizmendi, A. Carratalá, S. El Maraghi, A. Yehia, M. Bakry, A. Shoman, F. N. Backes, M. M. Bianchin, S. R. R. Vieira, A. de Souza, A. N. Backes, C. Klein, M. S. Kalaiselvan, M. K. Renuka, A. S. Arunkumar, A. Lozano, O. Lheureux, R. Badenes, J. L. Vincent, J. Creteur, F. S. Taccone, C. Gallaher, S. Cattlin, S. Gordon, J. Picard, V. Fontana, O. Bond, L. Nobile, J. L. Vincent, J. Creteur, F. S. Taccone, S. Mrozek, L. Delamarre, F. Capilla, T. Al-Saati, O. Fourcade, T. Geeraerts, A. M. Dominguez-Berrot, M. Gonzalez-Vaquero, M. E. Vallejo-Pascual, D. Gupta, B. D. Ivory, M. Chopra, J. McCarthy, C. L. Felderhof, C. MacNeil, F. Rubulotta, P. Waldauf, M. Maggiorini, F. Duska, on behalf of Department of Professional Development, ESICM, R. R. L. Fumis, J. M. Vieira Junior, G. Amarante, A. Skorko, S. Sanders, J. Aron, R. J. Kroll, C. Redfearn, P. Krishnan, J. E. Khalil, F. Kovari, N. Kongpolprom, V. Gulia, E. Lourenço, L. Melão, C. Duro, G. Baptista, A. Alves, B. Arminda, M. Rodrigues, A. Marreiros, C. Granja, J. Hayward, F. Baldwin, R. Gray, P. A. Katinakis, M. Stijf, M. Ten Kleij, M. Jansen-Frederiks, R. Broek, M. de Bruijne, P. E. Spronk, K. Sinha, M. Luney, K. Palmer, L. Keating, M. Abu-Habsa, R. Bahl, N. Baskaralingam, A. Ahmad, L. Kanapeckaite, P. Bhatti, S. Glace, S. Jeyabraba, H. F. Lewis, A. Kostopoulos, M. Raja, A. West, A. Ely, L. M. Turkoglu, P. Zolfaghari, J. P. Baptista, M. P. Marques, P. Martins, J. Pimentel, D. Gupta, Y. C. Su, S. Villacres, M. E. Stone, A. Parsikia, S. Medar, K. P. O’Dea, J. Porter, N. Tirlapur, J. M. Jonathan, S. Singh, M. Takata, Critical Care Research Group, M. Abu-Habsa, A. Ahmad, E. McWhirter, R. Lyon, M. L. Hariz, E. Azmi, J. Alkhan, S. Honeybul, V. Movsisyan, S. Petrikov, Z. Marutyan, I. Aliev, A. Evdokimov, E. Antonucci, T. Merz, C. Hartmann, P. Pelosi, E. Calzia, P. Radermacher, B. Nußbaum, C. Hartmann, M. Huber-Lang, M. Gröger, P. Radermacher, B. Nußbaum, B. Nußbaum, E. Antonucci, E. Calzia, P. Pelosi, P. Radermacher, C. Hartmann, E. Svoren-Jabalera, E. E. Davenport, P. Humburg, J. Knight, C. J. Hinds, I. J. Jun, W. J. Kim, E. H. Lee, G. Besch, A. Perrotti, M. Puyraveau, L. Carteron, M. Baltres, E. Samain, S. Chocron, S. Pili-Floury, E. P. Plata-Menchaca, J. Sabater-Riera, M. Estruch, E. Boza, F. Sbraga, J. Toscana-Fernández, E. Bruguera-Pellicer, J. Ordoñez-Llanos, X. L. Pérez-Fernández, SIRAKI group, P. Cavaleiro, A. Tralhão, M. Arrigo, J.-P. Lopes, M. Lebrun, B. Cholley, J. L. PerezVela, H. MarinMateos, J. J. Jimenez Rivera, M. A. Alcala Llorente, B. Gonzalez De Marcos, F. J. Gonzalez Fernandez, C. Garcia Laborda, D. Fernandez Zamora, Grupo ESBAGA, J. C. Lopez Delgado, C. Imperiali, D. Berbel-Franco, M. Dastis, G. Moreno-Gonzalez, J. Perez-Sanchez, I. Romera-Peregrina, R. Abellan-Lencina, A. Martinez-Pascual, V. Fuentes-Mila, M. Gonzalez-Romero, J. Górka, K. Górka, T. Iwaniec, M. Frołow, K. Polok, J. Fronczek, M. Kózka, J. Musiał, W. Szczeklik, A. González Pérez, P. Florez Ordoñez, A. Giribet, M. A. Alonso Cuervo, R. Alonso Cuervo, M. A. Rodriguez Esteban, L. Iglesias Fraile, C. Ponte Mittelbrum, G. Muñiz Albaiceta, F. Ampatzidou, M. Sileli, G. Kehagioglou, A. Madesis, T. Karaiskos, C. Moursia, H. Maleoglou, K. Leleki, G. Drossos, Z. Uz, Y. Ince, R. Papatella, E. Bulent, P. Guerci, C. Ince, B. De Mol, V. Vicka, D. Gineityte, D. Ringaitiene, I. Norkiene, J. Sipylaite, C. Möller, C. Fleischmann, D. O. Thomas-Rueddel, V. Vlasakov, B. Rochwerg, P. Theurer, L. Gattinoni, K. Reinhart, C. S. Hartog, A. González Pérez, J. Zanabili Al Sibai, P. Martinez Camblor, P. Alvarez Fernandez, J. M. García Gala, J. Silba Guisasola, G. Muñiz Albaiceta, T. Tamura, T. Yatabe, I. Miyajima, K. Yamashita, M. Yokoyama, F. Ampatzidou, G. Kehagioglou, E. Dalampini, M. Nastou, A. Baddour, A. Ignatiadis, T. Asteri, G. Drossos, K. E. Hathorn, S. W. Purtle, C. M. Horkan, F. K. Gibbons, K. B. Christopher, M. V. Viana, T. A. Tonietto, L. A. Gross, V. L. Costa, A. L. J. Tavares, B. O. Lisboa, R. B. Moraes, S. R. Vieira, L. V. Viana, M. J. Azevedo, G. D. Ceniccola, R. S. F. Pequeno, T. P. Holanda, V. S. Mendonça, W. M. C. Araújo, L. S. F. Carvalho, E. Segaran, L. Vickers, K. Brinchmann, I. Wignall, F. Rubulotta, I. De Brito-Ashurst, R. del Olmo, M. J. Esteban, C. Vaquerizo, R. Carreño, V. Gálvez, G. Kaminsky, B. Nieto, M. Fuentes, M. A. De la Torre, E. Torres, A. Alonso, C. Velayos, T. Saldaña, A. Escribá, J. GRIP, R. Kölegård, P. Sundblad, O. Rooyackers, Ben Naser, F. Jaziri, A. Ben Jazia, M. Barghouth, O. Hentati, W. Skouri, M. El Euch, M. Mahfoudhi, S. Turki, K. Ben Abdelghni, Ben Abdallah, B. N. M. Maha, J. Cánovas, F. Sotos, A. López, M. Lorente, A. Burruezo, D. Torres, K. Polok, A. Włudarczyk, J. Górka, A. Hałek, J. Musiał, W. Szczeklik, A. Ben Jazia, F. Jaziri, M. Bargouth, M. Bennasr, S. Turki, K. Ben Abdelghani, T. Ben Abdallah, H. J. de Grooth, I. L. Geenen, J. J. Parienti, H. M. Oudemans-van Straaten, H. P. Shum, H. S. King, K. C. Chan, W. W. Yan, J. Gonzalez Londoño, C. Lorencio Cardenas, M. Morales Pedrosa, C. Murcia Gubianas, C. Fuster Bertolin, N. Vila Batllori, J. M. Sirvent, K. Wykes, J. Jack, P. Morgan, A. Mukhopadhyay, H. Y. Chan, Y. Kowitlawakul, D. Remani, C. S. F. Leong, C. J. Henry, Z. A. Puthucheary, N. Mendsaikhan, T. Begzjav, G. Lundeg, M. Dünser, E. D. Valenzuela Espinoza, S. P. Welsh, M. F. Motta, E. Guerra, M. C. l. Zerpa, F. Zechner, M. Furche, F. Berdaguer, P. N. Rubatto Birri, A. Risso-Vazquez, A. Dubin, F. D. Masevicius, D. Greaney, A. Magee, G. Fitzpatrick, R. G. Lugo-Cob, L. A. Sánchez-Hurtado, P. C. Arvizu-Tachiquín, B. C. Tejeda-Huezo, A. A. Cano-Oviedo, J. A. Baltazar-Torres, M. S. Aydogan, T. Togal, A. Taha, H. Z. Chai, C. Kam, S. S. Yang Razali, V. Sivasamy, L. Y. Kuan, V. Poulose, M. A. Lopez Morales, S. Castro, T. Pires, L. Melão, A. Krystopchuk, I. Pereira, C. Granja, L. U. Taniguchi, E. M. C. Pires, J. M. Vieira, L. C. P. Azevedo

**Affiliations:** 1 Baylor College of Medicine, Neurology/Neurocritical Care, Houston, TX USA; 2 Erasmus Hospital, Intensive Care Medicine, Brussels, Belgium; 3 Centre Hospitalier Régional Orléans, Réaimation Médicale, Orléans, France; 4 University of British Columbia, Vancouver, Canada; 5 University of Adelaide, Discipline of Acute Care Medicine, Adelaide, Australia; 6 Royal Adelaide Hospital, Intensive Care, Adelaide, Australia; 7 Griffith University, Menzies Health Institute of Queensland, Gold Coast, Australia; 8 University of Adelaide, Discipline of Medicine, Adelaide, Australia; 9 Kingston General Hospital, Clinical Evaluation Research Unit, Kingston, Canada; 10 University of Oxford, Nuffield Department of Clinical Neurosciences, Oxford, UK; 11 Oxford University Hospitals NHS Foundation Trust, Neurosciences Intensive Care Unit, Oxford, UK; 12 King’s College Hospital, Department of Neurosurgery, London, UK; 13 King’ College London, Institute of Psychiatry, Psychology and Neuroscience, Department of Clinical Neuroscience, London, UK; 14 Rice University, Computer Science, Houston, TX USA; 15 Baylor College of Medicine, Neurology, Neurocritical Care, Houston, TX USA; 16 Baylor College of Medicine, Neurosurgery, Houston, TX USA; 17 Baylor College of Medicine, Pediatric Cardiology, Houston, TX USA; 18 University of Twente, Clinical Neurophysiology, Enschede, Netherlands; 19 Rijnstate Hospital, Neurology, Arnhem, Netherlands; 20 Medical Spectrum Twente, Clinical Neurophysiology, Enschede, Netherlands; 21 Medical Spectrum Twente, Intensive Care, Enschede, Netherlands; 22 Rijnstate Hospital, Intensive Care, Arnhem, Netherlands; 23 CHUV, Department of Intensive Care Medicine, Neuroscience Critical Care Research Group, Lausanne, Switzerland; 24 Aga Khan University Hospital, Anaesthesiology, Karachi, Pakistan; 25 Centro Hospitalar São João, Intensive Care, Porto, Portugal; 26 Faculty of Medicine, University of Porto, Medicine, Porto, Portugal; 27 Faculdade de Ciências, Universidade do Porto, Departamento de Matemática, Porto, Portugal; 28 Centro de Matemática, Universidade do Porto, Porto, Portugal; 29 The Biomedical Research Networking Center in Bioengineering, Biomaterials and Nanomedicine, Zaragoza, Spain; 30 Centro Hospitalar São João, Porto, Portugal; 31 Centro Hospitalar São João, Neurosurgery, Porto, Portugal; 32 Faculty of Medicine, University of Porto, Clinical Neurosciences, Porto, Portugal; 33 Faculdade de Ciências, Universidade do Porto, Porto, Portugal; 34 Faculty of Medicine, Alexandria University, Critical Care Medicine Department, Alexandria, Egypt; 35 Faculty of Medicine, Alexandria University, Radiology Department, Alexandria, Egypt; 36 Erasme University Hospital, Université Libre de Bruxelles, Department of Intensive Care, Brussels, Belgium; 37 University of Pittsburgh, School of Nursing, Department of Acute & Tertiary Care, Pittsburgh, Pennsylvania, PA USA; 38 University of Pittsburgh, School of Medicine, Department of Neurosurgery, Pittsburgh, PA USA; 39 University of Alexandria, Criticalcare, Alexandria, Egypt; 40 University of Alexandria, Neurology, Alexandria, Egypt; 41 University of Geneva, Department of Microbiology and Molecular Medicine (MIMOL), Geneva, Switzerland; 42 Geneva University Hospital, Division of Intensive Care, Geneva, Switzerland; 43 Medanta - The Medicity, Gurgaon, India; 44 University of Thessaly School of Medicine, Department of Critical Care Medicine, Larisa, Greece; 45 Hospital Virgen del Rocio, Sevilla, Spain; 46 Changi General Hospital, Respiratory & Critical Care Medicine, Singapore, Singapore; 47 Changi General Hospital, Clinical Trials & Research Unit, Singapore, Singapore; 48 Hacettepe University, Internal Medicine, Ankara, Turkey; 49 Hacettepe University, Intensive Care Medicine, Ankara, Turkey; 50 Hacettepe University, Internal medicine, Ankara, Turkey; 51 Hacettepe University, Intensive care medicine, Ankara, Turkey; 52 General Hospital of Thessaloniki Agios Pavlos, ICU, Thessaloniki, Greece; 53 General Hospital of Nikaia-Pireus AG. Panteleimon, Actinotherapy, Pireus, Greece; 54 General Hospital of Athens “Agioi Anargyroi, ICU, Athens, Greece; 55 General Hospital of Thessaloniki Theageneio, ICU, Thessaloniki, Greece; 56 General Hospital of Nikaia-Pireus AG. Panteleimon, Anesthesiology, Pireus, Greece; 57 General Hospital of Thessaloniki Agios Pavlos, Nursing Department Management, Thessaloniki, Greece; 58 University of Athens, Faculty of Nursing, Critical Care Directorate, Athens, Greece; 59 Université Paris Diderot, Sorbonne Paris Cité, INSERM UMR1160, Paris, France; 60 Université Paris Sud / CEA, Inserm U1184, Fontenay Aux Roses, France; 61 APHP, Lariboisiere University Hospital, Surgical ICU, Paris, France; 62 University Medical Center Hamburg-Eppendorf, Department of Anaesthesiology, Hamburg, Germany; 63 University Medical Center Hamburg-Eppendorf, Institute of Clinical Pharmacology and Toxicology, Hamburg, Germany; 64 University Medical Center Hamburg-Eppendorf, Department of Intensive Care Medicine, Hamburg, Germany; 65 University of Crete, Medical School, University Hospital, PICU, Heraklion, Greece; 66 University of Crete, Medical School, Paediatric Haematology Oncology, Heraklion, Greece; 67 University of Crete, Medical School, University Hospital, ICU, Heraklion, Greece; 68 Institute of Molecular Biology and Biotechnology (IMBB), Foundation for Research and Technology - Hellas (FORTH), Heraklion, Greece; 69 University Hospital Virgen de la Victoria / IBIMA, Clinical Chemistry Department, Málaga, Spain; 70 University Hospital Virgen de la Victoria / IBIMA, Department of Intensive Care Unit, Málaga, Spain; 71 University of Crete, Medical School, University Hospital, PICU, Heraklion, Greece; 72 University of Crete, Medical School, Paediatric Haematology Oncology, Heraklion, Greece; 73 University of Crete, Medical School, University Hospital, ICU, Heraklion, Greece; 74 Institute of Molecular Biology and Biotechnology (IMBB), Foundation for Research and Technology - Hellas (FORTH), Heraklion, Greece; 75 University Hospital Marques de Valdecilla, Santander, Spain; 76 University Hospital Vall d´Hebron, Barcelona, Spain; 77 University Hospital A Coruña, La Coruña, Spain; 78 University Hospital Puerta de Hierro, Madrid, Spain; 79 University Hospital Reina Sofia, Cordoba, Spain; 80 University Hospital 12 de Octubre, Madrid, Spain; 81 University Hospital La Fe, Valencia, Spain; 82 Nursing School of University of Cantabria, Santander, Spain; 83 Central Manchester Foundation Trust, Critical Care Department, Manchester, UK; 84 Medical University of Vienna, Anesthesiology & Intensiv Care, Vienna, Austria; 85 Cleveland Clinic, Outcomes Research, Cleveland, OH USA; 86 Quantium Medical, Barcelona, Spain; 87 University Medical Center Groningen, University of Groningen, Gronigen, Netherlands; 88 Ghent University, Ghent, Belgium; 89 Center for Biomedical Engineering Research, Barcelona, Spain; 90 Radboud University Nijmegen Medical Centre, Intensive Care, Nijmegen, Netherlands; 91 Leeds Teaching Hospitals NHS Trust, Adult Critical Care, Leeds, UK; 92 University Medical Center Utrecht, Intensive Care Center, Utrecht, Netherlands; 93 Radboud University Nijmegen Medical Centre, Intensive Care Medicine, Nijmegen, Netherlands; 94 Isala Zwolle, Geriatrics, Zwolle, Netherlands; 95 abvsciences, Vancouver, Canada; 96 VU University Medical Center, Intensive Care, Amsterdam, Netherlands; 97 VU University Medical Center, Psychiatry, Amsterdam, Netherlands; 98 Centre for Human Drugs Research, Leiden, Netherlands; 99 Tata Memorial Hospital, Mumbai, India; 100 Western Parana State University Hospital, Cascavel, Brazil; 101 Instituto Nacional de Cancerología, Department of Critical Care Medicine, Mexico, Mexico; 102 Shiraz University of Medical Sciences, Shiraz Anesthesiology and Critical Care Research Center, Shiraz, Islamic Republic of Iran; 103 Department of Epidemiology, School of Public Health - Shiraz University of Medical Sciences, Shiraz, Islamic Republic of Iran; 104 National Center for Addiction Studies, Tehran, Islamic Republic of Iran; 105 University Hospital, Vrije Universiteit Brussel, Brussels, Belgium; 106 Hospital Universitari de Bellvitge, Intensive Care Medicine, Barcelona, Spain; 107 Queen Elizabeth University hospital, Anaesthetics and Intensive Care, Glasgow, UK; 108 Barts Health NHS Trust, Renal and Transplant Medicine, London, UK; 109 Barts Health NHS Trust, Adult Critical Care, London, UK; 110 Central Manchester Foundation NHS Trust, Manchester, UK; 111 Health Education North West, Manchester, UK; 112 Hopital Universitaire Necker Enfants Malades, Pediatric Cardiac Intensice Care Unit, Paris, France; 113 Pitie-Salpetriere Hospital and Pierre and Marie Curie University, Reanimation Chirurgicale Cardio Vasculaire et Thoracique, Paris, France; 114 Arnaud de Villeneuve, Anesthésie Réanimation, Montpellier, France; 115 Hopital Laennec CHU de Nantes, Anesthesie Réanimation, Nantes, France; 116 Maison du Haut leveque CHU Bordeaux, Congenital Cardiac Surgery Unit, Bordeaux, France; 117 Clinic Pasteur, Service Anesthesie Réanimation, Toulouse, France; 118 Hopital Trousseau, Réanimation Néonatale et Polyvalente, Paris, France; 119 Hopital Arnaud de Villeneuve, Réanimation Pédiatrique et Neonatale, Montpellier, France; 120 CHU de Nantes, Pédiatric Intensive Care Unit, Nantes, France; 121 CHU Bocage, Réanimation Cardio Vasculaire et Polyvalente, Dijon, France; 122 Air Liquide Santé International, Reserach Center Paris Saclay, Paris, France; 123 Hopital Start-Tilman, Services de Soins Intensifs Généraux, Liege, Belgium; 124 Hopital Necker, Réanimation Néonatale, Paris, France; 125 American Memorial Hospital, Réanilmation Néonatale et Polyvalente, Reims, France; 126 Air Liquide Santé International, Gentilly, France; 127 Hopital Necker Enfants Malades, Pediatric Cardiac Intensive Care, Paris, France; 128 Barts Health NHS Trust, Renal and Transplant Medicine, London, UK; 129 Barts Health NHS Trust, Adult Critical Care, London, UK; 130 Yonsei University College of Medicine, Anesthesia and Pain Medicine, Seoul, Republic of Korea; 131 Seoul National University College of Medicine, Department of Surgery, Seoul, Republic of Korea; 132 Seoul National University College of Medicine, Department of Anesthesiology, Seoul, Republic of Korea; 133 Imperial College London, London, UK; 134 Royal Surrey County Hospital, ICU and SPACeR Research Group, Guildford, UK; 135 University of Surey, Guildford, UK; 136 University of Surrey, Guildford, UK; 137 Royal Surrey County Hospital, ICU and SPACeR Research Group, Intensive Care, Guildford, UK; 138 University of Southampton, Medical School, Southampton, UK; 139 Royal Surrey County Hospital, Department of Gynaecological Oncology, Guildford, UK; 140 Royal Surrey County Hospital, Intensive Care, SPACeR Research Group, Guildford, UK; 142 University of Foggia, Anesthesiology and Intensive Care, Foggia, Italy; 143 University of Ferrara, Anesthesiology and Intensive Care, Ferrara, Italy; 144 University of Foggia, Thoracic Surgery, Foggia, Italy; 145 Facultad Medicina Ciudad Real, Hospital General Universitario de Ciudad Real. Anestesiologia y Reanimacion, Ciudad Real, Spain; 146 Facultad Medicina Ciudad Real, Hospital General Universitario de Ciudad Real, Cuidados Criticos Pediatricos, Ciudad Real, Spain; 147 Facultad Medicina Ciudad Real, Hospital General Universitario de Ciudad Real, Cirugía Hepatobiliar, Ciudad Real, Spain; 148 Hospital General Universitario de Ciudad Real, Anestesiologia y Reanimación, Ciudad Real, Spain; 149 Hôpital de Bicêtre, Hôpitaux universitaires Paris-Sud, Université Paris-Sud, Service de réanimation médicale, Inserm UMR_S999, Le Kremlin-Bicêtre, France; 150 The First Affiliated Hospital of Chongqing Medical University, Department of Emergency Medicine and Critical Care Medicine, Chongqing, China; 151 Affiliated Hospital of Guizhou Medical University, Department of Critical Care Medicine, Guiyang, China; 152 Ospedale Luigi Sacco - Milano, Department of Anaesthesia and Intensive Care, Milan, Italy; 153 Politecnico di Milano, Department of Electronics information and Bioengineering, Milan, Italy; 154 Università degli studi di Milano, Department of Biomedical Science of Health, Milan, Italy; 155 Oslo University Hospital, Acute Medicine, Oslo, Norway; 156 Oslo University Hospital, Departement of Cardiology, Oslo, Norway; 157 Oslo University Hospital, Departement of Anesthesiology, Oslo, Norway; 158 Oslo University Hospital, Departement of Acute Medicin, Oslo, Norway; 159 VU University Medical Center Amsterdam, Amsterdam, Netherlands; 160 Medical Center Leeuwarden, Intensive Care, Leeuwarden, Netherlands; 161 University Medical Center Groningen, Internal Medicine / Nephrology, Groningen, Netherlands; 162 1st Medical Faculty, Charles University, Anaesthesia and Intensive Care, Prague, Czech Republic; 163 Vseobecna Fakutni Nemocnice, KARIM, Prague, Czech Republic; 164 General University Hospital, 1st Faculty of Medicine, Charles University in Prague, Prague, Czech Republic; 165 1st Medical Faculty, Charles University, Prague, Czech Republic; 166 Seoul National University Bundang Hospital, Department of Pharmacy, Seongnam-si, Republic of Korea; 167 Seoul National University, College of Pharmacy, Seoul, Republic of Korea; 168 Seoul National University Bundang Hospital, Division of Pulmonary and Critical Care Medicine, Department of Internal Medicine, Seongnam-si, Republic of Korea; 169 Austin Health, University of Melbourne, Department of Intensive Care, Heidelberg, Australia; 170 Changi General Hospital, Department of Anaesthesia and Surgical Intensive Care, Singapore, Singapore; 171 Lucerne Cantonal Hospital, Department of Intensive Care, Lucerne, Switzerland; 172 Karolinska Institutet, Section of Anaesthesia and Intensive Care Medicine, Department of Physiology and Pharmacology, Stockholm, Sweden; 173 The University of Melbourne, Parkville, Melbourne Australia; 174 TioHundra AB, Norrtälje, Sweden; 175 Swedish Poisons Information Centre, Stockholm, Sweden; 176 Department of Clinical Science and Education, Södersjukhuset, Karolinska Institutet, Stockholm, Sweden; 177 TioHundra AB, Anaesthesia and Intensive Care, Norrtälje, Sweden; 178 Clinica Universidad de Navarra, Anesthesiology, Pamplona, Spain; 179 Clinica Universidad de Navarra, Hematology, Pamplona, Spain; 180 Hospital Can Misses, Intensive Care Unit, Ibiza, Spain; 181 Hospital Can Misses, Clinical Analysis Service, Ibiza, Spain; 182 RN ICU Nurse, VU University Medical Centre, Amsterdam, Netherlands; 183 ICU Nursing staff manager, VU University Medical Center, Amsterdam, Netherlands; 184 Shiraz University of Medical Sciences, Clinical Pharmacy Department, Shiraz, Islamic Republic of Iran; 185 Shiraz University of Medical Sciences, Shiraz, Islamic Republic of Iran; 186 Eskisehir Osmangazi University Faculty of Medicine, Department of Anesthesiology and Reanimation, Division of Intensive Care, Eskisehir, Turkey; 187 Dalhousie University, Queen Elizabeth II Health Sciences Centre, Division of Neurosurgery, Halifax, Canada; 188 Dalhousie University, Critical Care, Halifax, Canada; 189 Trauma Nova Scotia, Halifax, Canada; 190 Attikon University Hospital, 2nd Department of Anaesthesiology, Athens, Greece; 191 Harokopeio University, Department of Nutrition and Dietetics, School of Health Science and Education, Athens, Greece; 192 ’Agia Olga’ Hospital, Laboratory of Pathology, Athens, Greece; 193 Aretaieion University Hospital, Laboratory of Cytology, Athens, Greece; 194 London’s Air Ambulance, Pre-hospital Emergency Medicine, London, UK; 195 Newcastle upon Tyne Hospitals NHS Foundation Trust, Perioperative and Critical Care, Newcastle upon Tyne, UK; 196 Newcastle University, Clinical Trials Unit, Newcastle upon Tyne, UK; 197 Newcastle University, Institute of Health & Society, Newcastle upon Tyne, UK; 198 City Hospitals Sunderland NHS Foundation Trust, Anaesthesia, Sunderland, UK; 199 South Tees Hospitals NHS Foundation Trust, Anaesthesia, Middlesborough, UK; 200 University Hospital of Gran Canaria Dr. Negrín, Intensive Care Unit, Las Palmas de Gran Canaria, Spain; 201 University Hospital of Gran Canaria Dr. Negrín, Pharmacy Department, Las Palmas de Gran Canaria, Spain; 202 University Hospital of Gran Canaria Dr. Negrín, Microbiology Department, Las Palmas de Gran Canaria, Spain; 203 University of Las Palmas de Gran Canaria, Mathematics and Informatics Deparment, Las Palmas de Gran Canaria, Spain; 204 CHUV, Department of Intensive Care Medicine, Neuroscience Critical Care Research Group, Lausanne, Switzerland; 205 University Hospital, Department of Anesthesia and Intensive Care Medicine, Nice, France; 206 University Hospital, Department of Anesthesia and Intensive Care Medicine, Grenoble, France; 207 Charité - Universitaetsmedizin Berlin, Berlin, Germany; 208 Berlin Institute of Health (BIH), Berlin, Germany; 209 Max Delbrück Center for Molecular Medicine (MDC), Berlin, Germany; 210 ECRC - Experimental and Clinical Research Center, Berlin, Germany; 211 Immanuel Hospital Bernau, Bernau, Germany; 212 Corporació Sanitària i Universitaria Parc Taulí, Universitat Autònoma de Barcelona, Critical Care Department, Sabadell, Spain; 213 Hospital Prof. Dr. Fernando Fonseca, Serviço de Medicina I, Amadora, Portugal; 214 Technische Universität München, II. Medizinische Klinik, Munich, Germany; 215 Cairo University Medical School, Intensive Care Department, Cairo, Egypt; 216 Faculty of Medicine, Fayoum University, Intensive Care Department, Fayoum, Egypt; 217 Cairo University Medical School, Intensive care Department, Cairo, Egypt; 218 University Hospital Erasme, Université Libre de Bruxelles, Critical Care, Brussels, Belgium; 219 University Medical Center Hamburg-Eppendorf, Center of Anaesthesiology and Intensive Care Medicine, Hamburg, Germany; 220 University Hospital of Homburg/Saar, Homburg/Saar, Germany; 221 Saarland University, Campus Homburg, Homburg/Saar, Germany; 222 Hospital Universitari Mútua de Terrassa, ICU, Barcelona, Spain; 223 Hospital Sant Joan de Dèu-Fundació Althaia Manresa, ICU, Barcelona, Spain; 224 Hospital Juan XXIII, Tarragona, Spain; 225 Hospital Universitario Marqués de Valdecilla, ICU, Santander, Spain; 226 Hospital de A Coruña, ICU, A Coruña, Spain; 227 Hospital Verge de la Cinta, ICU, Tortosa, Spain; 228 Hospital Universitario de Canarias, Tenerife, Spain; 229 Hospital Universitari Politècnic La Fe, Valencia, Spain; 230 Consorci Sanitari Integral Moisés Broggi, Barcelona, Spain; 231 Consorci Hospitalari de Terrassa, Barcelona, Spain; 232 Hospital Virgen de la Luz-SESCAM, Cuenca, Spain; 233 Hospital Medico-Quirúrgico de Jaén, Jaén, Spain; 234 Hospital Clínico de Valencia, Valencia, Spain; 235 Hospital General de Catalunya, Barcelona, Spain; 236 Hospital General de Vic, Vic, Spain; 237 Complexo Hospitalario Universitario de Ourense, Ourense, Spain; 238 Hospital General Universitario de Castellón, Castellón, Spain; 239 Hospital Universitario Rafael Méndez de Lorca, Murcia, Spain; 240 University of Toronto, Interdepartmental Division of Critical Care Medicine, Toronto, Canada; 241 Sorbonne Paris Cité, UPMC Univ Paris 06 INSERM, UMRS1158, Groupe Hospitalier Pitié-Salpêtrière Charles Foix Service de Pneumologie et Réanimation Médicale, Paris, France; 242 University of Toronto, Department of Medicine, Toronto, Canada; 243 University of Toronto, Department of Physical Therapy, Toronto, Canada; 244 Hospital Israelita Albert Einstein, Critical Care Medicine, São Paulo, Brazil; 245 Academic Medical Center, University of Amsterdam, Intensive Care, Amsterdam, Netherlands; 246 University Hospital Carl Gustav Carus, Technische Universität Dresden, 21Pulmonary Engineering Group, Department of Anesthesiology and Intensive Care Medicine, Dresden, Germany; 247 IRCCS San Martino IST, University of Genoa, Surgical Sciences and Integrated Diagnostics, Genoa, Italy; 248 Hospices Civils de Lyon, Lyon, France; 249 INSERM 955, IMRB, Créteil, France; 250 APHM, CHU Nord, Marseille, France; 251 CHU Nantes, Nantes, France; 252 C-CLIN Sud-Est, Pierre Bénite, France; 253 Université Pierre et Marie Curie, UMR_S 1158 and Hôpital Pitié-Salpêtrière, Respiratory Division and Medical ICU, Paris, France; 254 Hôpital Dupuytren, Limoges, France; 255 CHU de Clermont-Ferrand and Université d’Auvergne, Clermont-Ferrand, France; 256 Université Grenoble-Alpes and CHU Grenoble Alpes, Grenoble, France; 257 CHU d’Angers, Angers, France; 258 Erasmus Medical Center, Intensive Care, Rotterdam, Netherlands; 259 Erasmus Medical Center, Pharmacy, Rotterdam, Netherlands; 260 Erasmus Medical Center, Psychiatry, Rotterdam, Netherlands; 261 Northeastern University, School of Pharmacy, Boston, MA USA; 262 I Servizio Anestesia Rianimazione, Parma, Italy; 263 Neurochirurgia e Neurotraumatologia, Parma, Italy; 264 Nantes University Hospital, Creteil, France; 265 University Hospital of Angers, Angers, France; 266 University Hospital of Rennes, Rennes, France; 267 University Hospital of Toulouse, Toulouse, France; 268 University hospital of Montpellier, Montpellier, France; 269 University Hospital of Poitiers, Poitiers, France; 270 APHP, Beaujon, Beaujon, France; 271 APHP, Henri Mondor, Creteil, France; 272 Nantes University Hospital, Nantes, France; 273 University of Milan - Bicocca, School of Medicine and Surgery, Milan, Italy; 274 San Gerardo Hospital, Neurointensive Care, Department of Emergency and Intensive Care, Monza, Italy; 275 San Gerardo Hospital, Neurosurgical Clinic, Department of Neurosciences, Monza, Italy; 276 Hospital General de Catalunya, Intensive Care Department, Sant Cugat del Valles, Spain; 277 Hospital Universitari Mútua de Terrassa, Intensive Care Department, Terrassa, Spain; 278 AGAUR, Grup Recerca Emergent, Terrassa, Spain; 279 Hospital del Mar, Intensive Care Department, Barcelona, Spain; 280 Hospital Universitari de Tarragona Joan XXIII, Intensive Care Department, Tarragona, Spain; 281 Academic Medical Center, University of Amsterdam, Amsterdam, Netherlands; 282 St James’s University Hospital, Dublin, Ireland; 283 Hospital São Francisco Xavier, Lisbon, Portugal; 284 Hospital Clinic, Barcelona, Spain; 285 Autonomous University of Barcelona, Barcelona, Spain; 286 Lille University Hospital, ICU, Lille, France; 287 Trinity Centre for Health Sciences, St James’s University Hospital, Critical Care Medicine, Dublin, Ireland; 288 Centro Hospitalar de Lisboa Ocidental, São Francisco Xavier Hospital, ICU, Lisbon, Portugal; 289 D’Or Institute for Research and Education, Rio de Janeiro, Brazil; 290 Joan XXIII University Hospital, Institut d’Investigació Sanitària Pere Virgili, Tarragona, Spain; 291 VU University Medical Center Amsterdam, Intensive Care, Amsterdam, Netherlands; 292 ST Elisabeth Twee Steden Hospital, Intensive Care, Tilburg, Netherlands; 293 Medisch Spectrum Twente, Intensive Care, Enschede, Netherlands; 294 University Medical Center Groningen, Intensive Care, Amsterdam, Netherlands; 295 University Medical Center Utrecht, Intensive Care, Utrecht, Netherlands; 296 Ulss 15 Alta Padovana, Anesthesia and Intensive Care Unit, Camposampiero, Italy; 297 Ulss 15 Alta Padovana, Epidemiology Unit, Camposampiero, Italy; 298 Hospital Universitari de Bellvitge, Intensive Care, L’ Hospitalet de Llobregat, Spain; 299 Hospital Universitari de Bellvitge, Cardiac Surgery, L’ Hospitalet de Llobregat, Spain; 300 University of Southampton, Southampton, UK; 301 University Hospital Southampton, Southampton, UK; 302 Royal Surrey County Hospital, ICU and SPACeR Research Group, Guildford, UK; 303 University of Surrey, Guildford, UK; 304 Department of Intensive Care Medicine and Surrey Peri-Operative Anaesthesia and Critical Care Collaborative Research Group (SPACER), Guildford, UK; 305 Department of Surgery, Royal Surrey County Hospital NHS Foundation Trust, Guildford, UK; 306 Department of Anaesthesia, Royal Surrey County Hospital NHS Foundation Trust, Guildford, UK; 307 Department of Surgery, Royal Surrey County Hospital NHS Foundation Trust, Guilford, UK; 308 Department of Intensive Care Medicine and Surrey Peri-Operative Anaesthesia and Critical Care Collaborative Research Group (SPACER) and Department of Anaesthesia, Guildford, UK; 309 Wakayama Medical University, Department of Emergency and Critical Care Medicine, Wakayama, Japan; 310 Tohoku University Hospital Emergency Center, Division of Emergency and Critical Care Medicine, Sendai, Japan; 311 Hyogo College of Medicine, Department of Clinical Epidemiology, Nishinomiya, Japan; 312 Osaka City University Graduate School of Medicine, Department of Trauma and Critical Care Medicine, Osaka, Japan; 313 Osaka City General Hospital, Emergency and Urgent Medical Care Center, Osaka, Japan; 314 Hyogo College of Medicine, Emergency and Critical Care Center, Nishinomiya, Japan; 315 Saga University Hospital, Advanced Emergency and Critical Care Center, Saga, Japan; 316 National Hospital Organization Kyoto Medical Center, Department of Emergency Medicine, Critical Care, Kyoto, Japan; 317 Sapporo Medical University, Department of Emergency Medicine, Sapporo, Japan; 318 Yamaguchi Grand Medical Center, Department of Anesthesiology, Yamaguchi, Japan; 319 Hyogo College of Medicine, Division of General Medicine, Department of Internal Medicine, Nishinomiya, Japan; 320 Hirosaki University Graduate School of Medicine, Department of Disaster and Emergency Medicine, Hirosaki, Japan; 321 University of Copenhagen, Rigshospitalet, Department of Intensive Care, København, Denmark; 322 Copenhagen Trial Unit, Center for Clinical Intervention Research, Copenhagen, Denmark; 323 University of Copenhagen, Rigshospitalet, Section for Transfusion Medicine, Copenhagen, Denmark; 324 University Medical Center Utrecht, Department of Intensive Care, Utrecht, Netherlands; 325 Julius Center for Health Sciences and Primary Care University Medical Center Utrecht, Department of Medical Humanities, Utrecht, Netherlands; 326 Julius Center for Health Sciences and Primary Care University Medical Center Utrecht, Department of Epidemiology, Utrecht, Netherlands; 327 University Hospitals Birmingham NHS Trust, Critical Care, Birmingham, UK; 328 University Hospital Birmingham, Critical Care, Birmingham, UK; 329 HIGA Gral San Martin, La Plata, Argentina; 330 Hospital de Trauma F Abete, Municipio Islas Malvinas, Buenos Aires, Argentina; 331 Sanatorio Anchorena, Buenos Aires, Argentina; 332 Hospital Municipal Chivilcoy, Chivilcoy, Argentina; 333 Instituto Fleni, Escobar, Argentina; 334 Instituto De Neurología Cognitiva-INECO, Buenos Aires, Argentina; 335 Hospital Escuela de Agudos Dr. Ramón Madariaga, Posadas, Argentina; 336 HIGA Prof. Dr. Luis Güemes, Haedo, Argentina; 337 Hospital Misericordia, Cordoba, Argentina; 338 Hospital Italiano Buenos Aires, Buenos Aires, Argentina; 339 Hospital Francisco López Lima, Gral Roca, Argentina; 340 Alta Complejidad en Red, Hospital El Cruce, Dr. Néstor Carlos Kirchner, Florencio Varela, Argentina; 341 HZGA Mi Pueblo Florencio Varela, Florencio Varela, Argentina; 342 Hospital Español, Mendoza, Argentina; 343 Clinica Pueyrredon, Mar del Plata, Argentina; 344 Hospital Zonal Bariloche, Bariloche, Argentina; 345 Clinica CEMEP, Ushuaia, Argentina; 346 Chia Nan University of Pharmacy and Science, Recreation and Health-Care Management, Tainan, Taiwan, Province of China; 347 Chi-Mei Medical Center, Intensive Care Medicine, Tainan, Taiwan, Province of China; 348 Chi-Mei Medical Center, Liouying District, Intensive Care Medicine, Tainan, Taiwan, Province of China; 349 Chi Mei Medical Center, Internal Medicine, Tainan, Taiwan, Province of China; 350 Brigham and Women’s Hospital, Department of Medicine, Boston, MA USA; 351 Massachusetts General Hospital, Pulmonary and Critical Care Medicine, Boston, MA USA; 352 Brigham and Women’s Hospital, Renal Division, Boston, MA USA; 353 Brigham and Women’s Hospital, Channing Division of Network Medicine, Boston, MA USA; 354 VU University Medical Center Amsterdam, Department of Nutrition and Dietetics, Internal Medicine, Amsterdam, Netherlands; 355 Amsterdam University of Applied Sciences, Amsterdam, Netherlands; 356 Brigham and Women’s Hospital, Department of Nutrition, Boston, USA; 357 Brigham and Women’s Hospital, Department of Surgery, Boston, USA; 358 Brigham And Women’s Hospital, Renal Division, Boston, USA; 359 Brigham and Women’s Hospital, Channing Division of Network Medicine, Boston, USA; 360 The First Affiliated Hospital of Sun Yat-Sen University, Guangzhou, China; 361 Clínica Las Condes, Medicina Intensiva, Santiago, Chile; 362 Universidad de Chile, Program of Anatomy and Developmental Biology, Institute of Biomedical Sciences, Santiago, Chile; 363 Universidad Católica de Chile, Departamento de Fisiología, Santiago, Chile; 364 Aalborg University, Respiratory and Critical Care Group (Rcare), Department of Health Science and Technology, Aalborg, Denmark; 365 Aalborg University Hospital, Department of Anesthesiology, Aalborg, Denmark; 366 Charité - Universitaetsmedizin Berlin, Berlin, Germany; 367 Berlin Institute of Health (BIH), Berlin, Germany; 368 John Radcliffe Hospital, Adult Intensive Care Unit, Oxford, UK; 369 Ministry of Health, Labour and Welfare, Government of Japan, Tuberculosis and Infectious Diseases Control, Tokyo, Japan; 370 Tsukuba Medical Center Hospital, Emergency Medicine and Critical Care Medicine, Tsukuba, Japan; 371 University of Tsukuba, Health Services Research, Tsukuba, Japan; 372 National Defense Medical College, Traumatology and Emergency Medicine, Tokorozawa, Japan; 373 Japan Community Healthcare Organization, Tokyo, Japan; 374 Dalhousie University, Nova Scotia Trauma Program, Halifax, Canada; 375 Dalhousie University, Undergraduate Medical Education, Halifax, Canada; 376 Yonsei University College of Medicine, Department of Surgery, Seoul, Republic of Korea; 377 Hospital Clínico Universitario, Intensive Care Unit, Santiago de Compostela, Spain; 378 Fundación Ramón Dominguez, Unidad de Epidemiología e Investigación Clínica, Santiago de Compostela, Spain; 379 Instituto de Investigaciones Sanitarias, Grupo de Genética y Biología del Desarrollo de las Enfermedades Renales, Santiago de Compostela, Spain; 380 Universitaetsklinikum Jena, Department of Anesthesiology and Intensive Care, Jena, Germany; 381 Universitaetsklinikum Jena, Center for Sepsis Control and Care (CSCC), Jena, Germany; 382 KCMH, Nursing Department, Bangkok, Thailand; 383 Mahidol University, Faculty of Public Health, Bangkok, Thailand; 384 Núcleo de Ensino e Pesquisa Hospital da Cidade, Intensive Care, Salvador, Brazil; 385 Universidade Salvador - UNIFACS, Núcleo de Pesquisa Clínica, Salvador, Brazil; 386 Queen Elizabeth University Hospital, Anaesthetics and Intensive Care, Glasgow, UK; 387 University of Adelaide, Discipline of Acute Care Medicine, Adelaide, Australia; 388 Royal Adelaide Hospital, Intensive Care Unit, Adelaide, Australia; 389 University of Adelaide, Discipline of Medicine, Adelaide, Australia; 390 Royal Adelaide Hospital, Department of Endocrinology, Adelaide, Australia; 391 Flinders University, Department of Critical Care Medicine, Adelaide, Australia; 392 Flinders Medical Centre, Department of Intensive Care Medicine, Adelaide, Australia; 393 Lyell McEwin Hospital, Department of Intensive Care Medicine, Adelaide, Australia; 394 Baker IDI Heart and Diabetes Institute, Melbourne, Australia; 395 Kochi Medical School, Department of Anesthesiology and Intensive Care Medicine, Nankoku, Japan; 396 Tokai University Hachioji Hospital, Department of Emergency and Critical Care Medicine, Hachioji, Japan; 397 Kochi Medical School, Integrated Center for Advanced Medical Technologies, Nankoku, Japan; 398 Kobe University Hospital, Department of Anesthesiology, Kobe, Japan; 399 Royal Adelaide Hospital, Department of Critical Care Services, Adelaide, Australia; 400 University of Adelaide, Discipline of Acute Care Medicine, Adelaide, Australia; 401 University of Adelaide, Discipline of Medicine, Adelaide, Australia; 402 Royal Adelaide Hospital, Endocrine and Metabolic Unit, Adelaide, Australia; 403 Flinders University, Department of Critical Care Medicine, Adelaide, Australia; 404 Flinders Medical Centre, Department of Intensive Care Medicine, Adelaide, Australia; 405 Lyell McEwin Hospital, Department of Intensive Care Medicine, Adelaide, Australia; 406 Baker IDI Heart and Diabetes Institute, Melbourne, Australia; 407 Kobe University Hospital, Anesthesiology, Kobe, Japan; 408 Royal Adelaide Hospital, Intensive Care Unit, Adelaide, Australia; 409 University of Adelaide, Discipline of Acute Care Medicine, Adelaide, Australia; 410 Addenbrooke’s Hospital, Neurosciences Critical Care Unit, Cambridge, UK; 411 University of Adelaide, NHMRC Centre for Research Excellence, Adelaide, Australia; 412 University of Sheffield, Academic Unit of Diabetes, Endocrinology and Metabolism, Sheffield, UK; 413 University of Adelaide, Discipline of Medicine, Adelaide, Australia; 414 Anesthesiology and Critical Care Medicine, Hôpital Cardiologique, Hospices Civils de Lyon, Bron, France; 415 VetAgro Sup, Ecole vétérinaire de Lyon, Marcy l’Etoile, France; 416 Anesthesiology and Critical Care Medicine, Centre Hospitalier Lyon Sud, Hospices Civils de Lyon, Pierre Benite, France; 417 Academic Medical Center, University of Amsterdam, Department of Translational Physiology, Amsterdam, Netherlands; 418 University Hospital of Nancy, Departement of Anaesthesiology and Critical Care Medicine, Vandoeuvre-Les-Nancy, France; 419 University of Lorraine, INSERM U1116, Vandoeuvre-Les-Nancy, France; 420 Science Faculty, University of Istanbul, Department of Biology, Istanbul, Turkey; 421 Austin Health, University of Melbourne, Department of Intensive Care, Heidelberg, Australia; 422 Lucerne Cantonal Hospital, Department of Intensive Care, Lucerne, Switzerland; 423 Cattinara Hospital, Trieste University School of Medicine, Department of Perioperative Medicine, Intensive Care and Emergency, Trieste, Italy; 424 Australian and New Zealand Intensive Care Research Centre (ANZIC-RC), Monash University, Department of Epidemiology and Preventive Medicine, Melbourne, Australia; 425 Karolinska Institutet, Section of Anaesthesia and Intensive Care Medicine, Department of Physiology and Pharmacology, Stockholm, Sweden; 426 Université Libre de Bruxelles (ULB) - Hôpital Erasme, Intensive Care, Brussels, Belgium; 427 Erasme University Hospital, Université Libre de Bruxelles, Brussels, Belgium; 428 Oita Almeida Hospital, Intensive Care Unit, Oita, Japan; 429 Oita Almeida Hospital, Anesthesiology, Oita, Japan; 430 The Americam British Cowdray Medical Center, Critical Care Department’Dr. Mario Shapiro’, Mexico City, Mexico; 431 Medical University of Vienna, Internal Medicine III, Gastroenterology and Hepatology, Vienna, Austria; 432 Medical University Center Hamburg-Eppendorf, Intensive Care Medicine, Hamburg, Germany; 433 Otto-Wagner-Spital, Respiratory and Critical Care Medicine, Vienna, Austria; 434 Shiraz University of Medical Sciences, Trauma Research Center, Shiraz, Islamic Republic of Iran; 435 Shiraz University of Medical Sciences, Shiraz, Islamic Republic of Iran; 436 Shiraz University of Medical Sciences, Anesthesiology and Critical Care Research Center, Shiraz, Islamic Republic of Iran; 437 King’s College Hospital (Denmark Hill), Intensive Care Medicine, London, UK; 438 Universidade Católica Portuguesa, Instituto de Bioética, Porto, Portugal; 439 Centro Hospitalar do Porto, Hospital de Santo António, UCIP-Departamento de Anestesia e Cuidados Intensivos, Porto, Portugal; 440 Universidade do Porto, Instituto de Ciências Biomédicas Dr. Abel Salazar, Porto, Portugal; 441 Servicio Canario de Salud, Porto, Portugal; 442 Jena University Hospital, Anaesthesiologyy and Intensive Care, Jena, Germany; 443 Jena University Hospital, Center for Sepsis Control and Care, Jena, Germany; 444 Oslo University Hospital, Anesthesiology and critical care, Oslo, Norway; 445 University of Oslo, Center of medical ethics, Oslo, Norway; 446 Oslo University Hospital, Neurosurgery, Oslo, Norway; 447 Sir Charles Gairdner Hospital, Neurosurgery, Perth, Australia; 448 Royal Perth Hospital, Perth, Australia; 449 Virgen de la Victoria Hospital, Intensive Care, Málaga, Spain; 450 General University Hospital, 1st Faculty of Medicine, Charles University in Prague, Department of Anaesthesia and Intensive Care Medicine, Prague, Czech Republic; 451 3rd Medical Faculty, Charles University, Department of Anaesthesia and Intensive Care Medicine, Prague, Czech Republic; 452 Hospital Universitario Puerta de Hierro Majadahonda, Madrid, Spain; 453 Hospital del SAS de Jerez, Intensive Care Unit, Jerez de la Frontera, Spain; 454 Hospital de Sagunto, Intensive Care Unit, Sagunto, Spain; 455 Hospital Universitario Torrejón de Ardoz, Intensive Care Unit, Madrid, Spain; 456 Hospital Universitario de Girona, Intensive Care Unit, Girona, Spain; 457 Algarve Hospital Center, Faro Unit, Emergency and Intensive Care Department, Faro, Portugal; 458 Algarve Hospital Center, Portimão Unit, Emergency and Intensive Care Department, Portimão, Portugal; 459 University of Algarve, Department of Biomedical Sciences and Medicine and CINTESIS-UALG, Faro, Portugal; 460 Faculty of Medicine of Porto, CINTESIS, Porto, Portugal; 461 BLK Superspeciality Hospital, Critical Care Medicine, New Delhi, India; 462 Lady Hardinge Medical College, Anaesthesiology, New Delhi, India; 463 University Medical Center Utrecht, IC Center, Utrecht, Netherlands; 464 University Medical Center Utrecht, Educational Center, Utrecht, Netherlands; 465 University Medical Center Utrecht, Internal Medicine, Utrecht, Netherlands; 466 Hospital del SAS de Jerez, Intensive Care Unit, Jerez de la Frontera, Spain; 467 Hospital del SAS de Jerez, Jerez de la Frontera, Spain; 468 North Middlesex University Hospital, Anaesthesia/ITU, London, UK; 469 North Middlesex University Hospital, ICU, London, UK; 470 University of Toronto, Toronto, Canada; 471 Royal Victoria Hospital, Belfast, UK; 472 SickKids Hospital, Toronto, Canada; 473 St Michael’s Hospital, Toronto, Canada; 474 St Thomas’ Hospital, London, UK; 475 University of Montreal, Montreal, Canada; 476 Children’s Hospital of Eastern Ontario, Ottawa, Canada; 477 London Health Sciences Centre, London, Canada; 478 Queen’s University, Belfast, UK; 479 University of Amsterdam, Amsterdam, Netherlands; 480 University Hospital Birmingham, Critical Care Medicine, Birmingham, UK; 481 Uzbekistan Research Center of Emergency Medicine, Tashkent, Uzbekistan; 482 Tashkent Institute of Postgraduate Medical Education, Tashkent, Uzbekistan; 483 ABC Medical Center, Intensive Care Unit, Mexico City, Mexico; 484 Medanta- The Medicity, Gurgaon, India; 485 Yonsei University, Wonju College of Medicine, Emergency Medicine, Wonju, Republic of Korea; 486 Yonsei University, Wonju College of Medicine. Department of Internal Medicine, Wonju, Republic of Korea; 487 Sir Ganga Ram Hospital, Delhi, India; 488 Farhat Hached University Hospital, Medical Intensive Care Unit, Sousse, Tunisia; 489 Ibn Al Jazzar Faculty of Medicine, University of Sousse, Department of Community Medicine, Sousse, Tunisia; 490 Research Laboratory N° LR14ES05, Interactions of the Cardiopulmonary System Ibn Al Jazzar Faculty of Medicine. University of Sousse, Sousse, Tunisia; 491 Royal Surrey County Hospital, Intensive Care, Guildford, UK; 492 University Hospital of Santa Maria, CHLN, Intensive Care Department, Lisbon, Portugal; 493 Hospital Juarez de México, ICU, Mexico City, Mexico; 494 University of Helsinki and Helsinki University Hospital, Helsinki, Finland; 495 Bern University Hospital and University of Bern, Bern, Switzerland; 496 Tampere University Hospital, Tampere, Finland; 497 Oulu University Hospital, Medical Research Center Oulu, Oulu, Finland; 498 North Karelia Central Hospital, Joensuu, Finland; 499 Institute of Cardiology, Cardiac Intensive Care Clinic, Warsaw, Poland; 500 Institute of Cardiology, Department of Valvular Heart Diseases, Warsaw, Poland; 501 Institute of Cardiology, Department of Interventional Cardiology and Angiology, Warsaw, Poland; 502 Institute of Cardiology, Cardiac Surgery and Transplantology Clinic, Warsaw, Poland; 503 Kozminski University, Department of Quantitative Methods & Information Technology, Warsaw, Poland; 504 Miedzyleski Hospital, 1st Department of Internal Medicine, Warsaw, Poland; 505 Hospital Universitario Central de Asturias, Oviedo, Spain; 506 UMCG, Department of Critical Care, Groningen, Netherlands; 507 Royal Brompton and Harefield NHS Foundation Trust, London, UK; 508 Oslo University Hospital Ullevål, Department of Anaesthesiology, Oslo, Norway; 509 University of Oslo, Oslo, Norway; 510 Oslo University Hospital Ullevål, Department of Medical Biochemistry, Oslo, Norway; 511 Oslo University Hospital Ullevål, Department of Nephrology, Oslo, Norway; 512 Complejo Hospitalario de Granada, Granada, Spain; 513 Complejo Hospitalario de Jaén, Jaén, Spain; 514 Shaheed Beheshti University of Medical Sciences, Tehran, Islamic Republic of Iran; 515 Shiraz University of Medical Sciences, Anesthesia and Intensive Care Department, Shiraz, Islamic Republic of Iran; 516 Anesthesiology and Critical Care Research Center, Shiraz University of Medical Sciences, Shiraz, Islamic Republic of Iran; 517 Shiraz University of Medical Sciences, Shiraz, Islamic Republic of Iran; 518 Instituto Mexicano del Seguro Social, Intensive Care Unit, Leon, Mexico; 519 Universidad de Guanajuato, Intensive Care Unit, Leon, Mexico; 520 Faculty of Medicine - Ain Shams University, Anaesthesia, Intensive Care and Pain, Cairo, Egypt; 521 Faculty of Medicine - Ain Shams University, Clinical Pathology, Cairo, Egypt; 522 National Hepatology and Tropical Medicine Research Inistitute, Intensive Care, Cairo, Egypt; 523 University Medical Center Hamburg-Eppendorf, Intensive Care Medicine, Hamburg, Germany; 524 University Medical Center Hamburg-Eppendorf, Internal Medicine 1, Hamburg, Germany; 525 University College London Hospitals NHS Foundation Trust, London, UK; 526 Dalhousie University, Medical School, Halifax, Canada; 527 Dalhousie University, Critical Care Medicine, Halifax, Canada; 528 Dalhousie University, Anesthesiology, Pain Management and Perioperative Medicine, Halifax, Canada; 529 Dalhousie University, General Internal Medicine, Halifax, Canada; 530 UCL, Bloomsbury Institute of Intensive Care Medicine, London, UK; 531 St. Johns Medical College, Critical Care Medicine, Bangalore, India; 532 Sanatorio Otamendi y Miroli, Servicio de Terapia Intensiva, Buenos Aires, Argentina; 533 Facultad de Ciencias Médicas, Universidad Nacional de La Plata, Cátedra de Farmacología Aplicada, La Plata, Argentina; 534 Academic Medical Center, University of Amsterdam, Translational Physiology, Amsterdam, Netherlands; 535 Instituto Cardiovascular Rosario, Rosario, Argentina; 536 Grupo Oroño, Unidad de Epidemiologia Clinica, Rosario, Argentina; 537 Sanatorio Parque, Terapia Intensiva y Cuidados Criticos, Rosario, Argentina; 538 University of Rouen, INSERM U1096, Rouen, France; 539 INSERM U995, Lille, France; 540 University of Rouen, Service Commun d’Analyse Comportementale (SCAC), Rouen, France; 541 University of Lyon, UMR INSERM U1060, Lyon, France; 542 University of Rouen, INSERM U1096 - Service de Réanimation Médicale CHU Rouen, Rouen, France; 543 UCL, Bloomsbury Institute of Intensive Care Medicine, London, UK; 544 Dipartimento di Fisiopatologia Medico-Chirurgica e dei Trapianti, Università degli Studi di Milano, Milan, Italy; 545 VU University Medical Center, Intensive Care, Amsterdam, Netherlands; 546 VU University Medical Center, Internal Medicine, Amsterdam, Netherlands; 547 VU University Medical Center, Physiology, Amsterdam, Netherlands; 548 Pontificia Universidad Catolica de Chile, Facultad de Medicina, Departamento de Medicina Intensiva, Santiago, Chile; 549 Hospital Clínico, Universidad de Chile, Unidad de Pacientes Críticos, Santiago, Chile; 550 Universidad de Passo Fundo, Passo Fundo, Brazil; 551 Pontificia Universidad Catolica de Chile, Departamento de Cirugía Digestiva, Santiago, Chile; 552 Hospital de la Florida, Unidad de Pacientes Críticos, Santiago, Chile; 553 Fundación Valle del Lili, Intensive Care Medicine Department, Cali, Colombia; 554 University Hospital of Pisa, Department of Anaesthesia and Critical Care Medicine, Cardiothoracic and Vascular Anaesthesia, Pisa, Italy; 555 University of Pittsburgh, Department of Critical Care Medicine, Pittsburgh, USA; 556 Pontificia Universidad Catolica de Chile, Facultad de Medicina, Departamento de Medicina Intensiva, Santiago, Chile; 557 Fundación Valle del Lili, Intensive Care Medicine Department, Cali, Colombia; 558 Chonbuk National University Hospital, Nursing, Jeonjusi, Republic of Korea; 559 Chonbuk National Universiy Hospital, Internal Medicine, Jeonjusi, Republic of Korea; 562 Clinic for Cardiac Surgery Clinical Center of Serbia, ICU, Belgrade, Serbia; 563 Clinic for Cardiac Surgery Clinical Center of Serbia, Surgery, Belgrade, Serbia; 564 Princess Alexandra Hospital, Anaesthetics, Harlow, UK; 565 Southend University Hospital, Intensive Care, Essex, UK; 566 Ministry of Health, Ankara, Turkey; 567 Ministry of Health, Lubiana, Slovenia; 568 European Union, Budapest, Hungary; 569 Ankara University, Ankara, Turkey; 570 Baylor College of Medicine, Neurology, Neurocritical Care, Houston, USA; 571 BCM, Center for Ethics, Houston, USA; 572 Universitätsklinik für Intensivmedizin, Bern, Switzerland; 573 Hospital Israelita Albert Einstein, São Paulo, Brazil; 574 Hospital Albert Einstein, Pacientes Graves, São Paulo, Brazil; 575 Hospital Israelita Albert Einstein, São Paulo, Brazil; 576 Erasme University Hospital, Université Libre de Bruxelles, Department of Intensive Care, Brussels, Belgium; 577 Hospital Universitario Puerta de Hierro Majadahonda, Majadahonda, Spain; 578 Hospital Universitario Virgen del Rocío, UCI, Sevilla, Spain; 579 Hospital Universitario Virgen del Rocío, Sevilla, Spain; 580 Queen Alexandra Hospital, Critical Care, Portsmouth, UK; 581 Sheffield Hallam University, Faculty of Health & Wellbeing, Sheffield, UK; 582 Royal Surrey County Hospital NHS Foundation Trust, Department of Anaesthesia, Guildford, UK; 583 Royal Surrey County Hospital NHS Foundation Trust, Department of Intensive Care, Guildford, UK; 584 IRCCS San Raffaele Scientific Institute, Milan, Italy; 585 IRCCS San Raffaele Scientific Institute, Milan, Italy; 586 University Medical Center Utrecht, Intensive Care Center, Utrecht, Netherlands; 587 University Medical Center Utrecht, Julius Center for Health Sciences and Primary Care, Utrecht, Netherlands; 588 Academic Medical Center, University of Amsterdam, Intensive Care, Amsterdam, Netherlands; 589 Academic Medical Center, University of Amsterdam, Center for Experimental and Molecular Medicine, Amsterdam, Netherlands; 590 Academic Medical Center, University of Amsterdam, Center for Infection and Immunity, Amsterdam, Netherlands; 591 Academic Medical Center, University of Amsterdam, Division of Infectious Diseases, Amsterdam, Netherlands; 592 University Medical Center Utrecht, Department of Anesthesiology, Utrecht, Netherlands; 593 University Medical Center Utrecht, Department of Medical Microbiology, Utrecht, Netherlands; 594 University of Manitoba, Internal Medicine, Winnipeg, Canada; 595 University of Manitoba, Critical Care, Winnipeg, Canada; 596 University of Manitoba, Infectious Disease, Winnipeg, Canada; 597 University of Manitoba, Haematology, Winnipeg, Canada; 598 University of Dalhousie, Halifax, Canada; 599 Universite Laval, Anesthesia, Quebec City, Canada; 600 Universite Laval, Critical Care, Quebec City, Canada; 601 Geneva University Hospitals, Department of Pharmacology, Anaesthesia and Intensive Care, Geneva, Switzerland; 602 Politecnico di Milano, Department of Electronics, Information and Bioengineering (DEIB), Milan, Italy; 603 Hospital Dr. Nélio Mendonça, Intensive Care, Funchal, Portugal; 604 Universidad Autonoma de México, Ciudad de México, Mexico; 605 Cairo University, Critical Care, Cairo, Egypt; 606 University of Manchester, Critical Care, Manchester, UK; 607 Université Libre de Bruxelles (ULB) - Hôpital Erasme, Brussels, Belgium; 608 King Hamad University Hospital, Intensive Care, Muharaq, Bahrain; 609 King Hamad University Hospital, Muharaq, Bahrain; 610 Saga University Hospital, Saga City, Japan; 611 UOC Anesthesia and Intensive Care Unit, Hospital of Padova, Padova, Italy; 612 Thrombotic and Hemorrhagic Diseases Unit, Department of Medicine, University of Padua, Padova, Italy; 613 UOC Anesthesia and Intensive Care Unit, Department of Medicine-DIMED, Padova, Italy; 614 Federal University of São Paulo, Morphology and Genetics, São Paulo, Brazil; 615 Biological Institute of São Paulo, São Paulo, Brazil; 616 Federal University of São Paulo, Surgery, São Paulo, Brazil; 617 Uppsala University, Department of Surgical Sciences, Uppsala, Sweden; 618 Uppsala University, Department of Medical Sciences, Uppsala, Sweden; 619 Ruijin Hospital, Shanghai Jiaotong University School of Medicine, Shanghai, China; 620 Xanthi General Hospital, ICU, Xanthi, Greece; 621 Changhua Christian Hospital, Medicine, Changhua, Taiwan, Province of China; 622 Changhua Christian Hospital, Pharmacy, Changhua, Taiwan, Province of China; 623 Yuanlin Christian Hospital, Changhua, Taiwan, Province of China; 624 Hospital Virgen de la Concha, Medicina Intensiva, Zamora, Spain; 625 Critical Care Center, University Hospital of Lille, Lille, France; 626 Hopital Sainte Musse, Toulon, France; 627 Hamilton Medical, Research and Development, Bonaduz, Switzerland; 628 University Medical Center Schleswig-Holstein, Campus Kiel, Anesthesiology and Intensive Care Medicine, Kiel, Germany; 629 San Gerardo Hospital, Monza, Italy; 630 Fondazione IRCCS Ca’ Granda Ospedale Maggiore Policlinico, Milan, Italy; 631 University of Milan-Bicocca, Health Sciences, Monza, Italy; 632 Adult Critical Care Medicine Residency Program, Interdepartmental Division of Critical Care Medicine University of Toronto, Toronto, Canada; 633 University of Toronto, Department Critical Care Medicine, Toronto, Canada; 634 Toronto Lung Transplant Program, University of Toronto, Toronto, Canada; 635 VU University Medical Center Amsterdam, Intensive Care, Amsterdam, Netherlands; 636 VU University Medical Center Amsterdam, Internal Medicine, Amsterdam, Netherlands; 637 Hospital de la Santa Creu i Sant Pau, Servei de Medicina Intensiva, Barcelona, Spain; 638 AOU Cittá della Salute e della Scienza di Torino, Department of Anesthesia and Critical Care, Torino, Italy; 639 Maastricht University Medical Centre + , Maastricht, Netherlands; 640 Erasmus Medical Center, Adult Intensive Care, Rotterdam, Netherlands; 641 Ajou University, Pulmonology, Suwon, Republic of Korea; 642 Ajou University, Suwon, Republic of Korea; 643 University Hospital Birmingham, Critical Care Medicine, Birmingham, UK; 644 Aalborg University, Respiratory and Critical Care Group (Rcare), Department Health Science and Technology, Aalborg, Denmark; 645 Herning Hospital, Department of Anesthesiology, Herning, Denmark; 646 University of Ferrara, Department of Morphology, Experimental Medicine and Surgery. Section of Anaesthesia and Intensive Care. Arcispedale Sant’ Anna, Ferrara, Italy; 647 Mermaid Care A/S, Aalborg, Denmark; 648 G. Papanikolaou Hospital, Cardiac Surgery ICU, Thessaloniki, Greece; 649 G. Papanikolaou Hospital, Cardiac Surgery, Thessaloniki, Greece; 650 OLVG, ICU, Amsterdam, Netherlands; 651 Royal Bolton NHS Trust, Anaesthesia and Intensive Care, Bolton, UK; 652 Rigshospitalet, Copenhagen University Hospital, Copenhagen, Denmark; 653 Hvidovre Hospital, Copenhagen, Denmark; 654 Milton Keynes University Hospital NHS Foundation Trust, Department of Critical Care, Milton Keynes, UK; 655 University of Oxford, Wellcome Trust Centre for Human Genetics, Oxford, UK; 656 Milton Keynes University Hospital NHS Foundation Trust, Blood Borne Virus Clinic, Milton Keynes, UK; 657 Vall d´Hebrón University Hospital, Intensive Care Unit, Barcelona, Spain; 658 Hospital Vall d’Hebron, Clinical Oncology Department, Barcelona, Spain; 659 Vall d´Hebrón University Hospital, Clinical Oncology Department, Barcelona, Spain; 660 Vall d´Hebrón University Hospital, Clinical Haematology Department, Barcelona, Spain; 661 Vall d´Hebrón Research Institute, Barcelona, Spain; 662 Complejo Hospitalario de Granada, Granada, Spain; 663 Hospital Universitario Puerta de Hierro Majadahonda, Majadahonda, Spain; 664 Heart of England NHS Foundation Trust, Intensive Care, Birmingham, UK; 665 Vall d’Hebron University Hospital, Intensive Care, Barcelona, Spain; 666 Vall d’Hebron University Hospital, Hematology, Barcelona, Spain; 667 Hospital Universitario Puerta de Hierro Majadahonda, Intensive Care, Madrid, Spain; 668 Hospital Universitario Puerta de Hierro Majadahonda, Madrid, Spain; 669 Ghent University Hospital, Hematology, Ghent, Belgium; 670 Institut Paoli Calmettes, Marseilles, France; 671 Hôpital Cochin, Paris, France; 672 Hôpital Saint Louis, Paris, France; 673 Centre Hospitalier Universitaire d’Angers, Angers, France; 674 Groupe Hospitalier Pitié-Salpêtrière Charles Foix, Paris, France; 675 Hôpital d’Avicenne, Bobigny, France; 676 Centre Hospitalier de Roubaix, Roubaix, France; 677 Hôpital Mignot, Versailles, France; 678 Centre Hospitalier Départemental site de Montaigu, La Roche Sur Yon, France; 679 Hôpital Brabois, Nancy, France; 680 Hôpital de la Cavale Blanche, Brest, France; 681 Institut Jules Bordet, ULB, Brussels, Belgium; 682 Hôpital Albert Michallon, Grenoble, France; 683 Hôpital Roger Salengro, Lille, France; 684 Paris Diderot Sorbonne University, Paris, France; 685 Ghent University Hospital, Ghent, Belgium; 686 Instituto Português de Oncologia de Lisboa FG, EPE, Oncologia Médica, Lisbon, Portugal; 687 Instituto Português de Oncologia de Lisboa FG, EPE, Unidade de Cuidados Intensivos e Intermédios, Lisbon, Portugal; 688 National Research Center for Hematology, ICU, Moscow, Russian Federation; 689 National Research Center for Hematology, Hematology Department, Moscow, Russian Federation; 690 National Research Center for Hematology, BMT Department, Moscow, Russian Federation; 691 National Research Center for Hematology, Physiology of Hematopoiesis Lab, Moscow, Russian Federation; 692 National Research Center for Hematology, Biostatistics Department, Moscow, Russian Federation; 693 National Research Center for Hematology, Moscow, Russian Federation; 694 Chi Mei Medical Center, Intensive Care Medicine, Tainan, Taiwan, Province of China; 695 Sehitkamil State Hospital, Anesthesia and Reanimation, Gaziantep, Turkey; 696 Sehitkamil State Hospital, General Surgery, Gaziantep, Turkey; 697 Sehitkamil State Hospital, Brain Surgery, Gaziantep, Turkey; 698 Sehitkamil State Hospital, Internal Medicine, Gaziantep, Turkey; 699 University of Szeged, Szeged, Hungary; 700 Jahn Ferenc Teaching Hospital, Budapest, Hungary; 701 University of Pécs, Department of Anatomy, Pécs, Hungary; 702 Papageorgiou General Hospital, ICU, Thessaloniki, Greece; 703 AHEPA University Hospital, ICU, Thessaloniki, Greece; 704 Technological Educational Institutes of Ipeirus, Ioannina, Greece; 705 University Hospital of Ioannina, ICU, Ioannina, Greece; 706 Queen Elizabeth University Hospital, NHS GG&C, Department of Critical Care, Glasgow, UK; 707 AMNCH Tallaght Hospital, Nephrology, Dublin, Ireland; 708 AMNCH Tallaght Hospital, Intensive Care, Dublin, Ireland; 709 Trinity College, School of Pharmacy and Pharmaceutical Sciences, Dublin, Ireland; 710 AMNCH Tallaght Hospital, Pharmacy, Dublin, Ireland; 711 King Fhad University Hospital, Department of Pharmacy, Al-Khobar, Saudi Arabia; 712 East Surrey Hospital, ICU, Redhill, UK; 714 Hospital Universitari i Politècnic la Fe, Intensive Care Unit, Valencia, Spain; 715 Hospital Universitari i Politècnic la Fe, Microbiology, Valencia, Spain; 716 Hospital Universitari i Politècnic la Fe, Pharmacy, Valencia, Spain; 717 VU University Medical Center, Intensive Care, Amsterdam, Netherlands; 718 Zaans Medisch Centrum, Intensive Care, Zaandam, Netherlands; 719 Alexandria University, Critical Care Medicine, Alexandria, Egypt; 720 Nottingham University Hospitals NHS Trust, Department of Anaesthesia and Critical Care, Nottingham, UK; 721 Notingham University Hospitals NHS Trust, Department of Anaesthesia and Critical Care, Nottingham, UK; 722 Education and Research Institute, Hospital Sírio-Libanês, Intensive Care Unit, São Paulo, Brazil; 723 Hospital das Clínicas, Universidade de São Paulo, Emergency Medicine Discipline, São Paulo, Brazil; 724 Education and Research Institute, Hospital Sírio-Libanês, Intensive Care Unit, Sao Paulo, Brazil; 725 Western Parana State University Hospital, Cascavel, Brazil; 726 University of Ferrara, Department of Morphology, Surgery and Experimental Medicine, Ferrara, Italy; 727 UCIP, Hospital Santo António, Porto, Portugal; 728 University of Queensland, Brisbane, Australia; 729 Griffith University, Brisbane, Australia; 730 Queensland University of Technology, Brisbane, Australia; 731 Royal Brisbane & Womens Hospital, Brisbane, Australia; 732 Hospital Pedro Hispano, Matosinhos, Portugal; 733 UCIP, Hospital Santo António, Porto, Portugal; 734 Mayo Clinic, Rochester, USA; 735 Hospital Universitario Príncipe de Asturias, Alcalá de Henares, Madrid, Spain; 736 Hospital Universitario Santa Lucía, Intensive Care Unit, Cartagena, Spain; 737 Akdeniz University, Antalya, Turkey; 738 Seoul National University Hospital, Anesthesiology and Critical Care, Seoul, Republic of Korea; 739 Seoul National University Hospital, Surgery, Seoul, Republic of Korea; 740 Núcleo de Ensino e Pesquisa Hospital da Cidade, Intensive Care, Salvador, Brazil; 741 Universidade Salvador - UNIFACS, Núcleo de Pesquisa Clínica, Salvador, Brazil; 742 Hospital Universitario y Politécnico La Fe, ICU, Valencia, Spain; 743 Nottingham University Hospitals NHS Trust, Adult Critical Care, Nottingham, UK; 744 Ng Teng Fong General Hospital (Jurong Healthcare), Intensive Care, Singapore, Singapore; 745 Ng Teng Fong General Hospital (Jurong Healthcare), Clinical Research Unit, Singapore, Singapore; 746 Complejo Hospitalario de Granada, ICU, Granada, Spain; 747 Sri Ramachandra University, Department of Critical Care Medicine, Chennai, India; 748 Sri Ramachandra University, Department of Anaesthesiology, Chennai, India; 749 International Renal Research Institute of Vicenza (IRRIV), Department of Nephrology, Dialysis and Transplantation, Vicenza, Italy; 750 San Bortolo Hospital, Department of Anesthesiology and Intensive Care, San Bortolo Hospital, Vicenza, Italy; 751 San Bortolo Hospital, Department of Anesthesiology and Intensive Care, San Bortolo Hospital, Vicenza, Italy; 752 Medical University Innsbruck, Division of Intensive Care and Emergency Medicine, Department of Internal Medicine, Innsbruck, Austria; 753 Hospital Virgen de las Nieves, Intensive Care, Granada, Spain; 754 Hospital Regional, Intensive Care, Malaga, Spain; 755 Hospital Virgen de la Victoria, Malaga, Spain; 756 Hospital Santa Ana, Motril, Spain; 757 Hospital Virgen de las Nieves, Intensive Care, Granada, Spain; 758 Hospital Santa Ana, Motril, Spain; 759 Hospital Infanta Margarita, Intensive Care, Cabra, Spain; 760 Hospital Regional, Intensive Care, Malaga, Spain; 761 Hospital Virgen de la Victoria, Malaga, Spain; 762 Shiraz University Of Medical Sciences, Shiraz, Islamic Republic of Iran; 763 Odessa National Medical University, ICU, Odessa, Ukraine; 764 Odessa National Medical University, Odessa, Ukraine; 765 Hospital Clinic, Pulmonary and Critical Care Medicine, Barcelona, Spain; 766 Hospital Clinic, Barcelona, Spain; 767 University of Milan, Fisiopatologia Medico-Chirurgica e dei Trapianti, Milan, Italy; 768 Chungnam National University Hospital, Anesthesiology and Pain Medicine, Daejon, Republic of Korea; 769 Complejo Hospitalario de Granada, ICU, Granada, Spain; 770 Centro Hospitalar do Porto, Hospital de Santo António, UCIP-Departamento de Anestesiologia, Cuidados Intensivos e Emergência, Porto, Portugal; 771 Universidade do Porto, Instituto de Ciências Biomédicas Dr. Abel Salazar, Porto, Portugal; 772 Berufsgenossenschaftliches Universitätsklinikum Bergmannsheil, Surgical Clinic and Polyclinic, Bochum, Germany; 773 Berufsgenossenschaftliches Universitätsklinikum Bergmannsheil, Surgical Clinic - Department of Spinal Cord Injuries, Bochum, Germany; 774 Fundació Parc Taulí, Sabadell, Spain; 775 CIBERES, Sabadell, Spain; 776 Surgical Intensive Care Unit Ponchaillou University Hospital, Rennes, France; 777 Critical Care Center - Corporació Sanitària i Universitària Parc Taulí, Sabadell, Spain; 778 University of Calgary, Critical Care Medicine, Calgary, Canada; 779 Federal University of São Paulo, Morphology and Genetics, São Paulo, Brazil; 780 Federal University of São Paulo, Surgery, São Paulo, Brazil; 781 Biological Institute of São Paulo, São Paulo, Brazil; 782 Hallym University Medical Center, Chuncheon, Republic of Korea; 783 Yonsei University College of Medicine, Seoul, Republic of Korea; 784 Chair for Medical Information Technology, RWTH Aachen University, Aachen, Germany; 785 Department of Anesthesiology and Intensive Care Medicine, Campus Charité Mitte and Campus Virchow-Klinikum, Charité-University Medicine, Berlin, Germany; 786 Fritz Stephan GmbH, Gackenbach, Germany; 787 Federal University of São Paulo, Surgery, São Paulo, Brazil; 788 Federal University of São Paulo, Morphology and Genetics, São Paulo, Brazil; 789 Biological Institute of São Paulo, São Paulo, Brazil; 790 Hospital de Especialidades, Centro Médico La Raza, IMSS, Intensive Care Unit, Mexico, Mexico; 791 University of Algarve, Department of Biomedical Sciences and Medicine, Faro, Portugal; 792 Centro Hospitalar do Algarve, Hospital de Faro, Emergency and Intensive Care Department, Faro, Portugal; 793 Faculty of Medicine of Porto, CINTESIS, Porto, Portugal; 794 University of Toledo Medical Center, Toledo, United States; 795 Fondazione IRCCS Ca’ Granda Ospedale Maggiore Policlinico, Dipartimento di Anestesia, Rianimazione ed Emergenza Urgenza, Milan, Italy; 796 Università degli Studi di Milano, Dipartimento di Fisiopatologia Medico-Chirurgica e dei Trapianti, Milan, Italy; 797 Fondazione IRCCS Ca’ Granda Ospedale Maggiore Policlinico, Centro di Ricerche Chirurgiche Precliniche, Milan, Italy; 798 Uppsala University, Department of Medical Sciences - Hedenstierna Laboratory, Uppsala, Sweden; 799 Università di Bari Aldo Moro, Department of Emergency and Organ Transplant, Bari, Italy; 800 Université de Picardie Jules Verne, Laboratoire Peritox EA -INERIS, Amiens, France; 801 University of Helsinki, Department of Physics, Helsinki, Finland; 802 European Synchrotron Radiation Facility, Grenoble, France; 803 University of Ferrara, Morphology, Surgery and Experimental Medicine, Ferrara, Italy; 804 Uppsala University, Hedenstierna Laboratory, Department of Surgical Sciences, Uppsala, Sweden; 805 Bari University, Department of Emergency and Organ Transplant, Bari, Italy; 806 Uppsala University, Hedenstierna Laboratory, Department of Medical Sciences, Uppsala, Sweden; 807 Uppsala University, Section of Radiology, Department of Surgical Sciences, Uppsala, Sweden; 808 Mälarsjukhuset, Clinic for Anesthesiology and Intensive Care, Eskilstuna, Sweden; 809 Uppsala University, Centre for Clinical Research Sörmland, Uppsala, Sweden; 810 Karolinska University Hospital, Department of Anaesthesia, Intensive Care and Surgical Services, Stockholm, Sweden; 811 Hospital for Sick Children, University of Toronto, Physiology and Experimental Medicine, Toronto, Canada; 812 Hospital for Sick Children, University of Toronto, Departments of Critical Care Medicine and Anesthesia, Toronto, Canada; 813 University of Toronto, Interdepartmental Division of Critical Care Medicine, Toronto, Canada; 814 University Health Network and Mount Sinai Hospital, Division of Respirology, Department of Medicine, Toronto, Canada; 815 University of Toronto Saint Michael’s Hospital and Keenan Research Centre, Toronto, Canada; 816 Heart Institute (Incor) Hospital das Clínicas da Faculdade de Medicina da Universidade de São Paulo, Laboratório de Pneumologia LIM-09, Disciplina de Pneumologia, São Paulo, Brazil; 817 Technische Universität München, II. Medizinische Klinik, Munich, Germany; 818 Hospital General Universitario de Ciudad Real, Anestesiologia y Reanimación, Ciudad Real, Spain; 819 Facultad Medicina Ciudad Real, Hospital General Universitario de Ciudad Real, Anestesiologia y Reanimacion, Ciudad Real, Spain; 820 Facultad de Medicina Ciudad Real, Hospital General Universitario de Ciudad Real, Ciudad Real, Spain; 821 Facultad de Medicina Ciudad Real, Hospital General Universitario de Ciudad Real, Cirugía Hepatobiliar, Ciudad Real, Spain; 822 Facultad de Medicina Ciudad Real, Hospital General Universitario de Ciudad Real, Cuidados Criticos Pediatricos, Ciudad Real, Spain; 823 University Medical Center Groningen, University of Groningen, Department of Anesthesiology, Groningen, Netherlands; 824 University Medical Center Groningen, University of Groningen, Department of Intensive Care, Groningen, Netherlands; 825 University Medical Center Groningen, University of Groningen, Department of Surgery, Groningen, Netherlands; 826 Medical University Center Hamburg-Eppendorf, Department for Intensive Care Medicine, Hamburg, Germany; 827 Medical University of Vienna, Department of Emergency Medicine, Vienna, Austria; 828 UKSH, Campus Kiel, Department of Anaesthesiology and Intensive Care Medicine, Kiel, Germany; 829 UKSH, Campus Kiel, Institute for Clinical Chemistry, Kiel, Germany; 830 Hospital Ramón y Cajal, Madrid, Spain; 831 Medical University Center Hamburg-Eppendorf, Department for Intensive Care Medicine, Hamburg, Germany; 832 Medical University of Vienna, Department of Emergency Medicine, Vienna, Austria; 833 University Medical Center Hamburg-Eppendorf, Hamburg, Germany; 834 Medical University of Vienna, Vienna, Austria; 835 Medical University of Graz, Graz, Austria; 836 University Medical Center Hamburg-Eppendorf, Hamburg, Germany; 837 Teaching Hospital Policlinico S.Orsola-Malpighi, Department of Surgery, Intensive Care and Transplantation, Bologna, Italy; 838 Teaching Hospital Policlinico S.Orsola-Malpighi, Nephrology, Dialysis, Hypertension, Bologna, Italy; 839 Science and Technology Park for Medicine, Mirandola, Italy; 840 Aferetica s.r.l, Bologna, Italy; 841 Morales Meseguer Hospital, Murcia, Spain; 842 Complejo Universitario Carlos Haya, Málaga, Spain; 843 Hospital Curry Cabral, CHLC, UCIP7, Lisbon, Portugal; 844 Complejo Universitario Carlos Haya, Málaga, Spain; 845 University of Perugia, Department of Clinical and Experimental Medicine, Perugia, Italy; 846 National and Kapodistrian University of Athens, First Critical Care Department, Athens, Greece; 847 University Hospital, University of Crete, Pediatric Intensive Care Unit, Heraklion, Greece; 848 National and Kapodistrian University of Athens, Division of Endocrinology, Metabolism and Diabetes, First Department of Pediatrics, Athens, Greece; 849 Eunice Kennedy Shriver National Institute of Child Health and Human Development (NICHD), National Institutes of Health, Section on Endocrinology and Genetics, Bethesda, USA; 850 National and Kapodistrian University of Athens, 1st Department of Internal-Medicine - Propaedeutic, Athens, Greece; 851 Evangelismos Hospital, Immunology-Histocompatibility Department, Athens, Greece; 852 Evangelismos Hospital, Department of Endocrinology - Diabetes, Athens, Greece; 853 UOC Anesthesia and Intensive Care Unit, Hospital of Padova, Padova, Italy; 854 Thrombotic and Hemorrhagic Diseases Unit, Department of Medicine, University of Padua, Padova, Italy; 855 UO Anesthesia and Intensive Care Unit, Department of Medicine-DIMED, Padova, Italy; 856 McMaster University, Michael G. DeGroote School of Medicine, St. Catharines, Canada; 857 Niagara Health System, St. Catharines, Canada; 858 University of St. Andrews, Bute School of Medicine, St. Andrews, UK; 859 McMaster University, Department of Medicine, Hamilton, Canada; 860 Hospital Universitario Santa Lucía, Biochemistry Department, Cartagena, Spain; 861 Hospital Universitario Santa Lucía, Critical Care Unit, Cartagena, Spain; 862 Hospital São Francisco Xavier, Unidade de Cuidados Intensivos Polivalente, Lisbon, Portugal; 863 D’Or Institute for Research and Education, Rio de Janeiro, Brazil; 864 St James’s Hospital, Department of Clinical Medicine, Dublin, Ireland; 865 Hospital Joan XXIII, Tarragona, Spain; 866 Centre Hospitalier Régional Universitaire de Lille, Lille, France; 867 Helsinki University/Helsinki University Hospital, Division of Anesthesia, Intensive Care and Pain Medicine, Hus, Finland; 868 Vaasa Central Hospital, Vaasa, Finland; 869 Helsinki University/Helsinki University Hospital, Division of Anesthesia, Intensive Care and Pain Medicine, Helsinki, Finland; 870 Inselspital, Bern University Hospital and University of Bern, Bern, Switzerland; 871 Lapland Central Hospital, Rovaniemi, Finland; 872 Inselspital, Bern University Hospital and University of Bern, Bern, Finland; 873 Tampere University Hospital, Tampere, Finland; 874 Rigshospitalet, Copenhagen University Hospital, Copenhagen, Denmark; 875 Skane University Hospital, Intensive and Perioperative Care, Malmö, Sweden; 876 Skane University Hospital, Infectious Diseases, Malmö, Sweden; 877 Lund University, Internal Medicine, Malmö, Sweden; 878 Linkoping University, Anesthesiology and Intensive Care, Linkoping, Sweden; 879 Hospital Clínico Universitario, Intensive Care Unit, Santiago de Compostela, Spain; 880 Fundación Ramón Dominguez, Unidad de Epidemiología e Investigación Clínica, Santiago de Compostela, Spain; 881 Instituto de Investigaciones Sanitarias, Grupo de genética y Biología del Desarrollo de las Enfermedades Renales, Santiago de Compostela, Spain; 882 Musashino Red Cross Hospital, Tokyo, Japan; 883 Hospital General Universitario Santa Lucia, Intensive Care, Cartagena, Spain; 884 Hospital General Universitario Santa Lucia, Biochemistry Department, Cartagena, Spain; 885 Hospital General Universitario Santa Lucia, Cartagena, Spain; 886 Hospital Clinic, Pulmonary and Critical Care Medicine, Barcelona, Spain; 887 Corporació Parc Taulí, Universitat Autonoma de Barcelona, Parc Taulí, Sabadell, Spain; 888 Hospital Clinic, Barcelona, Spain; 889 Trinity College, Dublin, Ireland; 890 Federal University of Rio de Janeiro, Paediatric Intensive Care Unit, Rio de Janeiro, Brazil; 891 D’Or Institute for Research and Education, Intensive Care Research, Rio de Janeiro, Brazil; 892 Nova Medical School (Universidade Nova de Lisboa), Intensive Care Unit, Lisbon, Portugal; 893 Rede D’Or São Luiz, Paediatric Intensive Care Unit - Rio’s D’Or Hospital, Rio de Janeiro, Brazil; 894 D’Or Institute for Research and Education, Paediatric Research, Rio de Janeiro, Brazil; 895 Federal University of Rio de Janeiro, Paediatric Intensive Care Unit and Paediatric Department, Rio de Janeiro, Brazil; 896 Federal University of Rio de Janeiro, Internal Medicine Department, Medical School, Rio de Janeiro, Brazil; 897 Hospital Virgen del Rocio, Seville, Spain; 898 Hospital Virgen del Rocio, Sevilla, Spain; 899 University of Pittsburgh, Department of Chemical Engineering, Pittsburgh, USA; 900 University of Pittsburgh, CRISMA Center, Department of Critical Care Medicine, Pittsburgh, USA; 901 University of Pittsburgh, McGowan Institute for Regenerative Medicine, Pittsburgh, USA; 902 University of Pittsburgh, Department of Bioengineering, Pittsburgh, USA; 903 University of Gothenburg, Institue of Clinical Sciences, Gothenburg, Sweden; 904 Centre Hospitalier Universitaire de Grenoble, Pôle Anesthésie Réanimation, La Tronche, France; 905 Department of Medicine, University of Fribourg, Laboratory of Integrative Cardiovascular and Metabolic Physiology, Fribourg, Switzerland; 906 Grenoble Institut des Neurosciences, INSERM U1216, Grenoble, France; 907 Grenoble Alpes Université, Grenoble, France; 908 Avignon University, LAPEC EA4278, Avignon, France; 909 Hopital Erasme, Department of Intensive Care, Brussels, Belgium; 910 Sapienza University, Department of Anesthesia and Intensive Care, Rome, Italy; 911 Hospital Clinic Universitari, Department of Anesthesia and Intensive Care, Valencia, Spain; 912 St Mary Hospital and Charing Cross Hospital Imperial College NHS Trust, Department of Anaesthesia and Intensive Care Medicine, London, UK; 913 CHU Dijon, Department of Anesthesia and Intensive Care, Dijon, France; 914 Fondazione IRCCS Ca’ Granda Ospedale Maggiore Policlinico, Milan, Italy; 915 Milan University, Milan, Italy; 916 Philips Healthcare, Milan, Italy; 917 Hospital Clinic Universitari Valencia, Valencia, Spain; 918 Faculty of Medicine - Beni Suef University, Critical Care Department, Cairo, Egypt; 919 Faculty of Medicine Cairo University, Critical Care Department, Cairo, Egypt; 920 Fever Hospital, Critical Care Department, Shebin Elkom, Menoufia, Egypt; 921 Hospital de Clínicas de Porto Alegre, Intensive Care Medicine, Neurology, Porto Alegre, Brazil; 922 Hospital de Clínicas de Porto Alegre, Neurology, Porto Alegre, Brazil; 923 Hospital de Clínicas de Porto Alegre, Intensive Care Medicine, Porto Alegre, Brazil; 924 Hospital de Clínicas de Porto Alegre, Porto Alegre, Brazil; 925 Sri Ramachandra University, Department of Critical Care Medicine, Chennai, India; 926 Sri Ramachandra University, Department of Anesthesiology, Chennai, India; 927 Erasme Hospital, Intensive Care, Brussels, Belgium; 928 Hospital Clínico de Valencia, Anesthesia and Intensive Care, Valencia, Spain; 929 Imperial College Healthcare NHS Trust, Anaesthesia, London, UK; 930 Imperial College Healthcare NHS Trust, Critical Care, London, UK; 931 Erasme University Hospital, Université Libre de Bruxelles, Intensive Care, Brussels, Belgium; 932 University Hospital of Toulouse, Department of Anaesthesia and Intensive Care, Toulouse, France; 933 University Toulouse 3 Paul Sabatier, Equipe “Modélisation de l’agression traumatique”, Toulouse, France; 934 University Paul Sabatier, Unité INSERM/UPS US006, Toulouse, France; 935 Complejo Asistencial Universitario de Leon, Intensive Care Unit, Leon, Spain; 936 Hospital Rio Carrion, Intensive Care Unit, Palencia, Spain; 937 Universidad de Leon, Economia y Estadistica, Leon, Spain; 938 All India Institute of Medical Sciences, Neurosciences Centre, Delhi, India; 939 Torbay Hospital, Intensive Care, Torquay, UK; 940 Derriford Hospital, Intensive Care, Plymouth, UK; 941 Queen Elizabeth University Hospital, NHS GG&C, Anaesthetics & ICM, Glasgow, UK; 942 Imperial College London, London, UK; 943 3rd School of Medicine, Charles University in Prague, Anaesthesia and Critical Care, Prague, Czech Republic; 944 UniversitätsSpital Zürich, Medical ICU, Zürich, Switzerland; 945 Examinations Committee, ESICM, Brussels, Belgium; 946 Hospital Sírio-Libanês, Intensive Care Unit, São Paulo, Brazil; 947 Bristol Royal Infirmary, Bristol, UK; 948 Medway Maritime Hospital, Anaesthesia and Intensive Care, Gillingham, UK; 949 North Middlesex University Hospital, Critical Care, London, UK; 950 North Middlesex University Hospital, ICU, London, UK; 951 King Chulalongkorn Memmorial Hospital, Thai Red Cross and Chulalonkorn University, Critical Care and Pulmonary Medicine, Bangkok, Thailand; 952 Good Hope Hospital, Anaesthesia + Intensive care, Sutton Coldfield, UK; 953 Centro Hospitalar do Algarve, Hospital de Faro, Emergency and Intensive Care Department, Faro, Portugal; 954 Biomedical Sciences and Medical Department, Algarve University, Faro, Portugal; 955 University of Medicine of Porto, CIDES/CINTESIS, Porto, Portugal; 956 University of Medicine of Faro, Faro, Portugal; 957 Brighton and Sussex University Trust, ICU, Brighton, UK; 958 Gelre Hospitals, Apeldoorn, Netherlands; 959 VU University Medical Center, Department of Public and Occupational Health, Amsterdam, Netherlands; 960 Royal Berkshire Hospital, Reading, UK; 961 Barts Health NHS Trust, Critical Care, London, UK; 962 Kings College Hospital NHS Foundation Trust, London, UK; 963 Kent, Surrey and Sussex Air Ambulance, Surrey, UK; 964 King’s College London, London, UK; 965 King’s College London, London, UK; 966 Barts and the London School of Anaesthesia, The Royal London, Intensive Care Medicine, London, UK; 967 The Royal London Hospital, London, UK; 968 GKT Medical School, London, UK; 969 Centro Hospitalar e Universitário de Coimbra (CHUC), Intensive Care Medicine, Coimbra, Portugal; 970 Centro Hospitalar e Universitário de Coimbra (CHUC), Bioestatistics, Coimbra, Portugal; 971 All India Institute of Medical Sciences, Neurosciences Centre, Delhi, India; 972 Dalin Tzu Chi Hospital, Buddhist Tzu Chi Medical Foundation, Chiayi, Taiwan, Province of China; 973 Jacobi Medical Center/Albert Einstein College of Medicine, Pediatrics, Bronx, USA; 974 Albert Einstein College of Medicine & Children’s Hospital at Montefiore, Pediatrics, Division of Pediatric Critical Care, Bronx, USA; 975 Imperial College London, London, UK; 976 Barts Health NHS Trust, Critical Care, London, UK; 977 Kent, Surrey and Sussex Air Ambulance, HEMS, Surrey, UK; 978 King’s College London, School of Medicine, London, UK; 979 Kent, Surrey and Sussex Air Ambulance, Surrey, UK; 980 Edinburgh Royal Infirmary, Emergency Department, Edinburgh, UK; 981 BDF Hospital, Anaesthesia ICU, Manama, Bahrain; 982 BDF Hospital, Manama, Bahrain; 983 Sir Charles Gairdner Hospital, Neurosurgery, Perth, Australia; 984 N.V. Sklifosovsky Research Institute of Emergency Medicine of the Moscow Healthcare Department, Moscow, Russian Federation; 985 N.V. Sklifosovsky Research Institute of Emergency Medicine of the Moscow Healthcare Department, Moscow, Russian Federation; 986 University of Genoa, Department of Surgical Sciences and Integrated Diagnostics, IRCCS San Martino IST, Genoa, Italy; 987 University Hospital Ulm, Institute of Anesthesiological Pathophysiology and Process Development, Ulm, Germany; 988 University Hospital Ulm, Anesthesiology, Ulm, Germany; 989 Department of Anesthesiology, University Hospital, Ulm, Germany; 990 Department of Traumatology, Hand-, Plastic- and Reconstructive Surgery, University Hospital, Ulm, Germany; 991 Institute of Anesthesiological Pathophysiology and Process Development, Ulm, Germany; 992 University Hospital Ulm, Anesthesiology, Ulm, Germany; 993 University of Genoa, Department of Surgical Sciences and Integrated Diagnostics, IRCCS San Martino IST, Genoa, Italy; 994 University Hospital Ulm, Institute of Anesthesiological Pathophysiology and Process Development, Ulm, Germany; 995 Queen Mary University of London, Queen’s Hospital University Hospital Trust, London, UK; 996 Oxford University Hospitals NHS Trust, Wellcome Trust Centre for Human Genetics, Oxford, UK; 997 Queen Mary University of London, Barts Hospital and The London School of Medicine, London, UK; 998 Asan Medical Center, Department of Anesthesia, Seoul, Republic of Korea; 999 University Hospital of Besancon, Anesthesiology and Critical Care Medicine, Besançon, France; 1000 University of Franche-Comté, Besançon, France; 1001 University Hospital of Besancon, Thoracic and Cardiovascular Surgery, Besançon, France; 1002 University Hospital of Besancon, Clinical Methodology Center, Besançon, France; 1003 Instituto Nacional de Ciencias Médicas y Nutrición Salvador Zubirán, Critical Care, México, Mexico; 1004 Hospital Universitario de Bellvitge, Critical Care, L´Hospitalet, Spain; 1005 Instituto de Investigacion Biomédica de Bellvitge, L´Hospitalet, Spain; 1006 Hospital de la Santa Creu i Sant Pau, Laboratori de Recerca Cardiovascular, Barcelona, Spain; 1007 Hospital Universitari de Bellvitge, Anesthesiology, L´Hospitalet, Spain; 1008 Hospital Universitari de Bellvitge, Cardiac Surgery, L´Hospitalet, Spain; 1009 Hospital de la Santa Creu i Sant Pau, Institut de Recerca Cardiovascular, Barcelona, Spain; 1010 Hospital Universitari de Bellvitge, Critical Care, L´Hospitalet, Spain; 1011 Algarve Hospital Center, Faro Unit, Faro, Portugal; 1012 Hospital de Santa Cruz / CHLO, Lisbon, Portugal; 1013 Zurich University Hospital, Zurich, Switzerland; 1014 Georges Pompidou European Hospital, Paris, France; 1015 Hospital 12 de Octubre, Madrid, Spain; 1016 Complejo Universitario Canarias, Tenerife, Spain; 1017 Fundacion Jimenez Diaz, Madrid, Spain; 1018 Hospital 12 de la Princesa, Madrid, Spain; 1019 Hospital Virgen Macarena, Sevilla, Spain; 1020 Hospital Miguel Servet, Zaragoza, Spain; 1021 Hospital Carlos Haya, Malaga, Spain; 1022 Hospital Universitari de Bellvitge, Intensive Care, L’ Hospitalet de Llobregat, Spain; 1023 Hospital Universitari de Bellvitge, Laboratory, L’ Hospitalet de Llobregat, Spain; 1024 Jagiellonian University Medical College, IInd Department of Internal Medicine, Kraków, Poland; 1025 Jagiellonian University Medical College, Kraków, Poland; 1026 St. John Grande Hospital, Vascular Surgery Department, Kraków, Poland; 1027 HUCA, ICU, Oviedo, Spain; 1028 G. Papanikolaou Hospital, Thessaloniki, Greece; 1029 Academic Medical Center, University of Amsterdam, Transaltional Physiology, Amsterdam, Netherlands; 1030 Academic Medical Center, University of Amsterdam, Cardio-Thoracic Surgery, Amsterdam, Netherlands; 1031 Vilnius University, Vilnius, Lithuania; 1032 Jena University Hospital, Department for Anesthesiology and Intensive Care, Jena, Germany; 1033 Jena University Hospital, Center for Sepsis Control and Care, Jena, Germany; 1034 McMaster University, Department of Medicine (Division of Critical Care) & Department of Clinical Epidemiology & Biostatistics, Hamilton, Canada; 1035 Università degli Studi di Milano, Dipartimento di Fisiopatologica Medico-Chirurgica e dei Trapianti, Milan, Italy; 1036 Hospital Universitario Central de Asturias, Intensive Care Unit 1–2, Oviedo, Spain; 1037 Hospital Universitario Central de Asturias, Hematology Department, Oviedo, Spain; 1038 University of Oviedo, Oviedo, Spain; 1039 Hospital Universitario Central de Asturias, Intensive Care Unit 3, Oviedo, Spain; 1040 Hospital Universitario Central de Asturias, Cardiac Surgery, Oviedo, Spain; 1041 Kochi Medical School, Department of Anesthesiology and Intensive Care, Nankoku, Japan; 1042 G. Papanikolaou Hospital, Cardiac Surgery ICU, Thessaloniki, Greece; 1043 G. Papanikolaou Hospital, Cardiac Surgery, Thessaloniki, Greece; 1044 Brigham and Women’s Hospital, Department of Medicine, Boston, USA; 1045 University of Colorado, Division of Pulmonary Sciences and Critical Care Medicine, Boulder, USA; 1046 Massachusetts General Hospital, Pulmonary and Critical Care Medicine, Boston, USA; 1047 Brigham and Women’s Hospital, Renal Division, Boston, USA; 1048 Brigham and Women’s Hospital, Channing Division of Network Medicine, Boston, USA; 1049 Hospital de Clínicas de Porto Alegre, Intensive Care Unit, Porto Alegre, Brazil; 1050 Hospital Nossa Senhora Conceição, Intensive Care Unit, Porto Alegre, Brazil; 1051 Federal do Rio Grande do Sul, Faculdade de Medicina, Porto Alegre, Brazil; 1052 Universidade Federal do Rio Grande do Sul, Faculdade de Medicina - Department of Intensive Care, Porto Alegre, Brazil; 1053 Universidade Federal do Rio Grande do Sul, Faculdade de Medicina - Department of Medical Nutrition, Porto Alegre, Brazil; 1054 Universidade Federal do Rio Grande do Sul, Faculdade de Medicina - Department of Endocrinology, Porto Alegre, Brazil; 1055 Hospital de Base do Distrito Federal, Residência em Nutrição Clínica, Brasília, Brazil; 1056 Universidade de Brasília, Departamento de Nutrição, Brasília, Brazil; 1057 Universidade Estadual de Campinas, Universidade de Ciências Médicas, Campinas, Brazil; 1058 Imperial College Healthcare NHS Trust, Adult Critical Care, London, UK; 1059 Royal Brompton and Harefield NHS Foundation Trust, London, UK; 1060 Fuenlabrada University Hospital, Intensive Care Unit, Madrid, Spain; 1061 Fuenlabrada University Hospital, Pharmacology Department, Madrid, Spain; 1062 Fuenlabrada University Hospital, Madrid, Spain; 1063 Karolinska University Hospital / Karolinska Institutet, Department of Anesthesiology and Intensive Care, Huddinge, Sweden; 1064 School of Technology and Health, Royal Institute of Technology, Department of Environmental Physiology, Swedish Aerospace Physiology Center, Stockholm, Sweden; 1065 Charles Nicolles Hospital, Systemic Disease, Tunis, Tunisia; 1066 Morales Meseguer Hospital, Murcia, Spain; 1067 Jagiellonian University Medical College, Kraków, Poland; 1068 Jagiellonian University Medical College, IInd Department of Internal Medicine, Kraków, Poland; 1069 Charles Nicolles Hospital, Systemic Disease, Tunis, Tunisia; 1070 VU University Medical Center, Department of Intensive Care, Amsterdam, Netherlands; 1071 Centre Hospitalier Universitaire de Caen, Unité de Biostatistique et de Recherche Clinique, Caen, France; 1072 Pamela Youde Nethersole Eastern Hospital, Hong Kong, China; 1073 Tuen Mun Hospital, Hong Kong, Hong Kong, China; 1074 Dr Josep Trueta University Hospital, Intensive Care Unit, Girona, Spain; 1075 East Surrey Hospital, Intensive Care Unit, London, UK; 1076 National University Health System, Singapore, Singapore; 1077 Clinical Nutrition Research Centre,Singapore Institute for Clinical Sciences, Singapore, Singapore; 1078 Intermed Hospital, Intensive Care Department, Ulaanbaatar, Mongolia; 1079 Health Sciences University of Mongolia, Division of Emergency Medicine and Anesthesia, Ulaanbaatar, Mongolia; 1080 Salzburg General Hospital and Paracelsus Private, Salzburg, Austria; 1081 Sanatorio Otamendi y Miroli, Buenos Aires, Argentina; 1082 AMNCH, Critical Care, Dublin, Ireland; 1083 AMNCH, Dublin, Ireland; 1084 Hospital de Especialidades, Centro Médico La Raza, IMSS, Intensive Care Unit, Mexico, Mexico; 1085 Inonu University, Intensive Care, Malatya, Turkey; 1086 Changi General Hospital, Respiratory and Critical Care Medicine, Singapore, Singapore; 1087 Changi General Hospital, Singapore, Singapore; 1088 St Georges’s Foundation Trust, Neuro Intensive Care Unit, London, UK; 1089 Centro Hospitalar do Algarve, Serviço de Medicina Intensiva, Emergencia, Urgência e Cuidados Intensivos, Faro, Portugal; 1090 Universidade do Algarve, Ciências Biomédicas e Medicina, Faro, Portugal; 1091 Universidade do Porto, Faculdade de Medicina, CINTESIS, Porto, Portugal; 1092 Education and Research Institute, Hospital Sírio-Libanês, Intensive Care Unit, Sao Paulo, Brazil; 1093 Hospital das Clínicas, Universidade de São Paulo, Emergency Medicine Discipline, Sao Paulo, Brazil

## NEUROMONITORING

### A391 Physiologic monitoring for neurocritically-ill patients: an international survey of intensivists

#### S. Sivakumar^1^, F.S. Taccone^2^, K.A. Desai^1^, C. Lazaridis^1^

##### ^1^Baylor College of Medicine, Neurology/Neurocritical Care, Houston, TX, USA; ^2^Erasmus Hospital, Intensive Care Medicine, Brussels, Belgium

###### **Correspondence:** C. Lazaridis - Baylor College of Medicine, Neurology/Neurocritical Care, Houston, TX, USA

**Introduction** In addition to systemic hemodynamics, the management of neurocritically ill patients is often informed by neuromonitoring. In the absence of high-level evidence clinicians are often guided by personal and local expertise. Little is known about practices as they pertain to the use of such monitoring in patients with acute brain injury (ABI).

**Objectives** To investigate practices in bedside monitoring for ABI patients. Particularly interested in differences among “neurointensivists” (NIs; defined here as intensivists whose clinical practice is comprised > 1/3 by neurocritical care) and other intensivists (OIs). Also, to explore patterns specific to traumatic brain injury (TBI) and subarachnoid hemorrhage (SAH), as well as preferences and availability of particular technologies/devices.

**Methods** Electronic survey of 22 items including two case-based scenarios; endorsed by SCCM (9,000 recipients) and ESICM (on-line newsletter) in 2013. A sample size of 370 was calculated based on a population of 10,000 physician members, a 5 % margin error, and 95 % confidence interval. We summarized results using descriptive statistics (proportions with 95 % confidence intervals). A chi-square test was used to compare proportions of responses between NIs and OIs with a significance p < 0.05.

**Results** There were 655 responders (66 % completion rate); 422(65 %) were classified as OIs and 226(35 %) as NIs. More NIs follow hemodynamic protocols for neurocritically-ill patients (56 % vs. 43 %, p 0.001), in TBI (44.5 % vs. 33.3 %, p 0.007), and in SAH (38.1 % vs. 21.3 %, p < 000.1). For delayed cerebral ischemia (DCI), more NIs target cardiac index (CI) (35 % vs. 21 %, p 0.0001), and fluid responsiveness (62 % vs. 53 %, p 0.03), use more bedside ultrasound (BUS) (42 % vs. 29 %, p 0.005) and arterial waveform analysis (40 % vs. 29 %, p 0.02). For DCI neuromonitoring, NIs use more angiography (57 % vs. 43 %, p 0.004), TCD (46 % vs. 38 %, p 0.0001), and CTP (32 % vs.16 %, p 0.0001). For CPP optimization in TBI, NIs use more arterial waveform analysis (45 % vs. 35 %, p 0.019), and BUS (37 % vs. 27.7 %, p 0.023), while more OIs monitor mixed venous oxygen saturation (54.1 % vs. 45 %, p 0.045). For TBI neuromonitoring, NIs use more PbtO_2_ (28 % vs. 10 %, p 0.0001). In the case scenario of raised ICP/low PbtO_2_, most employ analgosedation (47 %) and osmotherapy (38 %). Fewer make use of preserved pressure reactivity, particularly OIs (vasopressor use 23 % vs. 34 %, p 0.014).

**Conclusions** There is large heterogeneity in the use of monitoring protocols, variables, and technologies/devices. “Neurointensivists” not only employ more neuromonitoring but also more hemodynamic monitoring in patients with acute brain injury. ICP/CPP remain the most commonly followed neuro-variables in TBI patients, with low use of other brain-physiology parameters, suggesting that clinicians make limited efforts to individualize these goals.

### A392 A prospective observational pilot study of cerebral autoregulation measured by near infrared spectroscopy (NIRS) in patients with septic shock

#### M. Skarzynski^1^, M. Sekhon^2^, W. Henderson^2^, D. Griesdale^2^

##### ^1^Centre Hospitalier Régional Orléans, Réaimation Médicale, Orléans, France; ^2^University of British Columbia, Vancouver, Canada

###### **Correspondence:** M. Skarzynski - Centre Hospitalier Régional Orléans, Réaimation Médicale, Orléans, France

**Introduction** Impairment of cerebral autoregulation has been proposed as a possible explanation of cognitive dysfunction in patients with septic shock. Although transcranial Doppler has previously been used to assess cerebral autoregulation, this technology can only evaluate at single points in time. In contrast, near-infrared spectroscopy offers continuous assessment of cerebral autoregulation.

**Objectives** Assess cerebral autoregulation using NIRS in patients admitted to the intensive care unit with septic shock.

**Methods** We included 20 patients admitted with septic shock admitted to the intensive care unit (ICU) at Vancouver General Hospital (VGH). The ICU is a 31-bed mixed medical-surgical unit affiliated with the University of British Columbia. We excluded patients with acute or chronic neurological disorders, end stage liver disease, long-term dialysis, and those admitted following a cardiac arrest. We measured regional cerebral oximetry (rSO2) by NIRS (INVOS®, Covidien, Ireland) for 24 hours. NIRS and mean arterial pressure (MAP) data were collected in real time using ICM + ® brain monitoring software (Cambridge University, UK). ICM+ calculates a moving Pearson correlation coefficient (COx) between 30 consecutive, 10 second average MAP and rSO2 values. Impaired cerebral autoregulation was defined as a COx greater than 0.3. We also defined the impaired autoregulation index (IARindex) as the percentage of monitoring time spent with an impaired autoregulation. The IARindex was calculated for each 6 hours period (H_0_H_6_; H_6_H_12_;H_12_H_18_, H_18_H_24_), and for 24 hours.

**Results** We analyzed 19 patients, one patient being excluded from analysis due to removal for arterial line [mean (Standard deviation); median (interquartile)] age 67(12), APACHE II score 21(6) median MAP 72 [67–75] mmHg, median rSO2 64 [57–70] %, median end tidal carbon dioxide 30 [27–35] mmHg and median temperature 37.1 [36.8-37.3] °C. After removal of artefacts, the mean monitoring time was 22 h08 (8 h54). All patients had impaired cerebral autoregulation during their monitoring time. The mean IAR index was 17 (9.5) %. During H_0_H_6_ and H_18_H_24_, the majority of our patients; respectively 53 and 71 % had an IAR index > 10 %.

**Conclusion** According to our data, patients with septic shock had impaired cerebral autoregulation within the first 24 hours of their admission in the ICU. In our patients, we described a variability of distribution of impaired autoregulation according to time.

**References**

Schramm P, Klein KU, Falkenberg L, et al. Impaired cerebrovascular autoregulation in patients with severe sepsis and sepsis-associated delirium. Crit Care 2012; **16**: R181.

Aries MJH, Czosnyka M, Budohoski KP, et al. Continuous determination of optimal cerebral perfusion pressure in traumatic brain injury. Crit. Care Med. 2012.

**Fig. 1 (abstract 392).**
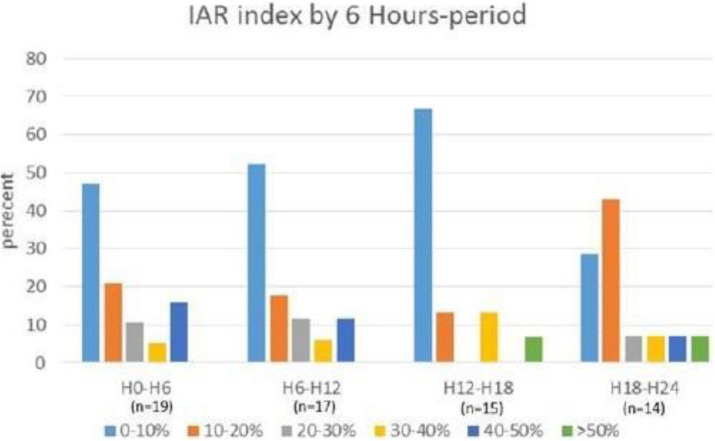
IAR index by 6 hours period

### A393 Changes in muscle thickness throughout hospitalisation after traumatic brain injury

#### L. Chapple^1^, A. Deane^1,2^, L. Williams^3^, R. Strickland^2^, K. Lange^4^, D. Heyland^5^, M. Chapman^1,2^

##### ^1^University of Adelaide, Discipline of Acute Care Medicine, Adelaide, Australia; ^2^Royal Adelaide Hospital, Intensive Care, Adelaide, Australia; ^3^Griffith University, Menzies Health Institute of Queensland, Gold Coast, Australia; ^4^University of Adelaide, Discipline of Medicine, Adelaide, Australia; ^5^Kingston General Hospital, Clinical Evaluation Research Unit, Kingston, Canada

###### **Correspondence:** L. Chapple - University of Adelaide, Discipline of Acute Care Medicine, Adelaide, Australia

**Introduction** Patients with a traumatic brain injury (TBI) remain in hospital for extended periods. It is likely that muscle mass in these patients diminishes over the hospital admission, yet this has not previously been quantified. Ultrasonography may provide a useful measure of changes in muscle size over the hospital stay.

**Objectives** To quantify changes in ultrasound-derived quadriceps muscle layer thickness (QMLT) and establish the feasibility of repeated ultrasound examinations throughout the entire hospitalisation of patients with a TBI.

**Methods** Adult patients with a moderate-severe TBI (Glasgow Coma Scale 3–12) consecutively admitted to the intensive care unit (ICU) at a single trauma referral centre over 12 months were eligible following informed consent. Ultrasounds of QMLT at the midpoint and two-thirds between the anterior superior iliac spine and top of the patella were conducted weekly during admission. Data were censored at 3-months and are mean (SD) unless otherwise stated.

**Results** Thirty-three patients [45.4 (16.6) years; 88 % male; 55 % severe TBI; Trauma Injury Severity Score 0.5 (0.3); median APACHE II 18 (IQR: 14–22); admission body mass index 27.2 (6.5) kg/m^2^] were studied. Primary injury cause was vehicular (58 %) and 67 % of TBIs were multi-trauma. Median length of ICU and ward-based stay was 13.4 [IQR: 6.5-17.9] and 31.8 [9.4-52.4] days, respectively. At 3-months all patients were alive and four remained in hospital. A total of 123 ultrasounds were taken; 30 (24 %) in ICU and 93 (76 %) on the acute ward, equal to 3.7 ultrasounds per patient. Ultrasounds were not possible at 21 % of time-points in hospital due to patient agitation and 61 % at 3-month follow-up due to inability to attend appointment in person. Twenty-eight (85 %) patients had >1 ultrasound taken during their admission, for which the mean baseline measure was 1.78 (0.72) cm and within patient standard deviation 0.3 cm. Nineteen patients (58 %) had >2 ultrasounds during the hospital admission with a baseline measure of 1.78 (0.68) and proximate measure to discharge of 1.53 (0.52) cm. There was a 6.2 (35.8) % decrease between the baseline and proximate discharge measures. QMLT decreased over the first four weeks of hospital admission [week 1 (n = 22): 2.01 (0.84), week 2 (n = 24): 1.66 (0.59), week 3 (n = 19): 1.50 (0.57), week 4 (n = 13): 1.40 (0.38)]. At 3-months the QMLT was 2.00 (0.7) cm (n = 22), with a decrease from baseline of 3.2 (26.0) %.

**Conclusions** This is the first study to describe longitudinal change in muscle size over the hospital admission in patients with a moderate-severe TBI. Whilst technically feasible, issues with compliance and missing data and intra-individual variation between measurements are challenges when conducting regular ultrasound measures in this population. Muscle thickness reduced throughout hospitalisation, but improved towards baseline by 3-months.

### A394 Acute impairment of saccadic eye movement is associated with cerebral infarction after aneurysmal subarachnoid haemorrhage

#### M.J. Rowland^1,2^, P. Garry^1,2^, J. Westbrook^1,2^, R. Corkill^2^, C.A. Antoniades^1,2^, K.T. Pattinson^1,2^

##### ^1^University of Oxford, Nuffield Department of Clinical Neurosciences, Oxford, UK; ^2^Oxford University Hospitals NHS Foundation Trust, Neurosciences Intensive Care Unit, Oxford, UK

###### **Correspondence:** M.J. Rowland - Oxford University Hospitals NHS Foundation Trust, Neurosciences Intensive Care Unit, Oxford, UK

**Introduction** Cerebral infarction due to delayed cerebral ischaemia (DCI) remains a significant cause of morbidity and mortality following aneurysmal subarachnoid haemorrhage (SAH). Damage to the brain in the first 72 hours (“early brain injury”) is likely to play a key pathophysiological role but remains difficult to quantify objectively and non-invasively at the bedside- especially in those patients who do not require sedation or ventilation. Current diagnostic modalities used in routine clinical practice are either invasive, require ionising radiation or contrast or have a high learning curve and user variability.

**Objectives** We sought to determine whether saccadic eye movements are impaired following SAH and whether measurement of saccadic latency (SL) in the acute period post-aneurysm rupture is associated with the likelihood of developing DCI in patients after SAH.

**Methods** 24 male/female patients (mean age 53.32, range 31–70) with World Federation of Neurosurgeons (WFNS) grade I and II aneurysmal SAH, treated endovascularly within 72 hours of rupture were recruited. DCI and DCI-related cerebral infarction were defined as per consensus guidelines.^1^ Saccadometry data was collected at three time points: in the first 72 hours, between days 5 and 10 and at three months post-SAH. Data from 10 healthy age/gender matched controls was collected on one occasion for comparison.

**Results** Age-adjusted median SL in patients was significantly prolonged in the first 72 hours post-SAH when compared to controls (188.7, 95 % CI = (176.9, 202.2)ms v 160.7, 95 % CI = (145.6, 179.4)ms, p = 0.0054 *t*-test). By 3 months post-SAH, there was no significant difference in median SL compared to controls (188.7, 95 % CI = (176.9, 202.2)ms v 180.0, 95 % CI = (165.1, 197.8)ms, p = 0.4175 *t*-test). Patients diagnosed with cerebral infarction due to DCI had a significantly higher age-adjusted median SL in the first 72 hours than those without infarction (240.6, 95 % CI = (216.7, 270.3)ms v 204.1, 95 % CI = (190.7, 219.5)ms, p = 0.0157 *t*-test). This difference was more pronounced during days 5–10 post-SAH - the peak incidence for DCI (303.7, 95 % CI = (266.7 352.7)ms v 207.6, 95 % CI = (193.7, 223.6)ms, p < 0.0001 *t*-test). A binary generalised linear model showed that median SL in the first 72 hours was the only significant predictor of cerebral infarction after SAH.

**Conclusions** This is the first study to use saccadometry to measure eye movements in patients with SAH during the acute period post-rupture. Results show that median saccadic latency in the first 72 hours following SAH is an independent predictor of the risk of developing cerebral infarction due to DCI. Saccadometry may act as a potential objective biomarker of early brain injury to guide the need for intensive care admission and treatment.

**References**

1. Vergouwen MDI et al. 2010, Stroke;(41):2391–2395

**Grant acknowledgment**

MRC (UK).

### A395 The incidence of spreading depolarisations in ischaemic brain injury using non-invasive near-infrared spectroscopy

#### G. Fatania^1^, A.J. Strong^2^

##### ^1^King's College Hospital, Department of Neurosurgery, London, UK; ^2^King's College London, Institute of Psychiatry, Psychology and Neuroscience, Department of Clinical Neuroscience, London, UK

###### **Correspondence:** G. Fatania - King's College Hospital, Department of Neurosurgery, London, UK

**Introduction** Spreading depolarisations (SD) occur spontaneously in ischaemic cortex and are implicated in the evolution of the ischaemic penumbra. Subsequent enlargement of the ischaemic lesion causes worse neurological outcomes. Monitoring for SDs is solely performed through invasive electrocorticography (ECoG) which is located when a patient requires emergency surgery. SDs can cause significant haemodynamic changes which may be amenable to non-invasive monitoring.

**Objectives** This study seeks to develop such a technique using near-infrared spectroscopy (NIRS). NIRS allows surrogate measure of cerebral blood flow by inferring changes in the concentration of oxyhaemoglobin and deoxyhaemoglobin.

**Methods** 5 ischaemic brain injury patients requiring emergency neurosurgery (4 retrospective, 1 prospective) recruited to the COSBID study at King's College Hospital (and monitored with ECoG) underwent concomitant NIRS monitoring. The NIRS data was analysed for significant haemodynamic response to SDs.

**Results** In total, three ECoG-confirmed SDs occurred during NIRS monitoring, each in a different patient. Two acute (one minute) hypoperfusion responses were seen after an SD in one patient. One acute (one minute) hyperperfusion response was seen after an SD in one patient. Three subacute (20 minute) hypoperfusion responses were seen across two patients.

**Conclusion** This preliminary data is promising that NIRS can be used to assess the haemodynamic responses secondary to SDs. More data is required so that the responses can be characterised adequately which could lead to a novel non-invasive monitoring technique.

### A396 Predicting intracranial pressure and brain tissue oxygen crises in patients with severe traumatic brain injury

#### R.B. Myers^1^, C. Lazaridis^2^, C.M. Jermaine^1^, C.S. Robertson^3^, C.G. Rusin^4^

##### ^1^Rice University, Computer Science, Houston, TX, USA; ^2^Baylor College of Medicine, Neurology, Neurocritical Care, Houston, TX, USA; ^3^Baylor College of Medicine, Neurosurgery, Houston, TX, USA; ^4^Baylor College of Medicine, Pediatric Cardiology, Houston, TX, USA

###### **Correspondence:** C. Lazaridis - Baylor College of Medicine, Neurology, Neurocritical Care, Houston, TX, USA

**Introduction** Two central physiologic targets in the management of severe traumatic brain injury (TBI) are the intracranial pressure (ICP) and the partial brain tissue oxygen tension (PbtO_2_). Intracranial pressure and PbtO_2_ thresholds are incorporated into step-tiered clinical protocols and guidelines however by the time treatment is provided, it may be too late. The ability to predict the onset of these “crisis” events would potentially provide clinicians with valuable time to attempt aborting the episode and/or to appropriately manage it. Combining statistical machine learning with physiologic and clinical insight allows the construction of robust quantitative models to predict future events such as elevated ICP or low PbtO_2._ Such models provide targets for evidence-based individualized treatment in real time.

**Objectives** Develop computer algorithms that can recognize physiologic patterns in TBI patients that occur in advance of intracranial pressure ICP and PbtO_2_ crises. The automated early detection of crisis precursors can provide clinicians with time to intervene in order to prevent or mitigate secondary brain injury.

**Methods** A retrospective study was conducted from prospectively collected physiologic data. Intracranial pressure and PbtO_2_ crisis events were defined as ICP ≥ 20 mmHg lasting at least 15 minutes and PbtO_2_ values < 10 mmHg for at least 10 minutes, respectively. The physiologic data preceding each crisis event were used to identify precursors associated with crisis onset. Multivariate classification models were applied to recorded data in 30-minute epochs of time to predict crises between 15 and 360 minutes in the future. Our cohort consisted of 817 severe TBI subjects admitted to the neurosurgical intensive care unit of Ben Taub General Hospital in Houston, Texas.

**Results** Our algorithm can predict the onset of an ICP crisis with 30 minutes advance warning and an AUC of 0.86 using only ICP measurements and time since last crisis. An analogous algorithm can predict the start of PbtO_2_ crises with 30 minutes advanced warning and an AUC of 0.91.

**Conclusions** We report here novel algorithms that provide accurate and timely predictions of intracranial hypertension and tissue hypoxia crises in patients with severe TBI. Almost all of the information needed to predict the onset of these events is contained within the signal of interest and the time since last crisis. These predictive algorithms offer clinicians the opportunity to prepare and potentially prevent secondary brain injury insults.

**Grant acknowledgment**

Supported by a training fellowship from the Keck Center of the Gulf Coast Consortia, on Rice University´s NLM Training Program in Biomedical Informatics (grant number T15LM007093) and by the NSF under grant number 0964526.

### A397 Early EEG for outcome prediction of postanoxic coma: validation of undisputable predictive value and cost-effectiveness analysis

#### J. Hofmeijer^1,2^, L. Sondag^2^, M.C. Tjepkema-Cloostermans^3^, A. Beishuizen^4^, F.H. Bosch^5^, M.J.A.M. van Putten^1,3^

##### ^1^University of Twente, Clinical Neurophysiology, Enschede, Netherlands; ^2^Rijnstate Hospital, Neurology, Arnhem, Netherlands; ^3^Medical Spectrum Twente, Clinical Neurophysiology, Enschede, Netherlands; ^4^Medical Spectrum Twente, Intensive Care, Enschede, Netherlands; ^5^Rijnstate Hospital, Intensive Care, Arnhem, Netherlands

###### **Correspondence:** J. Hofmeijer - Rijnstate Hospital, Neurology, Arnhem, Netherlands

**Introduction** Early identification of patients without potential for recovery of brain function may prevent inappropriate treatment of comatose patients after cardiac arrest. We recently showed that evolution of the EEG pattern within the first 24 hours after cardiac arrest robustly contributes to multimodal prediction of either poor or good outcome [1].

**Objectives** We aim to confirm our results and present a cost-effectiveness analysis.

**Methods** 432 consecutive comatose patients after cardiac arrest were included in a prospective cohort study on two intensive care units. Continuous EEG was measured during the first three days. EEGs were visually classified as unfavorable (isoelectric, low-voltage, burst-suppression-with-identical-bursts), intermediate, or favorable (continuous patterns), at 12, 24, 48, and 72 hours by two reviewers, independently. Outcome was dichotomized as good (CPC score 1 or 2) or poor (CPC score 3, 4, or 5) at six months. EEG parameters were related to outcome using logistic regression analysis. Cost-effectiveness in the hospital is currently estimated by decision tree analysis.

**Results** Poor outcome occurred 54 % of included patients. Single parameters unequivocally predicting poor outcome in the first 277 patients were an unfavorable EEG pattern at 24 hours, absent pupillary light responses at 48 hours, and absent SSEPs at 72 hours. Together, these had a specificity of 100 % and a sensitivity of 50 %. Favorable EEG patterns at 12 hours were strongly associated with good outcome. EEG beyond 24 hours had no additional predictive value. For the remaining 155 patients, EEG analyses are ongoing. Data on cost-effectiveness, based on the assumption of EEG based treatment discontinuation, will be presented.

**Conclusions** EEG within 24 hours is a robust contributor to prediction of poor or good outcome and should be included in guidelines for treatment of comatose patients after cardiac arrest.

**Reference**

1. Hofmeijer et al. Neurology 2015;85:137–43.

### A398 Cerebral energy dysfunction and hyperemia during the early brain injury phase following aneurysmal subarachnoid hemorrhage

#### L. Carteron, C. Patet, D. Solari, M. Oddo

##### CHUV, Department of Intensive Care Medicine, Neuroscience Critical Care Research Group, Lausanne, Switzerland

###### **Correspondence:** L. Carteron - CHUV, Department of Intensive Care Medicine, Neuroscience Critical Care Research Group, Lausanne, Switzerland

**Introduction** Mechanisms of early brain injury (EBI) following aneurysmal subarachnoid hemorrhage (SAH) are poorly understood. Using brain perfusion CT (pCT) and cerebral microdialysis (CMD), we examined the relationship of cerebral energy metabolism with brain perfusion during the EBI phase after SAH in humans.

**Methods** Prospective observational cohort of poor-grade SAH patients monitored with cerebral microdialysis (CMD) who were resuscitated according to current guidelines. Data from brain pCT (performed 44 ± 25 hrs from ictus) were matched to CMD epochs displaying cerebral energy dysfunction, defined by a CMD lactate/pyruvate ratio > 40 and/or lactate > 4 mmol/L.

**Results** A total of 19 pCT and 127 hours of CMD samples (15 patients) were analyzed. The majority (14/19) of pCT were associated with cerebral energy dysfunction, despite main cerebral physiologic variables were within normal range (intracranial pressure 14.2 ± 7.2 mmHg, PbtO_2_ 25 ± 10 mmHg). Energy dysfunction was associated with simultaneous normal (n = 9; 28–65 mL/100 g/min) or hyperemic cerebral blood flow (n = 5; 69–85 mL/100 g/min). Energy dysfunction also correlated with concomitant pathological elevations of glutamate and glycerol, that were more pronounced in hyperemic than normal pCT (LPR 54 ± 12 vs. 42 ± 7 and glycerol 157 ± 76 vs. 95 ± 41 μmol/L, both *p* < 0.01; glutamate 38 ± 52 vs. 26 ± 24 μmol/L, *p* = 0.18, *t*-test for comparisons between groups).

**Conclusions** EBI after SAH is associated with profound cerebral metabolic and cellular distress, despite normal-hyperemic brain perfusion. Our findings reveal alternative pathophysiological mechanisms to ischemia in the EBI phase of SAH in humans and support therapeutic strategies targeted to energy dysfunction in this setting.

GRANTS

Supported by research grants from the Swiss National Science Foundation (SNSF), the Novartis Foundation for Biomedical Research, the Société Française d'Anesthésie et de Réanimation (SFAR) and the “Fondation des Gueules Cassées”.

### A399 Optic nerve sheath diameter evaluated by transorbital sonography in healthy volunteers from Pakistan

#### M.A. Ali

##### Aga Khan University Hospital, Anaesthesiology, Karachi, Pakistan

**Introduction** Raised intracranial pressure is a common manifestation of severe brain injury. Rapid diagnosis and timely intervention is required to prevent secondary brain damage and death.An accurate, reliable, noninvasive, point-of-care monitoring device to identify presence of intracranial hypertension would be helpful in situations where there is clinical suspicion for intracranial hypertension. Bedside ocular ultrasound is an emerging noninvasive technique to measure optic nerve sheath diameter. Knowledge of the normal range of optic nerve sheath diameter in a healthy population is essential to interpret this measurement as a marker of raised intracranial pressure in clinical practice.

**Objectives** To evaluate normal optic nerve sheath diameter in healthy volunteers in Pakistan.

**Methods** Hundred healthy volunteers of Pakistani origin, aged more than 18 years were recruited in the study. The ultrasound probe was placed on the superior and lateral aspect of the orbit against the upper eyelid with the eye closed. For each subject, the primary investigator performed three measurements on each eye. The measurements of each eye were then averaged to yield a mean optic nerve sheath diameter (ONSD). Results are presented as mean ± standard deviation (SD). Statistical analysis was performed with SPSS software version 19. Mann Whitney *U* test was used to compare unpaired variables between genders and Wilcoxon matched pairs signed rank test to compare left and right eyes.

**Results** The median ONSD of right eye was 4.84 mm and 95 % of individuals had mean ONSD in the range 4.84-4.97 mm while the median ONSD of left eye was 4.86 mm and 95 % of individuals had mean ONSD in the range 4.85-4.96 mm. There was no difference among the 3 repeated measures of ONSD in each eye. There was no relationship between ONSD with age, gender and measurement taken between left and right eyes.

**Conclusions** 95 % of healthy Pakistani adults have an ONSD less than 4.82 mm. ONSD more than 4.82 mm in this population should be considered abnormal and may reflect raised intracranial pressure.

**Note:** This abstract has been previously published and is available at [3]. It is included here as a complete record of the abstracts from the conference.

**References**

1. Brain Trauma Foundation, American Association of Neurological Surgeons, Congress of Neurological Surgeons, Joint Section on Neurotrauma, Critical Care, AANS/CNS. Guidelines for the management of severe traumatic brain injury. VI. Indications for intracranial pressure monitoring. J Neurotrauma. 2007; 24(Suppl 1):S37-44.

2. Morgenstern LB, Hemphill JC III, Anderson C, Becker K, Broderick JP, Connolly ES Jr, Greenberg SM, Huang JN, Mac-Donald RL, Messe´ SR, Mitchell PH, Selim M, Tamargo RJ, American Heart Association Stroke Council and Council on Cardiovascular Nursing. Guidelines for the management of spontaneous intracerebral hemorrhage: a guideline for healthcare professionals from the American Heart Association/American Stroke Association. Stroke. 2010;41:2108–29.

3. Ashgar A, Hasmi M, Hussain A (2015) Optic nerve sheath diameter evaluated by transorbital sonography in health volunteers from Pakistan. Anaesthesia, Pain & Intensive Care 19(3):p282.

### A400 Heart rate variability and multimodal brain monitoring before and after decompressive craniectomy in traumatic brain injury

#### C. Dias^1,2^, R. Almeida^3,4,5^, A. Vaz-Ferreira^6^, J. Silva^6^, E. Monteiro^1,2^, A. Cerejo^7,8^, A.P. Rocha^4,9^

##### ^1^Centro Hospitalar São João, Intensive Care, Porto, Portugal; ^2^Faculty of Medicine, University of Porto, Medicine, Porto, Portugal; ^3^Faculdade de Ciências, Universidade do Porto, Departamento de Matemática, Porto, Portugal; ^4^Centro de Matemática, Universidade do Porto, Porto, Portugal; ^5^The Biomedical Research Networking Center in Bioengineering, Biomaterials and Nanomedicine, Zaragoza, Spain; ^6^Centro Hospitalar São João, Porto, Portugal; ^7^Centro Hospitalar São João, Neurosurgery, Porto, Portugal; ^8^Faculty of Medicine, University of Porto, Clinical Neurosciences, Porto, Portugal; ^9^Faculdade de Ciências, Universidade do Porto, Porto, Portugal

###### **Correspondence:** C. Dias - Faculty of Medicine, University of Porto, Medicine, Porto, Portugal

**Introduction** Autonomic control and cerebral autoregulation (CAR) may be disturbed after traumatic brain injury (TBI) and become severely impaired due to intracranial hypertension (IH). Decompressive craniectomy (DC) after TBI is a tiered therapy for patients with IH, but the links between autonomic derangement, intracranial pressure (ICP), impaired cerebral autoregulation and outcome remain poorly explored.

**Objectives** We aimed to evaluate the relationship between heart rate variability (HRV), as a surrogate of autonomic control, ICP and CAR before and after DC.

**Methods** We retrospectively studied 9 adult TBI patients, admitted to the Neurocritical Care Unit at Hospital São João, Porto that were submitted to primary or secondary DC and had completed monitoring records 12 h before and 24 h after surgery. Patients were monitored with continuous ECG, ICP, cerebral perfusion pressure, cerebral autoregulation with pressure reactivity and pressure-volume compensatory reserve index (RAP). Automatic delineation of ECG signal was performed using a wavelet-based approach and HRV analysis in time and frequency domain was done according to the guidelines for both periods selected.

**Fig. 2 (abstract A400).**
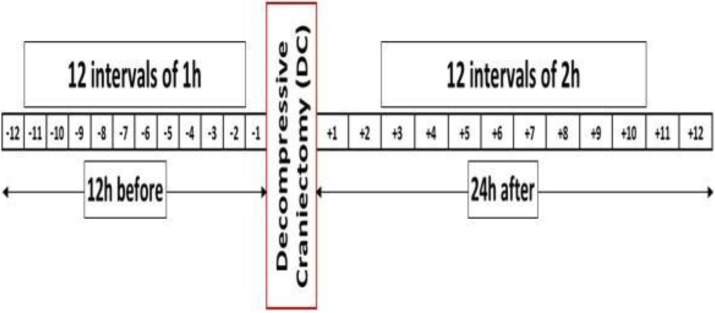
Monitoring time frame analysis before and after DC

**Results** A total of 48 consecutive TBI patients submitted to DC were screened, but we had to exclude 39 patients because of incomplete monitoring data. The median age was 27 (IQR13), 7 were men, median GCS was 6 (IQR3), median GOS was 4 (IQR2) and hospital mortality was 22 %. DC led to a significantly decrease in ICP maximum value (p = 0.011), CPP (p = 0.038) and RAP (p = 0.008) after surgery. There were no significant differences between values of PRx and HRV variables both in time and frequency domain. However, we found statistically significant correlations between HRV nonparametric variables and multimodal brain monitoring variables (Fig. 2). Before DC normalized low frequency (LFn), high frequency (HFn) and LF/HF ratio are positively correlated with ICP maximum (ICPmax) value, (respectively Rs = 0.5,p = 0.000; Rs = 0.3,p = 0.005;Rs = 0.4,p = 0.0001). After DC the correlation becomes negative but non-significant. Total power (Tp) has a positive and significant correlation with CPP before DC (Rs = 0.5,p = 0.000). RAP is negatively correlated with Tp and HF after DC (Rs = −0.4,p = 0.000;Rs = −0.5,p = 0.000). PRx increases after DC and we could not find any important correlation with HRV.

**Conclusions** Further studies are warranted to better understand the brain changes and autonomic distress associated with high intracranial pressure and decompressive craniectomy.

**References**

1. HRV: Standards of measurement, physiological interpretation, and clinical use. Task Force of the European Soc. of Cardiology and the North American Soc. of Pacing and Electrophysiology. Eur Heart J 1996;17(3):354–81.

2. Sykora M, et al. Autonomic Impairment in Severe TBI: A Multimodal Neuromonitoring Study. Crit Care Med 2016.

3. Kolias AG, et al. Decompressive craniectomy following TBI: developing the evidence base. Br J Neurosurg 2016;30(2):246–50.

**Fig. 3 (abstract A400).**
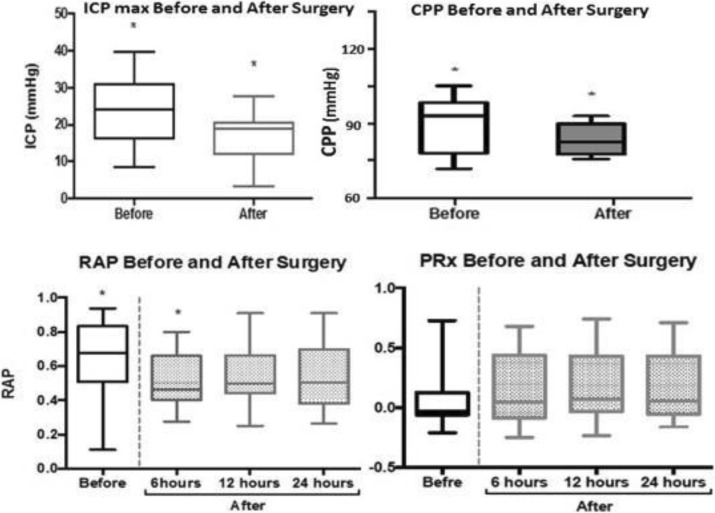
Multimodal Brain Monitoring before and afterDC

### A401 Value of noninvasive ultrasonographic techniques in assessing increased intracranial pressure in patients with moderate to severe traumatic brain injury

#### A.A. Elsayed^1^, A.M. Abougabal^2^, B.N. Beshey^1^, K.M. Alzahaby^1^

##### ^1^Faculty of Medicine, Alexandria University, Critical Care Medicine Department, Alexandria, Egypt; ^2^Faculty of Medicine, Alexandria University, Radiology Department, Alexandria, Egypt

###### **Correspondence**: K.M. Alzahaby - Faculty of Medicine, Alexandria University, Critical Care Medicine Department, Alexandria, Egypt

**Introduction:** The idea of a non-invasive method of measuring intracranial pressure (ICP) is captivating, as disadvantages seen in relation to the invasive methods of ICP measuring, that is, hemorrhage, infection, and costly expenses are avoidable.

**Objectives:** Determine the value of ultrasonographic transcranial Doppler (TCD) and optic nerve sheath diameter(ONSD)in assessing increased ICP and outcome of moderate to severe traumatic brain injury patients.

**Methods:** 40 patients with moderate to severe traumatic brain injury(GCS ≤ 13) admitted to a university teaching hospital were enrolled. ONSD and TCD measurements were performed daily for 7 days. TCD was performed on both middle cerebral arteries(MCA), recording peak systolic (sFV), end diastolic(dFV)and mean time-averaged(mFV)blood flow velocities in cm/s. The Gosling and King's pulsatility index(PI) was calculated:PI = sFV-dFV/mFV. Marshall and Rotterdam head CT neuroimaging scales were recorded on admission, 48 hours and 5 to 7 days later. Glasgow Outcome Scale(GOS)was assessed six months after discharge for survivors.

**Results:** The PI significantly increased in days 2 and 3 then it showed a non-significant decrease in day 4 and 5, all compared to baseline value. This was followed by a significant decrease in days 6 and 7. Comparing the PI's daily reading with the previous day was significant, increasing from day 1 to day 2, and from day 2 to day 3 then decreasing from day 3 till day 7(p < 0.001). The PI showed a significant direct correlation the Marshall and Rotterdam Scales on days 3 and 7, and Rotterdam Scale on day 7. A significant direct correlation was demonstrated between the ICU LOS and the PI determined on days 4 to7. No significant correlation was found between the mean ONSD and the PI measured on days 1 to 5, but a significant direct correlation was demonstrated between the two parameters on day 6 and day 7. The sFV, dFV and mFV were significantly higher on days 2 to 4 in survivors than in non-survivors. While the PI on day 2 and 3 was significantly lower in survivors than in non-survivors. On day 2, a mFV of ≤27.31 cm/sec was 94.44 % accurate in detecting non-survivors. On day 3, a mFV of ≤6.62 cm/sec was 100 % accurate in detecting non-survivors. On day 2, a PI of >1.26 was 89.47 % accurate in detecting non-survivors. On day 3, a PI of >1.87 was 100 % accurate in detecting non-survivors.

**Conclusions:** There was a significant day-to-day changes in the ONSD and PI in TBI patients making serial estimation of clinically/radiologically diagnosed raised ICP possible in the absence of invasive ICP measurement.The correlation between PI and CT imaging of the head together with its cost-effectiveness, safety to the patient and permanent availability makes the sonographic approach highly beneficial. TCD derived parameters predicted survival in TBI patients.TCD derived PI correlated directly with ICU length of stay.

**Fig. 4 (abstract A401).**
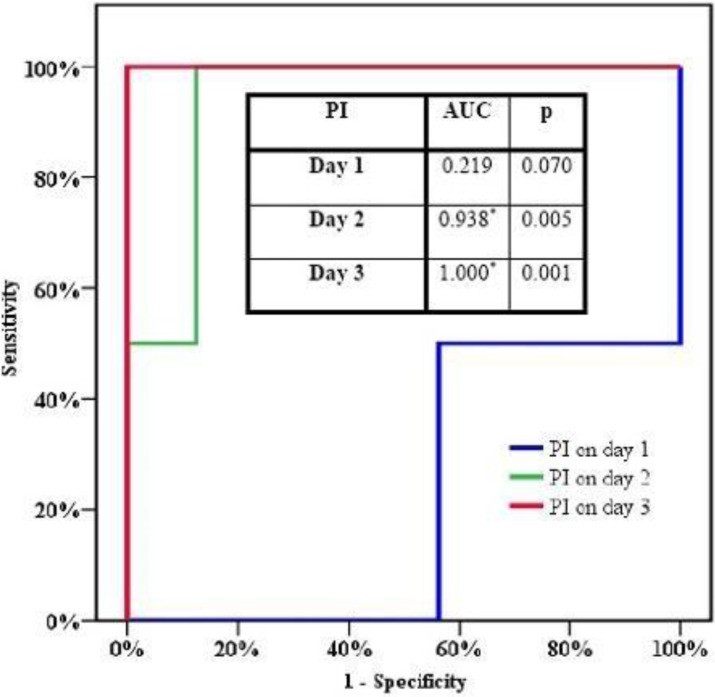
ROC curve for PI to determine its ability to predi

**Fig. 5 (abstract A401).**
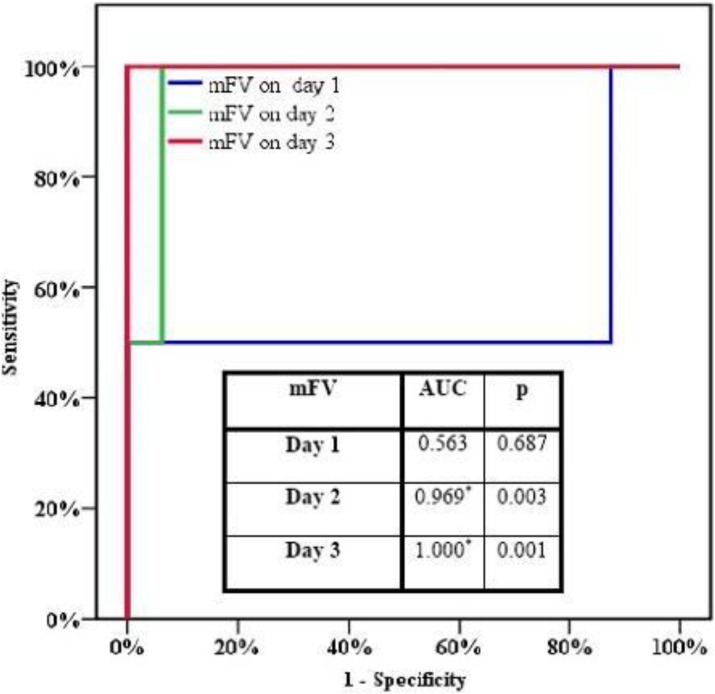
ROC curve for mFV to determine its ability to pred

**Fig. 6 (abstract A401).**
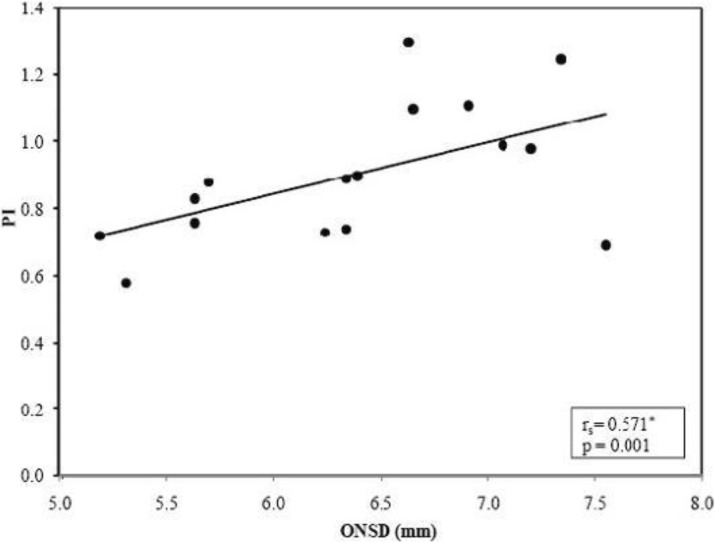
Correlation between ONSD and PI on day 7

### A402 Cerebral near-infrared spectroscopy in adults on veno-arterial extracorporeal membrane oxygenation

#### S. Pozzebon, A. Blandino Ortiz, S. Cristallini, O. Lheureux, A. Brasseur, J.-L. Vincent, J. Creteur, F.S. Taccone

##### Erasme University Hospital, Université Libre de Bruxelles, Department of Intensive Care, Brussels, Belgium

###### **Correspondence:** S. Pozzebon - Erasme University Hospital, Université Libre de Bruxelles, Department of Intensive Care, Brussels, Belgium

**Introduction:** Veno-arterial extracorporeal membrane oxygenation (VA-ECMO) is increasingly used to treat severe cardio-pulmonary failure. Among the possible complications, central nervous system (CNS) events, such as stroke or hemorrhage, are associated with long-term neurologic morbidity and increased mortality. Additionally, peripheral VA-ECMO can result in delivery of hypoxic blood to the brain. The use of cerebral near-infrared spectroscopy (NIRS) can detect cerebral hypoperfusion non-invasively.

**Methods:** We reviewed our institutional VA-ECMO database (n = 159) from November 2008 to December 2015 and identified those patients who were monitored with cerebral NIRS. Sensors were placed on the patients´ foreheads using the Foresight device (CAS Medical Systems, Inc, Branford, USA). Regional saturation (rSO2) was recorded and analysed by calculating the time below different thresholds (60 %, 55 % and 50 %).

**Results:** A total of 39 patients (age: 54 [48–72] years) had cerebral NIRS monitoring during VA-ECMO for cardiogenic shock (n = 19), refractory cardiac arrest (n = 14) and post heart/lung transplantation (n = 6). ECMO was applied for 6 [3–10] days and NIRS monitoring for 3 [2–4] days. Nine (23 %) patients developed an ischemic stroke (8/9 in the anterior circulation) and 6 (15 %) differential hypoxia -lower PaO2 in the upper body than in the lower body, because of normal cardiac output with severe impairment of pulmonary function- during the first 3 days of monitoring. Hospital mortality was 22/39 (56 %). Twenty-seven (69 %) patients had a drop of rSO2 < 60 % for at least 5 % of the NIRS monitoring period, resulting in hemodynamic interventions, which involved increasing pressure, oxygenation, and/or ECMO flow. In the 3/6 patients with differential hypoxia, these interventions were unsuccessful and a veno-arterial-venous ECMO was implemented. Patients developing ischemic stroke had a higher differential right-left rSO2 (11[6–12]% vs. 6 [4–7]%; p = 0.004) than others. Survivors had a shorter rSO2 time < 60 % than non-survivors (6.6[1.2-24.5]% vs. 43 [12–60]%; p = 0.007).

**Conclusions:** Cerebral NIRS monitoring may be helpful in patients undergoing VA-ECMO to detect cerebrovascular events or brain hypoperfusion/reduced oxygen delivery. In these patients cerebral hypoperfusion is associated with a poor outcome.

### A403 Cerebral regional tissue oxygenation in patients with neurocardiac injury after subarachnoid hemorrhage

#### M. Hravnak^1^, K. Yousef^1^, Y. Chang^2^, E. Crago^1^, R.M. Friedlander^2^

##### ^1^University of Pittsburgh, School of Nursing; Department of Acute & Tertiary Care, Pittsburgh, Pennsylvania, PA, USA; ^2^University of Pittsburgh, School of Medicine: Department of Neurosurgery, Pittsburgh, PA, USA

###### **Correspondence:** M. Hravnak - University of Pittsburgh, School of Nursing; Department of Acute & Tertiary Care, Pittsburgh, Pennsylvania, PA, USA

**Introduction:** Although it has been demonstrated previously that patients with aneurysmal subarachnoid hemorrhage (aSAH) may also experience neurocardiac injury, the impact of this complication on cerebral and peripheral tissue perfusion is not well understood.

**Objectives:** We aimed to determine if neurocardiac injury as defined by increased levels of cardiac troponin I (cTnI) was associated with changes in regional cerebral (CrSO_2_) and peripheral (PrSO_2_) tissue oxygen saturation as measured with continuous near-infrared spectroscopy (NIRS) across days 1–3 after aSAH.

**Methods:** Longitudinal prospective analysis of 13 patients with aSAH. Inclusion criteria: age 21–75 years, spontaneous aneurysm rupture, Fisher grade >1 and/or Hunt and Hess grade >2, and with NIRS data. Exclusion: traumatic SAH, and recent myocardial dysfunction. cTnI was measured at least daily. Continuous non-invasive CrSO_2_ and PrSO_2_ commenced immediately following study enrollment. CrSO_2_ was acquired utilizing two separate sensors affixed to the left and right forehead, while PrSO_2_ was obtained utilizing a single sensor attached to either the left or right thenar eminence. Continuous CrSO_2_ and PrSO_2_ values were recorded every 5 milliseconds and then averaged on hourly intervals for statistical analyses. The daily peak values of cTnI were used in the analysis as a time-varying variable. The hourly averaged CrSO_2_ and PrSO_2_ values were also treated as time varying variables. Generalized estimating equation (GEE) was used to evaluate the association between cTnI and both right and left CrSO2, as well as PrSO_2_, where cTnI was used as the independent variable and CrSO_2/_PrSO_2_ as dependent variables, while controlling for age and gender.

**Fig. 7 (abstract A403).**
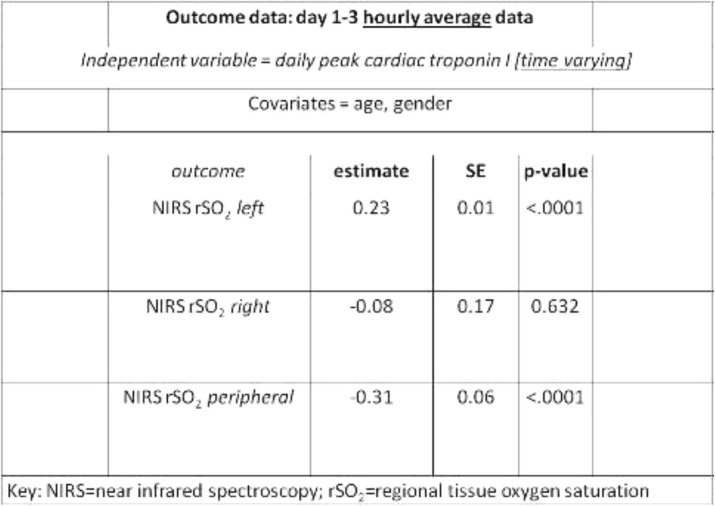
ᅟ

**Results:** The 13 patients in the analysis had a mean age of 56.6 years, SD = 8.9, and were predominantly female (85 %). The GEE modeling revealed that higher peak cTnI was significantly associated with lower PrSO_2_ and higher left-sided CrSO_2_. Higher cTnI was also associated with a trend toward lower CrSO_2_ on the right side, but that was not statistically significant (Fig. 7).

**Conclusions:** Neurocardiac injury after aSAH as defined by higher levels of cTnI is associated with impaired peripheral perfusion, but its impact on cerebral perfusion is variable. Further investigation in a larger sample is needed to see if site of the aneurysm, cerebral vascular autoregulation, or vasospasm may play a role.

**Grant acknowledgment**

NIH R01NR014221

### A404 Study of the incidence of convulsive and non-convulsive seizures in the acute phase of ischemic cerebrovascular stroke

#### S.A. Abdelmonem^1^, S.A. Tahon^2^, T.A. Helmy^1^, H.S. Meligy^1^

##### ^1^University of Alexandria, Criticalcare, Alexandria, Egypt; ^2^University of Alexandria, Neurology, Alexandria, Egypt

###### **Correspondence:** S.A. Abdelmonem - University of Alexandria, Criticalcare, Alexandria, Egypt

**Introduction:** Stroke is a major health problem including both ischemic and hemorrhagic types. Although a long-recognized clinical phenomenon, there remain many questions regarding the epidemiology of seizures and epilepsy after ischemic stroke, their effect on outcome, and their treatment [1].

**Objectives:** This pilot study assesses the incidence of seizures in acute ischemic cerebrovascular disease.

**Methods:** The study was carried out on all patients presented within the first 24 of ischemic cerebrovascular stroke admitted to the units of Critical Care Medicine, Alexandria Main University Hospital, during a period of 6 months. EEG was performed in the first 24 of presentation, at the end of the first and second week of admission and if the level of consciousness deteriorated at any time during the acute phase of cerebral infarction and not explained by CT findings or any metabolic derangement.

**Results:** Among all the study group, the incidence of overall seizures was 20 %, of them, incidence of non-convulsive seizures were 13.3 % compared to 6.6 % of the patients showed convulsive seizures. Most seizures occurred on the first 24 hours of ischemia (66.7 %) compared to those occurring at the end of first week (25 %) and those at the end of the second week (8.3 %).

**Conclusions:** Ischemic stroke is considered as a risk factor for the development of seizures and status epilepticus both convulsive and non-convulsive types especially during the first twenty-four hours.

**References**

1- WHO. Stroke 1989. Recommendations on stroke prevention, and therapy. Report of the WHO Task Force on stroke and other cerebrovascular disorders. Stroke; a journal of cerebral circulation 1989; 20:1407–31

**Grant acknowledgment**

Thanks to all members of the Department of Critical Care Medicine, University of Alexandria

## Contemporary issues in infection and sepsis I

### A405 Does plasma from septic patients influence tumor cell growth?

#### F. Puig^1^, I. Dunn-Siegrist^1^, J. Pugin^1,2^

##### ^1^University of Geneva, Department of Microbiology and Molecular Medicine (MIMOL), Geneva, Switzerland; ^2^Geneva University Hospital, Division of Intensive Care, Geneva, Switzerland

###### **Correspondence:** F. Puig - University of Geneva, Department of Microbiology and Molecular Medicine (MIMOL), Geneva, Switzerland

**Introduction:** Sepsis is associated with immune suppression, including depressed cellular immunity. A decrease in surveillance by immune cells could be one of the mechanisms explaining the growing perception that patients surviving sepsis are at increased risk of developing cancers. However, other mechanisms may be involved in this increased rate of tumors in sepsis survivors, involving humoral factors. Sepsis is also associated with marked up- or down-regulation of various soluble mediators affecting cell growth. We hypothesized that some of these mediators may promote the proliferation of tumors.

**Objective:** To identify and isolate this (those) plasma factor(s) enhancing tumor cell growth.

**Methods:** Heparinized human blood samples were collected from patients with septic shock and in healthy donors. Human adenocarcinoma epithelial cells from lung (A549), colon (SW620), breast (Hs578T), and liver (HepG2) and human monocytic leukemia cells (THP-1 and HL-60) were grown to 70 % confluence and incubated with 5 - 15 % septic or healthy plasma, or control media. After 24 hours, cell proliferation was evaluated by MTT Cell Proliferation Assay. Septic and healthy plasma fractionation was performed using classical biochemical techniques.

**Results:** Septic plasma supported significantly higher epithelial cell proliferation than plasma from healthy subjects (+48 % in A549, +59 % in SW620, +41 % in Hs578T, and +29 % in HepG2 cells with 15 % plasma). Interestingly, this effect was not observed in tumoral cells of monocytic origin. Adding healthy plasma to septic plasma decreased the tumor epithelial cell proliferation effect seen with septic plasma alone in a dose-dependent manner. Initial plasma fractionation studies suggest that the proliferation factor(s) in septic plasma remains in an immunoglobulin- and albumin-free plasma fraction, is(are) not precipitable by 50 % ammonium sulfate, and is(are) a trypsin-sensitive protein(s) > 50 kDa.

**Conclusions:** The identification of such plasma protein(s) affecting tumor cell growth could be of great interest in the fields of sepsis and cancer biology.

**Grant acknowledgment**

Unrestricted research grant from Novartis Research Foundation (15B094).

### A406 High Flow Nasal Cannula (HFNC) as an alternative to noninvasive ventilation (NIV) in acute respiratory failure (ARF) in immunosuppressed patients - an Indian post liver transplant experience

#### S. Gupta, D. Govil, S. Srinivasan, S.J. Patel, J.K. N, A. Gupta, D.S. Tomar, M. Shafi, R. Harne, D.P. Arora, N. Talwar, S. Mazumdar

##### Medanta - The Medicity, Gurgaon, India

###### **Correspondence:** S. Gupta - Medanta - The Medicity, Gurgaon, India

**Introduction:** In immunocompromised patients, acute respiratory failure (ARF) is associated with high mortality [1] and many studies have confirmed the utility of prolonged non invasive ventilation (NIV) in such condition. Lately High Flow Nasal Cannula (HFNC) has been used in ARF in various patient subsets but not specifically in post transplant patients.

**Objectives:** To compare HFNC vs NIV as the modality to manage ARF in postoperative hypoxemia in post liver transplant patients.

**Methods:** This was a pilot study conducted in a Liver Transplant intensive care unit (ICU) of a tertiary care hospital in India. We randomly assigned 20 consecutive post transplant patients who developed respiratory failure in the post-operative period to either HFNC group (n = 10) or to NIV group (n = 10). The HFNC was initiated at a flow rate of 60 l/min whereas NIV was set at EPAP of 5 cm and IPAP at 10 cm. Both the device setting and oxygen titration was done according to arterial blood gas (ABG) analysis. Apart from ABG analysis, we assessed the COMFORT scale as well as the RASS and CAM-ICU scale of the patient and also assessed the total nutritional deficit at the end of 48-hr therapy duration. The need for invasive mechanical ventilation (IMV) was also noted for both the groups.

**Results:** None of the patients in the HFNC group required need for IMV whereas 2 patients in NIV group had to be intubated. Patients in the HFNC group had better average PaO2 as compared to NIV (98.2 vs 72.6 mm Hg) respectively. The patients in the NIV group received more sedation with dexmedetomidine as compared to HFNC group (22 vs 10 hrs) respectively. The patients in the HFNC group were more comfortable as compared to NIV group and two patients in the NIV group developed delirium for which they required IMV. The average RASS score was 0 to +1 in the HFNC group whereas it ranged from −2 to +2 in the NIV group. All patients were fed either orally or enterally but the NIV group consumed less feeding due to the inability to feed orally and apprehension of aspiration due to aerophagia when fed enterally. The NIV group received 52 % less calories as compared to HFNC group in 48-hr period.

**Conclusions:** HFNC is may be an excellent armamentarium for managing hyperemic respiratory failure in immunocompromised patients with reduced risk of intubation, more comfortable to the patients and little interruption in providing adequate nutrition.

**References**

1. Lemaire V, Mokart D, Mayaux J, Lambert J, Rabbat A, Demoule A, Azoulay E. The effects of a 2-h trial of high-flow oxygen by nasal cannula versus venturi mask in immunocompromised patients with hypoxemic acute respiratory failure: a multicentre randomized trial. Crit Care 2015 Nov 2;19:380

**Fig. 8 (abstract A406).**
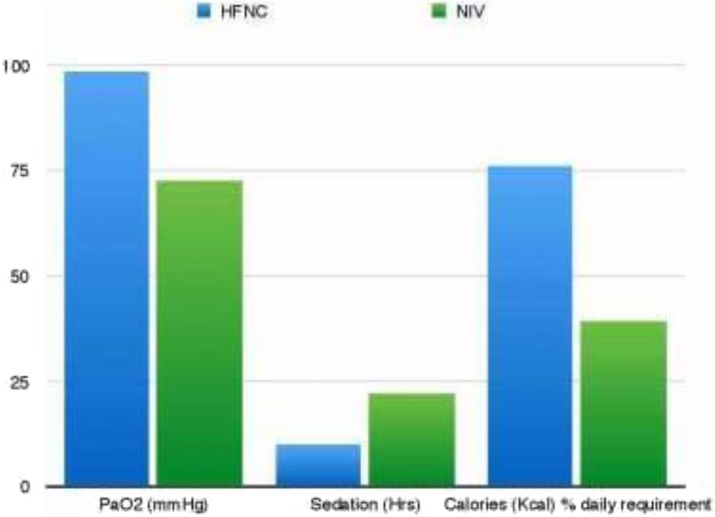
HFNC vs NIV

### A407 Intra-abdominal hypertension increases the frequency of ventilator associated pneumonia

#### E.E. Papakrivou, D. Makris, E. Manoulakas, B. Tsolaki, B. Karadodas, E. Zakynthinos

##### University of Thessaly School of Medicine, Department of Critical Care Medicine, Larisa, Greece

###### **Correspondence:** E.E. Papakrivou - University of Thessaly School of Medicine, Department of Critical Care Medicine, Larisa, Greece

**Introduction:** To study the effect of intra-abdominal hypertension (IAH) on the frequency of ventilator associated pneumonia (VAP) in critical care patients with risk factors for IAH.

**Methods:** This one-center prospective study was conducted in the ICU of the University Hospital of Larissa, Greece. Consecutive critical care patients were recruited if they presented risk factors for IAH. Patients were evaluated systematically for IAH and VAP for a 28-day period.

**Results:** Twenty-four out of 59 (41 %) patients presented IAH and 28 (47.4 %) presented VAP; seventeen (70.83 %) patients presented VAP following IAH. Multivariate analysis showed that VAP [1.18(1.10-1.22) (p = 0.004)], COPD [1.28(1.09-1.86) (p = 0.001)] and the use of antacids [9.54(2.74-33.19) (p = 0.016)] were independently associated with IAP.

**Conclusion:** IAH may have an adverse impact on the frequency of VAP in critically ill patients with risk factors for IAH.

### A408 Azithromycin modulates inflammatory response in a murine model of Pseudomonas aeruginosa severe infection

#### I. Palacios Garcia, A. Diaz Martin, V. Sanchez Encinares, M. Pachón Ibañez, J. Garnacho Montero, G. Labrador, T. Cebrero Cangueiro

##### Hospital Virgen del Rocio, Sevilla, Spain

###### **Correspondence:** I. Palacios Garcia - Hospital Virgen del Rocio, Sevilla, Spain

**Objectives:** Macrolides, apart from its antibiotic properties, are able to modulate the inflammatory response: inhibit production and secretion of pro-inflammatory cytokines (IL-1, IL-6, IL-8 and TNF-α), increase levels of anti-inflammatory cytokines (IL-10) and inhibit secretion of nitric oxide (NO). Our working hypothesis is that the use of azithromycin (AZM) with ceftazidime (CFZ) in a mouse model of severe sepsis by *P. aeruginosa*, modulates the inflammatory response.

**Methods:** lethal sepsis model mouse with a clinical P. aeruginosa strain (Pa4) is characterized. i) CFZ (dose 100 mg / kg / ip / 12 h), ii) AZM (30 mg / kg / ip / 24 h): To study the inflammatory response, the following treatment groups (n = 15) for 72 hours were performed iii) COMB: CFZ + AZM, iv) control of infected animals and untreated group (CON) and v) group of uninfected mice. TNF-α determinations, IL-6, IL-10 and nitrite / nitrate (NOx) routine employed as a surrogate marker of NO metabolism in mouse plasma by ELISA (commercially available kits) ratio were performed.

**Results:** We compare the TNF-α, IL-10 and NOx plasma concentrations, in each group (4 and 8 hours post-treatment) related to those obtained in the CON group.

The COMB (AZM + CFZ) group showed lower plasma concentrations of TNF-α (pg/ml) than AZM and CFZ groups: [CON: 1477–716; COMB: 720–567; AZM: 1911–1663; CFZ: 793–666].

Plasma concentrations of IL-10 (pg/ml) were higher in the COMB and AZM groups than in the CFZ one: [CON: 1868–1761; COMB: 1541–2035; AZM: 1860–2002; CFZ: 1898–1886].

NOx concentrations (M) observed were lower in the COMB group than in AZM and CFZ ones: [CON: 76–47; COMB: 59–28; AZM: 61–51; CFZ: 35–42].

**Conclusions:** These results suggest the immunomodulatory capability of AZM as an adjunct treatment to appropriate antibiotic. Further studies are needed to infer these findings to human setting.

**Grant acknowledgment**

This project (PI10/01563) was funded by the "Health Research Fund" Health Institute Carlos III.

### A409 Intravenous glutamine increases risk of death in severe sepsis

#### V. Poulose^1^, J. Koh^1^, J.W. Kam^2^

##### ^1^Changi General Hospital, Respiratory & Critical Care Medicine, Singapore, Singapore; ^2^Changi General Hospital, Clinical Trials & Research Unit, Singapore, Singapore

###### **Correspondence:** V. Poulose - Changi General Hospital, Respiratory & Critical Care Medicine, Singapore, Singapore

**Introduction:** Intravenous glutamine can have beneficial effects on critically ill patients by preserving gut barrier and improving immune function.

**Objectives:** We wanted to prove the benefit of intravenous glutamine in patients admitted to medical intensive care unit (MICU) with severe sepsis and receiving enteral nutrition.

**Methods:** Randomized, single center, double -blind, placebo-controlled, pilot study on patients admitted to the MICU who met the criteria for severe sepsis. In the intervention arm, intravenous glutamine was given for 5 days at a dose of 0.5 g/kg body weight/day. All patients were fed enterally as per the MICU feeding protocol. The primary outcomes were 28-day mortality and the occurrence of new infections. We also looked at severity scores (SOFA), ICU length of stay (LOS), hospital LOS and duration of mechanical ventilation.

**Results:** Thirty nine patients were randomized to receive glutamine (n = 19) or placebo (n = 20). The glutamine group had less disease severity than placebo (median SOFA score 8 versus 11, p =0.038)*.* There was no difference in 28-day mortality between the glutamine and placebo groups (42 % vs 15 %, p = 0.06). When adjusted for disease severity, the glutamine arm had 5.6 times higher death rates (95 % CI 1.1-30.2, p = 0.044). The glutamine group had lesser incidence of new infections (0 % vs 30 %, p = 0.02). There was no difference in ICU LOS, hospital LOS or the duration of mechanical ventilation.

**Conclusions:** Intravenous glutamine increases mortality risk in ICU patients with severe sepsis, although it reduces risk of new infections

**Grant acknowledgement**

SingHealth Foundation Research Grant

### A410 Sequential vitamin d measurement in patients with septic shock: could vitamin D levels be suppressed in septic shock?

#### H. Yeter^1^, A. Kara^2^, O. Aktepe^3^, A. Topeli^4^

##### ^1^Hacettepe University, Internal Medicine, Ankara, Turkey; ^2^Hacettepe University, Intensive Care Medicine, Ankara, Turkey; ^3^Hacettepe University, Internal medicine, Ankara, Turkey; ^4^Hacettepe University, Intensive care medicine, Ankara, Turkey

###### **Correspondence:** H. Yeter - Hacettepe University, Internal Medicine, Ankara, Turkey

**Objective:** Sepsis is characterized by dysregulated immune response to infection leading to organ dysfunction. Vitamin D plays a pivot role in the immune system and low levels of vitamin D have been shown to be associated with worse outcome in septic patients. In this study we tested vitamin D levels sequentially and aimed to define the vitamin D response in septic shock.

**Methods:** Between September 2014 and January 2016, 41patients with septic shock were included in the study. Patients were excluded if they had a disease affecting calcium and vitamin D metabolism such as malignancy, chronic kidney disease, parathyroid disorders, pancreatitis, tumor lysis syndrome, rhabdomyolysis, renal tubular disorders and pregnancy. We measured vitamin D levels in day 1 and day 5after diagnosis of septic shock.

**Results:** The median (min-max) age of 41 patients was 67 (19–88). 21of these patients were male. The median APACHE II score was 28(11–45). Day 1 and day 5 median vitamin D levels were 6.8 ng/ml(1–30) and 12 (2–29) ng/ml, respectively. Baseline corrected calcium and ionized calcium levels were 9.04 mg/dl (4.70-11.50) and 1.09 mmol/L (0.80-1.22). 21of the 41 septic shock patients died within 28 day. The APACHE II scores were similar between the survivor and non-survivor groups [27.5 (11–40), 30 (11–45), p = 0.13]. Baseline median corrected calcium and ionized calcium levels of survivors vs. non-survivors were 9.01 mg/dl (8.3-10.0) vs 9.16 mg/dl (4.7-11.5) and 1.07 mmol/L (0.90-1.18) vs. 1.10 mmol/L (0.80-1.22). Day 1 median vitamin D levels of survivors and non-survivors were 8.7 ng/ml(4.3-30.4) and5.3 ng/ml (1.0-21.7), respectively(p = 0.047). Day 5 vitamin D levels were not statistically different between survivors and non-survivors (n = 17; 12.3 ng/ml and n = 7; 5.7 ng/ml, p = 0.37).Vitamin D levels increased in the survivor group from 8.7 ng/ml in day 1 to 12.4 ng/m lin day 5, however the difference was not statistically different (p = 0.18). Vitamin D levels did not change in the non-survivor group from day 1 to day 5 (5.3 ng/ml to 5.7 ng/ml, p = 0.89).Kaplan Meier survival analysis revealed that patients with vitamin D levels ≥ 6.8 ng/ml (median value) had increased 28-day survival as compared to patients with low vitamin D levels (<6.8 ng/ml) (long rank test p = 0.012).

**Conclusion:** Our study showed that vitamin D response might be different between surviving and non-surviving patients during the course of septic shock. Surviving patients had higher day 1vitamin D levels as compared to non-surviving patients. Increase in vitamin D level from day 1 to 5 suggests a clinically significant albeit statistically insignificant association of vitamin D and survival, in line with high vitamin D levels in baseline comparisons of the groups, favoring survivors, due to the limited sample size. Further studies in larger groups are clearly warranted.

### A411 Central line associated blood stream infections in the obese and overweight critically ill patients (preliminary data)

#### I. Tsolakoglou^1^, G. Intas^2^, P. Stergiannis^3^, A.A. Kolaros^4^, E. Chalari^5^, E. Athanasiadou^6^, A. Martika^1^, G. Fildisis^7^

##### ^1^General Hospital of Thessaloniki Agios Pavlos, ICU, Thessaloniki, Greece; ^2^General Hospital of Nikaia-Pireus AG. Panteleimon, Actinotherapy, Pireus, Greece; ^3^General Hospital of Athens “Agioi Anargyroi, ICU, Athens, Greece; ^4^General Hospital of Thessaloniki Theageneio, ICU, Thessaloniki, Greece; ^5^General Hospital of Nikaia-Pireus AG. Panteleimon, Anesthesiology, Pireus, Greece; ^6^General Hospital of Thessaloniki Agios Pavlos, Nursing Department Management, Thessaloniki, Greece, ^7^University of Athens, Faculty of Nursing, Critical Care Directorate, Athens, Greece

###### **Correspondence:** I. Tsolakoglou - General Hospital of Thessaloniki Agios Pavlos, ICU, Thessaloniki, Greece

**Introduction:** Central-Line-Associated Bloodstream Infections (CLABSI) have been studied extensively in ICU patients. There are no data in the literature regarding a potential association between obesity and CLABSI.

**Objectives:** To test the hypothesis that CLABSI depends on the presence of obesity in critically ill patients.

**Methods:** We conducted an 18-month observational study on 576 critically ill patients, in three general ICUs in Greece. All patients had inserted a triple-lumen catheter in a central vein (internal jugular, femoral or subclavian). Body Mass Index (BMI) was determined by a dietitian on ICU admission. BMI was categorized a priori as < 18.5 (underweight), 18.5-24.9 (normal weight), 25–29.9 (overweight), and >30 (obese). CLABSI was diagnosed by examining the catheter's tip and a blood sample. Multivariate logistic regression analysis was used to estimate the association between BMI groups and CLABSI.

**Results:** From the 576 critically ill patients, (258 men, 318 women) mean aged 62.3 ± 18.4 years, 28 (4.9 %) were underweight, 220 (38.2 %) normal weight, 234 (40.6 %) overweight and 94 (16.3 %) obese. CLABSI was diagnosed in 156 (27.1 %) patients. Overweight and obese patients had significant higher CLABSI rates than the other patients (p < 0.05). Obese patients had significantly less survival rates (p < 0.05). Patient's data according to the BMI category are shown on Table 1. Additional adjustment for obesity-central line catheter association for the presence of CLABSI attenuates the obesity- central line catheter association: underweight CLABSI OR = 1.79 (95 % CI 1.54-2.03; p = 0.006); normal weight CLABSI OR = 1.86 (95 % CI 1.80-1.93; p = 0.003); overweight CLABSI OR = 1.63 (95 % CI 1.54-1.72; p = 0.001); obese- CLABSI OR = 1.64 (95 % CI 1.50-1.78, p = 0.001).

**Conclusions:** Obesity appears to be associated with the presence of CLABSI in critically ill patients. This could be partially attributed to the more efforts made by physicians to insert the catheter in the obese patients than the other patients.

**REFERENCE(S)**

Tagliabue C, Principi N, Giavoli C, Esposito S. Obesity: impact of infections and response to vaccines. Eur J Clin Microbiol Infect Dis. 2016 Mar;35(3):325–31.

**Table 1 (abstract A411).** Patient's characteristics according to BMI categor

	Underweight (N=28)	Normal weight (N=220)	Overweight (N=234)	Obese (N=94)	p
Apache II score	23.7±7.7	20.5±6.9	23.2±6.9	26.8±5.7	0.001
Femoral vein catheter insertion, n (%)	10 (35.7%)	30 (13.6%)	28 (11.9%)	14 (14.9%)	0.025
Subclavian vein catheter insertion, n (%)	10 (35.7%)	142 (64.5%)	156 (65%)	54 (61.4%)	0.025
Jugular vein catheter insertion, n (%)	8 (28.6%)	48 (21.9%)	56 (21.1%)	20 (23.7%)	0.025
N of attempts for catheter insertion	1.3±0.6	1.1±0.2	2.1±0.6	3.3±1.2	0.028
CLABSI, n (%)	6 (21.4%)	30 (13.6%)	86 (36.8%)	34 (36.2%)	0.001
ICU LOS	11.4±11.8	15.1±10.7	23.1±16.5	24.4±10.4	0.001
Hospital LOS	16.2±14.1	21.1±10.6	28.9±12.3	29.3±15.4	0.001
Survival, n (%)	16 (42.9%)	148 (67.3%)	94 (40.2%)	8 (8.5%)	0.001

### A412 Reactive oxygen species (ROS) production and leukocyte immunoglobulin-like receptors (LILR) expression by immune cells in pleural fluid (PL) and blood (BL) in critical care septic patients

#### V. Faivre^1^, C. Mengelle^1^, B. Favier^2^, D. Payen^1,3^

##### ^1^Université Paris Diderot, Sorbonne Paris Cité, INSERM UMR1160, Paris, France; ^2^Université Paris Sud / CEA, Inserm U1184, Fontenay Aux Roses, France; ^3^APHP, Lariboisiere University Hospital, Surgical ICU, Paris, France

###### **Correspondence:** V. Faivre - Université Paris Diderot, Sorbonne Paris Cité, INSERM UMR1160, Paris, France

**Introduction:** Sepsis induces a hyperinflammatory phase with a concomitant immunosuppression that predominates after initial phase and exposes to a failure of controlling primary infection, or to an increased risk of secondary infections. Blood monocyte (Mo) HLA-DR expression (MHC Class II) seems to characterize well innate immunodepression. The LILR expression on immune cells interacting with HLA class I induces a functional inhibition of neutrophils (PMN) as shown by the expression of LILR B2 modified during sepsis^1^.

**Objectives:** To investigate the expression of LILR subtypes on blood Mo CD16- (classical Mo) and Mo CD16+ (non-classical patroller) as on PMNs and eosinophils (Eo) *vs* expression on Pl cells, more related to lung tissue inflammation^2^, during sepsis.

**Methods:** In patients having severe sepsis or septic shock: 1/(flow cytometry)LILR A2, LILR B1, LILR B2, LILR B3, LILR B4 and HLA I on Mo CD16-, Mo CD16+, PMN (CD16+ Granulocytes (Gr)) and CD16- Gr in Bl and Pl. 2/ (chemiluminescence^3^) Spontaneous and stimulated ROS production. Measurements done at the 1^st^ collection of PL (T1) and a week after (T2) when possible. Statistical analysis: non parametric tests. Ethical committee: CPP Ile De France VII, n° PP 15–010.

**Results:** 11 patients were enrolled, vs 7 healthy volunteers (HV, Bl only). Compared to HV, in Bl, HLA-DR expression decreased in patients on Mo CD16- (p < 0,05) with a higher ROS production (p < 0,05). HLA-DR expression was higher on Mo CD16+. All LILR expressions were significantly higher on Mo CD16-, Mo CD16+, PMNs, except for LILRB1, with a decrease in their ligand HLA-I (p < 0,05). On Gr CD16-, LILR B1 only was increased. Comparison between Bl and Pl showed no differences except for LILRB3 expression being higher on PMNs with a higher HLA-DR expression on Mo CD16-. All LILR expressions decreased between T1 and T2 (p < 0,05) regardless the patients prognosis.

**Conclusions:** The increased LILR expression in Bl myeloid cells during sepsis was similar in Pl cells, with a decrease along time without relation with prognosis. LILR expression could be used both in Mo and PMNs in association with Bl Mo HLA-DR to characterize immunodepression and the potential therapy for immunomodulation.

**References**

1. Baudhuin J et al. PNAS 2013, 110: 17957–17962.

2. Marie C et al. Am. J. Respir. Crit. Care Med. 1997, 156: 1515–1522.

3. Lukaszewicz et al. Ann Crit Care 2012; 2: 10.

**Grant acknowledgement**

Fondation pour la Recherche Médicale, Commissariat à l'Energie Atomique, Ministère de l'Enseignement Supérieur et de la Recherche.

### A413 The impact of red blood cell (RBC) transfusion on sphingosine-1-phosphate (S1P) levels of critically ill patients

#### A. Poppe^1^, M.S. Winkler^1^, E. Mudersbach^2^, J. Schreiber^3^, M.-L. Wruck^1^, E. Schwedhelm^2^, S. Kluge^3^, C. Zöllner^1^

##### ^1^University Medical Center Hamburg-Eppendorf, Department of Anaesthesiology, Hamburg, Germany; ^2^University Medical Center Hamburg-Eppendorf, Institute of Clinical Pharmacology and Toxicology, Hamburg, Germany; ^3^University Medical Center Hamburg-Eppendorf, Department of Intensive Care Medicine, Hamburg, Germany

###### **Correspondence:** A. Poppe - University Medical Center Hamburg-Eppendorf, Department of Anaesthesiology, Hamburg, Germany

**Introduction:** Sphingosine-1-phosphate (S1P) is a G-protein coupled signaling lipid. In particular S1P has been shown to reduce sepsis induced endothelial leakage and cytokine release from immune cells and to regulate vascular tone.

Among others these processes are responsible for the high mortality of critically ill patients. Therefore S1P has been studied in critically ill patients; lately low S1P concentrations have been associated with sepsis severity by our group [1].

S1P is mainly released by haematopoietic cells such as erythrocytes (EC) and thrombocytes (TC) [2]. EC produce and secrete S1P continuously, whereas TC produce S1P but release S1P only upon activation [3].

**Objective:** We were interested if S1P concentration in blood of critically ill patients may be influenced by red blood cell (RBC) transfusion.

**Methods:** S1P concentration in serum and EDTA plasma of 28 critically ill patients was measured before and after transfusion of one RBC preservation at several times (pre and 30 min, 180 min, 24 h post). S1P was also measured in the transfused RBC preservation. Differences between transfusion of “fresh” (RBC ≤ 15d) and “old“(RBC > 15d) RBC were analysed.

Clinical data like SOFA scores, haemoglobin (HB) and haematocrit (Hct) values of all patients were monitored repeatedly.

**Results:** S1P concentration was higher in RBC preservations compared to serum and plasma levels in patients (P < 0,05). Serum S1P levels were higher compared to plasma S1P levels (p < 0,05), though serum and plasma levels correlated positively during time. S1P concentration in plasma declined 30 min after transfusion (p < 0,05) and increased to pre levels within 24 h. Serum levels did not change.

Comparing “fresh” and “old” blood preservations, S1P concentration was significantly higher in “fresh” RBC (P < 0,05). However in patients, blood S1P values did not differ if “fresh” or “old” RBCs have been transfused.

**Conclusions:** RBC preservations contain high amounts of S1P compared to patient´s endogenous S1P. S1P concentration in RBC preservations correlates negatively with duration of storage. Transfusion of RBC influences plasma S1P negatively within 30 min. S1P declines immediately after transfusion and increases thereafter during the next 24 h. Serum S1P levels are not changed by RBC transfusion.

However alteration of plasma S1P levels by RBC transfusion had no influence on the clinical outcome of our patients.

**References**

1. Winkler, M.S., et al., *Decreased serum concentrations of sphingosine-1-phosphate in sepsis.* Crit Care, 2015. **19**: p. 372.

2. Hanel, P., P. Andreani, and M.H. Graler, *Erythrocytes store and release sphingosine 1-phosphate in blood.* FASEB J, 2007. **21**(4): p. 1202–9.

3. Yatomi, Y., et al., *Sphingosine-1-phosphate: a platelet-activating sphingolipid released from agonist-stimulated human platelets.* Blood, 1995. **86**(1): p. 193–202.

### A414 Intensive care adult and pediatric patients at risk of mortality: inflammatory - immunity and metabolic profiles

#### T. Tavladaki^1^, A.M. Spanaki^1^, H. Dimitriou^2^, E. Kondili^3^, C. Choulaki^4^, E. Meleti^2^, D. Kafetzopoulos^4^, D. Georgopoulos^3^, G. Briassoulis^1^

##### ^1^University of Crete, Medical School, University Hospital, PICU, Heraklion, Greece; ^2^University of Crete, Medical School, Paediatric Haematology Oncology, Heraklion, Greece; ^3^University of Crete, Medical School, University Hospital, ICU, Heraklion, Greece; ^4^Institute of Molecular Biology and Biotechnology (IMBB), Foundation for Research and Technology - Hellas (FORTH), Heraklion, Greece

###### **Correspondence:** T. Tavladaki - University of Crete, Medical School, University Hospital, PICU, Heraklion, Greece

**Introduction:** Biomarkers influenced by tissue utilization, clearance, and metabolic derangements have not been shown to be able to identify complex cellular abnormalities of critical illness pathobiology.^1^ Currently there are no comparative data on metabolic and innate immunity - inflammatory changes in adult (A) and pediatric (P) patients with early-onset sepsis or systemic inflammatory response syndrome (SIRS).

**Objectives:** To determine the «at risk of mortality» profiles in A and P intensive care unit (ICU) patients in comparison to ICU-survivors and healthy (H) controls. To assess possible associations of heat shock proteins (HSP) and Resistin and Adiponectin hormone changes or oxygen consumption (VO2), dioxide production (VCO2), energy expenditure (EE) and metabolic pattern alterations with mortality in the early phase of critical illness.

**Methods:** Seventy-eight adults (S/22; SIRS/23; H/33) and 67 children (S/18; SIRS/23; H/26) mechanically ventilated were included in the study. Blood samples were obtained within 24-hours upon admission. Mean Fluorescence Intensity (MFI) for HSP expression in monocytes (m) or neutrophils (n) was determined by Flow Cytometry. Restistin, Adiponectin and extracellular (e) HSP72 were measured using ELISA and energy-expenditure (EE) by E-COVX. Genomic DNA was extracted with PureLink Genomic DNA kit to detect HSP72 SNPs.

**Results:** More patients with septic shock (p < 0.005) or patients with lactate >2 mmol/L (p < 0.02) were recorded among non-survivors (A 12.7 % vs. P 7.4 %) compared to survivors. APACHEII, eHSP72, troponin, and lactate levels were higher and VO_2_, VCO_2_, EE, metabolic pattern, and albumin lower among non-survivors compared to survivors in both groups (p < 0.05). Also, non-survivors P had higher PELOD, Resistin, and anion gap and lower EF (p < 0.05); non-survivors A showed a trend for higher eHSP72, nHSP72, and Resistin levels. In both age groups, the rs6457452 and rs1061581 HSP72 haplotypes were not related to mortality. In a logistic regression model, high lactate in A and resistin levels in P were independently associated with mortality (p < 0.01). For predicting sepsis-3 in both groups, low VO_2_, VCO_2_, metabolic pattern, and albumin and high lactate levels achieved a receiver operating characteristic curve >0.7 (p < 0.05).

**Conclusions:** Intensive care adult and pediatric patients at risk of mortality show similar inflammatory - immunity and metabolic profiles not influenced by the rs6457452 and rs1061581 HSP72 SNPs. High lactate levels and hypometabolism better predict mortality in both groups.

**Grant acknowledgement**

This research has been co-financed by the European Union (European Social Fund (ESF)) and Greek national funds through the Operational Program ´´Education and Lifelong Learning´´ of the National Strategic Reference Framework (NSRF)-Research Funding Program: THALES.

### A415 Prognosis value of immunoglobulins IGG, IGA and IGM in patients with severe sepsis or septic shock

#### A. García-de la Torre^1^, M.V. de la Torre-Prados^2^, T. Tsvetanova-Spasova^2^, P. Nuevo-Ortega^2^, C. Rueda-Molina^2^, A. Fernández-Porcel^2^, E. Camara-Sola^2^, L. Salido-Díaz^2^, A. García-Alcántara^2^

##### ^1^University Hospital Virgen de la Victoria / IBIMA, Clinical Chemistry Department, Málaga, Spain; ^2^University Hospital Virgen de la Victoria / IBIMA, Department of Intensive Care Unit, Málaga, Spain

###### **Correspondence:** A. García-de la Torre - University Hospital Virgen de la Victoria / IBIMA, Clinical Chemistry Department, Málaga, Spain

**Introduction:** Despite the promise of Ig therapy, the last Surviving Sepsis Guidelines (2012) does not suggest using intravenous Ig in adults with severe sepsis or septic shock, requiring more research in this field.

**Objectives**: Assess the prognostic value of serum immunoglobulins (Igs): IgG, IgA and IgM determined within 24 hours from severe sepsis (SS) or septic shock (SSh) onset.

**Methods:** A cohort study was performed in 133 critically ill adult patients admitted to the Intensive Care Unit (ICU). Demographic variables, severity score, mortality at 28 days were recorded, those patients who had been administered the first dose of intravenous immunoglobulins were excluded. The Igs were determined by nephelometry, Dimension Vista.® Siemens. Statistical analysis was performed using SPSS 15.0 for Windows (SPSS Inc. Chicago, IL, USA).

**Results:** Of the 133 patients enrolled in the study 16.5 % met SS criteria and 83.5 % SSh. The mean age was 62 [inter-quartile range (IR): 48.5-70.5] years, 62.4 % were men. The main sources of infection were: respiratory tract 36.8 % and intra-abdomen 28.6 % The mediam stay in the ICU was 6 [3.5-11] days and mortality at 28 days was 21.8 % (n = 29). Median serum IgG Igs were 792 [607 to 976.5] mg / dl, IgA 204 [147.5 to 315] mg / dl, and IgM 76 [46.5 to 132] mg / dl. Patients who died had significantly higher levels of clinical severity with APACHE II 29.8 vs 24.1, 8.9 vs 12.1 Sequential Organ Failure Assessment (SOFA) and organ dysfunction number 4.6 vs 3.6 and in serum IgA: 323 vs 195 mg / dl. This difference was not statistically significant in levels of IgG 822 vs. 774 mg / dl or IgM 86 vs. 72.5 mg / dl.

**Conclusions:** Serum levels of IgG, IgA and IgM were higher in patients who died, but only IgA showed a prognostic value when was measured within 24 hours from SS or SSh onset.

**References**

**1.** Alejandria MM, Lansang MAD, Dans LF, Mantaring III JB. Intravenous immunoglobulin for treating sepsis, severe sepsis and septic shock (Review). The Cochrane Library 2013, Issue 9, 1–107.

### A416 Similar adult and pediatric inflammatory - immunity and metabolic Sepsis3 profiles compared to SIRS and healthy controls

#### T. Tavladaki^1^, A.M. Spanaki^1^, H. Dimitriou^2^, E. Kondili^3^, C. Choulaki^4^, D.E. Meleti^2^, D. Kafetzopoulos^4^, D. Georgopoulos^3^, G. Briassoulis^1^

##### ^1^University of Crete, Medical School, University Hospital, PICU, Heraklion, Greece; ^2^University of Crete, Medical School, Paediatric Haematology Oncology, Heraklion, Greece; ^3^University of Crete, Medical School, University Hospital, ICU, Heraklion, Greece; ^4^Institute of Molecular Biology and Biotechnology (IMBB), Foundation for Research and Technology - Hellas (FORTH), Heraklion, Greece

###### **Correspondence:** T. Tavladaki - University of Crete, Medical School, University Hospital, PICU, Heraklion, Greece

**Introduction:** The new sepsis definition, known as Sepsis-3, shifted the diagnostic focus from infection with systemic inflammation to infection triggered organ failure, and it does away with the systemic inflammatory response syndrome criteria (SIRS). The role of cellular innate immunity and inflammatory-metabolic abnormalities in S and SIRS development has not been clarified.

**Objectives:** To examine early heat shock proteins (HSP) and Resistin and Adiponectin hormone changes, along with clinical, inflammatory, and metabolic profiles, in adult (A) and pediatric (P) intensive care unit (ICU) patients with sepsis (S). To compare the S profiles with those of SIRS or healthy-controls (H) in each group.

**Methods:** . Seventy-eight adults (S/22; SIRS/23; H/33) and 67 children (S/18; SIRS/23; H/26) mechanically ventilated were included in the study. Blood samples were obtained within 24-hours upon admission. Mean Fluorescence Intensity (MFI) for HSP expression in monocytes (m) or neutrophils (n) was determined using 4-colour Flow Cytometry. Restistin, Adiponectin and extracellular (e) HSP72 using ELISA. E-COVX for energy-expenditure (EE). Genomic DNA was extracted by using the PureLink Genomic DNA kit to detect the polymorphic HSP72 SNPs rs1061581 and rs6457452.

**Results:** Similarly in A and P ICU-patients, APACHEII, SAPS3, HR, CRP, lactate, creatinine, and resistin, were higher and EF, mHSP72, VO2, VCO2, EE, metabolic pattern, glucose, and albumin lower in S compared to SIRS and/or H (p < 0.05). Only PELOD and MAP in P and INR, nHSP72 and glucose in A differed among groups (p < 0.05). For predicting sepsis-3 in both groups CRP, resistin, lactate, and eHSP72 in P, achieved a receiver operating characteristic curve (AUROC) > 0.80 (p < 0.05). In a logistic regression model resistin and mHSP72 independently discriminated S from SIRS in children only (p < 0.001). In A and P, genotype HSP72 analysis did not disclose any group differences regarding the rs6457452 or the rs1061581 haplotypes.

**Conclusions:** Sepsis presents with repressed innate immunity and metabolism and enhanced inflammatory response in adult and pediatric septic patients. These changes may help in depicting a common for adults and children new sepsis-3 profile discriminated from the one of SIRS.

**Grant acknowledgement**

This research has been co-financed by the European Union (European Social Fund (ESF)) and Greek national funds through the Operational Program ´´Education and Lifelong Learning´´ of the National Strategic Reference Framework (NSRF)-Research Funding Program: THALES.

### A417 Role of biomarkers in early postoperative period of lung transplantation

#### B. Suberviola^1^, J. Riera^2^, L. Rellan^3^, M. Sanchez^4^, J.C. Robles^5^, E. Lopez^6^, R. Vicente^7^, E. Miñambres^1^, M. Santibañez^8^

##### ^1^University Hospital Marques de Valdecilla, Santander, Spain; ^2^University Hospital Vall d´Hebron, Barcelona, Spain; ^3^University Hospital A Coruña, La Coruña, Spain; ^4^University Hospital Puerta de Hierro, Madrid, Spain; ^5^University Hospital Reina Sofia, Cordoba, Spain; ^6^University Hospital 12 de Octubre, Madrid, Spain; ^7^University Hospital La Fe, Valencia, Spain; ^8^Nursing School of University of Cantabria, Santander, Spain

###### **Correspondence:** B. Suberviola - University Hospital Marques de Valdecilla, Santander, Spain

**Introduction:** The major reported causes of death within the first 30 days after lung transplantation (LT) are graft failure and non-CMV infections. Delay in starting effective antimicrobiological treatment in case of infections is associated with worse outcome. It is imperative to diagnose the presence of infectious complications after LT in a timely fashion. Biomarkers have been proposed as a tool to improve early diagnosis of infections in this population.

**Objectives:** To determine how sequential measurements of PCT and CRP could improve the diagnosis of early infectious complications after LT.

**Methods:** Multicentre prospective observational study between September 2014 and September 2015. The study included all 7 centres authorized to perform lung transplantation in Spain.Biomarker measurements were executed on ICU admission (day 1) and daily till 7th postoperative day. Patients were split into two groups depending on the presence of infectious complications.

**Results:** Two hundred and thirty three consecutive patients (148 men, median age 56 [range 47 to 61] years) underwent single (n = 108, 46.4 %) or bilateral (n = 125, 53.6 %) LT. In the whole population, both biomarkers, PCT and CRP, presented similar kinetics during study period with an initial increase in their levels, a plasma peak recorded in the first 48 hours and a progressive decline over the next days. PCT plasma levels were similar for PGD grades 1 and 2 and increased significantly in the group of patients with PGD grade 3. CRP levels were similar in all groups. The median PCT levels were significantly lower in patients without infection than in patients with 'Infection' for all days of follow up (Table 2). During the first 5 postoperative days (day 0 to day 4), PCT levels beyond median value were statistically associated with higher risk of infection. Thus, PCT levels beyond 0.50 ng/ml on ICU admission or 1.17 ng/ml on postoperative day 1 were associated with an increase in risk of infection of two-fold and three-fold respectively. Associations remain significant after adjusting by sex, age, need of postoperative ECMO, creatinine levels and type of lung transplant. However, after stratifying by primary graft dysfunction presentation these associations disappeared with respect to the group of patients with PGD 3 and increased in the NO PGD 3 group.Therefore, in the absence of PGD 3, previously described cut-off levels for day 0 and day 1 were significantly associated with an increase of three-fold and four-fold in the risk of infection development.

**Conclusions:** In absence of severe PGD, PCT peak levels are predictor of infectious complications development during the first postoperative week. PGD grade 3 produce an important increase of PCT levels and interfere with PCT infectious diagnosis ability. PCT was superior to CRP in infections diagnosis during this period.

**Grant acknowledgement**

Supported by a grant from the Mutua Madrileña Foundation (FMM 14/01).

**Table 2 (abstract A417).** Median levels for PCT levels for each day

	Overall	NO Infection	Infection in trasplant recipient		NO Dysfunction grade 3	Primary graft dysfunction grade 3	
	N=233	N=181	N=52		N=205	N=28	
PCT LEVELS	Median	Median	Median	P value	Median	Median	P value
ICU Admission	0.50	0.36	2.00	<0.001	0.47	4.57	<0.001
Day 1	1.17	1.01	3.83	<0.001	1.07	4.90	<0.001
Day 2	1.14	0.94	2.73	<0.001	1.01	2.61	0.006
Day 3	0.70	0.55	1.70	<0.001	0.69	1.32	0.402
Day 4	0.56	0.50	0.78	0.002	0.54	1.11	0.105
Day 5	0.28	0.20	0.40	0.006	0.28	0.48	0.680
Day 6	0.20	0.20	0.30	0.026	0.20	0.39	0.447

## PERIOPERATIVE INTENSIVE CARE AND SEDATION

### A418 A pilot study to assess the impact of anxiety and depression during admission in post-operative intensive care patients

#### M. Le Guen, J. Moore, N. Mason

##### Central Manchester Foundation Trust, Critical Care Department, Manchester, UK

###### **Correspondence:** M. Le Guen - Central Manchester Foundation Trust, Critical Care Department, Manchester, UK

**Introduction:** The development of neuropsychological dysfunction in survivors of critical care has been widely documented in the literature (1). In contrast, the impact on patient outcomes of anxiety and depression at the time of critical care admission has been less well documented (2).

**Objectives:** We conducted a pilot study to determine the feasibility of assessing critical care patients' anxiety and depression during their admission and the potential impact of these morbidities on patient outcomes.

**Methods:** Patients were recruited following admission to a general intensive care unit post surgical intervention. Patients had a Hospital Anxiety and Depression Scale (HADS) assessment on day 7 and day 15 post operatively, In addition demographic (gender, age, elective vs emergency surgery, cancer vs non-cancer surgery, prior anti-depressant use), intervention and outcome (length of stay, infection, additional oxygen requirement, in-dwelling catheter in situ) data was collected.

**Results:** One hundred and twenty two patients were recruited to the study and 69 (56.6 %) had a HADS assessment on day 7. Amongst the 52 patients that did not have a HADS assessment completed, 27 (52 %) were discharged prior to day 7 and 16 (31 %) either refused or were not appropriate for assessment.

The completed HADS assessments indicated that 29 % and 30 % of patients had significant anxiety and depression scores respectively. Increased day 7 median anxiety scores were associated with female gender (6 vs 4, P = 0.04), anti-depressant use (8 vs 5, P = 0.01), oxygen requirement (9 vs 5, P = 0.049), infection (7 vs 4, P = 0.05) and urinary catheter use (8 vs 4, P = 0.04). Increased median depression scores were associated with female gender (7 vs 5, P = 0.04) and hospital stay longer than 14 days

(7 vs 5, P = 0.007).

Twenty-four patients had a further HADS assessment on day 15, with 35 patients having been discharged from hospital in the intervening period. Based on Wilcoxon Signed Ranks tests, anxiety but not depression scores appeared to decrease during the patient's hospital stay (z = 2.03, P = 0.04 and z = 0.25, P = 0.8, respectively).

**Conclusions:** The assessment of anxiety and depression amongst peri-operative critical care patients is feasible. Increased anxiety levels appear associated with a number of interventions. Increased depression scores appear associated with increased hospital length of stay. Anxiety but not depression levels appear to decrease over time during patients' hospital stays.

**References**

1. Parker, Ann M., et al. "Posttraumatic Stress Disorder in Critical Illness Survivors: A Metaanalysis*." *Critical care medicine* 43.5 (2015): 1121–1129.

2. Rincon, Hernan G., et al. "Prevalence, detection and treatment of anxiety, depression, and delirium in the adult critical care unit." *Psychosomatics* 42.5 (2001): 391–396.

**Grant acknowledgement**

No grants were accepted or used to support this work

### A419 Opioid use after propofol or sevoflurane anesthesia: a randomized trial

#### M. Windpassinger^1^, O. Plattner^1^, E. Mascha^2^, D.I. Sessler^2^, Outcomes Research

##### ^1^Medical University of Vienna, Anesthesiology & Intensiv Care, Vienna, Austria; ^2^Cleveland Clinic, Outcomes Research, Cleveland, OH, USA

###### **Correspondence:** M. Windpassinger - Medical University of Vienna, Anesthesiology & Intensiv Care, Vienna, Austria

**Introduction:** Postoperative pain might be ameliorated by substituting propofol for sevoflurane anesthesia. Support for this theory comes from human pain models in which propofol reduced hyperalgesia and allodynia in response to pinprick and electric stimulation.^1^ Benefit may result from central and peripheral analgesic effects of sub-hypnotic doses of propofol, as well as suppression of spinal sensitization.^2,3^ Additional analgesic effects of propofol likely result from interactions with N-methyl-d-aspartate (NMDA), non-NMDA receptors, and via activation of gamma-aminobutyric acid A (GABA_A_) receptors in the dorsal root ganglion nociceptor cells.

Consistent with multiple analgesic mechanisms, some studies report that patients anesthetized with propofol have less postoperative pain than those anesthetized with volatile anesthestics.Other studies, though, do not support a postoperative analgesic effect of intraoperative propofol.

**Objectives:** Because it remains unclear whether intraoperative propofol analgesia ameliorates postoperative pain, we tested the primary hypothesis that postoperative opioid requirements are greater in patients anesthetized with sevoflurane than propofol.

**Methods:** Ninety patients having open vein stripping were randomized to either sevoflurane or propofol anesthesia. Pain was treated with bolus piritramid and patient-controlled morphine hydrochloride. The primary outcome was total opioid use from the end of surgery until the first postoperative morning. Pain scores (11-point Likert verbal response score) were recorded by a blinded investigator at 30-minute intervals for the initial 4 hours and on the first postoperative morning.

**Results:** Sevoflurane was not superior to propofol on postoperative opioid consumption, giving a ratio of means (95 % interim-adjusted CI) of 0.91 (0.33, 2.4), P = 0.74. Medians [quartiles] of morphine sulfate equivalents were 9.8 mg [4, 19] in the sevoflurane group and 10 [6, 20] mg in the propofol group. In addition, no difference on pain score over time was found between two groups, with a mean difference on an 11-point scale of 0.20 (95 % interim-adjusted CI: -0.36,0.73, P = 0.31).

**Conclusions:** Intraoperative sevoflurane did not reduce postoperative analgesia, which is consistent with most previous reports.

**References**

1. Bandschapp O., Filitz J., Ihmsen H et al. Analgesic and antihyperalgesic properties of propofol in a human pain model. Anesthesiology 2010;113:421–428

2. Jewett BA, Gibbs LM, Tarasuik A, Kendig J. Propofol and barbiturate depression of spinal nociceptive neurotransmission. Anesthesiology, 1992, Vol: 77:1148–1149

3. Cheng S., Yeh J., Flood P. Anaesthesia Matters:Patients anaesthetized with propofol have less postoperative pain than those anesthetized with isoflurane Anesthesia and Analgesia 2008, Vol:106:264–269

### A420 Comparison between fuzzy and quadratic models in the development of the EEG based consciousness index qCON

#### U. Melia^1^, J. Fontanet^1^, J.P. van den Berg^2^, M.M.R.F. Struys^2,3^, H.E.M. Vereecke^2,3^, E.W. Jensen^1,4^

##### ^1^Quantium Medical, Barcelona, Spain; ^2^University Medical Center Groningen, University of Groningen, Gronigen, Netherlands; ^3^Ghent University, Ghent, Belgium; ^4^Center for Biomedical Engineering Research, Barcelona, Spain

###### **Correspondence:** U. Melia - Quantium Medical, Barcelona, Spain

**Introduction:** In the last decades, several methods have been developed for the noninvasive assessment of the level of consciousness during general anesthesia by processing physiological signals. The performance of the developed indices depends on a large number of factors such as the choice of the signal, the selected parameters, the inter-individual variability, etc. However, it is not clear how the type of the model that is used for the index development and calculation can affect its performances.

**Objectives:** The objective of the study was to compare two electroencephalographic (EEG) derived depth of anesthesia indexes that were developed with two different models: an adaptive neuro-fuzzy inference system (qCON ANFIS) and a quadratic equation model (qCON QE). The models have the same inputs based on the energy on four EEG frequency bands.

**Methods:** After IRB approval, 71 patients scheduled for elective surgery were randomized in four groups. Anesthesia was induced using effect-site controlled, target controlled infusion with effect size concentration of propofol set to 8.6, 5.9, 3.6 or 2 μg/mL while the corresponding remifentanil was set to 1, 2, 4 and 8 ng/mL respectively. Data were used from 2.5 minutes before to 11 minutes after starting pumps. Patients enrolled were adults from both genders with age 53 ± 13 years, weight 79 ± 14 kg and height 174 ± 9 cm (mean ± standard deviation).

The EEG was recorded with qCON 2000 monitor (Quantium Medical, Barcelona, Spain) at a sampling frequency of 1024 Hz. The qCON ANFIS and qCON QE indices were calculated on one-second EEG windows.

The agreement between the two anesthesia indexes was evaluated with correlation (R^2^), prediction probability (Pk) and Bland Altman analysis by calculating the bias and the standard deviation (SD) of the differences between the two indexes. Limits of agreement were defined as the bias ± 1.96SD in which 95 % of the differences between the two indexes are expected to lie. It is considered a clinically acceptable level of agreement when two indexes would differ by less than 10 units.

**Fig. 9 (abstract A420).**
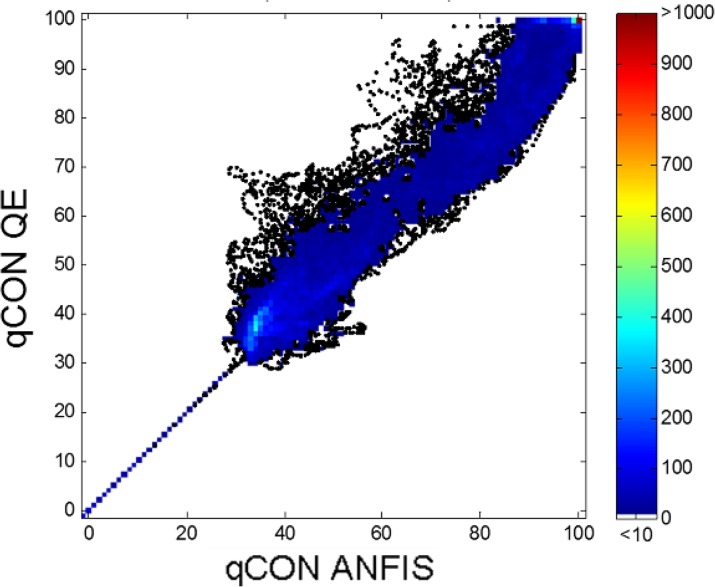
Scatter density plot of qCOn QE vs. qCO

**Fig. 10 (abstract A420).**
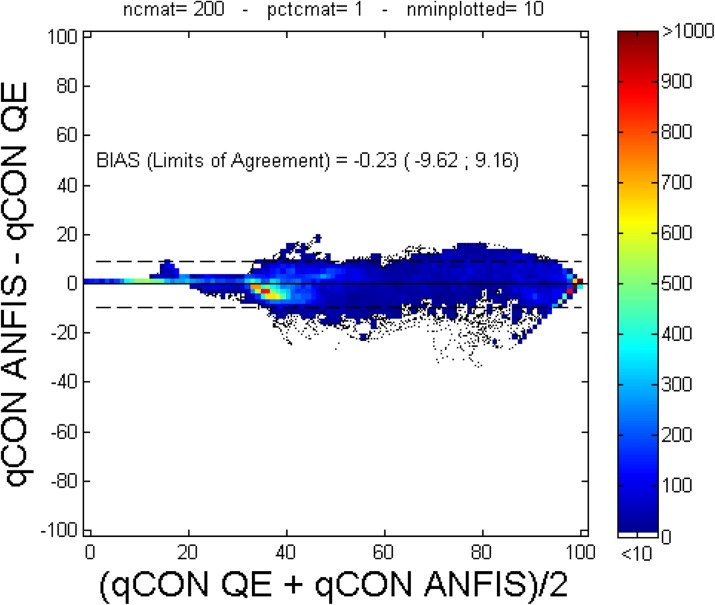
Bland Altman analysis: qCON QE vs. qCON

**Results:** Fig. 9 shows the density plot of qCON QE vs. qCON ANFIS. Table 3 shows the values of the R^2^ and Pk between qCON ANFIS and qCON QE. Figure. 10 shows the results of the Bland Altman analysis of qCON ANFIS versus qCON QE.

**Conclusions:** The two indexes qCON ANFIS and qCON QE that were developed with two different models showed a strong correlation (R^2^ > 0.95, Pk > 0.95) and a good agreement (Bias < 1 and limits of agreement < 10). In conclusions, changing the model of the qCON calculation from a fuzzy based model to a quadratic equation do not affect the index performances in depth of anesthesia assessment, with the advantage of working with a quadratic model structure that has less coefficients than the fuzzy model.

**Table 3 (abstract A420).** Values of R2, Pk (Standard Deviation SE)

Indexes	R2	Pk	SE
qCON ANFIS vs. qCONQE	0.9591	0.9637	0.0003

### A421 ICU workload in different groups of delirious patients

#### P.J.T. Rood, F. van de Schoor, K. van Tertholen, P. Pickkers, M. van den Boogaard

##### Radboud University Nijmegen Medical Centre, Intensive Care, Nijmegen, Netherlands

###### **Correspondence:** P.J.T. Rood - Radboud University Nijmegen Medical Centre, Intensive Care, Nijmegen, Netherlands

**Introduction:** Delirium occurs frequently in ICU patients and is associated with numerous adverse effects. Apart from the hyperactive, hypoactive and mixed subtype, recently the 'rapid reversible sedation-related delirium' was described which disappears after discontinuing of sedatives and is thought to have different outcomes then regular hypoactive delirium. Apart from a burden for the patient, the presence of delirium may also increase the workload for ICU professionals. Typically, workload in the ICU is measured using the Simplified Therapeutic Intervention Scoring System (TISS-28).

**Objectives:** To determine the effect of delirium status on ICU workload.

**Methods:** A retrospective cohort study was performed at the ICU of a university medical center. All ICU patients admitted between December 2011 and July 2013 were classified for their delirium sub-type status, using the CAM-ICU. Peterson criteria were used to distinguish between the delirium subtypes, added with Patel's criterion for rapid reversible sedation-related delirium. Demographics and other relevant data relating to ICU workload were collected.

**Results:** A total of 3,926 ICU patients were admitted during the study period (Table 4). The age was 62 ± 15 (mean ± SD), of which 64.6 % were male. The APACHE II score was 17 ± 7, and hospital mortality was 11.6 %. Patients were ventilated for median 2 [IQR 2–3] days. The LOS-ICU was median 1 [IQR 1–3] day, and in-hospital LOS was median 5 [IQR 5–17] days. In total 881 (22 %) patients were classified as delirious of which the alternating subtype had the highest incidence. Delirium duration was median 1 [IQR 1–2] day, the dose of haloperidol administered was median 19 [IQR 9–42] mg. The overall TISS-28 score was 28 ± 7. After adjusting for APACHE II score the TISS-28 score did not differ between delirious patients and non-delirious patients, (28.4 ± 6 versus 27.2 ± 8, respectively). In addition, no differences in TISS-28 score were found between the delirium subgroups. Patients with delirium stayed significantly longer on the mechanical ventilator, in the ICU and in-hospital (all *p <* 0*.*001).

**Conclusions:** Severity of illness but not delirium contributes in a higher workload in ICU patients. Furthermore, there were no differences in subgroups of delirium regarding workload. Importantly, however, delirium does burden ICU patients in the ICU.

**Table 4 (abstract A421).** Outcome measures stratified to delirium status

	Not delirious	Hyperactive	Hypoactive	Alternating	Rapid reversible
	N=3045	N=68	N=322	N=330	N=161
Mean TISS28	27±8	27±6	28±6	29±6	28±6
APACHE II score	17±6	19±6	21±7	21±7	17±5
Days ventilated	1 [1–2]	2 [1–3]	3 [2–10]	6 [2–14]	2 [1–2]
Delirium duration	0	1 [1–2]	1 [1–2]	1 [1–1]	1 [0–1]
Cumulative dose of haloperidol (mg)	0	9 [4–16]	17 [8–30]	24 [11–54]	1 [1–1]
ICU stay in days	1 [1–2]	2 [1–5]	5 [2–11]	7 [3–17]	1 [1–3]
Hospital stay in days	7 [4–14]	9 [5–17]	16 [9–31]	22 [12–38]	7 [6–14]

### A422 A colour coded, targeted RASS (Richmond Agitation-Sedation Scale) protocol to improve sedation practice

#### Z.J. Beardow, H. Redhead, K. Paramasivam

##### Leeds Teaching Hospitals NHS Trust, Adult Critical Care, Leeds, UK

###### **Correspondence:** Z.J. Beardow - Leeds Teaching Hospitals NHS Trust, Adult Critical Care, Leeds, UK

**Introduction:** Continuous infusions of sedative analgesia prolong the duration of mechanical ventilation and increase the risk of complications. Numerous scoring systems exist that assess depth of sedation, with current guidelines supporting the use of the RASS (Richmond Agitation-Sedation Scale) [1]. Studies highlight that caring for more awake patients is more demanding and increases workload for healthcare staff [2,3]). A preliminary audit was undertaken on our General Intensive Care Unt (GICU) in 2014–2015 to identify whether patients were optimal sedated, as determined by RASS.

**Objectives:** As a result of the previous audit three main areas for improvement were identified.

1. To achieve optimum levels of sedation as determined by RASS.

2. To achieve a gradual reduction in sedation to achieve spontaneous breathing on the ventilator at the earliest opportunity.

3. To achieve predictable and timely sedation holds.

**Methods:** A ´targeted RASS´ tool was developed, which was simple, colour coded and kept at the patient bed-side. A protocol for gathering data from audited sedation days was developed.

The protocol was instigated over a 5 week period from 4/1/2016 to 5/2/2016. All sedated patients were included, except for those having treatment withdrawn. On each patient, data was collected at the beginning and end of each shift. Including baseline and target RASS, times of RASS allocation made and sedation drug usage. Data was collated on a spreadsheet within the ICU.

**Results:** Total number of admissions were 156 patients, total number of sedated, ventilated days were 46. 38 out of the 46 patients had targets set during ward rounds (82 %) There was no difficulty allocating patients to groups.

11 patients were allocated to RED which achieved 100 % compliance.

13 patients were allocated to AMBER which achieved 63 % compliance. Reduction in sedation drugs and RASS at the end of each shift was, on average, reduced.

14 patients were allocated to GREEN which achieved a 32 % compliance. Sedation drugs were largely stopped or reduced. On average the RASS was lower. Successful extubation occurred in 5 patients. Agitation leading to re-sedation occurred in 8 patients.

**Conclusion:** A simple, clear and effective tool for communication between medical and nursing staff can improve practice and be translated into a real reduction in sedation drug administration and lower levels of sedation.

**References**

1. Barr, J et al. 2013, Clinical Practice guidelines for the management of pain, agitation and delirium in adult patients in the intensive care medicine.41.263

2. Everingham et al. 2014 ´Targeting sedation: the lived experience of the intensive care nurse. Journal of clinical nursing 23

3. Tingsvik et al. 2013 Meeting the challenge: ICU nurses´s experiences of lightly sedated patients. Australian Critical Care 26

### A423 EEG-based delirium monitoring: a prospective, multicentre validation study

#### T. Numan^1^, M. van den Boogaard^2^, A.M. Kamper^3^, P. Rood^2^, L.M. Peelen^1^, P.M. Zeman^4^, A.J. Slooter^1^

##### ^1^University Medical Center Utrecht, Intensive Care Center, Utrecht, Netherlands; ^2^Radboud University Nijmegen Medical Centre, Intensive Care Medicine, Nijmegen, Netherlands; ^3^Isala Zwolle, Geriatrics, Zwolle, Netherlands; ^4^abvsciences, Vancouver, Canada

###### **Correspondence:** A.J. Slooter ^_^ University Medical Center Utrecht, Intensive Care Center, Utrecht, Netherlands

**Introduction:** Delirium is common in Intensive Care Unit (ICU) patients and negatively affects outcome. However, recognition of delirium by ICU physicians is poor [1]. To improve detection, delirium screening tools have been developed, but these are subjective and insensitive in routine, daily practice [2]. Previously, we showed that patients with and without delirium could very well be distinguished with one minute of bipolar electroencephalography (EEG) and relative delta power as an indicator of slowing of background activity [3]. Based on these findings, a prototype EEG-based delirium monitor was built, with real-time artefact removal and an improved detection algorithm.

**Objectives:** To validate an EEG-based delirium monitor in patients at risk of delirium.

**Methods:** In this ongoing multicentre study, we will include 154 frail, surgical patients aged ≥60 years. Preoperatively (T-1) and on postoperative day 1 to 3 an EEG recording is performed together with a standardized cognitive assessment that is a video-taped. Diagnosis of delirium is based on the video by two, or in case of disagreement, three delirium experts (psychiatrists, geriatricians or neurologists) independent of each other, and independent of the EEG recording. EEGs are automatically analysed after artefact removal using waveform analyses, as well as analysis of relative delta power, and compared with the classification of the delirium experts (gold standard), to obtain sensitivity, specificity and the predictive values.

**Preliminary results:** Among 45 patients (75 % men, mean age 76 years, SD 7 years), 5 patients were diagnosed as delirious. The figure shows the development of delirium in an example patient and the findings of the waveform analysis on the consecutive days after surgery. Sensitivity and specificity of the waveform- and relative delta power analyses were promising, and final results will be presented at the ESICM meeting.

**Conclusions:** Our findings suggest that EEG-based monitoring is promising for objective and reliable detection of delirium in routine daily practice.

**References**

1. Eijk, M. M. J. van *et al.* Comparison of delirium assessment tools in a mixed intensive care unit. *Crit. Care Med.***37,** 1881–5 (2009).

2. Eijk, M. M. J. van *et al.* Routine use of the confusion assessment method for the intensive care unit: a multicenter study*. Am J Respir Crit Care Med.***184,** 340–4 (2011).

3. Van der Kooi, A. W. *et al.* Delirium detection using EEG: what and where to measure. *Chest***147,** 94–101 (2015).

**Fig. 11 (abstract A423).**
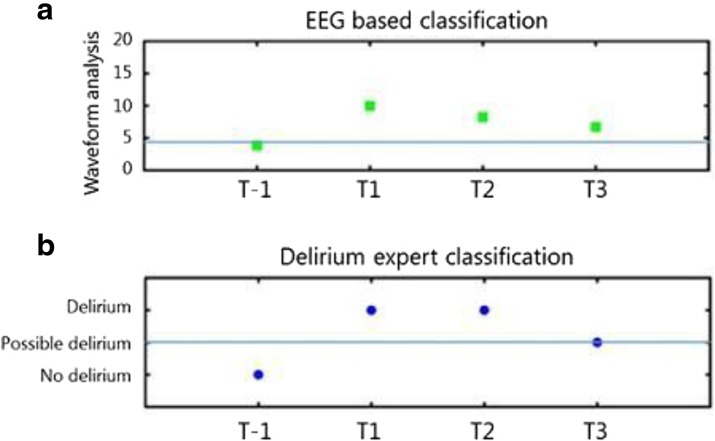
Classification by EEG and expert

### A424 Unsuspected serotonin toxicity in the ICU

#### C.E. van Ewijk^1^, G.E. Jacobs^2,3^, A.R.J. Girbes^1^

##### ^1^VU University Medical Center, Intensive Care, Amsterdam, Netherlands; ^2^VU University Medical Center, Psychiatry, Amsterdam, Netherlands; ^3^Centre for Human Drugs Research, Leiden, Netherlands

###### **Correspondence:** C.E. van Ewijk - VU University Medical Center, Intensive Care, Amsterdam, Netherlands

**Introduction:** Delirium is a frequently occurring syndrome in patients admitted to the Intensive Care Unit (ICU) or Medium Care Unit (MCU), yet the pathophysiology remains poorly understood. An excess of central serotonin can lead to an altered mental status, associated with autonomic hyperactivity, and neuromuscular excitation. (Boyer EW *et al*. 2005, Buckley NA *et al.* 2014) Drugs with serotonergic properties are frequently and for prolonged periods administered to ICU/MCU patients. Therefore central serotonergic toxicity may constitute a predisposing, contributing or precipitating factor in the emergence of delirium.

**Purpose:** to determine the number of patients admitted to the ICU or MCU who are diagnosed with delirium, and who show characteristics of serotonin toxicity (Sternbach 1991, Dunkley EJC *et al.* 2003) in association with the administration of serotonergic drugs.

**Methods:** During a 10-week prospective observational cohort study in the ICU and MCU, patients aged 18 or older, diagnosed with delirium in the ICU or MCU were included. Patients were considered as delirious in case of a positive CAM-ICU and/or at the start of haloperidol prescription on suspicion of delirium. Once included, patients were screened for recent administered serotonergic drugs and screened for physical signs associated with serotonin toxicity by a standardized physical examination by a specifically trained physician.

**Results:** A total of 61 patients diagnosed with delirium were enrolled. In 44 out of 61 patients (72,1 %) the use of drugs potentially contributing to serotonergic toxicity was recorded. Out of 44 patients, 7 (16 %) patients showed physical signs of serotonin toxicity and in addition met the Hunter serotonin toxicity criteria, suggesting the presence of serotonergic toxicity. None of these patients were recognized as such by the treating physicians.

**CONCLUSIONS:** A significant proportion of delirious patients in the ICU might in fact be classified as suffering from central serotonin toxicity. The awareness of potential serotonin toxicity is low among physicians.

**References**

1. Boyer EW, Shannon M (2005) The serotonin syndrome. N Engl J Med 352:1112–20.

2. Buckley NA et al. (2014) Serotonin syndrome. Bmj 1626(February):15–8.

3. Sternbach H (1991) The Serotonin Syndrome. Am J Psychiatry 148(June):705–13

4. Dunkley EJC et al. (2003) The hunter serotonin toxicity criteria: Simple and accurate diagnostic decision rules for serotonin toxicity. QJM - Mon J Assoc Physicians 96:635–42.

### A425 Prospective study to determine the incidence and risk factors for delirium in cancer patients in ICU

#### S.N. Myatra, M.M. Harish, N.R. Prabu, S. Siddiqui, A.P. Kulkarni, J.V. Divatia

##### Tata Memorial Hospital, Mumbai, India

###### **Correspondence:** MM M. Harish - Tata Memorial Hospital, Mumbai, India

**Introduction:** Delirium in ICU associated with both short and long term adverse outcomes. Cancer patients admitted to ICU have several risk factors to develop delirium. Most published data on delirium are about general ICU patients and there are no large studies in cancer patients in ICU.

**Objectives:** We conducted prospective study to know the incidence and risk factors associated with delirium in cancer patients.

**Methods:** Adult cancer patients in ICU were included over a period of 6 months. Demographic data, type/stage of cancer, treatment details including drugs administered were noted. Hemodynamic status, mechanical ventilation, presence of sepsis, patient's sleep and mobilization status were also noted for the assessment of risk factors associated with delirium. Sedation was scored using the Richmond Agitation-Sedation Scale (RASS) and delirium was assessed using the Confusion Assessment Method (CAM-ICU) per shift by the bedside nurses, if they were responsive to verbal commands (RASS score of −3 or lighter level of sedation). If delirium was positive even in one shift in a day, it was considered as a delirium day.

**Results:** Patients with comorbidities showed higher incidence of ICU delirium as compared to those without {73(63.5 %) vs 42(36.5 %)}. Tobacco consumers showed higher incidence of delirium as compared to those without {85(73.1 %) vs 30(26.1 %)}. Recent chemotherapy showed positive correlation with delirium as compared to those who did not receive chemotherapy or received it more than one month back {63(54.8 %) vs 51(44.3 %) vs 1(0.9 %)}. Sleep deprived patients had higher incidence of delirium as compared to patients without {90(78.3 %) vs 25(21.7 %)}. There was a significant association of Midazolam with delirium as compared to those who did not receive the drug{104(90.4 %) vs 11(9.6 %), p-0.000}. Patients who had delirium had median length of stay 7 days as compared to 3 days among without delirium. On multivariate analysis of the risk factors comorbidity(P-0.003,OR-13.3), tobacco consumption(P-0.015,OR-5.95), Midazolam(P-0.000,OR-47.50), mechanical ventilation(P-0.022,OR-9.09) and sleep deprivation (P-0.030,OR-7.18) showed statistically significant association with the delirium. Delirium also showed significant association with increased length of both ICU (P-0.000,OR-1.73) and hospital stay (P-0.004,OR-1.02).

**Conclusion:** Incidence of ICU delirium among cancer patients was 70.6 %. Recent chemotherapy mechanical ventilation, Midazolam, comorbidities, tobacco and sleep deprivation were the independent predictor of ICU delirium in cancer patients. ICU delirium increase both ICU and hospital length of stay.

**References**

1. American Psychiatric Association, Diagnostic and Statistical Manual, 4th ed, APA Press, Washington, DC 1994.

2. Ely ICM 2001;27,1892-1900, Ely JAMA 2004;291:1753–1762,

3. Lim SM, CCM 2004;32:2254–2259

### A426 Characteristics of critical patients with and without delirium submitted to mobilize early active

#### L.D. Murbach, M.A. Leite, E.F. Osaku, C.R.L.M. Costa, M. Pelenz, N.M. Neitzke, M.M. Moraes, J.L. Jaskowiak, M.M.M. Silva, R.S. Zaponi, L.R.L. Abentroth, S.M. Ogasawara, A.C. Jorge, P.A.D. Duarte

##### Western Parana State University Hospital, Cascavel, Brazil

###### **Correspondence:** L.D. Murbach - Western Parana State University Hospital, Cascavel, Brazil

**Introduction:** Delirium is defined as an acute cognitive impairment, inattention and disorganized thoughts, may be associated with the use of benzodiazepines, opioid administration, mechanical ventilation duration and immobilization, the management of it depends on multidisciplinary team strategies.

**Objective:** The purpose of this study was to compare the results of patients with and without Delirium that were submitted to an early mobilization protocol in the Intensive Care Unit (ICU).

**Methods:** Cohort study in the general ICU of a University Hospital, Southern Brazil. The study was conducted between February and December 2013. Were excluded patients using mechanical ventilation for less than 24 hours, under 18 years old, patients submitted to passive exercises or who did not have early mobilization. Only patients that received active and active-assisted early mobilization were included. Patients were divided between Delirium and no-Delirium groups. Data were reported as mean and standard deviation. The variables were compared through of Mann–Whitney test, *t*-test parametric and the statistical significance level was 5 % (p ≤ 0.05).

**Results:** In the period were admitted 474 patients of which 127 were included. The incidence of Delirium was 48 %. The main causes of admission in groups with delirium and without delirium were medical non-neurological (34 % vs. 33 %), medical neurological (22 % vs. 9 %) and Traumatic Brain Injury (22 % vs. 26 %). There were no significant. The data are demonstrated in Tables 5 and 6.

**Conclusion:** In this cohort study, we found no differences in the outcomes of patients with or without delirium submitted to early active mobilization. Most of patients had good response to an early mobilization protocol.

**Table 5 (abstract A426).** ᅟ

	Delirium (61)	Non Delirium (66)	p-value
Male (n-%)	43 (70)	43 (65)	
Age (n-%)	49±18.25	43±19.55	0.10
APACHE II	24±4.94	24 ± 6.51	0.49
SOFA	12±3.28	11 ± 3.73	0.17
Death ICU (n-%)	2 (3)	8 (13)	0.131

**Table 6 (abstract A426).** **ᅟ**

	Delirium (61)	Non Delirium (66)	p-value
MV (hours)	179±124.09	175±183.61	0.26
Sedation time (hours)	100±80.52	108±122.18	0.62
Midazolam (mg)	2532±3168.53	3258±5828.26	0.68
Fentanyl (μg)	45804±70319.75	41422±84192.46	0.25
Propofol (mg)	3289±9146.06	4570±9392.23	0.89
Haloperidol (mg)	39±38.85	54±40.33	0.15
Glasgow Coma Scale - ICU discharge	14±1.42	13±1.92	0.57
Functional independence measure	61±31.34	68±30.03	0.40

### A427 Incidence and risk factors of delirium in an oncological intensive care unit

#### N. Hernández-Sánchez, L.A. Sánchez-Hurtado, F.J. García-Guillen, S.A. Ñamendys-Silva

##### Instituto Nacional de Cancerología, Department of Critical Care Medicine, Mexico, Mexico

###### **Correspondence:** N. Hernández-Sánchez -Instituto Nacional de Cancerología, Department of Critical Care Medicine, Mexico, Mexico

**Introduction:** The presence of delirium in the intensive care unit (ICU) is often underestimated due to its variable clinical presentation. The incidence and risk factors of delirium have not been studied in critically ill cancer patients.

**Objective**: To evaluate the incidence and risk factors of delirium in critically ill cancer patients.

**Methods:** The present study was an observational and descriptive study that included 69 critically ill cancer patients admitted to ICU of the Instituto Nacional de Cancerología of México, between December 2015 and March 2016. Delirium was diagnosed by daily evaluation using the Confusion Assessment Method for the ICU (CAM-ICU). The clinical data was obtained from the patient´s medical record. No interventions were done.

**Results:** Of the 69 critically ill cancer patients included in this study, 35 (50.7 %) were men; with a median age of 49.55 years; 2 (7.2 %) had history of delirium, and 2 (2.9 %) had delirium at ICU admission. Solid tumors were the most frequent malignances 49 (71 %), and 20 (29 %) had hematological malignancies. The principal admission diagnosis to ICU was septic shock in 27 (39.1 %). During the first 24 hours in ICU, 20 (68.1 %) required mechanical ventilation, 25 (36.1 %) received midazolam as a sedative, and 47 (68.1 %) fentanyl as an analgesic.

The incidence of delirium was 15.9 %. The hyperactive type delirium was the most common. There was no difference between risk factors (sex, age, comorbidities, history of delirium, use of analgesic, sedation or corticosteroids, and severity of critical illness) in patients who presented delirium and those who did not. The most important independent risk factors for delirium were the total number of ventilated days during ICU stay (P = 0.009) and the length of stay in ICU (P < 0.001). The following variables were independent predictors of delirium: the total number of ventilated days during an ICU stay, relative risk (RR) 1.09 (95 % confidence interval (CI); 0.98 to 1.22) and the length of stay in ICU, RR: 1.173 (95 % CI; 1.04 to 1.32). The ICU mortality was 10.1 %, no patients with delirium died.

**Conclusion:** The incidence of delirium in the oncologic ICU is low, the length of stay in ICU and the total number of ventilated days during an ICU stay were the most important risk factors for its presentation; There is no relation between delirium and ICU mortality.

**References**

1. Reade M. et al. Sedation and delirium in the intensive care unit. N Engl J Med 2014; 370:444–454

2. Uchida M. et al. Prevalence, course and factors associated with delirium in ederly patients with advanced cancer: a longitudinal observational study. Jpn J Clin Oncol, 2015, 45 (10): 934–940

### A428 Comparison of opium tincture and methadone on pain and agitation control in inhalational opium addicted patients admitted to critical care unit: a pilot study

#### B. Maghsoudi^1^, M. Emami^1^, M.B. Khosravi^1^, F. Zand^1^, H.R. Tabatabaie^2^, M. Masjedi^1^, G. Sabetiyan^1^, A. Mokri^3^, Nurses of the Central and General ICUs of Shiraz Namazi Hospital

##### ^1^Shiraz University of Medical Sciences, Shiraz Anesthesiology and Critical Care Research Center, Shiraz, Islamic Republic of Iran; ^2^Department of Epidemiology, School of Public Health - Shiraz University of Medical Sciences, Shiraz, Islamic Republic of Iran; ^3^National Center for Addiction Studies, Tehran, Islamic Republic of Iran

###### **Correspondence:** B. Maghsoudi - Shiraz University of Medical Sciences, Shiraz Anesthesiology and Critical Care Research Center, Shiraz, Islamic Republic of Iran

**Introduction:** Addiction is one of the main problems in our society, Iran,about 3 % of the whole population. Many of critically ill patients needing intensive care units (ICU)admission suffer from underlying disease such as ischemic heart disease, hypertension, diabetes and so on, and are opium addicted too. Consequently, the control of pain and agitation in the intensive care unit plays an important role in early discharge and improving patient´s outcome.

**Objectives:** Comparing opium tincture and methadone on the control of pain, agitation, and ICU outcomes in opium addicted ICU admitted patients.

**Methods:** In this randomized pilot study, a total of 50 inhalant opium addicted patients aged 18–84 years, enrolled into the study and they were divided into two groups of 25, the opium tincture(T) and methadone group(M). Patients received morphine infusion according to needs before allowing enteral drugs (at most one day). After that(as day one of study), in the T group, patients received 10 cc opium tincture in dark tea(total 50 cc) through nasogastric (NGT) tube and 10 cc normal saline intravenously (IV) every 8 hours. The M group received 50 cc dark tea via NGT plus 10 mg mehadone diluting to 10 cc IV every 8 hours.Then patient´s pain and agitation were measured and compared based on (BPS) behavioral pain scale and Richmond agitation sedation scale (RASS), and delirium by CAM-ICU. If BPS scale was desirable (−0, 1), the same dose continued, but if it was ≤ −1 or ≥ 2, the doses was decreased or increased by 2.5 (cc or mg) respectively at next interval. Rescue medications were IV morphine, midazolam and haloperidol.

**Results:** Current study shows that the use of opium tincture in the pain management(by BPS) of opium addicted ICU patients had significant desirable impact and, (p-value = 0.041) compared with methadone especially after deleting confounding variables (morphine, midazolam and haloperidol) (P-value = 0.019).But considering agitation (RASS) there was no difference before (p-value = 0.684) and after the deleting confounding variables (p-value = 0.630) between the two groups. Extubation time, duration of ICU and hospital stay was not different between the two groups.

**Conclusions:** In inhalational opium addicted patients admitted to ICU enable of starting oral medications, pain is better controlled with Opium Tincture in comparison to Methadone.

**References**

1. Comparison of **tincture** of **opium** and methadone to control opioid withdrawal in a Thai treatment centre.Jittiwutikarn J, Ali R, White JM, Bochner F, Somogyi AA, Foster DJ.Br J Clin Pharmacol. 2004 Nov;58(5):536–41

2. Flexible dosing of **tincture** of **opium** in the management of opioid withdrawal: pharmacokinetics and pharmacodynamics.Somogyi AA, Larsen M, Abadi RM, Jittiwutikarn J, Ali R, White JM. Br J Clin Pharmacol. 2008 Nov;66(5):640–7

**Grant acknowledgement**

We acknowledge Shiraz university of medical sciences and Iranian center for addiction Studies for their support.

### A429 Sevoflurane sedation in critically ill patients under extracorporeal membrane oxygenation

#### J. Troubleyn, M. Diltoer, R. Jacobs, D.N. Nguyen, E. De Waele, J. De Regt, P.M. Honoré, V. Van Gorp, H.D. Spapen

##### University Hospital, Vrije Universiteit Brussel, Brussels, Belgium

###### **Correspondence:** J. Troubleyn - University Hospital, Vrije Universiteit Brussel, Brussels, Belgium

**Introduction:** Patients with severe ARDS or intractable cardiogenic shock are increasingly treated with veno-venous or arterio-venous extracorporeal membrane oxygenation (ECMO). During ECMO, intravenous (IV) sedation with midazolam (MIDA) and/or propofol (PROP) may be inadequate because these drugs become highly sequestrated in the circuit. The volatile anaesthetic sevoflurane (SEVO) has been shown to be a potential alternative to IV sedation in cardiac surgery and general ICU patients.

**Objectives:** We assessed the efficacy and safety of prolonged use of SEVO in a cohort of hemodynamically unstable critically ill patients under ECMO.

**Methods:** Patients initially sedated with IV MIDA and/or PROP and then switched to SEVO, were retrospectively reviewed. The liquid form of SEVO was infused via a modified heat-moisture exchanger (AnaConDa®, Sedana Medical AB, Uppsala, Sweden) placed between the Y-piece and the endotracheal tube. SEVO was given as an initial bolus of 1.2 mL followed by a continuous infusion started at 5 mL/h. Infusion rate was adjusted to reach a measured end-tidal SEVO (ETS) concentration between 0.8 and 1.4 %. Sedation level aimed to obtain a RASS score of −3 to −4. Mean blood pressure and norepinephrine requirement were evaluated before start of SEVO and after respectively 2 h and 6 h of treatment. ETS was measured after stabilization, and at 24 h and 72 h. Wilcoxon signed rank test was used to compare variables over time. Values were expressed as means ± SD.

**Results:** Twenty-one patients (12 males, age 53 ± 15 years; 16 ARDS and 5 cardiogenic shock) were studied. Mean APACHE II score was 25 ± 5. MIDA and PROP dose before start of SEVO were respectively 2.1 ± 2.6 mg/min and 2.4 ± 0.9 μg/kg/min. Ten patients were curarized. Initiation of SEVO allowed immediate cessation of IV sedation and curarization. Opioid analgesia was continued unchanged. SEVO treatment was not associated with changes in MAP (73 ± 12 vs. 72 ± 10 mmHg; p = 0.56) or norepinephrine need (0.11 ± 0.06 vs. 0.13 ± 0.19; p = 0.83). As compared with baseline (1.1 ± 0.3 %), ETS remained unchanged after 24 h (1.2 ± 0.3 %) and 72 h (1.3 ± 0.4 %). Patients were treated for 13 ± 9 days. Twelve patients (57 %) survived. No adverse events were noted. Random 8 h-samples of ambient air in the bedside breathing zone revealed SEVO concentrations well below the National Institute for Occupational Safety and Health 2 ppm limit.

**Conclusions:** In hemodynamically unstable patients under ECMO, prolonged SEVO sedation is an effective, well-tolerated, and safe alternative for "classic" IV MIDA and PROP-based sedation.

### A430 A pilot study using bispectral index (BIS) to adjust sedation in patients with no neurological pathology in elderly critical care patients

#### R.S. Contreras, N.D. Toapanta, G. Moreno, J. Sabater, H. Torrado, M. Gonzalez, M. Marin, E. Farigola, A. Gonzalez, J. Fernandez, A. Vera, X. Gisbert, C. Juliá, J. Uya, L. Corral, Sedation an Delirium Group Hospital Universitari de Bellvitge

##### Hospital Universitari de Bellvitge, Intensive Care Medicine, Barcelona, Spain

###### **Correspondence:** R.S. Contreras - Hospital Universitari de Bellvitge, Intensive Care Medicine, Barcelona, Spain

**Introduction:** Patients admitted to the intensive care unit (ICU) usually require use of hypnotics and sedatives to ensure comfort and proper adaptation to mechanical ventilation. An important requirement for an adequate sedation is frequent and proper assessment of its depth. Inadequate sedation can lead to problems of over-sedation, under-sedation and/or delirium in ICU, especially in elderly patients.

**Objective:** To compare the total dose of sedative use and the rate of over-sedation in patients over 65 years admitted to the ICU, adjusting sedation by monitoring with BIS® versus monitoring with the exclusive use of sedation scales.

**Methods:** A randomized, clinical trial including patients over 65 years who were admitted to the ICU affected with medical or surgical pathology of non neurological etiology who required sedation for more than 24 hours to maintain adaptation to mechanical ventilation. Patients were randomized into two groups: the intervention group using BIS monitoring to adjust sedation in order to maintain values between 50–60 and; the control group in which sedation was adjusted with the exclusive use of Richmond Agitation-Sedation Scale (RASS) to maintain RASS −2. The study was approved by the institution's Research Ethics Committee. Statistical analysis: We used the chi square test to compare categorical data and proportions, and the *T*-test, Mann–Whitney test or Kruskal-Wallis, as appropriate, to compare continuous variables. An alpha level of 0.05 was used to determine statistical significance. All data in the present study was analyzed using SPSS version 22.0 (SPSS Inc, Chicago, USA).

**Results:** Until now 56 patients have been randomized. 27 (48 %) patients in the control group and 29 (52 %) in the intervention group. Related to the general characteristics, demographics (age, weight, height, gender), medical history (hypertension, diabetes, cognitive impairment, lung disease and chronic renal failure), pathology and admission severity scores (APACHE II and SOFA) there were not statistically significant differences between groups. Besides analgesia; midazolam, propofol or remifentanil were also used in both groups (without significant differences). There were not statistically significant differences in midazolam and propofol, total daily dose, days of mechanical ventilation, ventilator associated pneumonia, delirium, ICU mortality and ICU length of stay.

**Conclusions:** No statistically significant differences were observed between the groups of this pilot study using BIS to adjust the sedation. We attribute these findings mainly to the lack of adherence to the protocol.

**References**

1. *Sedation and analgesia in the critically ill adult*. Fraser GL, Riker RR - Curr Opin Anaesthesiol - April 1, 2007; 20 (2); 119–23 2.

**Acknowledgment**

We acknowledge ICU physicians, nurses and personnel for their contribution and especially to Antonio Diaz-Prieto for his contribution to this study designing the database program.

### A431 An audit of sedation use and sedation scores in intensive care

#### I. Elias-Jones, L. Gemmell, A. MacKay

##### Queen Elizabeth University hospital, Anaesthetics and Intensive Care, Glasgow, UK

###### **Correspondence:** I. Elias-Jones - Queen Elizabeth University hospital, Anaesthetics and Intensive Care, Glasgow, UK

**Introduction:** How much sedation is given, and for how long, is important in determining patient outcome as both over sedation and under sedation can have potentially deleterious consequences. Over sedation can increase time on ventilator support and prolong ICU duration of stay. Under sedation can cause hyper-catabolism, immunosupporession, hypercoagulability and increase sympathetic activity.

**Objectives:** The aim of this audit was to look at our use of sedation in a large 18bed teaching unit in Glasgow. We aimed to look at propofol dosage, to particularly look if patients exceeded their maximum hourly dose, sedation scoring tools and whether it was regularly assessed, the addition of other sedative agents and when, and whether sedation load was associated with increased ventilator days.

**Methods:** We looked at a snapshot of 40patients picked at random admitted to the Queen Elizabeth University hospital, Glasgow over the previous six months. The Richmond-Agitation Sedation scale was used as the sedation scoring tool.

**Results:** Data was collected for 40patients. 57.5 % were male. The median age was 57 years. Weight and height were not documented for 21patients. 6 patients were given in excess of the recommended dose of propofol according to weight (mg/kg/hr). These were six of the patients in which weight was actually documented so exceeding maximum recommended daily dosing could have been more of an issue than is described. All patients were sedated with propofol and an opiate, either remifentanil or alfentanil. Half of the patients were found to be over sedated in the time period, with RASS scores of −4 or −5. Six out of the forty patients did not have sedation scores documented daily as part of their care. When other agents were considered, they were considered relatively late in the patient stay (days 4–5). CK and triglycerides were never checked when propofol had been used in high doses for a significant length of time. Average length of ventilation was 4 days (12 hours - 12 days) with unsurprisingly higher propofol use seen in the patients ventilated for longer periods of time. Excess sedation was more common in the younger patient with a past history of drug or alcohol dependence.

**Conclusions:** Delirium and over sedation is associated with an increase in morbidity and mortality. As can be seen from our snapshot of data, we are poor at documenting daily sedation scores, with a significant proportion of our patients being documented as over sedated. Propofol use is excessive, and could perhaps lead to iatrogenic complications and morbidity. Patients who were heavily sedated were ventilated for longer.

We are now working on a sedation protocol within our unit to improve our practice.

**References**

1. K.Rowe. Sedation in the Intensive Care Unit. CEACCP 2008:8(2): 50–55

## Perioperative intensive care and fluid management

### A432 Peri operative use of plasmalyte in kidney transplant recipients reduces the incidence of RRT in the immediate post-operative period

#### D. Randall^1^, A. Adwaney^1^, M. Blunden^1^, J.R. Prowle^1,2^, C.J. Kirwan^1,2^

##### ^1^Barts Health NHS Trust, Renal and Transplant Medicine, London, UK; ^2^Barts Health NHS Trust, Adult Critical Care, London, UK

###### **Correspondence:** C.J. Kirwan - Barts Health NHS Trust, Renal and Transplant Medicine, London, UK

**Introduction:** There is evidence that the type of intravenous fluid used in patients who are critically ill or who have undergone major surgery may have an impact on the progression and recovery of Acute Kidney Injury. Patients undergoing renal transplantation are not specifically included in this work. Our centre changed the general peri-operative fluid regimen from routine use of normal saline (NS) to Plasmalyte (balanced solution containing physiological sodium, chloride, potassium, magnesium with an acetate buffer), including all patients undergoing renal transplantation. We hypothesised that this change would reduce peri-operative hyperkalaemia and thus reduce the need for immediate renal replacement therapy (RRT).

**Methods:** The peri-operative fluid management guidance was changed in March 2013. We performed a single centre retrospective observational study of consecutive renal transplant recipient's pre and post policy change to assess its impact on transplant patients. The local renal database, patient notes, a computer based pathology system and clinic letters were used to collect data. We excluded patients with incomplete data of the peri-operative period, who had not received the standard fluid regimen of the time and those with an immediate surgical complication that rendered dialysis inevitable.

**Fig. 12 (abstract A432).**
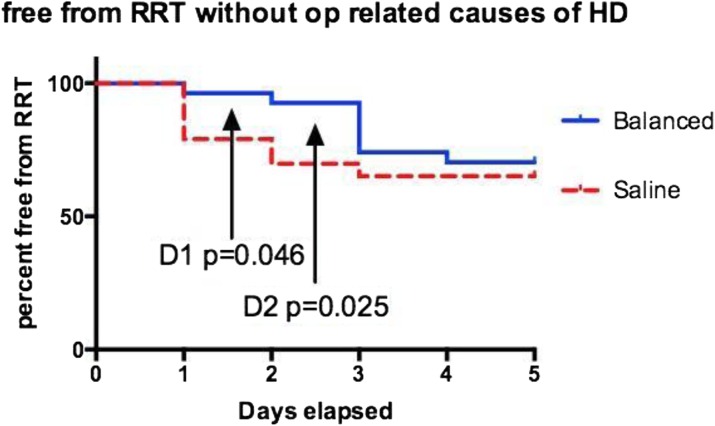
ᅟ

**Fig. 13 (abstract A432).**
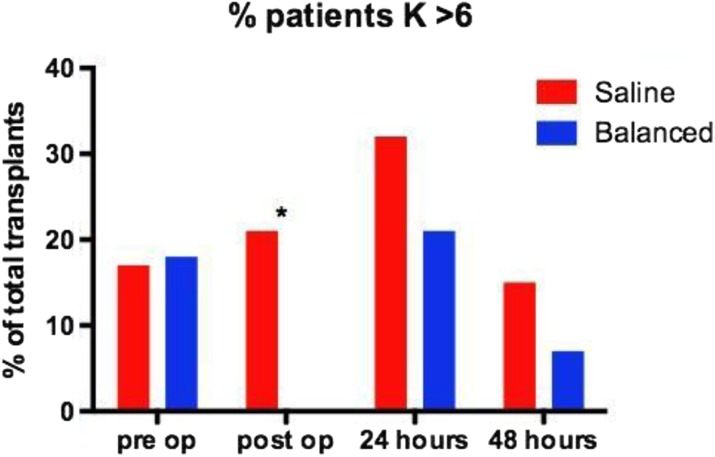
ᅟ

**Results:** There were 47 transplant recipients in the NS group and 29 in the Plasmalyte group. The percentage of patients not requiring RRT in the first 48 hours post operatively in the Plasmalyte group were significantly higher than in the NS group (D1 97 vs 81 % p 0.046, D2 94 vs. 72 %, p 0.025) (Fig. 12). There were no cases of hyperkalaemia in the immediate post operative period for patients who had received Plasmalyte (P < 0.0001) (Fig. 13).

A greater urine output in the first 24 hours after surgery was observed in the plasmalyte group, 2195mls (95 % CI:1423–2966) vs. 913.8mls (95 % CI:563.3-1264, P < 0.0001) which may indicate earlier onset of recovery from the post transplant AKI. Baseline characteristics were similar between each group except there were a higher proportion of DCD donors in the NS group compared to the Plasmalyte group (36 % vs. 17 %, p < 0.001).

**Conclusions:** Renal transplant recipients who did not have an immediate surgical complication and received only Plasmalyte in the peri-operative period, had a lower incidence of RRT in the first 48 hours following surgery than those who received normal saline. This may be attributed to the lower incidence of immediate hyperkalaemia in the Plasmalyte group.

### A433 Enhanced recovery after surgery plus reduces pulmonary morbidity after major surgery - 1 year on

#### N. Thomas^1^, A. Martin^2^, H. Owen^2^, L. Darwin^2^, D. Conway^1^, D. Atkinson^1^, M. Sharman^1^, J. Moore^1^

##### ^1^Central Manchester Foundation NHS Trust, Manchester, UK; ^2^Health Education North West, Manchester, UK

###### **Correspondence:** N. Thomas - Central Manchester Foundation NHS Trust, Manchester, UK

**Introduction:** Pulmonary complications after major surgery are common affecting up to 30 % of patients, increasing hospital length of stay, short and long-term mortality^1^. Despite this, very little specific action is taken to prevent post-operative pulmonary complications (PPC) within major surgery programmes. Even the Enhanced recovery after surgery [ERAS] programme, does not specifically address this.

**Objectives:** Central Manchester Foundation Trust (CMFT) successfully implemented ERAS+ aimed at reducing PPC in 2014 and we now have results to show its sustained benefit.

**Methods:** Using quality improvement methodology, a multidisciplinary team developed an innovative surgical pathway combined with elements of ERAS. Working with the Boston Medical Centre, we produced ICOUGH UK^2^ consisting of - **I**ncentive spirometry, **C**oughing and deep breathing, **O**ral care, **U**nderstanding (patient and family education), **G**etting out of bed and **H**ead-of-bed elevation. Additional innovations within the ERAS+ include: Prehabilitation/Reablement tools; SURGERY SCHOOL - patient and family preparation session and ICOUGH UK multimedia resources including ICOUGH TV. Pathway innovation occurred during sponsored listening sessions with patients and relatives. We introduced ICOUGH prescriptions attached to drug charts to promote compliance and this was audited weekly. All elective surgical patients requiring critical care admission were screened for the development of PPC on days 1, 3, 5, 7 and 15 after surgery using standard definitions^3^. We prospectively measured baseline PPC between April 2013–4 and repeated PPC audit following introduction of ERAS+ in September 2014 through to Jan 2016.

**Results:** ERAS+ has been successfully developed and embedded in a large NHS university teaching hospital. More than 200 critical care nurses have been trained in ERAS+. Surgery school has been embedded, with over 350 patients attending; qualitative feedback has been overwhelmingly positive. Since September 2014, utilising ERAS+ within critical care in greater than 500 patients has resulted in a sustained reduction of 40 % in PPCs.

**Conclusions:** ERAS+ implementation into a large teaching hospital has successfully reduced PPCs in major surgery patients. ERAS+ can be rapidly adopted by European healthcare, with a significant reduction in patient morbidity and hospital length of stay. This innovation aligns with domains 1, 3–5 of the NHS Outcome Framework^4^.

**References**

Moonesinghe et al. 'Survival after postoperative morbidity: a longitudinal observational cohort study' BJA 2014 113; 6: 977–84

1. ICOUGH-http://www.cmft.nhs.uk/information-for-patients-visitors-and-carers/enhanced-recovery-programme/icoughuk

2. Kroenke et al. Operative risk in patients with severe obstructive pulmonary disease. *Arch Intern Med*. 1992;152:967–971

3. NHS Outcome Framework - https://www.gov.uk/government/publications/nhs-outcomes-framework-2015-to-2016

**Fig. 14 (abstract A433).**
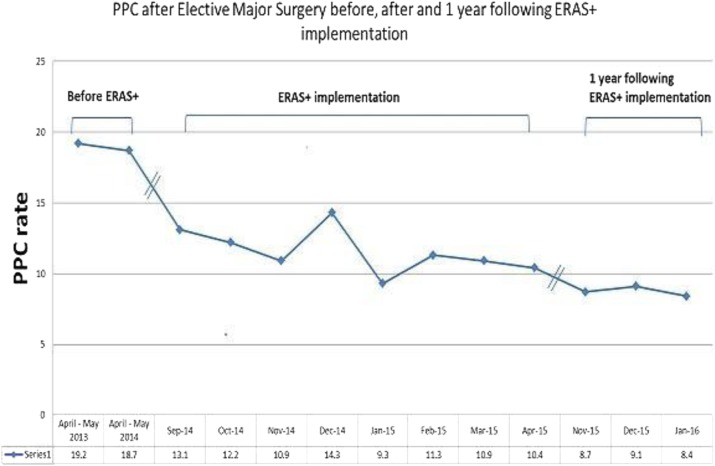
Incidence of PPC in major elective surgery patient

### A434 The use of inhaled nitric oxide (INO) in neonatal, pediatric and adult intensive care units, a franco belgian multicenter prospective survey from the positive study group

#### C. Barbanti^1^, J. Amour^2^, P. Gaudard^3^, B. Rozec^4^, P. Mauriat^5^, M. M'rini^6^, P.L. Leger^7^, G. Cambonie^8^, J.M. Liet^9^, C. Girard^10^, S. Laroche^11^, P. Damas^12^, Z. Assaf^13^, G. Loron^14^, L. Lecourt^15^, P. Pouard^16^

##### ^1^Hopital Universitaire Necker Enfants Malades, Pediatric Cardiac Intensice Care Unit, Paris, France; ^2^Pitie-Salpetriere Hospital and Pierre and Marie Curie University, Reanimation Chirurgicale Cardio Vasculaire et Thoracique, Paris, France; ^3^Arnaud de Villeneuve, Anesthésie Réanimation, Montpellier, France; ^4^Hopital Laennec CHU de Nantes, Anesthesie Réanimation, Nantes, France; ^5^Maison du Haut leveque CHU Bordeaux, Congenital Cardiac Surgery Unit, Bordeaux, France; ^6^Clinic Pasteur, Service Anesthesie Réanimation, Toulouse, France; ^7^Hopital Trousseau, Réanimation Néonatale et Polyvalente, Paris, France; ^8^Hopital Arnaud de Villeneuve, Réanimation Pédiatrique et Neonatale, Montpellier, France; ^9^CHU de Nantes, Pédiatric Intensive Care Unit, Nantes, France; ^10^CHU Bocage, Réanimation Cardio Vasculaire et Polyvalente, Dijon, France; ^11^Air Liquide Santé International, Reserach Center Paris Saclay, Paris, France; ^12^Hopital Start-Tilman, Services de Soins Intensifs Généraux, Liege, Belgium, ^13^Hopital Necker, Réanimation Néonatale, Paris, France; ^14^American Memorial Hospital, Réanilmation Néonatale et Polyvalente, Reims, France; ^15^Air Liquide Santé International, Gentilly, France; ^16^Hopital Necker Enfants Malades, Pediatric Cardiac Intensive Care, Paris, France

###### **Correspondence:** C. Barbanti - Hopital Universitaire Necker Enfants Malades, Pediatric Cardiac Intensice Care Unit, Paris, France

**Introduction:** Inhaled NO is a well-known selective pulmonary vasodilator in Europe since 20 years nevertheless few study really described the daily ICU practice. The objective of this study was to determine the gap between guidelines and real life.

**Methods:** This is a multicenter, prospective, observational study on iNO administered through an integrated delivery and monitoring device (EZ-Kinox) in 12 French and 1 Belgian centers for pulmonary arterial hypertension after cardiac surgery (PAH CS) and for persistent pulmonary hypertension of the newborn (PPHN). The following parameters were observed: dose, treatment duration, ventilation modes, monitoring procedures, weaning procedures and occurrence of a rebound effect. Concomitant pulmonary vasodilators treatments and safety data were also collected.

**Results:** 236 patients were studied among 238 patients enrolled within one year period : 81 children and 117 adults with PAHCC and 38 neonates with PPHN. Echocardiography or pulmonary artery catheterization were performed to diagnose PAH respectively in 86 % and 31 % of the cardiac patients. In the neonatal group 97.4 % had an echocardiographic diagnosis. Inhaled NO was initiated before ICU admission in adult population (57 %) but rarely in cardiac pediatric patients (12.7 %), p < 0.01 and 38.9 % in neonates. The median initial dosage of iNO set was 20 ppm [18–20] in the cardiac pediatric group and 10 ppm [10–15] for adults and 16.7 ppm [11.2-20] for neonates with a median duration respectively 3.9 days [1.9-6.1], 3.8 days [1.8-6.8] and 3.07 days [1.04-5.74]. The NO therapy classically was delivered during controlled ventilation mode including high frequency oscillation and more and more via non invasive ventilation as high flow nasal cannula. The clinical effect of iNO was considered sufficient in 89 % of the cases and the dose was gradually decreased before definitive withdrawal in 86 % of the cases. Adverse effects (AE) occurred 75 times (80 % of the patients without AE) including rebound effect in 1.2 % of children, 3.4 % of adults and 2.6 % of neonates, methaemoglobinemia was observed on 7.9 % of neonates and 1.7 % of adults. The NO_2_ generated by contact between NO and O2 was above 0.5 ppm value in 17 % of the pediatric cases, 1 % of the adult cases and never observed on neonates population. All these AE related to iNO recovered without sequelae. Pulmonary vasodilators (levosimendan, sildenafil, milrinone) were associated in 95 % of the cases in pediatrics, 23 % in adults (p < 0.01) and 23.7 % in neonates. ICU stay was 8 days [6–15] for children, 10 days [6–16] for adults and 3.7 days [1–5.7] for neonates.

**Discussion.** This survey confirms the good efficacy and safety of NO therapy in the three populations. Occurrence of rebound effect is particularly rare with a large application of a gradually withdrawal of iNO and pulmonary vasodilators use. The usage of last generation of NO devices subject to prior training allows good compliance with recommendations.

### A435 Peri-operative use of plasmalyte in kidney transplant recipients improves biochemical profile and may result in faster renal recovery

#### D. Randall^1^, A. Adwaney^1^, M. Blunden^1^, J.R. Prowle^1,2^, C.J. Kirwan^1,2^

##### ^1^Barts Health NHS Trust, Renal and Transplant Medicine, London, UK; ^2^Barts Health NHS Trust, Adult Critical Care, London, UK

###### **Correspondence:** C.J. Kirwan - Barts Health NHS Trust, Renal and Transplant Medicine, London, UK

**Introduction:** Administration of normal saline has been associated with the development and exacerbation of acute kidney injury, [1] and this may be related to hyperchloraemia. Since recipients of kidney transplants often require large volumes of fluid replacement, we hypothesised improved post-operative biochemistry and quicker renal recovery following a switch from saline to Plasmalyte fluid therapy during the routine peri-operative period.

**Methods:** The peri-operative fluid management guidance was changed in our centre in March 2013. We performed a single centre retrospective observational study of consecutive renal transplant recipients before and after the policy change to assess its impact on transplant patients. We used the local renal and pathology databases, paper and computerised patient notes and clinic letters to collect data. We excluded patients with incomplete data of the peri-operative period or who had not received the standard fluid regimen of the time.

**Fig. 15 (abstract A435).**
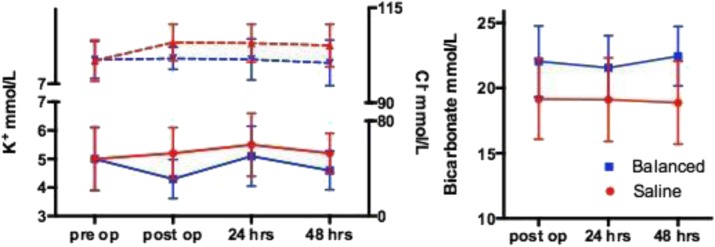
ᅟ

**Results:** 47 patients received normal saline and 29 received plasmalyte. Demographics were similar between groups, except the saline group contained more patients receiving a kidney donated after cardiac death (36 % vs 17 %, p < 0.001). In both groups, fluid administration rates were matched to urine output and overall fluid balance was equal between the two groups. Electrolyte data for the two groups are shown in Fig. 15 and demonstrate significantly higher potassium and chloride and lower bicarbonate levels in the normal saline group (P < 0.0001).

**Fig. 16 (abstract A435).**
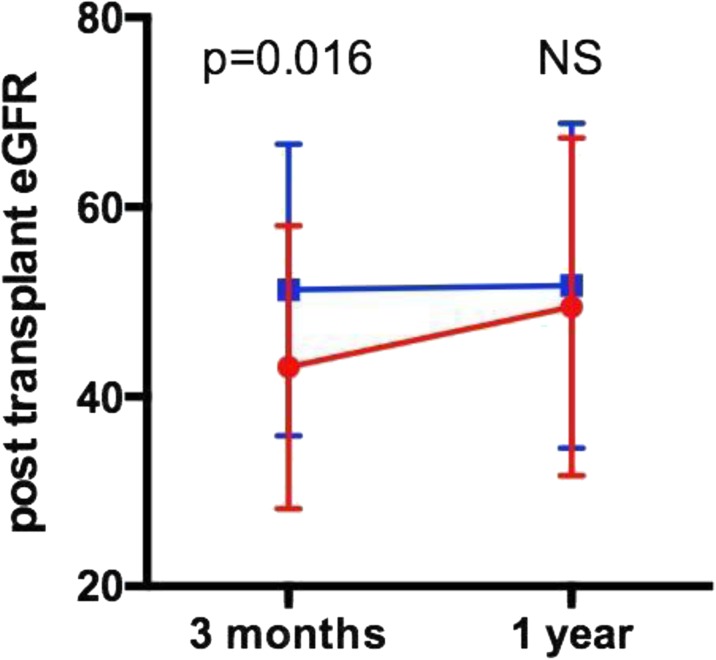
ᅟ

There was a higher proportion of hyperkalaemia (serum K^+^ >6.0 mmol/L) immediately post-op (21 % vs 0 %), over the first 24 hours (32 % vs 21 %) and over the second 24 hours post-operatively (15 % vs 7 %) in the patients who received normal saline (P < 0.001). The saline group had a longer length of stay (11 days vs 7 days, p < 0.0001), and lower eGFR at 3 months post-op (43 vs 51 ml/min; P = 0.016), although by 1-year post-transplant, mean renal function was identical at 51 ml/min in both groups (Fig. 16).

**Conclusions:** The sole use of Plasmalyte fluid replacement in the peri-operative period is associated with improved potassium, chloride and acid base homeostasis after kidney transplantation when compared to normal saline. The use of Plasmalyte is safe (despite the concern that it contains potassium), and may be associated with improved early renal outcomes. Whilst evidence of benefit for balanced replacement fluids in the general population is conflicting, after transplantation the effects may be more pronounced as the volumes of fluid administered are greater.

**References**

[1] Yunos NM et al. JAMA. 2012 Oct 17;308(15):1566–72

### A436 Albumin, type of surgery, and apache II score predict return home after surgery in critically ill patients aged more than 85 years running title: predictors for home return of patients aged >85 years

#### S.H. Kim, S. Na, J. Kim

##### Yonsei University College of Medicine, Anesthesia and Pain Medicine, Seoul, Republic of Korea

###### **Correspondence:** S.H. Kim - Yonsei University College of Medicine, Anesthesia and Pain Medicine, Seoul, Republic of Korea

**Introduction:** Owing to an aging society, both the number of operations for patients aged >85 years and the average age of patients admitted to the intensive care unit (ICU) are rapidly increasing. However, mortality is not an appropriate outcome measurement in patients aged >85 years; a more important outcome is home return (HR), because the quality of life is valuable to these patients.

**Objectives:** To identify predictors for HR of patients aged >85 years admitted to the ICU after surgery.

Study design and settings: Retrospective analysis of medical records at a university hospital.

**Subjects:** Patients aged >85 years admitted to the ICU after surgery (n = 187).

**Methods:** Patients were divided into a HR group (patients who returned home after discharge) and non-HR group (deceased or transferred to nursing facilities). Perioperative data and outcome were assessed and compared. Multivariate logistic regression analysis was conducted to identify independent predictors.

**Results:** The average age of patients was 88 years. HR occurred in 61 % of patients, and mortality was 9 %. The HR group showed higher preoperative albumin level than the non-HR group. More patients in the non-HR group experienced hip surgery than in the HR group (51 % vs. 12 %, P < 0.001). APACHE II score was higher (P < 0.001) in the non-HR group. In multivariate analysis, preoperative albumin, hip surgery, and APACHE II score were identified as independent predictors of HR.

**Conclusion:** Predictors of HR of surgical critically ill elderly patients included preoperative albumin level, hip surgery, and APACHE II score on ICU admission.

**References**

1. Hennessy D, Juzwishin K, Yergens D, Noseworthy T, Doig C. Outcomes of elderly survivors of intensive care: a review of the literature. Chest 2005; 127: 1764–74.

2. Nguyen YL, Angus DC, Boumendil A, Guidet B. The challenge of admitting the very elderly to intensive care. Ann Intensive Care 2011; 1: 29.

3. Bagshaw SM, Webb SA, Delaney A et al. Very old patients admitted to intensive care in Australia and New Zealand: a multi-centre cohort analysis. Crit Care 2009; 13: R45.

### A437 Postoperative serum chloride levels and acute kidney injury in the ICU after liver transplantation

#### S.-Y. Oh^1^, C.W. Jung^2^, S.-H. Yoo^2^, S.-H. Min^2^, E.-J. Chung^2^, H. Lee^2^, N.J. Lee^1^, K.W. Lee^1^, K.-S. Suh^1^, H.G. Ryu^2^

##### ^1^Seoul National University College of Medicine, Department of Surgery, Seoul, Republic of Korea; ^2^Seoul National University College of Medicine, Department of Anesthesiology, Seoul, Republic of Korea

###### **Correspondence:** S.-Y. Oh - Seoul National University College of Medicine, Department of Surgery, Seoul, Republic of Korea

**Introduction:** Immediate postoperative liver transplant recipients are at risk for developing acute kidney injury (AKI) with a reported incidence between 17 to 95 %. Recent findings suggest that the chloride-rich resuscitation fluids may increase the incidence of acute kidney injury in critically ill and surgical patients. Hyperchloremic fluids have been shown to induce renal vasoconstriction, attenuate renal artery flow velocity, and reduce renal cortical tissue perfusion.

**Objectives:** The association between postoperative serum chloride levels and the incidence of immediate postoperative acute kidney injury in the ICU in liver transplant recipients were evaluated.

**Methods:** Adult patients (≥18 years old) who underwent liver transplantation at Seoul National University Hospital between July 2010 and December 2012. Preoperative serum chloride levels were measured within 24 hours before the operation and postoperative serum chloride levels were routinely measured every 8 hours during the initial 72 hours after ICU admission (10 measurements). The average serum chloride level was defined as the average of the 10 serum chloride levels in patients who did not develop AKI or developed AKI 72 hours after liver transplantation and the average of the preoperative serum chloride level and the serum chloride levels measured before the development of AKI in those who developed AKI within 72 hours. Patients were divided into 3 groups according to their average chloride level.; normochloremia group (96–106 mEq/L), hypochloremia group (<96 mEq/L), or hyperchloremia group (>106 mEq/L). The primary outcome was postoperative AKI according to the RIFLE criteria. Secondary outcomes included in-hospital mortality and surgical site infection.

**Results:** AKI developed in 46.6 % (158/339) of the patients. The overall in-hospital mortality was 4.7 %. Compared to normochloremia, AKI was more frequent in patients with hyperchloremia (adjusted OR 1.826 [95 % CI 1.133-2.944], P = 0.013) and hypochloremia (adjusted OR 8.794 [95 % CI 1.820-42.498], P = 0.007). There was no difference in preoperative chloride levels between patients who developed AKI and those who did not (104.54 ± 8.41 vs 103.18 ± 6.14 mEq/L, P = 0.089).

**Conclusions:** Hyperchloremia and hypochloremia were both associated with an increased risk of developing AKI in the immediate postoperative period after liver transplantation in the ICU.

**References**

1. Hoste EA, Clermont G, Kersten A, et al. RIFLE criteria for acute kidney injury are associated with hospital mortality in critically ill patients: a cohort analysis. *Crit Care* 2006; **10**: R73

2. Barri YM, Sanchez EQ, Jennings LW, et al. Acute kidney injury following liver transplantation: definition and outcome. *Liver transplantation : official publication of the American Association for the Study of Liver Diseases and the International Liver Transplantation Society* 2009; **15**: 475–83

### A438 The association between serum sodium fluctuations and mortality in surgical patients requiring intensive care

#### D.C. Marshall, R.J. Goodson, J.D. Salciccioli, J. Shalhoub

##### Imperial College London, London, UK

###### **Correspondence:** J.D. Salciccioli - Imperial College London, London, UK

**Introduction:** Electrolyte abnormalities are common in critically ill populations and are associated with mortality. Previous reports have demonstrated a relationship between serum sodium fluctuations and mortality in a cohort of surgical critically ill patients (1–3).

**Objectives:** Our primary aim was to assess the association between serum sodium fluctuations and 28-day mortality in a cohort of adult surgical patients requiring intensive care. Our secondary aim was to assess the association between dysnatraemia and 28-day mortality in surgical patients requiring intensive care.

**Methods:** We performed a retrospective analysis of the Multi-Parameter Intelligent Monitoring in Intensive Care (MIMIC-II) database. Subjects were categorized by severity of dysnatraemia and sodium fluctuations during the index ICU admission. Dysnatraemia was defined as a sodium concentration upon ICU admission outside physiologic range (135–145 mmol/L). Univariate and multivariable logistic regression was used to test primary and secondary aims.

**Results:** We identified 8600 subjects, 39 % female with a median age of 66 years for analysis; 28-day mortality was 8 %. Fluctuations in serum sodium were found to be associated with 28-day mortality (adjusted odds ratio (OR) per 1 mmol/L change: 1.10 (95%CI 1.08-1.13)) in dysnatraemia. In subjects who remained normotraemic, there was an association between fluctuation in serum sodium and 28-day mortality (adjusted OR 1.13, 95%CI 1.10 - 1.16; p < 0.001). Subjects with dysnatraemia were more likely to be dead at 28-days (17 % vs 7 %; p < 0.001). Severity of dysnatraemia was associated with 28-day mortality (adjusted ORs (95 % CI): severe hyponatraemia (<130 mmol/L) 2.68 (1.72-4.17); mild hyponatraemia (130–134 mmol/L) 1.59 (1.24-2.05); mild hypernatraemia (146–150 mmol/L) 2.46 (1.76-3.43); severe hypernatraemia (>150 mmol/L) 4.73 (2.54-8.80)).

**Conclusions:** Fluctuations of serum sodium, including in patients whose sodium remained within the normal range, were independently associated with an increase in 28-day mortality. Severity of dysnatraemia was associated with 28-day mortality. These findings are consistent with previous reports and suggest that minor fluctuations in sodium may be associated with mortality.

**References**

1. Funk G-C, et al. Incidence and prognosis of dysnatremias present on ICU admission. Intensive Care Medicine 2009;36(2):304–11.

2. Gucyetmez B, et al. Dysnatremia on intensive care unit admission is a stronger risk factor when associated with organ dysfunction. Minerva Anestesiology 2014;80(10):1096–104

3. Sakr Y, et al. Fluctuations in Serum Sodium Level Are Associated With an Increased Risk of Death in Surgical ICU Patients: Critical Care Medicine 2013;41(1):133–42.

### A439 Venous-arterial CO_2_ to arterial-venous o_2_ difference ratio (CV-ACO_2_/DA-VO_2_) - the intra-operative anaerobic threshold? An assessment during liver resection surgery

#### E.K. Potter^1,2^, J. Kirk-Bayley^1,3^, N.D. Karanjia^1,3^, L.G. Forni^1,3^, B.C. Creagh-Brown^1,3^

##### ^1^Royal Surrey County Hospital, ICU and SPACeR Research Group, Guildford, UK; ^2^University of Surey, Guildford, UK; ^3^University of Surrey, Guildford, UK

###### **Correspondence:** E.K. Potter - Royal Surrey County Hospital, ICU and SPACeR Research Group, Guildford, UK

**Introduction:** Cv-aCO_2_/Da-vO_2_ has been suggested as a marker of adequacy of resuscitation in shock states. A ratio >1 reflects the metabolic tipping point at which CO_2_ production exceeds oxygen consumption, akin to the anaerobic threshold in cardiopulmonary exercise testing, but with the advantage of measurement from paired arterial and central venous blood gas analysis. It is of prognostic value in critical care, and in sepsis when combined with lactate[1, 2]. We investigate the role of Cv-aCO_2_/Da-vO_2_ in liver resection surgery. Our anaesthetic technique uses fluid restriction, diuresis and venodilation to decrease CVP, which improves outcome[3]. On completion of resection, goal-directed fluid therapy (GDFT) is initiated. This technique provides an ideal model of rapid haemodynamic changes in which to investigate Cv-aCO_2_/Da-vO_2_.

**Objectives:** To establish: 1) Is there a relationship between peri-operative Cv-aCO_2_/Da-vO_2_ and outcome? 2) If Cv-aCO_2_/Da-vO_2_, inverse CO_2_ gap or serum lactate correlate with cardiac output (CO)

**Methods:** This is a subset of data from the prospective observational study MicroHepFlow, investigating microcirculation in liver resection surgery. All patients received general and thoracic epidural anaesthesia, fluid restriction, GTN, remifentanil and furosemide. Oesophageal Doppler (OD) was used at the anaesthetists' discretion. Prospective data was collected at pre-defined time points. The Clavien-Dindo (CD) Classification was used for post-operative complications. Statistical analysis was with linear regression and Fischers exact test.

**Results:** In 31 patients, 17 with CO data, a Cv-aCO_2_/Da-vO_2_ > 1 and lactate ≥2 was common (55 % at maximal desiccation and 52 % following GDFT). There was no consistent relationship between this and the incidence of complications. C-D complications ≥ Grade III were rare (6 %). Neither Cv-aCO_2_/Da-vO_2_, inverse CO2 gap nor serum lactate concentration significantly correlated with CO.

**Conclusions:** The expected positive association between Cv-aCO_2_/Da-vO_2_ > 1 and lactate ≥2 with post-operative complications was not observed, potentially due to a low incidence of serious complications. The lack of significant correlation between Cv-aCO2 / Da-vO2 and inverse CO2 gap with cardiac output is informative and suggests that these variables are poor surrogates of cardiac output in this setting.

**References**

1. Mekontso-Dessap A, Castelain V, Anguel N, et al., (2002) Combination of venoarterial PCO2 difference with arteriovenous O2 content difference to detect anaerobic metabolism in patients. Intensive Care Med. 28: 272–277

2. Ospina-Tascon GA, Umana M, Bermudez W, et al., (2015) Combination of arterial lactate levels and venous-arterial CO2 to arterial-venous O 2 content difference ratio as markers of resuscitation in patients with septic shock. Intensive Care Med 41: 796–805

3. Jones RM, Moulton CE, Hardy KJ, (1998) Central venous pressure and its effect on blood loss during liver resection. Br J Surg. 85: 1058–1060

### A440 Predicting persistent requirement for moderate vasopressor support (vasoplegia) following major gynae-oncology debulking surgery (GODS) - vasogods, a single-centre retrospective ICU study

#### M. Bossy^1^, M. Nyman^2^, A. Tailor^3^, B. Creagh-Brown^4^, SPACeR group (Surrey Peri-operative, Anaesthesia and Critical Care Collaborative Research Group)

##### ^1^Royal Surrey County Hospital, ICU and SPACeR Research Group, Intensive Care, Guildford, UK; ^2^University of Southampton, Medical School, Southampton, UK; ^3^Royal Surrey County Hospital, Department of Gynaecological Oncology, Guildford, UK; ^4^Royal Surrey County Hospital, Intensive Care, SPACeR Research Group, Guildford, UK

###### **Correspondence:** M. Bossy - Royal Surrey County Hospital, ICU and SPACeR Research Group, Intensive Care, Guildford, UK

**Introduction:** Despite goal-directed fluid therapy, vasopressor support may be necessary in the early post-operative period to maintain adequate systemic blood pressure. Persistent requirement for moderate doses of vasopressor support (vasoplegia) has not been reported in patients recovering from following major gynae-oncology debulking surgery (GODS). Risk factors for, and complications of, vasoplegia have not been described.

**Objectives:** Describe the incidence of vasoplegia following GODS, and the risk factors for, and complications of, vasoplegia.

**Methods:** Patients were identified from our ICU patient database (WardWatcher software) and additional clinical details acquired from electronic databases including the IntelliSpace Critical Care and anesthesia (Philips) and a local bespoke system (The Guildford Gynae-oncology database). Statistical analysis was performed using binary logistic regression using log transformed data for all non-parametric variables (SPSS).

**Results:** Between February 2014 and September 2015 there were 176 elective admissions to our ICU for care of patients following GODS. The median age was 67 years (IQR range 56–76) and median weight was 70 kg. 75 % had ovarian or endometrial cancer. The median estimated blood loss (EBL) was 900mls. 67.1 % of this group had an epidural inserted for post-operative analgesia and the mean rate of a standard infusion was 10mls/h. 52.3 % of patients received a vasopressor for at least one hour during their post-operative stay on ICU (either phenylephrine or noradrenaline). 16.5 % of the group remained on a dose of noradrenaline ≥0.1mcg/kg/min at 08:00 on the first post operative day - considered to reflect vasoplegia. Median ICU length of stay (LOS) was 2.0 days (IQ range 1.1-3.0), and median hospital LOS 5.7 days (IQR 4.3-7.1). ICU mortality was < 1 % (1/176) and 6 month mortality 4 % (7/176). The only pre-operative variable associated with requiring vasopressors was haemoglobin; median was 119 g/L in those who required vs. 128 g/L in those who did not (p = 0.02). Epidural use was associated with requiring vasopressors (OR 3.74,CI 1.91-7.30, p < 0.005). Cumulative fluid balance was associated with vasoplegia (OR of log 26.22, CI 4.75-144.59, p < 0.005) and a peak lactate greater than median, 3.1 mmol/L (OR of log 4.99, CI 1.63-15.33, p = 0.005). The presence of vasoplegia was association with a prolonged hospital stay (odds ratio of 6.40, CI 2.31-17.70, p < 0.0005).

Hospital LOS in those who didn't require any vasopressors was 4.9d (3.9-6.1), in those who required some vasopressors was 5.8d (5.0-7.9), and in those with had vasoplegia was 7.0d (6.0-9.8), p < 0.0001 KWT.

**Conclusions:** Lower pre-operative haemoglobin concentration was associated with requirement for vasopressors, as was epidural use and cumulative fluid balance. Vasoplegia was uncommon, associated with cumulative fluid balance and elevated peak lactate, and a prolonged duration of hospital stay. Mortality was low.

### A441 Withdrawn

### A442 Noninvasive cardiac output and pressure measurements with finger cuff during lateral decubitus and one lung ventilation in thoracic surgery: preliminary results

#### D. D'Antini^1^, S. Spadaro^2^, F. Valentino^1^, F. Sollitto^3^, G. Cinnella^1^, L. Mirabella^1^

##### ^1^University of Foggia, Anesthesiology and Intensive Care, Foggia, Italy; ^2^University of Ferrara, Anesthesiology and Intensive Care, Ferrara, Italy; ^3^University of Foggia, Thoracic Surgery, Foggia, Italy

###### **Correspondence:** D. D'Antini - University of Foggia, Anesthesiology and Intensive Care, Foggia, Italy

**Introduction:** Patients undergoing thoracic surgery commonly undergo a radial catheter placement to fulfill the need for continuous blood pressure monitoring, other than blood gas

analyses 1.

**Objectives:** In this prospective observational study, we recorded cardiac output and pressure measurements obtained by an uncalibrated pulse contour method in a totally non invasive manner (ClearSight system, Edwards Lifesciences) to assess hemodynamic variations during normal and one lung ventilation (OLV) in supine and lateral decubitus in thoracic surgery.

**Methods:** After Ethical Committee approval was obtained, we enrolled patients with age > 18 years, scheduled for thoracic surgery and OLV with double-lumen tube in lateral decubitus. Exclusion criteria: ASA > III, severe baseline hemodynamic impairment which required invasive monitoring and amine infusion.

Key hemodynamic parameters, including Stroke Volume Index (SVI), Stroke Volume Variation (SVV), Cardiac Index (CI) and Continuous Blood Pressure (cBP) were recorded at three time points: two lung ventilation in supine position (T1), two lung ventilation in lateral decubitus (T2) and one lung ventilation in lateral decubitus (T3).

**Results:** 11 consecutive patients scheduled for thoracic surgery were included. Overall, our patients showed no hemodynamic impairment throughout the passage to lateral decubitus and OLV (CI 3,1 ± 0,9 l/min/m^2^ at T1, 2.5 ± 0.6 l/min/m^2^ at T2, 2.5 ± 0.7 l/min/m^2^ at T3; p = 0.09 T2 and T3 vs T1). Only in 3 patients a decrease in CI was detected after the position change (−37 %, −42 % and −43 %, respectively, T2 vs T1), which required prompt intervention.

**Conclusions:** The lack of invasiveness and the simplicity of the system allowed satisfactory monitoring and, subsequently, appropriate therapy, without the need of arterial catheter placement for invasive monitoring, while an automatic, intermittent, non-invasive blood pressure measurement would have not readily detected the observed hemodynamic variations.

Even if most studies underline the not-interchangeability of the finger cuff method with the currently used invasive devices^2^, in our low to moderate risk thoracic surgery patients not receiving an arterial line, ClearSight was able to track hemodynamic changes during the different phases of the surgical procedure, in particular those related to position changes and to the OLV.

**References**

1. Haas S, Kiefmann R, Eichhorn V, et al. Anaesthesia. 2009;58(11):1085–96.

2. Ameloot K, Palmers PJ, Malbrain ML. Curr Opin Crit Care. 2015;21(3):232–9.

### A443 The global end-diastolic volume (GEDI) could be more appropiate to reduce the intraoperative bleeding than central venous pressure (PVC) during mayor liver resections (metastasized colon carcinoma)

#### F.J. Redondo Calvo^1^, N. Bejarano^2^, D. Padilla^3^, V. Baladron^4^, P. Villajero^3^, R. Villazala^4^, J. Redondo^4^, A.S. Yuste^4^

##### ^1^Facultad Medicina Ciudad Real, Hospital General Universitario de Ciudad Real. Anestesiologia y Reanimacion, Ciudad Real, Spain; ^2^Facultad Medicina Ciudad Real, Hospital General Universitario de Ciudad Real, Cuidados Criticos Pediatricos, Ciudad Real, Spain; ^3^Facultad Medicina Ciudad Real, Hospital General Universitario de Ciudad Real, Cirugía Hepatobiliar, Ciudad Real, Spain; ^4^Hospital General Universitario de Ciudad Real, Anestesiologia y Reanimación, Ciudad Real, Spain

###### **Correspondence:** F.J. Redondo Calvo - Facultad Medicina Ciudad Real, Hospital General Universitario de Ciudad Real. Anestesiologia y Reanimacion, Ciudad Real, Spain

**Goal of study:** The aim of our study was to evaluate the predictive value of CVP with regard GEDI, and correlate these parameters to cardiac Index (CI) and extravascular lung water index (EVLWI).

**Methods:** Prospective study. Surgical intensive care unit, university hospital.

Patients and interventions: 129 hemodynamic measurements using the PiCCO (Pulsion Medical System, Germany) were performed in 28 patiens during major liver resection.

**Results:** Mean CVP (8,44 +/− 4,22 mmHg) was normal, whereas mean GEDI (625,3 +/− 111,34 mL/m2) was decreased.

Forty-two CVP measurements were elevated despite simultaneous GEDI levels indicating a normal or decreased preload. Sensitivity, specificity, positive predictive value, and negative predictive value of CVP with regard to volume depletion (GEDI < 650) were 8,25 (0–14,66. CI 95 %), 100 (99,89-100, CI 95 %), 100 (50–100, CI 95 %), 29, 4 (21,99-26,96, CI 95 %) respectively. CVP did not correlate to GEDI (r = −0,045, p = 0,42), CI (r = 0,13, p = 0,216) and EVLWI (extravascular lung water index) (r = −0,04, p = 0,38). GEDI significantly correlated to CI (r = −0,23, p < 0,01) and VVS (r = −0,32, p < 0,01).

**Conclusions:** Volume depletion according to GEDI was found in more than half the patiens. The predictive values of CVP with regard to volume depletion were low GEDI and its changes significantly correlated to CI and its changes, which was not observed for CVP. Therefore, GEDI appears to be more appropiate for volume management during mayor liver resections with the aim to avoid intraoperative bleeding and transfusión.

**Note:** This abstract has been previously published and is available at [4]. It is included here as a complete record of the abstracts from the conference.

**References**

1. Smyrniotis V, Kostopanagiotou G, Theodoraki K, et al. The role of central venous pressure and type of vascular control in blood loss during mayor liver resections. Am J Surg. 2004; 187:398–402

2. De la Rocca G, Costa MG, Coccia C, et al. Preload and hemodynamic assesment during liver transplantation. A comparison between pulmonary artery catheter and transpulmonar indicator dilution technique. Eur J Anesth 2002; 19:868–875

3. Huber W, Umgelter A, Reindl W, et al. Volume assessment in patients with necrotizing pancreatitis: a comparison of intrathoracic blood volumen index, central venous pressure, and hematocrit, and their correlation to cardiac index and extravascular lung wáter. Crit Care Med 2008; 36:2348–5.

4. Redondo Calvo FJ, Bejarano N, Padilla D, Villarejo P, Baladron V, Villazalo R, Yuste AS, Arenas P (2015) A comparison of global end-diastolic volume (GEDI) and central venous pressure (CVP) as parameter for volume assessment I patients during major liver resections. Intensive Care Medicine Experimental 3(Suppl 1): A216

**Grant acknowledgement**

We express our gratitude to Mutua Madrileña Fundation (Madrid, Spain) for its grant collaboration by without which this work could not have been completed.

### A444 Incidence, risk factors of weaning-induced pulmonary oedema and effects of fluid removal

#### J. Liu^1,2^, F. Shen^1,3^, J.-L. Teboul^1^, N. Anguel^1^, A. Beurton^1^, N. Bezaz^1^, C. Richard^1^, X. Monnet^1^

##### ^1^Hôpital de Bicêtre, Hôpitaux universitaires Paris-Sud, Université Paris-Sud, Service de réanimation médicale, Inserm UMR_S999, Le Kremlin-Bicêtre, France; ^2^The First Affiliated Hospital of Chongqing Medical University, Department of Emergency Medicine and Critical Care Medicine, Chongqing, China; ^3^Affiliated Hospital of Guizhou Medical University, Department of Critical Care Medicine, Guiyang, China

###### **Correspondence:** X. Monnet - Hôpital de Bicêtre, Hôpitaux universitaires Paris-Sud, Université Paris-Sud, Service de réanimation médicale, Inserm UMR_S999, Le Kremlin-Bicêtre, France

**Introduction:** Weaning-induced pulmonary oedema (WiPO) is a well-recognised cause of weaning failure, but its incidence and risk factors have not been established and the effects of fluid removal have not been systematically described.

**Objectives:** To describe the epidemiology of WiPO and the effects of diuretics.

**Methods:** We included 81 patients who performed 283 spontaneous breathing trials (SBT).Three experts established the diagnosis of WiPO based on various patient characteristics. Demographic data were collected. A passive leg raising (PLR) test was performed before SBT in 81 patients. We also observed the effects of diuretics when this treatment was decided.

**Results:** SBT failed in 129 cases (46 % of all SBT). WiPO occurred in 59 % of these failing cases. Compared to patients without WiPO (n = 52), patients with at least one WiPO (n = 29) had a significantly higher prevalence of obesity (45 % vs. 17 %, resp), COPD (38 % vs. 12 %, resp), dilated cardiopathy (48 % vs. 17 %, resp) and low left ventricular ejection fraction (55 % vs. 21 %, resp). In 16 cases with WiPO and a negative PLR at baseline, diuretics were administered. In 9 of these cases, the PLR remained negative before the following SBT. A new episode of WiPO occurred in 7 of these instances while the two other ones were extubated. In 7 other cases, the PLR became positive before the following SBT. WiPO did not occur anymore in 6 of these 7 patients who were extubated, while the remaining one was not.

**Conclusions:** WiPO was responsible for 59 % of weaning failures. Obesity, previous cardiopathy and COPD were its main risk factors. When fluid removal had changed preload-independence to preload-dependence, the following SBT was very likely to succeed.

### A445 Heart lung interactions measured by means of phase synchronization analysis under three ventilatory modes: pressure control, pressure support and NAVA

#### T. Fossali^1^, R. Colombo^1^, D. Ottolina^1^, M. Rossetti^1^, C. Mazzucco^2^, A. Marchi^2^, A. Porta^3^, E. Catena^1^

##### ^1^Ospedale Luigi Sacco - Milano, Department of Anaesthesia and Intensive Care, Milano, Italy; ^2^Politecnico di Milano, Department of Electronics information and Bioengineering, Milano, Italy; ^3^Università degli studi di Milano, Department of Biomedical Science of Health, Milano, Italy

###### **Correspondence:** T. Fossali - Ospedale Luigi Sacco - Milano, Department of Anaesthesia and Intensive Care, Milano, Italy

**Introduction:** Pulmonary and cardiac systems are deeply connected in physiologic conditions by autonomic nervous system. In particular Respiratory Sinus Arrhythmia (RSA) modifies heart rate in correspondence of inspiration to permit a better ventilation-perfusion matching. Cardio-respiratory phase synchronization analysis (CRPS) defined as a repetitive occurrence of m heartbeats at the same relative phases within n consecutive breathing cycles, also provides indexes relevant to nonlinear interactions between cardiac and respiratory systems complementary to RSA (1). These mechanisms are poorly studied during mechanical ventilation.

**Objectives:** Aim of the study was to compare cardio-ventilatory interactions in different mechanical ventilation modes by means of CRPS and RSA analysis.

**Methods:** The study was conducted in the Intensive Care Unit of Luigi Sacco Hospital, Milan, Italy. The experimental protocol consisted of three randomized sessions of 30 minutes corresponding to different ventilation modes: Pressure Control Ventilation (PCV), Pressure Support Ventilation (PSV) and Neurally Adjusted Ventilatory Assist (NAVA). Electrocardiographic (ECG) and airway volume (AWV) signals were acquired at 250 Hz sampling rate and continuously registered (Philips IntelliVue MX800, iXtrend software). Signals were analyzed offline: the Hilbert transform was applied to AWV signal to estimate the respiration phase. In the synchrogram the phase values of the AWV observed in correspondence of the R-wave peak on the ECG was drawn as function of the cardiac beat number. The quantification of cardio-respiratory coupling was assessed by the synchronization index (SYNC), i.e. the percentage of beats in which it could be observed the most frequent PLR. RSA amplitude, the difference between the longest and the shortest R-R interval on the ECG, was estimate for each respiratory cycle.

**Results:** 20 mechanically ventilated patients were included. Mechanical ventilation was set to guarantee constant minute ventilation (MV 10 ± 4 vs 10 ± 3 vs 10 ± 2 liters/minute for PCV, PSV and NAVA respectively). CRPS analysis showed a significant variation of SYNC index between the different modes (Fig. 3), while RSA was not able to detect meaningful differences (23 ± 26 ms, 24 ± 24 ms and 22 ± 22 ms, p = 0.6, for PCV, PSV and NAVA respectively).

**Conclusions:** Analysis of CRPS is possible in ventilated patients. Our results endorse the hypothesis that under ventilation with a fixed frequency, the interactions between cardiac and respiratory fluctuations were stronger than in the case of patient driven modes of mechanical ventilation, as PSV and NAVA (2). The lack of variation of RSA may be due to sedation and mechanical ventilation.

**References**

1. Schäfer C et al., Heartbeat synchronized with ventilation. Nature. 1998 Mar 19;392(6673):239–40.

2. Larsen PD et al., Cardioventilatory coupling: effects of IPPV. British Journal of Anaesthesia. 1999 82; 546–550.

## Drugs and poisons in the ICU

### A446 Substance abuse as predictor of in-hospital mortality in patients with cardiac arrest

#### K.H. Tollisen^1^, G.Ø. Andersen^2^, F. Heyerdahl^3^, D. Jacobsen^4^

##### ^1^Oslo University Hospital, Acute Medicine, Oslo, Norway; ^2^Oslo University Hospital, Departement of Cardiology, Oslo, Norway; ^3^Oslo University Hospital, Departement of Anesthesiology, Oslo, Norway; ^4^Oslo University Hospital, Departement of Acute Medicin, Oslo, Norway

###### **Correspondence:** K.H. Tollisen - Oslo University Hospital, Acute Medicine, Oslo, Norway

**Introduction:** Initial cardiac rhythm (ICR) is associated with mortality in cardiac arrest (CA) patients with return of spontaneous circulation (ROSC) [1]. However, knowledge regarding a possible association between substance abuse and mortality is limited.

**Objectives:** (1)To determine whether substance abuse is associated with in-hospital mortality in patients with CA admitted to ICU`s (intensive care units), and (2) to study the association between substance abuse and ICR.

**Methods:** CA patients with ROSC-- admitted to the ICU`s at Oslo University Hospital Ullevål from Feb.3, 2014 to Feb.2,2015 -- were included as part of a multicenter prospective observational study of ICU patients in Oslo (n = 900). Informed consent was given by the next of kin, and if possible, later by the patient. Illicit drug/alcohol abuse (acute or chronic) was assessed via standard questionnaires, biochemical analysis (drug screen) and medical records. The patients were divided into groups based on ICR: 1) non-shockable rhythm (asystole/pulseless electric activity) and 2) shockable rhythm (VF/VT). Chi-square test was used for statistical analysis, p value < 0.05 was considered statistically significant.

**Results:** 145 patients (116 (80 %) males)) were included, reflecting a participation rate of 95 %. 72(50 %) had non-shockable rhythm, and 73(50 %) had shockable rhythm (mean age 62 vs 63). There were significantly more females in the non-shockable group (21vs.8, p < 0.01), corresponding with earlier studies. Acute drug intoxication was considered triggering cause in 7 patients (5 %), all males (mean age 43vs 64). Main agents were: opioids (4/7), alcohol (2/7,both with blood levels > 4.2 g/L (92 mmol/L)) and benzodiazepines (1/7). All these patients were in the non-shockable group. However, the sample size was too small for further analysis to be pursued.

Among the 31 (21 %) classified with chronic substance abuse (27(87 %)males, mean age 59 vs. 64) alcohol was the predominant substance (75 %), followed by illicit substances (21 %) and prescription drugs (5 %). The proportion of patients with chronic abuse was significantly higher among patients with non-shockable rhythm (32 % vs 13 % p = 0.005). In-hospital mortality was significantly higher among patients with non-shockable rhythm (83 % vs 42 %, p < 0.001). Chronic abuse, however, was not significantly associated with in-hospital mortality (p = 0.43).

**Conclusions:** Chronic substance abuse is common among patients with CA and is significantly associated with non-shockable ICR. As expected, in-hospitality mortality was significantly higher in the non-shockable group. Although chronic abuse was associated with non-shockable initial rhythm, it was not associated with in-hospital mortality.

**References**

1. Sasson, C., et al., *Predictors of survival from out-of-hospital cardiac arrest: a systematic review and meta-analysis.* Circ Cardiovasc Qual Outcomes, 2010. **3**(1): p. 63–81.

### A447 Volatile or intravenous anesthetics in a mouse model of cardiac arrest?

#### M.C. de Waard, A.R.J. Girbes

##### VU University Medical Center Amsterdam, Amsterdam, Netherlands

###### **Correspondence:** M.C. de Waard - VU University Medical Center Amsterdam, Amsterdam, Netherlands

**Introduction:** Cardiac arrest (CA) is a major health problem in western countries. Despite improvements in quality of treatment, it still has a high rate of morbidity and mortality in patients. CA results in a harmful inflammatory response and overwhelming amounts of reactive oxygen species are produced, spreading out across the body, causing leakage of vascular walls and damage to organs. The mouse is an increasingly used preferred model in preclinical CA studies due to the knowledge concerning the mouse genome and the general availability of genetically modified mice. Volatile anesthetics are often used in these models as they are easy to apply and short-acting. However, volatile anesthetics are known to protect against I/R injury, reduce myocardial infarct size by preconditioning and may protect vital organs by postconditioning.

**Objectives:** This pilot study investigates the use of i.v. or volatile anesthetics in a mouse model for CA.

**Methods:** Experiments were approved by the ethical committee of VU Medical Centre Amsterdam. Mice randomly assigned to one of the two groups. Thereafter they were weighed, sedated with 4 % sevoflurane and buprenorphine (0.05 mg/kg s.c.) or a mixture of fentanyl/midazolam/acepromazine (FMA: 0.47 mg/kg, 9.38 mg/kg, 9.38 mg/kg; respectively i.p.), intubated and placed on a heating mat. The mice were pressure-controlled ventilated with 21 % O_2_ (90–100 breaths/min, PEEP 4cmH_2_O, peak 16cmH_2_O). Anaesthesia was maintained with 2.5-3 % sevoflurane or with FMA (0.08 mg/kg, 1.56 mg/kg, 1.56 mg/kg i.v.). 21 mice were sedated and anesthetized with sevoflurane and 6 with FMA. Electrocardiogram (ECG) was recorded continuously. For measurement of aortic pressure, a fluid filled polyethylene catheter was inserted in the right carotid artery, advanced into the aortic arch and connected to a pressure transducer. Via a fluid filled polyethylene catheter, inserted in the jugular vein, potassium chloride (KCl: 0.08 mg/kg) was administered and subsequently CA induced. CA was defined as a mean arterial pressure (MAP) of less than 20 mmHg and no ECG activity and lasted for 8 min. Thereafter the mice were resuscitated by manual chest compression (~300/min) and a bolus 0.1 ml epinephrine (0.1 mg/ml i.v.). Resuscitation ended after 15 min or upon ROSC, defined as sinus rhythm on ECG and mean arterial pressure >40 mmHg lasting at least 5 min.

**Results:** 17 out of 21 (81 %) mice in the sevoflurane group achieved ROSC within 90 seconds after the start of resuscitation. In contrast, none of the 6 mice sedated and anesthetized with FMA achieved ROSC within 15 minutes after CA. No significant differences in aortic pressure, heart rate or body temperature was observed before CA.

**Conclusion:** Sevoflurane is the best choice for anesthetics in a mouse CA model with successful resuscitation. However, our data suggest a profound postconditioning effect of sevoflurane which should be investigated in further detail.

### A448 Hydrochlorothiazide in ICU-acquired hypernatremia - a randomized controlled trial

#### M.C.O. van IJzendoorn^1,2^, H. Buter^1^, W.P. Kingma^1^, G.J. Navis^2^, E.C. Boerma^1^

##### ^1^Medical Center Leeuwarden, Intensive Care, Leeuwarden, Netherlands; ^2^University Medical Center Groningen, Internal Medicine / Nephrology, Groningen, Netherlands

###### **Correspondence:** M.C.O. van IJzendoorn - Medical Center Leeuwarden, Intensive Care, Leeuwarden, Netherlands

**Introduction:** Impaired renal sodium excretion may contribute to ICU-acquired hypernatremia (IAH). Thiazides are suggested as an effective treatment by interfering in renal sodium reabsorption [1].

**Objectives:** Primary aim of the study was to reduce serum sodium concentration (sNa) in patients with IAH, defined as sNa ≥ 143 mmol/l, with hydrochlorothiazide (HCT) in comparison to placebo. Secondary endpoints were a difference in urine sodium concentration (uNa) and the duration of severe IAH, defined as sNa ≥ 145 mmol/l.

**Methods:** A monocentric, double-blind placebo-controlled trial was conducted in 50 patients with IAH and an impaired renal cation excretion, defined as (urine potassium + uNa) < sNa in a spot urine sample. Patients were randomized for enteral HCT 25 mg (n = 25) or placebo (n = 25) 1qd for a maximum of 7 days. Patients on renal replacement therapy, on medications inducing diabetes insipidus or with recent use of diuretics were excluded. The trial received no funding and was registered at clinicaltrials.gov (NTC01974739).

**Fig. 17 (abstract A448).**
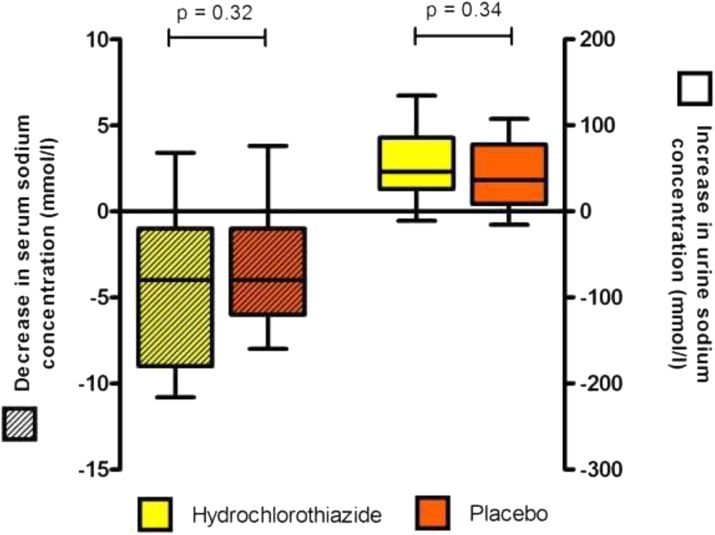
Decrease sNa and increase uNa at study end

**Results:** Baseline characteristics in both groups were comparable. At baseline median sNa was 146 mmol/l in both groups ([145–148] in the HCT-group, [144–150] in the placebo group, p = 0.4). Over time sNa decreased significantly with 4 mmol/l in both groups, with no significant difference between groups. At baseline uNa was 25 [10–62]mmol/l in the HCT-group and 20 [10–66]mmol/l in the placebo group. Over time uNa increased significantly in both groups: 46 [16–86]mmol/l in the HCT-group and 36 [9–78]mmol/l in the placebo group, with no difference between groups (Fig. 17, Table 7). Median duration of sNa ≥ 145 mmol/l was 3 days in both groups (p = 0.91). No adverse advents or side effects were registered.

**Conclusions:** HCT 25 1qd did not significantly affect sNa nor uNa in patients with IAH.

**References**

1. Overgaard-Steensen C, Ring T. (2013) Clinical review: Practical approach to hyponatraemia and hypernatraemia in critically ill patients. Crit Care. 17(1):206.

**Table 7 (abstract A448).** Results at last day of study inclusion

	HCT (n = 25)	Placebo (n = 25)	P-value
Serum [Na+], mmol/l	141 [137–147]	144 [139–146]	0.32
Urine [Na+], mmol/l	110 [70–124]	84 [52–126]	0.34
Decrease in serum [Na+], mmol	4 [1–9]	4 [2–6]	0.47
Increase in urine [Na+], mmol	46 [26–86]	36 [9–78]	0.31

### A449 Mass methanol poisoning in the czech republic 2012/2013. Hospital costs and clinical outcome

#### J. Rulisek^1^, M. Balik^2,3^, S. Zacharov^4^

##### ^1^1st Medical Faculty, Charles University, Anaesthesia and Intensive Care, Prague, Czech Republic; ^2^Vseobecna Fakutni Nemocnice, KARIM, Prague, Czech Republic; ^3^General University Hospital, 1st Faculty of Medicine, Charles University in Prague, Prague, Czech Republic; ^4^1st Medical Faculty, Charles University, Prague, Czech Republic

###### **Correspondence:** J. Rulisek - 1st Medical Faculty, Charles University, Anaesthesia and Intensive Care, Prague, Czech Republic

**Introduction:** Most of mass methanol outbreaks are reported in underdeveloped countries were financial resources are scarce. In The Czech Republic, in 2012–2013 the mass methanol outbreak was reported with 138 victims. Finding optimal cost-effective treatment is cornerstone for every medical system.

**Objectives:** Primary objective of our study was to define total hospital cost, find major parameters which influence total hospital cost and its relation to clinical outcome. Choice of antidote (ethanol or fomepizol) and choice of elimination technique (continuous or intermittent hemodialysis), its impact to hospital costs and clinical outcome were analyzed.

**Methods:** In cooperation with Czech Toxicology Information Centre (TIC) and Czech medical insurance companies reimbursed medical costs were defined. 54 survivors fulfilled follow up investigation. Hospital costs and its correlation to admission parameters was provided. Effectiveness of treatment modalities and consequent medical costs were analyzed.

**Results:** The median hospital costs were 2,422 (interquartile range, IQR: 1,364-4,748) euros. The median one-year medical costs were 633(IQR 439–1,228) euros. Anion gap, pH, HCO3-, lactate, serum creatinine, methanol level, decreased GCS strongly correlated with total hospital costs. All non-survivors were comatose at admission. Fomepizol treatment increase adjusted hospital costs 2,8 times without difference in clinical outcome. Choice of intermittent hemodialysis led to faster residual methanol clearance, formic acid clearance and showed trend to decreased hospital costs.

**Conclusions:** We were able to define major admission parameters that have impact to total hospital costs. Fomepizol antidote significantly increases costs without impact to clinical outcome. Intermittent hemodialysis shortens secondary elimination time and tend to lower costs.

### A450 Risk factor of linezolid-induced thrombocytopenia in the intensive care unit

#### H.S. Kim^1^, S.J. Jeon^1^, H. Namgung^1^, E. Lee^1^, E. Lee^2^, Y.-J. Cho^3^, Y.J. Lee^3^

##### ^1^Seoul National University Bundang Hospital, Department of Pharmacy, Seongnam-si, Republic of Korea; ^2^Seoul National University, College of Pharmacy, Seoul, Republic of Korea; ^3^Seoul National University Bundang Hospital, Division of Pulmonary and Critical Care Medicine, Department of Internal Medicine, Seongnam-si, Republic of Korea

###### **Correspondence:** H.S. Kim - Seoul National University Bundang Hospital, Department of Pharmacy, Seongnam-si, Republic of Korea

**Introduction:** Few studies have evaluated the prognostic importance of linezolid-induced thrombocytopenia in intensive care unit (ICU) patients because there are many confounding conditions such as sepsis, disseminated intravascular coagulation, and low thrombocytopenia level when admitted to the ICU.

**Objectives:** We determined the prevalence and risk factors of linezolid-induced thrombocytopenia in the ICU.

**Methods:** Patients treated with linezolid from January 2005 to December 2015 in the ICU were reviewed retrospectively. To differentiate other causes of thrombocytopenia, the Naranjo algorithm was used to determine the degree of probability for every linezolid-induced thrombocytopenia.

**Results:** Sixty patients were included in this study. The mean (SD) age of the patients was 69.8 (±11.9) years. Twenty-nine patients (48.3 %) presented linezolid-induced thrombocytopenia. Patients with thrombocytopenia had higher incidence of any malignancy (41.4 % vs. 9.7 % p = 0.007), elevated baseline creatinine (1.7 [0.9-2.5] vs. 0.9 [0.6-1.3], median [IQR] mg/dL, p = 0.042) and lowered platelet on the first day of linezolid administration (160 [128–230] vs. 194 [118–285] *103/mm3 median [IQR], p = 0.296). Patients who developed thrombocytopenia received more transfusions (34.5 % vs. 6.5 %, p = 0.009) and showed increased ICU mortality (62.1 % vs. 32.3 %, p = 0.037). By means of a logistic regression model, higher incidence of any malignancy (odds ratio, 9.060, 95 % confidence interval, 1.942-42.271) and elevated serum creatinine level after linezolid administration (odds ratio, 1.655, 95 % confidence interval, 1.027-2.669) were significant risk factors of linezolid-induced thrombocytopenia in the ICU.

**Conclusions:** Patients with any malignancy or elevated baseline creatinine who were treated with linezolid in the ICU are more likely to develop thrombocytopenia.

**References**

1. Zhang Z, Liang Z, Li H, Chen L, She D (2013) Comparative evaluation of thrombocytopenia in adult patients receiving linezolid or glycopeptides in a respiratory intensive care unit. Exp Ther Med 7(2):501–507. doi: 10.3892/etm.2013.1437

2. James N George (2015) Drug-induced thrombocytopenia UptodateWeb. http://www.uptodate.com/contents/drug-induced-thrombocytopenia. Accessed 20 February 2015.

3. John Papadopoulos (2012) Drug-Induced Complications in the Critically Ill Patient: A Guide for Recognition and Treatment, New York

4. Naranjo CA, Busto U, Sellers EM, Sandor P, Ruiz I, Roberts EA, Janecek E, Domecq C, Greenblatt DJ (1981) A method for estimating the probability of adverse drug reactions. Clin Pharmacol Ther. 30(2):239–245.

**Grant acknowledgement**

I wish to express my sincere thanks to Eunsook Lee, the chief of department of pharmacy, for providing me with all the necessary facilities. I take this opportunity to record my sincere thanks to all the faculty members of the ICU multidisciplinary team for their help and encouragement.

### A451 Physiological and biochemical effects of an intravenous furosemide bolus in critically ill patients

#### A. Huang^1,2^, L. Cioccari^1,3^, N. Luethi^1^, J. Mårtensson^1,4^, R. Bellomo^1,5^

##### ^1^Austin Health, University of Melbourne, Department of Intensive Care, Heidelberg, Australia; ^2^Changi General Hospital, Department of Anaesthesia and Surgical Intensive Care, Singapore, Singapore; ^3^Lucerne Cantonal Hospital, Department of Intensive Care, Lucerne, Switzerland; ^4^Karolinska Institutet, Section of Anaesthesia and Intensive Care Medicine, Department of Physiology and Pharmacology, Stockholm, Sweden; ^5^The University of Melbourne, Parkville, Melbourne, Australia

###### **Correspondence:** L. Cioccari - Austin Health, University of Melbourne, Department of Intensive Care, Heidelberg, Australia

**Introduction:** Intravenous boluses of furosemide are commonly given to critically ill patients to modulate fluid balance^1–3^. However, their effect on urinary output (UO), fluid balance, electrolyte losses and hemodynamics has not been systematically studied. Therefore, we sought to compare UO, fluid balance, electrolyte losses, biochemical, and hemodynamic effects in the six hours before and after the administration of a 40 mg IV bolus of furosemide.

**Methods:** We conducted a prospective observational study in critically ill patients where the attending clinician had decided to administer a 40 mg bolus of IV furosemide. We collected information on UO, fluid balance, urinary electrolyte losses, serum and urinary creatinine, serum biochemistry, and hemodynamics and compared the 6-hour pre-bolus period with the 6-hour post-bolus period. We performed linear regression analysis to identify predictors of the extent of urinary output response.

**Results:** We studied 24 patients (13 females) with a median (IQR) age of 72 (64, 81) years. The intravenous bolus induced a > 1000 ml increase in urine output (UO) in 6 (25 %) patients, a 500–1000 ml increase in 7 (29.2 %) patients, a < 500 ml increase in 9 (37.5 %) patients, and no increase in 2 (8.3 %) patients. The median UO increased in 23 (96 %) patients and failed to increase in 1 (4 %) patient for a median difference in UO of 561 (250, 1046) ml. The 6-hour fluid balance became negative in 16 (72.7 %) patients and positive in 8 (36.3 %) patients, with a median change of −520 (−1206, 98) ml. However, we observed no difference in free water clearance. Furosemide significantly increased urinary sodium, potassium and chloride losses by 67 (31, 129) mmol, 9.4 (1.8, 18) mmol and 61 (44, 138) mmol respectively, and decreased blood chloride levels by 1.5 (0.5, 3.0) mmol/l. There were no detectable changes in hemodynamics. On linear regression analysis, UO increased by 37 ml (95 % CI 19 to 56 ml) / 6 hour for every 1 mmHg increase in mean arterial pressure and decreased by 35 ml (95 % CI 9 to 61 ml) / 6 hour for every 1 g/L increase in serum albumin.

**Conclusions:** We defined the overall median effect of furosemide on urine output, urinary sodium, potassium and chloride losses and fluid balance in critically ill patients and provided information on how such effects were modulated by mean arterial pressure and albumin levels. Our findings are likely to assist clinicians in their therapeutic expectations and choices.

**References**

1. Bagshaw SM, Delaney A, Jones D, et al. Diuretics in the management of acute kidney injury: a multinational survey. Contributions to nephrology 2007;156:236–249.

2. Mehta RL, Pascual MT, Soroko S, et al. Diuretics, mortality, and nonrecovery of renal function in acute renal failure. Jama 2002;288(20):2547–2553.

3. Uchino S, Doig GS, Bellomo R, et al. Diuretics and mortality in acute renal failure. Critical care medicine 2004;32(8):1669–1677.

**Grant acknowledgement**

None.

### A452 No support for the use of intravenous lipid emulsion in oral poisoning -a systematic review and analysis of 169 published cases

#### M. Forsberg^1^, G. Edman^1^, J. Höjer^2,3^, S. Forsberg^3,4^

##### ^1^TioHundra AB, Norrtälje, Sweden; ^2^Swedish Poisons Information Centre, Stockholm, Sweden; ^3^Department of Clinical Science and Education, Södersjukhuset, Karolinska Institutet, Stockholm, Sweden; ^4^TioHundra AB, Anaesthesia and Intensive Care, Norrtälje, Sweden

###### **Correspondence:** M. Forsberg - TioHundra AB, Norrtälje, Sweden

**Introduction:** In 1998, Weinberg and colleagues reported results from a study that pointed towards reduced bupivacaine toxicity by intravenous lipid emulsion (ILE) in rats[1]. The use of ILE as an antidote has increased dramatically and associations in several countries have launched guidelines regarding the use of ILE in local anaesthetic systemic toxicity (LAST). Recently, two systematic reviews from the international expert team 'Lipid Emulsion Therapy Workgroup' were published[2,3]. The review on ILE for LAST concluded that treatment appears to be effective in some cases of LAST, but there is no evidence showing that ILE is more effective than vasopressors. The review on ILE for non-local anaesthetic toxicity concluded that the quality of evidence for ILE being an effective antidote remains low to very low.

**Objectives:** We present a systematic review including all published reports regarding humans treated with lipid rescue from 1998 until 2016.

**Methods:** Web of science and PubMed were used for the literature search. To limit the search we used predefined criteria. This resulted in 195 papers, which were independently reviewed and categorized by using a modified version of WHO-UMC causality categories[4]. This is a 6-graded assessment scale that combines clinical and pharmacological aspects with the quality of documentation. Grade 1 implies a certain causal connection, 2 probable, 3 possible and 4 unlikely. Grade 5 implies none or negative effect of ILE and 6 unassessable. Log p-values were identified for all involved toxins. Further, the reviewing experts evaluated which main symptom reasonable had led to the decision of giving ILE.

**Results:** In total, 135 case reports received grade 1–4 and 34 grade 5. Forty of the 135 case reports that had reported a positive effect of ILE were parenteral intoxications while 95 were oral poisonings. Three of 135 cases were classified as a certain correlation between giving ILE and improvement of symptoms, 34 cases as probable correlation, 71 possible and 27 unlikely. Mean classification grade in parenteral intoxications was 2.3. Mean classification grade among the oral poisonings was 3.1, which was a highly significant difference. None of the oral poisonings received the classification grade 'certain'. The parenteral intoxications had a mean log p-value of 4.2 which was significantly higher than that for oral poisonings which was 3.2. Indications for ILE in parenteral intoxications were cardiac arrest and neurological symptoms and for oral poisonings hypotension and arrhythmia.

**Conclusions:** The evidence for ILE being an effective antidote is sparse. Thirty-four out of 135 published cases indicates no or negative effect, despite the immense impact of publication bias. It seems reasonable not to use ILE in oral poisonings.

**References**

1. Weinberg Anesthesiology 1998;**88**:1071–5.

2. Hoegberg Clin Toxicol 2016;epubl

3. Levine Clin Toxicol 2016;epubl

4. WHO-UMC http://who-umc.org/Graphics/24734.pdf.

### A453 Fondaparinux treatment in patients with acute Heparin-induced thrombocytopenia and acute renal failure with continuous renal replacement therapy

#### M.T. Chiquito Freile^1^, F.N. Hidalgo^1^, J.A. Martinez Molina^1^, R. Lecumberri^2^, A. Figuerola Rosselló^1^, P. Medrano Travieso^1^

##### ^1^Clinica Universidad de Navarra, Anesthesiology, Pamplona, Spain; ^2^Clinica Universidad de Navarra, Hematology, Pamplona, Spain

###### **Correspondence:** M.T. Chiquito Freile - Clinica Universidad de Navarra, Anesthesiology, Pamplona, Spain

**Introduction:** Inmune Heparin-induced thrombocytopenia (HIT) is a prothrombotic disorder caused by an immune-mediated complication that results from a platelet activating inmune response triggered by the interaction of heparin with a specific platelet protein, platelet factor 4 (PFA). Fondaparinux is a safety treatment of HIT, even though is not recommended in acute renal failure (ARF) because of it renal elimination and significant risk of accumulation.

**Objectives:** Report the safety profile and efficacy of fondaparinux (thrombotic and major bleeding complications) in patients with HIT and ARF during continuous renal replacement therapy (CRRT).

**Methods:** We reviewed consecutive elegible patients with HIT and ARF in one medical university center (Clínica Universidad de Navarra) over a 5 year period, who received fondaparinux for anticoagulation at the time of the HIT diagnosis.

**Results:**

- Of the 12 patients recorded that received fondaparinux as anticoagulation therapy, 5 of them shared the criteria of HIT and ARF with CRRT. We analyzed four of the five patients, excluding one because of loss of information.

- The mean age of the patients was 67 yo, three men and one woman. All of them present acute renal failure with a mean basal creatinine of 2.57, meeting the criteria of CRRT.

- All patients had cardiac surgery with heparin administration last more than 24 hrs with a mean of 4.5 days.

- Platelets at admission were documented and in all cases they dropped more than 50 % of the original value.

- After the administration of Fondaparinux, all the patients had a recovery of the platelet count until the basal value, with a mean of 28.25 days.

- None of the patients developed new, recurrent or progressive thrombosis. No major bleeding complications were documented.

**Conclusions:** Fondaparinux is a safety alternative of anticoagulation therapy in patients with HIT and ARF during continuos renal replacement therapy.

**References**

- Yasser Sakr, Heparin-induced thrombocytopenia in the ICU: an overview. Crit Care.2011; 15:211

- Honore et al. Fondaparinux: another potential treatment for heparin-induced thrombocytopenia type II. Crit Care. 2016; 20:14

- T.E. Warkentin et al., Fondaparinux treatment of acute heparin-induced thrombocytopenia confirmed by the serotonin-release assay: a 30-month, 16 patient case series. Journal of Thrombosis and Haemostasis, 9: 2389–2396

### A454 Hyperthermia and multiple organ dysfunction syndrome associated with methamphetamine derivatives and 3,4-methylenedioxymethamphetamine ("ecstasy")

#### G. Tuero Leon^1^, J. Gonzalez Sanchez^1^, L. Sahuquillo Frias^2^, D. Balsells Rosello^2^, J.A. Garcia Verdejo^1^, J.A. Noria Serrano^1^

##### ^1^Hospital Can Misses, Intensive Care Unit, Ibiza, Spain; ^2^Hospital Can Misses, Clinical Analysis Service, Ibiza, Spain

###### **Correspondence:** G. Tuero Leon - Hospital Can Misses, Intensive Care Unit, Ibiza, Spain

**Introduction:** Hyperthermia with rhabdomyolysis and Multiple Organ Dysfunction Syndrome (MODS) is a rare, life-threatening complication of methamphetamine derivatives (MA) and 3,4-methylenedioxymethamphetamine (MDMA) consumption. The association between hyerthermia and MODS was established from case reports subject to publication bias.

**Objectives:** To explore the association between hyperthermia and MODS development in patients requiring ICU admission due to MA/MDMA intoxication.

**Methods:** Patients with a positive urine test for MA (Triage TOX Drug Screen. Biosite. USA) who required admission to ICU at Can Misses Hospital between the 1st of January 2004 and the 31th of December 2014 were identified. Patients admitted for major trauma, near-drowning or acute coronary syndrome were excluded. Vital signs, clinical data, toxicological results, time prior to ICU admission and mortality at discharge from ICU were recorded. Only tympanic temperatures were considered. SAPS-3 and daily SOFA scores were calculated. Hyperthermia was set as a tympanic temperature above 38,9 °C. MODS was defined by the presence of a score of 3 or 4 in at least two items of the SOFA scale, excluding the neurological at 48 hour of admission or at the time of death.

**Results:** Forty patients were identified after excluding three according to pre-established criteria. They were predominantly young (26 years (19–41)) male (33 patients) with no previous medical history (33 patients). The co-use of other substances was common. The main cause of admission was altered level of consciousness (35 patients), 25 (62.5 %) were in coma. Four cases were admitted due to an out-of-hospital cardiac arrest. Seventeen patients (42.5 %) presented hyperthermia, of which eleven had MODS (4 died within the first 24 hours). No patient with a maximum temperature below 39 °C developed MODS. Six patients with hyperthermia (35.2 %) had a favorable outcome without MODS. Among them, the maximum temperature (40.62 ± 0,7 vs 41.46 ± 0.7 °C, p 0,03), the maximum value of CPK (3.967 ± 1.283 vs 40.387 ± 1.027 U/L, p 0.01), the SAPS-3 (45,5 ± 4 vs 80,7 ± 3, p < 0,001) and SOFA scores (6 (0–9) vs 10 (6–19), p 0,001 at 24 hours; 2 (0–8) vs 15 (13–17), p 0,003 at 48 hours) were significantly lower. No differences in origin (e.g. club), co-use of other substances or temperature control measures were observed among this group. There was a trend toward a greater delay in admission to ICU (176 ± 77 vs 103 ± 9 min, p 0.76) that was not statistically significant among this group. All patients admitted due to out-of-hospital cardiac arrest presented with hyperthermia (41,8 ± 0,5 °C) and MODS (3 died within the first 24 hours).

**Conclusions:** 1. Hyperthermia appears to be strongly associated with the presence of MODS in patients with MA/MDMA intoxications.2. There is a subset of patients with hyperthermia who do not develop MODS. This is not fully explainable by differences in peak temperature or in hyperthermia management.

### A455 Double-check of IV medication at the bedside; does it bring safety or risks?

#### D. Winterwerp^1^, T. van Galen^2^

##### ^1^RN ICU Nurse, VU University Medical Centre, Amsterdam, Netherlands; ^2^ICU Nursing staff manager, VU University Medical Center, Amsterdam, Netherlands

###### **Correspondence:** D. Winterwerp - RN ICU Nurse, VU University Medical Centre, Amsterdam, Netherlands

**Introduction:** The Dutch Healthcare Inspectorate and Quality Institutes mandated hospitals to perform a double-check during IV medication preparation and administering at the bedside. Double check during IV med. preparation was already secured into the workflow. The bedside check is an addition to the nursing workload. The ICU nursing staff of VU University medical center (VUmc) is concerned that the bedside check will not be as effective as expected and that implementing this double check will generate disturbances in other ICU processes.

**Objectives:** Perform a workload measurement during 4 ICU shifts. Perform a literature study and survey other ICU's. Interview with the dedicated ICU pharmacist.

**Methods:** The workload measurement was performed to clarify how much extra time the bedside check added to the normal nursing task performance. We measured the frequency and time of the bedside check. The literature study was performed to find evidence of experiences and potential problems with the single check versus double check. Interviewing the pharmacist was to inquire the concerning double checks as well as supporting advice.

**Results:** The workload measurement has been performed during four 8-hour shifts. There were 9 to 12 patients admitted at the ICU during these shifts. The N/P ratio was 1:1.5 up to 2.0. The additional check takes 1,5 min. per patient, this is added to the 2 minutes that the IV med. preparation check already takes. The bedside check added 27 interruptions to the 15 interruptions for IV med. preparation because the preparation check included multiple medications simultaneously and the bedside check 1 of 2 IV meds per moment.

There are more quantitative studies then qualitative studies [1]. Most studies conclude that more studies need to be conducted [2].

Other Dutch hospitals struggle with the same problem. Some hospitals enlarged pharmacy service to prepare more medication to decrease nurse's workload.

**Discussion:** Evaluating double check workload, it was less time consuming then expected although the frequency of the check brings more interruptions. Every interruption brings a disturbance to nurse's concentration and work, this could potentially increase patient risk. Therefore the amount of interruptions should be reduced to a minimum. Meanwhile, technology makes giant leaps. This has the potential to replace the double check. BCMA is making progress and is more efficient and foul proof than human checks. Because of the wide spread variety in results its hard to draw further Conclusions: It is even doubtful whether further studies make sense, given the current advances in technology. If available, BCMA technique (combined with CPOE) should be implemented as soon as possible. If not available, than hospitals should focus on a select part of medication checking based on specific risks and workflow [3].

**References**

1. What is the Evidence on Double Checks? 2012

[2][3]Does a double check for IV medication improve patient safety? Rachel Cox

### A456 Rational colistin use in the intensive care units of a university affiliated hospital in Iran based on a standard treatment guideline

#### A. Vazin^1^, I. Karimzade^2^, A. Zand^2^

##### ^1^Shiraz University of Medical Sciences, Clinical Pharmacy Department, Shiraz, Islamic Republic of Iran; ^2^Shiraz University of Medical Sciences, Shiraz, Islamic Republic of Iran

###### **Correspondence:** A. Vazin - Shiraz University of Medical Sciences, Clinical Pharmacy Department, Shiraz, Islamic Republic of Iran

**Introduction:** Inadequate antimicrobial treatment of nosocomial infections is an independent determinant of mortality in the critically ill patient. Multi-drug resistant Gram-negative bacilli, mainly Acinetobacter species, are responsible for a significant proportion of nosocomial infections. Acinetobacter has been found to be resistant to many currently available antimicrobial agents. Recently there has been renewed interest in colistin, which had earlier been abandoned for serious adverse effects.

**Objectives:** To assess rational colistin use in a prospective study at the ICU wards of a university affiliated hospital in Iran

**Methods:** Standard treatment Guideline for colistin use were provided and approved by pharmacy and therapeutics committee and Study criteria were developed to assess the several parameters involved in colistin therapy. These parameters include the appropriateness of indication, dosing regimen, duration of therapy and monitoring for toxicity. Clinical and paraclinical parameters such as Glomerular Filtration Rate (GFR), microbial culture, antibacterial sensitivity, WBC count and fever were collected and recorded for analysis.

**Results:** One hundred patients were enrolled in this study, including 64 male and 36 female patients. In most of patients(97 %), the origin of infection was hospital acquired.In this study colistin therapy was 87 % specific, in the remaining 13 %, the initial therapy with colistin was empirical. The most frequent infection was ventilator associated pneumonia (69 %). None of the patients received loading dose. The maintenance dose of colistin in 76 %, dosing interval in 71 %, and duration of treatment in 84 %, was appropriate. In this study, 68.12 % of colistin use was compatible with standard treatment guideline.

**Conclusions:** Based on the results of this study,the main observed drawbacks were inappropriate dosing regimen,especially not considering loading dose in life threatening infections, inappropriate maintenance dose, and continuation of colistin, which has to be revised in order to achieve an effective treatment.

**References**

1. Shahbazi F, Dashti-khavidaki S. Colistin: efficacy and safety in different populations,Expert Rev. Clin. Pharmacol.2015:8(4):423–448.

2. Napier BA, Burd EM, Satola SW, et al. Clinical use of colistin induces cross-resistance to host antimicrobials in Acinetobacter baumannii. mBio.2013: 4(3):e00021-13.

3.Berlana D, Llop JM, Fort E, et al. Use of colistin in the treatment of multiple-drug-resistant gram-negative infections. Am J Health Syst Pharm 2005; 62:39.

4. Cantón R, Horcajada JP, Oliver A, Garbajosa PR, Vila J. Inappropriate use of antibiotics in hospitals: The complex relationship between antibiotic use and antimicrobial resistance. Enfermedades Infecciosas y Microbiología Clínica. 2013;31:3–11

**Grant acknowledgement**

We would also like to thank Shiraz University of medical sciences for financial support of this study.

### A457 Pregnancy and intoxication: a retrospective analysis of 18 patients

#### E. Ozen, S. Ekemen, A. Akcan, E. Sen, B. Buyukkidan Yelken

##### ^1^Eskisehir Osmangazi University Faculty of Medicine, Department of Anesthesiology and Reanimation, Division of Intensive Care, Eskisehir, Turkey

###### **Correspondence:** E. Ozen - Eskisehir Osmangazi University Faculty of Medicine, Department of Anesthesiology and Reanimation, Division of Intensive Care, Eskisehir, Turkey

**Objectives:** The aim of this study is to evaluate our treatment results in pregnant women presented with intoxication.

**Methods:** The patients admitted to intensive care unit (ICU) with intoxication between April 2010-May 2015 were evaluated retrospectively and 18 pregnant intoxication cases were achieved.

**Results:** Median age was 29 (22–40). Median pregnancy week was 15.5 (5–32). Eleven (61.1 %) patients were multi drug intoxication, 5 (27.8 %) patients were carbon monoxide (CO) intoxication and 2 patients were mushroom intoxication. The drugs that were taken in order to commit suicide were mostly antidepressants, antibiotics and analgesics. Median duration of stay in ICU was 1.5 (1–5) days. Median Glasgow Coma Score (GCS) was 15 (14–15). Additional comorbid diseases were positive in 4 (22.2 %) patients. Hyperbaric oxygen therapy (HBOT) was applied to all patients with CO intoxication. Median application number of HBOT was 1 (1–3). Activated carbon, proton pump inhibitors for gastric protection and medical treatment were given to all patients with multi drug intoxication. The 2 patients with mushroom intoxication received only symptomatic treatment.

When we compare the pre and post treatment AST, ALT and CK values of the patients with multi drug intoxication there were significant decrease. But, there were no differences between troponin values. In the patients with CO intoxication there were no significant differences between the pre and post treatment carboxyhemoglobin (COHb) values (Table 8).

All patients were discharged with healing. Seventeen of the patients had no problem related with their pregnancy and they gave birth at term (37–38 weeks). All babies were healthy at birth and they did not have any problem associated with intoxication. One of the patients ended her pregnancy with her own decision in her early pregnancy.

**Conclusions:** In conclusion, we did not observe any severe complication in clinically stable intoxication patients with normal laboratory findings in their pregnancy.

**Table 8 (abstract A457).** Comparison between pre and post treatment

		Pretreatment	Posttreatment	p
		Mean (Range)	Mean (Range)	
	AST	14.78 (11.00–20.20)	14.45 (9.00–20.00)	<0.001
Drug intoxication	ALT	9.76 (5.98–20.62)	9.00 (6.00–13.00)	<0.001
	CK	53.80 (33.00–85.00)	52.09 (30.00–120.00)	<0.001
	Troponin	0.03 (0.01–0.07)	0.016 (0.00–0.03)	NS
Carbon monoxide intoxication	COHb	14.25 (0.60–27.90)	2.70 (0.60–4.60)	NS

### A458 Alcohol-related major traumatic brain injury: a province-wide retrospective analysis

#### N. Kureshi^1,2^, L. Fenerty^1^, G. Thibault-Halman^1^, M. Erdogan^3^, S. Walling^1^, R.S. Green^2,3^, D.B. Clarke^1^

##### ^1^Dalhousie University, Queen Elizabeth II Health Sciences Centre, Division of Neurosurgery, Halifax, Canada; ^2^Dalhousie University, Critical Care, Halifax, Canada; ^3^Trauma Nova Scotia, Halifax, Canada

###### **Correspondence:** R.S. Green - Dalhousie University, Critical Care, Halifax, Canada

**Introduction:** Traumatic brain injury (TBI) is a leading cause of death and disability. In the Canadian province of Nova Scotia, TBI occurs in approximately 50 % of major trauma seen annually. Although alcohol use increases the risk of experiencing TBI, it remains unclear whether outcomes in alcohol-impaired TBI patients are different from those of unimpaired patients.

**Objectives:** The objective of this study was to describe the characteristics and patterns of major TBI seen over a 14-year period at the provincial level in Canada. We evaluated the effect of alcohol on length of stay (LOS) and mortality in patients with major TBI.

**Methods:** This was a retrospective case series. Data were obtained from the Nova Scotia Trauma Registry for all patients presenting with major TBI (abbreviated injury score [AIS] head ≥3) in Nova Scotia hospitals between 2002 and 2015. Injury rates were calculated on the basis of 100,000 population using population estimates from Statistics Canada. Patients were compared by blood alcohol concentration (BAC) at time of injury: negative (0–1.9 mmol/L), low (2–21 mmol/L), and moderate/high (≥22 mmol/L). A logistic regression model was constructed to test for outcomes and adjusted for the effects of age, gender, location, injury severity score (ISS), maximum AIS head, and BAC level.

**Results:** Overall, 4518 major TBI patients were seen in provincial hospitals during the study period. The mean age of TBI patients was 51 ± 25 years; 72 % were male. The majority of injuries were the result of blunt trauma (93 %), with relatively few major TBIs resulting from penetrating trauma (7 %). The most common mechanisms of injury were falls (46 %) and motor vehicle crashes (27 %). Analysis of census-based subpopulations demonstrated that injury rates varied significantly by geography (from 25 to 65 TBIs per 100,000 population). Testing for alcohol was performed in 43 % of cases. Patients who were tested for alcohol tended to be male (79 %) and middle-aged (mean age 44 ± 20 years). A positive BAC (i.e., ≥2 mmol/L) was found in 47 % of patients who were tested. Mean acute LOS was similar for all three BAC groups. Mortality was independently predicted by increasing age (odds ratio [OR] = 1.01; p < 0.001), male gender (OR = 1.30; p = 0.049), an ISS between 16 and 75 (OR = 2.67; p < 0.001), a maximum AIS head of 5 or 6 (OR = 3.21; p < 0.001), injuries occurring outside of the capital city of Halifax (OR = 1.70; p < 0.001), and having a lower BAC level (OR = 0.99; p < 0.001).

**Conclusions:** The results of this study show significant regional variation in major TBI rates in the province of Nova Scotia. Our findings suggest that low BAC levels are associated with increased mortality in major TBI patients. There are ongoing needs for prevention and intervention efforts that focus on unintentional falls and motor vehicle crashes, especially in older adults. Further study is warranted to elucidate the mechanisms underlying the effects of alcohol on outcomes in patients with major TBI.

### A459 N-acetylcysteine ameliorates liver injury in a rat model of intestinal ischemia-reperfusion

#### P. Briassoulis^1^, K. Kalimeris^1^, A. Ntzouvani^2^, T. Nomikos^2^, K. Papaparaskeva^3^, E. Politi^4^, G. Kostopanagiotou^1^

##### ^1^Attikon University Hospital, 2nd Department of Anaesthesiology, Athens, Greece; ^2^Harokopeio University, Department of Nutrition and Dietetics, School of Health Science and Education, Athens, Greece; ^3^'Agia Olga' Hospital, Laboratory of Pathology, Athens, Greece; ^4^Aretaieion University Hospital, Laboratory of Cytology, Athens, Greece

###### **Correspondence:** P. Briassoulis - Attikon University Hospital, 2nd Department of Anaesthesiology, Athens, Greece

**Introduction:** N-acetylcysteine (NAC) is an antioxidant with direct and indirect antioxidant actions used in the clinical setting. Oxidative stress is known to play a pivotal role in the intestinal ischemia-reperfusion (IIR).

**Objectives:** Therefore we studied the effect of different pretreatment regimens with NAC on the IIR injury in rats.

**Methods:** Thirty-five male Wistar rats were randomly assigned to 5 groups. In group SHAM only laparotomy was performed. Group CONTROL underwent IIR without NAC. In the other groups, NAC was administered intraperitoneally with different regimens: 150 mg/kg before ischemia (NAC150), 300 mg/kg before ischemia (NAC300) and 150 mg/kg before ischemia plus 150 mg/kg 5 min before reperfusion (NAC150 + 150). Measurements in tissues and blood were conducted at 4 hours of reperfusion following exsanguination.

**Results:** Histological score of the liver was significantly improved in NAC300 compared with control (1.7 ± 0.5 vs. 2.9 ± 1.1 respectively, p = 0.05). In addition, NAC treatment significantly reduced liver transaminases in all groups of treatment, mostly in group NAC300. Plasma malondialdehyde levels were lower with NAC treatment, although not statistically significantly. Lung glutathione peroxidase was significantly increased in group NAC300 (p = 0.04), while the other oxidation biomarkers showed no significant differences.

**Conclusions:** NAC exerts a significant protective role in liver injury following IIR, which seems to be independent of an intestinal protective effect. Additional administration of NAC before reperfusion was of no further benefit. The most effective regimen among the compared regimens was that of 300 mg/kg before ischemia.

**Keywords:** intestinal ischemia-reperfusion, n-acetylcysteine, oxidative stress, liver injury, rat model

## Oral Sessions: Tuesday, 4 October 2016

### Abstract award winning session

#### A460 Pre-hospital emergency anaesthesia in awake hypotensive trauma patients: life saving or detrimental?

##### K. Crewdson, M. Rehn, A. Weaver, K. Brohi, D. Lockey

###### London's Air Ambulance, Pre-hospital Emergency Medicine, London, UK

####### **Correspondence:** K. Crewdson - London's Air Ambulance, Pre-hospital Emergency Medicine, London, UK

**Introduction:** The benefits of pre-hospital emergency anaesthesia (PHEA) are controversial. The timing of this intervention can be a difficult decision, particularly for conscious trauma patients with significant hypotension secondary to presumed hypovolaemia. Patients who are hypovolaemic prior to induction of anaesthesia are at risk of severe cardiovascular instability post induction, which may be difficult to manage.

**Objectives:** The aim of this study was to compare the mortality rate for severely injured hypovolaemic trauma patients (without evidence of major neurological injury) undergoing PHEA with a patient cohort with the same physiology who were transported to hospital without PHEA.

**Methods:** A five year retrospective database review of a physician-led pre-hospital trauma service was performed to identify trauma patients who were hypotensive (systolic blood pressure < 90 mmHg) on scene with a GCS between 13 and 15 between 01/09/2009 and 31/08/2014. Pre-hospital records were consulted independently by two pre-hospital clinicians to determine the likelihood of hypovolaemia based on clinical factors.

The primary outcome measure was mortality, defined as death before hospital discharge. The secondary outcome measure was mortality for patients hypotensive on scene secondary to presumed hypovolaemia.

**Results:** In total, 9480 patients were attended in the study period; 236 patients were included in the final analysis. Of these, 101 patients received PHEA on scene and 135 did not. Fifteen patients who underwent PHEA on scene died (14.9 %) compared with six patients (4.4 %) in the group of patients who were not intubated on scene, p = 0.01. The unadjusted odds ratio for death using the exact logistic regression model was 3.763 (1.30-12.21) p = 0.01. This increased to 5.96 (95 % CI 1.76-20.17) p = 0.004 after adjustment for age, mechanism of injury and initial heart rate. Subgroup analysis identified a statistically significant increase in mortality for hypovolaemic trauma patients who underwent PHEA. In the group of 101 patients anaesthetised on scene, 58 patients (57.4 %) were considered to be hypovolaemic prior to induction of anaesthesia, 14 died (24 %). In the group of 43 patients (42.6 %) intubated on scene not meeting criteria for hypovolaemia, one patient died (2 %), p = 0.003. After adjustment for age, mechanism of injury and initial heart rate the odds ratio for mortality was 9.99 (95 % CI 1.69-58.98) p = 0.01.

**Conclusions:** Our results show an association between PHEA and mortality in awake hypotensive trauma patients, which is increased when hypotension is confirmed to be due to hypovolaemia. This study supports the hypothesis that where patients are hypovolaemic and awake on scene it might, where possible, be appropriate to delay induction of anaesthesia until after hospital arrival.

#### A461 The extra physiotherapy in critical care (EPICC) multi-centre randomised controlled trial

##### S. Wright^1^, K. Thomas^1^, C. Baker^1^, L. Mansfield^1^, V. Stafford^1^, C. Wade^1^, G. Watson^2^, A. Bryant^3^, T. Chadwick^3^, J. Shen^3^, J. Wilkinson^2^, J. Furneval^4^, A. Henderson^4^, K. Hugill^5^, P. Howard^5^, A. Roy^4^, S. Bonner^5^, S. Baudouin^1^

###### ^1^Newcastle upon Tyne Hospitals NHS Foundation Trust, Perioperative and Critical Care, Newcastle upon Tyne, UK; ^2^Newcastle University, Clinical Trials Unit, Newcastle upon Tyne, UK; ^3^Newcastle University, Institute of Health & Society, Newcastle upon Tyne, UK; ^4^City Hospitals Sunderland NHS Foundation Trust, Anaesthesia, Sunderland, UK; ^5^South Tees Hospitals NHS Foundation Trust, Anaesthesia, Middlesborough, UK

####### **Correspondence:** S. Baudouin - Newcastle upon Tyne Hospitals NHS Foundation Trust, Perioperative and Critical Care, Newcastle upon Tyne, UK

**Introduction:** Early Mobilisation Therapy (EMT) may improve outcome in the critically ill but the evidence is very limited ^1^. We have conducted a multi-centre randomised controlled trial (RCT) comparing two levels of EMT.

**Objectives:** To test the hypothesis that more intensive EMT will improve longer term physical outcome in the critically ill.

**Methods:** A full trial protocol has been published^2^. We conducted a 3 centre RCT between January 2012 to July 2015. Eligible patients had received at least 48 hours of invasive ventilation before recruitment. The Intervention arm received up to 90 min per day of EMT Monday to Friday whilst the control arm received up to 30 min per day Monday to Friday. The primary trial outcome was the physical component of the SF-36 quality of life measure at 6 months after recruitment.

**Results:** 308 patients were recruited (150 assigned to the intervention arm and 158 to control). The groups were well matched in terms of age, gender, pre-morbid function, illness severity, type of admission and BMI. Between enrollment and final follow-up at 6 months 94 (30.5 %) participants died. Of the remaining 214 participants 116 completed and returned the SF-36 at 6 months. There was no significant difference in baseline demographics between survivors who completed the SF-36 and those who failed to respond. Full survival data were available for all participants via a national database. The total number of EMT session delivered to the Intervention group was higher than the standard group (2061 v 1326) and the time delivered was correspondingly higher (456.2 v 255.8 hours in total). Physical health as measured by the SF-36 Physical Component score improved in both groups over the 6 month follow up period with no significant difference at 6 months (37.0 Intervention v 37.1 Control; difference in means and 95 % CI 0.3 [−6.1 to 6.7]). There was no significant difference in any of the secondary measures or in 6 month mortality (Intervention 28.7 % standard 34.4 % Cox proportional hazard ratio 1:00 [95 % CI 0.60-1.68]).

**Conclusions:** Intensive EMT did not improve the recovery or outcome at 6 months of critically ill patients ventilated for at least 48 hours compared to standard EMT.

**References**

1. Hashem MD et al. Early Mobilization and Rehabilitation of the Critically Ill Patient. *Chest* 2016: S0012-3692(16)41641-7

2. Thomas K et al. Extra Physiotherapy in Critical Care (EPICC) Trial Protocol: a randomised controlled trial of intensive versus standard physical rehabilitation therapy in the critically ill. *BMJ Open* 2015;5(5):e008035.

**Grant acknowledgement**

This abstract presents independent research funded by the National Institute for Health Research (NIHR) under its Research for Patient Benefit (RfPB) Programme (Grant Reference Number **PB-PG-0909-20021**). The views expressed are those of the author(s) and not necessarily those of the NHS, the NIHR or the Department of Health.

#### A462 Colonization, nosocomial infection and antibiotic consumption after four years application of selective digestive decontamination in an intensive care unit in a university hospital

##### C. Sánchez Ramírez^1^, S. Hípola Escalada^1^, M.A. Hernández Viera^1^, M. Cabrera Santana^1^, L. Caipe Balcázar^1^, N. Sangil Monroy^2^, F. Artiles Campelo^3^, C.F. Lübbe Vázquez^1^, P. Saavedra Santana^4^, S. Ruiz Santana^1^

###### ^1^University Hospital of Gran Canaria Dr. Negrín, Intensive Care Unit, Las Palmas de Gran Canaria, Spain; ^2^University Hospital of Gran Canaria Dr. Negrín, Pharmacy Department, Las Palmas de Gran Canaria, Spain; ^3^University Hospital of Gran Canaria Dr. Negrín, Microbiology Department, Las Palmas de Gran Canaria, Spain; ^4^University of Las Palmas de Gran Canaria, Mathematics and Informatics Deparment, Las Palmas de Gran Canaria, Spain

####### **Correspondence:** C. Sánchez Ramírez - University Hospital of Gran Canaria Dr. Negrín, Intensive Care Unit, Las Palmas de Gran Canaria, Spain

**Objectives:** To prospectively evaluate the impact of Selective Digestive Decontamination (SDD) application on nosocomial infections and colonization rates after 4 years in an ICU.

**Methods:** This study was conducted in a 30-bed-medical-surgical ICU. All consecutive patients admitted to the ICU from October 1, 2011 to September 30, 2015 expected to require tracheal intubation > 48 hours were given SDD (SDD study group) with a 4-day course of intravenous cefotaxime, plus enteral colistin, tobramycin, nystatin in an oropharyngeal paste and in a digestive solution. Oropharyngeal and rectal swabs were obtained on admission and once weekly. We used ENVIN nosocomial infection criteria. We compared all patients admitted to ICU who acquired nosocomial ICU infections from October 1, 2010 to September 30, 2011 (non-SDD group) to the SDD study group. In both groups, categorical variables were summarized as frequencies and percentages and the continuous ones as means and standard deviations (SD) when the data followed the normal distribution or medians and interquartile ranges (IQR) when they did not. The percentages were compared using the test of chi-square test or Fisher exact test, means with the *t*-test and medians with the Wilcoxon test for independent samples. Those variables that showed statistical significance in the univariate analysis were introduced in a multivariate logistic regression analysis. For each one of the acquired infections (catheter-related and other secondary bacteremias, pneumonia and urinary infections and antibiotic resistant bacteria (ARB) infections) the incidences per 1000 days of exposure in each cohort and the corresponding relative risks were obtained using the Poisson regression. Statistical significance was set at *p* ≤ 0.05. We analyzed colistin and tobramycin resistant colonization and also antibiotic consumption (Defined antibiotics Daily Doses (DDD).

**Fig. 18 (abstract A462).**
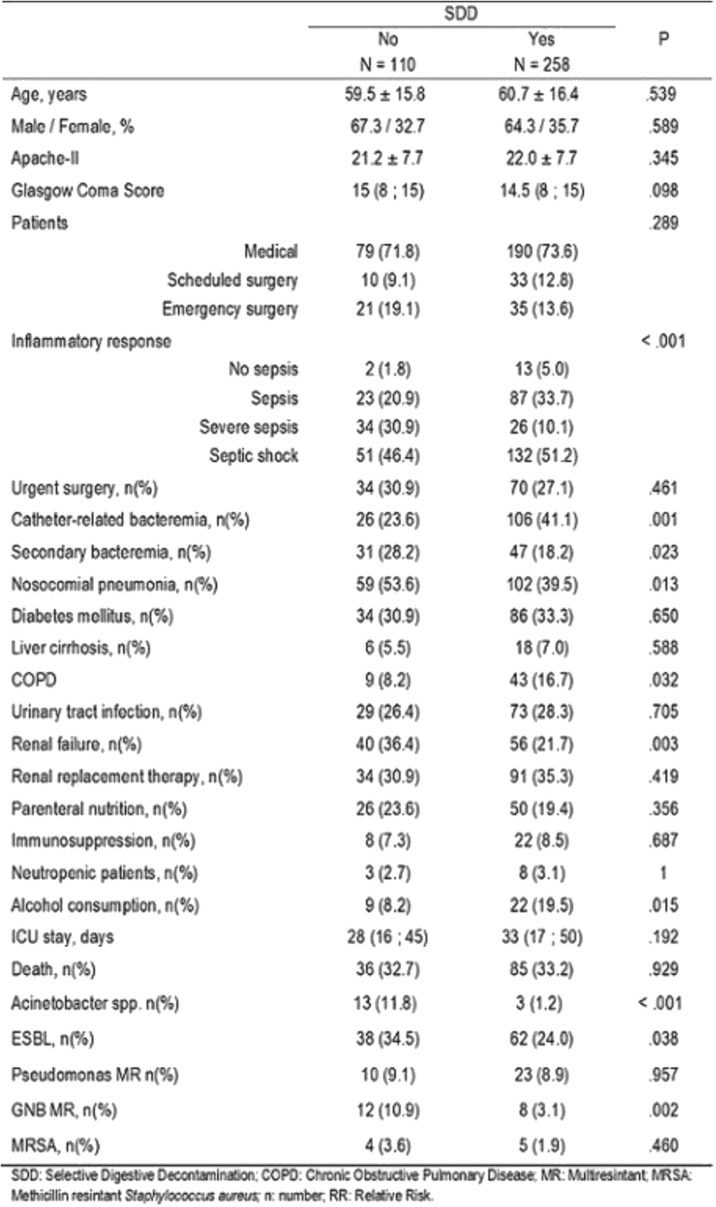
Univariate analysis

**Fig. 19 (abstract A462).**
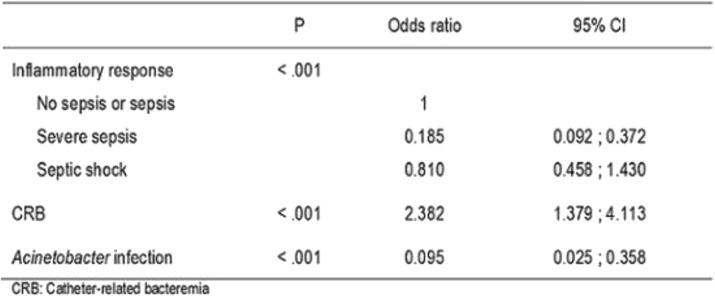
Multivariate logistic regression analysis

**Fig. 20 (abstract A462).**
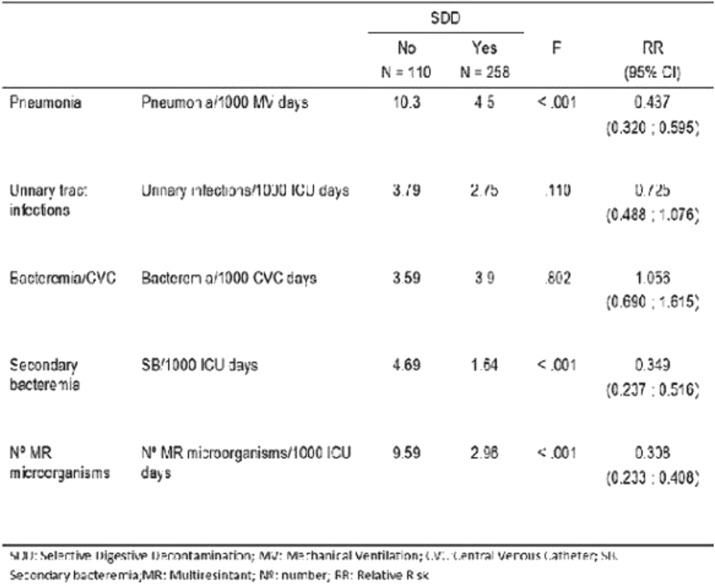
Nosocomial infection rates

**Results:** Results are shown in Figs. 18, 19 and 20.

There were no statistical significant differences between both groups in type of ICU admission or demographic data. Patients with SDD had significantly less Extended Spectrum Betalactamase (ESBL), Gram Negative Bacteria Multirresistant (GNB MR) and *Acinetobacter spp* infections. We had also a significant reduction in nosocomial pneumonias and other secondary bacteremias and ARB rates in SDD group versus non SDD. There was no infection by Clostridium difficile. The exogenous infections were 76,08 %. Colistin resistant colonization was 14,1 % and tobramycin resistant colonization was 20,7 % of samples. There was a decrease on the DDD/100 ICU stays after SDD.

**Conclusions:** After 4 years applying SDD a significant reduction of infections by ESBL, GNB MR and *Acinetobacter* was observed. A significant decrease of nosocomial pneumonia, secondary bacteremias and ARB infections rates was also shown. An antibiotic consumption reduction was found after SDD. Low rates of colistin and tobramycin resistant colonization bacteria have been observed.

#### A463 Hypertonic lactate improves cerebral energy metabolism and brain perfusion in critically ill patients with aneurysmal subarachnoid hemorrhage

##### L. Carteron^1^, C. Patet^1^, H. Quintard^2^, D. Solari^1^, P. Bouzat^3^, M. Oddo^1^

###### ^1^CHUV, Department of Intensive Care Medicine, Neuroscience Critical Care Research Group, Lausanne, Switzerland; ^2^University Hospital, Department of Anesthesia and Intensive Care Medicine, Nice, France; ^3^University Hospital, Department of Anesthesia and Intensive Care Medicine, Grenoble, France

####### **Correspondence:** L. Carteron - CHUV, Department of Intensive Care Medicine, Neuroscience Critical Care Research Group, Lausanne, Switzerland

**Introduction:** Lactate supports brain energy metabolism and promotes cerebral vasodilation. Hypertonic lactate (HL) solutions are emerging as a therapeutic alternative after acute brain injury however their effect following aneurysmal subarachnoid hemorrhage (SAH) is unknown.

**Methods:** We prospectively studied critically ill SAH patients (n = 7, age 64 ± 6 years) monitored with cerebral microdialysis (CMD) and transcranial Doppler (TCD) who were resuscitated according to current guidelines and had no intracranial hypertension (clinicaltrial.gov NCT01573507). Intervention consisted of a 3-h infusion of HL (30 μmol/kg/min, administered within 48 h from SAH, to achieve blood arterial lactate ≈ 4–5 mmol/L). Endpoints were the effect of HL on cerebral energy metabolism (using hourly CMD concentrations of lactate, pyruvate and glucose) and brain perfusion (using hourly TCD measurement of middle cerebral artery cerebral blood flow velocities, MCBFV).

**Results:** Treatment with HL was associated with a significant increase of cerebral extracellular lactate (3.6 ± 1.6 vs. 5.5 ± 1.5 mmol/L), pyruvate (118.5 ± 51.1 vs. 166.4 ± 47.5 μmol/L) and glucose (1.1 ± 0.2 vs. 1.6 ± 0.3 mmol/L; all *p* < 0.01, paired *t* test for comparisons between baseline and peak values during HL). HL therapy was also associated with improved brain perfusion (MCBFV 61.8 ± 28.5 vs. 79.7 ± 35.3 cm/sec; *p* < 0.05), while cerebral perfusion pressure (79 ± 16 vs. 83 ± 13 mmHg) and PbtO_2_ (20 ± 7 vs. 20 ± 7 mmHg) did not change significantly.

**Conclusions:** Our findings show that HL administered during the early post-injury phase following SAH improves brain energy metabolism by exerting a beneficial cerebral glucose sparing effect. HL also increases brain perfusion, which seems independent from cerebral perfusion pressure and PbtO_2_, and appears related to cerebral extracellular lactate supplementation. These preliminary data support larger studies to examine the value of HL solutions in patients with SAH and other forms of acute cerebrovascular diseases.

**Grants**

Supported by research grants from the Swiss National Science Foundation (SNSF), the Novartis Foundation for Biomedical Research, the Société Française d'Anesthésie et de Réanimation (SFAR) and the “Fondation des Gueules Cassées”.

#### A464 Randomized controlled trial using daily protocol based physiotherapy or protocol based physiotherapy with additional electrical muscle stimulation (EMS) in critically ill patients to prevent intensive care unit (ICU) acquired weakness (ICUAW)

##### T. Wollersheim^1,2^, J. Malleike^1^, K. Haas^1^, N. Carbon^1^, J. Schneider^1,2^, C. Birchmeier^3^, J. Fielitz^4,5^, S. Spuler^1,4^, S. Weber-Carstens^1,2^

###### ^1^Charité - Universitaetsmedizin Berlin, Berlin, Germany; ^2^Berlin Institute of Health (BIH), Berlin, Germany; ^3^Max Delbrück Center for Molecular Medicine (MDC), Berlin, Germany; ^4^ECRC - Experimental and Clinical Research Center, Berlin, Germany; ^5^Immanuel Hospital Bernau, Bernau, Germany

####### **Correspondence:** T. Wollersheim - Charité - Universitaetsmedizin Berlin, Berlin, Germany

**Introduction:** Intensive care unit acquired weakness (ICUAW) is a severe complication of critical illness. ICUAW is associated with muscle wasting, muscle weakness, respiratory failure as well as increased morbidity and mortality and features massive skeletal muscle atrophy by destruction of the contractile myosin elements. No specific treatment has been established.

**Objectives:** Aim of the study was to investigate if protocol-based

physiotherapy (pPT) or pPT with additional evoked muscle contraction by electrical muscle stimulation (EMS) are more effective to prevent skeletal muscle atrophy in critically ill patients when compared to standard physiotherapy (sPT).

**Methods:** A randomized clinical trial was performed. Participants (inclusion criterion: SOFA score ≥ 9) were randomly split into 2 groups, either treated started at the day of admission with sole pPT or pPT with EMS daily. Data of patients treated with standard physiotherapy (sPT) were available from an earlier trial[1]. An open surgical muscle biopsy from the *musculus vastus lateralis* was obtained at median day 15 of critical illness. Histological examination of muscle biopsies and quantification of myocyte cross-sectional area (MCSA) for type I, IIa, and IIb fibers using ImageJ software (fiber count > 100 per patient) were performed after applying the toluidine blue ATPase Method: Non-parametric tests were performed. Ethic vote (Charité EA 2/041/10).

**Results:** 39 critical ill patients were enrolled in this final analysis (19sPT, 11pPT, 9 pPT + EMS). There were no significant differences in baseline characteristics and fiber type distribution between the groups. The MCSA for all fiber types of patients with pPT with or without additional EMS were larger than the MCSA of patients with sPT. Compared to pPT alone, additional evoked muscle activation by EMS increases MCSA for type I,IIa and IIb muscle fibers. Data are shown as frequency-distribution histograms.

**Conclusions:** For the first time we show that pPT is associated with reduced skeletal muscle atrophy when compared to sPT. EMS resulted in an additional reduction of muscle atrophy. The histological findings showed reduced muscle atrophy through preventive physiotherapy procedures. Further studies are needed to solidify our findings and to analyze if this reduction in atrophic response can be confirmed on molecular (i.e. analysis of myosin heavy chain, protein homeostasis) and functional levels and improves the long-term outcome of critical ill patients.

**References**

**1.** Wollersheim et al. (2014) Dynamics of myosin degradation in intensive care unit-acquired weakness during severe critical illness. Intensive Care Med.

**GRANT ACKNOWLEDGEMENT**

Berlin Institute of Health (BIH), TRG3 and Clinical Scientist, DFG KFO 192/2 TP3.

**Fig. 21 (abstract A464).**
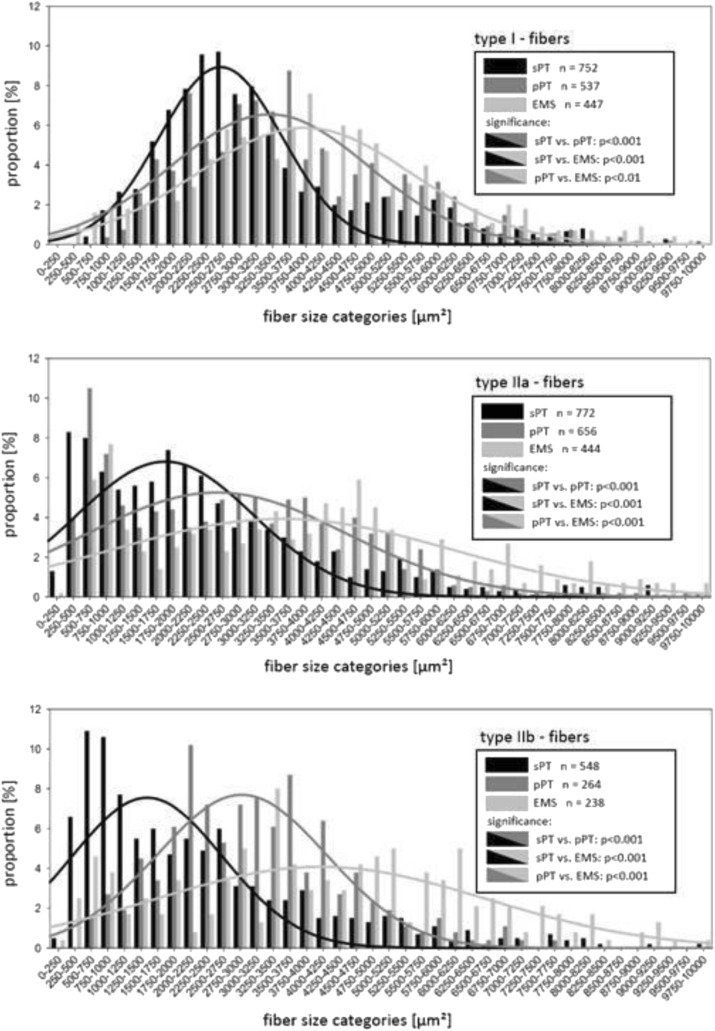
[MCSA]

### Haemodynamic monitoring

#### A465 Central venous-to-arterial carbon dioxide difference and thehaldane effect: a limiting factor, or an additional marker of severity in shock?

##### L. Enseñat^1^, A. Pérez-Madrigal^1^, P. Saludes^1^, L. Proença^1,2^, G. Gruartmoner^1^, C. Espinal^1^, J. Mesquida^1^

###### ^1^Corporació Sanitària i Universitaria Parc Taulí, Universitat Autònoma de Barcelona, Critical Care Department, Sabadell, Spain; ^2^Hospital Prof. Dr. Fernando Fonseca, Serviço de Medicina I, Amadora, Portugal

####### **Correspondence:** L. Enseñat - Corporació Sanitària i Universitaria Parc Taulí, Universitat Autònoma de Barcelona, Critical Care Department, Sabadell, Spain

**Objective:** Central venous-to-arterial carbon dioxide difference (P_cva_CO_2_) has demonstrated its prognostic value in critically ill patients suffering from shock, and current expert recommendations advocate for further resuscitation interventions when P_cva_CO_2_ is elevated. P_cva_CO_2_combination with arterial-venous oxygen content difference (P_cva_CO_2_/C_av_O_2_) seems to enhance its performance when assessing anaerobic metabolism. However, the fact that PCO_2_ values might be altered by changes in blood O_2_content (the Haldane effect), has been presented as a limitation of PCO_2_-derived variables. The present study aimed at exploring the impact of the Haldane effect on P_cva_CO_2_ and P_cva_CO_2_/C_av_O_2_ during the early phase of shock.

**Methods:** Prospective interventional study. Ventilated patients suffering from shock within the first 24 hours of ICU admission. Patients requiring FiO_2_ ≥ 0.5 were excluded. At inclusion, simultaneous arterial and central venous blood samples were collected. Patients underwent ahyperoxygenation test (5 minutes of FiO_2_ 100 %), and arterial and central venous blood samples were repeated. Oxygenation and CO_2_ variables were calculated at both timepoints.

**Results:** Twenty patients were studied. The main cause of shock was septic shock (70 %). The hyperoxygenation trial increased oxygenation parameters in arterial and venous blood, whereas PCO_2_ only changed at the venous site. Resulting P_cva_CO_2_ and P_cva_CO_2_/C_av_O_2_significantly increased (6.8 (4.9, 8.1) vs 7.6 (6.7, 8.5) mmHg, p 0.001; and 1.9 (1.4, 2.2) vs 2.3 (1.8, 3), p < 0.001, respectively). Baseline P_cva_CO_2_, P_cva_CO_2_/C_av_O_2_ and S_cv_O_2_ correlated with the magnitude of PO_2_ augmentation at the venous site within the trial(rho −0.46, p 0.04; rho 0.6, p < 0.01; andrho 0.7, p < 0.001, respectively). Increased P_cva_CO_2_/C_av_O_2_values were associated with higher mortality in our sample (1.46 (1.21, 1.89) survivorsvs2.23 (1.86, 2.8) non-survivors, p < 0.01).

**Conclusions:** P_cva_CO_2_ and P_cva_CO_2_/C_av_O_2_ are influenced by oxygenation changes not related to flow. Elevated P_cva_CO_2_ and P_cva_CO_2_/C_av_O_2_ values might not only derive from cardiac output inadequacy, but also from venous hyperoxia. Elevated P_cva_CO_2_/C_av_O_2_ values were associated with higher PO_2_ transmission to the venous compartment, suggesting higher shunting phenomena. Rather than a limitation, the contribution of the Haldane effect to CO_2_-derived variables might represent an additional marker of severity of shock.

**References**

1.- Mesquida J, et al. Central venous-to-arterial carbon dioxide difference combined with arterial-to-venous oxygen content difference is associated with lactate evolution in the hemodynamic resuscitation process in early septic shock. Crit Care 2015; 19:126

2.- Mekontso-Dessap et al. Combination of venoarterial PCO2 difference with arteriovenous O2 content difference to detect anaerobic metabolism in patients. Intensive Care Med 2002;28:272–277

#### A466 Pulmonary vascular permeability index and global ejection fraction: are the data appropriately corrected in case of femoral indicator injection for transpulmonary thermodilution?

##### W. Huber, M. Eckmann, F. Elkmann, A. Gruber, T. Lahmer, U. Mayr, A. Herner, R. Schellnegger, J. Schneider, R.M. Schmid

###### Technische Universität München, II. Medizinische Klinik, Munich, Germany

####### **Correspondence:** W. Huber - Technische Universität München, II. Medizinische Klinik, Munich, Germany

**Introduction:** Transpulmonary thermodilution (TPTD) has been established to measure cardiac output CO, global end-diastolic volume GEDV and extravascular lung water EVLW. To facilitate interpretation of these data several ratios have been developed, including pulmonary vascular permeability index (defined as EVLW/(0.25*GEDV)) and global ejection fraction ((4*stroke volume)/GEDV). PVPI and GEF have been associated to the aetiology of pulmonary oedema and to systolic cardiac function, respectively. However, several studies showed that the use of *femoral* venous access results in a marked overestimation of GEDV which also falsely reduces PVPI and GEF. One of these studies suggested a correction formula for femoral venous access that markedly reduces the bias for GEDV (1). Consequently, the last PiCCO-algorithm requires information about the CVC, and correction for femoral access has been shown. However, two recent studies demonstrated inconsistencies of this algorithm using incorrected GEDV for PVPI (2), but corrected GEDV for GEF. Nevertheless, these studies were based on mathematical analysis of data displayed by the PiCCO in a total of 15 patients equipped with only a *femoral*, but not with a *jugular* CVC.

**Objectives:** To compare PVPI_fem and GEF_fem derived from femoral TPTD to values derived from jugular indicator injection in 25 patients with both jugular and femoral CVCs.

**Methods:** 54 datasets in 25 patients CVC were recorded. Each dataset consisted of three triplicate TPTDs using the jugular venous access as the gold standard, while the the femoral access with (PVPI_fem_cor) and without (PVPI_fem_uncor) information about the femoral injection site was used to evaluate, if correction for femoral GEDV pertains to PVPI_fem and GEF_fem.

**Results:** 15 male, 10 female patients; 60 ± 15 years; 79 ± 16 kg; 173 ± 8 cm. PVPI_fem_uncor was significantly lower than PVPI_jug (1.48 ± 0.47 vs. 1.84 ± 0.53; p < 0.001). Similarly, PVPI_fem_cor was significantly lower than PVPI_jug (1.49 ± 0.46 vs. 1.84 ± 0.53; p < 0.001). This is explained by the finding that PVPI_fem_uncor was not different to PVPI_fem_cor (1.48 ± 0.47 vs. 1.49 ± 0.46; n.s.). This clearly suggests that correction for femoral CVC does not pertain to PVPI. GEF_fem_uncor was significantly lower than GEF_jug (20.6 ± 5.1 % vs. 25.0 ± 6.1 %; p < 0.001). By contrast, GEF_fem_cor was not different to GEF_jug (25.6 ± 5.8 % vs. 25.0 ± 6.1 %; n.s.). Furthermore, GEF_fem_cor was significantly higher than GEF_fem_uncor (25.6 ± 5.8 % vs. 20.6 ± 5.1 %; p < 0.001). This emphasizes that an appropriate correction for femoral CVC is applied to GEF_fem_cor.

**Conclusions:** Uncorrected femoral injection for TPTD results in significantly lower values for PVPI and GEF. While the last PiCCO algorithm appropriately corrects for GEF, the correction is not applied to PVPI. This results in substantial underestimation of PVPI.

**References**

1. Saugel, Huber et al., Crit Care 2010; 14(3):R95;

2. Berbara, Huber et al. BMC Anesthesiol. 2014

#### A467 Echocardiographic assessment of fluid responsiveness in spontaneously breathing patients with hemodynamic instability: anything new?

##### W. Ayoub^1^, W. Samy^1^, A. Esmat^2^, A. Battah^3^, S. Mukhtar^3^

###### ^1^Cairo University Medical School, Intensive Care Department, Cairo, Egypt; ^2^Faculty of Medicine, Fayoum University, Intensive Care Department, Fayoum, Egypt; ^3^Cairo University Medical School, Intensive care Department, Cairo, Egypt

####### **Correspondence:** W. Ayoub - Cairo University Medical School, Intensive Care Department, Cairo, Egypt

**Introduction:** Prediction of fluid responsiveness in hemodynamically unstable patients with spontaneous breathing activity has been a clinical challenge. It has been best assessed by passive leg raising test. Pre-ejection period, the time from the onset of ventricular depolarization to the beginning of left ventricular ejection, is a systolic time interval found to decrease with greater preload^1^. The effect of passive leg raising test on the pre-ejection period has not been studied in this context.

**Objectives:** Our objective was to test whether fluid responsiveness could be predicted by the response of pre-ejection period to passive leg raising test. We also examined whether baseline end expiratory inferior vena cava diameter could predict fluid responsiveness in this category of patients.

**Methods:** Thirty patients with spontaneous breathing activity considered for fluid loading were included. We used transthoracic echocardiography to measure stroke volume, and pre-ejection period before and during passive leg raising test as well as before and after fluid loading (500 ml saline 0.9 % over 15 minutes). An increase in stroke volume of 15 % or more after volume expansion defined fluid responders. We also measured baseline end expiratory inferior vena cava diameter obtained from the subcostal window.

**Results:** 19 patients were responders (63.3 %). Passive leg raising test induced-changes in stroke volume of ≥ 9.3 % predicted fluid responsiveness with a sensitivity of 100 % and specificity of 81.8 %, the area under receiver operating characteristic curve (AUC) was 0.96; 95 % confidence interval (CI) [0.91,1.0], meanwhile, passive leg raising test-induced changes in pre-ejection period of ≤ −5.0 % predicted fluid responsiveness with a sensitivity of 94.7 % and a specificity of 45.5 %, the AUC was 0.62; 95 % CI [0.4,0.85]. Baseline inferior vena cava diameter (in cm) failed to identify responders vs. non-responders (1.20 ± 0.37 vs 1.38 ± 0.51 respectively, p = 0.36).

**Conclusions:** In hemodynamically unstable patients with spontaneous breathing activity, passive leg raising test-induced increase in stroke volume of ≥ 9.3 % accurately predicted fluid responsiveness, while passive leg raising test-induced decrease in pre-ejection period of ≤ −5.0 % was sensitive, but not specific in the prediction of fluid responsiveness. Baseline inferior vena cava diameter failed to identify fluid responders.

**References**

1- Weissler AM. Current concepts in cardiology. Systolic-time intervals. N Engl J Med 296: 321–324, 1977

#### A468 Measurement of cutaneous blood flow using a laser doppler technique in patients with shock

##### W. Mongkolpun, D. Orbegozo Cortés, C.P.R. Cordeiro, J.-L. Vincent, J. Creteur

###### University Hospital Erasme, Université Libre de Bruxelles, Critical Care, Brussels, Belgium

####### **Correspondence:** W. Mongkolpun - University Hospital Erasme, Université Libre de Bruxelles, Critical Care, Brussels, Belgium

**Introduction:** Persistent signs of skin hypoperfusion after resuscitation in patients with shock are associated with worse outcomes, but assessment is rather subjective. Skin laser Doppler (SLD) techniques allow skin blood flow (SBF) to be measured more objectively and non-invasively.

**Objective:** To measure SBF at the extremities using a SLD technique in patients with shock and to determine if they are correlated to patients outcomes.

**Method:** SBF was evaluated in the fingertip and toe (PU: perfusion units) using a SLD (PeriFlux System 5000, Perimed, Sweden) at basal skin temperature and after warming to 37 °C in 28 consecutive patients admitted with circulatory shock (need for vasoactive agents to maintain MAP > 60 mmHg) on ICU admission and 10 healthy volunteers. Organ dysfunction was assessed using the sequential organ failure assessment (SOFA) score. Statistical analysis was performed using STATA 13.0. The area under the receiver operating curve (AUC) was calculated to identify the predictive performance of SBF. Results are presented as median (p25-75) values.

**Result:** Global ICU mortality was 32 %. Demographic data and SBF are shown in Table 9. The AUCs for prediction of mortality at basal temperature were 0.77 (0.58-0.97) and 0.65 (0.47-0.92) for the fingertip and toe respectively and 0.71 (0.51-0.93) for the SOFA score.

**Conclusion:** Measurements of SLD at fingertip and toe may represent an interesting monitoring technique for shock patients. Their prognostic value is similar to SOFA score.

**Table 9 (abstract A468).** Demograpic data

	Survivors(n=19)	Non survivors (n=9)	P	All (n=28)	Volunteer (n=10)	P
SOFA score	10(8–11)	12(9–13)	0.06	11(9–12)	-	-
Age (years)	63 (55–57)	58 (41–68)	0.20	62 (54–72)	30(36–31)	<0.01
Mean arterial pressure (mmHg)	69 (67–86)	67 (62–84)	0.40	69 (65–85)	-	-
Cardiac index (L/min/m2)	2.7 (2.4–3.2)	2.4 (1.6–4.4)	0.60	2.6 (2.1–4.4)	-	-
Noradrenaline (mcg/Kg/min)	0.16 (0.10–0.26)	0.50 (0.09–0.80)	0.16	0.18(0.10–0.45)	-	-
Lactate (mmol/L)	1.4 (1.1–1.7)	3.8 (1.6–4.9)	0.01	1.6 (1.2–2.8)	-	-
Shock Types						
•Septic, n	14	7	0.29	21	-	-
•Cardiogenic,n	5	1	-	6	-	-
•Haemorrhagic,n	0	1	-	1	-	-

**Table 10 (abstract A468).** SBF in Patients and Volunteers

	Survivors(n=19)	Non survivors (n=9)	P	All (n=28)	Volunteer (n=10)	P
Fingertip SBF at basal temperature (PU)	31.4 (11.1–49.4)	9.8 (7.8–12.3)	0.02	20.5(9.8–38.7)	175.7(196.5–218.0)	<0.01
Fingertip SBF at 37°C(PU)	60.9(21.2–70.1)	17.2(13.9–49.0)	0.09	49.7(15.8–70.0)	274.8(201.2–320.1)	<0.01
Toe SBF at basal temperature (PU)	32.5(8.7–44.7)	15.5(6.1–21.9)	0.20	16.3(8.2–35.8)	49.0(44.6–55.1)	0.02
Toe SBF at 37°C(PU)	39.2(19.5–72.9)	20.5(9.9–36.1)	0.04	30.7(17.3–52.7)	116.7(99.9–122.2)	<0.01

#### A469 Role of dynamic haemodynamic parameters for predicting fluid responsiveness in daily clinical routine in icu patients- results from a sub-analysis of the ICU-CardioMan trial

##### S. Funcke^1^, H. Groesdonk^2^, B. Saugel^1^, G. Wagenpfeil^3^, S. Wagenpfeil^3^, D.A. Reuter^1^

###### ^1^University Medical Center Hamburg-Eppendorf, Center of Anaesthesiology and Intensive Care Medicine, Hamburg, Germany; ^2^University Hospital of Homburg/Saar, Homburg/Saar, Germany; ^3^Saarland University, Campus Homburg, Homburg/Saar, Germany

####### **Correspondence:** S. Funcke - University Medical Center Hamburg-Eppendorf, Center of Anaesthesiology and Intensive Care Medicine, Hamburg, Germany

**Introduction:** Investigating the use of dynamic haemodynamic parameters, such as pulse pressure variation (PPV) and stroke volume variation (SVV), for predicting fluid responsiveness prior to volume expansion has led many scientific discussions lately. Also, according to current consensus statements their use is increasingly recommended in suitable patients [1]. Extended hemodynamic monitoring (EHD) beyond measurement of cardiac filling pressures (central venous pressure, pulmonary artery occlusion pressure) is still rare. A recent study revealed that only a minor part of critical ill patients are supervised with specific monitoring to assess fluid responsiveness [2]. It remains unclear if this is due to lacking availability of the specialised devices or the limitations of the use of automated functional parameters of preload.

**Objectives:** This analysis was designed to assess the availability of monitoring, the clinical suitability of using PPV or SVV, and if and how they are actually used in German, Swiss, and Austrian ICU´s.

**Methods:** This is a sub-analysis of the ICU-CardioMan Trial. For this multicentre, one day cross-sectional study data regarding patients' hemodynamic monitoring was collected from 161 participating ICUs from Germany, Switzerland and Austria by means of a web-based case report form. The ICUs gave detailed information on availability of EHD in general and provided data from 1789 patients concerning monitoring, as well as information on the patients' specific characteristics that have impact on treatment suitability of PPV and SVV to predict fluid responsiveness.

**Results:** EHD was widely available throughout the examined ICUs. Only a minority of patients was reported to be monitored with EHD during the study period (see Table 11). In the group of the patients that needed catecholamines and/or vasopressors (39.2 %) the share of patients monitored with EHD was significantly higher. In this subgroup, 66.8 % of those patients had sinus or pacer rhythm. Of this portion another 30.9 % were mechanically ventilated in a controlled mode.

**Conclusions:** Though widely available EHD to predict fluid responsiveness is still not comprehensively implemented in daily clinical routine. Over one third of patients that required catecholamines/ vasopressors fulfilled the criteria for assessment of PPV or SVV. The high share of patients not fulfilling the criteria for using PPV and SVV stresses the need for further development of functional parameters of preload operating independently from the presence of controlled mechanical ventilation.

**References**

1. Cecconi et al. Intensive Care Med. 2014; 40: 1795–815

2. Preau et al. Anaesth Crit Care Pain Med. 2015 Nov 19

**Table 11 (abstract A469).** Implemented extended monitoring

Implemented Monitoring	Total	Vasoactive agents	Mechanical ventilation
	n = 1789	n = 702	n = 874
Invasive pressure monitoring	87.0%	98.6%	96.1%
Cardiac output monitoring	12.3%	24.2%	20.8%
Cardiac filling pressures (CVP, PAOP)	55.4%	74.4%	69.5%
Dynamic parameters (SVV, PPV)	8.7%	16.5%	14.9%

### Mechanical ventilation: clinical studies

#### A470 Reconnection to mechanical ventilation for one hour after a successful spontaneous breathing trial reduces extubation failure and reintubation in critically ill patients: a multicenter randomized controlled trial

##### M.M. Fernandez^1^, R. Fernandez^2^, M. Magret^3^, A. González-Castro^4^, M.T. Bouza^5^, M. Ibañez^6^, C. García^7^, B. Balerdi^8^, A. Mas^9^, V. Arauzo^10^, J.M. Añón^11^, F. Ruiz^12^, J. Ferreres^13^, R. Tomás^14^, M. Alabert^15^, A.I. Tizón^16^, S. Altaba^17^, N. Llamas^18^

###### ^1^Hospital Universitari Mútua de Terrassa, ICU, Barcelona, Spain; ^2^Hospital Sant Joan de Dèu-Fundació Althaia Manresa, ICU, Barcelona, Spain; ^3^Hospital Juan XXIII, Tarragona, Spain; ^4^Hospital Universitario Marqués de Valdecilla, ICU, Santander, Spain; ^5^Hospital de A Coruña, ICU, A Coruña, Spain; ^6^Hospital Verge de la Cinta, ICU, Tortosa, Spain; ^7^Hospital Universitario de Canarias, Tenerife, Spain; ^8^Hospital Universitari Politècnic La Fe, Valencia, Spain; ^9^Consorci Sanitari Integral Moisés Broggi, Barcelona, Spain; ^10^Consorci Hospitalari de Terrassa, Barcelona, Spain; ^11^Hospital Virgen de la Luz-SESCAM, Cuenca, Spain; ^12^Hospital Medico-Quirúrgico de Jaén, Jaén, Spain; ^13^Hospital Clínico de Valencia, Valencia, Spain; ^14^Hospital General de Catalunya, Barcelona, Spain; ^15^Hospital General de Vic, Vic, Spain; ^16^Complexo Hospitalario Universitario de Ourense, Ourense, Spain; ^17^Hospital General Universitario de Castellón, Castellón, Spain; ^18^Hospital Universitario Rafael Méndez de Lorca, Murcia, Spain

####### **Correspondence:** M.M. Fernandez - Hospital Universitari Mútua de Terrassa, ICU, Barcelona, Spain

**Introduction:** Weaning from mechanical ventilation remains a challenge in intensive care units (ICU); respiratory muscle fatigue may be important in difficult weaning. We evaluated whether reconnection to mechanical ventilation for one hour after the effort of a spontaneous breathing trial could reduce extubation failure in critically ill patients.

**Objectives:** reconnection to mechanical ventilation for one hour after the effort of a spontaneous breathing trial could reduce extubation failure in critically ill patients.

**Methods:** In this prospective, randomized, multicenter trial, patients who successfully completed a spontaneous breathing trial were randomized to direct extubation (Control group) or reconnection to the ventilator for a one-hour rest before extubation (Rest group). Statistical analysis included multivariable logistic regression models for extubation failure.

**Results:** We randomized 243 patients to the Control group and 227 patients to the Rest group. The most common spontaneous breathing trial method was T-tube, and the median time from intubation to spontaneous breathing trial was similar between groups (5.7 days in the Control group and 5.5 days in the Rest group; p = 0.2). Extubation failure within 48 hours after extubation was more common in the Control than in the Rest group (58 (23.9 %) vs. 24 (10.6 %) patients; p < 0.001). Rescue noninvasive ventilation was administered in 47 (57 %) patients, 16 (34 %) of whom required reintubation. Reintubation was more common in the Control than in the Rest group (35 (14 %) vs. 12 (5 %) patients; p < 0.001) mainly due to inability to manage secretions in both groups. Hospital and ICU length of stay and mortality did not differ between groups.

**Conclusion:** One-hour rest after a successful SBT reduced the rates of extubation failure within 48 h after extubation and reintubation.

**References**

1. Tobin MJ, Perez W, Guenther SM, et al. The pattern of breathing during successful and unsuccessful trials of weaning from mechanical ventilation. Am Rev Respir Dis 1986;134:111–8.

2. Ely EW, Baker AM, Dunagan DP, et al. Effect on the duration of mechanical ventilation of identifying patients capable of breathing spontaneously. N Engl J Med 1996;335:1864–9.

3. Epstein S, Ciubotaru RL, Wong JB. Effect of failed extubation on the outcome of mechanical ventilation. Chest 1997;112:186–92.

4. Laghi F, D'Alfonso N, Tobin MJ. Pattern of recovery from diaphragmatic fatigue over 24 hours. J Appl Physiol 1995;79:539–46.

5. Laghi F, Cattapan S, Jubran A, et al. Is weaning failure caused by low-frequency fatigue of the diaphragm? Am J Respir Crit Care Med. 2003;167:120–7.

6. Goligher EC, Fan E, Herridge MS, et al. Evolution of diaphragm thickness during mechanical ventilation. Impact of inspiratory effort.Am J Respir Crit Care Med 2015;192:1080–8. (ClinicalTrials.gov number, NCT01915563)

#### A471 Driving pressure is a significant predictor of mortality in the Acurasys and Proseva randomized controlled trials in ARDS patients

##### C. Guérin^1,2^, L. Papazian^3^, J. Reignier^4^, L. Ayzac^5^, A. Loundou^3^, J.-M. Forel^3^

###### ^1^Hospices Civils de Lyon, Lyon, France; ^2^INSERM 955, IMRB, Créteil, France; ^3^APHM, CHU Nord, Marseille, France; ^4^CHU Nantes, Nantes, France; ^5^C-CLIN Sud-Est, Pierre Bénite, France

####### **Correspondence:** C. Guérin - Hospices Civils de Lyon, Lyon, France

**Introduction:** Driving pressure (ΔP) across the respiratory system has been shown to strongly predict hospital mortality in ARDS patients, with a threshold in the vicinity of 14–15 cm H2O above which the relative risk of mortality significantly increased (1). We wonder whether this result may be due to the wide range of tidal volume and PEEP used across the trials included (1).

**Objectives:** Therefore, we investigated ΔP in two trials in which lung protective mechanical ventilation was applied to ARDS patients. Our working hypothesis is that ΔP is a risk factor just like compliance (Crs) or Plateau pressure (Pplat) of the respiratory system are.

**Methods:** ARDS patients included in Acurasys (2) and Proseva (3) trials were used. The first trial compared the neuromuscular blocking agent (NMBA) cisatracurium vs. placebo and the second the prone vs. the supine position. Both had near inclusion criteria (notably PaO2/FIO2 < 150 mm Hg and PEEP ≥ 5 cm H2O) and similar lung protective mechanical ventilation (4) (in particular tidal volume 6 ml/kg predicted body weight and PEEP/FIO2 table). Both found survival benefit in the experimental group. SOFA, continuous NMBA infusion, prone position, combined use of NMBA and prone position, lactate, pH, PaCO2, PaO2/FIO2, lactate, breathing frequency, VT, PEEP, Pplat, Crs and ΔP were recorded at day 1 after inclusion together with gender, age and SAPSII at the time of admission and compared between survivors and nonsurvivors at day 90. Cox proportional hazard models were used with covariates significantly different between survivors and non survivors at the threshold of 0.20 and mortality at day 90 as dependent variable. We planned to test colinearity between ΔP, Crs and Pplat and, if it was verified, to use a specific Cox model for each of them.

**Results:** Both trials enrolled 805 patients of who 787 had data available at day 1 of who 533 survived and 254 did not. In the univariate analysis, ΔP averaged 13.7 ± 3.7 and 12.8 ± 3.7 cmH2O (P = 0.002) in nonsurvivors and survivors, respectively. Colinearity between ΔP, Crs and Pplat was statistically significant. The Cox model for ΔP alone is shown in Table 13. ΔP was associated with a 5 % increased risk of mortality at each 1 cmH2O-increment.

Hazard ratios (HR) were 1.05 (1.02-1.08) (P = 0.004) and 0.985 (0.972-0.999) (P = 0.023) for Pplat and Crs, respectively, in the specific analyses. PEEP and VT were not significant risk factors in any model.

**Conclusions:** When lung protective mechanical ventilation is applied ΔP is still a significant predictor of mortality.

**References**

1. Amato MB. N Engl J Med 2015: 372: 747–755

2. Papazian L. New England journal of medicine 2010:363: 1107–1116

3. Guerin C. N Engl J Med 2013:368: 2159–2168

4. ARDSnet N Engl J Med 2000:342: 1301–1308

**Table 12 (abstract A471).** ᅟ

Variable	HR	HR 95% lower CI	HR 95% upper CI	P value
Age, per year	1.04	1.03	1.05	<0.001
SOFA, per unit	1.06	1.02	1.10	0.001
Continuous NMBA, reference=yes	0.87	0.65	1.18	0.624
Prone position, reference=yes	0.69	0.50	0.94	0.021
Respiratory rate, per unit	1.01	0.99	1.01	0.376
Arterial pH, per unit	0.049	0.008	0.298	0.001
Lactate, per unit	15.84	1.22	205.18	0.034
Lactate x pH, per unit	0.69	0.48	0.98	0.039
ΔP, per cmH20	1.05	1.02	1.09	0.003

#### A472 Practice of ventilation in critically ill patients without ARDS at start of mechanical ventilation (provent) - an international observational study

##### A. Serpa Neto^1,2^, M. Gama de Abreu^3^, P. Pelosi^4^, M.J. Schultz^2^, for the PRoVENT investigators and the PROVE Network

###### ^1^Hospital Israelita Albert Einstein, Critical Care Medicine, São Paulo, Brazil; ^2^Academic Medical Center, University of Amsterdam, Intensive Care, Amsterdam, Netherlands; ^3^University Hospital Carl Gustav Carus, Technische Universität Dresden, 21Pulmonary Engineering Group, Department of Anesthesiology and Intensive Care Medicine, Dresden, Germany; ^4^IRCCS San Martino IST, University of Genoa, Surgical Sciences and Integrated Diagnostics, Genoa, Italy

####### **Correspondence:** A. Serpa Neto - Hospital Israelita Albert Einstein, Critical Care Medicine, São Paulo, Brazil

**INTRODUCTION:** Evidence for harm from mechanical ventilation strategies using high tidal volumes in patients without acute respiratory distress syndrome (ARDS) at onset of ventilation is increasing, but it is highly uncertain how these patients are currently ventilated in intensive care units (ICUs) worldwide.

**OBJECTIVES:** To describe ventilation practice in ICU patients without ARDS at onset of mechanical ventilation, and to compare ventilation settings and outcomes between patients at low *versus* high risk for ARDS.

**METHODS:** This was an international multicenter prospective cohort of patients without ARDS undergoing invasive ventilation, conducted during one week in 119 ICUs from 16 countries across four continents. The Lung Injury Prediction Score (LIPS) was used for risk stratification for ARDS (≤4 [low] vs. > 4 [high]). The primary outcome was to describe ventilation practice in ICU patients without ARDS at onset of mechanical ventilation. Secondary outcomes included comparison of ventilation settings and outcomes between patients at low *versus* high risk for ARDS.

**RESULTS:** Of 3,023 patients screened in participating ICUs, 935 fulfilled inclusion criteria (Fig. 23). Tidal volume sizes, and level of positive end-expiratory pressure (PEEP) were 7.9 [6.8-9.1] ml/kg predicted body weight (PBW), and 5 [5–7] cmH_2_O, respectively. Tidal volume sizes were not different between patients with low and high risk for ARDS; PEEP level was higher in patients with high risk though differences were minimal (6 [5–8] *versus* 5 [5–7] cm H_2_O; *p* < 0.001) (Fig. 24). Occurrence of pulmonary complications and hospital mortality was higher in patients with high risk for ARDS (35.4 % *vs* 23.4 %; *p* < 0.001 and 31.9 % *vs* 15.8 %; *p* < 0.001, respectively) (Fig. 25).

**CONCLUSIONS:** A large proportion of patients without ARDS at onset of mechanical ventilation received high tidal volumes. ?A3B2 show $132#?>Patients with higher risk for ARDS had higher incidence of pulmonary complications and worse clinical outcomes. These findings indicate the potential for improvement in the management of patients without ARDS.

**References**

- Determann RM, et al. Crit Care 2010;14:R1.

- Esteban A, et al. Am J Respir Crit Care Med 2013;188:220–30

- Serpa Neto A, et al. JAMA 2012;308:1651–1659.

- Serpa Neto A, et al. Crit Care Med 2015;43:2155–63.

- Serpa Neto A, et al. Intensive Care Med 2014;40:950–7.

**Fig. 22 (abstract A472).**
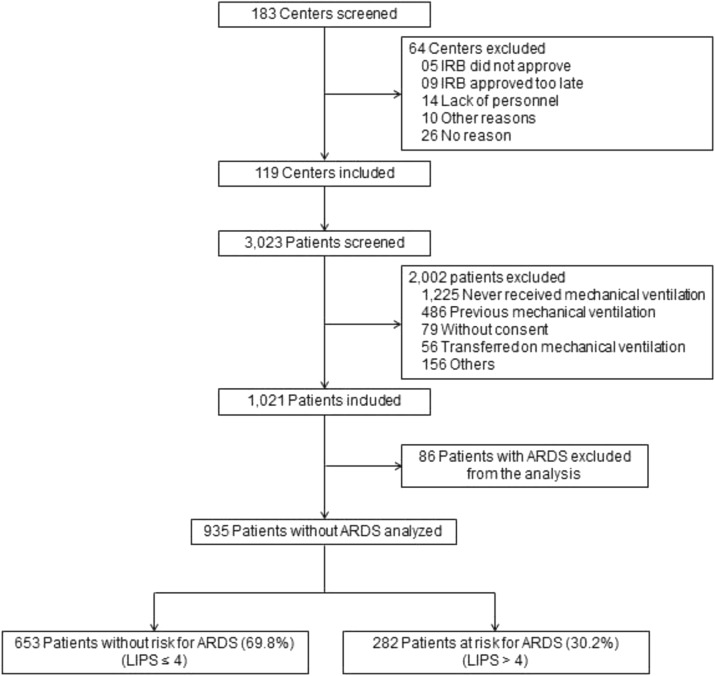
Flow of Patient Screening and Enrollment

**Fig. 23 (abstract A472).**
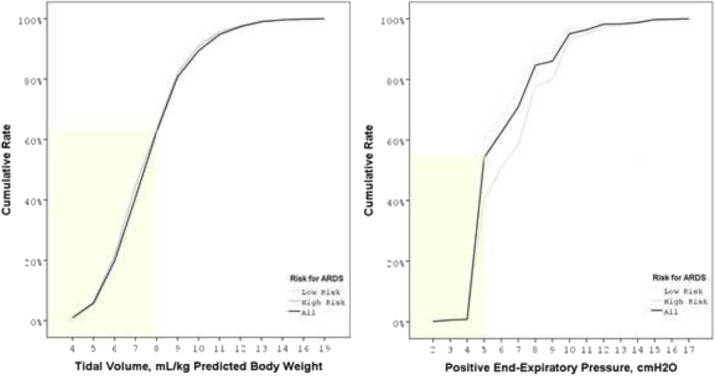
Ventilation parameters in patientswithout ARDS

**Fig. 24 (abstract A472).**
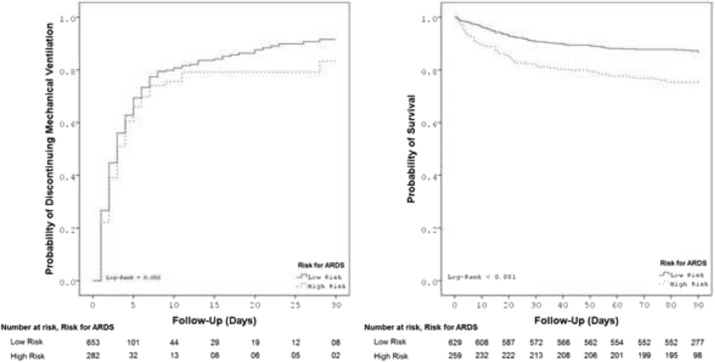
Outcome from patients without ARDS

#### A473 Are changes in diaphragm thickness during mechanical ventilation associated with clinical outcomes? A prospective multi-centre cohort study

##### E.C. Goligher^1^, E. Fan^1^, M. Herridge^1^, S. Vorona^1^, M. Sklar^1^, M. Dres^1,2^, N. Rittayamai^1^, A. Lanys^1^, C. Urrea^1^, G. Tomlinson^3^, W.D. Reid^4^, G.D. Rubenfeld^1^, B.P. Kavanagh^1^, L.J. Brochard^1^, N.D. Ferguson^1^

###### ^1^University of Toronto, Interdepartmental Division of Critical Care Medicine, Toronto, Canada; ^2^Sorbonne Paris Cité, UPMC Univ Paris 06 INSERM, UMRS1158, Groupe Hospitalier Pitié-Salpêtrière Charles Foix Service de Pneumologie et Réanimation Médicale, Paris, France; ^3^University of Toronto, Department of Medicine, Toronto, Canada; ^4^University of Toronto, Department of Physical Therapy, Toronto, Canada

####### **Correspondence:** E.C. Goligher - University of Toronto, Interdepartmental Division of Critical Care Medicine, Toronto, Canada

**Introduction:** Changes in diaphragm thickness (Tdi) are common during mechanical ventilation (MV) (1). However, the impact of changes in Tdi on clinical outcomes is unknown and it is uncertain whether targeting specific levels of inspiratory effort during MV would prevent changes in Tdi or accelerate liberation from MV.

**Objectives:** To determine whether changes in Tdi during the early course of MV are associated with impaired liberation from MV and to further establish the links between ventilator settings, patient inspiratory effort, changes in Tdi, and clinical outcomes.

**Methods:** In 3 ICUs, Tdi and diaphragm thickening fraction (TF, a measure of inspiratory effort) were prospectively measured on a daily basis by ultrasound. Patients were classified according to the initial change in Tdi recorded on the first day that the change in Tdi exceeded 10 % up to MV day 7. Patient characteristics, ventilator settings, severity of illness scores and outcomes in hospital were recorded. Predicted relationships were analyzed by multivariable regression modelling and causal mediation analysis.

**Results:** We enrolled 212 patients (outcomes available for 207). Initial changes in Tdi occurred early in the course of MV (median MV day 3, IQR 3–5). Consistent with our previous findings (1), the rate and direction of change in Tdi over time were strongly associated with TF in this cohort (p < 0.001). Controlled MV was associated with an accelerated decline in Tdi (p = 0.01) mediated by TF (proportion of effect mediated = 0.3, p < 0.01 for mediation effect). Both decreased and increased Tdi were associated with prolonged ventilator dependence (Table 12, Fig. 22, log rank p < 0.001), even after adjusting for age, severity of illness, sepsis, and comorbidities. Mean TF below 15 % or above 30 % over the first 3 days of MV was associated with prolonged ventilator dependence in survivors (adjusted p = 0.02) and a higher risk of complications of acute respiratory failure (adjusted p = 0.02). The associations between mean TF and these outcomes were mediated by changes in Tdi (proportion mediated 0.44, p = 0.05, and 0.29, p = 0.02, respectively).

**Conclusions:** Early changes in diaphragm thickness following initiation of MV are associated with marked differences in clinical outcomes. Both insufficient and excessive inspiratory effort levels are associated with prolonged ventilator dependence due in part to changes in Tdi. Titrating ventilatory support to maintain TF between 15-30 % might prevent changes in Tdi and accelerate liberation from MV.

**References**

1. Goligher EC et al. Evolution of Diaphragm Thickness during Mechanical Ventilation. Impact of Inspiratory Effort. *Am J Respir Crit Care Med* 2015;192:1080–1088.

**Grant acknowledgement**

Supported by a Post-Doctoral Fellowship from the Canadian Institutes of Health Research.

**Table 13 (abstract A473).** Outcomes associated with changes in Tdi during MV

	Initial change in diaphragm thickness during mechanical ventilation					
Outcome	>20% decrease (n=31)	10–20% decrease (n=46)	<10% change (n=85)	10–20% increase (n=29)	>20% increase (n=18)	p-value
Hospital mortality (%)	50%	29%	33%	36%	39%	0.41
Ventilator-free days at 60 days (median, IQR)	32 (0–50)	49 (12–55)	54 (0–57)	37 (0–53)	40 (0–50)	0.003
Duration of mechanical ventilation in survivors (median, IQR)	9 (7–14)	8.5 (5–18)	5 (3–8)	9 (5–22)	12 (9.5–20)	0.0002
Complications of acute respiratory failure (death/reintubation/tracheostomy/MV duration > 14 days) (%)	77%	57%	46%	64%	72%	0.023

**Fig. 25 (abstract A473).**
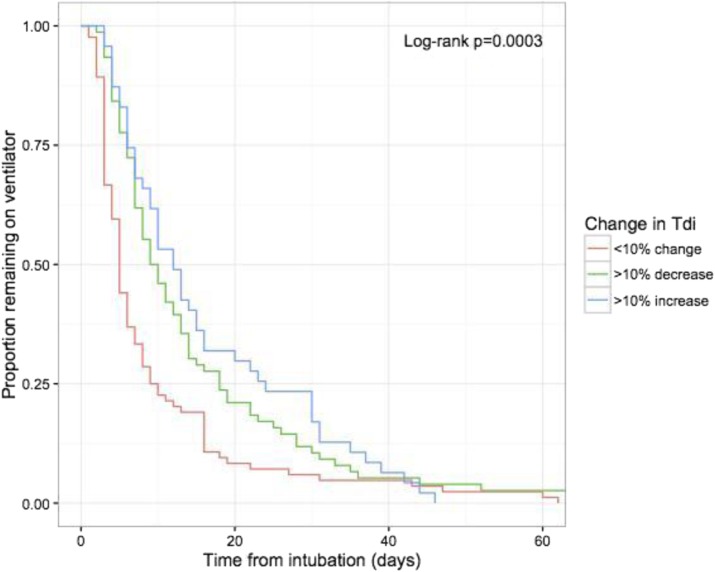
Time to disconnection from the ventilator varies with the initial change in Tdi

#### A474 Impact of breathing variability on clinical outcomes in mechanically ventilated patients - a prospective multicentric study

##### C. Rolland-Debord^1^, C. Bureau^1^, T. Poitou^1^, M. Clavel^2^, S. Perbet^3^, N. Terzi^4^, A. Kouatchet^5^, T. Similowski^1^, A. Demoule^1^

###### ^1^Université Pierre et Marie Curie, UMR_S 1158 and Hôpital Pitié-Salpêtrière, Respiratory Division and Medical ICU, Paris, France; ^2^Hôpital Dupuytren, Limoges, France; ^3^CHU de Clermont-Ferrand and Université d'Auvergne, Clermont-Ferrand, France; ^4^Université Grenoble-Alpes and CHU Grenoble Alpes, Grenoble, France; ^5^CHU d'Angers, Angers, France

####### **Correspondence:** C. Bureau - Université Pierre et Marie Curie, UMR_S 1158 and Hôpital Pitié-Salpêtrière, Respiratory Division and Medical ICU, Paris, France

**Introduction:** Breathing is a cyclic activity that is not monotonous and is therefore variable. During mechanical ventilation (MV), breathing variability is reduced. Breathing variability has been mostly quantified with two indices: the coefficient of variation (CV) that is defined as the standard deviation to the mean ratio, and the amplitude ratio of the spectrum's first harmonic to its zero frequency (H1/DC). Previous reports have suggested that low variability was associated with weaning failure and increased mortality. However, these studies have quantified variability either on very specific time points or on the contrary on the whole intensive care unit stay.

**Objectives:** The aim of our study was to quantify breathing variability at the early phase of weaning according to those two indices. We further evaluated the factors associated with and the prognosis impact of low breathing variability.

**Methods:** Ancillary study of a multicentre, randomized controlled trial comparing neurally ventilator adjusted assist to pressure support ventilation during the early phase of weaning. Airway flow and pressure were recorded during 20 minutes 12, 24, 36 and 48 hours following inclusion. Respiratory rate, tidal volume and mean inspiratory flow were measured. Variability was assessed according to CV and H1/DC. Impact of variability on clinical outcome was determined with a Receiver Operating Characteristic.

**Results:** 108 patients mechanically ventilated for 5 days (3–9) were included, 72 men (68 %), aged 66 (37–86) years, SAPS II 44 (35–59), 62 % were mechanically ventilated for de novo hypoxemic respiratory failure.

**Fig. 26 (abstract A474).**
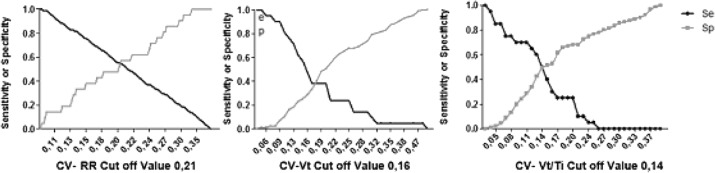
Receiver Operating Characteristic (ROC)

Sensitivity and specificity were plotted as functions of the ability of the CV of respiratory rate (RR), tidal volume (Vt) and mean inspiratory flow (Vt/Ti) to predict day-28 mortality. The curves intersected at a coefficient of variation of respectively 0.21 for respiratory rate, 0.16 for tidal volume and 0.14 for mean inspiratory flow (Fig. 26).

Day-28 mortality was significantly reduced in patients with a high CV-Vt (>16 %) (12 % vs 31 %, p = 0.02) and in patients with a low H1/DC (≤40 %) (16 % vs 60 % p = 0.04). Low variability was not associated with significant difference in term of hospital and intensive care unit length of stay, ventilator free days, and duration of MV. Pulmonary gas exchanges were greater in patients with high CV-Vt (PaO_2_/FiO_2_ = 193 (156–241) vs 230 (183–288), p = 0.03). No significant difference in term of gender, age, SAPS 2, Charlson score or length of MV prior to inclusion was observed with decreased variability.

**Conclusions:** In MV patients at the early phase of weaning, low variability is associated with higher mortality. Low variability is associated with worse oxygenation. Whether low variability is a causative factor of mortality or a consequence of severity remains to be determined.

### Therapies in neurointensive care: effects on physiology and outcomes

#### A475 Relationship between trough haloperidol concentrations and clinical response in critically ill adults with delirium who are managed with a scheduled IV haloperidol protocol

##### N. Hunfeld^1,2^, Z. Trogrlic^1^, S. Ladage^1^, R.J. Osse^3^, B. Koch^2^, W. Rietdijk^1^, J. Devlin^4^, M. van der Jagt^1^

###### ^1^Erasmus Medical Center, Intensive Care, Rotterdam, Netherlands; ^2^Erasmus Medical Center, Pharmacy, Rotterdam, Netherlands; ^3^Erasmus Medical Center, Psychiatry, Rotterdam, Netherlands; ^4^Northeastern University, School of Pharmacy, Boston, MA, USA

####### **Correspondence:** N. Hunfeld - Erasmus Medical Center, Intensive Care, Rotterdam, Netherlands

**Introduction:** Intravenous haloperidol (IVH) is frequently administered on a scheduled basis to treat delirium in critically ill adults. However, dosing recommendations for this indication have been derived from older studies evaluating the use of IVH in psychiatric patients [trough level H: 8.4 μg/L (SD 6.7 μg/L), 3–6 mg per day] without delirium and who are not critically ill. To establish the optimal dose of IVH in critically ill adults with delirium, the relationship between IVH serum concentrations and clinical response is required.

**Objective:** To establish the relationship between serum haloperidol concentrations and clinical response in critically ill adults with delirium who are managed with a scheduled IVH protocol.

**Methods:** This single-center, prospective study included consecutive critically ill adults with delirium without prior exposure to haloperidol, neurological or psychiatric disease, end-stage liver disease, a QTc interval ≥ 450 msec, requiring deep sedation, or requiring a medication known to interact with haloperidol. Delirium was deemed to be present when the bedside nurse and the intensivist agreed that the Intensive Care Delirium Screening Checklist (ICDSC) was ≥ 4 and was managed with a scheduled IVH delirium treatment protocol. IVH was initiated at 1 mg q8h (0.5 mg q8h in patients ≥ 70; 2 mg q8h if agitation present) and titrated upwards q24h by 0.5 mg q8h to 2 mg q8h based on the ICDSC score and delirium symptoms present. Trough haloperidol (TH) serum concentrations were measured on days 2 and 3 of IVH and analyzed with a validated liquid chromatography mass spectometry Method:

**Results:** Baseline characteristics of the 14 patients enrolled were: male (64 %), mean age of 65 (SD 8), Body Mass Index (BMI) of 28 (SD 5) and a SOFA score of 7 (IQR 4 to 8) and were initiated on IVH dose as follows: 0.5 mg q8h (n = 2), 1 mg q8h (n = 10) and 2 mg q8h (n = 2). IVH dosing across day 2 and 3 of haloperidol use was similar with the day it was initiated. The mean H dose (q8h) in the 48 h up to the TH determinations was 1.6 mg (SD 0.19). At the time of IVH initiation, the median (IQR) ICDSC score was 5 (4 to 5.3) and was relatively unchanged across day 2 [4 (3 to 5)] and day 3 [4 (3 to 5)], p = NS. The mean (SD) H serum concentration was not significantly different between days 2 and 3 [2.2 (1.5) vs. 1.5 (0.9) μg/L; *p* = 0.15].

**Conclusions:** This preliminary report is the first study to evaluate the pharmacokinetics and the pharmacodynamic response of low-dose IVH when administered to critically ill adults to treat delirium. We observed low trough levels of IVH and lasting delirium (for the first three days after initiation of IVH). The IVH dose that should be used to treat delirium in the ICU requires further investigation.

#### A476 Intracranial pressure after antipyretic therapy in acute brain injury

##### E. Picetti^1^, P. Ceccarelli^1^, F. Mensi^1^, L. Malchiodi^1^, S. Risolo^1^, I. Rossi^1^, M.V. Antonini^1^, F. Servadei^2^, M.L. Caspani^1^

###### ^1^I Servizio Anestesia Rianimazione, Parma, Italy; ^2^Neurochirurgia e Neurotraumatologia, Parma, Italy

####### **Correspondence:** E. Picetti - I Servizio Anestesia Rianimazione, Parma, Italy

**Introduction:** Fever is a dangerous secondary insult for the injured brain (1). In a recent study (2) examining Paracetamol administration for fever control in acute brain injury (ABI) patients (pts), a reduction in intracranial pressure (ICP) was observed only in subjects with a baseline ICP > 15 mmHg.

**Objectives:** 1) to analyze ICP trend after antipyretics administration, 2) to evaluate if ICP variations are influenced by ICP value before antipyretics administration.

**Methods:** Adults pts with ABI admitted to our Intensive Care Unit (ICU) were prospectively evaluated after antipyretics administration [4 time points: baseline (t-0), 30 minutes (t-30), 60 minutes (t-60) and 120 minutes (t-120)]. Inclusion criteria were: 1) monitoring of intra-arterial blood pressure, core temperature (Tc), and ICP and 2) a Tc ≥ 37.5 °C. Exclusion criteria were: 1) hypovolemia, 2) administration of drugs with hemodynamic effects during the study period, 3) administration of antipyretics in the 6 hours before the start of the study, 4) acute heart failure and 5) cerebral vasospasm.

**Results:** 62 pts [male 35; mean age 52,2 (SD ± 14,2) years; median Glasgow Coma Scale (GCS) 6 (IQR 3–7) at ICU admission] were enrolled. Diagnosis at ICU admission were: 33 (53,25 %) subarachnoid hemorrhage (SAH), 16 (25,8 %) traumatic brain injury (TBI), 10 (16,1 %) intracerebral hemorrhage (ICH) and 3 (4,8 %) acute ischemic stroke (AIS). Paracetamol (1 gr intravenous) was administered to 32 pts and Diclofenac Sodium (12,5 mg intramuscular) to 30 pts. At t-0, 34 pts (54,8 %) showed an ICP ≤ 15 and 28 (45,2 %) an ICP > 15 mmHg. No statistically significant changes in ICP were observed in the overall population after antipyretic administration: 14.9 ± 5.1, 14.9 ± 4.6, 14.4 ± 4.5, 15.1 ± 4.3 mmHg at t-0, t-30, t-60 and t-120 respectively (P = 0.6). A statistically significant reduction in ICP (19.5 ± 3.2, 17.6 ± 4.6, 16.4 ± 4.9, 16.0 ± 4.4 mmHg at t-0, t-30, t-60 and t-120 respectively; P < 0.001) was observed in the group of pts with t-0 ICP > 15 mmHg. Conversely, a statistically significant increase in ICP (11.2 ± 2.7, 12.7 ± 3.4, 12.8 ± 3.5, 14.4 ± 4.1 mmHg at t-0, t-30, t-60 and t-120 respectively; P < 0.001) was observed in the group of pts with a t-0 ICP ≤ 15 mmHg. A statistically significant reduction in Tc, mean arterial pressure (MAP) and cerebral perfusion pressure (CPP) was observed in all groups of patients (overall population and pts with ICP > or ≤ 15 mmHg) after antipyretic therapy.

**Conclusions:** ICP variations are influenced by ICP value before antipyretics administration. If these data are confirmed in future studies, the decision to start antipyretic therapy should take into account the baseline ICP value.

**References**

1. Thompson HJ et Al. Hyperthermia following traumatic brain injury: a critical evaluation. Neurobiol Dis 2003; 12: 163–173.

2. Picetti E et Al. Intravenous paracetamol for fever control in acute brain injury patients: cerebral and hemodynamic effects. Acta Neurochir 2014; 156(10): 1953–9.

#### A477 Continuous osmotherapy for the treatment of post-traumatic intracranial hypertension - a multicenter cohort study

##### A. Roquilly^1^, S. Lasocki^2^, P. Seguin^3^, T. Geeraerts^4^, P.F. Perrigault^5^, C. Dahyot-Fizelier^6^, C. Paugam-Burtz^7^, F. Cook^8^, R. Cinotti^9^, D. Demeure dit Latte^9^, P.J. Mahe^9^, C. Fortuit^9^, F. Feuillet^9^, K. Asehnoune^9^

###### ^1^Nantes University Hospital, Creteil, France; ^2^University Hospital of Angers, Angers, France; ^3^University Hospital of Rennes, Rennes, France; ^4^University Hospital of Toulouse, Toulouse, France; ^5^University hospital of Montpellier, Montpellier, France; ^6^University Hospital of Poitiers, Poitiers, France; ^7^APHP, Beaujon, Beaujon, France; ^8^APHP, Henri Mondor, Creteil, France; ^9^Nantes University Hospital, Nantes, France

####### **Correspondence:** A. Roquilly - Nantes University Hospital, Creteil, France

**Introduction:** Intracranial hypertension (ICH) is one of the main cause of death, and of poor neurological recovery after traumatic brain injury (TBI). The use of a continuous osmotherapy has been described in retrospective single-center studies, but its effectiveness is discussed.

**Objectives:** The first objective was to describe the association between the use of a continuous osmotherapy and mortality in patients developing a post-traumatic ICH. Secondary objectives were to investigate the impact of continuous osmotherapy on long-term neurological recovery and on tolerance.

**Methods:** We pooled the data from three prospective multicenter studies: the corti-TC trial (ref 1), the BI-VILI study (NCT01885507) and the Atlanrea cohort (NCT02426255). We included all patients with traumatic brain-injury, hospitalized in ICU and receiving mechanical ventilation for more than 24 hours.

ICH was defined as one or more episodes of intracranial pressure higher than 20 mmHg and that has required a specific therapeutic intervention.

In one participating center, continuous osmotherapy (NaCL20% (ref 2)) was initiated as the first line treatment of intracranial hypertension, and its administration was pursued up to the end of the period at risk of ICH.

The primary endpoint was the survival rate at day 90. The secondary endpoint was the Glasgow Outcome Scale (GOS) at day 90 (GOS = 1 death, GOS = 5 minor sequelae).

A crude comparison of patients with ICH treated or not with continuous osmotherapy was first performed. To consider the biases related to this observational study, an adjustment on potential confounders (age, glasgow coma scale, pupil reactivity, Marshall score, hypotension and hypoxemia) was performed by both a propensity analysis and a multivariate analysis.

The protocol for this study was approved by an ethics committee.

**Results:** Among the 1054 included patients, 545 (51.7 %) developed ICH.

Out of 143 patients with ICH and treated with a continuous osmotherapy, 106 (74.1 %) were alive at day 90 as compared to 265 (65.9 %) patients with ICH and not treated with continuous osmotherapy (p = 0.07). With the continuous osmotherapy, the relative risk of survival was 1.43 (95 % CI, 0998–2062, p = 0.05). Adjusted hazard ratio for survival at day 90 was 1.43 (95 % CI, 1.02 - 1.99, p = 0.003) in propensity score-adjusted analysis and 1.67 (95%CI, 1.12-2.50, p = 0.01) in multivariate analysis.

At day 90, GOS was higher in patients treated with continous osmotherapy (64 (45.2 %) patients had a GOS = 4–5 vs 115 (35.8 %), p = 0.01).

Severe hypernatremia (≥160 mmol / l) was more frequent in treated patients (9.1 % vs 2.2 %, p < 0.001). No case of central pontine myelinolysis was recorded.

**Conclusions:** Continuous osmotherapy for the treatment of post-traumatic ICH was associated with improved adjusted 90-day survival. A randomized clinical trial is warranted before definitive Conclusion:

**References**

1. Lancet Respir Med 2014; 2(9):706–16

2. Crit Care 2011; 15(5):R260.

**Grant acknowledgement**

None

#### A478 Intracerebral hemorrhage in ICU: is it worth treating?

##### C. Marzorati^1^, S. Spina^1^, V. Scaravilli^1^, A. Vargiolu^2^, M. Riva^1^, C. Giussani^1,3^, E. Sganzerla^1,3^, G. Citerio^1,2^

###### ^1^University of Milan - Bicocca, School of Medicine and Surgery, Milan, Italy; ^2^San Gerardo Hospital, Neurointensive Care, Department of Emergency and Intensive Care, Monza, Italy; ^3^San Gerardo Hospital, Neurosurgical Clinic, Department of Neurosciences, Monza, Italy

####### **Correspondence:** C. Marzorati - University of Milan - Bicocca, School of Medicine and Surgery, Milan, Italy

**Introduction:** Prediction of prognosis in Intracerebral hemorrhage (ICH) is always challenging: the *ICH score* was developed to predict outcomes and limit futile overtreatment^1^. Recent guidelines on ICH management have significantly modified our traditional nihilistic approach^2,3^.

**Objectives:** To compare observed and predicted 6-months mortality in a cohort of consecutive ICH patients admitted to the Neurosurgical ICU of San Gerardo Hospital (Monza, Italy) from 2013 to 2015.

**Methods:** Retrospective analysis of prospectively collected data of ICH patients with ICU length-of-stay >24 hours. We retrieved clinical data, CT scans, DNROs within 48 hours from admission, 30 days mortality and modified Rankin Scale (mRS) along with the ICH score and 6-months survival.

**Results:** 100 consecutive patients (64 ± 14 years old, 57 % males, median GCS 7 at admission, median ICH volume 75 cm^3^) were included.

DNRO: 28 %, all died, 71 ± 11 years old, median GCS 4 at admission, median ICH volume 110 cm^3^.

No DNRO: 72 %, 62 ± 15 years old, median GCS 9 at admission, median ICH volume 47 cm^3^ (p < 0.05 vs. DNRO). Surgery was performed in 71 %.

**Fig. 27 (abstract A478).**
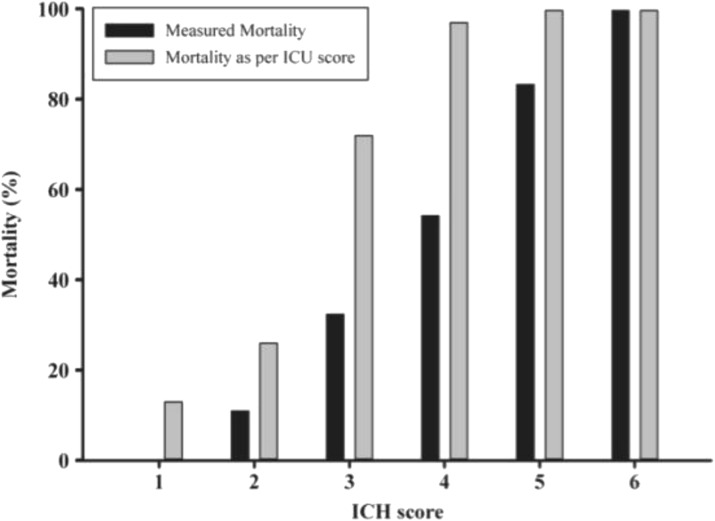
Mortality (observed and expected) at 30 days according to ICH score.

Overall, ICU mortality was lower than expected accordingly to the *ICH score* (see Fig. 27) (i.e. 33 %). mRS was ≤3 and >3 in 21 (30 %) and 46 (64 %) of the discharged patients, respectively. 180-days survival globally approached 60 % (95%CI 50–70).

**Fig. 28 (abstract A478).**
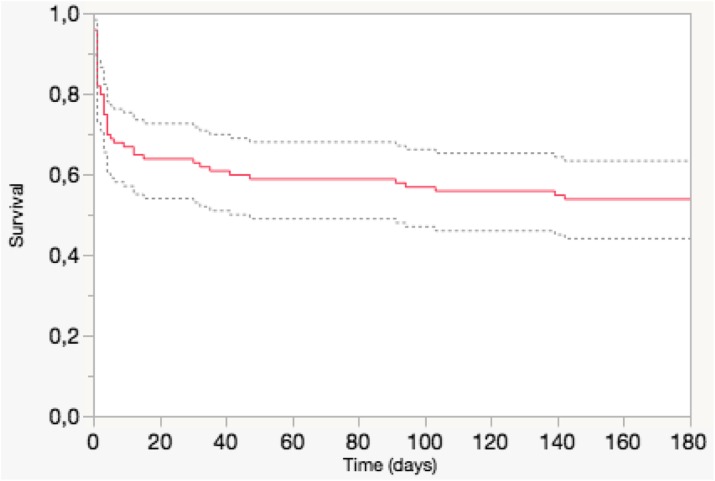
Global 180-days survival (±95%CI) of patients admitted to neuro-ICU with ICH (N= 100 patients).

**Fig. 29 (abstract A478).**
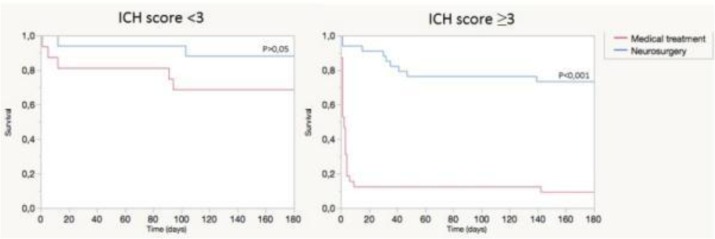
180-days survival of patients undergoing to neurosurgery and receiving only medical treatment

Considering the severity on admission:

- *ICH score* < 3 (33 %), median length of stay in ICU of 3 days. 17 (51 %) patients underwent to neurosurgery. Mortality was non-significantly different between patients receiving neurosurgery and patients receiving medical treatment.

- *ICH score* ≥ 3 (67 %) had a median length of stay of 5.5 days. Neurosurgery was performed in 34 (50 %), and observed short- and long-term mortality was significantly lower for patients that underwent to neurosurgery than in patients receiving medical treatment (see Figs. 28 and 29).

**Conclusions:** We observed mortality to be lower than predicted by *ICH score* and functional outcome to be acceptable in one-third of patients. Full treatment including neurosurgery significantly improved short and long term survival for patients with *ICH score* ≥3.

Prognosis estimation after an ICH remains a challenging subject and early DNROs and limitations of treatment should be carefully evaluated.

**References**

1. Hemphill, J. C. *et al*. The ICH score: a simple, reliable grading scale for intracerebral hemorrhage. *Stroke J. Cereb. Circ*. 32**,** 891–897 (2001).

2. Morgenstern, L. B. *et al.* Full medical support for intracerebral hemorrhage. *Neurology* 84**,** 1739–1744 (2015).

3. Jolink, W. M. T. *et al*. Time trends in incidence, case fatality, and mortality of intracerebral hemorrhage. *Neurology* 85**,** 1318–1324 (2015).

### Respiratory infections

#### A479 Clinical differences between influenza A (H1N1)PDM09 and influenza A(H3N2) infection in critically ill patients

##### S. Barbadillo^1^, F.J. González de Molina^2,3^, F. Álvarez-Lerma^4^, A. Rodríguez^5^, SEMICYUC/GETGAG Working Group

###### ^1^Hospital General de Catalunya, Intensive Care Department, Sant Cugat del Valles, Spain; ^2^Hospital Universitari Mútua de Terrassa, Intensive Care Department, Terrassa, Spain; ^3^AGAUR, Grup Recerca Emergent, Terrassa, Spain; ^4^Hospital del Mar, Intensive Care Department, Barcelona, Spain; ^5^Hospital Universitari de Tarragona Joan XXIII, Intensive Care Department, Tarragona, Spain

####### **Correspondence:** S. Barbadillo - Hospital General de Catalunya, Intensive Care Department, Sant Cugat del Valles, Spain

**Introduction:** Influenza infection causes severe morbidity and mortality around the world. Pandemic influenza A(H1N1)pdm09 have been describe to increases disease severity comparing with seasonal flu viruses. There are limited data comparing clinical differences between influenza A (H1N1)pdm09 and influenza A(H3N2) infection in critically ill patients.

**Objectives:** Our aim was to analyse the demographic and clinical differences between patients admitted to ICU due to pandemic influenza A(H1N1)pdm09 and seasonal influenza A(H3N2) infection.

**Methods:** Prospective, observational, multicenter study conducted in 148 Spanish ICUs from 2009 to 2015. Individuals with Influenza A(H1N1)pdm09 were compared with those infected by influenza A (H3N2). All serotypes were confirmed using RT-PCR at ICU admission. Patients´ demographic, clinical, radiologic features, laboratory values, ICU and hospital length of stay (LOS) and outcomes were recorded. Discrete variables are expressed as percentage and continuous variables as medians with 25th to 75th interquartile range (IQR). Differences between groups were assessed using the *x*2 test and the Fisher exact test for categoric variables and Mann–Whitney *U* test for continuous variables.

**Results:** Of 2898 patients with confirmed influenza infection at ICU admission, 110 were excluded due to influenza B (n = 56) or non-serotype A typification (n = 54). At all, 2421 patients with influenza A (H1N1) were compared to 367 patients with influenza A (H3N2). Patients with A(H1N1) were much younger (50 [38–61] vs 61 [50–73], P < 0.001), presented a higher multiorgan dysfunction (61,2 % vs 55,4 %, P = 0.038), and required more invasive mechanical ventilation (70,6 % vs 65.1 %, P = 0.046), and prone position(18.9 % vs 13.0 %, P = 0.007). Patients with A(H3N2) had more comorbidities: COPD (30.4 % vs 19.5 %, P < 0.001), heart failure(18,6 % vs 10.0 %, P < 0.001), chronic renal failure(14,5 % vs 7,6 %, P < 0.001), and diabetes(23,9 % vs 15,3 %, P < 0.001). Patients with A(H3N2) presented more bacterial coinfection pneumonia (26,5 % vs 14,4 %, P < 0.001) and a higher vaccination rate (23,5 % vs 5,7 %, P < 0.001). Influenza A(H1N1) group have an increased ICU length of stay (LOS) (11[5–21] vs 8[4–19], P < 0.001) but there is no difference in hospital LOS. No statistically significant difference in overall mortality was observed (21.9 % H1N1 vs 24.2 % H3N2, P = 0337).

**Conclusions:** Our data suggest than Infuenza A subtypes have different clinical presentation in critically ill patients. Influenza A(H1N1)pdm09 affect younger patients with less comorbidities suffering an acute and more severe respiratory disease. Influenza A (H3N2) is presented more frecuently with bacterial coinfection in older patients with more chronic illness and especially CPOD. The overall mortality was high without differences between groups.

**References:** Schaffer A.et al. BMC Public Health. 2012; 12:869. Mitchell R. et al. Am J Infect Control. 2013; 41:1032–7.

#### A480 The dynamics of the pulmonary microbiome during mechanical ventilation in the intensive care unit and the association with occurrence of pneumonia

##### T. Zakharkina^1^, I. Martin-Loeches^2^, S. Matamoros^1^, P. Povoa^3^, A. Torres^4^, J. Kastelijn^1^, J.-J. Hofstra^1^, M. de Jong^1^, M. Schultz^1^, P. Sterk^1^, A. Artigas^5^, L.J. Bos^1^

###### ^1^Academic Medical Center, University of Amsterdam, Amsterdam, Netherlands; ^2^St James's University Hospital, Dublin, Ireland; ^3^Hospital São Francisco Xavier, Lisbon, Portugal; ^4^Hospital Clinic, Barcelona, Spain,;^5^Autonomous University of Barcelona, Barcelona, Spain

####### **Correspondence:** L.J. Bos - Academic Medical Center, University of Amsterdam, Amsterdam, Netherlands

**Introduction:** During the past ten years the paradigm of a “healthy lung is a sterile lung” was challenged [1–5]. The healthy lung appeared to be populated by multiple resident bacterial species, that migrate to the distal airways from the oral cavity [2]. According to the adapted island model the respiratory microbiome represents a dynamic community, where the equilibrium point is achieved by the balance between immigration and elimination mechanisms [6].

**Objectives:** We hypothesized that mechanical ventilation and antibiotic administration decrease the diversity of the respiratory microbiome and that these changes are more profound in patients who develop ventilator-associated pneumonia (VAP).

**Methods:** Intubated and mechanically ventilated ICU-patients were included. Tracheal aspirates were obtained three times a week. 16S RNA sequencing with the Roche 454 platform was used to measure the composition of the respiratory microbiome. Associations were tested with linear mixed model analysis. The relative changes were compared between patients that did and did not develop VAP.

**Results:** 35 patients were included; 11 had VAP, 18 did not have VAP. Six additional patients developed pneumonia within the first 48 hours after intubation. Duration of mechanical ventilation was associated with a decrease in Shannon diversity (fixed-effect regression coefficient (ß): −0.03 [95%CI: −0.05 - -0.005]), but the administration of antibiotic therapy was not (fixed-effect ß: 0.06; 95%CI: −0.17 - 0.30). There was a statistically significant increase in abundance of Pseudomonadales and a decrease in abundance of Lactobacillales, Gemellales, Actinomycetales and Clostridiales between the moment of intubation and extubation. There were no statistically significant differences between patients that did and did not develop VAP.

**Conclusions:** Mechanical ventilation, but not antibiotic administration, was associated with changes in the respiratory microbiome. Duration of mechanical ventilation was associated with increased abundances of Pseudomonadales. The dynamics in the respiratory microbiome were not different between patients that did and did not develop VAP.

**References**

1. Dickson RP. *Ann Am Thorac Soc* 2015.

2. Bassis CM. mBio 2015.

3. Morris A. *Am J Respir Crit Care Med* 2013.

4. Zakharkina T. *PLoS One* 2013.

5. Segal LN. *Ann Am Thorac Soc* 2014.

6. Dickson RP. *Annu Rev Physiol* 2015.

**Grant acknowledgement**

Grant by the Instituto de Salud Carlos III (ISCIII) (ISCIII/FIS-PI 12/01815) Spanish Government and Institut Merieux Research grant.

#### A481 Impact of immunosuppression on the incidence, etiology, and outcome of ventilator-associated lower respiratory tract infections: a post-hoc analysis of the TAVeM database

##### A.-S. Moreau^1^, I. Martin-Loeches^2^, P. Povoa^3^, J. Salluh^4^, A. Rodriguez^5^, S. Nseir^1^, TAVeM study group

###### ^1^Lille University Hospital, ICU, Lille, France; ^2^Trinity Centre for Health Sciences, St James's University Hospital, Critical Care Medicine, Dublin, Ireland; ^3^Centro Hospitalar de Lisboa Ocidental, São Francisco Xavier Hospital, ICU, Lisbon, Portugal; ^4^D'Or Institute for Research and Education, Rio de Janeiro, Brazil; ^5^Joan XXIII University Hospital, Institut d'Investigació Sanitària Pere Virgili, Tarragona, Spain

####### **Correspondence:** A.-S. Moreau - Lille University Hospital, ICU, Lille, France

**Introduction:** Immunocompromised (IC) patients have poor outcome in the ICU. To our knowledge, no study to date has specifically evaluated ventilator-associated (VA) lower respiratory tract infections (LRTI) in this population.

**Objectives:** To determine the incidence, etiology and outcome of VA-LRTI in IC patients, and to compare it with patients with no apparent immunosuppression.

**Methods:** Post-hoc analysis of the large prospective multinational TAVeM database, coming from 114 ICUs [1]. All consecutive patients receiving mechanical ventilation for >48 h were included. The incidence, etiology and outcome of VA-LRTI (ventilator-associated tracheobronchitis (VAT), and ventilator-associated pneumonia (VAP) [1]) were compared between IC (neoplasia, haematological malignancy, AIDS, allogeneic stem cell transplant, immunosuppressant drug, organ transplant) and non-IC patients.

**Results:** Among the 2960 included patients, 663 (22 %) were IC. The incidence of VA-LRTI was significantly lower in IC compared with non-IC patients (116 (17.5 %) versus 573 (25 %), p < 0.0001, OR = 0.64 [95%CI 0.51-0.79]). Although VAT incidence was significantly lower in IC compared with non-IC patients (7.8 % vs 11.7 %, p = 0.04), no significant difference was found in VAP incidence between the two groups (9.7 % vs 13.3 %, p = 0.13, respectively).

In patients with VA-LRTI, rate of prior antibiotic treatment was significantly higher in IC compared with non-IC patients (80 % vs 66 %, p = 0.001). The incidence of progression from VAT to VAP was similar in IC and non-IC patients (7/52 [13 %] vs 32/268 [12 %], p = 0.76).

IC and non-IC patients received appropriate antibiotics for VA-LRTI in the same proportions (77 % vs 79 %, p = 0.5). Same results were obtained in VAT and VAP subgroups. Among IC patients with VAT, 39/52 (75 %) received appropriate antibiotics. Percentage of IC patients with progression from VAT to VAP was significantly lower in patients who received appropriate compared with those who received inappropriate antibiotic treatment (8 % vs 31 %, p = 0.035, OR 0.19 (95 % CI 0.03-0.99)).

The incidence of multidrug resistant (MDR) bacteria was higher in IC compared to non-IC patients with VA-LRTI (72 % vs 59 %, p = 0.01), similar results were found in the subgroup of patients with VAP (78 % vs 58 %, p = 0.001), but not in those with VAT (65 % vs 61 %, p = 0.52).

Among patients with VA-LRTI, MRSA (5.2 % vs 1.7 %, p = 0.025) and Enterobacter spp. (21 % vs 10 %, p = 0.001) were significantly more frequent in IC compared with non-IC patients.

ICU mortality rate was higher in IC compared with non-IC patients with VA-LRTI (54 %, vs 30 %, p < 0.0001, OR 2.68 (95 % CI 1.78-4.02)). Similar results were obtained in VAT and VAP subgroups.

**Conclusions:** Incidence of VA-LRTI is significantly lower in IC compared with non-IC patients. MDR bacteria and mortality rates are significantly higher in IC compared with non-IC patients with VA-LRTI.

**References**

1. Martin-Loeches I et al. Lancet Respir Med 2015, 3:859–68

**Table 14 (abstract A481).** Outcomes of patients with VA-LRTI

Immunocompromised n=663					Non-immunocompromised n = 2297			
	VAT n=52	VAP n=64	No VA-LRTI n=547	p	VAT n=268	VAP n=305	No VA-LRTI n=1724	p
Mechanical ventilation duration, d	16 (10–25.5)	15 (8–27)	7 (4–14)	<0.001	13 (8–22)	14 (8–26)	7 (4–12)	<0.001
Length of ICU stay, d	23 (16–38)	20 (13–30)	12 (7–20)	<0.001	21 (14–33)	21 (13–34)	12 (8–19)	<0.001
ICU mortality, %	42	64	39.5	0.001	26.5	34	26.5	0.016

#### A482 Procalcitonin guided antibiotic therapy in severe community- acquired pneumonia. Randomized controlled trial

##### E. de Jong^1^, J.A. van Oers^2^, A. Beishuizen^3^, A.R.J. Girbes^1^, M.W.N. Nijsten^4^, D.W. de Lange^5^

###### ^1^VU University Medical Center Amsterdam, Intensive Care, Amsterdam, Netherlands; ^2^ST Elisabeth Twee Steden Hospital, Intensive Care, Tilburg, Netherlands; ^3^Medisch Spectrum Twente, Intensive Care, Enschede, Netherlands; ^4^University Medical Center Groningen, Intensive Care, Amsterdam, Netherlands; ^5^University Medical Center Utrecht, Intensive Care, Utrecht, Netherlands

####### **Correspondence:** E. de Jong - VU University Medical Center Amsterdam, Intensive Care, Amsterdam, Netherlands

**Introduction:** Severe community-acquired pneumonia (CAP) remains a significant cause of morbidity and mortality [1]. In the Netherlands the guidelines recommends a duration of antibiotic therapy in severe CAP for at least five till seven days.

**Objectives:** The purpose of this trial was to evaluate whether procalcitonin measurements were able to reduce antibiotic usage in patients with severe CAP in Dutch intensive care units by reducing the duration of antibiotic treatment without increasing mortality or recurrent infections.

**Methods:** From 2009 until 2013 a randomized intervention trial was performed in three university medical centres and 12 teaching hospitals in the Netherlands [2]. In this Dutch multicentre trial all patients admitted to the intensive care unit and who received antibiotics for presumed infection were assigned to a PCT-guided or standard-of-care antibiotic discontinuation. In this study a sub analysis for all patients with a severe community-acquired pneumonia was performed. Stopping advice for antibiotics was provided when PCT was ≤20 % of its peak value or ≤0.5 ug/L. Mortality and duration of antibiotic treatment (DOT) were the primary endpoints of this study.

**Results:** In 15 ICUs 440 patients with severe community acquired pneumonia were randomized for PCT-guidance (n = 208) or standard-of-care (n = 232). The median DOT was 5.5 (3–8) and 7 days (4–10) respectively (P < 0 · 001). No difference in reinstitution of antibiotics or CRP-levels within 28 days after randomization was observed. Mortality at 28 days was 43/208 (20.7 %) and 58/232 (25 · 0 %) in the PCT and standard-of-care group respectively (P = 0.31). One-year mortality was 63/208 (30.3 %) and 90/232 (38.8 %) in the PCT and standard-of-care group respectively.

**Conclusions:** In this prospective randomized trial, the addition of PCT-measurements to assist intensivists in duration of antibiotic therapy in severe community-acquired pneumonia, resulted in a clear reduction of antibiotic treatment from 7 days (IQR 4–10 days) in the control group to 5.5 days (IQR 3–8 days) in the PCT-guided patient group, without increase of mortality or subsequent antibiotic prescriptions.

**References**

1. Ramírez P1, Ferrer M, Martí V, Reyes S, Martínez R, Menéndez R, Ewig S, Torres. Inflammatory biomarkers and prediction for intensive care unit admission in severe community-acquired pneumonia. Crit Care Med. 2011 Oct;39(10):2211–7. doi: 10.1097/CCM.0b013e3182257445.

2. Wiersinga WJ, Bonten MJ, Boersma WG, Jonkers RE, Aleva RM, Kullberg BJ, Schouten JA, Degener JE, Janknegt R, Verheij TJ, Sachs AP, Prins JM. SWAB/NVALT guidelines on the management of community-acquired pneumonia in adults. Neth J Med. 2012 Mar;70(2):90–101.

3. de Jong E, van Oers JA, Beishuizen A, Vos P, Vermeijden WJ, Haas LE, et al. Efficacy and safety of procalcitonin guidance in reducing the duration of antibiotic treatment in critically ill patients: a randomised, controlled, open-label trial. Lancet Infect Dis. 2016 Feb 29

**Fig. 30 (abstract A482).**
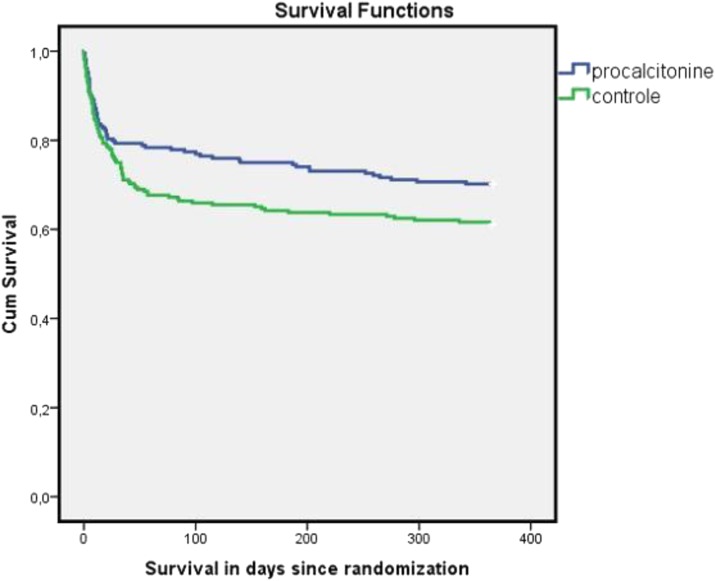
Kaplan-Meier plot for survival

#### A483 Type of stress-ulcer prophylaxis and incidence of ventilator-associated pneumonia

##### D. Bonvicini^1^, D. Labate^1^, L. Benacchio^2^, A. Olivieri^2^, E. Pizzirani^1^

###### ^1^Ulss 15 Alta Padovana, Anesthesia and Intensive Care Unit, Camposampiero, Italy; ^2^Ulss 15 Alta Padovana, Epidemiology Unit, Camposampiero, Italy

####### **Correspondence:** D. Labate - Ulss 15 Alta Padovana, Anesthesia and Intensive Care Unit, Camposampiero, Italy

**Introduction:** Ventilator-Associated Pneumonia (VAP) is a leading nosocomial infection in intensive care unit (ICU) patients undergoing mechanical ventilation (MV). Giving that such patients show an increasing risk of important gastrointestinal (GI) bleeding, stress-ulcer prophylaxis (SUP) has been recommended for the prevention of upper GI hemorrhage. SUP strategy rely on drugs that block the secretion of gastric acid and increase the gastric pH (histamine-2-receptor antagonists - H2RA, and proton pump inhibitor - PPI) and those that does not alter gastric pH (sucralfate). The increase of gastric pH leads to bacterial overgrowth and potential colonization of trachea determining a higher risk of VAP.

**Objectives:** In the setting of a study designated to assess VAP outcomes, we collected data on known VAP risk factors (SUP strategy).

Our objective was to evaluate the risk of VAP accounting for exposure to H2RA, PPI and sucralfate.

**Methods:** We recruited 772 patients in a multicenter prospective observational study, conducted in 21 Italian ICUs. Patients admitted to the ICU for at least 48 hours, received MV from the admission, and were followed until discharge, the day of withholding MV, the day of transfer in other hospital ward, or death.We recorded demographic data, admission category, and severity scores on admission. Each day of ICU staying SUP approach (H2RA, PPI or sucralfate) was recorded. VAP was defined as pneumonia occurring after a MV period of at least 48 hours during the ICU stay.Independent relationships between VAP incidence and the specific SUP approach was assessed by using a logistic regression analysis.

**Results:** Among 772 patients experiencing on average 11.3 days of MV, 116 (15 %) developed VAP. Sucralfate was less frequently used (8 %), while 28 % has been receiving H2RA and 77 % PPI. The risk of VAP was higher for patients treated mainly with PPI (OR 4.27, 95%CI 2.57-7.09) than those who received H2RA (OR 2.30, 95%CI 1.08-5.07).

**Conclusions:** Our study show that the use of ranitidine is associated with a lower risk of VAP, compared with PPI. Sucralfate was less frequently used in the participating ICUs. Although some meta-analysis indicate a lower risk of VAP associated with sucralfate, an important study have not established a superiority of this medication compared with ranitidine [1]. Miano et al. compared the use of pantoprazole vs ranitidine in Cardiothoracic Surgery patients showing that pantoprazole was associated with a higher rate of VAP [2]. Such VAP rate associated with PPI would seem to be linked to a greater antacid power than ranitidine. This can lead to a more extensive gastric bacterial colonization in patients treated with these drugs. Randomized-Controlled Trial of Stress-ulcer prophylaxis are needed to assess which drug show the highest risk of VAP and the relationship with the incidence of major GI bleeding.

**References**

1 Cook D et al. N Eng J Med 1998; 338:791–7

2 Miano TA et al. Chest 2009; 136(2):440–7

### Clinical studies in perioperative ICU

#### A484 Influence of postoperative albumin levels in the outcome of cardiac surgery

##### J.C. Lopez-Delgado^1^, M. Gonzalez-Romero^1^, V. Fuentes-Mila^1^, D. Berbel-Franco^1^, I. Romera-Peregrina^1^, A. Martinez-Pascual^1^, J. Perez-Sanchez^1^, R. Abellan-Lencina^1^, R.E. Ávila-Espinoza^1^, G. Moreno-Gonzalez^1^, F. Sbraga^2^

###### ^1^Hospital Universitari de Bellvitge, Intensive Care, L' Hospitalet de Llobregat, Spain; ^2^Hospital Universitari de Bellvitge, Cardiac Surgery, L' Hospitalet de Llobregat, Spain

####### **Correspondence:** J.C. Lopez-Delgado - Hospital Universitari de Bellvitge, Intensive Care, L' Hospitalet de Llobregat, Spain

**Introduction:** Low preoperative albumin reflects a poor nutritional status that influences outcome in cardiac surgery (CS). However, postoperative albumin levels are influenced by nutritional status and inflammatory response. Thus, its value and utility as a prognostic marker is unclear.

**Objectives:** To evaluate the influence of postoperative plasma levels of albumin in the outcome of patients who underwent cardiac surgery (CS).

**Methods:** Prospective, observational study in Surgical ICU in a referral hospital. Albumin was measured during the first 24 h after CS, together with clinical data and outcomes including in-hospital and long-term mortality (follow-up of 4.6 ± 2.4 years). Patients were classified into different categories based on plasma albumin levels: normal(≥35 g•L^−1^), low deficit(30–34.9 g•L^−1^), moderate deficit(25–29.9 g•L^−1^) and severe deficit (<25 g•L^−1^).

**Results:** 2818 patients were included. Mean age was 64.5 ± 11.6 years; 63.8 % (n = 1799) were male; Body Mass Index: 28 ± 4.3Kg · m-2. 5.8 %(n = 162) had normal levels, 32.8 %(n = 924) low deficit, 44.3 %(n = 1249) moderate deficit and 17.1 %(n = 483) severe deficit. We showed that not having low albumin levels 24 h after CS was protector for in-hospital (HR:0.844;95 % IC:0.805-0.844;*P = 0.007*) and long-term mortality (HR:0.846; 95 % IC:0.821-0.871; *P < 0.001*).

Subgroups of patients with low albumin levels showed worst survival during hospital stay (98.1 % for normal subgroup, 97.3 % low deficit, 95 % moderate deficit and 85.9 % severe deficit; *P < 0.001*) and from the long-term mortality scenario (5-year mortality was 94.3 % for normal subgroup, 87.4 % low deficit, 83.1 % moderate deficit and 72.4 % severe deficit; *P < 0.001*). Multivariable analysis showed higher in-hospital mortality, sepsis and hemorrhage related complications and higher ICU stay in subgroups of patients with low albumin levels

Predictors for the presence of any levels of hypoalbuminemia were suffering from any degree of chronic kidney disease (OR:1.316;95 % IC:1.085-1.595;*P = 0.005*), preoperative low hemoglobin levels (OR:1.060;95 % IC:1.033-1.088;*P < 0.001*), previous CS (OR:1.229;1.067-1.415;*P = 0.004*) and longer Cardiopulmonary bypass time (OR:1.904;95 % IC:1.902-2.128;*P < 0.001*).

**Conclusions:** Physicians may be aware about the degree of hypoalbuminemia in the postoperative of CS due to its influence in outcomes, even in long-term survival. Relationship between factors associated with nutritional and inflammatory status may be associated with the development of postoperative hypoalbuminemia.

**References**

1. Karas PL, Goh SL, Dhital K. Is low serum albumin associated with postoperative complications in patients undergoing cardiac surgery? Interact Cardiovasc Thorac Surg. 2015 Dec;21(6):777–86. doi: 10.1093/icvts/ivv247.

#### A485 Reducing the incidence of post-operative pulmonary complications (POPC) in high-risk surgical patients: development of a care bundle (CB) to be applied in addition to standard enhanced recovery pathways - a survey of the ESICM POIC section and a Delphi expert consensus by the POPC-CB investigators

##### S. Griffiths^1^, M.P.W. Grocott^1,2^, B. Creagh-Brown^3,4^, POPC-CB investigators

###### ^1^University of Southampton, Southampton, UK; ^2^University Hospital Southampton, Southampton, UK; ^3^Royal Surrey County Hospital, ICU and SPACeR Research Group, Guildford, UK; ^4^University of Surrey, Guildford, UK

####### **Correspondence:** B. Creagh-Brown - University of Surrey, Guildford, UK

**Introduction:** Post-operative pulmonary complications (POPC) are common with a reported incidence ranging from 2 %-40 %. Adverse outcomes include death, longer hospital stays and lower long term survival, which is independent of pre-operative risk. The most widely recognised prediction tool for POPCs is ARISCAT. Interventions to reduce POPCs have been studied individually but the use of a care bundle (CB) has not been widely investigated. Enhanced recovery (ER) pathways are 'standard of care' for major surgery - however some patients may benefit from additional focused interventions. An example of a CB for POPCs is the ´I COUGH´ study, which demonstrated a trend towards reduction in POPCs. Results may be superior if the intervention is targeted to a high-risk group.

**Objectives:**

1) To perform a survey of members of the ESICM POIC section: to choose international experts for the Delphi and to share their opinions on a POPC-CB.

2) To recruit a team of experts to participate in and complete an email-based Delphi consensus, leading to the formulation of a CB.

**Methods:** A SurveyMonkey of members of the ESICM POIC section was conducted. International experts were identified from this survey. A Delphi consensus was conducted anonymously via email over 3 rounds, to 36 independently chosen experts. Each round comprised of a series of statements which experts were asked to respond to using a five-point Likert scale. There were free text spaces for comments and the possibility for papers to be circulated. After each round, anonymous feedback was circulated to the Delphi group and the previous results were used to create the statements for the subsequent round. At the end of the process, final feedback was sent to the group with the consensus and suggested care bundle.

**Results:** Survey: 362 respondents.

·Routine screening for high-risk of POPC occurs in 50 % of centres.

·Information about current practice in relation to various pre-, intra- and post-operative interventions was ascertained.

·Almost 80 % of respondents would be happy to assess the feasibility of implementing a POPC-CB.

Delphi: The preferred number of components in the POPC care bundle was 7.

Care bundle elements supported by the consensus process were:

·PRE-operatively: a supervised exercise programme and inspiratory muscle training.

·INTRA-operatively: low tidal volume ventilation with individualised PEEP, use of routine monitoring to avoid hyperoxia and efforts made to limit neuromuscular blockade.

·POST-operatively: deep breathing exercises and mandatory elevation of the head of the bed.

**Conclusions:** A POPC care bundle has been suggested for use, in addition to enhanced recovery pathways, in major surgical patients at high-risk of POPC. Prospective evaluation of feasibility, before an evaluation of effectiveness, is now indicated.

#### A486 The development and impact of implementation of a quality improvement care pathway for patients undergoing an emergency laparotomy

##### J. Doyle^1^, P. Wilkerson^2^, Y. Soon^2^, S. Huddart^3^, M. Dickinson^3^, A. Riga^4^, A. Zuleika^5^

###### ^1^Department of Intensive Care Medicine and Surrey Peri-Operative Anaesthesia and Critical Care Collaborative Research Group (SPACER), Guildford, UK; ^2^Department of Surgery, Royal Surrey County Hospital NHS Foundation Trust, Guildford, UK; ^3^Department of Anaesthesia, Royal Surrey County Hospital NHS Foundation Trust, Guildford, UK; ^4^Department of Surgery, Royal Surrey County Hospital NHS Foundation Trust, Guilford, UK; ^5^Department of Intensive Care Medicine and Surrey Peri-Operative Anaesthesia and Critical Care Collaborative Research Group (SPACER) and Department of Anaesthesia, Guildford, UK

####### **Correspondence:** J. Doyle - Department of Intensive Care Medicine and Surrey Peri-Operative Anaesthesia and Critical Care Collaborative Research Group (SPACER), Guildford, UK

**Introduction:** 80 % of UK in-hospital surgical mortality occurs in those undergoing high-risk surgical intervention such as emergency laparotomy (EL) (1) these patients are among the largest consumers of hospital resources (2, 3). Furthermore it was suggested that UK outcomes are worse than comparable countries (4).

In the 2011 review of peri-operative care of high-risk patients NCEPOD reported care was good in less than half of patients leading to considerable health and financial costs (5).

As such there was a clear need for a change in the approach to patients having an EL. Since this study the first national emergency laparotomy audit (NELA) has been published demonstrating a 30-day mortality of 11 % (6).

**Objectives:** The aims were to create a focused, evidence-based Emergency Laparotomy Pathway (ELP) delivered by a multidisciplinary (MDT) team, utilising care bundles to improve delivery of care and assess the impact of the pathway on hospital mortality and length of stay (LOS).

**Methods**

**Pathway design:** A Emergency Laparotomy Group (ELG) was convened and designed the ELP based on principles of best basic practice and the recommendations of the RCS (2) and NCEPOD (5). The ELP covered the entire surgical stay of the patient in 4 stages.

**Study protocol:** Data was collected for consecutive patients, 50 pre and 96 post-ELP during 2011/2012. The primary outcome measure was in hospital mortality. Secondary outcomes were ICU mortality, ICU and hospital LOS.

**Results:** The in-hospital mortality fell from 21.7 % Pre-ELP to 9.6 % Post-ELP (p = 0.048). There is a strong trend towards significance in a survival analysis (p = 0.053). There was no significant difference in hospital LOS between Pre and Post cohorts. (Median 15.5 days vs 15 days, p = 0.875). There was no difference in the ICU LOS (3 vs 2 days, p = 0.3339), nor in the utilisation of high level beds.

**Conclusion:** The introduction of an ELP reduced the in hospital mortality significantly, this has since been further evidenced with a 4-centre (including our own) study demonstrating a case-mix adjusted decrease risk of death from 15.6 % to 9.6 %, p = 0.002 (7). ELP is a useful tool reducing the risk of failure to deliver optimal care.

**References**

1. Pearse RM. Identification and characterisation of the high-risk surgical population in the UK. Crit care. 2006;10(3):R81

2. Anderson ID. Report on Peri-op Care of the High Risk General Surgical Patient. 2011

3. Clarke A. Mortality and postop care after EL. Eur J of Anaes. 2011;28(1):16

4. Bennett-Guerrero E. Comparison of P-POSSUM risk-adjusted mortality rates after surgery between patients in the USA and UK. BJS. 2003;90(12):1593

5. Findlay GP. NCEPOD Knowing the Risk. review of the peri-op care of surgical patients. 2011

6. NELA project team. First patient report of NELA. RCoA 2015.

7. Huddart S. Use of a pathway quality improvement care bundle to reduce mortality after EL. BJS. 2015;102(1):57

**Grant**

Nil received

#### A487 Dexmedetomidine for ventilated septic patients in ICU: a multicenter randomized controlled trial

##### K. Miyamoto^1^, Y. Kawazoe^2^, T. Morimoto^3^, T. Yamamoto^4^, A. Fuke^5^, A. Hashimoto^6^, H. Koami^7^, S. Beppu^8^, Y. Katayama^9^, M. Ito^10^, Y. Ohta^11^, H. Yamamura^12^, DESIRE (DExmedetomidine for Sepsis in ICU Randomized Evaluation) Trial Investigators

###### ^1^Wakayama Medical University, Department of Emergency and Critical Care Medicine, Wakayama, Japan; ^2^Tohoku University Hospital Emergency Center, Division of Emergency and Critical Care Medicine, Sendai, Japan; ^3^Hyogo College of Medicine, Department of Clinical Epidemiology, Nishinomiya, Japan; ^4^Osaka City University Graduate School of Medicine, Department of Trauma and Critical Care Medicine, Osaka, Japan; ^5^Osaka City General Hospital, Emergency and Urgent Medical Care Center, Osaka, Japan; ^6^Hyogo College of Medicine, Emergency and Critical Care Center, Nishinomiya, Japan; ^7^Saga University Hospital, Advanced Emergency and Critical Care Center, Saga, Japan; ^8^National Hospital Organization Kyoto Medical Center, Department of Emergency Medicine, Critical Care, Kyoto, Japan; ^9^Sapporo Medical University, Department of Emergency Medicine, Sapporo, Japan; ^10^Yamaguchi Grand Medical Center, Department of Anesthesiology, Yamaguchi, Japan; ^11^Hyogo College of Medicine, Division of General Medicine, Department of Internal Medicine, Nishinomiya, Japan; ^12^Hirosaki University Graduate School of Medicine, Department of Disaster and Emergency Medicine, Hirosaki, Japan

####### **Correspondence:** K. Miyamoto - Wakayama Medical University, Department of Emergency and Critical Care Medicine, Wakayama, Japan

**Introduction:** Dexmedetomidine was shown to improve 28-day mortality compared with lorazepam in a subgroup analysis of sepsis patients in the MENDS randomized controlled trial^1)^.

**Objectives:** This study aimed to demonstrate whether a sedation strategy using dexmedetomidine was superior to that without dexmedetomidine in septic patients, in terms of 28-day mortality and ventilator-free days within a 28-day period.

**Methods:** A multicenter randomized controlled trial was conducted to evaluate the dexmedetomidine-based strategy in septic patients in eight ICUs. We included adult septic patients who were expected to require mechanical ventilation for at least 24 hours. Patients were randomized to receive sedation strategy either with dexmedetomidine (DEX group) or without dexmedetomidine (non-DEX group). Additionally, fentanyl, propofol, and midazolam were used as required to achieve the sedation goal in both groups which was set to Richmond Agitation Sedation Scale 0 (calm) for daytime and −2 (light sedation) for nighttime.

**Results:** We included 201 patients (mean age 68.8 years), of whom 127 (63 %) were men. Mean Acute Physiology And Chronic Health Evaluation (APACHE) II score was 23. The baseline characteristics were similar between groups. In the DEX group, well-controlled sedation rate was higher (p = 0.002) and delirium rate was lower (p = 0.03) during ventilation compared with those in the non-DEX group. At 28 days, 19 of 100 patients (19 %) in the DEX group and 28 of 101 patients (28 %) in the non-DEX group had died (p = 0.14). The ventilator-free days in a 28-day period did not differ between the groups, at 22 days (interquartile range [IQR], 17–25) and 21 days (IQR, 15.5-24.5) (p = 0.49). In a predefined subgroup analysis that included 104 patients with APACHE II score of 23 or more, 28-day mortality in the DEX group was significantly lower than that in the non-DEX group (odds ratio 0.40, 95 % confidential interval 0.17-0.93).

**Conclusions:** A sedation strategy using dexmedetomidine compared with a strategy without dexmedetomidine induced well-controlled sedation in ventilated septic patients but did not significantly improve 28-day mortality and the number of ventilator-free days. Although more critically ill patients may benefit from a dexmedetomidine strategy, further studies are needed to confirm this result.

**References**

Pandharipande PP et al. Crit Care 2010; 14: R38.

**Grant acknowledgement**

The design of this study was supported in part by Hospira Japan who also provided a non-contractual research grant to Wakayama Medical University for start-up purposes. However, data collection, data management, statistical analyses, interpretation and manuscript preparation were conducted solely by the academic investigators without any support from pharmaceutical companies.

The Institute for Clinical Effectiveness, a non-profit academic research organization, supported the implementation of this study.

#### A488 Lower versus higher haemoglobin threshold for blood transfusion in septic shock: exploratory subgroup analyses of a randomised trial

##### **S.L. Rygård**^1^, L.B. Holst^1^, J. Wetterslev^2^, P.I. Johansson^3^, A. Perner^1^

###### ^1^University of Copenhagen, Rigshospitalet, Department of Intensive Care, København, Denmark; ^2^Copenhagen Trial Unit, Center for Clinical Intervention Research, Copenhagen, Denmark; ^3^University of Copenhagen, Rigshospitalet, Section for Transfusion Medicine, Copenhagen, Denmark

####### **Correspondence:** S.L. Rygård - University of Copenhagen, Rigshospitalet, Department of Intensive Care, København, Denmark

**Introduction:** Using a restrictive transfusion strategy appears to be safe in critically ill patients [1,2] but there may be subgroups of patients benefiting from transfusion at a higher haemoglobin level [3].

**Objectives:** We explored if subgroups of patients with septic shock had worse outcome when transfused at a lower versus a higher haemoglobin threshold.

**Methods:** By performing post-hoc analyses of the whole trial population of 998 patients from the Transfusion Requirements in Septic Shock (TRISS) trial [2], we investigated the intervention effect in patients with severe co-morbidity (chronic lung disease, haematological malignancy or metastatic cancer), patients who had undergone surgery (either elective or acute) and patients with septic shock as defined by the new definition of septic shock: lactate above 2 mmol/l and treatment with vasopressors [4].

**Results:** The baseline characteristics were mostly similar between the 2 intervention groups in the different subgroups. There were no differences in the intervention effect on 90-day mortality in patients with chronic lung disease (test of interaction *P =* 0.31), haematological malignancy (*P =* 0.47), metastatic cancer (*P* = 0.51), septic shock by the new definition (*P* = 0.20) or in those who had undergone surgery (*P* = 0.99), see Table.

**Conclusions:** In exploratory analyses of a randomised trial in patients with septic shock, we observed no mortality benefit in any subgroups of transfusion at a haemoglobin threshold of 90 g/dl vs. 70 g/dl.

**References**

1. Forminsky E, Putzu A, Monaco F et al. *Liberal transfusion strategy improves survival in perioperative but not in critically ill patients. A meta-analysis of randomized trials* BJA 2015, 115: 511–19

2. Holst LB, Haase N, Wetterslev J, et al*. Lower versus higher hemoglobin threshold for transfusion in septic shock* N Engl J Med 2014, 371: 1381–91

3. Vincent JL*, Which carries the biggest risk: Aneamia or blood transfusion?* Transfusion Clinique et Biologique 2015, 22, 148–50

4. Singer M, Deutschman CS, Seymour CW et al. *The third international consensus definitions for sepsis and septic shock (Sepsis-3)* JAMA 2016, 315:801–10

**Grant acknowledgement**

None.

**Fig. 31 (abstract A488).**
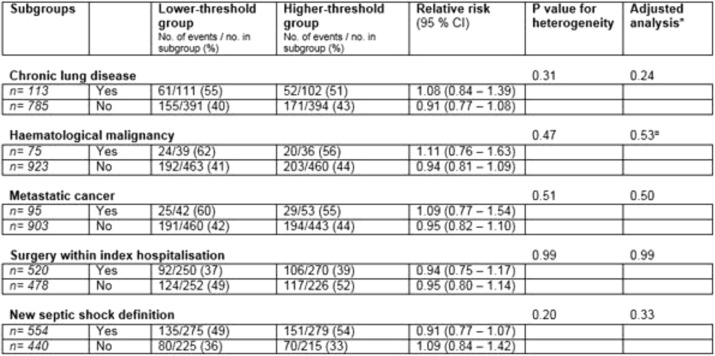
Results

### Factors affecting long-term ICU outcome

#### A489 Long-term ICU prognosis: how well do subjective ICU physician prognoses comply with the actual observed one-year outcomes?

##### I.W. Soliman^1^, D.W. de Lange^1^, D. van Dijk^1^, J.J.M. van Delden^2^, O.L. Cremer^1^, A.J.C. Slooter^1^, L.M. Peelen^1,3^

###### ^1^University Medical Center Utrecht, Department of Intensive Care, Utrecht, Netherlands; ^2^Julius Center for Health Sciences and Primary Care University Medical Center Utrecht, Department of Medical Humanities, Utrecht, Netherlands; ^3^Julius Center for Health Sciences and Primary Care University Medical Center Utrecht, Department of Epidemiology, Utrecht, Netherlands

####### **Correspondence:** I.W. Soliman - University Medical Center Utrecht, Department of Intensive Care, Utrecht, Netherlands

**Introduction:** At discharge from the Intensive Care Unit (ICU) physicians may integrate information of a patient's condition before ICU admission (medical history, functional status, quality of life) with the sequence of events during the ICU stay to estimate the prognosis of future well-being [1,2]. In 2006, the Sabadell group developed a simple but subjective prognostic score which made this prognosis explicit [1,2]. However, the Sabadell score has not been validated beyond hospital survival or for long-term health related quality of life (HRQoL).

**Objectives:** To compare ICU physicians' subjective long-term prognosis with the observed long-term survival and HRQoL.

**Methods:** The first admissions of all patients discharged alive from the ICU of the University Medical Center Utrecht between March 2012 and December 2014 were included in the study. The Sabadell score was determined and recorded by the treating physician at the time of ICU discharge ('0 - Good prognosis', '1 - Poor long-term prognosis (>6 months) with unlimited ICU readmission', '2 - Poor short-term prognosis (<6 months); ICU readmission debatable', or '3 - Death expected during hospitalisation, ICU readmission not recommended'). Survival status was verified using the municipal registry one year after ICU discharge. In one-year ICU survivors HRQoL was measured using the EuroQoL 5D-3L^TM^ index score, dichotomized at 0.4 into high or low HRQoL. The analyses included agreement between Sabadell score and one-year outcome categories, and a description of correctly and incorrectly estimated prognosis groups.

**Fig. 32 (abstract A489).**
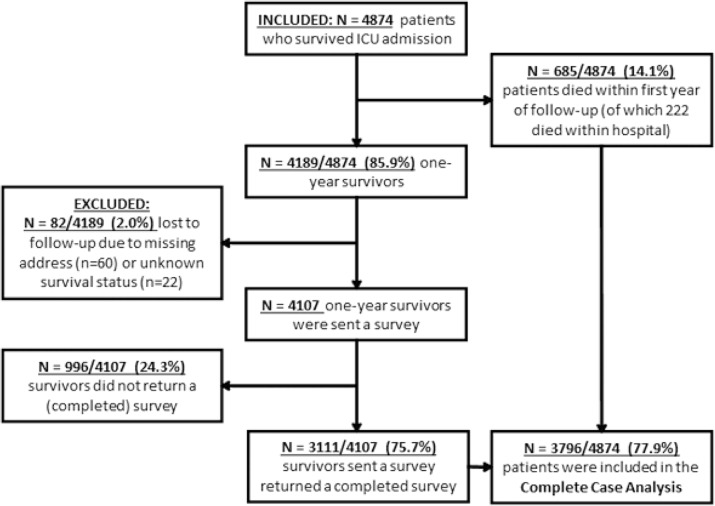
Flowchart

**Fig. 33 (abstract A489).**
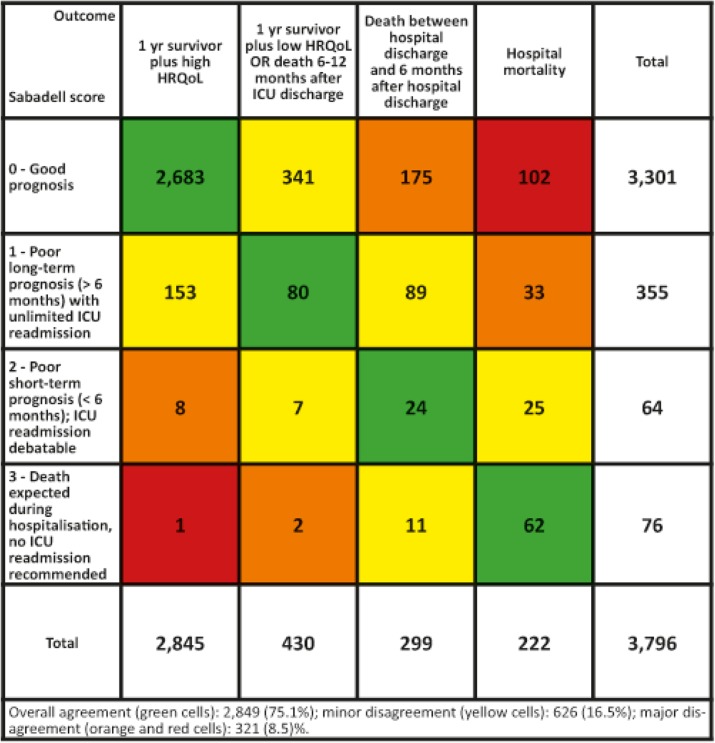
Agreement

**Results:** Of the 4,874 eligible patients, 3,796 (77.9 %) had completed follow-up and were included into the study (see Fig. 32). In-hospital mortality was 3.1 %, 9.3 %, 39.1 % and 81.6 % for the Sabadell scores 0–3 respectively. In 2,849/3796 (75.1 %) the Sabadell score accurately predicted one-year outcome (Fig. 33). Table 15 shows patient characteristics for the study population, and the groups where prognosis was over- and underestimated respectively. Compared to patients with a correct estimate of prognosis, both patient groups with over- or underestimated prognoses were more often admitted for non-surgical reasons, and more severely ill at admission and during ICU stay.

**Conclusions:** In three out of four patients, the Sabadell score was in accordance with one-year prognosis. Only in less than 10 % of patients surviving ICU there was major disagreement between a physician's prediction and the observed long-term outcome.

**References**

1. Fernandez R et al. Crit Care. 2006;10(6):R179

2. Fernandez R et al. Intensive Care Med. 2010;36(7):1196–201

**Grant acknowledgement**

This study was supported by the NutsOhra Foundation, project nr 1404–013

**Table 15 (abstract A489):** Patient characteristics

	Total population	Prognosis correctly estimated	Prognosis overestimated	Prognosis underestimated	p-value
N	3,796	2,849 (75.1%)^a^	765 (20.2%)^a^	182 (4.8%)^a^	--
Sex (male)	2,405 (63.4%)	1,833 (64.3%)	459 (60.0%)	113 (62.1%)	.081
Age at ICU admission	65 (55–73)	65 (55–72)	67 (58–75)	65 (53–73)	<.001
Admission type -					<.001
Elective surgical	2,317 (61.1%)	1,951 (68.5%)	302 (39.6%)	64 (35.4%)	
Urgent surgical	593 (15.6%)	401 (14.1%)	160 (21.0%)	32 (17.7%)	
Medical	881 (23.2%)	495 (17.4%)	301 (39.4%)	85 (47.0%)	
APACHE IV score	44 (33–60)	41 (31–54)	55 (41–76)	58 (42–81)	<.001
Total maximum SOFA score^b^	5 (3–8)	5 (3–8)	6 (4–10)	8 (4–11)	<.001
SOFA score at discharge	4 (3–6)	4 (3–6)	4 (3–6)	4 (3–6)	<.001
ICU length of stay	1 (1–2)	1 (1–2)	1 (1–4)	3 (1–8)	<.001

Continuous variables are presented as median (interquartile range) and tested for differences using a Kruskal Wallis test, categorical variables are presented as n (percentage) and tested for differences using a Chi-square test

*ICU* intensive care unit, *LoS* length of stay, *APACHE IV* acute physiology and chronic health evaluation fourth edition, *SOFA* sequential organ failure assessment

^a^percentage reflects proportion of total sample size

^b^Sum of highest SOFA component scores during admission

#### A490 Impact of an enhanced rehabilitation quality improvement project on long term survival of mechanically ventilated patients

##### D. McWilliams^1^, C. Snelson^2^

###### ^1^University Hospitals Birmingham NHS Trust, Critical Care, Birmingham, UK; ^2^University Hospital Birmingham, Critical Care, Birmingham, UK

####### **Correspondence:** C. Snelson - University Hospital Birmingham, Critical Care, Birmingham, UK

**Introduction:** Early and enhanced rehabilitation of patients in critical care is associated with improved patient outcomes (1). We have previously published the results of a quality improvement project promoting early and enhanced rehabilitation for patients mechanically ventilated for greater than 5 days, and shown an improvement in mobility levels at ICU discharge that was associated with a significant reduction in ICU and hospital length of stay, ventilator days and in-hospital mortality (2). The impact of such mobility programs on longer term outcomes is unknown.

**Objectives:** To assess the 3 year mortality of patients mechanically ventilated for more than 5 days and who survived to ICU discharge pre and post a quality improvement project enhancing rehabilitation on critical care.

**Methods:** The study took place within a large, tertiary referral ICU. A supportive rehabilitation team was created within the ICU in April 2012 with a focus on promoting early and enhanced rehabilitation for patients at high risk for prolonged ICU and hospital length of stays. The intervention has been described in detail in a previous publication, and was successful in improving mobility levels at ICU discharge. For all patients who survived to ICU discharge, mortality at 3 years was assessed.

**Results:** The mean age of the total court was 53 years. A total of 201 patients in the pre-quality improvement (Pre QI) group and 222 patients in the post quality improvement (Post QI) were discharged from ICU alive. The 3 year mortality rate was 81/201 (40.3 %) in the Pre QI group vs 60/222 (27 %) in the Post QI group; p = 0.004.

**Conclusions:** The introduction of early and enhanced rehabilitation within the ICU was associated with a significant reduction in 3 year mortality of ICU survivors. This hypothesis generating data warrants further evaluation in future randomized controlled trials of the intervention.

**References**

1. Hashem, M et al. Early mobilisation and rehabilitation of the critically ill patient. *Chest available online March 2016*

2. McWilliams et al. (2015) Enhancing rehabilitation of mechanically ventilated patients in the intensive care unit: a quality improvement project. *J of Crit Care 30; 8–13*

**Grant acknowledgement**

We are grateful to Queen Elizabeth Hospital Birmingham Charity for their support with this project

#### A491 Epidemiological, clinical characteristics and determinants of 1-year mortality after ICU discharge. The Caviuci study (evaluation of quality of life after ICU in Argentine)

##### A. Das Neves^1^, C.I. Loudet^1^, M. Busico^2^, D. Vazquez^3^, D. Villalba^4^, M. Veronesi^5^, A. Lischinsky^6^, F.J.L. López^7^, L. Benito Mori^8^, G. Plotnikow^3^, A. Díaz^9^, S. Giannasi^10^, R. Hernandez^11^, L. Krzisnik^12^, C. Cecotti^13^, L. Viola^14^, R. Lopez^15^, J.P. Sottile^16^, G. Benavent^17^, E. Estenssoro^1^

###### ^1^HIGA Gral San Martin, La Plata, Argentina; ^2^Hospital de Trauma F Abete, Municipio Islas Malvinas, Buenos Aires, Argentina; ^3^Sanatorio Anchorena, Buenos Aires, Argentina; ^4^Hospital Municipal Chivilcoy, Chivilcoy, Argentina; ^5^Instituto Fleni, Escobar, Argentina; ^6^Instituto De Neurología Cognitiva-INECO, Buenos Aires, Argentina; ^7^Hospital Escuela de Agudos Dr. Ramón Madariaga, Posadas, Argentina; ^8^HIGA Prof. Dr. Luis Güemes, Haedo, Argentina; ^9^Hospital Misericordia, Cordoba, Argentina; ^10^Hospital Italiano Buenos Aires, Buenos Aires, Argentina; ^11^Hospital Francisco López Lima, Gral Roca, Argentina; ^12^Alta Complejidad en Red, Hospital El Cruce, Dr. Néstor Carlos Kirchner, Florencio Varela, Argentina; ^13^HZGA Mi Pueblo Florencio Varela, Florencio Varela, Argentina; ^14^Hospital Español, Mendoza, Argentina; ^15^Clinica Pueyrredon, Mar del Plata, Argentina; ^16^Hospital Zonal Bariloche, Bariloche, Argentina; ^17^Clinica CEMEP, Ushuaia, Argentina

####### **Correspondence:** A. Das Neves - HIGA Gral San Martin, La Plata, Argentina

**Introduction:** Admission to the ICU deeply impacts on post-ICU mortality and quality of life, but the information on the issue is still scarce(1).

**Objectives:** To analyze epidemiological data and evolution of patients on mechanical ventilation (MV) in the ICU and to define independent variables associated to long-term (1 year) mortality.

**Methods:** National, prospective, multicenter cohort study sponsored by the Argentinian Society of Intensive Care Medicine, including patients receiving MV >72 hr (3/5-8/31/2014). Epidemiological and evolution variables were recorded, and after hospital discharge patients/their relatives were interviewed face-to-face or by telephone at 2, 6 and 12 months. Comparisons were made between survivors (S) and non-survivors (NS). Data are analyzed according to their nature; a p < .05 was considered significant. Independent predictors of post-ICU mortality were identified by logistic regression.

**Results:** 320 patients from 26 hospitals across Argentina were included. The data are shown on Tables 16, 17 and 18.

Global post-ICU mortality was 22 %; respectively 15 %, 5 % and 2 % at 2,6, and 12 months post hospital discharge. Independent predictors of mortality were: age (OR 1.07[.04-1.08;]; p 0.000) and SOFA_24hs_ (OR 1.12[1.02-.1.23];p 0.013). Calibration and discrimination of the model were adequate (GOF 0.407; AUROC 0.79, respectively).

**Conclusions:**

1. Most patients were middle-aged men, admitted to public hospitals for medical causes, 60 % with ≥1 comorbidities and very acutely ill, according to APACHE II and SOFA scores.

2. Complications as ARDS, shock and infections were frequent, occurring in more than a half of patients.

3. 1-year mortality was 22 %. Most patients died within two months after hospital discharge. Yet no single ICU event was associated with post-ICU mortality; only age and organ failures were independent predictors of this outcomes.

**Grant acknowledgement**

This study was supported by de Argentinian Society of Intensive Care (SATI).

**Table 16 (abstract A491).** Characteristics of Patients

	All	Survivors	Non Survivors	p
Age	50[29–66]	41[25–60]	66[53–74]	0.000
Male gender	184(58%)	118(57%)	59(41%)	0.79
Marital status Married/cohabiting Single Widower	153(48%)/ 133(43%)/ 34(11%)	46%/ 47%/ 8%	53%/ 29%/ 18%	0.005 Widower vs single P<0.001
Diagnostic category Medical Scheduled surgery Emergency surgery Trauma Trauma + Cranial trauma	51%/ 17%/ 8%/ 8%/ 15%	47%/ 18%/ 8%/ 9%/ 19%	66%/ 13%/ 9%/ 7%/ 6%	0.025
Occupational status Occupied (any type of employment/study)	51%	62%	40%	0.000
Years of study	9±4	9±4	9±4	0.45
Health Insurance	60%	57%	70%	0.05
Admission to public hospitals	65%	68%	56%	0.06
Transferred from Emergency Ward Home Operating room Another Hospital	49%/ 15%/ 1%/ 18%/ 17%	49%/ 13%/ 1%/ 19%/ 18%	49%/ 21%/ 3%/ 16%/ 11%	0.20

**Table 17 (abstract A491).** Severity Scores

	All	Survivivors	Non survivors	p
Charlson score 0 1 ≥ 2	118(37%) 72(22%)/ 130(41%)/	43% 21%/ 36%/	21% 31%/ 46%/	0.008
APACHE II	18±7	17±7	20±8	0.004
SOFA24hs	7±4	6±3	8±3	0.007
Admission Glasgow	14[8–15]	14[9–15]	14[8–15]	0.64

**Table 18 (abstract A491).** Events during ICU Stay

	All	Survivors	Non Survivors	p
Shock at admission	51%	49%	59%	0.15
Shock on evolution	57%	55%	63%	0.27
Duration of shock (days)	3[2–6]	3[2–5]	3[2–4]	0.3
ARDS	45%	48%	36%	0.07
Ventilator-associated pneumonia	26%	29%	19%	0.09
Catheter-related infection	41%	40%	43%	0.71
Length of MV(days)	10[6–19]	10[6–19]	10[6–19]	0.85
ICU stay (days)	16[10–26]	15[9–24]	18[11–27]	0.07
Hospital stay (days)	29[17–52]	28[16–52]	42[21–57]	0.06

#### A492 The outcomes of patients with severe dengue admitted to intensive care units

##### C.-M. Chen^1,2^, C.-C. Lai^3^, K.-C. Cheng^4^, W. Chou^1^, K.-S. Chan^2^

###### ^1^Chia Nan University of Pharmacy and Science, Recreation and Health-Care Management, Tainan, Taiwan, Province of China; ^2^Chi-Mei Medical Center, Intensive Care Medicine, Tainan, Taiwan, Province of China; ^3^Chi-Mei Medical Center, Liouying District, Intensive Care Medicine, Tainan, Taiwan, Province of China; ^4^Chi Mei Medical Center, Internal Medicine, Tainan, Taiwan, Province of China

####### **Correspondence:** C.-M. Chen - Chi-Mei Medical Center, Intensive Care Medicine, Tainan, Taiwan, Province of China

**Introduction:** To survey the outcome of patients with dengue infection admitted to intensive care unit (ICU).

**Objectives:** Because the outcome of adult patients with dengue infections requiring ICU admissions remains unclear, this study was conducted to assess the clinical manifestations and the prognostic factors of critical ill patients with severe dengue.

**Methods:** This study was conducted in a tertiary referral hospital of 96 adult ICU beds. In this retrospective study, all of the patients with laboratory-confirmed severe dengue infections and admitted to ICU were enrolled between July 31 and November 31 in 2015 during the large outbreak period. The medical records of all the recruited patients were retrospectively reviewed and the following information was collected: age, gender, clinical manifestations, disease severity scores, underlying conditions, laboratory examinations, and outcome. The primary endpoint is to find the predictors of in-hospital mortality.

**Results:** During study period, there were a total of 4787 patients with dengue infections, and 143 (2.99 %) patients with severe dengue infection required ICU admission. Among 143 critically ill patients, their mean age was 69.7 years. Hypertension (n = 90, 62.9 %) and diabetes mellitus (n = 70, 49.0 %) were the two most common underlying diseases. Eighty patients (55.9 %) had co-bacterial infections, and 33 patients had co-bacteremia. Hematologic system was the most common failure organ, followed by chest and cardiovascular systems. Fever was the most common presentation (n = 112, 78.3 %), followed by anorexia (n = 47, 32.9 %), and abdominal pain (n = 46, 32.2 %). Overall, a total of 33 patients had mortality, and the rate of mortality was 23.1 %. In multivariate analysis, we found that in-hospital mortality was significantly associated with lower Glasgow coma scale, lower platelet count before ICU discharge, and higher number of organ failures.

**Conclusions:** The outcome of severe dengue patients requiring ICU admission remains high and the mortality was associated with lower Glasgow coma scale, lower platelet counts and more organ failures. Additionally, more than half of patients would have bacterial infections during severe dengue infections.

**References**

1.Murray NE, Quam MB, Wilder-Smith A: **Epidemiology of dengue: past, present and future prospects**. *Clinical epidemiology* 2013, **5**:299–309.

2.Guzman MG, Harris E: **Dengue**. *Lancet (London, England)* 2015, **385**(9966):453–465.

**Grant acknowledgement**

no

#### A493 Eosinopenia in ICU survivors and post-hospital outcomes

##### L.E. Roeker^1^, C.M. Horkan^1^, F.K. Gibbons^2^, K.B. Christopher^3,4^

###### ^1^Brigham and Women's Hospital, Department of Medicine, Boston, MA, USA; ^2^Massachusetts General Hospital, Pulmonary and Critical Care Medicine, Boston, MA, USA; ^3^Brigham and Women's Hospital, Renal Division, Boston, MA, USA; ^4^Brigham and Women's Hospital, Channing Division of Network Medicine, Boston, MA, USA

####### **Correspondence:** L.E. Roeker - Brigham and Women's Hospital, Department of Medicine, Boston, MA, USA

**Introduction:** Eosinopenia is associated with short term adverse outcomes in the critically ill. In survivors of critical care, it is not know if eosinopenia is predictive of adverse outcomes following hospital discharge.

**Objectives:** We hypothesized that eosinopenia at ICU admission would be associated with increased hospital readmission rates and higher mortality following hospital discharge.

**Methods:** We performed a two center observational study of patients treated in medical and surgical intensive care units. We studied 68,648 patients, age ≥ 18 years, who received critical care between 1998 and 2012 and survived hospitalization. The exposure of interest was absolute eosinophil count within 48 hours of ICU admission and categorized a priori as ≤10/ul, 10-50/ul, 50-350/ul (normal range) and >350/ul. The primary outcome was all cause mortality in the 90 days following hospital discharge determined using the US Social Security Administration Death Master File. 365-day follow-up was present in all cohort patients. Adjusted odds ratios were estimated by multivariable logistic regression models with inclusion of covariate terms for age, race, gender, Deyo-Charlson Index, patient type (medical versus surgical), sepsis and number of organs with acute failure.

**Results:** The cohort patients were 57.8 % male, 20.3 % nonwhite and 48.8 % surgical. 10.1 % of the cohort had sepsis, and the mean age was 61.7 years. Median [IQR] absolute eosinophil count was 90 [30–190]. 90-day post discharge mortality was 7.7 %. 30-day readmission rate was 14.3 %. A decreased absolute eosinophil count was a robustly associated with all-cause post discharge mortality as well as hospital readmission and discharge to a care facility instead of home. Patients with an absolute eosinophil count of ≤10/ul or 10-50/ul have an adjusted OR of 90-day post-discharge mortality of 1.58 (95%CI, 1.46-1.71; P < 0.001) or 1.49 (95%CI, 1.38-1.60; P < 0.001) relative to patients with an absolute eosinophil count of 50-350/ul. Patients with an absolute eosinophil count of ≤10/ul or 10-50/ul have an adjusted OR of 30-day readmission of 1.08 (95%CI, 1.01-1.15; P = 0.018) or 1.10 (95%CI, 1.04-1.17; P = 0.001) relative to patients with an absolute eosinophil count of 50-350/ul. Finally, patients with an absolute eosinophil count of ≤10/ul or 10-50/ul have an adjusted OR of discharge to a care facility of 1.34 (95%CI, 1.28-1.41; P < 0.001) or 1.30 (95%CI, 1.24-1.36; P < 0.001) relative to patients with an absolute eosinophil count of 50-350/ul. An absolute eosinophil count >350/ul was not associated with study outcomes.

**Conclusions:** In critical illness survivors, eosinopenia at ICU admission is a robust predictor of mortality following discharge, hospital readmission, and discharge to a care facility. Eosinopenia is a marker for ICU survivors at an especially high risk for adverse outcomes. Thus, patients with eosinopenia may benefit from closer post discharge follow-up and higher intensity rehabilitation.

### Nutritional aspects in the ICU

#### A494 Protein intake, nutritional status and outcomes in ICU survivors

##### P.J.M. Weijs^1,2^, K.M. Mogensen^3^, J.D. Rawn^4^, M.K. Robinson^4^, K.B. Christopher^5,6^

###### ^1^VU University Medical Center Amsterdam, Department of Nutrition and Dietetics, Internal Medicine, Amsterdam, Netherlands; ^2^Amsterdam University of Applied Sciences, Amsterdam, Netherlands; ^3^Brigham and Women's Hospital, Department of Nutrition, Boston, USA; ^4^Brigham and Women's Hospital, Department of Surgery, Boston, USA; ^5^Brigham And Women's Hospital, Renal Division, Boston, USA; ^6^Brigham and Women's Hospital, Channing Division of Network Medicine, Boston, USA

####### **Correspondence:** P.J.M. Weijs - Amsterdam University of Applied Sciences, Amsterdam, Netherlands

**Introduction:** Critical illness is marked by hypermetabolism and increased protein catabolism. While studies suggest that protein delivery may be beneficial for critical illness outcomes, to date, limited information exists regarding the association between protein delivery during hospitalization and outcomes in ICU survivors following hospital discharge.

**Objectives:** We hypothesized that higher protein intake might have a protective effect in patients with malnutrition.

**Methods:** We performed a single center observational study of patients treated in medical intensive care units in Boston, Massachusetts. We studied 801 patients age ≥ 18 years, who received critical care following between 2004 and 2011 and survived to hospital discharge. All patients underwent a Registered Dietitian formal assessment within 48 hours of ICU admission. The exposure of interest, grams of protein per kilogram body weight delivered per day, was determined from all oral, enteral and parenteral sources for up to 28 days. Nutrition status was categorized as non-specific malnutrition, protein-energy malnutrition, or at risk for malnutrition via anthropometric measurements, clinical signs of malnutrition, malnutrition risk factors, and metabolic stress. The primary outcome was all cause 90-day post-discharge mortality. Adjusted odds ratios were estimated by mixed- effects logistic regression models to describe how 90-day post-discharge mortality differed with changes in protein delivery.

**Results:** The cohort was 55 % male, 79 % white with a mean age of 62.3 years. 22 % of the cohort had sepsis, 10 % had acute kidney injury and 35 % had non-cardiac acute respiratory failure. 59 % had non-specific malnutrition or protein-energy malnutrition. The 30, 90 and 365-day post-discharge mortality was 7.1 and 13.9 and 24.4 %. The average number of nutrition delivery days recorded was 15. In a mixed-effect logistic regression model adjusted for age, gender, race, Deyo-Charlson Index, acute organ failures, sepsis and percent energy needs met, 90-day post-discharge mortality rate was 17 % (95%CI: 6–26) lower for each 1 g/kg increase in daily protein delivery (*P* = 0.002) compared with the 90-day post-discharge mortality rate in the entire cohort [OR = 0.83 (95%CI 0.74-0.94; P = 0.002)]. In a subset of patients with malnutrition (n = 473): 90-day post-discharge mortality rate was 30 % (95%CI: 6–26) lower for each 1 g/kg increase in daily protein delivery (P < 0.001) compared with the 90-day post-discharge mortality rate in the entire cohort [OR = 0.70 (95%CI 0.61-0.81; P < 0.001)].

**Conclusions:** In adult MICU patients who survive to hospital discharge, protein intake appears to be predictive of out of hospital outcomes. Patients with improvements in protein intake during hospitalization independent of energy intake have decreased mortality in the 3 months following hospital discharge. The achievement of ideal protein delivery may be an important factor in ICU survivorship, especially in malnutrition.

#### A495 Safety and efficacy of a new parenteral lipid emulsion (SMOFlipid) in surgical critically ill patients

##### Z. Tang, C. Qiu, B. Ouyang, C. Cai, X. Guan

###### The First Affiliated Hospital of Sun Yat-Sen University, Guangzhou, China

####### **Correspondence:** Z. Tang - The First Affiliated Hospital of Sun Yat-Sen University, Guangzhou, China

**Introduction:** SMOFlipid 20 % is intravenous lipid emulsion (ILE) containing long-chain triglycerides (LCT), medium-chain triglycerides(MCT), olive oil, and fish oil as a mixed emulsion containing α-tocopherol. The aim was to assess the efficacy of this new ILE in surgery compared with MCT/LCT,and it was tested for safety, tolerance, metabolic and clinical efficacy in surgical patients.

**Objectives:** To assess the efficacy of this new ILE in gastrointestinal surgery compared with MCT/LCT.

**Methods:** In this prospective study, 42 patients were randomized to SMOFlipid 20 % or MCT/LCT (Lipovenoes 20 %) group. Clinical and biochemistry data were collected. Inflammatory markers (CRP, IL-6) and liver function indicators (ALT,AST,TBIL)were measured.

**Results:** 32 patients (17 males and 15 females) with a mean age of 51 years completed the study. The patients' baseline datas(age, gender, APACHE II) were similar in two groups(*p* > 0.05). The patients' baseline datas(age, gender, APACHE II)were similar in two groups.The increment of triglyceride on day 5 from baseline was significantly lower in SMOFlipid group than in Lipovenoes MCT/LCT group(*P* < 0.05). The concentrations of alanine transaminase (ALT), aspartate transaminase (AST) and totalbilirubin on day 5 were significantly lower in SMOFlipid group than in control group(*P* < 0.05). Inflammatory markers (CRP, IL-6)were not different between groups(*P* > 0.05).

**Conclusions:** SMOFlipid had a better triglyceride-lowering effectas compared with MCT/LCT and may be associated with a better liver tolerance in surgical critically ill.

**References**

1. Burrin DG, Ng K, Stoll B, et al. Impact of new-generation lipid emulsions on cellular mechanisms of parenteral nutrition-associated liverdisease. Adv Nutr, 2014,5(1):82–91.

2. Wu MH, Wang MY, Yang CY,et al.Randomized clinical trial of new intravenous lipid (SMOFlipid 20 %) versus medium-chain triglycerides/long-chaintriglycerides in adult patients undergoing gastrointestinal surgery,JPEN J Parenter Enteral Nutr, 2014,38(7):800–8.

#### A496 Dexmethasone-induced muscular atrophy is mediated by the expression of functional connexin based hemichannels

##### T. Regueira^1^, L. Cea^2^, S. Juan Carlos^3^, B. Elisa^3^, C. Puebla^3^, A. Vargas^3^

###### ^1^Clínica Las Condes, Medicina Intensiva, Santiago, Chile; ^2^Universidad de Chile, Program of Anatomy and Developmental Biology, Institute of Biomedical Sciences, Santiago, Chile; ^3^Universidad Católica de Chile, Departamento de Fisiología, Santiago, Chile

####### **Correspondence:** T. Regueira - Clínica Las Condes, Medicina Intensiva, Santiago, Chile

**Introduction:** Muscle atrophy is one of the most important and frequent problems observed in patients in Intensive Care Units (ICU). Several mechanisms and risk factors have been described to explain this syndrome. Glucocorticoids, which are frequently used in ICU, induce muscle atrophy, but the mechanism still remains to be elucidated. Connexin based hemichannels (Cx HCs), are channels formed by six connexin proteins and communicates the intra with extra cellular space since they are permeable to ions and small molecules (e.g., ATP). Recently, CxHCs have been associated to denervation-induced skeletal muscle atrophy (1), and may also participate in the pathogenesis of corticoids induced muscle atrophy.

**Objectives:** To evaluate the possible involvement of Cx HCs in dexamethasone-induced muscular atrophy.

**Methods:** C57Bl/6 wild type (WT) mice and Cx HCs knock out (KO) mice (Cx43fl/flCx45fl/fl and Cx43fl/flCx45fl/fl:Myo-Cre) mice were used. KO mice were skeletal muscle deficient for connexin 43 and 45. Also, Lanthanum, a connexin blocker, was used in experiments. Presence and functionality of Cx HCs was evaluated acutely after 5 hours of dexamethasone exposure, by cellular Ethidium uptake, Evans blue permeability, intracellular free Ca2+, resting membrane potential, immunohistochemistry, and western blot. NFkb Phosphorylation and mRNA levels of TNF-alpha were also assessed. Finally, muscle atrophy was evaluated by cross sectional area (CSA) after 7 days of dexamethasone exposure. Also, atrogin-1 and MurF-1, which are two E3 ligases from ubiquitin-proteasome system (intracellular degradation system), levels were evaluated.

**Results:** Freshly isolated myofibers from mice treated with dexamethasone by 5 h showed de novo expression of functional connexins 39, 43, 45 and pannexins (P2X7R) which were mainly distributed on sarcolemma. Cx HCs expression was present only in WT myofibers, was blocked by La, and was absent in KO mice. Accordingly, a significant increase in basal intracellular free Ca2+ levels, and a decrease of resting membrane potential of about 10 mV, was observed in myofibers from WT mice treated with dexamethasone compared with mice injected with saline buffer or in KO mice. Moreover, dexamethasone induced the phosphorylation of NFkb and increased the mRNA levels of TNF-alpha. Finally, seven days treatment with dexamethasone drastically reduced the CSA of myofibers from WT mice but not KO animals. Both, atrogin-1 and MurF-1 were elevated in WT animals in comparison with KO mice.

**Conclusions:** Dexamethasone induces the expression of Ca2+ permeable channels such as connexins, which leads to muscle atrophy. Connexins are upstream to the activation of the ubiquitin-proteasome system. Connexins are a novel family of proteins which should be studied for prevention of corticoids-induced muscle atrophy.

**References**

1. Cea et al. Proc Natl Acad Sci USA 2013,110(40).

**Grant acknowledgement**

Fondecyt 1141092

#### A497 Comparison of two indirect calorimetry devices at varying levels of inspired oxygen

##### M.K. Poulsen^1^, L.P. Thomsen^1^, S. Kjærgaard^2^, S.E. Rees^1^, D.S. Karbing^1^

###### ^1^Aalborg University, Respiratory and Critical Care Group (Rcare), Department of Health Science and Technology, Aalborg, Denmark; ^2^Aalborg University Hospital, Department of Anesthesiology, Aalborg, Denmark

####### **Correspondence:** S.E. Rees - Aalborg University, Respiratory and Critical Care Group (Rcare), Department of Health Science and Technology, Aalborg, Denmark

**Introduction:** Malnutrition increases morbidity, length of hospitalization and mortality in ICU patients (1), stressing the importance of energy expenditure (EE) assessment. Indirect calorimetry is the most accurate method, but available calorimeters do not support EE measurement at inspired oxygen (FiO_2_) >80 %, preventing measurement in the most severely ill. The Beacon Caresystem (Mermaid Care, Nørresundby, Denmark) for advising on mechanical ventilation includes indirect calorimetry designed for use in the full FiO_2_ range, but has not been compared to other calorimeters.

**Objectives:** To compare the Beacon Caresystem to the E-COVX (Datex-Ohmeda, GE Health Care, Helsinki, Finland) at varying FiO_2_.

**Methods:** Twenty healthy male subjects were included. Informed consent and ethical approval were obtained in all cases. Measurements from 16 subjects (age 32 ± 12 yrs) were analysed, the remainder excluded due to gas leakage. Subjects were mechanically ventilated by mask (Servo-I, Maquet, Solna, Sweden) in pressure support (PS: 3 cm H_2_O, PEEP: 3 cm H_2_O) at FiO_2_ of 21, 50 and 85 %, in that order, for 15 min at each FiO_2_. Analysis periods were selected where COV for average oxygen consumption (VO_2_) and CO_2_ production (VCO_2_) were < 5 % in five consecutive 1 min intervals (2). VO_2_, VCO_2_ and EE were compared between devices (Beacon - E-COVX) at each FiO_2_ by Bland-Altman analysis of bias and limits of agreement (LOA) and within devices between 21 and 85 % FiO_2_ by paired t-tests.

**Results:** VO_2_, VCO_2_ and EE bias [LOA] between devices were −27 [−73 - 19] ml/min, −5 [−28 - 18] ml/min, and −157 [−434 - 121] kcal/day at 21 % FiO_2_; −5 [−50 - 50] ml/min, 6 [−22 - 34] ml/min, and −12 [−345 - 320] kcal/day at 50 % FiO_2_; and 26 [−87 - 139] ml/min, 50 [16–84] ml/min, and 327 [−179 - 834] kcal/day at 85 % FiO_2_. Analysis was performed in 12 subjects at 85 % FiO_2_ as E-COVX was unable to measure EE in 4 subjects. Mean ± SD changes in VO_2_, VCO_2_ and EE within device from 21 to 85 % as percentage of mean were 11 ± 6 % (p < 0.001), 3 ± 6 % (p = 0.11) and 9 ± 6 % (p < 0.001) for Beacon Caresystem, and −4 ± 14 % (p = 0.415), −16 ± 10 % (p < 0.001) and −9 ± 12 % (p = 0.017) for E-COVX.

**Conclusions:** Measurements of VO_2_, VCO_2_ and EE were comparable between Beacon Caresystem and E-COVX at 21 and 50 % FiO_2_. Significant differences were observed at 85 % FiO_2_ where E-COVX slightly underestimated VCO_2_ and EE and Beacon Caresystem slightly overestimated VO_2_ and EE. Only Beacon Caresystem allowed EE measurement at 85 % FiO_2_ in all 16 subjects.

**References**

1. Goiburu ME et al. The impact of malnutrition on morbidity, mortality and length of hospital stay in trauma patients. Nutr Hosp. 2006;21(5):604–10.

2. Frankenfield D et al. Validation of a 5-minute steady state indirect calorimetry protocol for resting energy expenditure in critically ill patients. J Am Coll Nutr. 1996;15(4):397–402.

**Grant acknowledgement**

DSK and SER are minor shareholders and perform consultancy for Mermaid Care.

#### A498 Measuring resting energy expenditure in patients with veno-venous extracorporeal membrane oxygenation as a necessary tool to guarantee goal directed feeding (MEEP)

##### T. Wollersheim^1,2^, S. Frank^1^, M.C. Müller^1^, N.M. Carbon^1^, V. Skrypnikov^1^, P.A. Pickerodt^1^, R. Falk^1^, A. Mahlau^1^, S. Weber-Carstens^1,2^

###### ^1^Charité - Universitaetsmedizin Berlin, Berlin, Germany; ^2^Berlin Institute of Health (BIH), Berlin, Germany

####### **Correspondence:** T. Wollersheim - Berlin Institute of Health (BIH), Berlin, Germany

**Introduction:** Acute respiratory distress syndrome (ARDS) is a common lung disease. Extracorporeal lung support (ECLS) can be an additional lifesaving tool. Patients with severe ARDS often suffer from a catabolic state with massive loss of weight including skeletal muscle and fat tissue. For ICU patients in general there is evidence, that goal directed feeding could improve outcome and indirect calorimetry (IC) is the gold standard to measure resting energy expenditure (REE). Due to the O2 uptake and CO2 removal by lung and ECLS, an IC in the common way is not feasible. So far it is unclear if and which impact ECLS has on caloric needs of patients with ARDS.

**Objectives:** We introduce our MEEP (Measuring Energy Expenditure in ECMO Patients) protocol, which enables us to determine the REE in patients with ECLS. We investigated the impact of ECLS in ARDS patients on REE.

**Methods:** We perform a common IC and extend it by a calculation of the O2 uptake and the CO2 elimination by the ECMO filter due to blood gas analyses (BGAs) and the ECMO blood flow [1]. Sum O2 uptake and CO2 elimination were used in the equation of Weir [2] to calculate REE. With this protocol we performed a monocentric, controlled, prospective, observational pilot study. We included 20 patients with ARDS and ECMO treatment and 20 matched ARDS patients without ECLS as control. Informed consent was given by legal proxy. The COSMED Quark RMR® was used to perform IC, BGAs were done by ABL Flex 800 and filter blood flow was measured by the ECMO device. For all patients we measured resting energy expenditure and calculated the most prevalent predicting equations for REE for comparison. Nonparametric tests were performed. Data were shown as median [IQR]. P-values ≤ 0.05 are assumed as being significant. Ethic vote(EA1/293/13).

**Results:** Patient´s baseline characteristics were without significant differences except SOFA Score at time-point of measurement for patients with ECMO 12.5 [8.0/14.5] and without ECMO 7.5 [6.0/9.5] (p = 0.002). We report that our MEEP-protocol is safe, easy and feasible to perform in ECMO patients. We show for the first time, that measured REE values according to our MEEP protocol do not significantly differ between ARDS patients with ECMO (2013 kcal/d [1786/2333]) compared to ARDS patients without ECMO (1857 kcal/d [1602/2085]) (p = 0.165). All investigated equations predicting REE do not fit the measured REE according to the MEEP protocol neither in non-ECMO patients (data not shown) nor in ECMO patients.

**Conclusions:** Due to our MEEP-protocol REE is easy measurable in ARDS patients with ECLS. ECMO per se does not to influence REE. Equations to estimate caloric needs do not fit the REE of critically ill patients with ECMO. We recommend to implement sequential measurements of REE in critically ill during ECMO treatment to improve goal directed feeding.

**Fig. 34 (abstract A498).**
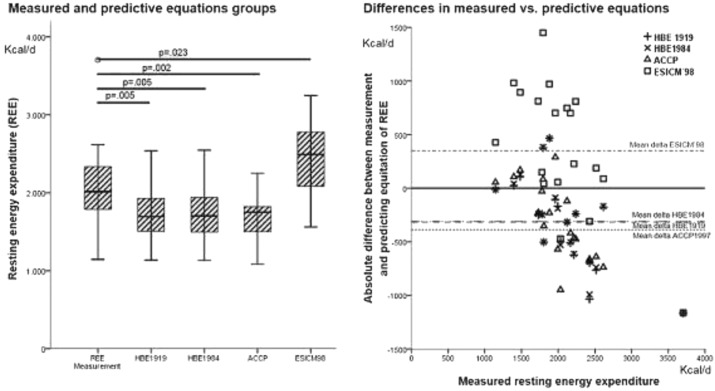
Resting energy expenditure in ECMO patients

### Trauma critical care

#### A499 CRASH-2 from the coal face - experience from a major trauma centre in the United Kingdom

##### A. Lee, R. Inglis, R. Morgan, G. Barker

###### John Radcliffe Hospital, Adult Intensive Care Unit, Oxford, UK

####### **Correspondence:** A. Lee - John Radcliffe Hospital, Adult Intensive Care Unit, Oxford, UK

**Introduction:** The administration of tranexamic acid to trauma patients with risk of haemorrhage is one of only seven treatments shown to improve mortality in critically ill patients in multi-centre randomized controlled trials [1,2]. Nonetheless, four years on from CRASH-2, the implementation of this simple, low risk intervention, which has the potential to save 600 lives a year in the UK, remains suboptimal [3].

**Objectives:** To measure the proportion of trauma patients deemed to be at risk of haemorrhage (as evidenced by the administration of a first dose of tranexamic acid) that correctly received the second dose of tranexamic acid. We designed a quality improvement project to improve compliance with this measure, and here we report preliminary Results:

**Methods:** All patients admitted under a 'trauma call' between January 2015 and January 2016 at the John Radcliffe Hospital in Oxford, a major trauma centre in the United Kingdom, were included in our study. Tranexamic acid administration information was collected for all patients. Interventions included disseminating tranexamic acid information to trauma doctors verbally and during departmental induction. We also added both doses of tranexamic acid into the electronic prescribing bundle for trauma patients in our hospital. Further interventions are ongoing.

**Results:** The John Radcliffe Hospital received 1373 patients as 'trauma calls' between January 2015 and January 2016. Of the 184 patients who received the first dose of tranexamic acid, 82 patients correctly received the second dose (45 %). We present a run-chart of percentage of patients receiving both doses of tranexamic acid over time, out of all the patients who received the first dose of tranexamic acid.

**Conclusions:** We have demonstrated that a majority of patients who would have received two doses of tranexamic acid under the terms of CRASH 2 are not receiving this life-saving treatment. This finding highlighted the difficulty in translating evidence-based recommendations into everyday practice and the need for quality improvement projects in this area. Responsibility for the administration of tranexamic acid lies across multiple specialties, and so measures to improve its administration need to target a diverse group of healthcare staff. Engaging stakeholders from all relevant parties has proved challenging and an early focus on inter-specialty collaboration is our key recommendation to centres who intend to implement similar initiatives.

**References**

1. Landoni G et al. Mortality in Multicenter Critical Care Trials: An Analysis of Interventions With a Significant Effect. Crit Care Med. 2015 Aug;43(8):1559–68.

2. Shakur H et al. Effects of tranexamic acid on death, vascular occlusive events, and blood transfusion in trauma patients with significant hemorrhage (CRASH-2): a randomized, placebo-controlled trial. The Lancet. 2010;376(9734):23–32.

3. Trauma Audit and Research Network (TARN) database.

**Fig. 35 (abstract A499).**
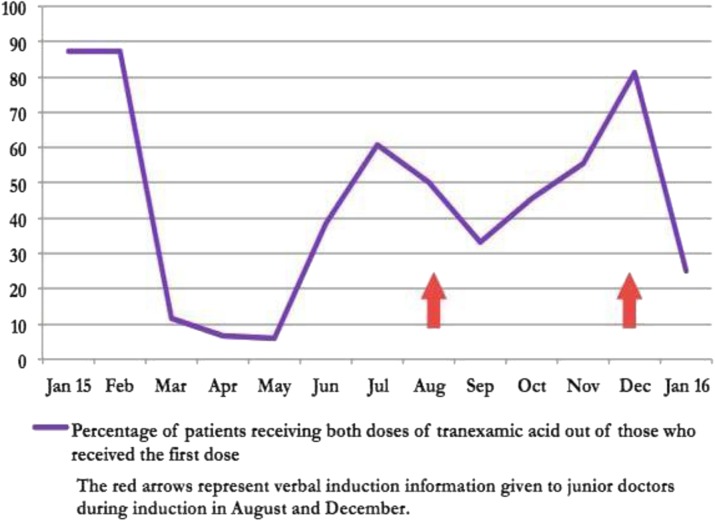
Run Chart TxA Audit Jan 2015 – Jan 2016

#### A500 Dynamic vital signs of patients with trauma and in-hospital mortality

##### K. Kamata^1^, T. Abe^2,3^, D. Saitoh^4^, Y. Tokuda^5^

###### ^1^Ministry of Health, Labour and Welfare, Government of Japan, Tuberculosis and Infectious Diseases Control, Tokyo, Japan; ^2^Tsukuba Medical Center Hospital, Emergency Medicine and Critical Care Medicine, Tsukuba, Japan; ^3^University of Tsukuba, Health Services Research, Tsukuba, Japan; ^4^National Defense Medical College, Traumatology and Emergency Medicine, Tokorozawa, Japan; ^5^Japan Community Healthcare Organization, Tokyo, Japan

####### **Correspondence:** K. Kamata - Ministry of Health, Labour and Welfare, Government of Japan, Tuberculosis and Infectious Diseases Control, Tokyo, Japan

**Introduction:** Emergency physicians are required to assess the severity of patient's illness and predict their prognosis with restricted situation at emergency department (ED). Trauma is one of the most severe injuries at ED and its outcome highly depends on initial appropriate management. Therefore, several scoring systems have been developed on the last decade. However, the environment on ED has changed and physicians have been still occasionally misled.

**Objectives:** Vital signs are widely used in both emergency medical service and ED. It is a role of not only the static parameter but also the dynamic one to help emergency physicians classify trauma patients' severity. Thus, we aimed to analyze the association between changes in vital sings (ΔVS) and in-hospital mortality among trauma patients.

**Methods:** This study was multicenter observational retrospective study from Japan Trauma Data Bank. It consisted of major emergency hospitals in Japan from January 2004 to December 2013. In this period, a total of 159,157 trauma patients were registered. Of these, 75,833 patients (48 %) were analyzed since all vital signs (systolic blood pressure, heart rate, and respiratory rate) were available both at site and at ED.

We developed a new scoring system to predict in-hospital mortality for trauma patients using three ΔVS and age. The patients were judged on these 4 items with one point added for each positive one; when systolic blood pressure decreased more than 30 mmHg, heart rate increased more than 20/min, respiratory rate increased 10/min, or old (age ≧65).

**Results:** Mean age (±SD) was 54 (±24) years old. 29,309 (39 %) were aged over 65 years old. 25,854 (34 %) were women. Overall, in-hospital mortality rate was 5,834 (7.7 %). The in-hospital mortality rate increased from 6.4 % to 18.9 % when systolic blood pressure decreased more than 30 mmHg. In a similar way, it increased from 7.0 % to 16.5 % when heart rates increased 20/min. Also, it increased from 7.5 % to 10.7 % when respiratory rates increase 10/min. Old patients (> = 65 years old) had severe outcome than young adults (it increased from 5.1 % to 11.8 %). As using our scoring system, in-hospital mortality rate increased: 0 point was 3.5 %; 1 point was 9.5 %; 2 points was 18.5 %; 3 points was 33.0 %; and 4 points was 48.6 %. The area under the ROC curve of this predictive model was 0.675 (95 % CI 0.668-0.683).

**Conclusions:** Our new trauma scoring system, ΔVS scoring system, which focus on changing vital signs, predicts in-hospital mortality. It is useful to improve that emergency physician's decision and identification of patients who need critical care.

**References**

1. Kondo et al. Critical Care 2011,15:R191

2. Mehmood et al. BMC Emergency Medicine 2015, 15(Suppl 2):S10

3. Gerdin et al. BMC Emergency Medicine(2016)16:15

**Grant acknowledgment**

This work was supported by JSPS KAKENHI Grant Number 16 K15388.

#### A501 Increased mortality in trauma patients who develop post-intubation hypotension

##### R.S. Green^1^, M.B. Butler^2^, M. Erdogan^1^

###### ^1^Dalhousie University, Nova Scotia Trauma Program, Halifax, Canada; ^2^Dalhousie University, Undergraduate Medical Education, Halifax, Canada

####### **Correspondence:** R.S. Green - Dalhousie University, Nova Scotia Trauma Program, Halifax, Canada

**Introduction:** Systemic hypotension is a predictor of increased mortality in patients with traumatic brain injuries. Post-intubation hypotension (PIH) has been demonstrated to be common and associated with poor patient outcomes in other critically ill patient populations requiring emergent endotracheal intubation (ETI). The importance of PIH remains unclear in the trauma population.

**Objectives:** To determine the incidence of PIH in adult trauma patients and assess the association of PIH with mortality.

**Methods:** This study was a retrospective case series of adult (≥16 years) trauma patients requiring intubation after referral to a provincial trauma team located in a level 1 center in Halifax, Nova Scotia, Canada between 2000 and 2015. PIH was defined as (a) decrease in systolic blood pressure (SBP) to < 90 mmHg; (b) reduction in SBP of 20 % from baseline; (c) decrease in mean arterial pressure to < 60 mmHg; or (d) initiation of, or increased infusion dosage of, any vasopressor medication (bolus or infusion) during the 15-minute period after ETI. Data was collected from a provincial trauma registry and the patient chart, and included demographics, co-morbidities, trauma characteristics, intubation time, as well as all fluids, medications, adverse events, interventions, and vital signs during the 15 minutes before/after ETI. We evaluated the incidence of PIH and created a logistic regression model to determine the likelihood of mortality in the PIH and non-PIH groups after controlling for gender, age, intubator, mechanism of trauma, injury severity, traumatic brain injury and volume of fluid administered.

**Results:** Overall, 477 patients arrived unintubated and required ETI by the trauma team over the study period, of which 444 patients met eligibility criteria and were included in the analysis. The incidence of PIH was 35.6 % (158/444) in our study population. In-hospital mortality occurred in 47 (29.7 %) in the PIH group, compared to 45 (15.7 %) in the non-PIH group. After controlling for possible confounding factors, the development of PIH was associated with increased mortality (OR = 2.17; CI: {1.25, 3.77}; P = 0.006).

**Conclusions:** In our study, development of PIH was common (35.6 %) and associated with increased mortality (OR 2.17). Clinicians should attempt to minimize hemodynamic instability during ETI in patients with traumatic injuries. Further investigation of PIH in the trauma population is warranted.

**References**

1. Green RS, Turgeon AF, et al. Postintubation hypotension in intensive care unit patients: A multicenter cohort study. J Crit Care. 2015;30(5):1055–60.

#### A502 Clinical characteristics and injury pattern of geriatric trauma patients

##### H. Tae Hwa, L. Jae Gil

###### Yonsei University College of Medicine, Department of Surgery, Seoul, Republic of Korea

####### **Correspondence:** H. Tae Hwa - Yonsei University College of Medicine, Department of Surgery, Seoul, Republic of Korea

**Introduction:** Recently, the population of elderly people has been increasing rapidly all over the world. Especially, Republic of Korea is now progressing to an aging society. The social activities of the aging population have increased, which has also increased the number of elderly patients injured.

**Objectives:** The aim of this study is to analyze the clinical characteristics and injury pattern of geritaric patients involved in trauma.

**Methods:** This study was conducted retrospectively from January 2015 to March 2016 among trauma patients who visited single trauma center in Seoul, Korea. The patients divided in two groups, a geriatrics group and an adult group on the basis of an age of 65. Patients under 18 years of age were excluded. The variables related with trauma were extracted and compared.

**Results:** Total number of the included patients was 384, and the number of elderly patients was 74. There were no significant differences in Revised Trauma Score (RTS), intensive care unit (ICU) admission, hospital stay between the two groups. There were differences in the mechanism and the locations of the injury, gender, injury severity score (ISS), trauma and injury severity score (TRISS), mortality, drinking at admission between the two groups. Most common injury mechanism in the adult group was driver traffic accident (24.5 %) and in the geriatric group was pedestrian traffic accident (45.9 %). Injury of extremity (44.8 % vs 59.5 %) (p = 0.028) was more common in the geriatric group than in the adult group. And female patient was more common in the geriatric group (26.8 % vs 40.5 %) (p = 0.020). The average ISS of the geriatric group was higher than that of the adult group (17.8+/−11.52 vs. 14.0+/−12.06, p = 0.015). The mortality was higher in the geriatric group (14.9 %) than in the adult group (4.5 %) (p = 0.003). But drinking at admission was higher in the adult group (33.9 % vs 10.8 %) (p < 0.00).

**Conclusions:** The mortality rate and the average ISS were greater within the geriatric group than the adult group despite there was no difference in the RTS, ICU admission. Also elderly people were prone to pedestrian traffic accident with the lower drinking rate compare than the adult group.

### Prediction models and outcome

#### A503 Sequential sofa score as predictor of outcome in septic patients

##### **R. Hernández Vaquero**^1^, E. Rodriguez-Ruiz^1^, A. Lopez Lago^1^, J.L. Garcia Allut^1^, A. Estany Gestal^2^, M.A. Garcia Gonzalez^3^

###### ^1^Hospital Clínico Universitario, Intensive Care Unit, Santiago de Compostela, Spain; ^2^Fundación Ramón Dominguez, Unidad de Epidemiología e Investigación Clínica, Santiago de Compostela, Spain; ^3^Instituto de Investigaciones Sanitarias, Grupo de Genética y Biología del Desarrollo de las Enfermedades Renales, Santiago de Compostela, Spain

####### **Correspondence:** R. Hernández Vaquero - Hospital Clínico Universitario, Intensive Care Unit, Santiago de Compostela, Spain

**Introduction:** Sepsis is a dynamic medical condition where patients´ clinical status can change rapidly in either direction. Different biomarkers and scores have been studied to assess sepsis severity and prognosis**.** Procalcitonin, C-reactive protein and interleukin-6 are biomarkers referred as helpful to follow evolution and outcomes. APACHE II model was developed to predict hospital outcomes, based on data from the first 24 hours. SOFA score describes the degree of organ dysfunction over time and evaluates morbidity, but has been used also for predicting mortality.

**Objective:** Our aim was to study the ability of different biomarkers (procalcitonin, C-reactive protein and interleukin-6) and scores (APACHE II and SOFA), determined on day 1, to predict outcome in septic patients. Besides, we studied sequential SOFA score to find out its dynamics at early sepsis and its correlation with mortality.

**Methods:** We assessed 48 consecutive septic patients admitted to our medico-surgical ICU. Procalcitonin, C-reactive protein and interleukin-6 were determined on day 1. APACHE II was calculated on day 1, and daily SOFA score was calculated for 5 consecutive days, determining also the maximum SOFA. Mann–Whitney U test and ROC curves were carried out to study association of these variables with 28-day mortality.

**Results:** In our cohort of septic patients (mean age 69 ± 13) there were 43 survivors and 5 non-survivors. Procalcitonin, C-reactive protein and interleukin-6 did not show correlation with 28-day mortality. APACHE II, SOFA scores on days 1 and 2, and maximum SOFA, also showed no correlation with 28-day mortality. However, when analyzing SOFA scores on days 3, 4 and 5, we found significant lower scores in survivors compared to non-survivors: SOFA-3 (*p* = 0.048), SOFA-4 (*p* = 0.016), and SOFA-5 (*p* = 0.018). The ROC curve analysis also showed association with 28-day mortality: SOFA-3 (AUC 0.772, *p* = 0.048), and stronger for SOFA-4 (AUC 0.823, *p* = 0.019), and SOFA-5 (AUC 0.820, *p* = 0.021).

**Conclusion:** Day 1 values for procalcitonin, C-reactive protein, interleukin-6, APACHE II and SOFA did not correlate with outcome. But, low SOFA scores on day 3, and specially in days 4 and 5, are good predictors of survival, meaning that after 48 hours of admission, regardless of the initial score, those patients with a low SOFA score have higher probability to survive. While SOFA scores at admission, and maximum SOFA, had bad performance in predicting mortality, sequential SOFA scores add information on the course of the disease, and improve the ability to predict the likelihood of survival. Larger studies are required to find out SOFA based prediction models with improved accuracy.

**References**

(1) Biron *et al.* (2015) Biomarkers for sepsis: what is and what might be? Biomark Insights, 10 (Suppl 4):7–17.

(2) Minne *et al.* (2008) Evaluation of SOFA-based models for predicting mortality in the ICU: A systematic review. Critical Care, 12:R161.

#### A504 Development of a sepsis mortality prediction model for use with German hospital claims data

##### D.O. Thomas-Rüddel^1,2^, D. Schwarzkopf^2^, C. Fleischmann^1,2^, K. Reinhart^1,2^

###### ^1^Universitaetsklinikum Jena, Department of Anesthesiology and Intensive Care, Jena, Germany; ^2^Universitaetsklinikum Jena, Center for Sepsis Control and Care (CSCC), Jena, Germany

####### **Correspondence:** D.O. Thomas-Rüddel - Universitaetsklinikum Jena, Center for Sepsis Control and Care (CSCC), Jena, Germany

**Introduction:** Epidemiological sepsis research in administrative data is widely used in the USA. Risk adjustment models [1] enable adjusted outcome comparisons and benchmarks between regions, hospitals, or over time. For Germany we recently reported first results of an administrative data Approach [2] but a risk model is lacking.

**Objectives:** To develop and asses a risk model of hospital mortality for sepsis based on German hospital claims data.

**Methods:** The hospital claims data of almost all inpatients in Germany is combined into one dataset at the Federal Statistical Office. Anonymized statistical analysis can be performed via remote data access. In data from 2010–2013 all adult cases with pathogen-based or clinical sepsis codes (A & R codes, ICD-10) were classified by severity. Age, sex, type of hospital admission, coded foci of infection and comorbidities based on categories of the Charlson and Elixhauser comorbidity indices, coding of sepsis as main diagnosis and year of treatment were assessed as possible risk factors. Predictors of hospital mortality were identified by backwards binary logistic regression in a derivation cohorts separately for each severity group. Performance of models was tested in validation cohorts. Models were then fitted with the selected predictors for the different severities in all cases and predicted mortality risk was calculated. Combined model performance across all severities was assessed by discrimination using the “area under the curve” (AUC), by calibration plotting observed against predicted mortality over risk quantiles, and by explained variance.

**Fig. 36 (abstract A504).**
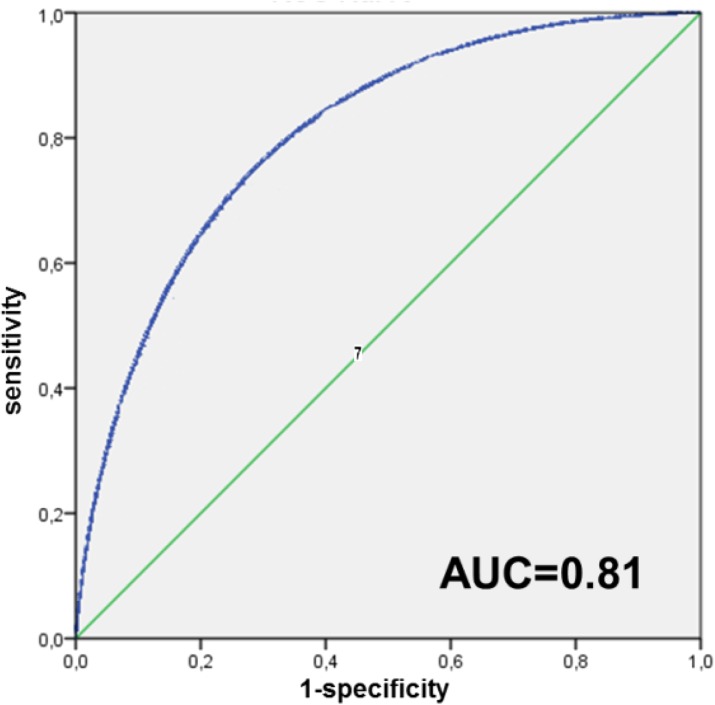
ROC curve of combined model

**Fig. 37 (abstract A504).**
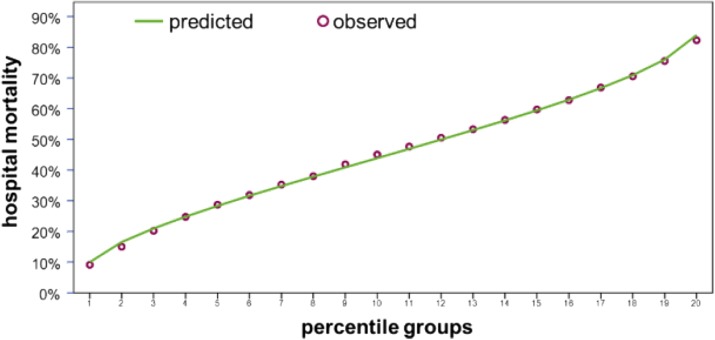
Predicted and observed hospital mortality

**Results:** 441 207 cases with sepsis without organ dysfunction (hospital mortality 13,5 %), 284 883 with severe sepsis (mortality 40,3 %) and 112 609 with septic shock (mortality 59,6 %) were identified. 41–43 of 53 assessed predictors remained in the final models. Overall discrimination (Fig. 36), explained variance (R^2^ = 0.25), and calibration (Fig. 37) were good. Strongest risk factors were age and advanced liver and hemato-oncologic disease. A urogenital focus and sepsis as main diagnosis were associated with lower hospital mortality. Mortality decreased from 2010 to 2013 for sepsis (p < 0.0001) and severe sepsis (p < 0.0001) but not for septic shock (p = 0.11).

**Conclusions:** We developed a well performing risk adjustment model for sepsis mortality in German administrative data. This enables comparisons and benchmarks from administrative data in Germany. Hospital characteristics will be included into the model in the next development step.

**References**

1. Ford DW et al. *Critical care medicine* 2016

2. Fleischmann C et al. *Deutsches Arzteblatt international* 2016

**Grant acknowledgement**

The CSCC is funded by the German Federal Ministry of Education and Research (BMBF), Germany, FKZ: 01EO1502.

#### A505 Modified early warning score to predict mortality in hospital: a systematic review and meta-analysis

##### S. Suwanpasu^1^, Y. Sattayasomboon^2^

###### ^1^KCMH, Nursing Department, Bangkok, Thailand; ^2^Mahidol University, Faculty of Public Health, Bangkok, Thailand

####### **Correspondence:** S. Suwanpasu - KCMH, Nursing Department, Bangkok, Thailand

**Introduction:** Patients at risk of rapid deterioration and critical illness often have preceding changes in their physiological parameters.

**Objectives:** The systematic review quantifies the prognostic accuracy of screening instruments of the Modified Early Warning Score (MEWS) to detect risk of in-hospital mortality.

**Methods:** The review process followed guidelines consisting of 5 steps suggested for systematic reviews. Relevant studies from January 2000 to December 2015 were obtained from electronic databases. Standards for Reporting of Diagnostic accuracy (STARD) and Quality Assessment of Diagnostic Accuracy Studies instrument (QUADAS-2) were used to assess individual study quality and bias. MedCalc statistic software was used to assess and combine the data and diagnostic accuracy across studies. Pooled diagnostic odds ratio and area under The ROC Curve (AUC) analysis was used to evaluate the prognostic accuracy of MEWS tool.

**Results:** A total of 402 citations were identified yielding 16 studies for inclusion in this systematic review. Studies were significantly heterogeneous in terms of age and sample size. For predicting in-hospital death, MEWS with high risk group as cut-off ≥ 4 and ≥ 5 had the Diagnostic odds ratio (DOR) of 14.278 (95 % Confidence Interval [CI] 12.185 to 16.730, *I*^2^ = 56.59 %]) and 3.28 (95%CI:2.489 to 4.323, *I*^2^ = 48.64 %). On pooled AUC analysis, there was a trend for MEWS to estimate fair at the discriminative power of test. AUC of MEWS ≥ 4 was 0.778 (95%CI:0.715 to 0.841, *I*^2^ = 89.54 %) and of MEWS ≥ 5 was 0.646 (95%CI:0.611 to 0.682, *I*^2^ = 49.69 %).

**Conclusions:** On Meta-analysis of sixteen of MEWS studies, this showed a robust positive trend to predict in-hospital death. MEWS equal 4 or greater may be the favored tool to alert the deterioration in hospitalized patients.

**Grant acknowledgement**

Nursing Department, King Chulalongkorn Memorial Hospital, Thai Red Cross Soceity

**Note:** This abstract has been previously published and is available at [1]. It is included here as a complete record of the abstracts from the conference.

**References**

1. Suwanpasu S, Sattayasomboon Y (2016) Accuracy of modified early warning scores for predicting mortality in hospital: a systematic review and meta-analysis. J Intensive & Crit Care 2:2.

#### A506 Validation of SAPS 3 E APACHE II in elderly patients admitted to brazilian intensive care unit

##### N.M. Filgueiras Filho^1,2^, J.C.A. Oliveira^1^, C.S. Ballalai^1,2^, C.V. De Lucia^1,2^, G.P. Araponga^1^, L.N. Veiga^1^, C.S. Silva^1,2^, M.E. Garrido^1,2^, B.B. Ramos^1,2^, E.F. Ricaldi^1,2^, S.S. Gomes^1,2^, GEMINI

###### ^1^Núcleo de Ensino e Pesquisa Hospital da Cidade, Intensive Care, Salvador, Brazil; ^2^Universidade Salvador - UNIFACS, Núcleo de Pesquisa Clínica, Salvador, Brazil

####### **Correspondence:** N.M. Filgueiras Filho - Universidade Salvador - UNIFACS, Núcleo de Pesquisa Clínica, Salvador, Brazil

**Introduction:** The world population is ageing. Together with this fact there is increase rate of elderly patient's admissions to Intensive Care Units. Therefore, studies aimed in elderly population are important. The prognostic scores have been widely used in intensive care medicine, among them SAPS 3 and APACHE II have a significant importance. The sensitivity and specificity of all the systems are vary depending on the score systems and the different groups of patients.

**Objectives:** Validate and compare SAPS 3 and APACHE II in elderly patients admitted in ICU in Salvador-BA, Brazil.

**Methods:** Prospective cohort study placed in a general ICU. In the study were included patients above age of 65 admitted to general ICU, from March 2010 to May 2013. All the patients were assessed using the SAPS 3 (Global Equation and Central & South American Equation) and APACHE II scoring systems. Logistic regressions analysis was used to calculate the sensitivity, specificity and accuracy as well as the OR probability of mortality. The ability to predict mortality was performed with ROC curves. The differences between observed-to-predicted mortality were analyzed with the Hosmer-Lemeshow test.

**Results:** There were analyzed 1106 patients, 497 (44.9 %) males. Median age: 76 years (IQR: 71–83 years). Hospital mortality 207 (18.7 %). Median SAPS 3 and APACHE II: 48 (IQR: 42–55), 14 (IQR: 11–18). The sensitivity, specificity and accuracy were respectively 70 %, 68.7 % and 71.4 % for SAPS 3 Global Equation; 70 %, 68.7 % and 71.4 % for SAPS 3 Central & South American Equation (CSA); 67.6 %, 69.1 % and 68.81 % for APACHE II. The area under the curve (AUC) of these models was: 0.748 for SAPS 3 Global Equation, 0.745 for SAPS 3 Central & South American Equation (CSA) and 0.740 for APACHE II. But the Hosmer-Lemeshow goodness-of-fit test H statistic also revealed poor performance for SAPS 3 Central & South American Equation (CSA) scoring system.

**Conclusions:** In our series, the SAPS 3 Global Equation is the model that best predicts mortality in patients with at least 65 years admitted to a Brazilian ICU. Also, SAPS 3 Central & South American Equation (CSA) revealed poor calibration for critical care elderly patients.

**References**

1- Sacanella E, Pérez-Castejón JM, Nicolás JM et al. Intensive Care Med. 2009 Mar;35(3):550–5. 2- Soares M, Silva UV, Teles JM et al. Intensive Care Med. 2010 Jul;36(7):1188–95.

#### A507 Evaluation of QSOFA as a predictive tool using a restrospective ICU cohort

##### L. Gemmell, A. MacKay, C. Wright, R.I. Docking, P. Doherty, E. Black, P. Stenhouse

###### Queen Elizabeth University Hospital, Anaesthetics and Intensive Care, Glasgow, UK

####### **Correspondence:** L. Gemmell - Queen Elizabeth University Hospital, Anaesthetics and Intensive Care, Glasgow, UK

**Introduction:** Recent guidelines on the definition of sepsis from the Sepsis 3 working group have suggested the use of a simplified version of the SOFA score as an initial way to screen for sepsis (1). This score allocates a point each for new altered mentation (GCS < 15), respiratory rate greater than 22 and a systolic blood pressure < 100 mmHg. It has been given the term qSOFA based on the original SOFA (sepsis related organ failure assessment). The purpose of the score is to identify patients early with sepsis as dealy in recognition is associated with an increased mortality and increased length of stay. We decided to evaluate whether the qSOFA score when applied to ICU patients could be validated for use in our patient population.

**Methods:** A historical patient cohort was identified by searching the WardWatcher CIS dataset for all patients admitted from 01/01/204 - 31/12/2014 to a five bedded teaching hospital Intensive Care Unit in Glasgow. These patients demographic data was collected along with the parameters from qSOFA (as above) along with outcome data. After removal of patients with incomplete datasets, statistical analysis was performed.

**Results:** There were 3357 patients included in the study, with analysis of 1353 due to incomplete qSOFA data. Baeline data for these patients was as follows: 58.9 % male, mean age 57.4 +/− 0.9 years, mean APACHE-II score 19.7 +/− 0.5, mean predicted mortality 37 +/− 1.5 %, mean length of stay 5.0 +/− 0. days and mortality of 33 %. Using the available data from Wardwatcher we generated qSOFA data as below.

**Conclusions:** As can be seen from above there is a good correlation between the qSOFA and APACHE-II in terms of recognition of severity. There is a significant correlation between qSOFA and increased length of stay and mortality. This would suggest that qSOFA is a quick and useful tool that can be used on ICU admission to aid prognostication which can assist in discussions with patients, relatives and colleagues.

**References**

1. Seymour et al. Assessment of clinical criteria for sepsis. JAMA 2016;315(8):762–774

**Table 19 (abstract A507).** qSOFA

qSOFA	0	1	2	3	p-value
n	97	384	574	296	
APACHE II	13.5 +/− 1.2	16.2 +/− 0.8	20.4 +/− 0.7	24.8 +/− 1.3	<0.001
ICU stay	3.6 +/− 0.8	4.4 +/− 0.5	5.1 +/− 0.5	5.7 +/− 0.8	0.006
Mortality	12.4%	21.9%	35.5%	50.3%	<0.001

## Poster Corner Sessions

### ACID-BASE BALANCE AND INTERMEDIARY METABOLISM

#### A508 The risk of type 2 diabetes in survivors of critical illness with stress induced hyperglycaemia

##### M.P. Plummer^1,2^, M.E. Finnis^1,2^, L.K. Phillips^3,4^, P. Kar^1,2^, S. Bihari^5,6^, V. Biradar^7^, S. Moodie^2^, M. Horowitz^3,4^, J.E. Shaw^8^, A.M. Deane^1,2^

###### ^1^University of Adelaide, Discipline of Acute Care Medicine, Adelaide, Australia; ^2^Royal Adelaide Hospital, Intensive Care Unit, Adelaide, Australia; ^3^University of Adelaide, Discipline of Medicine, Adelaide, Australia; ^4^Royal Adelaide Hospital, Department of Endocrinology, Adelaide, Australia; ^5^Flinders University, Department of Critical Care Medicine, Adelaide, Australia; ^6^Flinders Medical Centre, Department of Intensive Care Medicine, Adelaide, Australia; ^7^Lyell McEwin Hospital, Department of Intensive Care Medicine, Adelaide, Australia; ^8^Baker IDI Heart and Diabetes Institute, Melbourne, Australia

####### **Correspondence:** M.P. Plummer - Royal Adelaide Hospital, Intensive Care Unit, Adelaide, Australia

**Introduction:** Hyperglycaemia occurs frequently in the critically ill and may be secondary to either diabetes (recognised or not), or stress induced hyperglycaemia. Stress induced hyperglycaemia occurs in patients who have normal glucose tolerance following resolution of their acute illness. Whether stress induced hyperglycaemia unmasks latent insulin resistance and/or impaired β-cell function has not been adequately explored.

**Objectives:** To evaluate the association between stress induced hyperglycaemia, the development of diabetes and long-term mortality in survivors of critical illness.

**Methods:** This is a retrospective, multi-centre observational study. All adult patients surviving admission to a tertiary intensive care unit (ICU) in South Australia between 2004 and 2011 were included. Stress induced hyperglycaemia was defined as a blood glucose ≥ 11.1 mmol/l within the first 24 hours of ICU admission. Prevalent diabetes was identified through ICD-10 coding or prior registration with the Australian National Diabetes Service Scheme (NDSS). Incident diabetes was identified as NDSS registration > 30 days after hospital discharge until July 2015. The predicted risk of developing diabetes was described as sub-hazard ratios using competing risk regression. Survival was assessed using Cox proportional hazards regression.

**Results:** Stress induced hyperglycaemia was identified in 2,883 (17 %) of 17,074 patients without diabetes. The overall incidence of subsequent type 2 diabetes following critical illness was 4.8 % (821 of 17,074). The risk of diabetes in patients with stress induced hyperglycaemia was approximately double that of those without (HR 1.91 (95 % CI 1.62, 2.26), *p* < 0.001) and was sustained regardless of age or severity of illness. Subdividing age into approximate deciles the greatest risk was seen in the 50–59 year age group with over a seven-fold risk (HR 7.90 (5.38, 11.60), *p* < 0.001). Stress induced hyperglycaemia was not associated with increased long-term mortality in patients who survived their hospital admission.

**Conclusions:** Stress induced hyperglycaemia within 24 hours of admission to the ICU identifies patients at greater risk of subsequent diabetes.

**Grant acknowledgement**

This project was supported by grants from the Diabetes Australia Research Trust and a University of Adelaide, Discipline of Acute Care Medicine Maurice Sando Project Grant.

#### A509 Identifying an optimal target for blood glucose management among critically ill patients: a network meta-analysis

##### T. Yatabe^1^, S. Inoue^2^, M. Sakaguchi^3^, M. Egi^4^

###### ^1^Kochi Medical School, Department of Anesthesiology and Intensive Care Medicine, Nankoku, Japan; ^2^Tokai University Hachioji Hospital, Department of Emergency and Critical Care Medicine, Hachioji, Japan; ^3^Kochi Medical School, Integrated Center for Advanced Medical Technologies, Nankoku, Japan; ^4^Kobe University Hospital, Department of Anesthesiology, Kobe, Japan

####### **Correspondence:** T. Yatabe - Kochi Medical School, Department of Anesthesiology and Intensive Care Medicine, Nankoku, Japan

**Introduction:** Although several clinical guidelines recommend maintaining blood glucose between 144 and 180 mg/dL, these are insufficient evidences to assess the benefit and harm of this glycemic target.

**Objectives:** We conducted a network meta-analysis to assess the impact of glycemic target 144-180 mg/dL on the incidence of hypoglycemia, the risk of infection and mortality in compared with other glycemic target as < 110 mg/dL, 110-144 mg/dL and >180 mg/dL.

**Methods:** We considered all studies from three recent systematic reviews [1–3] and searched the PubMed database for studies that compared target blood glucose levels among critically ill patients.

The primary outcomes were in-hospital mortality, and the secondary outcomes were the incidence of hypoglycemia, and risk of sepsis or bloodstream infection.

**Results:** We identified 71 studies through the re-analysis of the three systematic reviews, and 431 studies through the PubMed search. Thirty-nine potentially eligible studies were considered for inclusion, although 12 studies were excluded after screening the full texts. Thus, the network meta-analysis included 14,495 patients from 27 studies. The mean patient age was 61.6 years, and 21.7 % of the patients had diabetes. There was no significant difference of in-hospital mortality and risk of sepsis or bloodstream infection among 3 glycemic bands (<110 mg/dL, 110-144 mg/dL and 144-180 mg/dL). Target blood glucose levels of 144–180 mg/dL had significantly lower in-hospital mortality and risk of sepsis or bloodstream infection in compared with >180 mg/dL (odds ratio [OR]:0.82, 95 % credible intervals [CI]: 0.69-0.96; OR: 0.69, 95 % CI: 0.52-0.92, respectively). Our network meta-analysis revealed that target blood glucose levels of < 110 mg/dL and 110–144 mg/dL were associated with a 6–7 fold higher risk of hypoglycemia, compared to targets of 144–180 mg/dL and >180 mg/dL. There were no significant differences in the risk of hypoglycemia between < 110 mg/dL and 110–144 mg/dL (OR = 0.76 (95 % CI: 0.49-1.11). There were also no significant differences in the risk of hypoglycemia between and between 144–180 mg/dL and >180 mg/dL (OR = 1.0 (95 % CI: 0.30-2.70). The rank probability analysis indicated that a target blood glucose level of 144–180 mg/dL had the highest probability of being the best target blood glucose level for reducing hypoglycemia and hospital mortality rates and was the second best target level, following a target level of 110–144 mg/dL, for reducing sepsis or bloodstream infection rates.

**Conclusions:** The results of our network meta-analysis indicate that target blood glucose levels of 144–180 mg/dL is an optimal target for balancing the risks and benefits of insulin therapy among critically ill patients.

**References**

[1] Wiener RS,et al. JAMA. 2008;300:933–44.

[2] Friedrich JO, et al. Crit Care. 2010;14:324.

[3] Song F, et al. Biomed Res Int. 2014;2014:698265.

**Grant acknowledgement**

None.

#### A510 Long-term mortality of critically ill patients with diabetes who survive admission to intensive care

##### Y. Ali Abdelhamid^1,2^, M.P. Plummer^1,2^, M.E. Finnis^1,2^, L.K. Phillips^3,4^, P. Kar^1,2^, S. Bihari^5,6^, V. Biradar^7^, S. Moodie^1^, M. Horowitz^3,4^, J.E. Shaw^8^, A.M. Deane^1,2^

###### ^1^Royal Adelaide Hospital, Department of Critical Care Services, Adelaide, Australia; ^2^University of Adelaide, Discipline of Acute Care Medicine, Adelaide, Australia; ^3^University of Adelaide, Discipline of Medicine, Adelaide, Australia; ^4^Royal Adelaide Hospital, Endocrine and Metabolic Unit, Adelaide, Australia; ^5^Flinders University, Department of Critical Care Medicine, Adelaide, Australia; ^6^Flinders Medical Centre, Department of Intensive Care Medicine, Adelaide, Australia; ^7^Lyell McEwin Hospital, Department of Intensive Care Medicine, Adelaide, Australia; ^8^Baker IDI Heart and Diabetes Institute, Melbourne, Australia

####### **Correspondence:** Y. Ali Abdelhamid - University of Adelaide, Discipline of Acute Care Medicine, Adelaide, Australia

**Introduction:** Diabetes is a risk factor for the development and severity of critical illness, and the need for Intensive Care Unit (ICU) admission. Despite this, diabetes does not confer a greater risk of death for those with critical illness within the index hospital admission. However, long-term outcomes in patients with diabetes who survive ICU admission remain unknown.

**Objectives:** Our objectives were to evaluate the effect of diabetes on both long-term survival rates and average years of life lost for patients admitted to ICU and who survived hospitalisation.

**Methods:** We evaluated all adult patients in South Australia between 2004 and 2011 who survived hospitalisation requiring admission to a Public Hospital ICU. Demographic and admission data from the Australia and New Zealand Intensive Care Society Adult Patient Database were linked to population based datasets to match (i) International Classification of Disease (ICD-10) codes for diabetes through the Department of Health Integrated South Australian Activity Collection dataset, (ii) diabetes diagnosis in the national register (the National Diabetes Service Scheme), and (iii) mortality through the Australian National Death Index. Life years lost were calculated using age and sex specific life-tables from the Australian Bureau of Statistics.

Data are presented as frequencies and proportions for categorical variables and mean (standard deviation) or median [interquartile range] for continuous variables. Between group comparisons were performed by *t*-test, Wilcoxon rank-sum or Chi-square tests. Longitudinal survival was analysed using Cox proportional hazards regression and average life years lost by linear regression.

**Fig. 38 (abstract A510).**
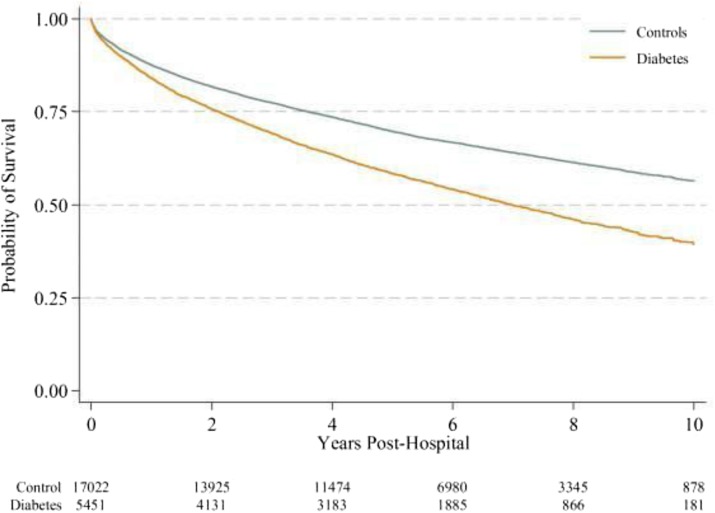
ᅟ

**Results:** 5451 patients with and 17022 patients without diabetes were included. Patients with diabetes were older (64.7 (14.9) vs 57.6 (19.7) years; p < 0.0001) and had higher illness severity on admission to ICU (APACHE III 62 [48, 80] vs 54 [38, 72]; p < 0.0001). Crude mortality rates [95%CI] were 105.5 [101.6-109.6] and 67.6 [65.9-69.3] per 1000 person years for those with and without diabetes respectively. Patients with diabetes were more likely to die after hospital discharge (unadjusted hazard ratio 1.52 [95 % CI 1.46-1.59]; p < 0.001) (Fig. 38).

**Fig. 39 (abstract A510).**
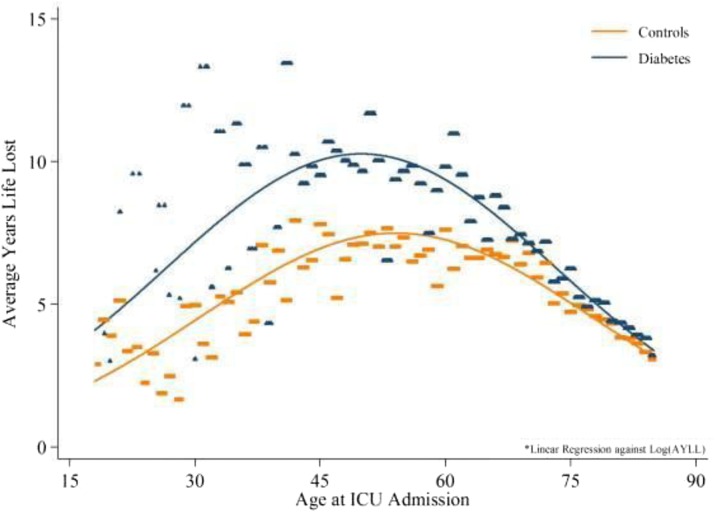
ᅟ

Diabetes remained an independent risk factor for death when adjusted for age, sex, site, indigenous status, APACHE III score and admission diagnosis (hazard ratio 1.17 [95 % CI 1.12-1.22]; p < 0.01). Patients with diabetes also suffered a greater number of average life years lost and this was most marked in the 4^th^ and 5^th^ decades of life (Fig. 39).

**Conclusions:** Mortality and number of life years lost for ICU survivors with diabetes is greater than for survivors without diabetes. Strategies to improve outcomes for these patients should be evaluated.

**GRANT ACKNOWLEDGEMENT**

Dr Ali Abdelhamid is supported by a Royal Adelaide Hospital Clarkson Scholarship. The study was supported by a Diabetes Australia Research Trust Project Grant.

#### A511 Glycated hemoglobin A1C on the day of emergency surgery is the marker of premorbid glycemic control

##### M. Hokka, M. Egi, S. Mizobuchi

###### Kobe University Hospital, Anesthesiology, Kobe, Japan

####### **Correspondence:** M. Hokka - Kobe University Hospital, Anesthesiology, Kobe, Japan

**Introduction:** Glycated hemoglobin A1c (HbA1c) is used to estimate chronic glycemic control. Recently, several studies suggested that the optimal glycemic control in acutely ill patients might be better being adjusted according to the premorbid chronic glycemic control (1–3). However, the majority of patients would not have been measured the HbA1c-level prior to the acute illness. As the HbA1c-level would be affected by the patients' conditions, HbA1c-levels during an acute illness may not reflect the actual premorbid glycemic status. Only limited research has been conducted so far to assess the value of HbA1c-levels during the state of an acute illness to estimate the premorbid glycemic control (4).

**Objectives:** Assessment of the relationship between HbA1c during the state of an acute illness and HbA1c levels measured within 30 days before an acute illness.

**Methods:** These are retrospective observational studies. The Ethics Committee of the Kobe University Hospital approved this investigation. The committee waived the need for informed consent concerning studies involving the use of databases. We screened patients' HbA1c levels that were measured within 30 days prior to an emergency surgery, requiring general anesthesia. Among them, we selected patients whose HbA1c levels were also measured on the day of the surgery. Thus we obtained HbA1c-levels measured within 30 days before the emergency surgery (preHbA1c) and on the day of the surgery (opeHbA1c). Those HbA1c-levels were measured in the same laboratory. The correlations of the two HbA1c levels were assessed, using Pearson's correlation coefficient. Agreement between the two HbA1c-levels was assessed using the Bland-Altman approach.

**Results:** We studied 100 HbA1c-levels in 50 patients. The patients were 66.8 years old in average. 31 of them were male patients (62 %). 38 out of 50 patients (76 %) were required emergency cardiovascular surgery. PreHbA1c was measured 11.4 days before the emergency operation. The average preHbA1c was 6.3 % and the opeHbA1c was 6.2 %. There is a significant correlation between preHbA1c and opeHbA1c (r = 0.83, p < 0.001). The mean difference between preHbA1c and opeHbA1c was −0.1 % (95 % confidential interval; −1.2 % to 0.9 %). This difference of the two HbA1c did not correlate with the time between the two measurements (r = 0.17, p = 0.29).

**Conclusions:** The HbA1c-level on the day of surgery is a useful marker of premorbid glycemic control in patients requiring emergency surgery.

**References**

1) Crit Care Med 2011; 39:105–111

2) Intensive Care Med 2014; 40:973–980

3) Intensive Care Med. 2016;42(4):562–71

4) Crit Care Med.2016 [Epub ahead of print

#### A512 Effects of antecedent hypoglycaemia on cardiac and gastric responses to subsequent hypoglycaemia in health

##### P. Kar^1,2^, M. Plummer^3^, Y. Ali Abdelhamid^1,2^, E. Giersch^1^, M. Summers^1^, S. Hatzinikolas^4^, S. Heller^5^, M. Chapman^1,2,4^, K. Jones^4,6^, M. Horowitz^4,6^, A. Deane^1,2,4^

###### ^1^Royal Adelaide Hospital, Intensive Care Unit, Adelaide, Australia; ^2^University of Adelaide, Discipline of Acute Care Medicine, Adelaide, Australia; ^3^Addenbrooke's Hospital, Neurosciences Critical Care Unit, Cambridge, UK; ^4^University of Adelaide, NHMRC Centre for Research Excellence, Adelaide, Australia; ^5^University of Sheffield, Academic Unit of Diabetes, Endocrinology and Metabolism, Sheffield, UK; ^6^University of Adelaide, Discipline of Medicine, Adelaide, Australia

####### **Correspondence:** P. Kar - University of Adelaide, Discipline of Acute Care Medicine, Adelaide, Australia

**Introduction:** Hypoglycaemia during ICU admission is associated with adverse outcomes after discharge. In both health and patients with diabetes, acute hypoglycaemia triggers counter-regulatory responses increasing cardiac contractility and accelerating gastric emptying. However, antecedent hypoglycaemia attenuates endogenous catecholamine and vagal responses to subsequent hypoglycaemia^1^. Accordingly, patients who were hypoglycaemic during ICU may be unable to mount a physiological response to hypoglycaemia once discharged from ICU.

**Objectives:** To determine the effects of antecedent hypoglycaemia on cardiac and gastric responses to subsequent hypoglycaemia in health.

**Methods:** Ten healthy men (age 22.5 (1.0) years, BMI 23.8 (0.5) kg/m^2^) were studied on two occasions, lasting 30 hours, separated by ≥ 6 weeks and randomised to either 'control' (Day 1 (_C1_) - three euglycaemic clamps at a blood glucose of 6 mmol/L followed day 2 (_C2_) - one hypoglycaemic clamp at 2.8 mmol/L), or 'antecedent hypoglycaemia' (Day 1 (_AH1_) - three clamps at blood glucose of 2.8 mmol/L followed by day 2 (_AH2_) - one clamp at 2.8 mmol/L). Cardiac contractility was measured using fractional shortening (FS) via 2D echocardiography on days 1 and 2 of the control (FS_C1_ and FS_C2_) and antecedent hypoglycaemia (FS_AH1_ and FS_AH2_) periods. Gastric emptying (GE) was measured on day 1 and day 2, for both control (GE_C1_ and GE_C2_) and antecedent hypoglycaemia (GE_AH1_ and GE_AH2_) study periods, using scintigraphy, following ingestion of a standardised meal consisting of 100 g of minced beef labelled with 20 MBq ^99m^technetium-sulphur colloid. Radioisotopic data were acquired for 180 minutes. Data are mean (SE).

**Fig. 40 (abstract A512).**
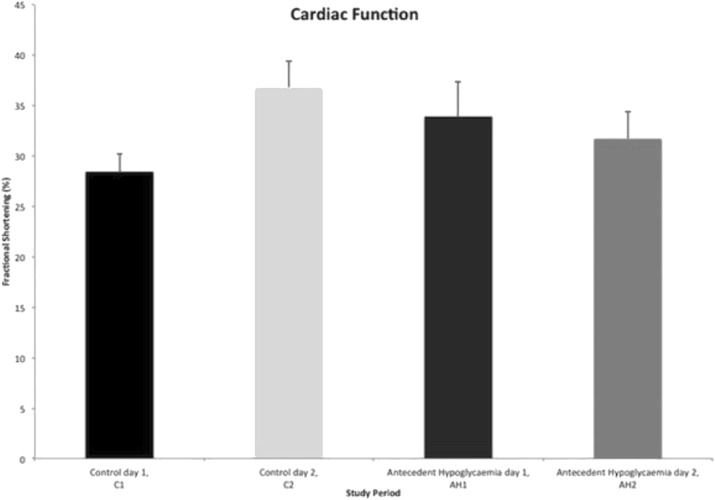
Cardiac fractional shortening on day 1 and day 2 of control and antecedent hypoglycaemia study periods

**Fig. 41 (abstract A512).**
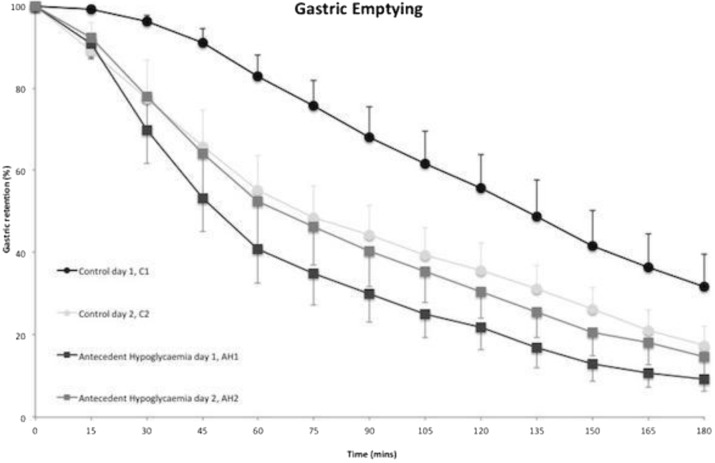
Gastric emptying curves on day 1 and day 2 of control and antecedent hypoglycaemia study periods

**Results:** Fractional shortening was greater (FS_C2_ vs. FS_C1_, P < 0.01) and gastric emptying faster (GE_C2 AUC_ vs. GE_C1 AUC_, P = 0.01) during acute, single episode hypoglycaemia compared to euglycaemia. When testing the effect of antecedent hypoglycaemia compared to a single episode of hypoglycaemia, fractional shortening may be attenuated (FS_AH2_ vs. FS_C2_, P = 0.06), whereas gastric emptying was unaffected (GE_AH2__AUC_ vs. GE_C2 AUC_, P = 0.74) (Figs. 40 and 41).

**Conclusions:** In health, the increase in cardiac contractility during acute hypoglycaemia may be affected by antecedent hypoglycaemia whereas acceleration of gastric emptying during acute hypoglycaemia does not appear to be affected by antecedent was unaffected. These data suggest that antecedent hypoglycaemia during ICU may have “downstream” effects after discharge.

**References**

1. Heller SR, Cryer PE. Reduced neuroendocrine and symptomatic responses to subsequent hypoglycemia after 1 episode of hypoglycemia in nondiabetic humans. Diabetes. Feb 1991;40(2):223–226

**Grant acknowledgement**

Dr Kar is supported by a Royal Adelaide Hospital A.R. Clarkson Scholarship.

#### A513 Limits of the Stewart approach for plasmatic acid–base disturbance interpretation: an illustration with a mechanically ventilated porcin model of metabolic acidosis

##### R. Schweizer^1^, M. Jacquet-Lagreze^1^, P. Portran^1^, S. Junot^2^, B. Allaouchiche^3^, J.-L. Fellahi^1^

###### ^1^Anesthesiology and Critical Care Medicine, Hôpital Cardiologique, Hospices Civils de Lyon, Bron, France; ^2^VetAgro Sup, Ecole vétérinaire de Lyon, Marcy l'Etoile, France; ^3^Anesthesiology and Critical Care Medicine, Centre Hospitalier Lyon Sud, Hospices Civils de Lyon, Pierre Benite, France

####### **Correspondence:** R. Schweizer - Anesthesiology and Critical Care Medicine, Hôpital Cardiologique, Hospices Civils de Lyon, Bron, France

**Introduction:** Plasmatic acid–base equilibrium remains a confusing area more than a century after the Henderson-Hasselbalch equation publication. However, a consensus exists concerning arterial carbon dioxide pressure (pCO_2_) that would represent respiratory part of this equilibrium ^1^. Stewart described this parameter as "independent", in contrast to "dependent" parameters, e.g. pH and HCO_3_^−^^2^.

**Objectives:** The primary objective of this experimental trial is to demonstrate that lactic acid infusion increase pCO_2_. This would be inconsistent with Stewart approach.

**Methods:** After local ethical committee approval, 9 anesthetized, curarized and mechanically ventilated piglets were studied. Lactic acid infusion (0.33 mmol.kg^−1^.min^−1^) was administered. Each 3 minutes, an arterial gasometry was done. Each minute, the following parameters were collected: mean arterial pressure (MAP), cardiac output measured by transpulmonary thermodilution (CO), pulse oxymetry (SpO_2_) and end-tidal CO_2_ (EtCO_2_).

Bicarbonate (HCO_3_^−^) was calculated like this: HCO_3_^−^ = 0.03*pCO_2_*10^pH-6.1^. Total CO_2_(tCO_2_) was calculated like this: tCO_2_ = HCO_3_^−^ + 0.03*pCO_2_

The primary endpoints were pCO_2_ increase and tCO_2_ stability.

To test the hypothesis of normal distribution, Shapiro Wilk test was applied. The p values were obtained from ANOVA analyses. p < 0.05 was considered as significant.

**Fig. 42 (abstract A513).**
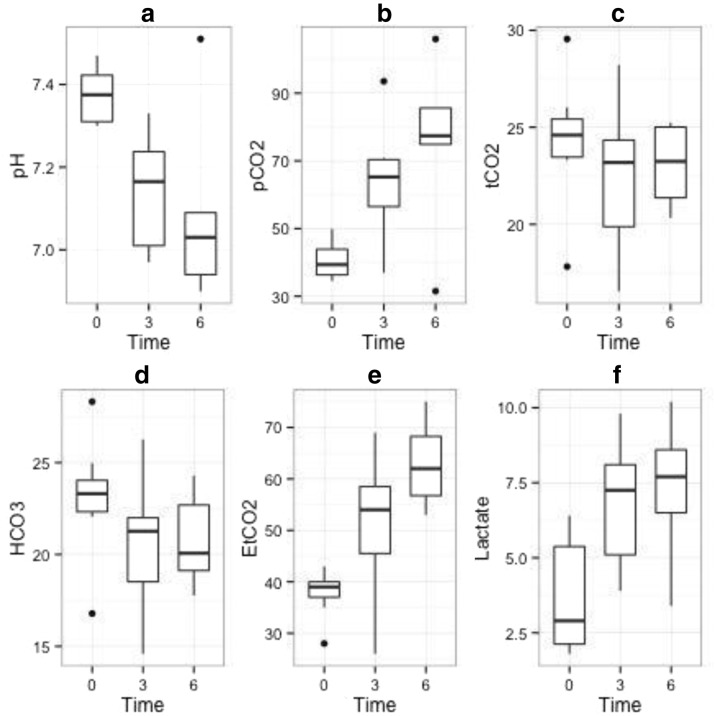
ᅟ

**Results:** The results were: a decrease in pH (p < 0.001) (Fig. 42a), an increase in pCO_2_ (p = 0.002) (Fig. 42b), a decrease in HCO_3_^−^ (p = 0.015) (Fig. 42d) without significant alteration in tCO_2_ (p = 0.08) (Fig. 42c). We also observed: an increase in EtCO_2_ (p = 0.001) (Fig. 42e) and lactatemia (p < 0.001) (Fig. 42f). CO (p = 0.052), MAP (p = 0.506) and SpO2 (p = 0.905) were not significantly altered.

**Conclusions:** This in vivo metabolic acidosis model with stable minute ventilation shows an increase in pCO_2_ without significant alteration in tCO_2_. Although these results were biochemically predictable, they question one of the basements of Stewart approach, that considered pCO_2_ as an independent parameter. In our study, pCO_2_ seems a dependent parameter. tCO_2_, too often neglected parameter, seems an independent parameter.

**References**

(1) HJ Adrogué et al., Assessing acid–base disorders, Kidney Int, 2009; 76(12):1239–47

(2) PA Stewart, Modern quantitative acid–base chemistry, Can J Physiol Pharmacol, 1983; 61(12):1444–61

#### A514 The role of bicarbonate precursors in balanced fluids during haemorrhagic shock with and without compromised liver function

##### P. Guerci^1,2,3^, B. Ergin^1^, A. Kapucu^4^, C. Ince^1^

###### ^1^Academic Medical Center, University of Amsterdam, Department of Translational Physiology, Amsterdam, Netherlands; ^2^University Hospital of Nancy, Departement of Anaesthesiology and Critical Care Medicine, Vandoeuvre-Les-Nancy, France; ^3^University of Lorraine, INSERM U1116, Vandoeuvre-Les-Nancy, France; ^4^Science Faculty, University of Istanbul, Department of Biology, Istanbul, Turkey

####### **Correspondence:** P. Guerci - University of Lorraine, INSERM U1116, Vandoeuvre-Les-Nancy, France

**Introduction:** Lactate, acetate and gluconate are anions used in balanced resuscitation fluids of which lactate and acetate are considered bicarbonate precursors. Several studies have demonstrated that balanced fluids are commonly used for volume expansion of critically ill patients who may also have impaired liver function (1,2). Little is known of the metabolic fate of this anions when the primary metabolising organ is compromised. Moreover, the role of gluconate embedded in PlasmaLyte in terms of acid–base control is unknown.

**Objectives:** This study investigated the role of the liver in the efficacy of balanced and unbalanced solutions to correct acid–base alterations and renal haemodynamics and microvascular oxygenation in a rat model of resuscitated hemorrhagic shock (HS).

**Methods:** Ringer's Lactate (RL), Ringer's Acetate (RA), Plasma-Lyte (PL) or normal saline (NS) were administered following HS in the presence or absence of a 70 % partial liver resection (PLR). Renal haemodynamics and microvascular oxygenation (by oxygen-dependent quenching of phosphorescence) were measured as well as levels of lactate, gluconate and acetate in plasma and urine. Kidney wet and dry weighing was also assessed. **Results:** PLR resulted in increased liver enzymes compared in control and HS groups (p < 0.01). HS decreased systemic and renal haemodynamics and reduced microvascular kidney oxygenation to 20 and 14 mmHg for cortex and medulla respectively, associated with lactic acidosis (p < 0.01). Resuscitation with balanced fluids did not fully restore renal oxygenation (p < 0.01). RA and PL increased bicarbonate levels (15.5 ± 1.1 and 16.4 ± 1.6 vs 13.4 ± 2.0 and 11.5 ± 2.5 mmol/L) and restored pH better than RL or NS (7.29 ± 0.09 and 7.30 ± 0.04 vs 7.22 ± 0.12 and 7.14 ± 0.09) respectively in the PLR experiment (p < 0.01). PLR caused an increase in plasma gluconate after PL resuscitation (10.4 ± 2.8 vs 13.8 ± 5.1 g/L p < 0.05).

**Conclusions:** Acetate buffered balanced fluids show superior buffering effects than RL and NS. Gluconate is partially metabolized by the liver although it does not contribute to acid–base control because of large excretion in urine. Acetate is metabolized regardless of liver function and may be the most efficient bicarbonate precursor. Lactate infusion tends to overwhelm the metabolism capacities of the residual liver. NS seemed to be the most unsuitable fluid when compared to balanced fluids, even in cases of liver failure.

**References**

1. Cecconi M et al. Fluid challenges in intensive care: the FENICE study: A global inception cohort study. *Intensive Care Med* 2015; **41**: 1529–37

2. Boulain T et al. Volume expansion in the first 4 days of shock: a prospective multicentre study in 19 French intensive care units. *Intensive Care Med* 2015; **41**: 248–56

**Grant acknowledgement**

P. Guerci is supported by a grant from the Société Française d'Anesthésie-Réanimation (SFAR), France. This study has been funded by Baxter HealthCare.

**Fig. 43 (abstract A514).**
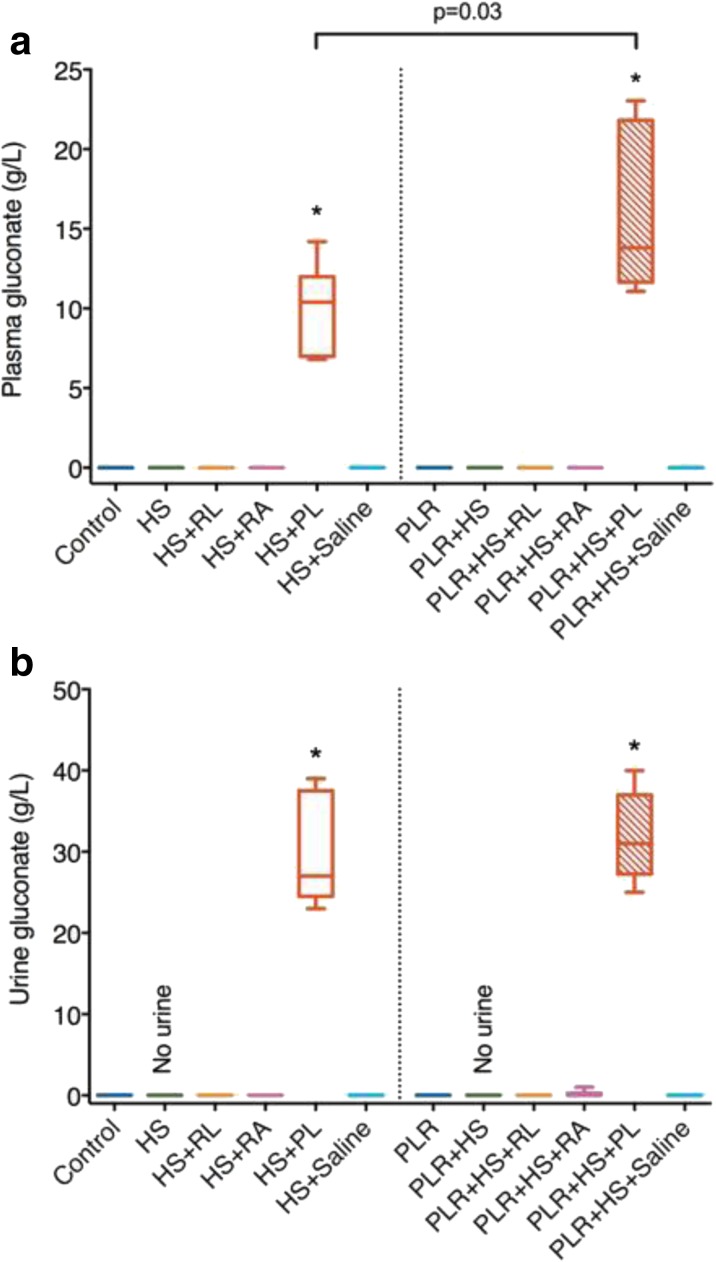
Plasma and urine gluconate levels after fluid

#### A515 Prevalence of ketosis, ketonuria and ketoacidosis during permissive hyperglycemia in critically ill patients with diabetes

##### L. Cioccari^1,2^, N. Luethi^1^, M. Crisman^1,3^, R. Bellomo^1,4^, J. Mårtensson^1,5^

###### ^1^Austin Health, University of Melbourne, Department of Intensive Care, Heidelberg, Australia; ^2^Lucerne Cantonal Hospital, Department of Intensive Care, Lucerne, Switzerland; ^3^Cattinara Hospital, Trieste University School of Medicine, Department of Perioperative Medicine, Intensive Care and Emergency, Trieste, Italy; ^4^Australian and New Zealand Intensive Care Research Centre (ANZIC-RC), Monash University, Department of Epidemiology and Preventive Medicine, Melbourne, Australia; ^5^Karolinska Institutet, Section of Anaesthesia and Intensive Care Medicine, Department of Physiology and Pharmacology, Stockholm, Sweden

####### **Correspondence:** L. Cioccari - Lucerne Cantonal Hospital, Department of Intensive Care, Lucerne, Switzerland

**Introduction:** Recent evidence suggests that a more liberal glycemic control (target blood glucose level between 10 and 14 mmol/l) may be beneficial in patients with diabetes adapted to chronic hyperglycemia (glycated hemoglobin A1c [HbA1c] >7 %)^1^. However, whether such liberal strategies leads to relative insulin deficiency, accelerated ketone body production and ketoacidosis is uncertain. Accordingly, we conducted a prospective observational study to explore the incidence of ketosis, ketonuria and ketoacidosis during liberal glucose management of critically ill diabetic patients.

**Methods:** We studied 60 critically ill diabetic patients treated according to a liberal glucose protocol targeting a blood glucose level (BGL) between 10-14 mmol/l. We performed daily measurement of bedside blood 3-beta-hydroxybutyrate (β-OHB) and semi-quantitative urine ketones on ICU admission and on the following mornings during the ICU stay, for a maximum of 10 consecutive days.

**Results:** Median (IQR) blood ketone level on admission was 0.3 (0.1, 0.8) mmol/l. Ketoacidosis was rare (3 %), but some level of ketosis (β-OHB ≥ 0.6 mmol/l) was found in 38 patients (63 %) at some stage during their ICU stay. However, there was no significant difference in severity or prevalence of ketonemia and ketonuria among patients with BGL above (permissive hyperglycemia) or below 10 mmol/l. On multivariate linear regression there was no association between blood ketone levels and BGL, HbA1c, lactate levels or APACHE III score.

**Conclusions:** Liberal glycemic control in critically ill, predominantly type 2, diabetic patients itself does not appear to lead to increased ketogenesis. We found no difference in prevalence and severity of ketonemia between patients with or without permissive hyperglycemia. Furthermore, severity of ketosis was unrelated to blood glucose levels.

**References**

1. Mårtensson J, Bellomo R. The Rationale for Permissive Hyperglycemia in Critically Ill Patients with Diabetes. In: Vincent J-L, editor. Annual Update in Intensive Care and Emergency Medicine 2016. Cham: Springer International Publishing; 2016. p. 365–372.

#### A516 Evaluation of different glucose variability indices with continuous and intermittent glucose monitoring

##### C. Righy Shinotsuka, D. Fagnoul, A. Brasseur, D. Orbegozo, J.-L. Vincent, J.-C. Preiser

###### Université Libre de Bruxelles (ULB) - Hôpital Erasme, Intensive Care, Brussels, Belgium

####### **Correspondence:** C. Righy Shinotsuka - Université Libre de Bruxelles (ULB) - Hôpital Erasme, Intensive Care, Brussels, Belgium

**Introduction:** Reducing glucose variability (GV) is one of the aims of blood glucose (BG) control as it is an independent predictor of ICU and hospital mortality. Continuous glucose monitoring (CGM) might help to decrease GV by providing frequent measurements of glucose and information about glucose trend. However, the metrics of GV might differ according to the frequency of measurement.

**Objectives:** To evaluate the correlation between GV indices derived from intermittent glucose values and from CGM.

**Methods:** A correlation between GV indices derived from intermittent readings (blood gas analyser [BGA]) and those derived from CGM was searched from data recorded in the same patients over the same period of time.

Data from patients recruited for the MANAGE (Manual vs. Automated Monitoring Accuracy of Glucose) II trial between July 2012 and April 2014 were analysed. Inclusion criteria were age >18 years, expected ICU length of stay of ≥ 3 days, an Acute Physiology and Chronic Health Evaluation (APACHE) II ≥ 10, hyperglycemia >150 mg/dl at the time of admission and need for a central venous catheter, one lumen of which was connected to Optiscanner® (Optiscan, Hayward, CA), a validated mid-infrared spectroscopy method of glucose measurement. GV indices (standard deviation [SD], glucose variability index [GVI], glucose lability index [GLI], mean amplitude of glycemic excursions [MAGE], J-index and maximal glucose change [MGC]) were calculated from blood glucose (in mg/dl) measured intermittently by BGA and by CGM over the same period. Pearson´s correlation coefficient between intermittent and CGM values was calculated for each metric. A Bland-Altman plot with bias (mean difference between the device and BGA measurements) and limits of agreement (bias ± 1.96 x standard deviation of the bias) was used to analyse the agreement between the device and the BGA results.

**Results:** Data from 88 patients were analysed. For each index, there was a significant correlation (p < 0.0001) between values calculated from intermittent readings and CGM. However, correlations were higher for J-index (0.96), SD (0.95) and GVI (0.9) than for MGC (0.88), GLI (0.82) and MAGE (0.77). The mean difference and limits of agreement (lower limit, upper limit) observed in Bland-Altman plot were 0.01 (−0.10, 0.12) for GVI, −0.43 (−34.34, 33.47) for MGC, −0.57 (−11.90, 10.76) for SD, 0.76 (−6.23, 7.74) for MAGE, −5.59 (−110.82, 99.64) for GLI and −1034.74 (−13524.05, 11454.57) for J-index.

**Conclusions:** The most reliable GV indices (highest correlation and lowest bias) are GVI and SD. These are the least influenced by the timing of measurement.

#### A517 Effects of near-continuous glucose monitoring as a guide for glycemic control: a cluster-randomized study

##### J.-C. Preiser, O. Lheureux, A. Thooft, S. Brimioulle, J.-L. Vincent

###### Erasme University Hospital, Université Libre de Bruxelles, Brussels, Belgium

####### **Correspondence:** J.-C. Preiser - Erasme University Hospital, Université Libre de Bruxelles, Brussels, Belgium

**Introduction:** Each of the three domains of dysglycemia, i.e. hyperglycemia, hypoglycemia and high glycemic variability is associated with poor outcome. The glycemic control with insulin to maintain blood glucose (BG) within a narrow range as currently recommended involves repeated checks of BG. Near-continuous intravascular monitoring (CGM) can represent a useful tool to facilitate and to improve glycemic control.

**Objectives:** To assess the effects of the use of CGM on the quality and safety of glycemic control.

**Methods:** Adult critically ill patients expected to stay at least 3 days in the department of intensive care of the Erasme University Hospital in Brussels, Belgium and presenting an hyperglycemia (BG > 150 mg/dl) up to 6 hours after admission and / or ongoing insulin therapy with a patent access to a large peripheral vein were equipped with a catheter connected to a enzymatic CGM sensor (GlucoClear®, Edwards Lifesciences, Irvine, CA). Two of the 4 units of the department were randomized to adjust insulin infusion rate to keep BG between 90 and 150 mg/dl (dynamic scale) using the values displayed on the monitor (unblinded group, U). In the 2 other units (blind group (B)), no value was displayed on the screen. The quality of glycemic control assessed by the proportion of time in range (TIR) and the safety assessed by the percentage of time spent below 70 mg/dl (TB70) were calculated from the values recorded by the CGM for each day and for the whole study period and compared between the two groups by ANOVA.

**Results:** Seventy-seven eligible patients (age 61 ± 8 years, 56 males, medical admission 74 %, APACHE II 24 ± 5, ICU mortality 23 %) consented to participate and were included in the U group (n = 39) or the B group (n = 38). 43107 BG values (22721 in the U group and 20386 in the B group) were recorded during 1 day (n = 77), 2 days (n = 64) or 3 days (n = 40). TIR did not differ between groups (70 ± 27 (U) vs 73 ± 23 % (B)), nor for any of the days of recording. However, the TB70 was lower in the U group than in the B group (0.4 ± 0.9 vs 1.6 ± 3.4 %, p < .05).

**Conclusions:** The use of a CGM-based strategy improved the safety of glycemic control in a mixed population of ICU patients with stress hyperglycemia.

#### A518 The effects of computer regulated continuous blood glucose management in diabetic patients underwent cardiac surgery

##### H. Iwasaka^1^, S. Tahara^2^, M. Nagamine^2^, A. Ichigatani^2^

###### ^1^Oita Almeida Hospital, Intensive Care Unit, Oita, Japan; ^2^Oita Almeida Hospital, Anesthesiology, Oita, Japan

####### **Correspondence:** H. Iwasaka - Oita Almeida Hospital, Intensive Care Unit, Oita, Japan

**Introduction:** It is well known that poor preoperative blood glucose control is associated with poor postoperative blood glucose control and postoperative complications. Postoperative hyperglycemia is common in patients underwent cardiac surgery, especially in those with a preoperative elevated HbA1c. The STG-55 (Nikkiso CO., Ltd., Tokyo, Japan) is a newly developed and commercialized computer regulated continuous blood glucose management apparatus.

**Objectives:** To compare the usefulness and the workload of ICU nurses of the STG-55 with the conventional sliding scale method in diabetic patients underwent cardiac surgery.

**Methods:** The study was approved by the Institutional Review Board, and written, informed consent was obtained from all participants. This study included 28 patients underwent elective cardiac surgery with preoperative HbA1c > 6.0 %. The management of blood glucose was performed from immediately after admission to the ICU for two research days. The patients were randomly assigned to control blood glucose levels with the STG-55 group (n = 16) or sliding scale method, SS group (n = 12). The usefulness of blood glucose management defined the incidence of hyperglycemia (blood glucose > 180 mg/dL), hypoglycemia (<80 mg/dL), and the maximum glycemic variability (maximum blood glucose level minus minimum blood glucose level). The workload of ICU nurses defined the number of blood samplings for the management of blood glucose and the number of calls made to the physician.

**Results:** The two groups were comparable with respect to age, gender, height, weight, duration of surgery, the amount of blood loss during surgery and the preoperative HbA1c. The blood glucose levels determined STG-55 and ABL3 acid–base laboratory analyzer were strongly correlated (R2 = 0.936), with nearly identical values. The incidence of hyperglycemia and hypoglycemia was significantly lower in the STG-55 group (1.9 ± 1.3 vs 6.7 ± 4.9 times/day, p < 0.001; 0.2 ± 0.1 vs 5.8 ± 6.0 times/day, p < 0.001) than SS group. The maximum glycemic variability was also significantly lower in the STG-55 group (46 ± 32 vs 283 ± 165 mg/dL, p < 0.01). The frequency of blood samplings (4.2 ± 3.7 vs 21.5 ± 14.1 times/day, p < 0.01), and the number of calls made to physician (2.1 ± 2.2 vs 6.9 ± 5.4 times/day, p < 0.05) were significantly lower in the STG-55 group than SS group.

**Conclusions:** Use of the STG-55 in the ICU contributed to improved blood glucose management and reduced workload of ICU nurses compared to using the sliding scale method.

**References**

1. Hasegawa A, et al. Surg Today 2001, 41: 1385–1390.

**Grant acknowledgement**

None.

#### A519 A physicochemical approach to acid–base balance in critically ill patients after infusion of seven different types of balanced fluids

##### A. Rugerio Cabrera, E. Monares Zepeda, J. Franco Granillo, J.S. Aguirre Sánchez, A.A. Tanaka Montoya, A. Pedraza Montenegro, G.A. Gálvez Blanco, C.M. Coronado Robles

###### The Americam British Cowdray Medical Center, Critical Care Department 'Dr. Mario Shapiro', Mexico City, Mexico

####### **Correspondence:** A. Rugerio Cabrera - The Americam British Cowdray Medical Center, Critical Care Department 'Dr. Mario Shapiro', Mexico City, Mexico

**Introduction:** Acid–base status in a body fluid is physically determined by several “independent variables”. These are: PCO_2_, the “strong ion difference” (SID) and all the strong anions (among them is Cl-), and concentrations of nonvolatile weak acids (Atot). Normal acid–base status is achieved when the independent variables have normal (empirically established) values. The Simplified Fencl-Stewart´s Method can be used at the bedside of the patient and is more accurate for the assessment of acid–base balance. Omron, E. developed a physicochemical model of the projected change in standard base excess (SBE) as a consequence of infused crystalloid solutions of common use (isotonic saline and balanced fluids); unfortunately this was a clinical simulation at standard physiological state. In addition, Kaplan, L. evaluated acid–base status after the administration of balanced fluids in trauma patients. Nevertheless, to our knowledge, there are no other clinical trials that evaluate de administration of other types of balanced fluids.

**Objective:** To assess acid–base status of critically ill patients after the infusion of seven different types of balanced solutions

**Methods:** This was a retrospective, observational and descriptive study, conducted in an intensive care unit of a tertiary care hospital. We included all patients above 18 years old admitted to this department from January 2014 to December 2015. We evaluated the effects on acid–base balance after the infusion of seven different solutions: 1) Hartmann + 17.8 mEq/L sodium bicarbonate (NaHCO_3_) (SID 45.8), 2) Hartmann + 8.9 mEq/L NaHCO_3_ (SID 36.9), 3) Hartmann + 15 mEq/L NaHCO_3_ (SID 43), 4) Hartmann + 25 mEq/L NaHCO3 (SID 53), 5) Hartmann (SID 28), 6) normal saline 0.45 % + 77 mEq/L NaHCO_3_ (SID 75), and 7) dextrose solution 5 % + 154 mEq/L NaHCO3 (SID 154). Arterial blood gases, serum electrolytes, and proteins were measured in the same blood sample. Also SIDa, SEDe, SIG, ATOT, pCO_2_, change in standard base excess (SBE), pH, [HCO_3_], [Na]p and SOFA were calculated. pH, SBE and PCO_2_ were estimated with the ABL8000 FLEX blood gas analyzer. Data are mean ± SD or percents. We used data analysis package SSPS.

**Results:** Ninety-nine patients were included. Of these, 54 % were women and 45 % men. The most used solutions were Hartmann (25 %), Hartmann + 8.9 mEq/L NaHCO_3_ (21 %), and Hartmann + 25 mEq/l NaHCO3 (18 %). Before the infusion, SIDe was under 30 mEq/L in 30 % of patients and above in 23 % of them. The effect on the SIDe was significant before the infusion of different solutions (p 0.01), SIDe > 30 ± 8 mEq/L. No metabolic alkalosis or greater decrease of SIDa/SIDe was observed.

**Conclusions:** This study assesses additional varieties of fluids that have a different SID in the clinical setting. No major acid–base disturbances were observed.

**References**

1. Vladimir, F. jabor, A. et al. Diagnosis of metabolic acid–base disturbances in critically ill patients. Am J Respir Crit Care Med. 2000; 162:2246–2251.

#### A520 Acid–base disturbances and their clinical relevance in critically ill patients with acute-on-chronic liver failure - a preliminary analysis

##### A. Drolz^1,2^, T. Horvatits^1,2^, K. Roedl^1,2^, K. Rutter^1,2^, S. Kluge^2^, G.C. Funk^3^, B. Schneeweiss^1^, V. Fuhrmann^1,2^

###### ^1^Medical University of Vienna, Internal Medicine III, Gastroenterology and Hepatology, Vienna, Austria; ^2^Medical University Center Hamburg-Eppendorf, Intensive Care Medicine, Hamburg, Germany; ^3^Otto-Wagner-Spital, Respiratory and Critical Care Medicine, Vienna, Austria

####### **Correspondence:** A. Drolz - Medical University Center Hamburg-Eppendorf, Intensive Care Medicine, Hamburg, Germany

**Introduction:** Severe acid–base balance abnormalities are frequently observed in critically ill patients at the intensive care unit (ICU) and can be present in different patterns. Acid–base profiles of critically ill cirrhotic patients with acute-on-chronic liver failure (ACLF) have not been investigated.

**Objectives:** To assess disturbances of acid–base status in critically ill patients with ACLF in comparison to patients without.

**Methods:** Preliminary analysis of patients admitted to medical ICU at the Medical University of Vienna. Presence of cirrhosis and ACLF, respectively, was assessed, and blood for laboratory- and blood gas analysis was drawn from an arterial line. Reference values were obtained from blood samples of healthy volunteers (n = 14). Mortality was assessed on site.

**Results:** A total of 615 patients were studied, 142 of these patients had underlying liver cirrhosis. One hundred twenty-eight patients fulfilled criteria of ACLF. Median SOFA score of the total cohort was 10 (IQR 6–13), median SAPS II score 53 (IQR 38–70) and median age 60 (IQR 49–69) years.

Patients with liver cirrhosis showed a marked metabolic acidosis compared to critically ill patients without (Base excess (BE) -6.8 (IQR −12.3 to 0.8) mmol/l vs. -1.5 (IQR −5.2 to 2.7) mmol/l, p < 0.001). Analysis of the BE subcomponents revealed that this acidosis was primarily attributable to hyperchloremia (BE_Cl_ = −4.6 (IQR −7.5 to 0.8) mmol/l), lactate (BE_Lactate_ = −2.7 (IQR −6.1 to −1.0) mmol/l), and unmeasured anions (BE_UMA_ = −1.6 (IQR −6.2 to 1.8) mmol/l). Interestingly, hyperchloremic acidosis antagonized by hypoalbuminemic alkalosis was found in critically ill patients with and without liver disease. In patients with cirrhosis, we observed respiratory alkalosis (partial pressure of arterial carbon dioxide (PaCO_2_) = 36.4 (IQR 28.5 - 44.5) mmHg) counteracting metabolic acidosis.

**Fig. 44 (abstract A520).**
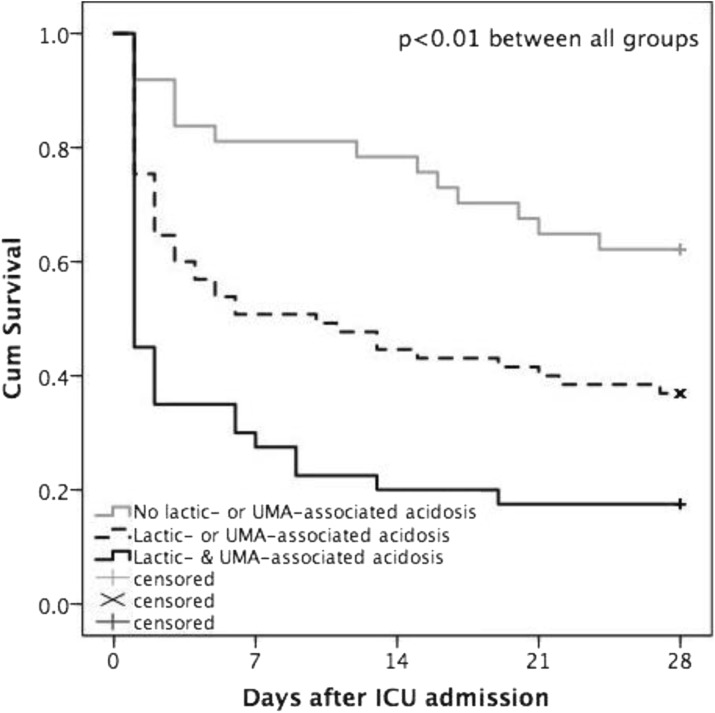
Kaplan Meier Plot: UMA means unmeasured anions

With progression to ACLF, the compensatory mechanisms (hyperventilation and hypoalbuminemia) became insufficient resulting in progression of metabolic acidosis. Accordingly, BE in patients with ACLF grade 3 was −10.1 (IQR −17.4 to −3.6) mmol/l with a resulting pH of 7.29 (IQR 7.14 to 7.41). Lactate (BE_Lactate_ = −3.6 (IQR −8.3 to −1.5) mmol/l) and unmeasured anions (BE_UMA_ = −3.9 (IQR −8.9 to 0.9) mmol/l) were main contributors to metabolic acidosis in ACLF grade 3. The extent of metabolic derangement attributable to lactate and unmeasured anions, respectively, in patients with cirrhosis was associated with 28-day-mortality (Fig. 44).

**Conclusions:** Metabolic acidosis is a common feature of cirrhosis and ACLF at the ICU and is more severe compared to patients without cirrhosis. Acidosis in ACLF is primarily attributable to lactate and unmeasured anions. Although hyperchloremic acidosis did contribute to metabolic acidosis in critically ill patients, we found no clinically relevant difference in chloride-related acidosis between patients with and without liver disease.

#### A521 Unmeasured anions in deceased donor: can they predict liver transplantation outcome?

##### G. Sabetian^1^, F. Pooresmaeel^2^, F. Zand^3^, S. Ghaffaripour^2^, A. Farbod^2^, H. Tabei^2^

###### ^1^Shiraz University of Medical Sciences, Trauma Research Center, Shiraz, Islamic Republic of Iran; ^2^Shiraz University of Medical Sciences, Shiraz, Islamic Republic of Iran; ^3^Shiraz University of Medical Sciences, Anesthesiology and Critical Care Research Center, Shiraz, Islamic Republic of Iran

####### **Correspondence:** G. Sabetian - Shiraz University of Medical Sciences, Trauma Research Center, Shiraz, Islamic Republic of Iran

**Introduction:** Liver transplantation is the treatment of choice in end stage liver disease, but significant mortality rate and organ shortage has resulted in multiple attempts to chose good donors and appropriate recipients. (1) The Stewart´s approach is a new concept in critical ill patients and can identify occult acid–base disturbances. (2,) Although study on Stewart´s approach were increased in recent years but its diagnostic and prognostic advantages remain contraversial. (3)

**Objectives:** Our primary objective was correlation between SIG (strong ion difference) in donor and graft function. Secondary objective was comparison between SIG and marginal graft criteria as a predictor for liver transplantation outcome.

**Methods:** In 51 donors, marginal graft criteria were determined. SIG in all cases were calculated and we followed mortality and complications in recepiants at 72 hr,1 week, 1 month after transplantation.

**Results:** 54.9 % of livers were marginal. Mean MELD Score in recipients was 21.25 ± 5.95 . According to calculated SIG value, recipients were divided into SIG > 10 and SIG < 10. This study showed that no difference was seen in these two groups(pvalue; 0.154) .Also no difference was seen in two groups.(marginal and non-marginal)(Pvalue = 0.245)**Conclusions:** In this study, SIG was not a good predictor for liver transplantation outcome.

**References**

1)Shaked A,Nones F,Olthoff KM,Lucey MR.Assessment of liver function:Pre and peritransplant evaluation.Clin Chem 1997; 43: 1539–1545.

2)Ali Y,Abouelnaga S,Khalaf H,Kamel Y.Physical chemical approach versus traditional technique in analyzing blood gases and electrolytes during liver transplant surgery.Transplantation Proceedings2010;42:861–864.

3)Dubin A,Menises MM,Masevicius FD,Moseinco MC,Daniela KDM,Ventrice E,et al. Comparison of three different methods of evaluation of metabolic acid–base disorder.Crit Care Med 2007;35(27):1264–1270.

**Grant acknowledgement**

Financial support was exclusively provided by Shiraz University of Medical Sciences.

### Ethics of end-of-life-care

#### A522 End of life care in haematology patients: a job for critical care outreach services?

##### L. Taheri, R. Anandanadesan, V. Metaxa

###### ^1^King's College Hospital (Denmark Hill), Intensive Care Medicine, London, UK

####### **Correspondence:** L. Taheri - King's College Hospital (Denmark Hill), Intensive Care Medicine, London, UK

**Introduction:** Despite recent medical advances and improved survival, patients with haematological malignancies (HM) still have a high mortality, and end-of-life (EoL) care has become an integral part of their treatment.^1^ Evidence suggests less palliative care involvement and limited advanced care planning in patients with HM, probably because of the unclear transition between curative and palliative phases of their disease.^1,2^ Critical Care Outreach Services (CCOS) have been shown to participate in more than 50 % of EoL planning as part of their everyday workload.^3^

**Objectives:** To evaluate the role and input of CCOS in EoL care of patients with HM.

**Methods:** We retrospectively reviewed the records of all patients with HM from January 2014 to October 2015 who were referred to CCOS in a London specialist hospital. Variables analysed included age, diagnosis, ICU admission, time spent by CCOS, interventions provided and in-hospital mortality.

**Fig. 45 (abstract A522).**
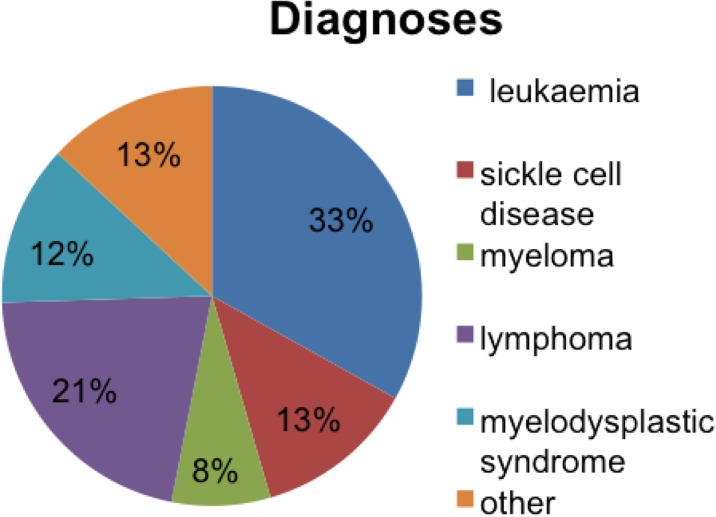
ᅟ

**Results:** There were 145 patients who were reviewed on 257 different occasions. Their age ranged from 18 to 84 years (median 56) and their diagnoses are shown in Fig. 45.

Of those, 16/145 (11 %) patients were identified as palliative; 81 % of them received EoL care on the ward. National Early Warning Scores at referral ranged from 2 to 10 (median 7). A total of 723 days was spent by CCOS in reviewing patients with HM, with 146 days (20 % of their clinical time) spent on palliative patients alone (median time 5.5d vs. 3d for non-palliative patients). Overall, in-hospital mortality among these patients was 81 %.

The services provided by CCOS to palliative patients were mainly facilitation of symptom control (67 %) and/or support of ward teams in making treatment limitation decisions (78 %). High Flow Nasal Cannula Oxygen (HFNC) was initiated in 38 % of patients for symptom control, which was deemed successful in all but 2 patients.

**Conclusions:** A considerable part of the CCOS workload is spent on supporting the management of palliative haematology patients. Involvement in alleviating symptoms and initiating EoL discussions features strongly in the interventions requested by the ward teams. The significant use of HFNC therapy for symptom control that was observed in this population also warrants further investigation.

**References**

1. Howell DA et al. Haematological malignancy: are patients appropriately referred for specialist palliative and hospice care? A systematic review and meta-analysis of published data. *Palliative Medicine* 2010;25:630–41

2. National Institute for Clinical Excellence. Improving outcomes in haematological cancers: the manual. London: National Institute for clinical Excellence. October 2003

3. Pattison N et al. Negotiating Transitions: Involvement of Critical Care Outreach Teams in End-of-Life Decision Making. *Am J Crit Care* 2015;24:232–40

#### A523 Contact with death, ethical decisions, and communication of bad news in intensive care and palliative units: results from a mixed-methods study

##### C. Teixeira^1,2,3^, S.M. Pereira^1^, P. Hernández-Marrero^1,4^, A.S. Carvalho^1^

###### ^1^Universidade Católica Portuguesa, Instituto de Bioética, Porto, Portugal; ^2^Centro Hospitalar do Porto, Hospital de Santo António, UCIP-Departamento de Anestesia e Cuidados Intensivos, Porto, Portugal; ^3^Universidade do Porto, Instituto de Ciências Biomédicas Dr. Abel Salazar, Porto, Portugal; ^4^Servicio Canario de Salud, Porto, Portugal

####### **Correspondence:** C. Teixeira - Universidade do Porto, Instituto de Ciências Biomédicas Dr. Abel Salazar, Porto, Portugal

**Introduction:** Professionals working in intensive care and palliative units (ICUs/PCUs) care for patients with life-threatening diseases, make ethical decisions, and provide end-of-life care. However, while palliative care aims to reduce suffering, intensive care has a major focus on saving lives.

**Objectives:** To identify and compare the experiences of ICU and PCU healthcare professionals related to: contact with dying and death, making of ethical decisions and communication and delivery of bad news.

**Methods:** Mixed approach, combining quantitative (questionnaire on experiences in the work context) and qualitative ones (interviews with doctors and nurses). 10 ICU and 9 PCU participated in this study. 392 professionals completed the survey; 28 were interviewed. A descriptive quantitative analysis was performed; the chi-square test was used to analyse the association between variables (significance level of p < .05). Interviews were subject to content analysis.

**Results:** In the week prior to survey completion, more professionals working in ICUs reported a patient's death; this was not statistically significant. The experience most mentioned by the professionals of both types of units during interviews was caring for patients nearing death. In the week before completing the questionnaire, the most common ethical decision was palliative/terminal sedation; this was more, often in ICUs (27 % vs. 12 %; p = .004). In the day of questionnaire completion, the most frequent ethical decision was also palliative sedation. Though this decision was more frequent in ICU, statistical significance was not reached (p = .440). The communication of the diagnosis/prognosis to the patient, either in the week before or in the day of questionnaire completion was more frequent in PCUs (45 % vs. 29 %, p = .005; 22 % vs. 12 %, p = .026, respectively). Communication about the diagnosis/prognosis with the family in the week before survey completion was held with equal frequency by professionals from both contexts (58 % of professionals). Although not reaching statistical significance (p = .303), more professionals from PCU proceeded to communication with family about the diagnosis and prognosis (32 % vs. 26 %) in the survey day. From the analysis of the interviews, it was denoted that it were mainly professionals of PCU who referred to the communication on the diagnosis/prognosis, both with the patient as with the family.

**Conclusions:** The workplace experiences in ICU and PCU are, despite some differences, guided by similarities. Caring for patients with life-threatening situations and imminent death and the need to make ethical decisions occur frequently in both contexts. The communication about the diagnosis/prognosis occurs more often in PCU. This highlights the need for integrating communication strategies of palliative care, in intensive care.

**References**

1. Curtis JR, Vincent JL. Ethics and end-of-life care on the adult intensive care unit. *Lancet*. 2010 Oct 16;376(9749):1347–53. Epub 2010 Oct 11.

#### A524 An opportunity for advance decision-making: pre-operative risk stratification for adverse ICU outcomes in elective surgical patients

##### M. Beckmann^1^, C.S. Hartog^2^, D. Schwarzkopf^2^, A. Raadts^1^

###### ^1^Jena University Hospital, Anaesthesiologyy and Intensive Care, Jena, Germany; ^2^Jena University Hospital, Center for Sepsis Control and Care, Jena, Germany

####### **Correspondence:** M. Beckmann - Jena University Hospital, Anaesthesiologyy and Intensive Care, Jena, Germany

**Introduction:** Complex elective surgery is increasingly provided to an aging population with heightened risk for prolonged ICU stay or death. While risks of surgical and anaesthetic procedures are routinely discussed preoperatively in the process of obtaining informed consent, the patients' preferences in case of unfavourable ICU outcomes are rarely discussed and most critically ill patients do not have advance directives. If patients at risk of a prolonged ICU stay or death could be identified preoperatively, this would provide an opportunity to make their preferences known in a timely and adequate manner.

**Objectives:** To identify the predictive validity of routine pre-operative risk assessment for an unfavourable ICU course (ICU stay > 24 hours or death), and to assess the prevalence of advance directives in these patients.

**Methods:** Among all 13437 elective adult surgical cases seen in a tertiary university hospital´s pre-operative anaesthesiology clinic in 2014, 1832 consecutive cases were drawn. Data were extracted from hospital and ICU databases, including patient demographics, pre-operative American Society of Anesthesiologists (ASA) classification, length of ICU stay, mortality and presence of advance directives. A receiver operating characteristic analysis was conducted to test the predictive validity of ASA for a) having an ICU stay > 24 h and b) dying in the ICU or afterwards. Optimal cut-offs were identified by maximum Youden's J statistic.

**Results:** Among 1832 patients, 937 (51 %) were male, median age was 63 years (interquartile range 49–74), planned procedures were mainly from General, Trauma, Cardiothoracic, Eye, Gynecological, Urological or ENT Surgery. Pre-operatively, patients were classified into ASA risk classes (15 %, 41 %, 40 % and 4 % into ASA 1–4, respectively). Postoperatively, 504 (28 %) patients were admitted to the ICU. Of these, 373 (74 %) had an ICU LOS > 24 hours and 68 (13 %) died in the ICU or afterwards. Among patients with an ICU stay > 24 hours, presence of an AD was documented in 49 (15 %) and power of attorney for a legal proxy in 71 (22 %). Pre-operative ASA classification predicted an ICU stay >24 h with an area under the curve (AUC) of 0.79, and death in the ICU or afterwards with an AUC of 0.85. The optimal cut-off for both adverse outcomes was ASA ≥ 3. For ICU stay >24 h sensitivity was 0.85, specificity was 0.67; for death in or after ICU sensitivity was 0.93 and specificity was 0.58.

**Conclusions:** Preoperative ASA classification with a cut-off of ≥ 3 has a good predictive validity to identify the risk of prolonged ICU stay or death in patients undergoing elective surgery. Such risk stratification could be useful to initiate advance decision-making at the time of the pre-operative work-up of these patients and thus increase the prevalence of adequate ADs in the ICU.

**Grant acknowledgement**

Funded partially by the Federal Ministry of Education and Research (BMBF), Germany, grant no: 01EO1002.

#### A525 Treatment-limiting-decisions in patients with severe traumatic brain injury in a Norwegian trauma hospital

##### A. Robertsen^1^, R. Førde^2^, N.-O. Skaga^1^, E. Helseth^3^

###### ^1^Oslo University Hospital, Anesthesiology and critical care, Oslo, Norway; ^2^University of Oslo, Center of medical ethics, Oslo, Norway; ^3^Oslo University Hospital, Neurosurgery, Oslo, Norway

####### **Correspondence:** A. Robertsen - Oslo University Hospital, Anesthesiology and critical care, Oslo, Norway

**Introduction:** Studies have shown variations in practice across hospitals regarding treatment-limitations and mortality for brain-injured patients.

**Objectives:** To study treatment-limitations and associated mortality in a Norwegian trauma hospital and documentation of ethical aspects such as presence of advanced directives, dialogue with families, multi-team discussions, reasons and considerations behind decisions*,* conflicts and involvement of clinical ethics committees.

**Methods:** A retrospective study of a 2-year cohort of severe head injured patients admitted 2011–12 to Oslo university hospital, Norway. Trauma registry data were combined with data from medical records. For data validation a definition guide for study variables was developed. Adults with abbreviated injury score head 4,5,6 were included (n = 579).

**Results:** Eighty-five % of all patients were admitted to ICU. Treatment limitations were identified in 17 % of cases (101 patients). Decisions were: Withholding organ support (12 cases), withholding surgery (52 cases), withdrawing intracranial pressure-targeted therapy (23 cases), DNR-orders (44 cases), no-escalation of treatment (19 cases) or withdrawing organ support (44 cases). For some patients initial decisions were changed (19 cases) or revoked (3 cases) along the dynamic treatment trajectory. Twenty-six patients with devastating brain injury progressed to brain death. No patients had advanced directives. Dialogue with family was documented in most cases (98). No major conflict between families and treatment team was identified and there was no involvement by CEC. Rationale behind decisions was identified as medical only in 80 % of cases. Treatment-limitations followed situations categorized as futile (59 cases) or “potentially inappropriate treatment” (42 cases) (1). The overall 30-day mortality was 16 % (35 % for patient with GCS < 9 and 7,5 % for patients with GCS >8). Treatment-limitations were identified in 93 % of cases of in-hospital death. In-hospital mortality was 73 %, 30-day mortality 82 % and 2-year mortality 93 % in the treatment-limiting group (n = 101 patients, 25 patients were transferred to other facilities with limitations). In-hospital mortality was 1 %, 30-day mortality 2 % and 2-year mortality 8 % for patients without limitations made at the trauma hospital (n = 478).

**Conclusions:** Treatment limitations are common in patients with traumatic brain injury and were closely associated with in-hospital death. Withholding or withdrawing life-sustaining therapy in this early phase of hospitalization was primarily based on the medical situation and at the discretion of the physician. Whether patients' values and preferences were adequately addressed or had an impact on decisions (when appropriate) remains unclear.

**References**

1. Rubin, M. A. and J. Bonomo (2016). "Neurocritical Care Society Views on "Potentially Inappropriate Treatments in Intensive Care Units"." American Journal of Respiratory and Critical Care Medicine **193**(4): 466–467.

#### A526 Ethical considerations when performing life saving but non restorative neurosurgical intervention. The oracle stroke study

##### S. Honeybul^1^, K. Ho^2^

###### ^1^Sir Charles Gairdner Hospital, Neurosurgery, Perth, Australia; ^2^Royal Perth Hospital, Perth, Australia

####### **Correspondence:** S. Honeybul - Sir Charles Gairdner Hospital, Neurosurgery, Perth, Australia

**Introduction:** This study assessed opinion of healthcare workers about consent and acceptable outcome in the context of decompressive hemicraniectomy for 'malignant cerebral artery infarction”

**Method:** Seven Hundred and seventy three healthcare workers at the two major public neurosurgical centres in Western Australia participated in this study. Participants were asked to record their opinion regarding consent and acceptable outcome based on the Modified Rankin Score (mRS). They were then given a detailed analysis of the evidence for clinical efficacy of the procedure and an explanation of the “disability paradox”. They were then asked to reconsider their initial responses.

**Results:** Of the 773 participants included in the study 407 (52.7 %) initially felt that they would provide consent for a decompressive craniectomy as a life-saving procedure but only a minority of them considered mRS 4 or 5 as an acceptable outcome (for mRS ≤ 4: n = 67, 8.7 %; for mRS = 4: n = 57, 7.4 %). After introducing the concept of the disability paradox and the evidence for the clinical efficacy of decompressive craniectomy, more participants were unwilling to accept decompressive craniectomy (18.1 % vs. 37.8 %) but, at the same time, more were willing to accept mRS ≤ 4 as an acceptable outcome (for mRS ≤4: n = 92, 11.9 %; for mRS = 4: n = 79, 10.2 %).

**Conclusion:** Most participants felt that survival with dependency to be unacceptable. However, many would appear to be willing to provide consent for surgery in the hope that they may survive with some degree of independence.

#### A527 Analysis of patients assessed but not admitted to ICU of a secondary hospital in Malaga

##### P. Martinez Lopez, M. Nieto Gonzalez, P. Nuevo Ortega, E. Camara Sola, T. Spasova, M.V. de la Torre-Prados

###### Virgen de la Victoria Hospital, Intensive Care, Málaga, Spain

####### **Correspondence:** M. Nieto Gonzalez - Virgen de la Victoria Hospital, Intensive Care, Málaga, Spain

**Objective:** To analyze why patients who are assessed by an intensivist physician for some reasons are not admitted to ICU. Also we want to find out what happened to this patients after intensivist evaluation.

**Design:** Observational and prospective study during 12 consecutive months.

Scope: Second level Hospital with 550 beds and 18 ICU boxes.

**Method:** We searched daily for patients who were assessed but not admitted to ICU during 12 consecutive months. We write down medical record number, which of the hospital departments asked for that ICU evaluation and the reason why the patient was not finally admitted to ICU (low severity, life-sustaining treatment limitation or not ICU bed availability). We follow up those patients until hospital discharged, finding either decease, hospital discharge or later ICU admission.

**Results:** 1297 patients were assessed but not admitted to ICU. The reasons for not admission were low severity in 736 patients (56.8 %), life-sustaining treatment limitation in 459 patients (35.5 %) and no ICU bed availability in 102 situations (7.9 %). Main hospital departments that asked for assessment without consequent ICU admission were Emergency (N = 899, 69 %), Internal Medicine (N = 140, 10.8 %) and General Surgery (N = 62, 4.8 %). Life-sustaining treatment limitation decision was mostly taken in Emergency Department (N = 253) and Internal Medicine (N = 83), 166 cases survived and were discharged from hospital (36 %). 736 patients were dismissed because of low severity, but 91 were finally admitted to ICU later (12.3 %) and 125 patients died (16.9 %). 102 patients were not admitted because of no ICU bed availability, majority were treated in Observation Room in Emergency Department (N = 94, 92.2 %); 29 of them were admitted to ICU later (28.4 %) and 20 died (19.6 %).

**Conclusions:** From these results we conclude that maybe the lack of beds in ICU can generate a belated admission to ICU and higher mortality rate in recoverable patients. One third of patients with a life-sustaining treatment limitation decision finally survive the hospitalization. Also we conclude that a considerable amount of intensivist clinical assistance during the shifts takes place outside de ICU.

#### A528 Oddicus - how much care is at odds with patient's values and preferences at the end of life?

##### O. Kopecky^1^, K. Rusinova^1^, P. Waldauf^2^, Z. Cepeplikova^1^, M. Balik^1^

###### ^1^General University Hospital, 1st Faculty of Medicine, Charles University in Prague, Department of Anaesthesia and Intensive Care Medicine, Prague, Czech Republic; ^2^3rd Medical Faculty, Charles University, Department of Anaesthesia and Intensive Care Medicine, Prague, Czech Republic

####### **Correspondence:** K. Rusinova - General University Hospital, 1st Faculty of Medicine, Charles University in Prague, Department of Anaesthesia and Intensive Care Medicine, Prague, Czech Republic

**Introduction:** Addressing the quality of end of life care has become an important focus of research and patient management in the ICU. Although quality of care and support for patients who die in the ICU and their families are well described, a little is known about the decision making process and the quality of care provided for patients dying outside the ICU.

**Objectives:** To characterize the care for patients dying during the last hospitalization in terms of circumstances of death, provided palliative care, medical decision making, nurses perceptions and family experience.

**Methods:** A prospective observational cohort study in two tertiary university centers in Prague, Czech Republic. Data from the patient documentation and semi-structured questionnaire with the attending physician and the nurse present at the moment of death were collected. A semi-structured phone interview with the family member was held 3–6 months after the death. The study has received an IRB approval.

**Results:** Among 44236 patients hospitalized between February 2015 and February 2016, 1097 has died, of them 926 patients (86,4 %) were enrolled in the study (42.1 % in the ICU and 57.9 % at wards). The basic patients´ characteristic is presented in the Table 20. The majority of deaths in the hospitals were expected and hence not preceded by a cardiopulmonary resuscitation. The mention of a poor prognosis was documented in a majority of cases, the DNR and DNI being the most frequent type of treatment limitation both in ICU and wards. The treatment limitation was communicated with the family fare more often than to the patient (81 % and 31 %, respectively). Patients did not have an advance directives document, nor were their treatment preferences documented during the last hospitalization (0,5 and 1,62 %, respectively). The various aspects of treatment limitations in the ICU vs. wards are presented in Table 21.

**Conclusions:** This is the first large prospective study comparing the decision making process and the perceptions of clinicians and family members of patients dying in a tertiary care hospital. The study shows an insufficient interdisciplinary and intra-team communication with discrepancies between the documented and the agreed treatment limitations. Furthermore, a difficult communication with patients and their families in final phases of patients´ lives suggests a gap between the care provided and the patients preferences regarding the end-of-life, although these are known to be key aspects of good end-of-life care.

**References**

1. Piers RD, Azoulay E, et al. Inappropriate care in European ICUs: confronting views from nurses and junior and senior physicians. Chest. 2014;146(2):267–75.

2. Rusinova K, Kukal J et al. Limited family members/staff communication in ICUs in the Czech and Slovak Republics considerably increases anxiety in patients´ relatives. BMC Psychiatry. 2014:27;14–21

**Grant acknowledgement**

This study was supported by AVAST foundation.

**Table 20 (abstract A528).** Patient characteristics

Characteristic of the patients	N=926
Age (mean; ±SD)	75±13.8
Charleson comorbidity scale (mean;±SD)	6.9 ±2.7
Performance status at hospital admission on the scale from 0 to 4	0–1.24% 1–4.8% 2–9.45% 3–30.3% 4–54.2%
Advance directives (%)	0.5
Length of the last hospitalization (median days; IQR)	8 (2–19)

**Table 21 (abstract A528).** Comparison of treatment limitations: ICU vs. wards

	ICU (N= 390)	Wards (N=536)	Logistic regression, (OR, 95% CI), p
Documented poor prognosis	90,7%	88.3%	1.3 (0.8;2.1), p=0.3
Death expected during current hospitalization (perception of the physician)	89,3%	84.6%	1.5 (1.01;2.3), p=0.044
Palliative care provided (perception of the physician)	41,9%	62.8%	0.5 (0.4;0.7), p<0.001
Documented treatment limitation	DNR: 88.4%; DNI: 47.6%; no RRT: 31.1%; no catecholamines: 25,8%	DNR: 72.9%; DNI: 56,8%; No ICU transfer 19.4%; No antibiotics 6.5%	2.8 (1.8;4.4), p<0.001; 0.7 (0.5;0.95), p=0.024; NA; NA
Limitation of treatment never discussed among staff	31,5%	36.6%	0.8 (0.6;1.05), p=0.112
CPR 24h prior death	13,8%	3,7%	3.9 (2.3;6.6), p<0.001
Nurse involved in decision 4.5	4,5%	3.2%	1.4 (0.6;3.1), p=0.385
Info about the treatment limit: − to the family - to the nurse - to the patient	76.3%; 98.3%; 40.0%	81.4%; 96.0%; 31.1%	0.7 (0.5;1.1), p=0.114; 2.4 (0.9;6.9), p=0.098; 1.5 (0.5;4.6), p=0.5
Family present at the moment of death	15,8%	10,7%	1.6 (1.06;2.3), p=0.024

#### A529 Analgesia and sedation after limitation of life sustaining treatment thru terminal extubation. A comparison between controlled cardiac death donors and non-donors

##### J. Palamidessi Domínguez, P. Matia Almudevar, S. Alcántara Carmona, J.J. Rubio Muñoz, D. Palacios Castañeda, A. Naharro Abellán, P. Rodríguez Villamizar, J. Veganzones Ramos, L. Pérez Pérez, A. Pérez Lucendo, M. Camós Ejarque

###### Hospital Universitario Puerta de Hierro Majadahonda, Madrid, Spain

####### **Correspondence:** J. Palamidessi Domínguez - Hospital Universitario Puerta de Hierro Majadahonda, Madrid, Spain

**Objective:** To compare the different strategies of analgesia and sedation applied in control cardiac death donors (DCD) versus non-donors after limitation of life sustaining treatment (LLST) thru terminal extubation (TE).

**Methods:** Retrospective study (January 2012 - March 2016) in a Spanish tertiary hospital. We studied all patients that underwent LLST thru TE. Demographic factors, general patient characteristics (comorbidities, diagnosis upon ICU admission, APACHE II on admission and SOFA in the previous 24 h before TE), ICU length of stay and timing from TE to cardiac arrest together with their donor/non-donor condition were recorded. We also analyzed the different types and doses of analgesic and sedation drugs (morphine, fentanyl, remifentanyl, propofol and midazolam) and the different strategies used (bolus administration, increase of the infusion rate or both). In order to simplify dosage analysis we used the intervals previously described by other authors^1^. Statistical analysis with c^2^ and Mann–Whitney *U* test. Statistical significance p < 0,05.

**Results:** During the period studied we recovered a total of 68 patients with LLST thru TE that were divided in two groups: DCD (group A, n = 26) and non-donors (group B, n = 42). Demographic data and general characteristic are described in Table 22.

**Fig. 46 (abstract A529).**
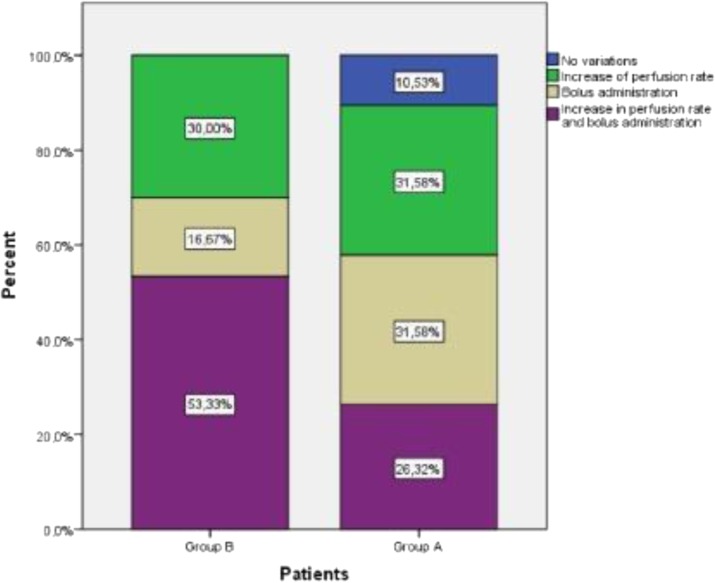
ᅟ

There were no statistical differences regarding the different types and doses of analgesia and sedation, and the different strategies used in group A vs group B (Table 23 and Fig. 46). Although none of the following reached statistical significance we found that the number of patients receiving doses described as superior to standard accounted for an important percentage in both groups, and that there was a higher rate of bolus administration followed by an increase in infusion rate un group B (53,3 %) when compared to group A (29,4 %).

**Conclusion:** In our study we found no differences in dose, type and strategy applied for analgesia and sedation after TE regardless of whether the patient was a potential organ donor or not.

**References**

1. Troug RD et al. Recommendations for end-of-life care in the intensive care unit: A consensus statement by the American College of Critical Care Medicine. Crit Care Med 2008 Vol. 36, No. 3

**Table 22 (abstract A529).** Demographic data and general characteristics

	Group A	Group B
Age (mean)	53±12 years	66±15 years*
Presence of comorbidity	73%	90% (p 0,058)
Specific comorbidities:		
− Cardiovascular risk factors − Heart disease − All other comorbidities without differences between groups	46% 4% (1 patient)	76% 31%
Main diagnosis upon ICU admission (without differences between groups)	1*) Cardiac arrest; 42,3%	1*) Cardiac arrest; 38,1%
	2*) Ischemic stroke and respiratory pathology: 15,4%	2*) Hemorrhagic stroke; 26,2%
	3*) Hemorrhagic stroke: 7,7%	3*) Ischemic stroke: 9,5%
APACHE II on admission (mean)	24±6 points	27±8 points
SOFA 24 hours before TE (mean)	7±2 points	9±3 points*
ICU length of stay (mean)	12±11 days	5±5 days*
Timing from TE to cardiac arrest (median)	11 (9 to 17) minutes	18 (10 to 105) minutes*

*p<0,05

**Table 23 (abstract A529).** Analgesia and sedation practices

	Group A	Group B
Standard dose	15,8%	16,7%*
High dose	84,2%	83,3%*
Bolus OR increase of perfusion rate	70,6%	46,7%*
Bolus AND increase of perfusion rate	29,4%	53,3%*

*p>0,05

#### A530 National survey about bioethics quality indicators in spanish intensive care units

##### A. Estella^1^, V. Lopez Camps^2^, M.C. Martín^3^, N. Masnou^4^, Bioethics work group of SEMICYUC.

###### ^1^Hospital del SAS de Jerez, Intensive Care Unit, Jerez de la Frontera, Spain; ^2^Hospital de Sagunto, Intensive Care Unit, Sagunto, Spain; ^3^Hospital Universitario Torrejón de Ardoz, Intensive Care Unit, Madrid, Spain; ^4^Hospital Universitario de Girona, Intensive Care Unit, Girona, Spain

####### **Correspondence:** A. Estella - Hospital del SAS de Jerez, Intensive Care Unit, Jerez de la Frontera, Spain

**Introduction:** Quality of care is a key aspect of intensive care medicine. Spanish Society of Intensive and Critical Care and Coronary Units (SEMICYUC) published in 2011 quality indicators in critically ill patients.

**Objectives:** The aim of the present study was to analyze the degree of compliance of the six bioethics quality indicators in Spanish ICU.

**Methods:** Multicenter survey in Spanish ICU. During a time of 30 days a questionnaire was sent to local investigators of ICU. Question were related with the six bioethics quality indicators published by SEMICYUC: Indicator 96: Adequacy of care at the end of the life. Indicator 97: Information to patients and their relatives in ICU. Indicator 98: advance directives in decision makings. Indicator 99: Informed written consent forms corrected filled out . Indicator 100: Limitation life support. Indicator 101: Restraints applications. The data were analyzed using SPSS version 15 for Windows.

**Results:** 68 ICU participate. 50 belongs to public health services and 14 with other financial management. 70,6 % were university hospitals. Indicator 96: 44 % have end of life protocol available. Consensus decisions with participation of nurses in 79,6 %. Indicator 97: Daily information in 97 %, 82 % have specific room for information, in 92 % information is given by the doctor and in 61 % information is written in the medical record. Indicator 98: Advance directives 53 %. Indicator 99: percentage of informed written consent forms correctly filled out was variable according type of procedure. Indicator 100: 48 % have a limitation life support written form. Indicator 101: 40 % have a restraint application protocol.

**Conclusions:** Best compliance was observed in the indicator related with the information in ICU.

We must to improve variability observed about written inform consents and to promote advance directives in clinical decision making.

Bioethics quality of care in ICU take on great importance.

**References**

1. Quality indicators in critically ill patients. Madrid (Spain): Spanish Society of Intensive and Critical Care and Units Coronary (SEMICYUC);2011. 185p.

#### A531 Religious beliefs may be associated with poor comprehension of end-of-life decisions

##### S. Barbosa^1^, A. Varela^1^, I. Palma^1^, L. Cristina^1^, E. Nunes^1^, I. Pereira^1^, G. Campello^2^, C. Granja^1,3,4^

###### ^1^Algarve Hospital Center, Faro Unit, Emergency and Intensive Care Department, Faro, Portugal; ^2^Algarve Hospital Center, Portimão Unit, Emergency and Intensive Care Department, Portimão, Portugal; ^3^University of Algarve, Department of Biomedical Sciences and Medicine and CINTESIS-UALG, Faro, Portugal; ^4^Faculty of Medicine of Porto, CINTESIS, Porto, Portugal

####### **Correspondence:** S. Barbosa - Algarve Hospital Center, Faro Unit, Emergency and Intensive Care Department, Faro, Portugal

**Introduction:** Despite technological advances that allow the support of organ and prolongs life, death in intensive care units is frequent. However, the success of care should not only be measured by survival, but should include the quality of life of each patient who survives and the comfort of the terminally ill.

**Objectives:** To evaluate the comprehension of nurses and physicians concerning end-of-life decisions.

**Methods:** The study consisted of a cross-sectional survey with a self-completed questionnaire applied to nurses and physicians on a voluntary and anonymous basis. The questionnaire was constructed around the meaning of end-of-life decisions: withdrawal and withhold life-sustaining treatments and do not resuscitate decisions.

**Results:** A total of 174 questionnaires were delivered and 109 were returned (response rate of 63 %). However, two of the questionnaires were excluded by multiple responses to each question. Seventy-one percent were nurses, 29 % were physicians and 59 % were female; the median age was 35 years-old and the median years of practicing was 11; 64 % had religious beliefs (53 % of the physicians and 68 % of the nurses). The comprehension of end-of-life decisions was noted in 86 % of the physicians and nurses: 93 % comprehended the meaning of withdrawal and withhold life-sustaining treatments and 97 % comprehended the meaning of do not resuscitate decision. There were no significant statistical differences in the comprehension of end-of-life decisions between physicians and nurses. The religious belief was a factor significantly associated with poor comprehension of end-of-life decisions (*p* 0,044), namely withhold life-sustaining treatment.

**Conclusions:** In this study, we found that not all physicians and nurses of an emergency and intensive care department comprehend the meaning of end-of-life decisions. Religious belief was significantly associated with poor comprehension of en-of-life decisions, namely withhold life-sustaining treatment. This study draws our attention to the need to improve training and education about end-of-life decisions in the emergency and intensive care units and to conduct further studies to evaluate the comprehension of the nurses and physicians on other departments of the hospital.

**References**

1- Granja C, Teixeira-Pinto A, Costa-Pereira A. Attitudes towards do-not-resuscitate decisions: differences among health professionals in a Portuguese hospital. Intensive Care Med 2001; 27:555–558.

2- Carlet J, Thijs L, et al. Challenges in end-of-life care in the ICU. Statement of the 5^th^ International consensus conference in critical care: Brussels, Belgium, April 2003. Intensive Care Med 2004 30:770–784.

#### A532 Family participation in end of life care discussions in an Indian hospital

##### R. Pande^1^, M. Pandey^2^, S. Varghese^1^, M. Chanu^1^

###### ^1^BLK Superspeciality Hospital, Critical Care Medicine, New Delhi, India; ^2^Lady Hardinge Medical College, Anaesthesiology, New Delhi, India

####### **Correspondence:** R. Pande - BLK Superspeciality Hospital, Critical Care Medicine, New Delhi, India

**Introduction:** Withholding or withdrawal has been reported in 19-50 % of deaths in Indian ICUs, withdrawal being limited to only 8 % of cases^1^. Various reasons have been identified as barriers to end of life care in India^2^. A recent physician interview based Asian multinational study has highlighted significant difference in such practices^3^.

**Objectives:** This study was undertaken to look at the attitude of Indian families towards end of life care (EOL) issues.

**Methods:** A standard protocol for family discussion was developed. Relevant end of life care issues was discussed in presence of admitting physician, ICU nurse, medical administrator and members of ICU team. The filled up EOL family discussions forms related to 44 critically ill adult patients admitted to ICU between March 2010 & March 2011 were retrospectively analyzed. The discussions held during non-regular hours were excluded.

**Results:** The data included 26 male and 18 female patients, admitted with a medical diagnosis (n = 18), surgical (n = 4), trauma (n = 6) or cancer (n = 16). The age of patients varied from 20–40 years (22.7 %, n = 10), 40–60 yrs. (18 %, n = 8), 60–80 yrs. (36.36 %, n = 16) and 10 patients (22.7 %) were >80 years of age. In majority of the discussions more than 3 members of the family participated (n = 29), 2 members (n = 12) and 1member in 3 patients.

All families participated in end of life care discussions. They understood and agreed on a situation of medical futility and irreversibility in their loved ones and 79.54 % (n = 35) families requested for no further escalation in treatment. 43 % (n = 19) families agreed for No CPR orders, whereas 20.45 % (n = 9) agreed for Do not intubate and do not ventilate orders. Only 4.54 % (n = 2) families agreed for withdrawal of ventilator, 9 % agrees for withdrawal of vasopressors and only 1 family (2.27 %) agreed for organ donation. Majority of the families (90.9 %, n = 40) tried to understand the measures given to alleviate pain and the feeling of dyspnoea related to ventilator withholding or withdrawal.

**Conclusions:** Our study shows willingness of families to participate in end of life care discussions and accepting medical futility and irreversibility of such situations. Withholding life care was more acceptable than withdrawing.

**References**

1. Kapadia F, Singh M, Divatia J et al. Limitation and withdrawal of intensive therapy at end of life: practices in intensive care units in Mumbai, India. Crit Care Med. 2005; 33(6):1272–5.

2. Mani RK. End-of-life care in India. Intensive Care Med. 2006;32(7):1066–8.

3. Phua J, Joynt GM, Nishimura M et al. Withholding and withdrawal of life-sustaining treatments in low-middle-income versus high-income Asian countries and regions. Intensive Care Med 2016. DOI 10.1007/s00134-016-4347-y

#### A533 Development and evaluation of an e-learning to enhance advanced care planning discussion by residents in a university medical center, a pilot study

##### M.J. Van Dam^1,2^, E.W.M.T. Ter Braak^2,3^

###### ^1^University Medical Center Utrecht, IC Center, Utrecht, Netherlands; ^2^University Medical Center Utrecht, Educational Center, Utrecht, Netherlands; ^3^University Medical Center Utrecht, Internal Medicine, Utrecht, Netherlands

####### **Correspondence:** M.J. Van Dam - University Medical Center Utrecht, Educational Center, Utrecht, Netherlands

**Introduction:** Advance care planning (ACP) is the process in which the patient makes decisions in consultation with health care givers about future health care once they become incapable of deliberate medical treatment decisions. ACP is increasingly recognized as area of concern respecting quality of care in the ICU and other wards in the hospital. However, research shows that ACP discussions (ACPd) are still rare in hospital settings: clinicians underestimate the number of patients willing to discuss ACP and the feeling being unskilled, inadequately trained or inexperienced may hamper a meaningful patient-doctor discussion.

**Objectives:** To develop and evaluate an e-module preparing residents to perform effective and timely ACPd.

**Methods:** Development of an e-module aiming to equip residents with knowledge, skills and appropriate attitudes for ACPd, preferably in a simulated situation before real life practice in the workplace. The design of this e-module is inspired by the 4C/ID-model. First, experts explain the ethical, legal and social issues concerning ACP that constitute the learning goals of the e-module. Subsequently, five cases are presented in videos illustrating appropriate performance. Each case is provided with comments from experts and residents and their advice based on personal experience. Cases are presented in increasing complexity sequence. Roles of patients are performed by trained simulation patients. Residents, senior staff and nurses demonstrate lifelike ACPd designed to illustrate preset educational goals.

Documenting ACP agreements in the electronic health record is shown using screen shots. Finally, background information by experts and useful links are offered. Learners work through the e-module at their own pace with the option to interrupt and looking back. The e-module was piloted in a group of residents purposefully sampled from a variety of specialties with a web-based 5 point Likert scale survey with room for narrative explanation to evaluate satisfaction of participants.

**Results:** In this pilot 18 residents participated, mean age 30.4 (+/− SD 2.6) year, 33 % male, in training for neurology (n = 5), internal medicine (n = 5), surgery (n = 3), anesthesiology (n = 2), cardiology (n = 1) and undisclosed (n = 2), ranging from 1^st^ to 6^th^ year of training (median 3^rd^). 83 % of participants (strongly) agreed with the statement that the e-module is well-arranged. 89 % (n = 16) review the e-module as giving sufficient insight in the necessity of ACPd. Ten residents (55 %) appraise the e-module offering guidance for performing ACPd themselves in clinical practice. Overall satisfaction was 7/10 points (median, range 6–8).

**Conclusions:** This pilot suggests s that an e-module showing appropriate behaviors based on clarified skills and attitudes by role models in lifelike situations may help to engage residents in learning activities and actual practice to master ACPd, thus contributing to quality of care.

#### A534 Limitation of life sustaining therapy in patients died early in ICU

##### A. Estella^1^, M. Gracia^1^, R. Viciana^2^, M. Recuerda^1^, L. Perez Fontaiña^1^

###### ^1^Hospital del SAS de Jerez, Intensive Care Unit, Jerez de la Frontera, Spain; ^2^Hospital del SAS de Jerez, Jerez de la Frontera, Spain

####### **Correspondence:** A. Estella - Hospital del SAS de Jerez, Intensive Care Unit, Jerez de la Frontera, Spain

**Introduction:** Early mortality in ICU may be related to the reason for admission, comorbidity, severity and initial therapy.

**Objectives:** To describe severity and comorbidity relationship with early mortality in patients admitted in ICU.To analyze differences in patients early died in ICU according limitation of life-sustaining therapy (LST) decisions.

**Methods:** Observational study in an ICU of a community hospital. Time of study: 12 months. Inclusion criteria were patients who died within the first 48 hours of ICU admission. The variables analyzed were age, sex, comorbidity Charlson index, APACHE II and lactate levels at ICU admission. Two groups of patients were compared according LST decisions. The data were analyzed using SPSS version 18 for Windows.

**Results:** 65 consecutive patients were analyzed. In 23 % limitation of life sustaining therapy was decided. Table 24 shows the differences between the patients who died with and without LST, lactate levels at ICU admission were 96.1 and 43.2 mg/dl in Not LST group and LST group respectively. There were not observed differences in reason of admission.

**Conclusions:** Predominant clinical profile of patients died early in ICU was elderly patient with high comorbidity, APACHE II and lactate levels at admission. Limitation of life-sustaining therapy decisions were not associated with severity parameters. Comorbidity was higher in this group of patients.

**Table 24 (abstract A534).** Clinical characteristics of patients

	LST (n:15)	Not LST (n:50)
Age, median	72	67.5
Sex (male%/female%)	46,7/53,3	66/34
APACHE II, median	27	26
Origin prior ICU: Emergency; Medical ward Surgical ward Extrahospitalary	20%; 13,3%; 20%; 46,7%	34%; 20%; 8%; 38%.
Mechanical ventilation (%)	86,7	94
Vasoactive drug (%)	73,3	80
Charlson Index <3 3–5 >5	13,3% 13,3% 76,3%	16% 28% 56%

#### A535 This is how we do it, an audit on end of life care decision in critical care unit NMUH

##### B. Tharmalingam^1^, F. Kovari^2^

###### ^1^North Middlesex University Hospital, Anaesthesia/ITU, London, UK; ^2^North Middlesex University Hospital, ICU, London, UK

####### **Correspondence:** B. Tharmalingam - North Middlesex University Hospital, Anaesthesia/ITU, London, UK

Majority of death occur in critical care unit (CCU) after withdrawal/withholding of treatment. Our aim was to analyse the documentation of the complex issues surrounding end of life care (EOL) decision in our CCU. We collected data about our CCU practice of EOL decision and compared with the best practice recommended by Royal college of Anaesthetist in 2012. Our goal was to improve the quality of care of the patients with EOL in our CCU. It's a retrospective audit. All the patients in CCU for whom the decision of withdrawal of life prolonging treatment from 01/04/2014 -31/03/2015 were included in this study. The data was taken from ACUBASE electronic notes and CCU monitoring charts of the relevant cases and analysed using Microsoft EXCEL.

Out of the 217 deaths in CCU 81 (37.3 %) were due to withdrawal of treatment. We had found the excellent documentation regarding family discussion (93.8 %) and formal decision of withdrawal of support (83.9 %). All the patients (100 %) had valid DNACPR form. The documentation of method of withdrawal was 59.2 %, on the other hand the treatment limitation on admission as advanced care plan, documentation of patient discussion about EOL, consideration of spiritual aspect, time of withdrawal, organ donation were documented in 22 %,2 %,12 %,16 % and 30 % of cases respectively. In the method of withdrawal we audited the organ supports and symptom relief medications. Only 73 % of the patients had analgesics and 26 % had sedatives during the process of dying. This is because the prescription was done in drug chart. Feeds/fluids were continued in 78 % of patients.

As a result of this audit we proposed the following actions To formulate a template in ACUBASE with essential elements of EOL which will be filled by the relevant CCU consultant and copy of it will be handed over to the bed side nurse for further care. The time of withdrawal will be documented by the bed side nurse after family gathering and fulfilment of spiritual aspects in the monitoring chart and the doctor who certifies the death will document it on the ACUBASE.

**Fig. 47 (abstract A535).**
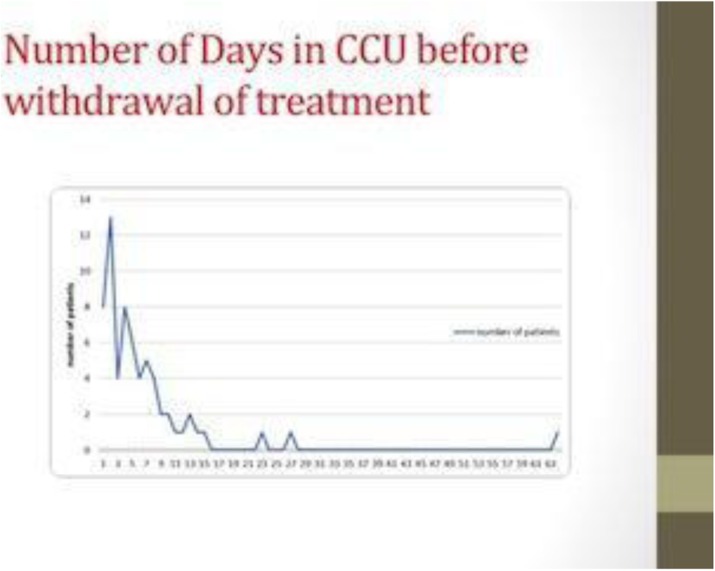
Number of Days in CCU before withdrawal of treatme

### MECHANICAL VENTILATION

#### A536 Variation in definition of prolonged mechanical ventilation: a scoping review

##### L. Rose^1^, M. Mcginlay^2^, R. Amin^3^, K. Burns^4^, B. Connolly^5^, N. Hart^5^, P. Jouvet^6^, S. Katz^7^, D. Leasa^8^, C. Mawdsley^8^, D. Mcauley^9^, M. Schultz^10^, B. Blackwood^9^

###### ^1^University of Toronto, Toronto, Canada; ^2^Royal Victoria Hospital, Belfast, UK; ^3^SickKids Hospital, Toronto, Canada, ^4^St Michael's Hospital, Toronto, Canada; ^5^St Thomas' Hospital, London, UK; ^6^University of Montreal, Montreal, Canada; ^7^Children's Hospital of Eastern Ontario, Ottawa, Canada; ^8^London Health Sciences Centre, London, Canada; ^9^Queen's University, Belfast, UK; ^10^University of Amsterdam, Amsterdam, Netherlands

####### **Correspondence:** L. Rose - University of Toronto, Toronto, Canada

**Introduction:** Consistency of definitional criteria for terminology applied to describe various patient cohorts receiving mechanical ventilation within intensive care unit (ICU) and post-acute care settings is important for understanding prevalence, risk stratification, effectiveness of interventions, and projections for resource allocation. Although a 2005 consensus conference defined prolonged mechanical ventilation (PMV) as mechanical ventilation for ≥21 consecutive days (1), we hypothesized that variable definitions are used when applying this terminology.

**Objectives:** To quantify use of the term PMV and how it is defined within the literature.

**Methods:** Scoping review of studies (all designs except single case study) reporting a study population (adult and paediatric) using the term PMV or synonym. Two authors screened electronic databases (1980–2013) and abstracted data (country; care venue; diagnostic categories; terms used to describe cohort, definitional criteria for these terms, and rationale; and reported outcomes) independently on a standardized form with accuracy verified by a 3rd author.

**Results:** We screened 5331 references, identified 539 references for full text review and excluded 120. Of the 419 studies meeting inclusion criteria, 363 (87 %) were conducted in a single care venue type (most commonly ICU), 30 (7 %) in multiple care venue types, 17 (4 %) were database studies, and 9 (2 %) surveys. Studies represented cohorts from 38 countries most commonly the US (187, 44 %). Most studies (297, 71 %) reported data on a heterogeneous patient cohorts, 66 (16 %) studies reported data on surgical patients only (46/66, 70 % cardiac surgery). Other studies described chronic obstructive pulmonary disease (16, 4 %), trauma (22, 5 %), neuromuscular (17, 4 %), and sepsis (1, 0.2 %) cohorts.

A total of 741 terms were used to refer to the 419 study cohorts. Most commonly used terms were: PMV (253, 60 %), admission to a specialized unit (107, 26 %), and long-term mechanical ventilation (79, 19 %). Some authors (282, 67 %) defined their cohorts based on duration of mechanical ventilation with 154 (55 %) using this as the sole criterion. We identified 37 different durations of ventilation ranging from 5 hours to 1 year with >21 days being the most common (28/282, 7 %). For studies describing a surgical PMV cohort, minimum ventilation duration required for cohort inclusion was ≥24 hours for 20/66 (30 %) studies with ≥15 days as the maximum duration. More than half (237, 57 %) did not provide a reason/rationale for the definitional criteria used, with only 28 (7 %) studies referring to a consensus definition.

**Conclusions:** Substantial variation exists in the terminology and definitional criteria for cohorts of patients receiving mechanical ventilation. Standardization of terminology and definitional criteria is required for study data to be maximally informative.

**References**

1. MacIntyre et al. 2005; 128: 3937–54

**Grant acknowledgement**

Canadian Institutes of Health Research.

#### A537 Introducing the routine use of capnography to a tertiary critical care unit

##### S. Denham, R. Worrall, M. Arshad, P. Isherwood

###### University Hospital Birmingham, Critical Care Medicine, Birmingham, UK

####### **Correspondence:** S. Denham - University Hospital Birmingham, Critical Care Medicine, Birmingham, UK

**Introduction and objectives:** The use of continuous waveform capnography is well established within the theatre environment, and is now considered a standard of care within intensive care by UK national bodies^1 2 3^.

University Hospital Birmingham is a large UK teaching hospital. Historically within this unit the use of capnography has not always been routine on all invasively ventilated patients. This quality improvement project aimed to implement national guidelines in our unit.

**Methods:** To assess the usage, availability and attitudes towards using capnography, a baseline audit of the use of capnography on ventilated patients, during airway interventions and patient transfers was performed.

Concurrently a staff questionnaire was circulated to the multidisciplinary ICU team to complete, in order to assess their knowledge and attitudes towards capnography and promote awareness of national guidelines.

A business case was developed to purchase sufficient modules and consumables to allow for use on every invasively ventilated patient.

Teaching of critical care staff was carried out by the trusts practice development team in conjunction with volunteers from within the critical care team. This covered the system set up and interpretation of the capnography waveform.

Mandatory use of capnography was then introduced into local guidelines and its use was then re-audited.

**Results:** The audit results are shown in Table 25.

**Conclusions:** The routine use of capnography for invasively ventilated patients has risen by 62 %.

The use for airway interventions and patient transfers appears to have remained at the same level or fallen though both these area had much higher baseline rates of use.

This highlights an area for further improvement and the wider dissemination of this work will contribute to further improvement in local practice.

**References**

**1** Association of Anaesthetists of Great Britain & Ireland. The use of Capnography Outside the Operating Theatre. AAGBI Safety Statement. London, 2011. http://www.aagbi.org/sit es/default/files/Capnography (accessed 24/03/16)

**2** Intensive Care Society (UK). Capnograhpy Guidelines 2014. http://www.ics.ac.uk/EasysiteWeb/getresource.axd?AssetID=452&type=full&servicetype=Attachment (accessed 24/03/16)

**3** Resuscitation Council (UK) Resuscitation Guidelines 2015 https://www.resus.org.uk/resuscitation-guidelines/adult-advanced-life-support/ (accessed 24/03/16)

**Table 25 (abstract A537).** Audit Results at Baseline and Post Quali

	Baseline (22/01/14)	Re Audit (05/01/16)
Mechanically Ventilated Patients	24	18
Capnography in Use in Bed space	5/24 (21%)	15/18 (83%)
Capnography used for Airway Interventions	12/13 (92%)	7/11 (64%)
Capnography used for Ventilated Patient Transfer	21/35 (60%)	3/5 (60%)

#### A538 Development of acute respiratory distress syndrome after aspiration in patients with isolated traumatic brain injury

##### A. Khadjibaev^1^, D. Sabirov^2^, A. Rosstalnaya^2^, F. Parpibaev^1^, V. Sharipova^1^

###### ^1^Uzbekistan Research Center of Emergency Medicine, Tashkent, Uzbekistan; ^2^Tashkent Institute of Postgraduate Medical Education, Tashkent, Uzbekistan

####### **Correspondence:** A. Khadjibaev - Uzbekistan Research Center of Emergency Medicine, Tashkent, Uzbekistan

**Introduction:** Disturbances of external respiratory function (ERF) are in a leading position among extracranial complications after severe traumatic brain injury (STBI). According to the opinion of the most specialists, disturbances of ERF belong to the complications resulted from aspiration and damage of mucous tunic of tracheobronchial tree. Aspiration of gastric contents usually occurs owing to consciousness depression accompanied by STBI.

**Objectives:** To determine the development periods and to study the development rate of acute respiratory distress syndrome (ARDS) in isolated STBI complicated with aspiration of gastric contents and blood.

**Methods:** The study was conducted in neurosurgery intensive care unit at Republic Research Center of Emergency Medicine. 33 of 165 patients with STBI admitted between 2013 and 2014 had aspiration. 28 were male and 5 were female. Mean age was 35 ± 3. Glasgow coma score after admission was 5–9. All patients were intubated and mechanically ventilated. Patients were divided into 2 groups according to the etiology of aspiration agent. First group (n = 33) - patients with aspiration of gastric contents or blood. Second group (n = 33) - control group, i.e. with isolated STBI without aspiration. Aspiration was confirmed with fiberoptic bronchoscopy.

**Results:** We conducted analysis of extravascular fluid in the lungs. In 21 patients with aspiration of gastric contents (first group) the amount of extravascular water in lungs (EVWL) 3–6 hours after the trauma was 7.8 ± 3.0 mL/kg (normal value is 3–7 mL/kg), PaO2/FiO2 ratio was 275.0 ± 127.2, after 24 hours EVWL was 7.3 ± 2.9 mL/kg, PaO2/FiO2 ratio was 291.8 ± 94.1. Fifteen patients were with ARDS criteria and their EVWL was 9.6 ± 1.6 mL/kg, PaO2/FiO2 ratio was 218.0 ± 72.9. In 12 patients with aspiration of blood, EVWL 3–6 hours after the trauma was 9.3 ± 2.9 mL/kg, PaO2/FiO2 ratio was 336.2 ± 20.7. Nine of them had ARDS criteria and their EVWL was 10.2 ± 2.2 mL/kg, after 24 hours it was 10.5 ± 4.9 mL/kg. In the second group after 3–6 hours, EVWL was 6.8 ± 1.5 mL/kg, PaO2/FiO2 ratio was 330.5 ± 183.2 and none of the patients had ARDS criteria. After 24 hours EVWL was 6.6 ± 0.8 mL/kg, PaO2/FiO2 ratio was 323.9 ± 122.4, and three of them had ARDS criteria, EVWL was 9.6 ± 1.4 mL/kg, PaO2/FiO2 ratio was 234.0 ± 17.2.

**Conclusions:** Hence in patients with STBI and aspiration of gastric contents we observed significant increase in EVWL (p = 0.05) and decrease in PaO2/FiO2 ratio in the first hours after trauma in comparison with the patients without aspiration. Accumulation of EVWL can be considered as one of ARDS criteria, but only with other signs of damage. ARDS is registered in the first hours after trauma roughly in 50 % of cases in patients with aspiration of both gastric contents and blood.

#### A539 Non-invasive ventilation as an alternative treatment of acute respiratory failure in the critical care unit

##### G.A. Galvez Blanco, C.I. Olvera Guzman, J.S. Aguirre Sánchez, J. Franco Granillo

###### ABC Medical Center, Intensive Care Unit, Mexico City, Mexico

####### **Correspondence:** G.A. Galvez Blanco - BC Medical Center, Intensive Care Unit, Mexico City, Mexico

**Introduction:** Acute respiratory failure (ARF) can be divided in hypoxemic or hypercapnic, depending on the leading modification in blood gas analysis, being the first one the most common. Mechanical Ventilatory Support is one of the treatment mainstays of patients in the Intensive Care Units (ICU), either as management for a primary respiratory imbalance or to protect the airway. Non-invasive mechanical ventilation (NIMV) is defined as any method in which positive pressure is delivered by a face mask or any other interphase without the use of an endotracheal tube.

**Objetives:** It is well established in which pathologies NIMV can be useful, but an inadequate patient selection may cause an increment in morbidity and mortality as well as a delay in endotracheal intubation. While the aim in most studies is to determine which pathologies NIMV will have a better outcome, the main objective in our study was to define whether the blood gas values (pH, paCO_2_ and paO_2_) are the ones that may indicate which patients are better suited for NIMV.

**Methods:** Retrospective observational study of all adult patients admitted to the ICU (mixed ICU) between March 2014 and April 2016 but data presented in this abstract is a preliminary report as we pretend to add more patients in the ongoing months. Patients with DNR order were excluded as well as patients that required endotracheal intubation in the first 2 hours after the beginning of NIMV. Patient's data was analyzed with the purpose of finding cutoff points in blood gas values that could suggest which patients had a better outcome. Patients divided in two groups (survivors or non-survivors) and mean values were compared using Student's *t* test. A *p* value of less than 0.05 was considered to be statistically significant.

**Results:** 52 patients were included and their data analyzed: n = 22 (42.3 %) were female and n = 30 (57.7 %) male, with a mean age of 74 ± 12 (36–94). Of all, n = 15 (29 %) required endotracheal intubation; overall mortality rate was 23.1 % (n = 12). Patients who survived had higher PaO_2_/FiO_2_ ratio than those who did not (*p* = 0.04) and tend towards higher PaCO_2_ levels although *p* values were not statistically significant (*p* = 0.07). ARF cause with the highest mortality was cardiogenic acute pulmonary edema in n = 5 patients (41.7 %).

**Conclusion:** The use of NIMV can be considered as an alternative to endotracheal intubation in hypercapnic patients while the benefit of its use has not been proven in severe hypoxemia (PaO_2_/FiO_2_ ratio less than 100) at least in our group of patients and it actually may lead to a worst outcome.

**References**

1. Moreno Garcia et al. Success/Failure prediction of non-invasivemechanical ventilation in Intensive Care Units. Using multiclassifiers and feature selection methodsMethods Inf Med 2015; 30:54.

2. Davidson AC, et al. British Thoracic Society/ Intensive Care Society Guideline for the ventilatory management of acute hypercapnic respiratory failure in adults. BMJ Open Resp Res 2016;3:e000133.

#### A540 A modified technique of percutaneous dilatational tracheostomy (PCT) in coagulopathic patients having difficult anatomy as assessed by real-time ultrasound

##### S. Gupta, D. Govil, S. Srinivasan, S.J. Patel, J.K. N, A. Gupta, M. Shafi, D.S. Tomar, R. Harne, D.P. Arora, N. Talwar, S. Mazumdar

###### Medanta- The Medicity, Gurgaon, India

####### **Correspondence:** S. Gupta - Medanta- The Medicity, Gurgaon, India

**Introduction:** The recent literature has demonstrated the feasibility of ultrasound in performing PCT in coagulopathic patients with less complications(1) but none of the studies have included patients with coagulopathy and difficult neck anatomy by ultrasound.

**Objectives:** To validate the new technique of performing PCT in a patient with unsatisfactory neck anatomy as defined by a innovative classification under real time ultrasound.

**Methods:** We prospectively conducted this pilot study of 28 PCT in coagulopathic patients. We defined the neck anatomy in three categories depending on the presence of blood vessels in the track of needle even at the lowest visible inter-tracheal space on ultrasound. The unsatisfactory category was defined as presence of multiple vessels in the centre of the field, good category had one blood vessel in the centre of the field, excellent category had clean procedure field with no blood vessels. The technique that we adopted in PCT in unsatisfactory category was liberal instillation of 2 % Xylocaine in the skin and subcutaneous tissue between the vessels and creating a potential space for the needle. We also avoided putting any predetermined incision on the skin during the procedure and followed an entire seldinger technique by dilating the skin and subcutaneous tissue using the 5Fr tracheal dilator provided in Portex® ULTRAperc®. The entire procedure was done under real time ultrasound guidance. Blood loss was estimated by the number of gauze pieces soaked with blood with one gauze piece soaking 5 ml.

**Results:** The average local anesthesia requirement was around 10 ml for instillation. The INR ranged from 2.1 to 2.9 and the platelet count ranged from 20,000 to 75,000. None of the patients required any blood product transfusion. The maximum blood loss was 15 ml and none of the patients required abandoning of the procedure.

**Conclusions:** This modification of the classical technique of PCT in patients having unfavorable neck anatomy resulted in increased safety with minimal blood loss. Ultrasound guidance is a very useful modality to define such unfavorable neck anatomy which are non amenable by just palpation of neck. Correction of coagulopathy may not be mandatory if real-time ultrasound is used during this modified technique of PCT.

**References**

1. Rajajee V, Williamson AC, West TB. Impact of real-time ultrasound guidance on complications of percutaneous dilatational tracheostomy: a propensity score analysis. Crit Care. 2015;19(1):198

**Grant acknowledgement**

None.

**Fig. 48 (abstract A540).**
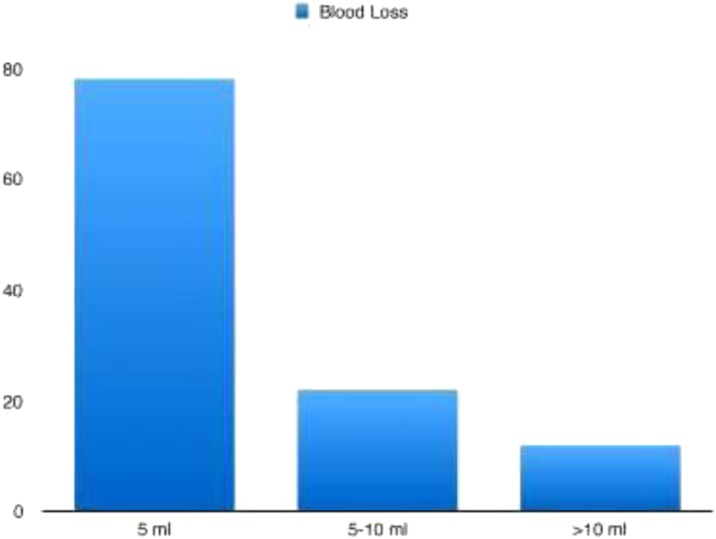
Blood loss

#### A541 The usefulness of delta neutrophil index for predicting superimposed pneumonia in patients with acute decompensated heart failure at the emergency department

##### Y.S. Cha^1^, S.J. Lee^2^

###### ^1^Yonsei University, Wonju College of Medicine, Emergency Medicine, Wonju, Republic of Korea; ^2^Yonsei University, Wonju College of Medicine. Department of Internal Medicine, Wonju, Republic of Korea

####### **Correspondence:** Y.S. Cha - Yonsei University, Wonju College of Medicine, Emergency Medicine, Wonju, Republic of Korea

**Introduction:** Respiratory infections have long been recognized as precipitators of exacerbations and a concomitant pneumonia has been reported in 7-10 % of acute decompensated heart failure (ADHF) patients. Detecting patients, who present with an ADHF and signs of superimposed pneumonia, is difficult because of the non-specific nature of physical examination or chest x-ray abnormalities in the setting of cardiogenic pulmonary edema. Nahm et al. developed neutrophil delta neutrophil index (DNI) as a new indicator for immature neutrophils and DNI expression is induced by bacterial infection.

**Objectives:** No information is available on the clinical usefulness of DNI with respect to the diagnosis of superimposed pneumonia in patients with ADHF. Therefore, we evaluated the presence of difference of initial DNI according to presence of pneumonia in patients with ADHF, the predictive value of DNI and the usefulness of DNI in combination with C-reactive protein (CRP), which is the commonly used for predicting inflammation and infection in the ED, for diagnosis of superimposed pneumonia in patients presenting with ADHF at the ED.

**Methods:** This is a retrospective and observational study of consecutive patients more than 18 years of age who were diagnosed with an ADHF in the ED over a 7 month period. Patients were categorized by the ADHF group and the superimposed pneumonia group. Serum C-reactive protein (CRP), DNI, and β-natriuretic peptide (BNP) were measured upon ED arrival.

**Results:** The superimposed pneumonia group included 78 patients (38.4 %). Median initial DNI and CRP were significantly higher in the superimposed pneumonia group (0 % vs. 1.2 %, p < 0.001 and 0.52 mg/dL vs. 7.33 mg/dL, p < 0.001), respectively. In the receiver operation characteristic (ROC) curve for initial DNI and serum CRP for differentiating superimposed pneumonia in ADHF patients, area under curve (AUC) of DNI [0.800 (95 % confidence interval 0.737-0.853)] and CRP [0.834 (95 % confidence interval 0.774-0.883)] were all good and there was no a statistical difference of AUC between two inflammatory markers (p = 0.367). The AUC of the initial DNI and CRP combination [0.862 (95 % CI 0.806-0.907)] was not significantly higher than the AUC for initial CRP alone [0.834 (95 % CI 0.774-.883)] (p = 0.099).

**Conclusions:** Initial DNI was significantly higher in the superimposed pneumonia group than in the ADHF group and the prediction ability for ADHF with superimposed pneumonia of initial DNI in the ED was as good as those of serum CRP.

**References**

1. Khand AU, Gemmell I, Rankin AC, et al. Clinical events leading to the progression of heart failure: insights from a national database of hospital discharges. Eur Heart J 2001;22:153–64.

2. Nahm CH, Choi JW, Lee J. Delta neutrophil index in automated immature granulocyte counts for assessing disease severity of patients with sepsis. *Ann Clin Lab Sci* 2008;**38**:241–6.

#### A542 Survival benefit of prone ventilation and feasibility of prolonged (>20 hours) prone positioning in severe H1N1 ARDS: experience from India. (A tertiary care teaching hospital and ECMO center)

##### N. Tyagi, R. K. Rajput, S. Taneja, V. K. Singh, S.C. Sharma, S. Mittal, B.K. Rao

###### Sir Ganga Ram Hospital, Delhi, India

####### **Correspondence:** N. Tyagi - Sir Ganga Ram Hospital, Delhi, India

**Introduction:** Gas exchange is more severely impaired in H1N1-ARDS often necessitating rescue therapies.Those with severe ARDS if can´t be offered ECMO have Prone ventilation as only option. Prone ventilation^1^ hasn't been studied in details as far as H1N1-ARDS is concerned.

**Objectives:** We examined whether early application of prone positioning would improve survival among patients with severe H1N1 ARDS.

We also tried to assess feasibility of prolonged proning (>20 Hours)

**Method:** A single center observational study of 61 adult patients, with PCR confirmed H1N1 infection, admitted between December 1, 2014 & June 31, 2015.

Patients with severe ARDS as per Berlin definition, after 6–12 hours of mechanical ventilation were assigned to undergo prone-positioning sessions of at least 20 hours or stay supine as per family/treating physician´s decision.

Patients who ended receiving ECMO were excluded.

Those who had P/F ratio ≤1.0 on FiO_2_ ≥ 0.8 and PEEP ≥ 10 cmH_2_O at end of 20 hrs were kept further proned in sets of 4 hrs till they achieved this goal and then turned supine.

The primary outcome was 28 days all cause mortality after inclusion.

Respiratory characteristics,effect of first prone ventilation on the P:F ratio and PEEP requirement,complications and clinical outcome were collected.

**Results:** 31 patients had severe ARDS after 6–12 hours of mechanical ventilation.

28 days mortality rate in these patients was 51.61 %(n = 16).

Mean P:F ratio was 0.73 + 0.14.

All patients had a Compliance of < 20 ml/cmH_2_O (Mean cpl 16.3 + 1.52 ml/cmH_2_O).

17 patients were turned to prone position within 48 hours of mechanical ventilation.

13 patients remained in supine position,1 patient with 26 week pregnancy received HFOV.

Mortality rates in prone & supine group were 47.05 % (n = 8) & 61.53 % (n = 8) respectively. (p = 0.431)

12 patients showed a significant increase in P:F (≥30 %) but only 8 showed a concomitant decrease in PEEP (≥2cmH2O) at the end of first prone session.

Target end points of proning got achieved in 11 patients.

Mean duration of prone session was 21.97 + 6.17 hours.

Average number of prone sessions was 2.5 among survivors & 4.5 among non-survivors.

7 patients received session of prolonged prone positioning (>24 hrs) out of which 4 (57.14 %) survived.

Complications: 10 patients needed to stop enteral feeding transiently during prone session due to increased residual gastric aspirate.

9 patients had Grade 1 pressure sores on the face.

1 patient in prolonged proning had sudden cardiac arrest during the prone session.

**Conclusions:** Easy feasibility & rapid response assessment make prone ventilation an ideal choice for the initial treatment of patients with severe H1N1-ARDS.

For who can not be put on ECMO for various reasons, prolonged proning is an option as rescue therapy and should be evaluated by RCTs.

**References**

1.Beitler JR, Shaefi S, Montesi SB, et al. prone positioning reduces mortality from ARDS in the low tidal volume era: a meta-analysis.ICM 2014; 40:332

**Grant acknowledgement**

None

#### A543 A comparison of the neuromuscular blockade induced by original brand-name (NIMBEX®) and generic (CISATREX®) cisatracurium in mechanically ventilated medical critically ill patients

##### J. Ayachi^1^, N. Fraj^1^, S. Romdhani^1^, A. Khedher^1^, K. Meddeb^1^, N. Sma^1^, A. Azouzi^1^, R. Bouneb^1^, I. Chouchene^1^, M. El Ghardallou^2^, M. Boussarsar^1,3^

###### ^1^Farhat Hached University Hospital, Medical Intensive Care Unit, Sousse, Tunisia; ^2^Ibn Al Jazzar Faculty of Medicine, University of Sousse, Department of Community Medicine, Sousse, Tunisia; ^3^Research Laboratory N° LR14ES05, Interactions of the Cardiopulmonary System, Ibn Al Jazzar Faculty of Medicine. University of Sousse, Sousse, Tunisia

####### **Correspondence:** J. Ayachi - Farhat Hached University Hospital, Medical Intensive Care Unit, Sousse, Tunisia

**Introduction:** Use of generic drugs, which are bioequivalent to brand-name drugs, is now a common practice. However, there is still concern among patients and physicians that brand-name drugs are more efficient than generic drugs (1).

**Objectives:** To compare efficiency and tolerance of two marketed forms: original brand-name (NIMBEX®) and generic (CISATREX®) cisatracurium-induced paralysis in hypoxemic ventilated patients.

**Material and methods:** A cross-over, randomized, double-blind physiological trial. Was compared, neuromuscular blockade induced respectively by the original brand-name and generic continuous cisatracurium infusion during two successive periods, separated by a one hour washout period. Neuromuscular function was monitored by visual estimation of the contractions (number and height) of the thumb in response to a calibrated train-of-four (TOF) stimulation every 5 minutes. A continuous infusion of cisatracurium was started at 0.06 mg^−1^kg^−1^h^−1^. The cisatracurium infusion rate was then increased by increments of 0.03 mg^−1^kg^−1^h^−1^ every thirty minutes to reach and sustain a TOF of 2/4. Were measured, the delay of paralysis defined by the time needed from infusion onset to reach a TOF of 2/4 and respective recovery times defined by the time needed to reach a TOF of 4/4 after stopping cisatracurium infusion. Under optimized sedation and ventilation, patients admitted for hypoxemic acute respiratory failure with a P/F < 200 and requiring paralysis were enrolled. Patient's characteristics and SAPS II score were assessed at admission. Ramsay sedation scale, respiratory and hemodynamic parameters, P/F ratio were evaluated at baseline. Tolerance was assessed by significant variations (>30 %) of heart rate and/or systolic blood pressure. Number needed to demonstrate a significant difference between the delays to reach a TOF of 2/4 in the two studied cisatracurium forms was estimated at 22 patients.

**Fig. 49 (abstract A543).**
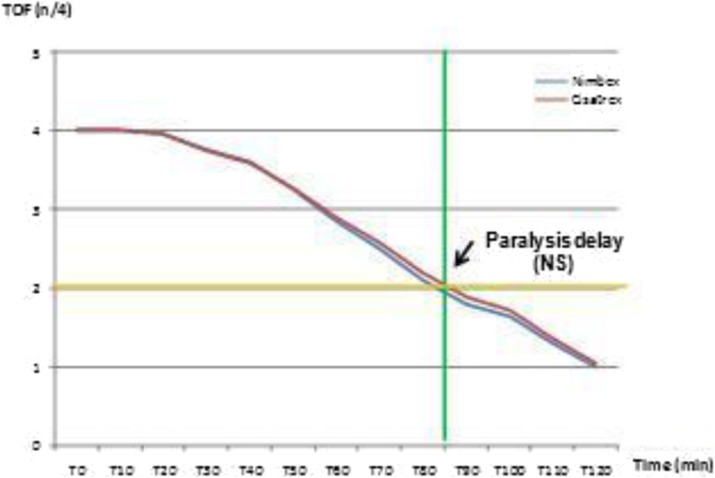
ᅟ

**Results:** Twenty two patients were included. Median age was 56[41, 73] years. 14(61,9 %) were male. 17(77.3 %) had ARDS and 5(22.7) had status asthmaticus. Median SAPS II at admission, 34[25, 43]. 11(52,3 %) patients had a PaO_2_/FIO_2_ ratio < 100. Median paralysis delay was respectively 70[44, 96]min in NIMBEX® and 70[43, 97]min in CISATREX®; (p = 0.579). Figure. 49 displays respective TOF kinetics in the two forms of cisatracurium. The median recovery time from paralysis was respectively 60[40, 80]min in NIMBEX® and 60[42, 78]min in CISATREX® (p = 0.924). There was no significant hemodynamic variations neither in NIMBEX® nor in CISATREX®.

**Conclusion:** The present study revealed no significant difference in efficacy as in tolerance between original brand-name NIMBEX® and generic (CISATREX®) of cisatracurium.

**References**

1 Shrank W, Cox E, Fischer MA, Mehta J,et al. Patient perceptions of generic medications. Health *Aff (Millwood).2009 Mar-Apr; 28(2):546–56*

**Grant acknowledgement**

MédiS Laboratories, Tunisia.

#### A544 Titrating sedation according to sedation scores in intensive care - a patient safety audit

##### R. Jennings, E. Walter

###### Royal Surrey County Hospital, Intensive Care, Guildford, UK

####### **Correspondence:** R. Jennings ^_^ Royal Surrey County Hospital, Intensive Care, Guildford, UK

**Introduction:** The Richmond Agitation-Sedation Score (RASS) records the level of sedation of a patient from −5 to +4, and has been used in the Royal Surrey County Hospital Intensive Care Unit since November 2012. Maintaining optimum levels of sedation (RASS −2 to 0) improves clinical outcomes (1).

**Objectives:** The objectives of this audit were to assess the median level of sedation in ventilated patients, record any variation in score at different times of day or night and measure the frequency of recorded score. This audit was then repeated to measure any improvement in practice.

**Methods:** All patients sedated and ventilated during July 2015 were audited and then re-audited in November 2015. Patients were excluded if it was felt that reducing the level of sedation was inappropriate.

**Results:** 300 scores were obtained during July 2015 and 672 in November 2015. The median sedation score was −2 in July and −3 in November. 41.5 % were too heavily sedated at −3 or below in July and 53.3 % in November.

The majority of scores were recorded frequently with 84 % of patients in July having a score recorded at least every 2 hours and 91 % in November.

**Conclusions:** Sedation scores are being recorded frequently, with few long gaps for either month. Sedation protocols should be used in order to target the lowest possible levels of sedation. There should be discussion around whether targets should be different at night compared with daytime.

There has been no improvement in sedation practice in November compared with July 2015. Fewer than half (**45.5 %**) of patients were sedated to optimum levels in July and this fell to **38.2 %** in November. There may be merit in maintaining an infusion of the opioid, whilst reducing or stopping the hypnotic, rather than stopping both completely. Maintaining staff awareness of the benefits of optimising sedation is prudent.

**References**

1. Barr JL, Fraser GL, Puntillo K, Ely EW. Et al. Clinical practice guidelines for the management of pain, agitation, and delirium in adult patients in the intensive care unit. *Critical Care Medicine* 2013; **41**(1):263–306.

**Grant acknowledgement**

Nil grant obtained.

#### A545 A case series of severe ARDS from *Legionella* pneumonia successfully treated with extracorporeal membrane oxygenation

##### J.M. Ribeiro, I. Moniz, R. Marçal, A.C. Santos, C. Candeias, Z. Costa e Silva

###### University Hospital of Santa Maria, CHLN, Intensive Care Department, Lisbon, Portugal

####### **Correspondence:** J.M. Ribeiro - University Hospital of Santa Maria, CHLN, Intensive Care Department, Lisbon, Portugal

**Introduction:** The management of severe *Legionella* pneumonia can include extracorporeal membrane oxygenation (ECMO) to rescue those patients which develop refractory hypoxemia or extreme respiratory acidosis. However, some physicians remain skeptical about this approach, considering that evidence is very weak and only derived from case series from single ECMO centers. We believe that reporting experience from diverse centers is important to expand the knowledge and the predictors of benefit from ECMO support in patients with *Legionella* pneumonia.

**Objectives:** Clinical, physiological, functional and biomechanical characterization of a population with severe *Legionella* pneumonia treated with extracorporeal life support in a ELSO recognized ECMO centre.

**Methods:** Protocol-driven prospective registration and analysis of data from a population of 9 patients diagnosed with severe acute respiratory distress syndrome (ARDS) secondary to *Legionella* infection, with additional respiratory physiology evaluation and characterization during the ECMO run treatment.

**Results:** From 2013 to 2015, nine patients with *Legionella* pneumonia and severe ARDS have failed conventional mechanical ventilation and were treated with extracorporeal oxygenation (*Quadrox HLS or PLS Oxygenator System* from Maquet). Median age was 46.6 ± 12.3 and all patients were male. ECMO criteria admission were hypoxemia (median pre-ECMO PaO2:FiO_2_ of 73.5 ± 22.9 mmHg) associated with respiratory acidosis (median pre-ECMO PaCO_2_ of 69,6 ± 12,5 mmHg and pH of 7.22 ± 0,08). Murray score was 3.17 ± 0,16 and SAPS II was 43.7 ± 9.8. Symptoms to cannulation was 4.4 ± 2.2 days, which represented a very early strategy to ECMO implementation. Initial static compliance was 25.9 ± 7.6 ml/cmH_2_O, did not changed significantly by day four (27.7 ± 13,4), and improved to 38.1 ± 13.4 by day seven. Six patients were smokers, six had acute kidney injury AKIN 3 and three had circulatory shock. During ECMO run, all patients were additionally conventionally ventilated with a lung rest strategy (Vt 2.0 ml/kg, driving pressure below 15 cmH_2_O and PEEP of 10 to 14 mmHg). Seven patients were treated with azithromycin and two patients with levofloxacin. We did not prescribed corticosteroids in any patient. All patients survived ECMO run (median time of 9.8 ± 4.5 days) and hospital discharge.

**Conclusions:** We showed excellent results derived from a strategy of extracorporeal oxygenation in patients with the most severe forms of *Legionella* pneumonia. We believe that early referral and early implementation of a protocol-driven ECMO technique, with better understanding of its physiological and management principles, might improve the outcomes of patients with severe *Legionella* pneumonia.

**Grant acknowledgement**

No grants.

#### A546 Percutaneus tracheostomy complications in Hospital Juarez de Mexico intensive care unit

##### S.E. Zamora Gomez, O.R. Perez Nieto, J.A. Castanon Gonzalez, A.I. Vasquez Cuellar

###### Hospital Juarez de México, ICU, Mexico City, Mexico

####### **Correspondence:** S.E. Zamora Gomez - Hospital Juarez de México, ICU, Mexico City, Mexico

**Introduction:** Percutaneous Tracheostomy is a procedure commonly performed in the intensive care unit^1^, being considered as first choice in patients in critical condition due to the lower rate of complications associated with this procedure^2^, reports of perioperative complications ranging from 2 to 6 %^3^ such as bleeding, death, false passage, extubating, pneumothorax, emphysema, hypertension and inability to perform the technique; Postoperative complications were reported from 9 to 17 %^3^, including bleeding, displaced tube, pneumothorax, infection and death. In our intensive care unit we have one year experience of performing percutaneous tracheostomy, with very low rate of complications so far.

**Objectives:** To determine the incidence of complications related to percutaneous tracheostomy in critical ill patients in our intensive care unit.

**Methods:** A retrospective study was conducted in our intensive care unit during March of 2015 to February of 2016, counting 23 patients who underwent percutaneous tracheostomy for any indication, and the complications observed in each procedure were recorded in a data collection sheet.

**Results:** We obtained a population of 23 patients, 11 females and 12 males, all diagnoses of these patients were divided into groups, including 9 patients with lung disease (39.2 %), 7 patients with neurological disease (30.4 %), and 7 patients with abdominal disease (30.4 %), with an average of 8.47 days of mechanical ventilation, and an average of 15.4 minutes duration of the percutaneous tracheostomy, in which 2 episodes of extubating without clinical repercussion as perioperative complications were reported (8.9 %), and a tube displacement which required endotracheal intubation as a postoperative complication (2.3 %).

**Conclusions:** We observed a low incidence of perioperative complications in our patient population, as well as a very low incidence of postoperative complications compared with that reported in the reviewed articles, no serious complications in any patient who perform percutaneous tracheostomy were observed. We strongly recommend percutaneous tracheostomy as a safe method to the bedside of most patients in critical condition, regardless of their disease.

**References**

1. Griffiths J, Barber VS, Morgan L, Young JD. Systematic review and meta-analysis of studies of the timing of tracheostomy in adult patients undergoing artificial ventilation. BMJ 2005.

2. Freeman BD, Isabella K, Lin N, Buchman TG. A meta-analysis of prospective trials comparing percutaneous and surgical tracheostomy in critically ill patients. Chest. 2000;118:1412–8.

3. Massick et al. Bedside tracheostomy in the intensive care unit: A prospective randomized trial comparing open surgical tracheotomy UIT Endoscopically guided percutaneous ditational tracheotomy. Laryngoscope 2001; 111: 494–500.

### BIOMARKERS FOR AKI

#### A547 Three-year mortality in 30-day survivors of critical care with acute kidney injury - data from prospective Finnaki study

##### H. Mildh^1^, V. Pettilä^1,2^, A.-M. Korhonen^1^, S. Karlsson^3^, T. Ala-Kokko^4^, M. Reinikainen^5^, S.T. Vaara^1^, The FINNAKI Study Group

###### ^1^University of Helsinki and Helsinki University Hospital, Helsinki, Finland; ^2^Bern University Hospital and University of Bern, Bern, Switzerland; ^3^Tampere University Hospital, Tampere, Finland; ^4^Oulu University Hospital, Medical Research Center Oulu, Oulu, Finland, ^5^North Karelia Central Hospital, Joensuu, Finland

####### **Correspondence:** H. Mildh - University of Helsinki and Helsinki University Hospital, Helsinki, Finland

**Introduction:** Acute Kidney Injury (AKI) is a frequently encountered syndrome in the critically ill. While the role of AKI in increasing short-term mortality is evident, results regarding its role in long-term mortality are conflicting. Advanced age and pre-existing co-morbidities are known risk factors for AKI and also the main determinants for the long term-mortality of initial survivors of intensive care (1).

**Objectives:** To determine whether AKI is an independent risk factor for increased three-year mortality among 30-day survivors of critical care.

**Methods:** This prospective study enrolled 2901 patients from 17 Finnish intensive care units (ICU) in 2011–2012. In this analysis, we included 30-day survivors of the cohort. We compared the crude three-year mortality of AKI and non-AKI patients and adjusted for confounders using Cox proportional hazard model. We performed sensitivity analyses by excluding patients with 1) chronic kidney disease (CKD), 2) stage 1 AKI, or 3) CKD, stage 1 AKI only, or an estimated pre-admission creatinine.

**Results:** The study included 2336 30-day survivors of intensive care. Of these, 808 (34.6 %, 95 % CI 32.7 %-36.5 %) developed AKI according to the Kidney Disease: Improving Global Outcomes -criteria. The crude mortality at three years was 190/808 (23.5 %, 20.6 %-26.4 %) in the AKI patients and 289/1528 (18.9 %, 17.0 %-20.9 %) in the non-AKI patients (*p* = 0.010). In adjusted Cox model, AKI was not an independent risk factor for three-year mortality (hazard ratio (HR) 1.05; CI 95 % 0.86-1.27, *p* = 0.644), whereas age (HR 1.03; 1.03-1.04), chronic obstructive pulmonary disease (HR 1.63; 1.26-2.10), chronic kidney disease (HR 1.53; 1.23-2.07), chronic liver failure (2.90; 1.89-4.44), malignancy (HR 3.18; 2.18-4.62), rheumatoid diseases (HR 1.87; 1.35-2.60) and poor premorbid functional performance were. Pre-existing arteriosclerosis, diabetes, hypertension, systolic heart failure, thrombophilia and characteristics of the ICU admission and treatment (operative admission, higher SAPS II score without age and renal points, use of vasoactives and severe sepsis) did not independently associate with an increased hazard for three-year mortality. In the sensitivity analyses, AKI was not associated with an increased hazard for three-year mortality.

**Conclusions:** Our findings imply that increased long-term mortality among patients with AKI who survive critical illness is not related to AKI *per se*, but rather to increased age and pre-existing comorbidities.

**References**

1. Garland A, Olafson K, Ramsey CD, Yogendran M, Fransoo F; Distinct determinants of long-term and short-term survival in critical illness; Intensive Care Med 2014, 40, 1097–1105

**Grant acknowledgement**

This study was supported by the Sigrid Juselius Foundation, Päivikki and Sakari Sohlberg Foundation, and Institutional Grants from the Helsinki University Hospital.

#### A548 Beta- 2 microglobulin as a key player in cognitive decline related to acute kidney injury (AKI)

##### M. Zaleska-Kociecka^1^, M. Grabowski^2^, M. Dąbrowski^3^, S. Wozniak^4^, K. Piotrowska^5^, M. Banaszewski^1^, J. Imiela^6^, J. Stepinska^1^

###### ^1^Institute of Cardiology, Cardiac Intensive Care Clinic, Warsaw, Poland; ^2^Institute of Cardiology, Department of Valvular Heart Diseases, Warsaw, Poland; ^3^Institute of Cardiology, Department of Interventional Cardiology and Angiology, Warsaw, Poland,; ^4^Institute of Cardiology, Cardiac Surgery and Transplantology Clinic, Warsaw, Poland; ^5^Kozminski University, Department of Quantitative Methods & Information Technology, Warsaw, Poland; ^6^Miedzyleski Hospital, 1st Department of Internal Medicine, Warsaw, Poland

####### **Correspondence:** M. Zaleska-Kociecka - Institute of Cardiology, Cardiac Intensive Care Clinic, Warsaw, Poland

**Introduction:** After years of negligence, a kidney-brain crosstalk has become an issue [1].The link between cognitive impairment and chronic kidney disease is widely acknowledged. Less is known about the influence of AKI. Recently, an increase of serum beta-2 microglobulin (B2M), an uremic toxin, was proved to be associated with cognitive decline (CD) in mice [2].

**Objectives:** To investigate relation between acute and chronic changes of kidney biomarkers: B2M, creatinine, cystatin C, NGAL and cognition in patients (pts) undergoing aortic valve replacement (AVR)

**Methods:** Eighty over 70-year-old consecutive pts with severe aortic stenosis undergoing surgical AVR (SAVR, n = 40) or trascatheter AVR (TAVR, n = 40) were enrolled in prospective, observational single-centre study. All biomarkers were tested before AVR and 6, 12, 18, 24, 36 and 48 h afterwards, at discharge and at 6-month follow-up. Mini Mental State Examination (MMSE) was performed before AVR, at discharge and follow-up.

**Results:** Higher serum B2M levels were consequently correlated with lower MMSE score at all time-points: at baseline (0.305; p = 0.01), at hospital discharge (0.296; p = 0.013) and at 6-month follow-up (0.496; p < 0.001). Correlations of other biomarkers were inconsistent. There was also correlation between B2M increase from baseline to discharge and CD, (drop in MMSE) over hospital stay (309; p = 0.009). This was not observed within other biomarkers.

The risk of in-hospital CD was significantly higher in pts with B2M increase over the median change of 0.42 mg/dl (OR 2.66;95 % CI 1.02-6.94; p = 0.043). None such relation was noted with other biomarkers.

Univariate log. regression analysis indicated trend for association between in-hospital B2M increase and CD (OR 2.02; 95 % CI 0.994-4.09; p = 0.052). Significant risk factors were baseline MMSE (OR 1.32; 95 % CI 1.09-1.6; p = 0.004) and max. postprocedural CRP (1.06; 95 % CI 1.0-1.13; p = 0.035). In multivariate log.regression analysis adjusted for demographic variables, typical cardiovascular and periprocedural risk factors in-hospital increase of B2M was third the most associated with CD (OR 2.55; 95 % CI 0.878-7.38; p = 0.085). It followed higher baseline MMSE (OR 0.703; 95 % CI 0.533-0.927; p = 0.013) and diabetes mellitus (OR 4.32; 95 % CI 0.92-20.4; p = 0.065).

We did not find relation between changes of B2M and cognition over the follow-up. The connection could have been obscured by multimorbidities burden in this population.

**Conclusions:** Our study is the first in human to report relation between acute changes of uremic toxin and cognition with B2M as a key player. By analogy to cardio-renal syndrome, four types of neuro-renal syndrome should become an issue.

**References**

1. Lu, R., Kiernan et al. Kidney-brain crosstalk in the acute and chronic setting. *Nat Rev Nephrol.***11**, 707–719 (2015).

2. Smith, L.K. et al. Villeda. β2-microglobulin is a systemic pro-aging factor that impairs cognitive function and neurogenesis. *Nat Med.* 21, 932–937 (2015).

#### A549 Acute kidney injury in cardiac intensive care unit: incidence and risk factors

##### A. González Pérez, P. Florez Ordoñez, A. Giribet, M.A. Alonso Cuervo, R. Alonso Cuervo, M.A. Rodriguez Esteban, L. Iglesias Fraile, C. Ponte Mittelbrum, G. Muñiz Albaiceta

###### Hospital Universitario Central de Asturias, Oviedo, Spain

####### **Correspondence:** A. González Pérez - Hospital Universitario Central de Asturias, Oviedo, Spain

**Introduction:** Acute kidney injury (AKI) is a serious complication in patients admitted to the Intensive Care Unit (ICU) which increases morbidity and mortality. Controversy exist about the associated risk factors.

**Objectives:** The aim of this study was to analyze the incidence of AKI in cardiac ICU and related risk factors.

**Methods:** Retrospective observational study of patients admitted to a Cardiac ICU who developed AKI between 2004 and 2014. AKI was defined as impaired renal function with elevation to twice baseline serum creatinine or need renal replacement therapy (RRT). Demographic data and clinical variables to determine AKI incidence were collected. In patients undergoing cardiac surgery, risk factors for AKI development and history of intraoperative blood products transfusion were analyzed. Values expressed as mean + − SD or %.Significant variables in the univariate analysis were entered into a multivariate logistic regression model to calculate the Odds Ratio (OR) with confidence interval of 95 %.

**Results:** We analyzed a series of 8146 patients, mostly males (64.8 %), age 66.92 +/− 12.60 years. Patients origin: 6171 (75.8 %) underwent cardiac surgery of valve replacement and / or coronary bypass graft, 507 (6.2 %) of hospitalization floor, 478 (5.9 %) of Emergency Department, 354 (4.3 %) of Coronary Care Unit, 307 (3.8 %) from other Hospitals, 149 (1.8 %) other ICU, 123 (1.5 %) after cardiac catheterization, and 57 (0.7 %) postoperative Care Unit. AKI developed in 763 patients (9.5 %).Two groups were compared. Group 0: without AKI and group 1 with AKI development during ICU stay. Patients in group 1 were older: 66.71 +/− 12.72 vs 68.88 +/− 11.22 years; p < 0.001; mostly males: 68.7 %; the average ICU stay was higher: 6.09 +/− 12.89 vs 16.44 +/− 21.21 days; p < 0.001. 382 patients in AKI group died (50.2 %) and 20.3 % were treated with RRT. The subgroup of patients undergoing cardiac surgery who developed AKI had a higher baseline creatinine, urea and LOGISTIC EUROSCORE: 1.05 +/− 0.54 vs 1.39 +/− 0.60 p < 0.001; 49.90 +/− 22.25 vs 69.18 +/− 32.19 p < 0.001; 6.06 +/− 4.42 vs 9.76 +/− 7.64 p < 0.001, respectively. They also received more intraoperative red blood cells 1.62 +/− 2.32 vs 2.88 +/− 2.26 p < 0.001, plasma 1.25+/−1.80 vs 2.59+/−2.19 p < 0.001 and platelets transfusion 0.40+/−0.62 vs 0.81+/−0.74 p < 0.001. In the multivalent analysis intraoperative red blood cells, plasma and platelets transfusion proved to be independents factors for the development of AKI in ICU: OR 1.147 CI (1.070 to 1.230) p < 0.001; 1.365 CI (1.280 to 1.445) p < 0.001; 2.054 CI (1.644 to 2.515) p < 0.001, respectively.

**Conclusions:** age > 70 years old, baseline creatinine, baseline urea, preoperative hemoglobin were risk factors for development of AKI in our study. Intraoperative transfusion of packed red blood cells, plasma and platelets were independents factors of development of AKI after cardiac surgery.

#### A550 Incidence of acute kidney injury in critically ill patients varies with the definition used

##### J. Koeze, F. Keus, W. Dieperink, I.C.C. van der Horst, M. van Meurs, J.G. Zijlstra

###### UMCG, Department of Critical Care, Groningen, Netherlands

####### **Correspondence:** J. Koeze - UMCG, Department of Critical Care, Groningen, Netherlands

**Introduction:** Acute kidney injury (AKI) is a serious complication of critical illness with attributed morbidity and mortality both at short-term and long-term. (1, 2) The incidence of AKI in critically ill patients varies substantially with the population evaluated and the definitions used. (1, 3, 4) We aimed to assess how the timing and the incidence of AKI varies with AKI definitions, both with and without using urine output criteria.

**Methods:** We included all patients admitted to our 42 bed intensive care unit (ICU) from 1^st^ January 2014 until the 11^th^ of June 2014. Incidences of AKI and timing were estimated using RIFLE, AKIN and KDIGO definitions.

Results. A total of 1376 critically ill patients were included in our cohort. AKI incidence proportions varied from 15 to 21 % using RIFLE, AKIN and KDIGO serum creatinine criteria. AKI incidence proportions varied from 35 to 38 % when urine output criteria were added to the RIFLE, AKIN and KDIGO definitions. AKI was detected after a median of 24 hours (IQR 24–48 hours) using serum creatinine criteria and AKI was detected after a median of 13 hours (IQR 7–23 hours) using urine output criteria.

Conclusion. Incidences of AKI are influenced by which definition is used. Addition of urine output criteria to serum creatinine criteria may result in a doubling of AKI incidences and an eleven hours time gain in a critically ill population.

**References**

1) Gammelager H, Christiansen CF, Johansen MB, Tonnesen E, Jespersen B, Sorensen HT. One-year mortality among danish intensive care patients with acute kidney injury: A cohort study. Crit Care. 2012;16(4):R124.

2) Gammelager H, Christiansen CF, Johansen MB, Tonnesen E, Jespersen B, Sorensen HT. Five-year risk of end-stage renal disease among intensive care patients surviving dialysis-requiring acute kidney injury: A nationwide cohort study. Crit Care. 2013;17(4):R145.

3) Fujii T, Uchino S, Takinami M, Bellomo R. Validation of the kidney disease improving global outcomes criteria for AKI and comparison of three criteria in hospitalized patients. Clin J Am Soc Nephrol. 2014;9(5):848–854.

4) Wlodzimirow KA, Abu-Hanna A, Slabbekoorn M, Chamuleau RA, Schultz MJ, Bouman CS. A comparison of RIFLE with and without urine output criteria for acute kidney injury in critically ill patients. Crit Care. 2012;16(5):R200.

#### A551 Acute kidney injury (AKI) requiring continuous renal replacement therapies (CRRT) in a cardiothoracic intensive care unit: incidence, indications and results

##### S. Roberts, C. Hernandez Caballero, G. Isgro, D. Hall

###### Royal Brompton and Harefield NHS Foundation Trust, London, UK

####### **Correspondence:** C. Hernandez Caballero - Royal Brompton and Harefield NHS Foundation Trust, London, UK

**Introduction: BACKGROUND.** The incidence of acute kidney injury (AKI) occurring in patients admitted to intensive care units (ICUs) ranges from 30 % to 60 %. It has shown to be an important predictor of morbidity and mortality in the critically ill and continuous renal replacement therapies represent a cornerstone in its management. The Kidney Disease Improving Global Outcomes Guideline (KDIGO Guideline) on renal support for AKI suggests initiating CRRT when life-threatening changes in fluid, electrolyte and acid–base balance exist.

**Objectives:** To determine the incidence and prognosis of AKI requiring CRRT in a tertiary cardiothoracic centre.

**Fig. 50 (abstract A551).**
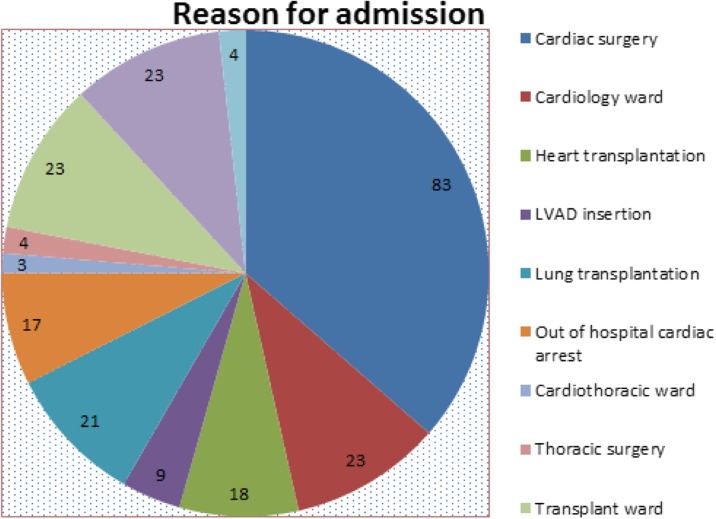
Reason for admission

**Methods:** Of the 1829 patients admitted to level 3 areas in Harefield Hospital during 2015, 229 required CRRT (12.52 %). 161 (70 %) were male, with a median age of 59 years. 109 (47.6 %) were hypertensive and 55 (24 %) were diabetic. The most frequent reason for admission was cardiac surgery (36.2 %, 83 patients), followed by medical admissions from the cardiology ward (10 %, 23 patients) and patients on veno-arterial extracorporeal membrane oxygenation (VA-ECMO, 10 %, 23 patients); further breakdown is shown in Fig. 50. The most prevalent indication for initiation of CRRT was metabolic acidosis (58.5 %, 134 patients), followed by fluid overload (15.3 %, 24 patients) and uraemia (13.1 %, 30 patients). 69.9 % of the patients were haemodynamically unstable when CRRT was initiated (as defined by the need for two or more vasoactive drugs). The ITU mortality in the CRRT group was 36.2 % (83 patients) compared with 6.01 % (110 patients) in the general group admitted to level 3 areas. The mean length of stay in ITU was 18 days in the CRRT group vs. 3.6 days in the general level 3 group.

**Conclusions:** Acute kidney injury is associated with an increased risk of morbidity, mortality, and length of stay. In our tertiary cardiothoracic unit metabolic acidosis is the most frequent indication.

**References:** Kidney Disease: Improving Global Outcomes (KDIGO) Acute Kidney Injury Work Group. KDIGO Clinical Practice Guideline for Acute Kidney Injury. Kidney inter., Suppl. 2012; 2: 1–138

**Fig. 51 (abstract A551).**
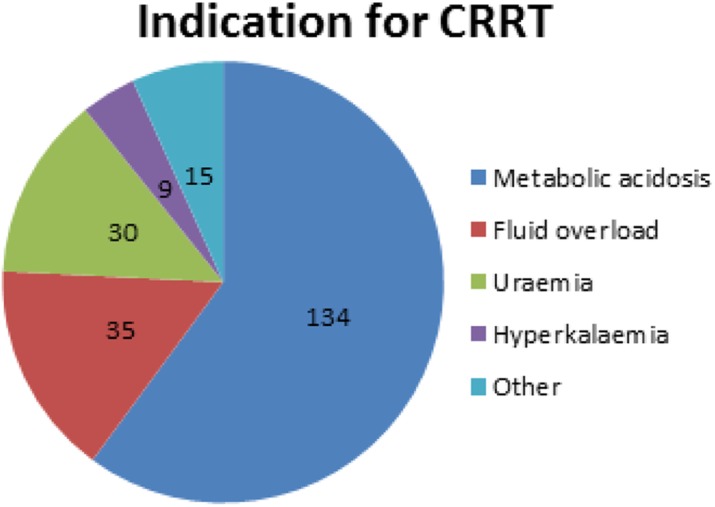
Indication for CRRT

#### A552 Stability of urinary biomarkers of acute kidney injury

##### S. Beitland^1,2^, A.-M.S. Trøseid^3^, B.S. Brusletto^3^, B.E. Waldum-Grevbo^4^, J.-P. Berg^2,3^, K. Sunde^1,2^

###### ^1^Oslo University Hospital Ullevål, Department of Anaesthesiology, Oslo, Norway; ^2^University of Oslo, Oslo, Norway; ^3^Oslo University Hospital Ullevål, Department of Medical Biochemistry, Oslo, Norway; ^4^Oslo University Hospital Ullevål, Department of Nephrology, Oslo, Norway

####### **Correspondence:** S. Beitland - University of Oslo, Oslo, Norway

**Introduction:** New biomarkers are important for early detection of acute kidney injury (AKI), but few studies have previously evaluated the stability.

OBJECTIVES The aim of this study was to investigate whether timing of centrifugation and/or storage time in a refrigerator influenced the measured concentrations of urinary AKI biomarkers.

**Methods:** Ten adult intensive care unit patients (with and without AKI) donated urine aliquoted into 8 aliquots of 2 ml. Half of the aliquots were centrifuged (500 RCF, 20 °C, 5 minutes) early (immediately) and the remaining late (after thawing). Aliquots were stored in a refrigerator for 0, 1, 2 and 3 days and frozen at −70 °C before analyses. The measured biomarkers were Cystatin C and Neutrophil Gelatinase-Associated Lipocalcin (NGAL) using the Bio-Plex Pro Human Kidney Toxicity Panel 2 assay, and Tissue inhibitor of Metalloproteinase 2 (TIMP-2) and Insulin-like Growth Factor-Binding Protein 7 (IGFBP-7) using the NephroCheck test. Measured concentrations are reported as median (interquartile range), and compared using a 2-tailed paired Wilcoxon signed rank test.

**Results:** Biomarker concentrations were similar in aliquots with early versus (vs.) late centrifugation not stored in a refrigerator (Cystatin C 1342 (52–3960) vs. 1173 (56–3887) ng/ml, p = 0.80, NGAL 1296 (42–3200) vs. 1402 (39–2661) ng/ml, p = 0.26 and NephroCheck 0.22 (0.09-3.10) vs. 0.23 (0.08-2.32), p = 0.77, respectively). Biomarker levels were comparable in early centrifuged aliquots stored in a refrigerator for 0 vs. 3 days (Cystatin C 1342 (52–3960) vs. 1312 (61–3834) ng/ml, p = 0.29, NGAL 1296 (42–3200) vs. 1085 (41–3090) ng/ml, p = 0.26 and NephroCheck 0.22 (0.09-3.10) vs. 0.26 (0.09-2.46), p = 0.29, respectively).

**Conclusions:** The measured concentrations of urinary Cystatin C, NGAL and the NephroCheck test were not influenced by timing of centrifugation and/or storage in a refrigerator up to three days.

#### A553 Aminopeptidasic activity in different urine fractions improves early diagnosis of acute kidney injury in patients undergone to cardiac surgery

##### D. García Huertas^1^, F. Manzano^1^, M.M. Jiménez- Quintana^1^, A. Osuna^1^, F. Santiago-Ruiz^1^, C. Rodríguez-Mejías^1^, R. Wangensteen^2^

###### ^1^Complejo Hospitalario de Granada, Granada, Spain; ^2^Complejo Hospitalario de Jaén, Jaén, Spain

####### **Correspondence:** D. García Huertas ^_^ Complejo Hospitalario de Granada, Granada, Spain

**Introduction:** Diverse biomarkers are employed for diagnosis of acute kidney injury (AKI) and in the last years, new biomarkers are being studied for the early detection of this disease.

**Objective:** The aim of this work is to study if measurement of aminopeptidasic activities in different urine fractions may be of interest in the early diagnosis of acute kidney injury (AKI) in patients undergone to cardiac surgery.

**Methods:** A prospective, single-center and observational study was carried out in urine samples from patients undergone to cardiac surgery obtained at arrival to Critical Care Unit. Urine samples were centrifugated 10 minutes at 1000 g and supernatants (S1) were centrifugated 15 minutes at 17000 g to remove cellular fragments and other debris. Supernatants were subjected to ultracentrifugation 60 minutes at 200.000 g to obtain exosomic fraction and a final supernatant (S2). Glutamyl aminopeptidase (GluAp) and alanyl aminopeptidase (AlaAp) activities in the different urine fractions were quantified by fluorimetry in samples from patients.

**Results:** The study included 143 patients, 80 of them developed AKI. GluAp activity was significatively increased in patients that developed AKI after surgery (p < 0.05 vs. no AKI) in S1 and S2 supernatants. AlaAp activity was increased (p < 0.05) only in S2 supernatant. No differences were found in cellular or exosomic fractions from patients that developed AKI vs. no AKI patients. ROC-AUCs to distinguish AKI from no AKI patients were greater for AlaAp and GluAp in S2 than in S1 supernatant, and they were also greater than ROC-AUC for proteinuria, albuminuria, NAG and NGAL.

**Conclusions:** Aminopeptidasic enzymes can be detected in cellular, exosomic and soluble fractions of urine. Measurement of GluAp and AlaAp in soluble fraction improves sensitivity and specificity for early diagnosis of AKI in patients undergone to cardiac surgery.

#### A554 Serum cystatine-c versus creatine phosphokinase to predict acute kidney injury according to rifle criteria in adult trauma intensive care units within first 24 hours

##### H.R. Jamaati^1^, **M. Masjedi**^2,3^, F. Zand^3,4^, S.M.R. Hashemian^1^, G. Sabetian^4^, G. Abbasi^4^, V. Khaloo^4^, S.H.a. Tabei^4^, A. Kafilzadeh^4^, H. Haddad Bakhodaei^4^

###### ^1^Shaheed Beheshti University of Medical Sciences, Tehran, Islamic Republic of Iran; ^2^Shiraz University of Medical Sciences, Anesthesia and Intensive Care Department, Shiraz, Islamic Republic of Iran; ^3^Anesthesiology and Critical Care Research Center, Shiraz University of Medical Sciences, Shiraz, Islamic Republic of Iran; ^4^Shiraz University of Medical Sciences, Shiraz, Islamic Republic of Iran

####### **Correspondence:** M. Masjedi - Anesthesiology and Critical Care Research Center, Shiraz University of Medical Sciences, Shiraz, Islamic Republic of Iran

**Introduction:** Acute kidney injury (AKI) is a common clinical problem in critically ill patients, independently predicts poor outcome.Despite significant improvements in therapeutics, its mortality and morbidity remains high. A major reason for this is the lack of early markers for AKI and hence an unacceptable delay in initiating therapy. AKI, as a component of the crush syndrome, is the second cause of death after direct trauma, although it can be prevented by early and vigorous intravenous fluid therapy .Few studies have evaluated the epidemiology of AKI in trauma.

**Objectives:** To evaluate and compare the predictive value of serum cystatin-c (Cys-c) with creatine phosphokinase(CPK) for AKI in adult trauma patients admitted in ICU.

**Methods:** Retrospective interrogation of prospectively collected data of 300 adult trauma patients aged ≥ 18 years admitted and stayed more than 24 hours in ICU were done . We measured serum cystatin-c (Cys-c) and creatine phosphokinase (CPK) of previously collected and freezed serum samples. Impression of AKI was applied according to RIFLE criteria in intensive care unit within first 24 hr (early AKI) then association of Cys-c and CPK with different stages of AKI were evaluated.

**Results:** The median age was 34.8 (1 8–80) years, 90 % were male. The crude prevalence of AKI was 193 (65 %) with a maximum RIFLE category of Risk in 1 21 (40.3 %), Injury in 67 (22.3 %), and Failure 5 (1.7 %). Younger age and male sex were independently associated with higher risk of AKI in the first 24 hours after trauma . Mean APACHE IV score between AKI and Non-AKI groups were not statistically different. Considering serum Cys-c, the lower frequency of AKI (1 44,63.2 %) was observed among the patients with Cys-c < 0.78 and the higher (49, 71 %) among the patients with Cys-c ≥ 0.78 (p value = 0.001). According to serum CPK, the lowest frequency of AKI (87, 56.1 %) was observed among the patients with CPK < 2000 and the highest (1 8, 90 %) among the patients with CPK of 5000–1 0000 (p value = 0.003). For diagnosis of AKI, Cys-c showed to have significant odds ratio of 5.874 (p value: 0.003 with 95 % CI; 1,79-1 9.23) in comparison with CPK which had no correlation (odds ratio of 1). The predictive diagnostic performance of both Cys-c and CPK were fair on the first day (area under the curve 0.565 and 0.61 8, respectively) by plotting ROC curve

**Conclusions:** In adult trauma victims admitted in ICU, serum Cys-c but not CPK could predict development of early AKI .

**References**

1- Mai T. Nguyen & Prasad Devarajan .Biomarkers for the early detection of acute kidney injury. Pediatr Nephrol 2008; 23:2151–21 57

2- Sean M. Bagshaw, Carol George, R.T. Noel Gibney, Rinaldo Bellomo . A Multi-Center Evaluation of Early Acute Kidney Injury in Critically Ill Trauma Patients . Renal Failure 2008; 30:581–589

**Grant acknowledgement**

This study was financially supported by vice chancellory of research of Shiraz university of medical sciences.

#### A555 Predictive model for acute tubular necrosis complications and the need for early renal replacement therapy

##### J.A. Diaz^1,2^, R. Silva^1,2^, D.J. Garcia^1^, E. Luis^1^, M.N. Gomez^1^, R. Soriano^1^, P.L. Gonzalez^1^

###### ^1^Instituto Mexicano del Seguro Social, Intensive Care Unit, Leon, Mexico; ^2^Universidad de Guanajuato, Intensive Care Unit, Leon, Mexico

####### **Correspondence:** J.A. Diaz - Universidad de Guanajuato, Intensive Care Unit, Leon, Mexico

**Introduction:** Acute tubular necrosis (ATN) is an important complication in critically ill patients and it is associated with the need for early renal replacement therapy (RRT) and poor prognosis^1^. It is multifactorial being the principal causes; hemodynamic instability and systemic inflammatory response syndrome^2^. In patients with AKI, considering the hemodynamic status with Shock index (SI) and inflammatory response with serum C reactive protein (CRP) ^3^ could be predictors for the development of ATN and prepare the sources for the potential need of RRT.

**Objective:** Determine the validity of the Predictive Model for ATN Complications (PMAC) and the need for early RRT in AKI.

**Methods:** A prospective study conducted in critically ill adults with AKI in the Intensive Care Unit from UMAE1 to evaluate the validity of a predictive model (PMAC) for the development of ATN, complications and the need for early RRT. Sampling was not probabilistic with a total of 30 patients. The inclusion criteria were all critically ill adult's age between 18–75 years old that developed AKI based on the definition of KDIGO´S Guidelines^4^ in creatinine value and/or urinary output. We obtained the worst SI (Cardiac rate/Systolic arterial pressure) from the patient's vital signs registration and obtained CRP serum level in milligrams per deciliter (mg/dl). PMAC is obtained by the product of Shock index and CRP. We defined ATN by Sodium excretion fraction (FeNa) more than 2 %, urine nitrogen excretion fraction (FeUrea) more than 55-63 % and/or the evidence of granular casts in urinalysis. We also registered the AKI complications and if RRT was needed in the first 72 hrs.

**Results:** We evaluated the ROC curves using PMAC. A PMAC ≥ 9.8 predicts development of ATN with a sensitivity of 92 % and specificity of 76 %. PMAC ≥ 14 predicts development of any complication due to AKI with a sensitivity of 85 % and specificity of 88 %. PMAC ≥ 23 predicts the need for early RRT with a sensitivity of 88 % and specificity of 95 %.

**Conclusions:** In critically ill patients that present AKI, PMAC is an easy to obtain, accurately and cheap predictor. A cut off ≥ 9.8 is helpful to foretell diagnosis of ATN, ≥ 14 predicts development of AKI complications and ≥ 23 the need for early RRT.

**References**

1. De Mendonca A, Vincent JL, Suter PM, et al. Acute renal failure in the ICU: risk factors and outcome evaluated by the SOFA score. Intensive Care Med 2000; 26: 915e21. 2. Jitendra Kumar. Pathophysiology of ischemic acute tubular necrosis. Nephrology 2012; 0101:18–26.

3. Friedewald, J.J. and Rabb, H. Inflammatory cells in ischemic acute renal failure. Kidney Int 2004: 66; 486–491.

4. Kidney Disease: Improving Global Outcomes (KDIGO) Acute Kidney Injury Work Group. KDIGO Clinical Practice Guideline for Acute Kidney Injury. Kidney inter., Suppl. 2012; 2: 1–138.

**Grant acknowledgement**

This study did not receive any grant from any funding agency.

**Fig. 52 (abstract A555).**
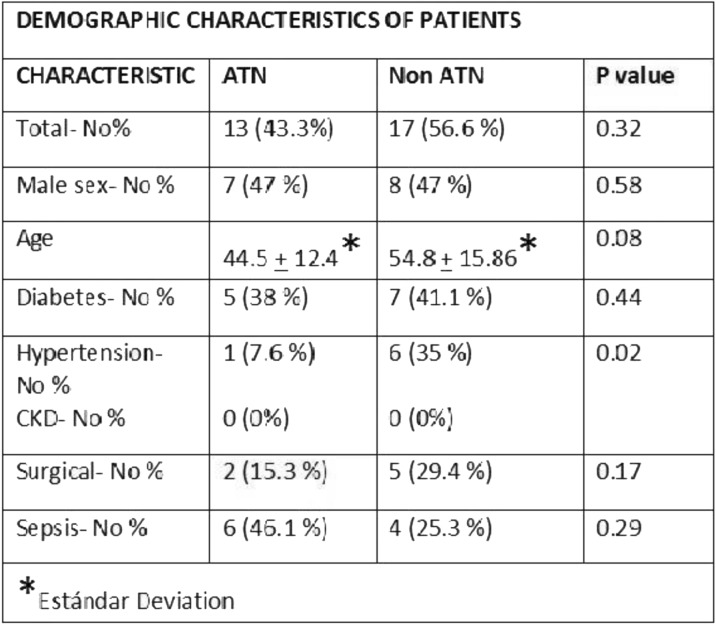
Demográfic chart

**Fig. 53 (abstract A555).**
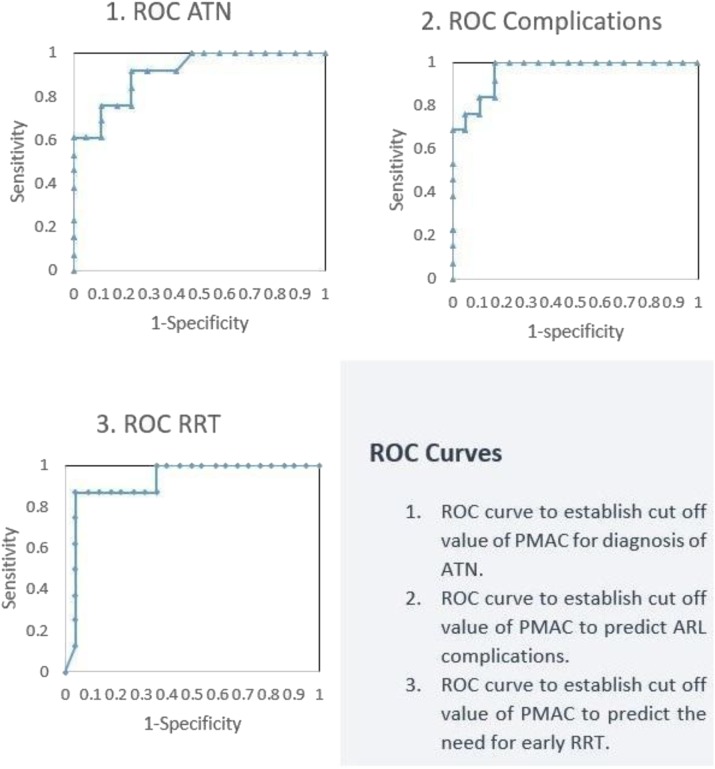
Roc curves

#### A556 Comparative study of urinary neutrophil gelatinase-associated lipocalin and interlukin-18 versus serum creatinine in early detection of acute kidney injury in critically ill patients

##### I.A. Ibrahim^1^, M.M. Rafik^2^, A.M. Al-Ansary^1^, M.A. Algendi^1^, A.A. Ali^3^

###### ^1^Faculty of Medicine - Ain Shams University, Anaesthesia, Intensive Care and Pain, Cairo, Egypt; ^2^Faculty of Medicine - Ain Shams University, Clinical Pathology, Cairo, Egypt; ^3^National Hepatology and Tropical Medicine Research Inistitute, Intensive Care, Cairo, Egypt

####### **Correspondence:** Faculty of Medicine - Ain Shams University, Anaesthesia, Intensive Care and Pain, Cairo, Egypt

**Introduction:** Many concerns are associated with the use of serum creatinine as the main determination of acute renal injury. The search for a reliable kidney injury biomarker is an area of continuous research interest.

**Objectives:** To determine weather uNGAL and/or uIL-18 are good predictors of acute kidney injury (AKI).

**Methods:** In this study, we examine urine neutrophil associated lipocalin (uNGAL) and urine interleukin (IL)-18 as predictors of AKI in comparison to serum creatinine in 30 patients admitted to ICU.

**Results:** We did not find any clear role of both biomarkers in the diagnosis and evaluation of patients with AKI. Urine NGAL and IL-18 did not show any statistical significance between patients who developed AKI versus those who did not. Although, our results showed that uIL-18 is found to be elevated in patients who developed AKI at the day of ICU admission AUC-ROC 0.805 (95 % CI: 0.514 - 1.0) and a level of 1000 pg/ml was specific but not sensitive in diagnosing AKI.

**Conclusions:** Urine NGAL and IL-18 role in diagnosing AKI cannot be clearly identified by comparing them to serum creatinine owing to the fact of different clinical value of both biomarkers.

**References**

1. Mehta RL, Kellum JA, Shah SV, Molitoris BA, Ronco C, Warnock DG, Levin A, the AKIN (2007); Acute Kidney Injury Network: report of an initiative to improve outcomes in acute kidney injury; Critical Care 11(2) R31

2. Viadya VS, Wikar SS, Ferguson MA, Collings FB Sunderland K, Gioules C, Bradwin G, Matsouaka R, Betensky RA, Curhan GC, Bonventre JV (2008); Urinary Biomarkers for Sensitive and Specific Detection of Acute Kidney Injury in Humans; Clin Trans Sci., 1(3) 200–208

**Fig. 54 (abstract A556).**
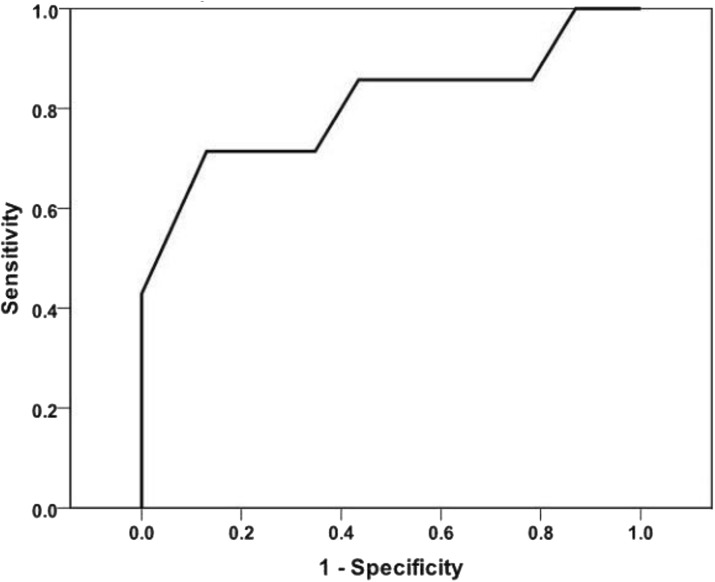
ROC curve for admission IL-18 in prediction of adm

**Table 26 (abstract A556).** Differance between AKI and no AKI groups regarding

	Time	AKI (N=13)	No AKI (N=17)	^P
Creatinine	Admission	0.82 ± 0.36	0.85 ± 0.27	^0.747
uNGAL	Admission	2.5 (1.3–3.3)	3.8 (1.0–4.8)	#0.483
uNGAL	Day 1	1.3 (1.3–4.0)	2.5 (0.8–3.8)	#0.592
uNGAL	Day 2	2.5 (1.3–3.8)	2.5 (1.0–4.0)	#0.902
uNGAL	Day 3	1.3 (1.0–3.9)	2.5 (1.0–3.9)	#0.680
uIL-18	Admission	500 (300–1650)	500 (225–700)	#0.432
uIL-18	Day 1	500 (130–750)	500 (200–725)	#0.711
uIL-18	Day 2	300 (250–650)	400(130–575)	#0.902
uIL-18	Day 3	400 (130–650)	200 (130–500)	#0.385

**Table 27 (abstract A556).** Diagnostic performance of renal biomarkers in pred

		AUC-ROC	SE	P	95% CI
Creatinine	Admission	0.593	0.112	0.409	0.374–0.813
uNGAL	Admission	0.569	0.116	0.544	0.342–0.795
uNGAL	Day 1	0.525	0.116	0.827	0.298–0.751
uNGAL	Day 2	0.503	0.114	0.981	0.278–0.727
uNGAL	Day 3	0.547	0.115	0.680	0.322–0.772
uIL-18	Admission	0.580	0.114	0.482	0.357–0.803
uIL-18	Day 1	0.549	0.115	0.662	0.325–0.774
uIL-18	Day 2	0.560	0.115	0.593	0.334–0.787
uIL-18	Day 3	0.635	0.110	0.234	0.419–0.850

**Table 28 (abstract A556).** Various biomarkers predictivity to AKI at admission

Variables	AUC-ROC	SE	P	95% CI
Creatinine	0.506	0.146	0.959	0.220–0.793
uNGAL	0.523	0.125	0.858	0.278–0.768
uIL-18	0.805	0.115	0.017*	0.514–1.000

#### A557 ADVOS® for treatment of respiratory acidosis in hypercapnic critically ill patients

##### V. Fuhrmann^1^, K. Roedl^1^, T. Horvatits^1^, A. Drolz^1^, K. Rutter^1^, D. Benten^2^, J. Kluwe^2^, S. Siedler^1^, S. Kluge^1^

###### ^1^University Medical Center Hamburg-Eppendorf, Intensive Care Medicine, Hamburg, Germany; ^2^University Medical Center Hamburg-Eppendorf, Internal Medicine 1, Hamburg, Germany

####### **Correspondence:** V. Fuhrmann - University Medical Center Hamburg-Eppendorf, Intensive Care Medicine, Hamburg, Germany

**Introduction:** ADVOS® (Hepa Wash GmbH, Munich, Germany) is a new advanced dialysis device that is approved in the European Union for treatment of kidney and liver failure. Apart from removing water soluble and albumin bound substances it is able to counteract severe acid–base-disequilibrium on an individualized basis.

**Objectives:** Aim of this study is to quantify the ADVOS®´ efficacy in improving respiratory acidosis in hypercapnic patients at the intensive care unit.

**Methods:** Retrospective analysis of patients with acute kidney failure and respiratory acidosis that were treated with the ADVOS®-device via a conventional dialysis catheter at an interdisciplinary intensive care unit.

**Results:** 10 patients were included in the analysis, 6 (60 %) were male, the mean age was 58 ± 16 years. All patients were mechanically ventilated and suffered from severe multiorgan failure. Mean SAPS II was 50 ± 12 and mean SOFA-Score was 16 ± 3. Parameters of mechanical ventilation did not change significantly during treatment with the ADVOS® device (PEEP_pre_ 11 ± 4 mbar versus PEEP_post_ 11 ± 4 mbar, p = ns, FiO_2pre_ 66 ± 21 versus FiO_2post_ 64 ± 24, p = ns, tidal volume _pre_ 371 ± 106 ml versus tidal volume_post_ 372 ± 109 ml, p = ns, respiratory rate_pre_ 28 ± 5 /min versus respiratory rate_post_ 29 ± 6 /min, p = ns). PaO2/FiO2 prior to treatment was 143 ± 77 mmHg versus 154 ± 70 mmHg at the end of treatment(p = ns).Mean blood flow rate of the ADVOS® device was 148 ± 68 ml/min, median flow rate of the concentrate was 160 ml/h and mean dialysate-pH-setting was 8,4 ± 0,5. Unfractioned heparin was used as anticoagulant in 6 patients and 4 patients had regional citrate anticoagulation. Mean duration of each treatment session was 16 ± 8 hours.pH and HCO_3−_ increased significantly during treatment with ADVOS® (pH_pre_ 7,28 ± 0,12 versus pH_post_ 7,38 ± 0,1, p < 0,001, HCO_3-pre_ 28 ± 8 mmHg versus HCO_3-post_ 32 ± 9 mmHg, p < 0,01) and P_a_CO_2_ decreased significantly (P_a_CO_2pre_ 66 ± 12 mmHg versus P_a_CO_2post_ 53 ± 10 mmHg, p < 0,001). We observed significant differences of the acid–base-status pre and post the ADVOS® device: P_v_CO_2preMem_ 67 ± 18 mmHg versus P_v_CO_2postMem_ 24 ± 15 mmHg, p < 0,001, pH_preMem_ 7,26 ± 0,14 versus pH_postMem_ 7,74 ± 0,23, p < 0,001, HCO_3-preMem_ 27 ± 7 mmHg versus HCO_3-postMem_ 32 ± 6 mmHg, p < 0,001). We could not observe any device related side effects during treatment. 28-survival-rate of the patients was 70 %.

**Conclusions:** ADVOS® is a new advanced dialysis device that is approved in the European Union. ADVOS® is able to remove CO_2_ without an extracorporeal membrane oxygenator and to support patients with respiratory acidosis.

#### A558 A survey of renal replacement therapy provision in united kingdom adult neurointensive care units

##### I. Adedugbe, G.T. Bird, Queen Square Neuroanaesthesia and Neurocritical Care Resreach Group

###### University College London Hospitals NHS Foundation Trust, London, UK

####### **Correspondence:** G.T. Bird - University College London Hospitals NHS Foundation Trust, London, UK

**Introduction:** Acute kidney injury (AKI) occurs in approximately 8-23 %^1,2^ of patients on neurointensive care units (NICU) and is an independent predictor of mortality, multiplying the risk of death by 6.17 times.^1^ The proportion of patients with chronic diseases is also increasing with a consequent increase in the number of patients requiring renal replacement therapy (RRT) on neurointensive care units. The immediate availability of renal replacement therapy on NICUs in the UK may therefore have an increasingly important impact on the morbidity and mortality of brain injured patients in the future.

**Methods:** A telephone survey of United Kingdom National Health Service (NHS) adult NICUs was undertaken, with 16 questions to illicit the logistics of delivery of RRT to NICU patients.

**Results:** Results were obtained from 81 % (25/31) of the adult neurosurgical centres in the NHS. Only 40 % (10/25) of centres have a dedicated NICU. Of these, only 4 had nursing staff trained to deliver RRT, with just 3 units possessing their own RRT equipment. 40 % (4/10) of the units with a dedicated NICU provided RRT on other intensive care units within the same hospital versus 33 % (5/15) of neurosurgical centres with mixed critical care patients. Some centres transferred their patients to other hospitals within the same trust for renal replacement therapy (30 % of dedicated NICUs versus none of the mixed intensive care units). 84 % (21/25) of units had < 30 patients a year requiring RRT. Five units used citrate regional anticoagulation for the circuit and only 4 units had point of care testing (ACT, TEG, ROTEM) for monitoring anticoagulation immediately available on the unit.

**Conclusions:** There are numerous barriers to the provision of renal replacement therapy in neurointensive care patients. This survey has not explored the physiological problems encountered with RRT in these patients, but has highlighted that the maintenance of training, competency and logistical problems sometimes affects the consistent, direct availability of RRT to NICU patients. This is especially true of units without traumatic brain injury patients, where medical staff may become deskilled in the practice of RRT over time. It also highlights the logistical challenges in providing renal replacement therapy in patients who are at high risk of intracranial bleeding without point of care testing immediately available to monitor the anticoagulation of the circuit.

**References**

1. Impact of non-neurological complications in severe traumatic brain injury outcome. Corral et al. Crit Care. 2012;16:R44.

2. Renal Dysfunction as an Independent Predictor of Outcome After Aneurysmal Subarachnoid Hemorrhage. A Single-Center Cohort Study. Zacharia et al. Stroke. 2009;40:2375–81.

#### A559 Assessing adherence to acls protocols in residents at a Canadian teaching hospital during simulated cardiac arrest

##### R.M. Kennedy^1^, S. Sharma^1^, M.B. Butler^2^, G. Yugi^3^, B.A. Haroon^2,4^, T. Witter^2,3^

###### ^1^Dalhousie University, Medical School, Halifax, Canada; ^2^Dalhousie University, Critical Care Medicine, Halifax, Canada; ^3^Dalhousie University, Anesthesiology, Pain Management and Perioperative Medicine, Halifax, Canada; ^4^Dalhousie University, General Internal Medicine, Halifax, Canada

####### **Correspondence:** T. Witter - Dalhousie University, Anesthesiology, Pain Management and Perioperative Medicine, Halifax, Canada

**Introduction:** Advanced cardiac life support (ACLS) has been the recognized standard of care in cardiac arrest since 1974. Several studies have demonstrated the importance of adherence to ACLS algorithms during cardiac arrests in improving return of spontaneous circulation^1^ and survival to hospital discharge^2^. However, many residents feel unsure about their skills in resuscitation, and those who feel confident may be falsely self-assured^3^.

**Objectives:** Analyse the adherence to ACLS guidelines in a standardized simulated cardiac arrest scenario and identify potential obstacles to guideline conformity.

**Methods:** This study used an observational design to evaluate adherence to ACLS protocol in residents attending standardized cardiac event simulations. 36 residents were exposed to the same scenario of ventricular fibrillation and were filmed and reviewed by two independent scorers. Their adherence to ACLS protocols was measured using a standardized checklist based on the 2010 AHA guidelines^4^.

**Results:** Overall adherence to ACLS protocol was fair (79.1 %). Residents consistently performed well in their assessment of the initial presentation (88.0 % adherence) and their initiation of chest compressions once pulselessness was detected (97.2 %). Quality of CPR was high in terms of hand placement, compression depth, and rate (97.2 %; 88.9 %; 97.2 %); however half (52.8 %) of the residents failed to place the backboard under the patient.

Residents struggled to keep CPR interruptions around defibrillation and rhythm analysis/pulse checks under 10 seconds (55.6 % and 59.7 % respectively). Only 63.9 % of the residents correctly identified the initial rhythm. No resident delivered a shock in under 1 minute from rhythm onset and in 36.1 % of cases it took longer than three minutes from rhythm onset to defibrillation.

**Conclusions:** In our study residents demonstrated overall satisfactory adherence to the 2010 ACLS guidelines, especially the detection of pulselessness and initiation of CPR showed good compliance. Future training should emphasize correct rhythm analysis and the handling of CPR interruptions while performing defibrillation and rhythm/pulse checks - this could lead to a better outcome in patients suffering a cardiac arrest.

**References**

1. McEvoy MD (2014) The effect of adherence to ACLS protocols on survival of event in the setting of in-hospital cardiac arrest. Resuscitation,85(1), 82–87.

2. Moretti M (2007) Advanced cardiac life support training improves long-term survival from in-hospital cardiac arrest. Resuscitation, 72(3), 458–465.

3. Wayne DB, (2006) Graduation medical residents self-assessment and performance of advanced cardiac life support skills. Medical Teacher 28(4), 365–369

4. McEvoy MD (2012) Validation of a detailed scoring checklist for use during advanced cardiac life support certification. Simulation in healthcare: journal of the Society for Simulation in Healthcare, 7(4), 222.

**Grant acknowledgement**

None.

### CARDIOVASCULAR DERANGEMENTS IN SEPSIS/DRUG THERAPIES

#### A560 Cardiac stress during septic shock can be demonstrated by release of troponin and BNP in translational studies

##### W. Khaliq, M. Singer

###### UCL, Bloomsbury Institute of Intensive Care Medicine, London, UK

####### **Correspondence:** W. Khaliq - UCL, Bloomsbury Institute of Intensive Care Medicine, London, UK

**Introduction:** Septic shock is associated with a severe cardiovascular stress response. Beta-blockade represents a novel way of addressing this; esmolol titrated to keep heart rate < 95 bpm in critically ill patients receiving high-dose noradrenaline resulted in improved outcomes [1]. We measured a series of biomarkers of cardiac stress and injury in an observational study of critically ill patients with septic shock. We then used a long-term fluid-resuscitated rat model of sepsis to assess the same cardiac markers.

Objectives. To measure the degree of cardiac stress in critical illness using the biomarkers of cardiac injury, troponin T (cTnT) and of ventricular dysfunction, B-natriuretic peptide (BNP).

**Methods:** Study 1: Observational study of 27 critically ill patients with septic shock from faecal peritonitis or community-acquired pneumonia. Daily measurements of cTnT and BNP were performed on days 0 to 3 (measured by ELISA).

Study 2: Awake, instrumented yet fully mobile male Wistar rats (350 ± 50 g) received an intraperitoneal injection of faecal slurry to induce sepsis. Fluid resuscitation (crystalloid 10 ml/kg/h) was commenced at 2 h. At 6 h, an echo-measured stroke volume rate cut-off of 0.20 ml was used to classify animals into predicted survivors (S) or non-survivors (NS) (n = 6 per group). Blood was sampled at 6 h and 24 h for cTnT and BNP (ELISA). Control animals were treated identically except for faecal slurry injection.

Results were analysed using two-way ANOVA and post-hoc testing and considered statistically significant when p < 0.05.

**Fig. 55 (abstract A560).**
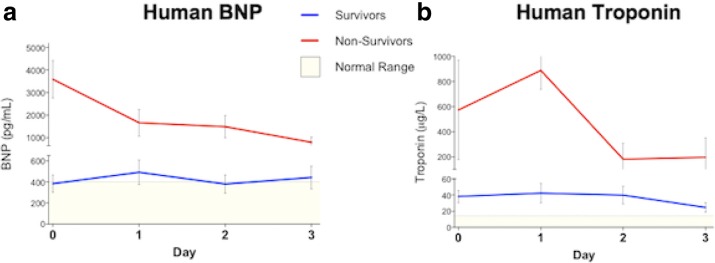
ICU patient levels of (a) BNP and (b) Troponin

**Fig. 56 (abstract A560).**
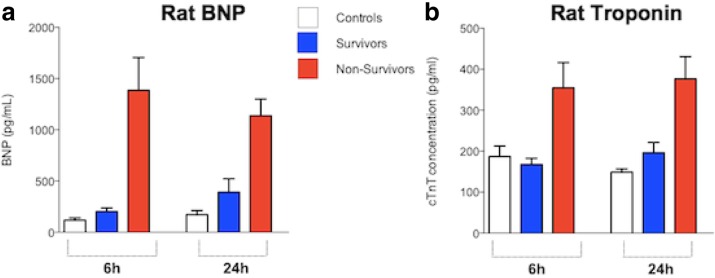
Rat sepsis levels of (a) BNP and (b) Troponin

**Results:** cTNT was significantly elevated in critically ill patients on the day of ICU admission, and was significantly higher in NS (Fig. 55). BNP was significantly elevated in NS patients only. Likewise, in the experimental model of sepsis (Fig. 56), non-surviving rats had an early elevation of the same cardiac biomarkers. (p < 0.05 for both cTnT and BNP compared to survivors).

**Conclusions:** Sepsis is associated with excess cardiac stress, as demonstrated in both a critically ill patient population and in a rat model of faecal peritonitis. Whether beta-blockade can be used to ameliorate this cardiac stress is the subject of further studies.

**References**

[1] Morelli A et al. *JAMA* 2013; 310: 1683–1691

**Grant acknowledgement**

ESICM Basic Science Award, UK Intensive Care Society Young Investigator Award, NIHR.

#### A561 Sepsis induced cardiac dysfunction as a predictor of mortality

##### A.A. Havaldar, B. Krishna, S. Sriram

###### St. Johns Medical College, Critical Care Medicine, Bangalore, India

####### **Correspondence:** A.A. Havaldar - St. Johns Medical College, Critical Care Medicine, Bangalore, India

**Introduction:** Sepsis is characterized by life threatening organ dysfunction with dysregulated immune response. Cardiac dysfunction seen in sepsis is unique as it is reversible within 7–10 days. Parker et al^1^ in 1984, showed paradoxically,lower ejection fraction in survivors of septic shock. It was suggested that non survivors,were unable to correct the vasoplegia(reduced systemic vascular resistance). This view has been questioned by a recent meta-analysis^2^. A prognostic model combining biomarkers like Troponin I and echocardiographic parameters has not been well described.

**Objectives:** To assess sepsis induced cardiac dysfunction by 2D echocardiography and Troponin I.

**Methods:** After obtaining institutional ethical committee approval,a prospective observational study was done in an university medical college from February 2016 to April 2016. Inclusion criteria were patients diagnosed with sepsis^3.^ Pregnant patients and patients with poor echo window were excluded. Echocardiographic assessment was done within 48 hrs of diagnosis of sepsis by standard methods. Primary outcome was ICU mortality and secondary outcome was ICU length of stay. Statistical analysis was done using STATA12™.Independent variables identified as significant in a bivariate analysis were included as co-variates in a logistic regression model with outcome as the dependent variable. Discrimination and calibration were assessed by receiver operating characteristics(ROC) and Hosmer-Lemeshow goodness of fit tests.

**Results:** Fifty eight patients were screened, ten were excluded due to poor echo window. Baseline characteristics and Troponin I were similar in survivors and non survivors, except APACHE II, SOFA and age. Echocardiographic parameters, MAPSE, a`and E/e` were found to be statistically significant. In the logistic predictive model only APACHE II and MAPSE were found to be significant co-variates.(APACHEII co-ef 0.4869 p = 0.02, MAPSE −9.3672 p = 0.04, constant 0.7109). The discrimination of this model was good ROC(0.95) and calibration was satisfactory(chi2(df8),1.98, p = 0.98).

**Conclusions:** Sepsis induced cardiac dysfunction assessed by echocardiography showed measurement of MAPSE when combined with APACHE II was a good predictor of mortality. Results of this study need further validation from larger study.

**References**

1. Parker MM, Shelhamer JH, Bacharach SL, Green MV, Natanson C, Frederick TM, Damske BA, Parrillo JE: Profound but reversible myocardial depression in patients with septic shock. Ann Intern Med 1984, 100:483–490.

2. Stephen J Huang, Marek Nalos and Anthony S McLean. Is early ventricular dysfunction or dilatation associated with lower mortality rate in adult severe sepsis and septic shock? A meta-analysis Critical Care 2013, 17:R96

3. Mervyn Singer, MD, FRCP; Clifford S. Deutschman, MD, MS; Christopher Warren Seymour The Third International Consensus Definitions for Sepsis and Septic Shock (Sepsis-3) JAMA February 23, 2016 Volume 315, Number 8

**Table 29 (abstract A561).** Patient characteristics

Variable	Survivors n=29	Non Survivors n=18	P value
Age	54.89(12.61)	65.94(11.51)	0.003
APACHE II	16.34(4.91)	25.84(6.44)	<0.001
SOFA	4.86(2.58)	10.57(3.70)	<0.001
a`	10.45(2.79)	7.99(3.31)	0.012
E/e`	6.32(1.75)	8.03(2.79)	0.025
MAPSE			0.026(Mann-Whitney U test

#### A562 Short-term hyperoxic ventilation does not affect sublingual microcirculation in patients with septic shock

##### E.D. Valenzuela Espinoza^1^, M.O. Pozo^2^, V.S. Kanoore Edul^2^, M. Furche^1^, M.F. Motta^1^, A. Risso Vazquez^1^, P.N. Rubatto Birri^1^, C. Ince^3^, A. Dubin^1,2^

###### ^1^Sanatorio Otamendi y Miroli, Servicio de Terapia Intensiva, Buenos Aires, Argentina; ^2^Facultad de Ciencias Médicas, Universidad Nacional de La Plata, Cátedra de Farmacología Aplicada, La Plata, Argentina; ^3^Academic Medical Center, University of Amsterdam, Translational Physiology, Amsterdam, Netherlands

####### **Correspondence:** A. Dubin - Facultad de Ciencias Médicas, Universidad Nacional de La Plata, Cátedra de Farmacología Aplicada, La Plata, Argentina

**Introduction:** The therapeutic use of hyperoxic ventilation in critically ill patients is controversial. A major concern is the induction of arteriolar vasoconstriction with subsequent microvascular hypoperfusion. Recently, severe sublingual microcirculatory alterations were reported with high oxygen inspired fraction in healthy volunteers.

**Objective:** To show the effects of short-term hyperoxic ventilation on sublingual microcirculation and peripheral perfusion in patients with septic shock.

**Methods:** The sublingual microcirculation was assessed by SDF-videomicroscopy in 11 patients with septic shock during normoxic ventilation and after 5 minutes of ventilation with FiO_2_ = 1.0. We also evaluated the capillary refill time (CRT). To rule out a delayed effect of hyperoxia, we studied 8 healthy volunteers in normoxia, and after 5´and 30´ of 100 % oxygen through a nasal mask.

**Fig. 57 (abstract A562).**
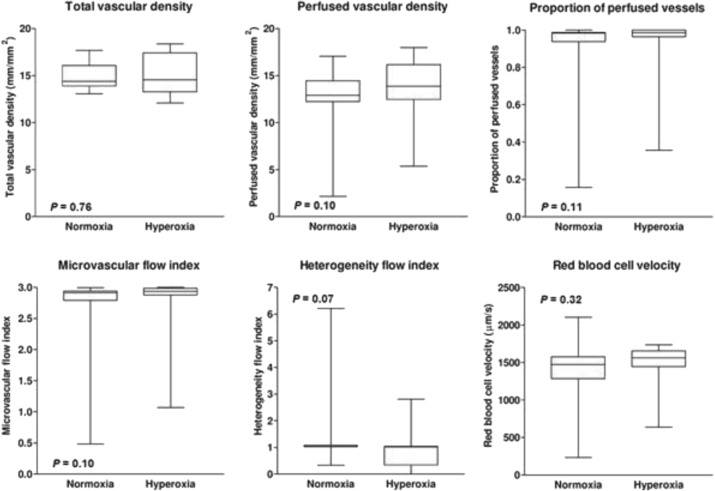
ᅟ

**Results:** In patients with septic shock, hyperoxic ventilation increased arterial PO_2_ from 75 [67–82] to 312 [278–415] mm Hg. Sublingual microcirculation (Fig. 57) and CRT (6.0 [4.0-9.4] vs. 8.3 [3.4-10.6]) remained unchanged. In normal subjects, arterial PO_2_ reached 388 [359–421] mm Hg after 5´of high FiO_2_. After 5´and 30´ of hyperoxia, there was no change in either sublingual perfusion or CRT.

**Conclusions:** In patients with septic shock and in healthy volunteers, short-term hyperoxic ventilation neither modified sublilgual microcirculation nor peripheral perfusion.

**References**

1. Orbegozo Cortés D, Puflea F, Donadello K, Taccone FS, Gottin L, Creteur J, Vincent JL, De Backer D. Normobaric hyperoxia alters the microcirculation in healthy volunteers. Microvasc Res 2015; 98:23–28

#### A563 Vasopressor agents in the treatment of shock: a network meta-analysis of 4,406 patients

##### A. Dogliotti^1,2^, A. Ramos^3^, C. Lovesio^3^

###### ^1^Instituto Cardiovascular Rosario, Rosario, Argentina; ^2^Grupo Oroño, Unidad de Epidemiologia Clinica, Rosario, Argentina; ^3^Sanatorio Parque, Terapia Intensiva y Cuidados Criticos, Rosario, Argentina

####### **Correspondence:** A. Dogliotti - Grupo Oroño, Unidad de Epidemiologia Clinica, Rosario, Argentina

**Introduction:** Many drugs are recommended as first-line vasopressor agents in the treatment of shock. There is a continuing controversy about whether one agent is superior to the other.

**Objectives:** To synthesise the evidence from trials using a multiple treatment comparison methods thereby permitting a broader comparison across multiple therapies.

**Methods:** Randomised controlled trials in patients with shock of vasopressor drugs were identified from MEDLINE, Embase, and Cochrane Central Register of Controlled Trials through July 2015. We performed a random-effects model within a Bayesian framework using Markov Chain Monte Carlo simulation to calculate pooled Odds Ratio and 95 % credibility intervals. The main outcome measure was death from any cause in-intensive care unit, in-hospital or 28 days.

**Results:** We identified 23 studies with 4,406 patients allocated to 10 treatments: dopamine, epinephrine, terlipressin, phenilephrine, and norepinephrine alone or combined with dobutamine, dopexamine, terlipressin and dobutamine or vasopressin. First, pair-wise meta-analyses with a random-effects model were carried out. Second, a random-effects model multitreatment meta-analysis within a Bayesian framework (using Markov Chain Monte Carlo simulation) was performed.

We performed 2 sensitivity analyses:

1) using only septic shock subgroups and

2) double blinded trials with intention to treat analysis.

We performed 45 comparisons. There was no significant between-group difference in the rate of death. In sensitivity analyses, the results were qualitatively similar to the results obtained with 23 studies.

**Conclusions:** There were no significant differences in the rate of death between patients with shock who were treated with any vasopressor agent.

**References**

1) Havel C, Arrich J, Losert H, et al. Vasopressors for hypotensive shock. Cochrane Database Syst Rev 2011.

2) De Backer D, Aldecoa C, Nijmi H, Vincent JL. Dopamine versus norepinephrine in the treatment of septic shock: A meta-analysis. Crit Care Med 2012; 40:725–730.

3) Oba Y, Lone NA. Mortality benefit of vasopressor and inotropic agents in septic shock: A Bayesian network meta-analysis of randomized controlled trials. Journal of Critical Care 2014; 29:706–710.

#### A564 PTP1B gene deletion improves glucose metabolism and limits cardiovascular dysfunction in experimental septic shock

##### E. Delile^1^, R. Nevière^2^, P.-A. Thiébaut^1^, J. Maupoint^1^, P. Mulder^1^, D. Coquerel^1^, S. Renet^1^, J.-C. do Rego^3^, J. Rieusset^4^, V. Richard^1^, F. Tamion^5^

###### ^1^University of Rouen, INSERM U1096, Rouen, France; ^2^INSERM U995, Lille, France; ^3^University of Rouen, Service Commun d'Analyse Comportementale (SCAC), Rouen, France; ^4^University of Lyon, UMR INSERM U1060, Lyon, France; ^5^University of Rouen, INSERM U1096 - Service de Réanimation Médicale CHU Rouen, Rouen, France

####### **Correspondence:** E. Delile - University of Rouen, INSERM U1096, Rouen, France

**Introduction:** Hyperglycemia is a feature of septic patient and has been associated with poor outcome and higher mortality. In contrast insulin has been shown to decrease mortality and to prevent the incidence of multi-organ failure but is often associated with deleterious hypoglycemia. Protein Tyrosine Phosphatase 1B (PTP1B) is a negative regulator of insulin signaling and NO production. We found that PTP1B inhibition or gene deletion improved mesenteric endothelial function, reduced cardiac and vascular inflammatory markers, leading to reduced cardiac dysfunction.

**Objectives:** The purpose of the present study was to assess the potential therapeutic effect of total PTP1B invalidation on glucose metabolism and cardiovascular insulin resistance using experimental model of sepsis.

**Methods:** Thus, in order to address this question, we developed a Cecal Ligation and Puncture (CLP) model of sepsis. CLP is followed by subcutaneous fluid resuscitation. To evaluate the potential therapeutic effect of PTP1B invalidation, we used PTP1B^−/−^ mice.

**Results:** Indirect calorimetry study showed that CLP induced a significative diminution of VO2 inspired, VCO2 expired, physical activity with food and water consumption. This study characterizes a severity of our CLP model without difference between WT vs PTP1B^−/−^.

Although this severe model, PTP1B^−/−^ mice showed improved GTT and ITT. Moreover, the limited increase of insulinemia in CLP PTP1B^−/−^ mice emphasizes the improvement of glucose metabolism.

Moreover, the heart expression of cytokines showed a significative increase of inflammation and oxidative stress in WT CLP mice compared with PTP1B^−/−^ mice. Plasmatic dosage of insulin before and 15 minutes after glucose injection showed an elevation of insulin concentration in WT CLP mice showing a functional pancreas.

Insulin- and flow-mediated dilatation assessed in isolated-perfused mesenteric arteries was abolished in WT CLP mice and was improved in PTP1B^−/−^ mice.

Isolated perfused heart study showed that CLP induced a significative reduction of left ventricular developed pressure and cardiac efficiency in WT and PTP1B^−/−^ mice. However, efficiency cardiac study showed a different utilization of substrate between WT CLP and PTP1B^−/−^ CLP, WT sham and PTP1B^−/−^ sham.

Moreover, we showed that PI3k/Akt molecular pathway is functional in CLP mice suggesting a GLUT4 expression or translocation alteration. In fact, GLUT4 expression study showed that in plasma membrane fraction and endosome fraction, GLUT4 expression is abolished in WT CLP mice.

We also found that PTP1B^−/−^ mice subjected to CLP had a higher survival rate compared to WT.

**Conclusions:** CLP-induced sepsis changes the carbohydrate mechanistic by abolishing GLUT4 expression and PTP1B inhibition restores GLUT4 expression and improves metabolic function, cardiovascular function and survival. This suggests that, PTP1B inhibition may be an attractive target for the treatment of sepsis.

**Note:** This abstract has been previously published and is available at [1]. It is included here as a complete record of the abstracts from the conference.

**References**

1. Delile E, Thiebaut PA, Do Rego JC, Coquerel D, Neviere R, Richard V, Tamion F (2016) PTP1B gene deletion improves glucose metabolism and limits cardiovascular dysfunction in experimental septic shock. Annals of Intensive Care 6(Suppl 1): P202

#### A565 Beta-blockade with esmolol modulates plasma inflammatory cytokine balance but not catecholamine levels in a long-term rat model of faecal peritonitis

##### W. Khaliq^1^, D.T. Andreis^1,2^, M. Singer^1^

###### ^1^UCL, Bloomsbury Institute of Intensive Care Medicine, London, UK; ^2^Dipartimento di Fisiopatologia Medico-Chirurgica e dei Trapianti, Università degli Studi di Milano, Milan, Italy

####### **Correspondence:** W. Khaliq - UCL, Bloomsbury Institute of Intensive Care Medicine, London, UK

**Introduction:** Septic shock is a condition of extreme physiological stress associated with significant haemodynamic, immune and hormonal abnormalities. Beta-blocker therapy is associated with improved organ dysfunction and survival in patients with severe, prolonged septic shock [1]. Precise mechanisms of putative benefit however remain uncertain.

**Objective:** To investigate the impact of esmolol on the inflammatory and catecholamine stress response during sepsis.

**Methods:** We used an awake, fluid-resuscitated rat model of faecal peritonitis in which 72 h mortality can be accurately predicted at 6 h post-insult by stroke volume (<0.20 ml) [2] and interleukin(IL)-6 [3]. After the 6 h echocardiogram, rats were randomised to receive either esmolol (75 mcg/kg/min, preceded by a loading dose of 500 mcg/kg for one minute) or equivalent volume of placebo for the next 18 hours. Rats (n = 12) then underwent repeat echocardiography, followed by terminal blood and tissue sampling. This included plasma levels of IL-6 (pro-inflammatory cytokine) and IL-10 (anti-inflammatory cytokine) (ELISA, BD Biosciences) and catecholamine levels (ELISA, Cusabio). Comparison was made against non-septic control rats (n = 6). Results were analysed using two-way ANOVA with post-hoc testing, and considered statistically significant when p < 0.05.

**Results:** Septic animals had increased levels of IL-6 and IL-10 at 24 h that were greater in predicted non-survivors (Table 30). Esmolol significantly decreased IL-6 levels in both predicted survivors and non-survivors compared to placebo-treated rats, whereas IL-10 levels were increased by esmolol. However, no change was seen in catecholamine levels.

**Conclusions:** Treatment with esmolol altered plasma cytokine balance towards a more anti-inflammatory profile in both predicted survivors and non-survivors in this model of faecal peritonitis. However, circulating catecholamines were unaffected.

**References**

[1] Morelli A et al. *JAMA* 2013; 310: 1683–1691

[2] Rudiger A et al. *Clin Sci* 2013; 124: 391–401

[3] Khaliq W et al. *Intensive Care Med Exp* 2014; 2(S1): 22

**Grant acknowledgement**

**Table 30 (abstract A565).** Data shown as median (IQR); ^p<0.05 versus placebo

		Control (n=6)	Predicted survival (n=7)	Predicted non-survival (n=5)
IL-6 (ng/mL)	Placebo	0.1 (0.0–0.1)	2.1 (2.0–3.5)	12.9 (8.2–18.4)
	Esmolol	-	1.4 (1.0–1.7) ^	3.0 (2.2–4.5) ^
IL-10 (ng/mL)	Placebo	0.0 (0.0–0.1)	0.4 (0.2–0.6)	0.7 (0.4–1.0)
	Esmolol	-	1.4 (1.3–3.2) ^	1.2 (0.7–3.2)
Adrenaline (ng/mL)	Placebo	5.5 (4.7–6.3)	7.1 (5.3–15.4)	6.2 (5.5–12.8)
	Esmolol	-	6.4 (5.2–12.3)	8.9 (7.0–9.3)
Noradrenaline (ng/mL)	Placebo	0.5 (0.2–0.7)	3.2 (2.9–4.8)	3.2 (1.2–5.1)
	Esmolol	-	2.6 (2.0–3.5)	3.0 (1.4–5.0)

ESICM Basic Science Award, UK Intensive Care Society Young Investigator Award, NIHR

#### A566 Isolated arteries from mice are not susceptible to the vasoconstrictive effects of hyperoxia

##### B. Smit^1^, Y.M. Smulders^2^, M.C. de Waard^1^, H.M. Oudemans - van Straaten^1^, A.R.J. Girbes^1^, E.C. Eringa^3^, A.M.E. Spoelstra-de Man^1^

###### ^1^VU University Medical Center, Intensive Care, Amsterdam, Netherlands; ^2^VU University Medical Center, Internal Medicine, Amsterdam, Netherlands; ^3^VU University Medical Center, Physiology, Amsterdam, Netherlands

####### **Correspondence:** B. Smit - VU University Medical Center, Intensive Care, Amsterdam, Netherlands

**Introduction:** Hospitalized patients often receive oxygen supplementation, which may lead to a supraphysiological oxygen tension (hyperoxia). Hyperoxia has hemodynamic effects, including an increase in systemic vascular resistance suggesting arteriolar vasoconstriction. Despite several in-vivo, ex-vivo and in-vitro studies involving humans and various types of animals, the mechanism behind hyperoxia-induced vasoconstriction remains unclear. To enhance our understanding of this phenomenon, we set out to develop an experimental model of hyperoxia-induced vasoconstriction in murine vessels.

**Objectives:** To develop an ex-vivo mouse model to study the underlying mechanisms of hyperoxic vasoconstriction.

**Methods:** Femoral arteries (conduit vessel) and gracilis- and mesenteric arterioles (resistance vessels) were isolated from 10–12 week old C57BL/6 mice. Vessels were pre-constricted with noradrenaline to approximately 50 % of their maximal diameter for vasodilation studies. Mesenteric arteries developed spontaneous myogenic tone. Agonist induced endothelium-dependent (acetylcholine, arachidonic acid) and -independent (nitroprusside, noradrenaline) dilation and constriction were examined under normoxic (PO_2_ ~ 80 mmHg) and several hyperoxic conditions (PO_2_ ~ 220, ~350 and ~660 mmHg) using no-flow pressure myography.

**Fig. 58 (abstract A566).**
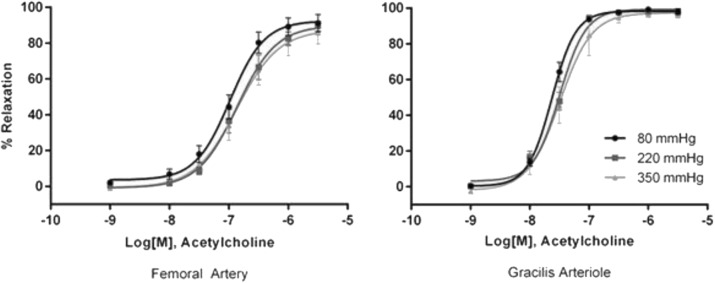
Endothelium dependent vasodilation

**Results:** Oxygen tension did not influence the endothelium-dependent dilation of femoral arteries (Fig. 58, left) or gracilis arterioles (Fig. 58, right). Endothelium independent dilation was not affected either. Comparably, oxygen tensions also did not influence the degree of spontaneous myogenic tone of mesenteric arteries or the response to arachidonic acid or noradrenaline.

**Conclusions:** In this study, the isolated femoral artery and gracilis and mesenteric resistance arterioles from mice did not show altered vascular tone responses under hyperoxic conditions. We therefore discourage researchers to use these vessels for mechanistic research of hyperoxic vasoconstriction.

#### A567 Esmolol ameliorates gut lactate production and impairment of exogenous lactate clearance in an endotoxic sheep model

##### L. Alegría^1^, D. Soto^1^, C. Luengo^2^, J. Gomez^3^, N. Jarufe^4^, A. Bruhn^1^, R. Castro^1^, E. Kattan^1^, P. Tapia^5^, R. Rebolledo^4^, P. Achurra^4^, G. Ospina-Tascón^6^, J. Bakker^1^, G. Hernández^1^

###### ^1^Pontificia Universidad Catolica de Chile, Facultad de Medicina, Departamento de Medicina Intensiva, Santiago, Chile; ^2^Hospital Clínico, Universidad de Chile, Unidad de Pacientes Críticos, Santiago, Chile; ^3^Universidad de Passo Fundo, Passo Fundo, Brazil; ^4^Pontificia Universidad Catolica de Chile, Departamento de Cirugía Digestiva, Santiago, Chile; ^5^Hospital de la Florida, Unidad de Pacientes Críticos, Santiago, Chile; ^6^Fundación Valle del Lili, Intensive Care Medicine Department, Cali, Colombia

####### **Correspondence:** L. Alegría - Pontificia Universidad Catolica de Chile, Facultad de Medicina, Departamento de Medicina Intensiva, Santiago, Chile

**Introduction:** The mechanisms of persistent hyperlactemia during endotoxic shock are probably multifactorial. Both hypoperfusionrelated anaerobic production and adrenergicdriven aerobic generation have been implicated. More recently an early and severe impairment in exogenous lactate clearance has also been described [1]. Theoretically, an excessive adrenergic response could influence all these mechanisms and thus aggravate the problem. Some small experimental and clinical studies have shown favorable effects on heart rate and hemodynamic or perfusion parameters, and also in inflammatory and metabolic parameters with beta-blockers. Esmolol (ESM) a short-acting selective beta-blocker has demonstrated to improve cardiac contractibility and vascular reactivity probably in relation to an anti-inflammatory effect. In a randomized-controlled study in stable septic shock patients, esmolol reduced heart rate (HR), decreased fluid requirements and lactate levels, and surprisingly showed a significant effect on mortality.

**Objectives:** To assess the effects of ESM on lactate production and exogenous lactate clearance in an endotoxic shock model.

**Fig. 59 (abstract A567).**
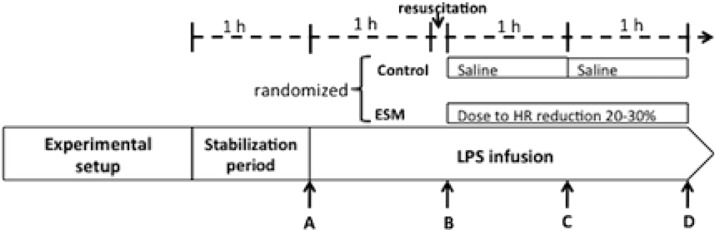
ᅟ

**Methods:** Twelve anesthetized sheep were subjected to a multimodal hemodynamic/perfusion assessment including hepatic and portal vein catheterizations, total hepatic blood flow, sublingual microcirculation, and muscle microdialysis. After the monitoring phase, all received a 5 mcg/kg LPS bolus (E coli O127:B8®) and then 4 mcg•kg1•hr1 for the rest of the experiment. After 1 hr they were volume resuscitated, and then randomized to LPS-control or LPS-ESM. Sampling and exogenous lactate clearances at different time-points (Fig. 59).

ESM was started at 15 mg/h and titrated every 5 minutes to achieve a reduction of 20-30 % in relation to HR at point B.

**Results:** ESM was hemodynamically well tolerated. Despite progressive hypotension in both groups, ESM was associated with lower arterial and portal vein lactate levels. Exogenous lactate clearance was significantly higher in ESM treated animals Table 31.

**Conclusions:** Esmolol was associated with lower arterial and portal lactate levels, and less impairment of exogenous lactate clearance in a model of septic shock. The use of ESM appears to be associated with beneficial effects on gut lactate generation and lactate clearance and exhibits no negative impact on systemic hemodynamics.

**References**

1. Tapia et al. Crit Care 2015 Apr 22;19:188.

**Grant acknowledgement**

FONDECYT 1130200, Chile.

**Table 31 (abstract A567).** **ᅟ**

Variable	Group	A	B	C	D	p
Arterial lactate (mmol/L)	Control ESM	2.0 ± 0.5 1.6 ± 0.4	5.1 ± 1.8 3.7 ± 0.9	8.1 ± 1.7 3.6 ± 1.0^b^	9.2 ± 1.8 4.5 ± 1.1^b^	a a
Muscle lactate (mmol/L)	Control ESM	3.8 ±2.0 3.3 ± 1.7	4.6 ± 1.3 3.6 ± 3.2	5.1 ± 1.3 5.0 ± 3.8	6.9 ± 3.5 5.4 ± 2.6	a
Lactate clearance (ml/kg/min)	Control ESM				2.43 ±1.14 7.32 ± 2.20^b^	
Portal vein lactate (mmol/L)	Control ESM	2.0± 0.5 1.1 ± 0.4^b^	4.2± 1.4 2.4 ± 0.7^b^	6.4±1.0 2.9 ± 0.9^b^	8.1± 1.1 4.0 ± 1.1^b^	a a

^a^Significant changes over time within groups (comparison made with Friedman test and post-hoc Bonferroni correction)

^b^Significant difference between control group and ESM group, respectively at the same time point (Comparison made with Mann-Whitney *U* test

#### A568 Ventricular arterial coupling and dynamic elastance during the early treatment of septic shock

##### P. Bertini^1^, F. Guarracino^1^, R. Baldassarri^1^, M.R. Pinsky^2^

###### ^1^University Hospital of Pisa, Department of Anaesthesia and Critical Care Medicine, Cardiothoracic and Vascular Anaesthesia, Pisa, Italy; ^2^University of Pittsburgh, Department of Critical Care Medicine, Pittsburgh, USA

####### **Correspondence:** P. Bertini - University Hospital of Pisa, Department of Anaesthesia and Critical Care Medicine, Cardiothoracic and Vascular Anaesthesia, Pisa, Italy

**Introduction:** Evaluation of cardiomechanics (end-systolic and arterial elastances), ventricular arterial coupling (VAC) and dynamic elastance (dynEl) in the critical care patient treated for septic shock has been recently addressed [1,2].

**Objectives:** To measured the effects of conventional treatment on cardiomechanics, VAC and dynEl in acute septic shock patients.

**Methods:** In 22 intensive care septic shock patients of different aetiologies we measured routine hemodynamic parameters such as mean arterial pressure (MAP), cardiac index (CI), pulse pressure and stroke volume variation (PPV, SVV) via pulse contour method, as well as VAC by the non invasively method of Chen et al.[3]. We calculated dynEl as SVV/PPV. We performed measurements prior to (T0) and after 20 ml/kg fluid challenges (T1). In a subgroup of 6 patients vasopressor support was also given between T0 and T1.

**Results:** In a multivariate analysis VAC was found to improve significantly in the fluid responder group (p = 0.005) after fluid resuscitation. Arterial elastance, which is a determinant of VAC was significantly reduced in those patients (p = 0.01) and dynEl, although not significantly, decreased. Not surprisingly we found a high correlation between dynEl and Ea.In those also receiving vasopressor therapy similar changes occurred.

**Conclusions:** Advanced bedside hemodynamic assessment is possible and underscore the potential value of defining the pathophysiologic mechanisms involved in septic shock. Although preliminary, we speculate that patients with low Ea, (higher VAC and lower dynEL) and low MAP would be fluid responders, whereas those with normal or high MAP and low Ea (high VAC and lower dynEl) fluid non-responders.

**References**

1. Guarracino F, Ferro B, Morelli A, Bertini P, Baldassarri R, Pinsky MR: **Ventriculoarterial decoupling in human septic shock**. *Critical care* 2014, **18**(2):R80.

2. Guinot PG, Bernard E, Levrard M, Dupont H, Lorne E: Dynamic arterial elastance predicts mean arterial pressure decrease associated with decreasing norepinephrine dosage in septic shock. *Critical Care (London, England)* 2015, 19:14.

3. Chen CH, Fetics B, Nevo E, Rochitte CE, Chiou KR, Ding PA, Kawaguchi M, Kass DA: Noninvasive single-beat determination of left ventricular end-systolic elastance in humans. *Journal of the American College of Cardiology* 2001, 38(7):2028–2034.

#### A569 Can hypoxic versus non-hypoxic persistent hyperlactatemia in septic shock be recognized in clinical practice? A proof-of-concept retrospective study

##### L. Alegría^1^, M. Vera^1^, J. Dreyse^1^, D. Carpio^1^, C. Henriquez^1^, D. Gajardo^1^, S. Bravo^1^, R. Castro^1^, G. Ospina-Tascón^2^, J. Bakker^1^, G. Hernández^1^

###### ^1^Pontificia Universidad Catolica de Chile, Facultad de Medicina, Departamento de Medicina Intensiva, Santiago, Chile; ^2^Fundación Valle del Lili, Intensive Care Medicine Department, Cali, Colombia

####### **Correspondence:** L. Alegría - Pontificia Universidad Catolica de Chile, Facultad de Medicina, Departamento de Medicina Intensiva, Santiago, Chile

**Introduction:** Persistent hyperlactatemia after initial septic shock resuscitation is associated with bad prognosis. However, at least three mechanisms are involved: anaerobic glycolysis in hypoperfused tissues, adrenergic-driven aerobic glycolysis and impaired lactate clearance. Only the first mechanism is sensitive to further resuscitation. Thus pursuing lactate normalization with additional fluids while confronted to non-hypoxic causes could lead to the toxicity of over-resuscitation with no clinical benefit. To develop clinical algorithms aimed at differentiating hypoxic vs non-hypoxic profiles could have relevant clinical consequences.

We hypothetized that a multimodal perfusion monitoring will aid in this purpose, since the presence of low ScvO_2_, or increased central venous-arterial pCO_2_ gradient, or an impaired peripheral perfusion together with hyperlactatemia might identify a hypoxic-related phenotype. This phenotype could be associated with worse outcomes as compared with a non-hypoxic profile.

**Objectives:** To address this subject we perfomed a proof-of-concept retrospective study to assess if this approach can effectively differentiate two clinical phenotypes among hyperlactatemic septic shock patients.

**Methods:** Retrospective analysis of a prospective database of patients with septic shock admitted to our ICU during the calendar year of 2014. Patients with persistent hyperlactatemia committed to full resuscitation and with complete multimodal perfusion monitoring and follow up after initial resuscitation were included. Statistical analysis included *t*-test and chi-square.

**Results:** 44 patients (age 64 ± 16y, APACHE II 21 ± 7, SOFA 9.9 ± 3.5, abdominal and pulmonary main sepsis sources, hospital mortality 13.6 %) were included. No difference in basal severity, hospital mortality, first 24 h fluids, cardiac index or NE requirements between patients with an hypoxic (36) compared to anon-hypoxic (8) lactate profile was observed. Other results are shown in Table 32.

**Conclusions:** Patients with a hypoxic-lactate profile required more days in the ICU, and tended to receive more MV. They also received more rescue therapies and inodilators. A multimodal perfusion monitoring might be useful to recognize a hypoxic profile among septic shock patients with persistent hyperlactatemia that appears to be associated with worse outcomes and a more complex management. Our findings should be validated in prospective studies.

**Table 32 (abstract A568).** **ᅟ**

	All patients (44)	Hypoxic (36)	Non Hypoxic (8)	P value
Arterial lactate (mmol/l)	4.8 ± 3.2	4.9 ± 3.4 (0 h) 3.4 ±2.9 (6 h) 2.5 ±1.7 (24 h	3.8 ± 1.4 (0 h) 4.0 ±1.9 (6 h) 2.7 ±2.1 (24 h)	ns
Venous O2 saturation (%)	73 ± 8	72± 9	78 ± 5	0.02
Venous-arterial pCO2 gradient (mmHg)	7.2 ± 0.5	8.4 ± 2.2	3.4 ± 1.5	<0.001
Capillary refill time (s)	4 ± 2	5 ± 2	3 ± 1	<0.001
ICU length of stay	13 ± 11	14 ± 12	9 ± 4	0.03
Mechanical ventilation (MV) days	8 ± 8	9± 9	6 ± 2	0.11
Hospital length of stay	25 (1–96)	27 ± 22	19 ± 12	0.13
Rescue therapy with high-volume hemofiltration (pts)	5	5 (14%)		
Inodilators use (pts)	7	7 (19%)		

#### A570 The clinical characteristics and influence of vasopressors-induced peripheral ischemia in shock patients

##### S. Kim^1^, M. Lee^1^, S.Y. Park^2^, S. So^2^, H. Lee^2^

###### ^1^Chonbuk National University Hospital, Nursing, Jeonjusi, Republic of Korea; ^2^Chonbuk National Universiy Hospital, Internal Medicine, Jeonjusi, Republic of Korea

####### **Correspondence:** S.Y. Park - Chonbuk National Universiy Hospital, Internal Medicine, Jeonjusi, Republic of Korea

**Introduction:** As a potent vasoconstrictor, vasopressors play a critical role in the management of fluid resistant hypotension in septic shock for maintain the mean arterial pressure more than 65 mm Hg. Because of the wide variability in individual vasopressor requirement, the absolute maximum dose of vasopressor is difficult to determine. In general, when starting vasopressors, their doses should be titrated to the desired effect by closely monitoring the adverse effects such as hypoperfusion (particularly affecting the extremities, mesentery or kidneys), dysrhythmias, myocardial ischemia, local effects, and hyperglycemia.

**Objectives:** We conducted the present study to assess the risk factors of inadequate perfusion of extremities during the vasopressors treatment in patients with severe septic shock.

**Methods:** In this cross-sectional study, we evaluated the medical records of 73 patients who received vasopressors such as norepinephrine (NE), epinephrine, dopamine, vasopressin and dobutamine more than 48 hours in patients with severe septic shock in the medical intensive care unit at a tertiary university-affiliated hospital, from April 2014 to December 2015. The patients were stratified into ischemia or non-ischemia (control) groups according to whether peripheral ischemia developed or not.

**Results:** A total 73 patients with septic shock were introduced vasopressors more than 48 hours. Basically, NE introduced in all patients but more than 2 vasopressors were 28, and 3 more than vasopressors were 14. Peripheral ischemic insults including color changes occurred in 43 patients (60 %). The median onset time for peripheral ischemia was 3.7 ± 4.2 days. Past medical conditions such as diabetes, hypertension, dyslipidemia, and cerebrovascular accidents, and clinical severities such as APACHE (Acute Physiology and Chronic Health Evaluation) II score, SAPS (severity of disease classification system), and SOFA (Sepsis-related Organ Failure Assessment) scores were not significant changes between the ischemic and non-ischemic groups. Lower body mass index (BMI) (20.0 % ± 5.4 % vs. 22.4 ± 2.9, respectively p < 0.05), need for additional inotropics, dobutamine (23 % vs. 3.3 %, respectively p < 0.05), and need for renal replacement therapy (76.7 % vs. 36.7 %, respectively p < 0.01) were significant differences between the ischemic and non-ischemic groups. While the patients who had ischemic insults were significantly higher mortality, combined vasopressors more than 2 drugs were not significant.

**Conclusions:** The use of vasopressors and its combined use more than 2 drugs may not influence on the development of peripheral ischemia in patients with severe septic shock. In the future, prospective studies are needed to suggest opinions for avoiding serious peripheral ischemia in vasopressor introduce.

#### A571 Withdrawn

#### A572 Postoperative cognitive dysfunction after cardiopulmonary bypass demonstrated by psychometric testing - is it really common complication?

##### M.B. Kačar^1^, S.M. Kačar^2^

###### ^1^Clinic for Cardiac Surgery Clinical Center of Serbia, ICU, Belgrade, Serbia, ^2^Clinic for Cardiac Surgery Clinical Center of Serbia, Surgery, Belgrade, Serbia

####### **Correspondence:** M.B. Kačar - Clinic for Cardiac Surgery Clinical Center of Serbia, ICU, Belgrade, Serbia

**Introduction and objectives:** Postoperative cognitive dysfunction (POCD) after cardiac surgery is a common complication with an unclear pathophysiology leading to significant morbidity. Goal of study was to examine the causes of cognitive impairment after cardiopulmonary bypass.

**Methods:** 84 consecutive, unselected patients undergoing CABG and/or valve procedures using CPB entered the study. Exclusion criteria included:recent stroke, high -grade carotid stenosis, chronic renal failure, hepatic insufficiency,aortic arch procedures, psychiatric desease.All pts were administered a validated neurocognitive battery (Mini Mental State Examination, Visual Memory Test) preoperatively, postoperatively prior to discharge (5–7 postoprtative day), and 3 months after operation. C-reactive protein (CRP) is also quantified from serum preoperatively, 6 h after operation and on discharge. Impact of hemodynamicaly stability, hypo- and normothermia, postoperative drainage, antifibrinolytics, age, time of CPB on POCD was assessed. T-test, Scheffe's test and ANOVA were used to demonstrate statistical significance.

**Results:** The incidence of early POCD was 17.9 % (15/84). Mini Mental State Examination on discharge was significantly impaired in this group compared with preoperative level (P = 0.023) especially in subtests such as orientation to time,attention, short-term memory, registration, exspressive speech. Visual memory test failed to detect this cognitive impairment.But, 21.3%pts (18/84) showed cognitive improvement on discharge compared with preoperative level(p = 0.034).All pts with POCD were hemodinamicaly stable, without excessive postoperative drainage, without prolonged mechanical ventilation and infection. Baseline patient characteristics and key perioperative data were similar between patients who developed early POCD compared with those who did not develop POCD (P = 0.372) . Pts with POCD had significantly elevated CRP at 6 h time point and on discharge compared with preoperative level (50.44 ± 7.3 vs.4.75 ± 1.18; p < 0,01 and 148.47 ± 5.32 vs. 4.75 ± 2.18;p < 0.001)**.**

**Conclusion:** Inflammatory stress is associated with POCD post CPB. Why some pts develop higher stress level leading to POCD is currently unknown. We dont know whether POCD is transient phenomenon, because data of psichometric tests after 3 months are lacking (research continues, we will have complete data for 3–6 months).

**References**

1. Basel R, Jaqmes R, et al.Serologic Markers of Brain Injury and cognitive Function After cardiopulmonary bypass.Ann Surg 2006;244(4):593–601.

2. Juliane K, Martin S, et al. Cardiopulmonary bypass affects cognitive brain function after coronary artery bypass grafting. Ann Thorac Surg 2001;72:1926–32.

3. Gao L, Taha R, Gauvin D, et al. Postoperative cognitive disfunction after cardiac surgery. Chest. 2005;128:3664–3670.

#### A573 Changing attitudes towards primary therapeutic hypothermia in traumatic brain injury in the United Kingdom

##### I. Uddin^1^, A.M. Belhaj^2^

###### ^1^Princess Alexandra Hospital, Anaesthetics, Harlow, UK; ^2^Southend University Hospital, Intensive Care, Essex, UK

####### **Correspondence:** I. Uddin - Princess Alexandra Hospital, Anaesthetics, Harlow, UK

**Introduction:** Traumatic brain injury (TBI) is a major cause of death and disability, particularly in the young(1). Traditionally, primary therapeutic hypothermia (PTH) was seen to be beneficial, and there has been reported evidence of modest hypothermia as a neuroprotectant that improved outcomes(2). However, most recent evidence revealed, when high quality trials were analysed, that the benefits of PTH on mortality and neurological outcome were lost(3). In addition, many studies have shown PTH to cause adverse effects(4).

**Objectives:** Our aim was to assess how the use of PTH for TBI has evolved in the United Kingdom in light of recent evidence.

**Methods:** We conducted a survey of 20 neuro-critical care units across the UK in 2010 and 2015. An electronic questionnaire examined local protocols for PTH, triggers for commencing and discontinuation, target temperature ranges, use of muscle relaxants, cooling equipment, monitoring and complications. A follow-up survey was conducted to assess for a change in local guidelines in view of recent evidence.

**Results:** In 2010, 90 % of the 20 neuro-critical care units had guidelines for the management of TBI, 89 % of which included PTH as part of their management: 37 % targeted a temperature of 33-34 °C, 42 % targeted 34-35 °C, 16 % targeted 35-36 °C, while 5 % normothermia. Practice showed wide variation between units with regards to triggers for starting PTH (for ICP control: 85 % and all severe TBI: 15 %), stopping PTH (ICP target: 82 %, a set time limit: 18 %), cooling equipment (intravascular 38 %, convection methods 62 %), temperature monitoring (rectal, oesophageal and intravascular) use of muscle relaxants (always with PTH 35 %, only with shivering 65 %), neuro-monitors (ICP and transcranial doppler are most common) and most encountered complications (immunoparesis and infection, cold diuresis, dysrhythmias and electrolyte imbalance). The 2015 follow-up survey, in contrast, showed that all surveyed hospitals now have guidelines for the management of TBI,and that PTH is no longer part of these guidelines, with 100 % of responding hospitals now targeting normothermia.

**Conclusions:** The follow-up survey suggests that the use of PTH has fallen dramatically out of favour in UK neuro-critical care units since 2010. Recent evidence casts doubt on the benefits and suggest the possibility of harm.This has likely contributed to the trend of maintaining normal body temperature.

**References**

(1) Ghajar J. Traumatic brain injury. Lancet 2000, 356:923–929.

(2) Dietrich WD, Bramlett HM. The evidence of hypothermia as a neuroprotectant in traumatic brain injury. Neurotherapeutics 2010;7: 43–50

(3) Georgio A.P. & Manara A.R. Role of therapeutic hypothermia in improving outcome after traumatic brain injury: a systematic review. Br. J. Anaesthesia 2013;110:357–367

(4) Qiu WS LW,Shen H, Wang WM, et al. Therapeutic effect of mild hypothermia an outcome of patients with severe traumatic brain injury. Chin J Traumatol 2005;8:27–32

**Fig. 60 (abstract A573).**
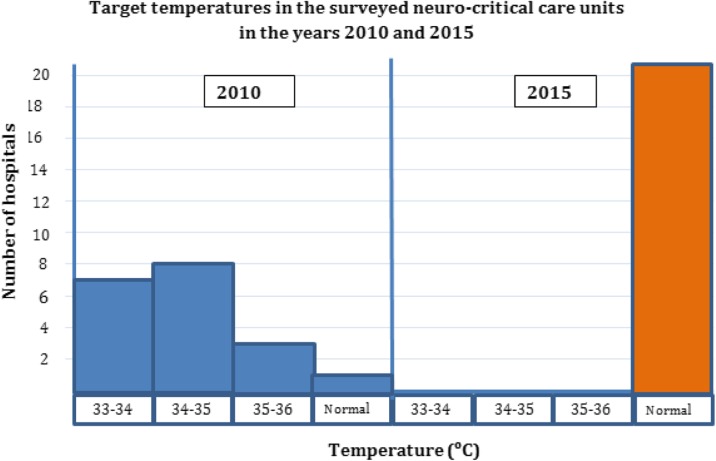
PTH graph

#### A574 Training needs assessment (TNA) in comparison with the results achieved after completing of the trainings for specialists of EU project technical assistance for alignment in organ donation in turkey

##### M.A. Aydın^1^, D. Avsec^2^, A. Kapuağası^1^, Ç. Kaymak^1^, L. Kovach^3^, İ. Şencan^1^, B. Meço^4^, M. Özçelik^4^, N. Ünal^4^

###### ^1^Ministry of Health, Ankara, Turkey; ^2^Ministry of Health, Lubiana, Slovenia; ^3^European Union, Budapest, Hungary; ^4^Ankara University, Ankara, Turkey

####### **Correspondence:** Ç. Kaymak - Ministry of Health, Ankara, Turkey

**Introduction:** Organ transplantation has become an important treatment option for patients with end-stage organ failure with the advances in the technology and related medical sciences. It is very important to improve the health care professionals' knowledge and awareness on organ donation and transplantation along with the safety and quality of donor organs.

**Objectives:** The main goal of the TNA Study was to identify weak points or 'gaps' in the professional's knowledge in organ donation and transplantation procedures in Turkey.

**Methods:** This cross sectional study was conducted via both qualitative and quantitative research methods during April 2013-May 2014. The questionnaire was related to obstacles, strengths, weaknesses and opportunities in the Turkish donor programme. The main purpose of the interview was to obtain in-depth insight into the training needs of Turkish transplant experts. A World Health Organization licensed Hennessy-Hicks training needs analysis questions (Hennessy, Hicks 2013) were incorporated in the assessment were used.

**Results:** The questionnaire was disseminated randomly among 134 health personnel (50 doctors and 84 nurses) involved in Turkish donor programme. The analysis of the questionnaires revealed that the knowledge about brain death (BD) diagnostics was not good enough in the study population. Furthermore, the group of all respondents was not experienced in the brain death determination: 22 % of them have not determined BD yet, 30 % of respondents 1–3 times, and only 20 % of respondents among doctors have determined BD more that 10 times. In doctors group, only 69,6 % of respondents perceive it as biological death - the percentage should be higher indicating no doubts in the concept of BD. A big part (32 %) of respondents estimated their acquaintance with donation procedures and organization of the national donation programme as little; 26 % are somewhat acquainted and 13 % not at all. Regrettably, 20 % of all respondents (%12 of doctors) do not absolutely support organ donation. And 4 % would not accept an organ from a deceased donor if needed. Furthermore, the results of self-evaluation of communication knowledge, skills and experiences for the performance of family interviews were too low: only 2 % of all respondents feel to have very much of knowledge, skills and experiences and 20 % feel they have not at all enough knowledge or skills for performing the discussions.

**Conclusions:** The results of the interviews revealed that lack of knowledge, awareness, responsibility and positive attitude towards about BD diagnosis and organ donation process of medical staff and nurses. Finally, lack of knowledge, skills and experiences related to the communication with the family.

**Acknowledgment**

The 'Training Needs Assessment (TNA) Study' was conducted as part of B2 activities within the project Technical Assistance for Alignment in Organ Donation Europe Aid/131052/D/SER/TR

#### A575 Organ conscription: a moral, just and maximally efficient organ procurement policy

##### C. Lazaridis^1,2^

###### ^1^Baylor College of Medicine, Neurology, Neurocritical Care, Houston, USA; ^2^BCM, Center for Ethics, Houston, USA

**Introduction:** There are currently over 120,000 people awaiting for a lifesaving organ transplant in the US with 22 patients per day dying on the waiting list.^1^ An organ shortage is also the case for most European countries. Furthermore, the demand is rising due to increasing burden of certain types of organ failure, and widening eligibility criteria for transplants. In regards to procurement policies the debate is often exhausted between opt-in and opt-out, and not enough attention has been devoted to a “non-opt” policy. Such a policy, in the literature, is better known as organ conscription.^2, 3^ Conscription entails the routine procurement of organs from the dead, making consent and family veto irrelevant. The policy tends to be uncritically rejected as being an extreme utilitarian position that violates core deontological side-constraints such as autonomy and personal sovereignty.

**Objectives:** To examine the permissibility of conscription via normative ethics and formal justice analyses.

**Methods:** Systematic review of the literature on the arguments for and against conscription to describe the current state of the debate. Subsequently and from a moral theory standpoint the policy is examined under the light of major normative theories such as consequentialism, deontology, and “mixed” views such as Parfit's triple theory. From a formal justice perspective, I employ Rawls's liberal principle of legitimacy and Sen's capabilities approach.

**Results:** There are three strands of arguments for and against conscription. These are based on normative ethics, justice and metaphysics. Contrary to main-stream views, conscription is not only defensible in consequentialist terms but obtains an even stronger force when examined under a deontological and mixed-theory lights, as well as formal justice theory. Potential conscription from patients who have been declared dead by neurologic criteria has been estimated to increase organ supply by many thousands per year.

**Conclusions:** We ought to reconsider our procurement policy in order to face organ shortage in ways that are ethical, just and maximally efficient. Organ conscription is such a policy.

**References**

1. United Network for Organ Sharing (UNOS) (2016) Organ donation and transplantation. UNOS, Richmond, Virginia. http://www.unos.org. Accessed 26 Jan 2016

2. Spital A, Taylor JS (2008) Routine recovery: an ethical plan for greatly increasing the supply of transplantable organs. Curr Opin Organ Transpl 13(2):202–206

3. Lazaridis C. Transforming ICU death into life-radically more. Intensive Care Med. 2016 Mar 21. [Epub ahead of print]

**Grant acknowledgement**

None.

#### A576 Pain and anxiety levels and preferences in ICU patients

##### B. Jenni-Moser, M.-M. Jeitziner

###### Universitätsklinik für Intensivmedizin, Bern, Switzerland

####### **Correspondence:** B. Jenni-Moser - Universitätsklinik für Intensivmedizin, Bern, Switzerland

**Introduction:** Pain and anxiety are distressing for patients in intensive care units (ICUs) and effective pain and anxiety assessment and management are problematic in ICU settings. Existing research identifying the level and trajectory of pain and anxiety patterns in ICU patients during the first days of ICU treatment is limited, as is information on patients' needs regarding analgosedation.

**Objectives:** To examine pain and anxiety patterns in ICU patients during the first three ICU days, and to identify patient preferences for analgosedation.

**Methods:** A prospective study was performed in the University Hospital Interdisciplinary (medical-surgical) ICU. Pain was assessed every four hours during the first three ICU days. Pain intensity was measured with a numeric rating scale (NRS), verbal rating scale (VRS), and behavioral assessment scale. Anxiety was assessed every 8 hours with either the NRS or VRS. Depth of sedation was measured using the Richmond Agitation-Sedation Scale (RASS). Patient preferences regarding analgosedation were assessed one week following ICU discharge using semi-structured interviews. Descriptive analyses were performed to identify pain and anxiety patterns and patient preferences.

**Results:** The sample consisted of 141 ICU patients (mean age: 68.77). The majority (55 %) were treated for heart disease. Patients were treated in the ICU on average for 4.6 days. Pain medications administered in the first three days included fentanyl for 94 % of the patients, propofol for 88 % of the patients, and midazolam for 44 % of the patients. Pain levels were relatively stable and consistent at a low intensity. Mean pain scores were highest on day 1 and slowly decreased to reach the lowest levels on day 3. NRS scores: Day 1: 3.96 (SD 3.58); Day 2: 3.84 (SD 2.79); Day 3: 2.37 (SD 1.74). Anxiety scores followed a similar pattern. Most of the patients were sleepy to lightly sedated (RASS: −1 to −2). The deepest sedation was found on the first ICU day. In general, 35 patients (25 %) experienced light sedation as helpful because it allowed them to comprehend what was happening. 17 patients (12 %) preferred moderate sedation and 89 patients (63 %) would rather have been deeply sedated so that they would be protected from awareness of stressful or difficult situations. Rapid changes in levels of sedation was experienced as stressful, anxiety provoking and disorienting.

**Conclusions:** This study demonstrated that patients in an ICU do not experience high levels of pain or anxiety when they are only lightly sedated. Patients need continuous updates in information pertaining to their treatment to help cope with the uncertainties arising from changes in levels of sedation.

#### A577 Multiprofessional team perspective in regards to early mobility process

##### M.S. Galassi^1^, F.L. Sales^1^, K.C.L. de Moraes^1^, C.L. Batista^1^, J.A. de Souza Júnior^1^, T.B. Marcari^1^, R. Lobato^1^, C.S.A.A. Castro^1^, L.M. de Souza^1^, F.F.P. Rodrigues^1^, N.G. Correa^1^, A.M. Pelegrini^1^, R.A.C. Eid^1^, K.T. Timenetsky^1^, D. Cazati^2^

###### ^1^Hospital Israelita Albert Einstein, São Paulo, Brazil; ^2^Hospital Albert Einstein, Pacientes Graves, São Paulo, Brazil

####### **Correspondence:** M.S. Galassi - Hospital Israelita Albert Einstein, São Paulo, Brazil

**Introduction:** Early mobility is defined as any activity capable of providing benefits to the patient from adequate positioning and passive movement to exercises and functional postures as orthostatism and ambulation. For early mobility success it is necessary a multiprofessional team working.

**Objective:** To collect multiprofessional team perspective in order to verify their involvement in early mobility process.

**Methods**: During a three days period we performed small groups dynamics with clinical examples to elucidate early mobility process. At the end, we evaluated the teams' perspective asking them to respond to one of each questions: 1) What do you consider important to the patient/family? 2) How can we contribute due to this experience?

**Results:** 474 professionals were approached (cleaning assistant, nursing assistant, nurses, speech therapists, physiotherapists, and clinicians). 155 professionals (33 %) chose to answer the first question. The most frequent responses were: learn to listen (11 %), put yourself in the others point of view (10 %), team work (6 %), care with respect and love as if your own family member (6 %). The other 319 professional (67 %) chose to answer the second question. We separated the answers in regards to attitude, consequence and feeling of this experience. Attitude: motivation (13 %), willpower (105), commitment (7 %), focus (7 %), attention (6 %). Consequence: dedication (17 %), safeness (14 %), humanization (14 %), communication (105). Feelings: empathy (12 %), affection (11 %), love (10 %), fulfillment (9 %), patience (8 %), understanding (7 %).

**Conclusion:** There is an emotional involvement of health care professional due to patients' assistance performance incentive.

**References**

1. Castro-Avila, A. C.; Serón P.; Fan E.; Gaete M.; Mickan S. Effect of early reabilitation during intensive care unit stay on functional status: systematic review and meta-analysis. PloS One 10(7); 2015.

2. Hodgson C. L.; Berney S.; Harrold M.; Saxena M.; Bellomo R. Clinical review: Early patient mobilization in the ICU. Critical Care 2013, 17:207.

#### A578 Therapeutic hypothermia in ICU: results from a cross sectional study from a private ICU in Brazil

##### M. Lobato, P.S. Diniz, L.L. Rocha, A.M. Cavalheiro, N.M. Lucinio, E.R. Santos

###### Hospital Israelita Albert Einstein, São Paulo, Brazil

####### **Correspondence:** M. Lobato - Hospital Israelita Albert Einstein, São Paulo, Brazil

**Introduction:** Therapeutic hypothermia is indicated for refractory intracranial hypertension [1] and post cardiac arrest [2]. It may have effects on controlling secondary brain injury, which may occur after traumatic brain injury or brain ischemia.

**Objectives:** To describe clinical and epidemiological profile of patients submitted to therapeutic hypothermia in a private ICU in Brazil.

**Methods: Design.** Cross sectional. Period and setting: January 2012 to January 2015 in a 41-bed ICU from a tertiary private hospital in Sao Paulo, Brazil. We included all ICU adult patients (age >18 years) submitted to therapeutic hypothermia for any reason during the period of study. Trained personnel retrospectively collected data regarding demographic data, such as age, hospital admission diagnosis, comorbidities, mechanical ventilation, vasopressors, laboratory parameters, therapeutic hypothermia indication, method of cooling, hypothermia duration and maintenance, target temperature, and complications. Therapeutic hypothermia indication and management was in accordance with international society guidelines.

**Results:** A total of 39 patients were submitted to therapeutic hypothermia during the study period. Most of the patients were male (23/39). About 1/3 of the patients were between 62–72 years. Hypertension (20/39), diabetes (12/39) and hypothyroidism (7/39) were the most prevalent comorbid conditions. Post cardiac arrest was the major indication for therapeutic hypothermia (16/39) followed by traumatic brain injury with refractory intracranial hypertension (6/39). All patients were induced with cooling blanket and 97,4 % (38/39) with ice in inguinal and axillary regions. Gavage was used in 48,7 % (19/39) and 4 °C saline infused in 41 % (16/39). Mean target cooling temperature was achieved in 2.2 (±1.6) hours and mean rewarming time was 20.7 (±8.2) hours. The two most frequent hypothermia complications were ventricular arrhythmias (10.3 %) and bleeding (thoracic, intra-abdominal or thoracic) (7.7 %). For those patients whose indication was hypothermia after post cardiac arrest (n = 25), the distribution of cardiac arrest rhythms was PEA (11/25), VT (10/25), and asystole (4/25). Intra-hospital arrest was slightly more prevalent (13/25) than extra-hospital. The mean CPR time was 20.9 (24.4) minutes. For this cohort of patients, intra-hospital mortality was 10.26 % (4/39).

**Conclusions:** In our cohort of patients, therapeutic hypothermia was mostly indicated for post cardiac arrest patients with appropriate management and low mortality and complication rates.

**References**

1. Andrews PJD et al. Hypothermia for Intracranial Hypertension after Traumatic Brain Injury. NEJM. 2015;373(25):2403–12.

2. Callaway CW et al. Part 8: Post Cardiac Arrest Care: 2015 American Heart Association Guidelines Update for Cardiopulmonary Resuscitation and Emergency Cardiovascular Care. Circulation. 2015;132(18 Suppl 2):S465-82.

**Grant acknowledgement**

None.

#### A579 Impact of restricted hip movement during ECMO on later joint mobility

##### **M. Norrenberg**, A. Gleize, J.C. Preiser

###### Erasme University Hospital, Université Libre de Bruxelles, Department of Intensive Care, Brussels, Belgium

####### **Correspondence:** M. Norrenberg- Erasme University Hospital, Université Libre de Bruxelles, Department of Intensive Care, Brussels, Belgium

**Introduction:** Bed rest, sedation and immobilization are unavoidable during the first days of treatment with Extra-Corporeal Membrane Oxygenation (ECMO), especially when cannulas are inserted through the femoral vessels. The effects of immobilization of the hip on later joint mobility on the side of a femoral cannula are unknown.

**Objectives:** To compare the range of motion (ROM) of immobilized joints to that of the other joints, which were passively mobilized.

**Methods:** All patients requiring veno-venous or veno-arterial ECMO with one femoral cannula in situ were included. Joints not affected by ECMO were mobilized every day and evaluated by goniometry every 3 days until ECMO withdrawal. After ECMO withdrawal, all joints were mobilized daily and evaluated every 3 days until ICU discharge. ROM was compared before and after mobilization (Student's t test) and in immobilized and mobilized joints (ANOVA).

**Results:** Ten patients (age 49 ± 15 years, 8 male, ICU mortality 40 %) with veno-venous (n = 5) or veno-arterial (n = 5) ECMO were included. Passive mobilization was well tolerated by all patients. The duration of sedation and ECMO was 6 ± 3 days. ROM was assessed until day 21 ± 13.Hip and knee ROM increased over time from the day of ECMO withdrawal to the day of ICU discharge (by 24 % for hips [p < 0.01] and by 17 % for knees [p < 0.01]) on both sides.

**Conclusions:** The results of this pilot study suggest that immobilization of a joint because of the presence of an ECMO cannula does not result in functional impairment.

**References**

De Jonghe B, Bastuji-Garin S,Sharshar Tarek, Outin H, Brochard L Does ICU-acquired paresis lengthen weaning from mechanical ventilation? Intensive Care Medicine 2004;30:1117–1121

**Grant acknowledgement**

None.

**Table 33 (abstract A579).** ROM

	Mean ROM (°) side of cannula D0	Mean ROM (°) side of cannula Ddis	Mean ROM (°) side without cannula D0	Mean ROM (°) side without cannula Ddis
Hip	87+13	108+11*	85+13	105+10*
Knee	105+10	129+18*	112+12	126+21*

* p< 0,01 D0 vs Ddis D0: at ECMO withdrawal Ddis: at ICU discharge

#### A580 A critical pathway for deceased donation in Hospital Universitario Puerta de Hierro

##### I. Fernández Simón, S. Alcántara Carmona, I. Lipperheide Valhonrat, J. Palamidessi Domínguez, A. Naharro Abellán, P. Matía Almudévar, F. Dávila, J.J. Rubio, A.J. Ramos

###### Hospital Universitario Puerta de Hierro Majadahonda, Majadahonda, Spain

####### **Correspondence:** I. Fernández Simón - Hospital Universitario Puerta de Hierro Majadahonda, Majadahonda, Spain

**Introduction:** Although the Spanish transplant network is regarded as one of the best in the world due to its high rate of donors (approximately 40 per million), nowadays the number of organs needed exceeds the demands.

**Objectives:** To describe the end of the life care practices in patients suffering from severe non-reversible brain injury in order to evaluate their suitability as potential organ donors and to introduce a pathway for their identification and transformation into real donors.

**Methods:** Multicentre, observational and prospective study (ACCORD project) in Spanish hospitals.

Two period study:

- First period (data collection): review of all the deceased patients in our hospital due to severe non- reversible brain injury and identification of potential organ donors.

- Second (intervention period): creation of a pathway to overcome obstacles for the identification of potentials donors and convert them into real donors.

**Results:** During the data collection phase we identified 24 patients with severe non-reversible brain injury. Thirteen of them (52.4 %) were males and 6 (25 %) were between 60 and 69 years old. The principal cause of death was stroke (haemorrhagic or ischemic). Fifteen patients (62.5 %) were admitted to the Intensive Care Unit (ICU) and the other nine (37,5 %) were not considerer by their primary care team as subsidiary of ICU care or as potential donors.

Of the 13 patients admitted to the ICU, six died in brain dead and nine after circulatory dead. Four of the deceased patients in brain dead were real donors and five in the circulatory death group [37,5 % (n = 9) absolute donation rate].

Of the nine patients not admitted, only one had medical contraindications for donation (33,3 % of potential donors).

In order to prepare for the intervention period, the data were analysed and shared with the different medical teams involved in the care of these patients and an algorithm was elaborated in order to identify the potential donors. Recollection of data for this second period is now underway.

**Conclusions:** Hospitalized patients with severe non-reversible brain injury could contribute to the increase in the number of real donors.

Programs aimed to spread the knowledge regarding the role of these patients as potential donors, and the creations of pathways and protocols to activate the transplant coordination team could be fundamental to increase the number of available organs.

The participation of the intensivist in this kind of projects is essential as conditional ICU admission plays an important role.

**References**

- ACCORD

- ACCORD SPAIN

- Domínguez-Gil B1, Delmonico FL, Shaheen FA, Matesanz R, The critical pathway for deceased donation: reportable uniformity in the approach to deceased donation. Transpl Int. 2011 Apr;24(4):373–8.

**Grant acknowledgement**

- Puerta de Hierro Universitary Hospital (all services)

- ONT

#### A581 Comparison between ICU bedside renal Doppler ultrasound and radioisotopes imaging tests after kidney transplantation

##### Á.J. Roldán Reina^1^, N. Palomo López^2^, M. Adriaensens Pérez^2^, D.X. Cuenca Apolo^2^, L. Martín Villén^2^, F.M. Porras López^2^, I. Palacios García^2^, J.R. Naranjo Izurieta^2^, J.J. Egea Guerrero^2^, Renal Transplantation HUVR

###### ^1^Hospital Universitario Virgen del Rocío, UCI, Sevilla, Spain; ^2^Hospital Universitario Virgen del Rocío, Sevilla, Spain

####### **Correspondence:** Á.J. Roldán Reina - Hospital Universitario Virgen del Rocío, UCI, Sevilla, Spain

**Introduction:** In recent years there have been major advances in renal transplantation, including postoperative management, reflected in decreased in-hospital mortality. However, it remains a complex procedure in a group of patients with high comorbidity. Acute graft failure is one of the most common complications, increased in recent years due to non-heart beating donors (NHBD). Bedside ultrasound and radioisotope imaging tests are routinely used during postoperative management in ICU.

**Objectives:** To analyze the concordance of renal doppler ultrasound (RDU) performed at bedside and radioisotopes tests after renal transplantation. Evaluate delayment in renal function in NHBD.

**Methods:** Retrospective, observational study from 2013 to 2014. Inclusion criteria: patients admitted in the ICU in the postoperative of renal transplantation. Variables: Demographic information, type of donation, analytical values, diuresis and bleeding during the ICU stay, results of the imaging tests (ultrasound resistance index less than 0.7 and good perfusion in scintigraphy), transfusion, creatinine levels at discharge, days of hospitalization in the ICU and mortality. Descriptive statistical analysis using U of Mann–Whitney test for differences between groups and Kappa test for the agreement between flow tests.

**Results:** 172 patients were included in our study. 62 % were male. The median age was 52 (RI 41–61) years old. Characteristics of the patients are shown in Table 34. The median time of ICU stay was 1 day (RI 1–2). No deaths were registered. Agreement between imaging tests reached a Kappa Index of 0.5 (p < 0.001) (Table 35).

**Conclusions:** Kidney transplantation postoperative has a short length of stay at the ICU.

Renal grafts from un-controlled NHBD showed a delay in the improvement of kidney function compared with other types of donors. A medium agreement between RDU and scintigraphy was observed.

**References**

1. Manikkam Suthanthiran, Terry B. Strom. Renal Transplantation. N Engl J Med 1994;331:365–376

2. Abramowicz D, Cochat P, Claas F et al. European Renal Best Practice Guideline on kidney donor and recipient evaluation and perioperative care. Nephrol Dial Transplant (2013) 28: ii1-ii71

3. Maarten G. Snoeijs,* Douglas E. Schaubel, Ronald Hene et al. Kidneys from Donors after Cardiac Death Provide Survival Benefit. J Am Soc Nephrol 2010. 21: 1015–1021.

4. Maarten Naesens, Line Heylen, Evelyne Lerut et al. Intrarrenal resistive index after Renal Transplantation. N Engl J Med 2013;369:1797–806.

**Grant acknowledgement**

We gratefully appreciate the invaluable of ICU staff.

**Table 34 (abstract A581).** ᅟ

Type of donor	Brain death	NHBD type II	NHBD type III	Living donor
N (%)	129 (75)	12 (7)	10 (5,8)	21 (12,2)
Lactate at ICU admission, median (IR)	1,6 (1,1–2,1)	1,4 (1–3,8)	1,3 (1,05–1,75)	1,3 (0,85–2,25)
Creatinine at ICU admission, median (IR)	6,01 (4,58–7,31)	6,65 (5,53–10,20)	6,47 (4,515–7,06)	5,31 (3,95–6,11)
Creatinine at Hospital discharge, median (IR)^a^	1,83 (1,35–2,84)	4,75 (3,24–6,24)	2,92 (1,86–4,35)	1,47 (1,05–1,66)
Diuresis during first 24 hours of ICU admission(%)				
Polyuria Oliguria Anuria	57,5 30,7 11,8	25 50 25	30 30 40	19 1 0
Bleeding during ICU stay (%)	5,8	9,1	0	3
Transfusion during hospitalization (%)	51,2	54,5	80	33,3

^a^Statistically significant differences

**Table 35 (abstract A581).** Agreement tests

	MAG3, n(%)			
	Normal test	Yes	No	Total
RDU, n(%)	Yes	136 (89,5)	16 (10,5)	152
	No	3 (20)	12 (80)	15
		139	28	167

#### A582 Development of an information booklet on rehabilitation in critical care

##### S. Calvert^1^, M. Quint^1^, K. Adeniji^1^, R. Young^2^

###### ^1^Queen Alexandra Hospital, Critical Care, Portsmouth, UK; ^2^Sheffield Hallam University, Faculty of Health & Wellbeing, Sheffield, UK

####### **Correspondence:** S. Calvert - Queen Alexandra Hospital, Critical Care, Portsmouth, UK

**Introduction:** Survival following critical illness is improving and the focus is moving towards optimisation of recovery. However, rehabilitation is poorly understood and national guidance recommends the need for information to be delivered to patients and their carers in an appropriate way (NICE CG83, 2009). In order to demonstrate adherence to national guidance and improve the patients' and their carers' awareness about rehabilitation in the acute phase of recovery following critical illness, this project aimed to develop, implement and evaluate an information booklet on rehabilitation after critical illness.

**Objectives:** To evaluate the impact of introducing an information booklet on compliance with CG83 standards.

**Methods:** A three stage approach was used. Firstly, current evidence was critiqued to identify the important and required informational needs. This information was then used to design and develop the information booklet by the author. This was then introduced and evaluated for a period of 8 weeks. Compliance with NICE CG83 was analysed using the guideline specific audit tool. A locally produced service evaluation questionnaire and a booklet feedback form were used to generate qualitative and quantitative outcomes.

**Results:** Over the evaluation period the booklet was issued on n = 29 occasions, with n = 13 completing analysis. Following the implementation of the booklet, compliance with NICE guidance (see Table 36) improved from 0 % to 100 %. Improvements were made in all but two areas of the service evaluation questionnaire. Out of 53 feedback comments received, 45 were positive.

**Conclusions:** Production of the critical illness information booklet was an effective method in meeting national guidelines for information provision to critical care patients and their carers. Patient feedback for the booklets was positive but on-going evaluation and adaptation of the current booklet is vital to ensure sustainability of its use. More rigorously designed research trials could give more reliable and generalisable findings.

**References**

1. NICE 2009. Rehabilitation after critical illness - Clinical Guideline 83. England: National Institute of Health and Care Excellence (NICE).

**Table 36 (abstract A582).** **ᅟ**

Criterion 5	% of patients or carers who received the following information during their critical care stay : • critical illness, interventions and treatments. • equipment. • possible short-term and/or long-term physical and non-physical problems which may require rehabilitation.
Pre Booklet:	0%
Post Booklet:	100%
Criterion 8	% of patients who received the following information before or as soon as possible after their discharge from critical care: • rehabilitation pathway • differences between critical care and ward-based care. • transfer of clinical responsibility to a different medical team.
Pre Booklet:	0%
Post Booklet:	100%
Criterion 9	% of patients identified as at risk who were given the contact details of the healthcare professional(s) coordinating their rehabilitation care pathway, on discharge from critical care.
Pre Booklet:	N/A (due to 0% compliance criterion 1 - named healthcare professional co-ordinating care pathway)
Post Booklet	100%

#### A583 Introduction of standardised critical care transfer bags to improve patient safety

##### D.D. Shevill^1^, E. Robertson^1^, P. Garside^2^, E. Walter^2^

###### ^1^Royal Surrey County Hospital NHS Foundation Trust, Department of Anaesthesia, Guildford, UK; ^2^Royal Surrey County Hospital NHS Foundation Trust, Department of Intensive Care, Guildford, UK

####### **Correspondence:** D.D. Shevill - Royal Surrey County Hospital NHS Foundation Trust, Department of Anaesthesia, Guildford, UK

**Introduction:** 11,000 inter-hospital transfers of critically ill patients are carried out each year (1). These transfers carry a significant risk; technical complications occur in up to 36 % of inter-hospital transfers (2). Recommended guidelines are available from the

Association of Anaesthetists of Great Britain and Ireland (AAGBI) and the Intensive Care Society (ICS).

**Objectives:** The department's existing system and equipment for transfers were perceived to be inadequate. This project was to assess the current system and implement any necessary change.

**Methods:** The contents of three existing bags were audited to assess uniformity. Users' perception and experience of the bags were audited. The results were poor. Following this, a standardised kit list and Standard Operating Procedure (SOP), compliant with ICS guidelines (3), were agreed by all members of the anaesthetic, theatre and ICU departments. New bags and protocols were introduced. Users' perception of the bags was then re-audited.

**Results:** The audit of the old bags showed that only 27 % of items were present in all 3 bags, that there was no standardised kit list, and that some items were out of date.

Overall rating of the bags increased from 3.5 to 8.4 following the introduction of these changes.

**Conclusions:** Introduction of a standardised kit list and a robust system for checking the bags has improved consistency, safety and user satisfaction. Following further use, the kit list will be reviewed and revised as necessary. Consideration should be given to the Surrey Critical Care Network adopting these bags and procedures.

**References**

1. Mackenzie PA, Smith EA, Wallace PGM. Transfer of adults between intensive care units in the United Kingdom: postal survey. BMJ 1997; **314**: 1455–1456.

2. Droogh JM, Smith M, Absalon AR, Ligtenberg JJM, Zijlstra JG. Transferring the critically ill patient: are we there yet?. Critical Care 2005; **19**: 62.

3. Guidelines for the transport of the critically ill adult. 2011. London, The Intensive Care Society

**Fig. 61 (abstract A583).**
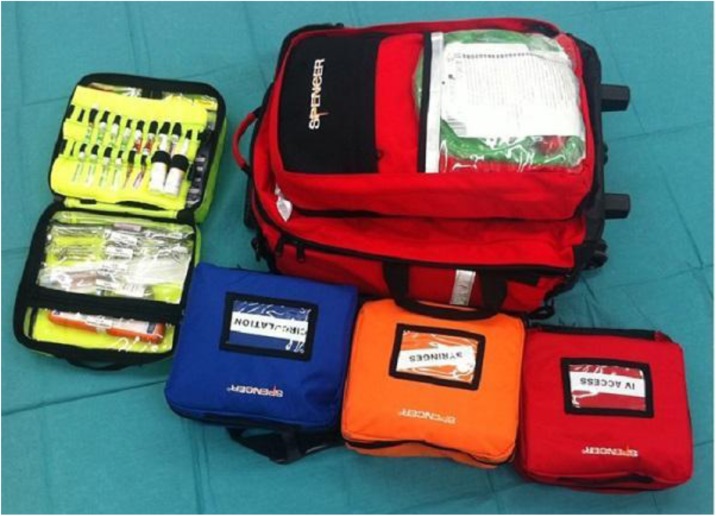
Picture of transfer bag

**Fig. 62 (abstract A583).**
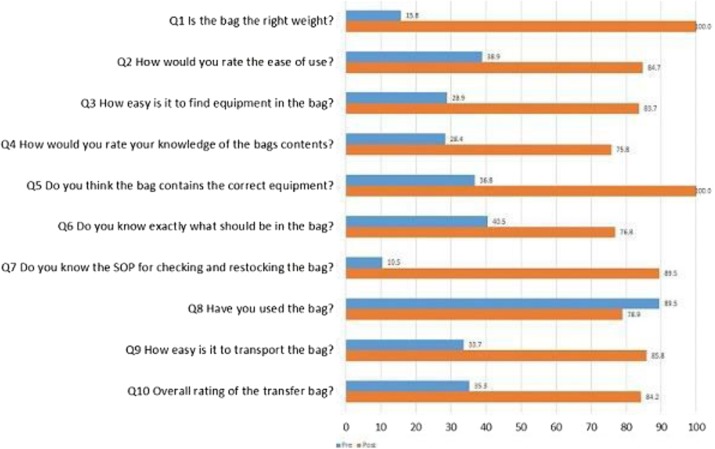
Table of questions

**Fig. 63 (abstract A583).**
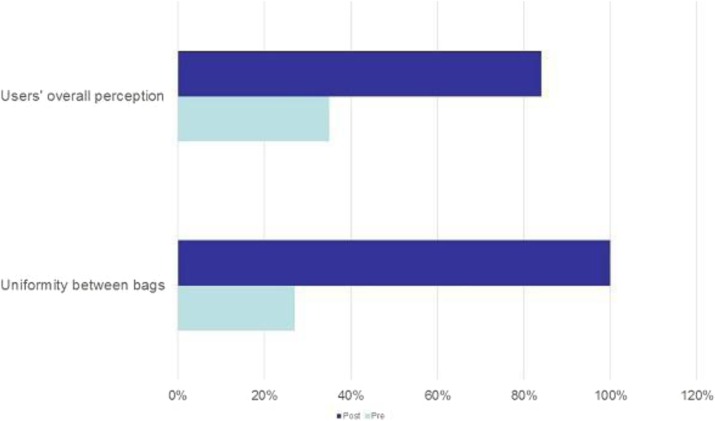
Table of questions 2

#### A584 Implementing the abcde bundle in a general Italian intensive care unit

##### P. Isotti^1^, M.M. De Vecchi^2^, A.E. Perduca^2^, A. Negro^2^, G. Villa^2^, D.F. Manara^2^, L. Cabrini^2^, A. Zangrillo^2^

###### ^1^IRCCS San Raffaele Scientific Institute, Milan, Italy; ^2^IRCCS San Raffaele Scientific Institute, Milano, Italy

####### **Correspondence:** P. Isotti - IRCCS San Raffaele Scientific Institute, Milan, Italy

**Introduction:** Delirium and Intensive Care Unit Acquired Weakness are two of the most common sequelae in patients recovered in Intensive Care Unit (ICU) [1,2]. The ABCDE bundle (Awakening and Breathing trials, Choice of sedative and analgesics, Delirium monitoring and Early mobility) is a multicomponent approach effective in preventing these complications [4]. Despite a large amount of literature about the ABCDE bundle is available, a previous attempt of implementation in an Italian environment has never been reported.

**Objectives:** The aim of this study was to implement the ABCDE bundle in a general ICU of a large university hospital in northern Italy.

**Methods:** A multidisciplinary team, consisting of critical care physicians and nurses, reviewed the literature and developed a protocol tailored on the Italian environment. Before the ABCDE bundle was part of the routine practice, education of nurses and physicians has been provided through meetings, reminders and poster that summarized the key steps of the protocol was posted by the patients´ bedside. After the ABCDE bundle was part of the routine practice a quality improvement (QI) program to strengthen the bundle knowledge has been realized: every nurse and physician attended a training course while data about facilitators and obstacles to the bundle implementation was collected. Data about adherence to the protocol has been collected to evaluate the effect of QI program.

**Results:** The ABCDE bundle has been implemented in November 2014. The Early mobility protocol became standard care from March 2015. During the QI program, some hindering factors to the implementation were identified, such as communication issues between team members, lack of resources and low confidence with the protocol. In the first quarter post-implementation 27 % of total patients were mobilized, in the second quarter post-implementation 67 % of total patients were mobilized dangling or out-of-bed. Adverse events related to the bundle were not observed. A year after the initial implementation, the “F” phase has begun to be incorporated into everyday practice, with a wider opening hours to relatives and a project of live musicians.

**Conclusions:** Although there is no available data about a previous implementation of the ABCDE bundle in an Italian setting, our experience demonstrate that it is feasible and safe. Further efforts are needed to spread the knowledge of the ABCDE bundle and improve adherence to protocol.

**References**

1. Mehta S, Cook D, Devlin JW, Skrobik Y, Meade M, Fergusson D, et al. Prevalence, risk factors, and outcomes of delirium in mechanically ventilated adults. *Crit Care Med*. 2015 Mar;43(3):557–66.

2. Hermans G, Van den Berghe G. Clinical review: intensive care unit acquired weakness. *Crit Care*. 2015 Aug;19:274.

3. Morandi A, Brummel NE, Ely EW. Sedation, delirium and mechanical ventilation: the "ABCDE" approach. *Curr Opin Crit Care*. 2011 Feb;17(1):43–9.

### ORGAN DYSFUNCTION IN SEPSIS

#### A585 Myocardial injury in critically ill patients with community-acquired pneumonia

##### J.F. Frencken^1,2^, L. van Baal^1^, L.M. Peelen^1,2^, D.W. Donker^1^, J. Horn^3^, T. van der Poll^4,5,6^, W.A. van Klei^7^, M.J.M. Bonten^2,8^, O.L. Cremer^1^

###### ^1^University Medical Center Utrecht, Intensive Care Center, Utrecht, Netherlands; ^2^University Medical Center Utrecht, Julius Center for Health Sciences and Primary Care, Utrecht, Netherlands; ^3^Academic Medical Center, University of Amsterdam, Intensive Care, Amsterdam, Netherlands; ^4^Academic Medical Center, University of Amsterdam, Center for Experimental and Molecular Medicine, Amsterdam, Netherlands; ^5^Academic Medical Center, University of Amsterdam, Center for Infection and Immunity, Amsterdam, Netherlands; ^6^Academic Medical Center, University of Amsterdam, Division of Infectious Diseases, Amsterdam, Netherlands; ^7^University Medical Center Utrecht, Department of Anesthesiology, Utrecht, Netherlands; ^8^University Medical Center Utrecht, Department of Medical Microbiology, Utrecht, Netherlands

####### **Correspondence:** J.F. Frencken - University Medical Center Utrecht, Julius Center for Health Sciences and Primary Care, Utrecht, Netherlands

**Introduction:** Myocardial injury, as reflected by elevated plasma cardiac troponin levels, has repeatedly been reported in patients with community-acquired pneumonia (CAP), but its temporal dynamics have not been systematically studied. Furthermore, although troponin release has been associated with increased mortality, its etiology still remains unknown.

**Objectives:** Our aim was to determine the incidence of troponin release in CAP patients and provide a detailed description of the clinical and pathophysiological setting during which it occurs.

**Methods:** We enrolled consecutive patients admitted with CAP to the intensive care units (ICU) of two tertiary care centers in the Netherlands between January 2011 and May 2015. Patients transferred from another hospital, patients after cardiac arrest, and patients not meeting criteria for organ failure were excluded. High-sensitivity cardiac troponin I (hs-cTnI, Abbott ARCHITECT STAT High Sensitive Troponin-I) was measured daily during the first week. ICU days were categorized into days with troponin release (days followed by a 40 % increase in hs-cTnI on the next day and an absolute rise >26 ng/L), days without troponin release (days followed by a >40 % and >26 ng/L decrease in hs-cTnI, or a value beneath the upper reference limit (URL) of 26 ng/L), and days with possible troponin release (if fulfilling none of these conditions).

**Results:** Among 200 patients with CAP we analyzed 179 subjects, yielding 709 classifiable ICU days. Median age was 63 (IQR 50–73) years, 113 (63 %) were males, and the median APACHE IV score was 83 (IQR 68–106). Troponin release above the URL occurred in 145 (81 %) individuals and peaked at a median of 136 ng/L (IQR 32–771). Patients with troponin release had more comorbidities (Charlson score 6.4 vs. 2.5 median, p-value 0.06), longer ICU stays (8.3 vs. 5.4 days, p-value 0.02), and higher hospital mortality (30 % vs. 15 %, p-value 0.06) than those without. Of the 709 observation days, 72 were classified as definite, 208 as possible, and 429 as no troponin release. Troponin release most frequently occurred on day 1 (32 %) with the majority (65 %) occurring before day 3. Inflammation and coagulation factors (fever, reduced platelet count, increased prothrombin time), as well as supply–demand mismatch factors (hypotension, high shock index, noradrenalin use, and respiratory failure) were increasingly prevalent on days with troponin release (Table 37). Furthermore, several extrinsic factors (e.g., acute kidney injury and cardiotoxic medication use) were also associated with troponin release.

**Conclusions:** Troponin release occurs in a majority of CAP patients. Increased myocardial workload (resulting in supply–demand mismatch) and an activated coagulation system could be potential causes of this injury.

**Grant acknowledgement**

This study was performed within the Molecular diagnosis And Risk stratification of Sepsis (MARS) project (grant-04 l-201).

**Table 37 (abstract A585).** ICU day characteristics

Variables	No troponin release	Possible troponin release	Definite troponin release	p-value
Inflammation/coagulation factors				
C-reactive protein (mg/L)	149 (72–255)	172 (74–256)	206 (60–303)	0.54
Fever (temperature > 38.5°C)	51 (13%)	20 (10%)	19 (27%)	0.03
Platelet count (10/L)	182 (105–252)	147 (74–224)	135 (70–214)	<0.001
Prothrombin time (seconds)	15 (13–16)	15 (14–17)	16 (14–19)	<0.001
Supply-demand mismatch factors				
Tachycardia (hours HR>120 beats/min)	0 (0–1)	0 (0–0)	0 (0–3)	0.16
Hypotension (hours MAP<60 mm Hg)	0 (0–0)	0 (0–1)	0 (0–1)	0.002
Shock index (hours HR/SBP > 1)	0 (0–6.0)	0 (0–5.0)	2 (0–11)	0.03
Noradrenalin dose (ng/kg/min)	89 (48–163)	98 (63–195)	138 (63–358)	<0.001
Respiratory failure (PaO2/FiO2<200)	273 (65%)	141 (69%)	56 (78%)	0.03
Severe anemia (Hb<5.0mmol/L female, <5.5mmol/L males)	61 (14%)	51 (25%)	16 (22%)	0.005
Extrinsic factors				
Acute kidney injury score*	125 (29%)	84 (40%)	33 (46%)	<0.001
Acute stroke,	8 (2%)	4 (2%)	3 (4%)	0.32
Use of cardiotoxic medication	18 (4%)	16 (8%)	8 (11%)	0.01
Acute heart failure	60 (14%)	26 (13%)	10 (14%)	0.79
Acute peri/myocarditis	3 (1%)	2 (1%)	1 (1%)	0.53
Acute myocardial infarction	0 (0%)	1 (0%)	2 (3%)	0.003

Table shows median (quartile 1–quartile 3) or n (%). *RIFLE stage at least ‘risk’

*ICU* intensive care unit, *HR* heart rate, *MAP* mean arterial pressure, *SBP* systolic blood pressure, *PaO2* partial pressure of oxygen in arterial blood, *FiO2* fraction of inspired oxygen, *Hb* haemoglobin, *RIFLE* risk injury failure loss end stage classification, *ng* nanogram, *kg* kilogram, *min* minute

#### A586 Evolution & impact of thrombocytopenia in septic shock

##### C.E. Menard^1^, A. Kumar^2,3^, E. Rimmer^4^, S. Doucette^5^, A.F. Turgeon^6,7^, B.L. Houston^4^, D.S. Houston^4^, R. Zarychanski^2,4^

###### ^1^University of Manitoba, Internal Medicine, Winnipeg, Canada; ^2^University of Manitoba, Critical Care, Winnipeg, Canada; ^3^University of Manitoba, Infectious Disease, Winnipeg, Canada; ^4^University of Manitoba, Haematology, Winnipeg, Canada; ^5^University of Dalhousie, Halifax, Canada; ^6^Universite Laval, Anesthesia, Quebec City, Canada; ^7^Universite Laval, Critical Care, Quebec City, Canada

####### **Correspondence:** C.E. Menard - University of Manitoba, Internal Medicine, Winnipeg, Canada

**Introduction:** Thrombocytopenia in patients with septic shock is common, occurring in 20-58 % of patients. While previous investigations have correlated platelet nadir to adverse outcomes, the time course describing both the evolution and recovery of thrombocytopenia in septic shock have not been well defined.

**Objectives:**

(1) To characterize the evolution of thrombocytopenia in patients diagnosed with septic shock, and

(2) to investigate the independent association of thrombocytopenia and clinically relevant patient outcomes.

**Methods:** Retrospective propensity-matched cohort study of adults patients with septic shock who were admitted to an intensive care unit (ICU) in one of two tertiary care hospitals in Winnipeg, Canada between 2007 and 2012. Data for this study was obtained from the Co-operative Antimicrobial Therapy of Septic Shock (CATSS) database which was further linked to the Winnipeg Regional Health Authority Critical care database and the Laboratory Information System. The time course of incident (new) thrombocytopenia was described relative to vasopressor requirements. Clinical outcomes of thrombocytopenic patients were compared to non-thrombocytopenic propensity-matched patients.

**Results:** Of 980 patients, 165 (16.8 %) had thrombocytopenia at ICU admission; 271 (27.7 %) developed thrombocytopenia in the ICU. The median time from admission to thrombocytopenia was 2 days (interquartile range [IQR] 1–3). Among survivors, the median time from incident thrombocytopenia to platelet recovery was 6 days (IQR 4–8). The average platelet nadir was 62 (SD 24.6) in survivors, versus 52 (SD 26.3) in non-survivors (p = 0.003). Platelet recovery lagged behind clinical recovery. The median time from vasopressor independence to platelet recovery was 2 days (IQR 0–4). Thrombocytopenia was not significantly associated with hospital mortality (OR 1.17; 95 % CI 0.81-1.69), but was associated with ICU length of stay (9 vs. 6 days; p < 0.01), duration of ventilation (7 versus 4 days; p < 0.01), duration of vasopressor use (4 versus 3; p < 0.01), major bleeding events (41.2 % vs. 17.6 %; OR 3.28), red blood cell transfusions (2 vs. 0; p < 0.01), and renal replacement therapy (69 % vs 40 %; OR 2.00).

**Conclusion:** Thrombocytopenia occurs early in the development of sepsis. In septic shock, platelet recovery lags behind clinical recovery. Thrombocytopenia is independently associated with a longer duration of vasopressor and ventilator support and increased major bleeding events, but may not be associated with increased mortality.

#### A587 Association between autonomic imbalance and recovery of organ function in early septic shock

##### B. Bollen Pinto^1^, M. Carrara^2^, M. Ferrario^2^, K. Bendjelid^1^

###### ^1^Geneva University Hospitals, Department of Pharmacology, Anaesthesia and Intensive Care, Geneva, Switzerland; ^2^Politecnico di Milano, Department of Electronics, Information and Bioengineering (DEIB), Milan, Italy

####### **Correspondence:** B. Bollen Pinto - Geneva University Hospitals, Department of Pharmacology, Anaesthesia and Intensive Care, Geneva, Switzerland

**Introduction:** Autonomic dysfunction is associated with increased mortality in septic shock (SS) [1] but little is known about the mechanisms behind this relation.

**Objective:** To analyze the association between changes in cardiovascular indices extracted from arterial blood pressure (ABP) and heart rate (HR) waveforms and progression of organ failure in SS.

**Methods:** This is a preliminary analysis of patients recruited in Geneva University Hospitals for the prospective observational study Shockomics, from 10.2014 to 12.2015. Twenty SS patients followed during the first 3 ICU days were stratified according to change in SOFA score from day 3 vs. day 1 into “Recovery (R)” (ΔSOFA < −1, n = 13) and “Non-recovery (NR)” (ΔSOFA ≥ 0, n = 7) groups. Time series of systolic (SAP) and diastolic (DAP) AP and heart period (HP) were extracted. Spectral indices were computed via autoregressive model as very low (VLF: 0–0.04Hz), low (LF: 0.04-0.15 Hz) and high frequency power (HF: 0.15-0.4 Hz). Baroreflex sensitivity (BRS) was computed by the spectral analysis and transfer function methods. Wilcoxon Ranksum and Friedman tests were used to assess differences between groups in each day and differences within the same group over the 3 days, respectively. Significance is for p < 0.05.

**Results:** There were no significant differences between groups in demographic characteristics or comorbidities. For the HP series, there was a significant increasing trend in total power from day 1 to 3 in the R group (p = 0.0249); LF% also increased in the R group only (p = 0.0125). Mean DAP values increased significantly in the R group from day 1 to 3 (p < 0.01). LF power (mmHg^2^), LF% and LF/HF in the DAP series increased significantly in the R group (p = 0.0183, p < 0.01 and p = 0,0498 respectively). In the 2^nd^ and 3^rd^ ICU days LF power (mmHg^2^), and LF% were higher in R compared NR patients (p = 0.0394 and p = 0.0476 respectively). There were no significant differences in the SAP series. Baroreflex indices showed an increasing trend in both groups without statistically significant results.

**Conclusions:** Variability of HR significantly increased in SS patients presenting organ function improvement over the first 3 ICU days. This may be driven by increased sympathetic autonomic activity. The mean value of DAP, considered to reflect vascular resistance driven by sympathetic vasomotor tone, raised only in the Recovery group. The ability to increase DAP could be a sign of responsiveness to vasoconstrictors. Interestingly SAP was not different between the 2 groups; this could be due to the fact that it is used as target for therapy. Further investigations are required to find specific associations with drug dosage, namely symphatotonic drugs, and clinically relevant outcomes.

**References**

[1] Pontet J 2003

**Grant acknowledgement**

Shockomics was awarded a FP7 grant (NCT02141607).

#### A588 Bilirubin's (un)friendly role in septic shock

##### J. Nunes, P. Diaz, G. Silva, S. Escórcio, S. Chaves, M. Jardim, N. Fernandes, M. Câmara, R. Duarte, C.A. Pereira, J. Vieira, J.J. Nóbrega

###### Hospital Dr. Nélio Mendonça, Intensive Care, Funchal, Portugal

####### **Correspondence:** J. Nunes - Hospital Dr. Nélio Mendonça, Intensive Care, Funchal, Portugal

**Introduction:** Mortality in severe sepsis and septic shock is predicted independently by a variety of factors, such as, hyperbilirubinemia, since there is evidence that it can induce inflammation, and apoptosis.^1–3^ However, animal data suggested that bilirubin has antioxidant properties that might be cytoprotective.^1,3^ To date, evidence of the discriminative power of bilirubin on survival due to its alleged antioxidant effects are lacking.

**Objectives:** We hypothesized that milder elevations of bilirubin might be protective and associated to better outcomes. Our secondary outcome is to identify a level of bilirubin with discriminative power either for survival or mortality.

**Materials and methods:** We conducted a retrospective study using our unit database, collecting data from 2008 to 2015, including patients >18 years-old, meeting the criteria for severe sepsis and septic shock according to the American College of Chest Physicians/Society of Critical Care Medicine Consensus Conference.^4^ We identified the highest serum bilirubin levels within 72 hours of ICU admission and then stratified into 4 cohorts based on hepatic SOFA cut-off values. SOFA and APACHE II scores were recorded.

**Results:** We analyzed 642 patients. The highest bilirubin level was 25,1 mg/dL. Stratifying in the four cohorts, we found a significant decrease in survival from a bilirubin ≥1,2 (OR from 2 to 7,2 (p < 0,001)) with the highest impact from moderate increases (Wald score of 26) (graphic 1). Patients with >11,9 mg/dL had a mean survival of 3 days. There was also a significant difference in severity scores, renal function, liver enzymes and lactate values between the groups, which was analyzed in a Logistic regression. There was no significant difference for UCI-days and length of mechanical ventilation.

**Discussion:** We found hyperbilirubinemia to be independently associated with increased risk of death but no difference in other outcomes, in this population. Despite a lower mortality rate, moderate elevations were not protective but were found to have a higher predictive weight to ICU mortality. We ponder if the protective role of mild elevations, might be true only for patients with hyperbilirubinemia over a much longer time frame that critical patients, which should be studied in future prospective studies.

**Conclusion:** Elevated bilirubin levels in patients with severe sepsis and septic shock were significantly associated with an increased risk of ICU mortality, but not longer ICU stay and length of mechanical ventilation. The protective role of the stress-induced rise of bilirubin in septic shock, was not proven.

**References**

1-Journal of Intensive Care Medicine 2015, Vol. 30(1) 23–29;

2-Thorax 2009;64:784–790;

3-Intensive Care Med (2016) 42:16–27;

4-Chest 1992;101:1644–55.

#### A589 Prevalence of euthyroid sick syndrome in patients with sepsis and its correlation with clinical severity variables

##### C.M. Coronado Robles, M.A. Montes de Oca-Sandoval, A. Sánchez-Rodríguez, J.G. Joya-Galeana, A. Correa-Morales, G. Camarena-Alejo, J. Aguirre-Sánchez, J. Franco-Granillo

###### Universidad Autonoma de México, Ciudad de México, Mexico

####### **Correspondence:** C.M. Coronado Robles - Universidad Autonoma de México, Ciudad de México, Mexico

**Introduction:** While the prevalence of euthyroid sick syndrome is known, which is present in approximately 60 % to 70 % of septic patients in an ICU unit, there is little information on their correlation with other clinical severity variables.

**Objectives:** To estimate the prevalence of euthyroid sick syndrome and correlate the findings of thyroid hormone profile with clinical severity variables.

**Methods:** During the period of March 2014 to February 2016, in an ICU unit ABC Medical Center in Mexico, were documented clinical and paraclinical findings including a thyroid hormone profile in septic patients of any etiology and severity criteria collected based on the scale of SOFA (sequential organ failure assessment).

**Results:** 51 patients were included with a mean age of 68 ± 15.6 years, 47 % females and 52.9 % males. BMI of 24.1 ± 4.3 kg/m2. The median ICU stay was 7 days (IQR 4–13) and post ICU stay of 1 (IQR 0–5). Invasive and non-invasive mechanical ventilation in 23.5 and 29.4 %, respectively. The overall mortality rate was 31.4 %. The SOFA average was 8.5 ± 3.8. PCR median 20.57 (IQR 12.9-28.17). PCT 5.24 (IQR 1.6- 42). The proportion of patients with a history of diabetes, cardiovascular and thyroid disease was 29.4, 64.7 and 21.6 %, respectively. The overall prevalence of euthyroid sick syndrome was 80.4 % (95 % CI 0.69 to 0.91 %). The correlations between thyroid hormone profile and clinical severity variables were: for T4T with Glasgow Coma Scale r = 0.33, (p = 0.017); for FT3 with days of hospital stay r = 0.8, (p = 0.028), T4T with SOFA r = −0.28, (p = 0.047); TSH with SOFA r = −0.3, (p = 0.031); FT4 with creatinine r = 0.4, (p = 0.018). Total protein with FT3 and T4T with r = 0.3, (p = 0.034) and r = 0.308, (p = 0.029), respectively. PCR with FT3 r = 0.84, (p = 0.017). The group of patients with euthyroid sick syndrome spent more days in the ICU 8 (IQR 4–16) vs 5 (IQR 3–7), without reaching statistical significance (p = 0.06).

**Conclusions:** We analyzed abnormalities in thyroid function tests and severity scales in septic patients in an intensive care unit, where prevalence of euthyroid sick syndrome was 80.4 %. A lower value of TSH and T4T correlated with the severity of patients. A lower Glasgow Coma Scale correlated with lower value of T4T. Euthyroid sick syndrome has a tendency to stay longer in the intensive care unit. Key words: Critical illness, Euthyroid sick syndrome.

**References**

1. Peeters rP, Van der Geyten S, Wouters PJ, et al., 2005 Tissue thyroid hormone levels in critical illness. J Clin Endocrinol Metab 90: 6498–6507.

2. Foteini E, Evangelia D, Marinella T, Serafeim N, Anastasia K. Thyroid function during critical illness. Hormones 2011, 10(2):117–124

#### A590 Evaluating the effect of sepsis & septic shock on myocardial functions by echocardiography & serum biomarkers level in peripheral veins & coronary sinus

##### M. Soliman^1^, A. Al Azab^1^, R. El Hossainy^1^, H. Nagy^1^, M. Nirmalan^2^

###### ^1^Cairo University, Critical Care, Cairo, Egypt; ^2^University of Manchester, Critical Care, Manchester, UK

####### **Correspondence:** M. Soliman - Cairo University, Critical Care, Cairo, Egypt

**Introduction:** Sepsis is a leading cause of death in critically ill patients despite the use of modern antibiotics and resuscitation therapies,Biomarkers & Cardiovascular changes have an important place in this process, myocardial depression occur in 40 % of septic patients.

**Objectives:** Assessment of myocardial dysfunction in sepsis & septic shock by echocardiography parameters & biomarkers.

**Methods:** 20 patients (group I) with sepsis or septic shock were included &10 patients (group II) were served as non-septic group.

Group I, Morbidity & mortality at the day 28 in ICU were targeted as end point. laboratory investigations, APACHEIV, SAPS II, SOFA scores were calculated. Biomarkers IL-1α, IL-1β, IL-6, IL-10, TNFα, CRP, NT-proBNP & Troponin level were estimated on admission & day 7 in peripheral vein (PV) &coronary sinus (CS).Trans-Thoracic Echocardiography (TTE) & Tissue Doppler Imaging (TDI) were done on admission & on day 7.

**Results:** Comparing group I Vs group II,Mortality rate was 45 %,there was a statistically significant difference for temperature (p = 0.001), HR (p = 0.001) & WBC count (p = 0.01) on admission. then upon comparing survivors Vs non-survivors in group I there was a statistical difference in HR on day 7 (p = 0.02), successful Vasopressors withdrawal (p = 0.02). P/F ratio (p = 0.02) & ScVO2 on day 7 (p = 0.03) .Regarding IL-1α, IL-1β, TNF-α & Troponin I there was no statistical significant difference between group I & II but IL-6, IL-10 & CRP showed statistically significant difference on admission PV & CS. Pro-BNP shows statistically significant difference in all CS samples either between septic & non-septic groups. Regarding ECHO upon comparing the survivors Vs non-survivors E`d/t on day 0 shows a statistically significant difference between both groups, SAPS II & 7th day SOFA are good predictive scores to mortality in sepsis.

**Conclusions:** Diastolic dysfunction was seen in 90 % of patients. fever, HR, WBC counts still good early indicators for diagnosis of sepsis.Vasopressors withdrawal on 7th day was good predictor for survival. Admission serums IL-6, IL-10 & CRP from PV were better indicators for Sepsis than IL-1, Pro-BNP & Troponin I.admission TNF-α & 7th day IL-6 levels were highly prognostic to mortality.CS samples proved that NT Pro-BNP is a good indicator for sepsis diagnosis & a good predictor for survival,TNF-α from CS samples was also a good predictor for mortality. SAPS II .A slower E`d/t on admission was good predictor to mortality.

**Note:** This abstract has been previously published and is available at [2]. It is included here as a complete record of the abstracts from the conference.

**References**

1. Angus DC,Epidemiology of severe sepsis in USA:Crit Care Med 2001,29:1303–1310.

2. Soliman M, Alazab A, El Hossainy R, Nirmalan M, Nagy H (2015) Evaluating the effect of sepsis and septic shock on myocardial functions by echocardiography and serum biomarker level in peripheral veins and coronary sinus. Critical Care 19(Suppl 1): P136

#### A591 Cerebral autoregulation during septic shock

##### I.A. Crippa, F. Zama Cavicchi, J.-L. Vincent, J. Creteur, F.S. Taccone

###### Université Libre de Bruxelles (ULB) - Hôpital Erasme, Brussels, Belgium

####### **Correspondence:** I.A. Crippa - Université Libre de Bruxelles (ULB) - Hôpital Erasme, Brussels, Belgium

**Introduction:** Sepsis-associated encephalopathy (SAE) is associated with increased morbidity and mortality in septic patients. The pathophysiology of SAE is incompletely elucidated. It has been hypothesized that impairment of cerebral autoregulation (CAR) would result in brain hypoperfusion and neuronal damage (1), but previous studies showed conflicting results (2,3).

**Objectives:** The aim of the study is to evaluate CAR in septic shock patients and test the hypothesis that its alteration is associated with clinical encephalopathy or brain perfusion.

**Methods:** Adult patients diagnosed with septic shock and admitted to the ICU stay were included (July 2015 - March 2016). Exclusion criteria were: intracranial disease; arrythmias; extra-corporeal membrane oxygenation; intra/extra-cranial arteriopathy. Transcranial Doppler (DWL, Germany) was performed insonating left middle cerebral artery (LMCA) with a 2-MHz probe. LMCA blood flow velocity (FV) and arterial blood pressure (BP) signals were simultaneously recorded; Pearson´s correlation coefficient between BP and FV (Mxa) was calculated using MATLAB (MathWorks, USA). Impaired CAR was defined as Mxa > 0.3 (4).

**Results:** 24 patients were included; median norepinephrine dose was 0.3 [0.13-0.66] mcg/kg/min. Site of infection was mainly pulmonary (4/24,17 %) and hematogenous (4,17 %). Arterial PCO2 was 35 [32–43] mmHg, pH was 7.37 [7.35-7.43], central venous oxygen saturation was 71 [62–73]% at time of TCD assessment. Eight patients (33 %) received sedation during examination. Median Mxa was 0.26 [−0.06-0.6] and CAR was impaired in 11 patients (46 %). No difference in Mxa was observed between sedated and non-sedated patients (p = 0.3), PaCO2 level < vs ≥ 40 mmHg (p = 0.9) and presence of encephalopathy (e.g. GCS < 15 on admission) (p = 0.5). Also, no association between Mxa and mean BP or dose of vasopressors was noted.

**Conclusions:** CAR was altered in almost half of the patients with septic shock. No association between autoregulation and clinical encephalopathy or determinants of brain perfusion was found (Table 38).

**References**

1. Taccone FS et al.Brain perfusion in sepsis.Curr Vasc Pharm 2013;11:170–86

2. Schramm P et al.Impaired cerebrovascular autoregulation in patients with severe sepsis and sepsis-associated delirium.Crit Care 2012;16:R181

3. Matta BF et al. Sepsis-induced vasoparalysis does not involve the cerebral vasculature: indirect evidence from autoregulation and carbon dioxide reactivity studies. Br J Anesthesia 1996;76:790–4

4. Czosnyka M et al.Monitoring of Cerebral Autoregulation in Head-Injured Patients.Stroke;1996:1829–34

**Table 38 (abstract A591).** Population characteristics (number (%), median [IQR])

Male 21 (87%)	
Age (years) 59 [50-67]	
Coronary artery disease 4 (17%)	
Apache II score 18 [13-25]	
ICU LOS (days) 8 [5-16]	

#### A592 Pancreatic injury in patients with septic shock: epidemiology and prognosis impact

##### A. Chaari^1^, K. Abdel Hakim^2^, H. Hassanein^2^, M. Etman^2^, M. El Bahr^2^, K. Bousselmi^2^, E.S. Khalil^2^, V. Kauts^2^, W.F. Casey^2^

###### ^1^King Hamad University Hospital, Intensive Care, Muharaq, Bahrain; ^2^King Hamad University Hospital, Muharaq, Bahrain

####### **Correspondence:** A. Chaari - King Hamad University Hospital, Intensive Care, Muharaq, Bahrain

**Introduction:** Septic shock is a life threatening condition associated with high mortality. Poor outcome is mainly related to the inadequacy between oxygen delivery and cellular demand leading to the onset of multiorgan dysfunction. Whether this multiorgan failure affect the pancreas is not fully investigated [1].

**Objective:** To assess the prognostic value of increased pancreatic enzymes in patients with septic shock.

**Methods:** We conducted a prospective study in a 12-bed medical surgical intensive care unit. All patients admitted with septic shock were included. Demographic, clinical and biological data were recorded on admission. Serum amylase and lipase levels were checked on day 1 and day 2. All the patients with increased amylase and/or lipase serum level underwent imaging investigations. Quantitative variables were expressed as median [quartiles] and qualitative variables were expressed as percentages. Quantitative variables were compared by Mann–Whitney test and qualitative variables were compared by Chi2 or Fisher exact test as appropriate.

**Results:** Thirty patients were included in our study. Median [quartile] age was 68 [58–81] years. Sex-ratio (M/F) was 1.7. Median [quartile] APACHE II score was 26.5 [19.8-32]. The cause of septic shock was pneumonia in 14 patients (46.7 %), urinary tract infection in 4 patients (13.3 %), necrotizing fasciitis in 7 patients (23.3 %) and intra-abdominal sepsis in 5 patients (16.7 %). Median [quartile] serum amylase was 44.3 [25.6-76.4] UI/L on day 1 and 49 [25.4-177] on day 2. Median [quartile] serum lipase was 132 [74–258.5] UI/L on day 1 and 142 [73–261] UI/L on day 2. Twelve patients (40 %) had increased amylase level and seven patients (23.3 %) had increased lipase level. All these patients underwent imaging tests: Three patients (10 %) had abdominal computed tomography and 9 patients (30) had abdominal ultrasonography. Only one patient (3.3 %) had radiological evidence of acute pancreatitis. Median [quartile] length of intensive care unit stay was 6 [3–11] Days. 28-day mortality was 36.7 %. There was no significant difference between survivors and deaths regarding the highest serum lipase and amylase levels recorded during the first 48 hours (respectively 90 [22–153] versus 46 [31–201]; p = 0.914 and 117 [65–255] versus 176 [116–295]; p = 0.197). Non-survivors at 28-day had higher APACHE II score (28 [27–32] versus 21[19–29]; p = 0.024) and required mechanical ventilation more frequently (100 % versus 42.1 %; p = 0.002).

**Conclusion:** The increase of pancreatic enzyme is common in patients with septic shock. However, pancreatic injury does not affect the prognosis of these patients.

**References**

1. Denz C, Siegel L, Lehmann KJ, Dagorn JC, Fiedler F: Is hyperlipasemia in critically ill patients of clinical importance? An observational CT study. Intensive care Med 2007, 33(9):1633–1636.

#### A593 The worse prognostic factor in septic patients: acute kidney injury and acute respiratory failure

##### H. Imahase, Y. Sakamoto, S. Inoue, K.C. Yamada, H. Koami, T. Miike, F. Nagashima, T. Iwamura

###### Saga University Hospital, Saga City, Japan

####### **Correspondence:** H. Imahase - Saga University Hospital, Saga City, Japan

**Introduction:** The survival rate of patients with sepsis is improving(1, 2).

In our center, although promoting the standardization and individualization of sepsis treatment, some patients die. We examined some factors of the patients with sepsis.

**Objectives:** We searched the worse factor of the prognosis of septic patients.

**Methods:** This study was a retrospective observational study in a single center.

We studied the patients with sepsis who admitted from the emergency department to the ICU in our hospital. We defined sepsis as patients with SOFA score 2 points worse than sepsis onset before (Sepsis-3 Definition) (3).

The study period was from Jan. 2014 to Jun. 2015.

Gender, age, APACHE2, SOFA, and KDIGO classification of AKI were checked.

**Results:** The number of patients was 60: non-survivor 18(30 %), and survivor 42(70 %).

Table 39 showed the characteristics of each group. Table 40 showed the correlation between KDIGO classification and the prognosis.

As APACHE2, SOFA, and KDIGO classification of AKI were bad, life prognosis was bad.

Among SOFA score, renal function and respiratory function were well correlated with patients mortality.

**Conclusions:** The acute kidney injury and acute respiratory failure were especially worse prognostic factor among septic patients.

**References**

1) Two Decades of Mortality Trends Among Patients With Severe Sepsis: A Comparative Meta-Analysis. Crit Care Med. 2014; 42(3): 625–631

2) Mortality Related to Severe Sepsis and Septic Shock Among Critically Ill patients in Australia and New Zealand, 2000–2012. JAMA 2014; 311(13): 1308–1316

3) The Third International Consensus Definitions for Sepsis and Septic Shock (Sepsis-3). JAMA. 2016 Feb.23; 315(8): 801–10

**Table 39 (abstract A593).** Patients characteristics

	Non-survive 18 cases	Survive 42 cases	
Gender Male : Female	10 : 8	19 : 23	
Age	70.9 ± 11.0	73.1 ± 13.7	p = 0.543
APACHE2	29.2 ± 8.5	17.8 ± 7.2	p < 0.01
SOFA score	12.4 ± 2.9	6.9 ± 3.5	p < 0.01

**Table 40 (abstract A593).** KDIGO classsification and the prognosis

KDIGO classification of AKI	Non-survive	Survive	Mortality (%)
non-AKI	2	22	8.3
1	7	13	35
2	4	4	50
3	3	2	60
on HD	2	1	66.7

#### A594 Preliminary findings on thromboelastometry profiles in septic patients admitted to intensive care unit

##### A. Boscolo^1^, V. Lucchetta^1^, E. Piasentini^1^, D. Bertini^1^, L. Manesso^1^, L. Spiezia^2^, P. Simioni^2^, C. Ori^3^

###### ^1^UOC Anesthesia and Intensive Care Unit, Hospital of Padova, Padova, Italy; ^2^Thrombotic and Hemorrhagic Diseases Unit, Department of Medicine, University of Padua, Padova, Italy; ^3^UOC Anesthesia and Intensive Care Unit, Department of Medicine-DIMED, Padova, Italy

####### **Correspondence:** A. Boscolo - UOC Anesthesia and Intensive Care Unit, Hospital of Padova, Padova, Italy

**Introduction:** Coagulopathy in septic shock(SS) is characterized by an imbalance between pro and anticoagulants.The coagulation cascade is over-activated,causing a consumption of clotting factors until DIC.This extreme condition is associated to a higher mortality.ROTEM®, a point-of-care assay based on viscoelastic measurements of coagulation changes in whole blood,could be useful to study the coagulative profiles in patients at risk of DIC[1]. However, the use of ROTEM®for the management of SS has not been validated,yet.

**Objectives:** The aim of this study was to evaluate the possible role of ROTEM®as a diagnostic and prognostic tool in SS.In particular,we assessed its ability to identify critically ill-patients at high risk of DIC.

**Methods:** 21patients(43%M,57%F)admitted to our ICU with a diagnosis of SS[2] were enrolled.Exclusion criteria were:end-stage heart failure and liver disease,severe cancer, age < 18y, pre-existent hematological disorders,ongoing anticoagulation and antiplatelet therapy. 21 healthy volunteers, age and sex matched, were recruited to establish references values for ROTEM®parameters.ROTEM®assays were performed at the time of ICU admission(baseline) and 24 h later(day1). ROTEM®(Tem International GmbH, München, Germany) was performed according to manufacturers'recommendations. The DIC-score, calculated according to the International Society for Thrombosis and Haemostasis, was determined over the first 24 h and 28-day mortality was recorded. Data were expressed as medians, IQR or percentages. T-test was used for comparison between groups. ROC curves and the correspondent AUC were used to assess viscoelastic parameters as diagnostic biomarkers of SS and DIC.

**Results:** In SS EXTEM-clotting time(CT) was significantly longer both at baseline and day 1[81.79 s(IQR64-88) and 73s(IQR66-77), respectively], compared to controls[60s(IQR45-64),p < 0.01)]. Furthermore, EXTEM-CT showed a great ability to identify septic patients(CT:AUC > 0.83,p < 0.01). No differences were noticed between survivors and non-survivors. ISTH-score for DIC was ≥ 5 in 24 % of patients, and their MCF in INTEM, EXTEM and FIBTEM was strongly reduced[DIC vs no-DIC:48 mm (IQR47-52) vs 68 mm (IQR65-73), 49 mm (IQR49-49) vs 69 mm (IQR66-74) and 7 mm (IQR4-11) vs 30 mm (IQR20-38) respectively, p < 0.05). The relative ROC-curves showed that MCF could be a valid biomarker for diagnosis of DIC(AUC > 0.95,p < 0.01 in INTEM, EXTEM, FIBTEM).

**Conclusion:** Our preliminary findings suggest that ROTEM®could be a valid tool for diagnosis of coagulopathy in SS.EXTEM-CT measurement underlined a severe impairment in the extrinsic coagulation pathway.MCF in INTEM,EXTEM and FIBTEM seemed to be useful biomarkers for the diagnosis of DIC.In conclusion,ROTEM®might have an additional value to identify coagulopathy in SS.

**References**

1. Muller MC et al. Utility of TEG and/or ROTEM in adults with sepsis:a systemic review.Crit Care´14;18:R30.

2. Singer M et al. The 3^rd^ international consesus definitions of sepsis and septic shock(SEPSIS3).Jama´16;315(8):801–10.

#### A595 Morphological alterations of liver in sepsis survivors. An experimental study

##### R.B. Souza^1^, A.M. Martins^2^, A.M.A. Liberatore^3^, Y.R. Kang^3^, M.N. Nakamae^3^, J.C.F. Vieira^3^, I.H.J. Koh^3^

###### ^1^Federal University of São Paulo, Morphology and Genetics, São Paulo, Brazil; ^2^Biological Institute of São Paulo, São Paulo, Brazil; ^3^Federal University of São Paulo, Surgery, São Paulo, Brazil

####### **Correspondence:** R.B. Souza - Federal University of São Paulo, Morphology and Genetics, São Paulo, Brazil

Hepatic dysfunction represents a common manifestation during the sepsis process, involving portal inflammation, centrilobular necrosis, lobular inflammation, hepatocellular apoptosis, steatosis and cholangitis. The splanchnic hypoperfusion and the subsequent microcirculation injuries are causative factors among other multiple factors leading to the hepatic dysfunction in sepsis. However, how much of the liver dysfunction is recovered after discharge from hospital is still little known. Herein we evaluated the impact of sepsis on liver structures in sepsis survivals.

**Methods:** Adult Wistar rats (200 g) (n = 12) were submitted to sepsis [iv. 2 mL *E. coli* 10^9^ CFU/ mL (S9), DL80 in 26 hours]. After 2 h, 6 h, 24 h and 30 days, (T2h, T6h, T24h and T30d), the animals were sacrificed, and samples of liver were collected and stained with H&E, PAS and Sudan-Black. The T30d animals were considered survivors since the mortality of sepsis S9 is 80 % from 26 hours after induction

**Results:** The histological results show that S-9 sepsis model determines a rapid and progressive liver damage. In the period of T2h, although predominant hepatic aspect of normality was observed, there was the onset of a sinusoidal congestion of focal occurrence, presence of polymorphonuclear cells in the lumen of the vein hepatic centrilobular and by PAS staining was observed the glycogen accumulation in cytoplasm of hepatocytes, except around centrilobular vein. At T6H, there was a generalized sinusoidal congestion, significant polymorphonuclear cells infiltration in the portal area and presence of leukocytes adhering to the endothelium of the centrilobular vein. Besides, was also observed some hepatocytes with shrunken nucleus suggesting apoptotic cell death in progress. PAS showed a overall decrease of glycogen in hepatocytes, suggesting a high consumption of energy due to the exacerbated inflammatory process of sepsis. Twenty four hours post sepsis, the liver was congested, mainly in centrilobular veins, suggestive of increased vascular resistance in the liver. The infiltration of polymorphonuclear cells, predominantly in centrilobular and periportal areas suggested the leucocyte invasion into the liver parenchyma and subsequent proinflammatory reaction. PAS staining showed the presence of multiple small vesicles inside the hepatocytes, suggestive of initial degenerative process in progress and this proved to be fat vesicles by Sudan Black staining. In 30 days after sepsis recovery, the sinusoidal spaces were compressed and some congestions were located in the centrilobular vein, moreover, there were liver cell degeneration with pycnosis and hyaline degeneration, suggesting a chronic phase of liver dysfunction. The overall findings demonstrated a multiple liver parenchymal abnormalities in post-sepsis period, suggesting the fragility of the liver physiology to fight against a new infectious challenge.

**Grant acknowledgement**

FAPESP 2011/20401-4.

**Fig. 64 (abstract A595).**
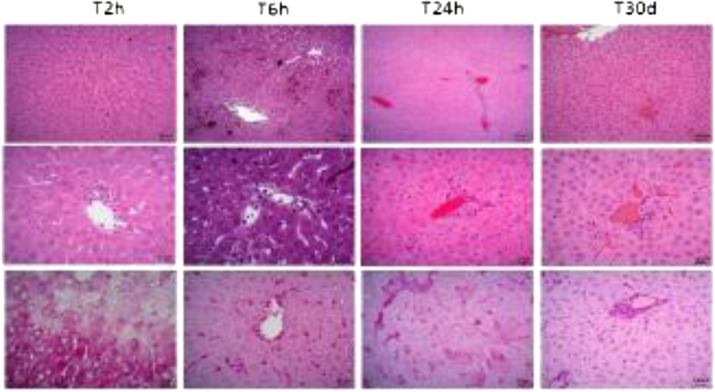
Kinetics of hepatic lesions of the acute phase (T2h-T24h) and post-sepsis recovery (T30d)

#### A596 Trans-splanchnic bacterial clearance is not influenced by pre-existing systemic inflammation in porcine septic shock

##### K. Hanslin^1^, F. Wilske^2^, P. Skorup^2^, J. Sjölin^2^, M. Lipcsey^1^

###### ^1^Uppsala University, Department of Surgical Sciences, Uppsala, Sweden; ^2^Uppsala University, Department of Medical Sciences, Uppsala, Sweden

####### **Correspondence:** K. Hanslin - Uppsala University, Department of Surgical Sciences, Uppsala, Sweden

**Introduction:** The splanchnic reticulo-endothelial system is fundamentally involved in bacterial clearance both via innate and adaptive immune responses. It has e.g. been reported that hyposplenism is associated with an unfavorable outcome in sepsis. We hypothesized that systemic inflammatory response (SIR) induced immunosuppression leads to decreased trans-splanchnic microbial clearance.

**Objectives:** To investigate if pre-existing systemic inflammation affects the splanchnic bacterial clearance.

**Methods:** Fifteen anesthetized pigs were subjected to an infusion of *E. coli* in a cervical vein for 3 hours. To induce a systemic inflammatory response, group “EtxPreExp” (n = 6) received a continuous intravenous endotoxin infusion starting 24 h prior to the *E. coli* infusion. Group “UnExp” (n = 6) received the bacterial infusion without prior endotoxin exposure. To understand the effects of 24 h anesthesia alone, three animals received saline instead of endotoxin for 24 h prior to the bacterial infusion (“Controls”). According to the predefined statistical plan, “Controls” were not included in the primary analysis. Bacterial counts in arterial and portal venous blood were analyzed hourly during the *E. coli* infusion, and were corrected for the bacterial dose administered per body weight. Blood samples were analyzed and physiologic parameters were recorded throughout the experiment.

**Fig. 65 (abstract A596).**
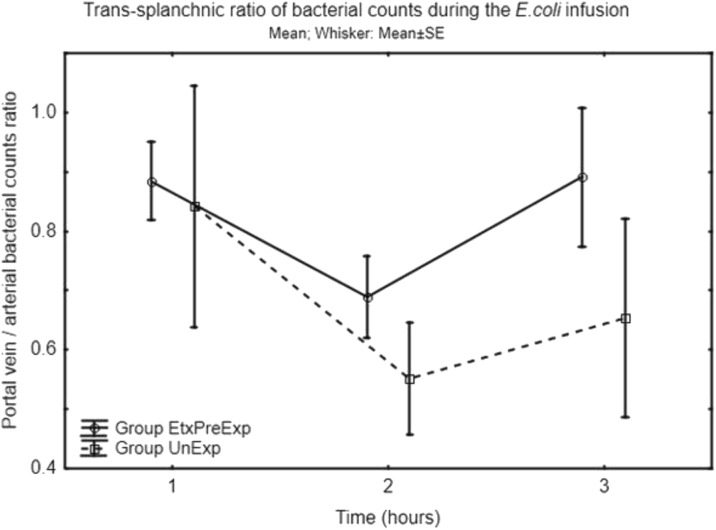
Trans-splanchnic ratio of bacterial counts

**Results:** All animals receiving endotoxin developed SIR prior to the *E. coli* infusion. The amounts of administered bacteria were comparable between the groups. There were no differences between EtxPreExp and UnExp groups in log arterial (2.47 (±0.49) vs. 2.38 (±0.57) CFU x mL^−1^_corr_; mean ± SD) or portal (2.40 (±0.36) vs. 2.11 (±0.77)) bacterial counts at 3 h, just before the end of the bacterial infusion. Furthermore, there was no difference in the ratio of portal vein to arterial bacterial counts between the EtxPreExp and UnExp groups (Fig. 65). White blood cell counts (p < 0.001), mean arterial pressure (p < 0.01), base excess (p < 0.05) and pH (p < 0.001) were lower, while arterial lactate levels (p < 0.001) were higher in UnExp vs. EtxPreExp group during and after the *E. coli* infusion.

**Conclusions:** Our preliminary results show that despite diminished immunologic and physiologic response, induced by pre-existing inflammatory response, the splanchnic bacterial clearance is not affected in *E. coli* septic shock.

#### A597 Biliary tract external drainage protects against liver injuries of severe acute pancreatitis rats via heme oxygenase-1 pathway

##### W. Jin Long, C. Er Zhen

###### Ruijin Hospital, Shanghai Jiaotong University School of Medicine, Shanghai, China

####### **Correspondence:** W. Jin Long - Ruijin Hospital, Shanghai Jiaotong University School of Medicine, Shanghai, China

**Objective:** To investigate the effect of biliary tract external drainage (BTED) on liver of severe acute pancreatitis (SAP) rats and the relationship with heme oxygenase-1 (HO-1) pathway.

**Methods:** SD rats weighing 250–300 g were randomly assigned into five groups (n = 6): sham surgery (SS) group, SAP group, SAP + BTED group, SAP+ zinc protoporphyrin IX (ZnPP) group, SAP + BTED + ZnPP group. The SAP model was induced via retrograde injection of 4 % sodium taurocholate (1 mL/kg) into biliopancreatic duct through duodenalwall. BTED was performed by inserting a cannula into the bile duct of SAP rats. Tissue and blood samples were collected 24 hours after surgery. Pathological changes in organs were scored. The level of amylase, alanine transaminase(ALT), aspartate aminotransferase (AST) in serum were measured. The expression of hemeoxygenase-1 (HO-1), tumor necrosis factor-α (TNF-α), and interleukin-6 (IL-6) in tissues were analyzed by real-time PCR and western-blot.

**Results:** Liver damage in SAP rats was significantly alleviated by BTED (p < 0.05). Compared to the SAP group, the serum level of amylase, ALT, AST, were significantly lower in the BTED group (p < 0.05). The BETD treatment led to a significant reduction of TNF-α, IL-6 level and a significant increase of HO-1 level in tissues than in SAP rat (p < 0.05). ZnPP significantly inhibited all mentioned effects.

**Conclusions:** BTED protected liver against SAP related injuries via the HO-1 pathway.

#### A598 Correlation between percentages of ventilated patients developed vap and nursing, severity and outcome indexes in icu patients

##### A. Vakalos, V. Avramidis

###### Xanthi General Hospital, ICU, Xanthi, Greece

####### **Correspondence:** A. Vakalos - Xanthi General Hospital, ICU, Xanthi, Greece

**Introduction:** Ventilator Associated Pneumonia (VAP) is one of the most frequently seen infections in ICU setting and may have an impact not only to the length of ICU stay, but o ICU outcome as well.

**Objectives:** The aim of our observation retrospective study was to test the hypothesis that a correlation exists between percentage of ventilated patients developed VAP (% VP) and the main nursing, severity and outcome indexes in our both medical and surgical ICU served in community hospital.

**Methods:** From January 2006 to June 2014 admitted to our ICU 620 patients, mean age 64.8 years, mean length of ICU stay (LOS) 14.2 days, mean mechanical ventilation duration per ventilated patient (V. Days) 12.23 days, mean APACHE II score on addition 21.2, predicted mortality 38.9 %, actual mortality 31.45 %, Standardized Mortality Ratio (SMR) 0.80. From our database we looked for between percentage of ventilated patients developed VAP (% PV VAP) as well as the above nursing and severity indexes per year from 2006 to 2013 and per six months period for the latest year 2014. Using linear correlation method, we looked for linear slope, correlation coefficient (r), and coefficient of determination (r^2^), and by linear regression method using ANOVA test we looked for p value, according % PV VAP and nursing (LOS, VD), severity (age, APACHE, Predicted mortality) and outcome (Actual mortality, SMR) indexes.

**Results**

**Conclusions:** According to our data, there was no statistically significant correlation detected between percentage of ventilated patients developed VAP and Age, LOS, APACHE II score, Predicted Mortality, Actual Mortality and Standardized Mortality Ratio. On the other hand, we detected statistical significant positive, moderate correlation between percentage of ventilated patients developed VAP and Ventilation Days. Our data suggest that the more the ventilation days, the more the percentage of ventilated patients developed VAP increases.

**Table 41 (abstract A598).** Correlation between percentages of ventilated pati

	Slope	St Error	r	r2	L. CI	U. CI	p value
Age	−0.0990	0.519	−0.064	0.004	−1.161	1.161	0.8685
LOS	0.020	0.191	0.051	0.002	−0.427	0.480	0.8953
V. Days	0.354	0.130	0.716	0.513	0.045	0.663	0.0300
APACHE	0.329	0.575	0.315	0.099	−0.557	1.217	0.4084
Pr Mortality	0.969	1.112	0.313	0.097	−1.661	3.601	0.4126
Act. Mortality	1.931	1.001	0.589	0.347	−0.437	4.300	0.0951
SMR	0.029	0.018	0.523	0.274	−0.013	0.072	0.1479

### MECHANICAL VENTILATION: MANAGEMENT AND MONITORING

#### A599 Better airway resistances reduction profile in intubated copd patients by personalized bronchodilator dosing: a randomized control trial

##### S.-H. Wu^1^, L.-J. Shyu^2^, C.-H. Li^1^, C.-H. Yu^1^, H.-C. Chen^3^, C.-H. Wang^1^, K.-H. Lin^1^

###### ^1^Changhua Christian Hospital, Medicine, Changhua, Taiwan, Province of China; ^2^Changhua Christian Hospital, Pharmacy, Changhua, Taiwan, Province of China; ^3^Yuanlin Christian Hospital, Changhua, Taiwan, Province of China

####### **Correspondence:** S.-H. Wu - Changhua Christian Hospital, Medicine, Changhua, Taiwan, Province of China

**Introduction:** Metered dose bronchodilator inhalation through ventilator circuit has long been used in intubated patients of chronic obstructive pulmonary disease (COPD). However, fixed drug dosage now commonly used has not been endorsed by manufacturers nor proved clinically effective [1]. Evidences suggest drug delivered this way has suboptimal lung deposition [2]. Also, great individual variations among patients make them imprecise and unreliable[3].

**Objectives:** We proposed an experimental bronchodilator dosing schedule based on each individual's airway resistance (R_aw_) monitored periodically. This study tests its efficacy in R_aw_ reduction.

**Methods:** Fifty-one just-admitted, invasively ventilated COPD patients were randomly assigned to receive either personalized or fixed bronchodilator dosing. Personal target R_aw_ was defined by measuring each one's R_aw_ after maximally broncho-dilated by consecutive 4, 8 and 16 puffs of fenoterol metered dose inhaler (100 mcg). Thereafter, R_aw_ was measured every 8 hours until the 28th day. Patients of the fixed dose group received only predetermined dose. Additional doses of bronchodilator were given to patients of the personalized dosing group when measured R_aw_ exceeded patient's target R_aw_. Both groups received rescue short-acting bronchodilator when dyspnea exacerbated.

**Results:** The relative deviation of R_aw_ from its personal target was expressed as (measured R_aw_ - target R_aw_)/target R_aw_. Experimental group has a smaller relative R_aw_ deviation than control group (0.09 ± 0.10 vs 0.44 ± 0.11, *P* = 0.022). In a multivariate linear regression model analysis, R_aw_ of experimental group tended to become closer to its personal target over time (−0.031 point of relative deviation every day). Whereas that tendency was deviation away from its target over time in control group (0.004 point of relative deviation every day) (Fig). The two groups did not differ significantly in ventilator-free days from day 1 to 28, percentage of breathing without assistance by day 28, episode of nosocomial pneumonia, total puffs of rescue bronchodilator, number of drug-related adverse effect or mortality rate at day 180.

**Conclusions:** Personalized dosing of bronchodilator inhalation to invasively ventilated COPD patients produce a better R_aw_ reduction profile than fixed dosing. The conclusions of this pilot study need consolidation by further investigations.

**References**

1. Dhand, R., Respir Care, 2005. **50**(10): p. 1331–4.

2. Fuller, H.D., et al., Am Rev Respir Dis, 1990. **141**(2): p. 440–4.

3. Malliotakis, P., et al., Crit Care, 2008. **12**(6): p. R140.

**Grant acknowledgement**

The study was supported by grant number 102-CCH-IRP-017 (Changhua Christian Hospital, Taiwan, R.O.C).

#### A600 Retrospective analysis of respiratory mechanics in ARDS: driving pressure and outcome

##### Z.E. Aray, C.F. Gómez, A.P. Tejero, D.D. Monge, V.M. Losada, C.M. Tarancón, S.D. Cortés, A.M. Gutiérrez, T.P. Álvarez

###### Hospital Virgen de la Concha, Medicina Intensiva, Zamora, Spain

####### **Correspondence:** Z.E. Aray - Hospital Virgen de la Concha, Medicina Intensiva, Zamora, Spain

**Introduction:** In severe Acute Respiratory Distress Syndrome, there are important structural pulmonary changes, deterioration of the oxygen permeability, residual pulmonary capacity, pulmonary compliance, airway resistance and recruitability. The use of lower plateau pressures, lower tidal volumes and higher PEEPs can improve survival.

**Objective:** Review our experience in the management and evolution of patients with Severe ARDS during 3 years in a second level hospital. In our analysis we took into account the treatment approach, mode of ventilation, PEEPs, the resistance and compliance values reported by the ventilator and recorded automatically.

**Methods:** A retrospective study of ICU patients requiring mechanical ventilation between 2013–2015 at Hospital Virgen de la Concha(Zamora, Spain) due to severe ARDS. 23 patients admitted during this period have a deterioration of PaO2/FiO2 to levels lower than or equal to 100 during a 24 hours period and requiring at least 5 days of ICU admission. Epidemiological data, presumed cause of ARDS, management (steroids, neuromuscular blocking agents, inhaled beta-agonists), mechanical ventilation parameters (maximum PEEP,resistance,compliance,driving pressure) were analysed. We divided arbitrarily between HOR (high concentration of oxygen required, greater than or equal to 60 %) and LOR period (low oxygen concentration required, less than 60 %).

**Results:** The overall severe ARDS mortality was of 56.52 %. The pronation strategy was carried out in 39.13 % of the patients. The median ICU stay, mechanical ventilation and high oxygen requirement (HOR) duration was of 24.47; 16.6 and 8.43 days. The maximum PEEP used in this group of patients was 17 cmH20; with a median value of 13.2 cmH20. The pulmonary resistance, compliance and driving pressure (DP) in the non-surviving group were (19.21 cmH20/l/sec; 45.39 ml/cmH20; 14.20 cmH20), in the surviving group were (15.38 cmH20/l/sec; 47.82 ml/cmH20; 12.10 cmH20). The mortality in the group with DP higher than 15 during HOR was 100 %, being 33.3 % in the group with lower DP values (p < 0.05).

**Conclusions:** We found that non-surviving patients have higher resistance and lower compliance values in their respiratory system and the improvement of its values were associated with better clinical outcome.The driving pressure was the variable that best stratified the surviving and non-surviving patients.

**References**

1. Amato M, Meade M, Slutsky A. Driving pressure and Survival in the Acute Respiratory Distress Syndrome N ENGL J MED 2015; 372:747–55

2. Villar J, Kacmarek RM, Pérez-Méndez L, Aguirre_Jaime. A high positive end expiratory pressure, low tidal volume ventilatory strategy improves outcome in persistent acute respiratory distress syndrome: a randomized, controlled trial. Crit Care Med 2006; 34:1311–8.

3. Protti A, Andreis DT, Monti M, et al. Lung stress and strain during mechanical ventilation: any difference between statics and dynamics? Crit Care Med 2013; 41:1046–55.

#### A601 Impact of COPD on the incidence and outcome of ventilator-associated conditions

##### A. Rouze, K. Jaffal, S. Six, K. Stolz, V. Cattoen, S. Nseir

###### Critical Care Center, University Hospital of Lille, Lille, France

####### **Correspondence:** A. Rouze - Critical Care Center, University Hospital of Lille, Lille, France

**Introduction:** In 2013, the Centers for Disease Control and Prevention (CDC) published a new surveillance paradigm for complications of mechanical ventilation (MV), in response to difficult surveillance of ventilator-associated pneumonia (VAP) in critically ill patients (1). No study has investigated the newly defined ventilator-associated conditions (VAC) and infection-related ventilator-associated complications (IVAC) among COPD population.

**Objectives:** Our study aims to determine the impact of COPD on the incidence and outcome of VAC and IVAC, and their relationship with VAP.

**Methods:** Retrospective analyse of prospectively collected data from patients requiring MV for more than 48 hours[SN1] . VAP episodes were assessed by prospective surveillance of nosocomial infections according to ATS/IDSA guidelines (2). VAC and IVAC were identified retrospectively according to current CDC definitions (1). We assessed VAC, IVAC, and VAP rates, and their impact on outcome, according to the presence or the absence of underlying COPD. The relationship between VAC, IVAC and VAP episodes was also assessed in COPD and non COPD patients. Qualitative and quantitative variables were compared using chi-square test and Mann–Whitney *U* test, respectively.

**Results:** Data from 472 patients (132 with COPD and 340 without COPD) were analyzed. Similar rates of VAC (43.2 vs 36.8 %, p = 0.199), IVAC (22.0 vs 16.8 %, p = 0.189) and VAP (28.8 vs 28.5 %, p = 0.956) were found in COPD and non COPD patients, respectively. Agreement between VAC or IVAC and VAP episodes was 21.1 % and 15.8 % respectively among COPD patients, 21.6 % and 33.3 % among patients without COPD. No significant difference was found in outcome of patients with VAC and IVAC between COPD and non COPD patients, unlike patients who developed VAP (Table 42).

**Conclusions:** Incidence and outcome of VAC and IVAC were similar in COPD and non COPD patients. Agreement between VAC or IVAC and VAP episodes was poor in the two groups.

**References**

1. Magill SS, Klompas M, Balk R, Burns SM, Deutschman CS, Diekema D, et al. Developing a new, national approach to surveillance for ventilator-associated events. Crit Care Med 2013;41(11):2467-75.

2. American Thoracic Society, Infectious Diseases Society of America. Guidelines for the management of adults with hospital-acquired, ventilator-associated, and healthcare-associated pneumonia. Am J Respir Crit Care Med 2005;171(4):388-416.

**Table 42 (abstract A601).** Outcome of patients with VAP, VAC and IVAC, in COP

	VAP (n=135)		VAC (n=182)		IVAC (n=86)	
	COPD(n=38)	No COPD (n=97)	COPD (n=57)	No COPD (n=125)	COPD (n=29)	No COPD (n=57)
MV duration (days)	38[21–53]	25[14–37]*	25[15–42]	23[12–35]	29[18–44]	29[12–41]
ICU LOS (days)	40[22–65]	29[18–42]*	31[20–46]	25[15–41]	37[20–50]	31[17–43]
ICU Mortality	22(58)	47(49)	26(46)	67(54)	12(41)	28(49)

Data are presented as number (%) or median (interquartile range)

*Abbreviations*: *MV* mechanical ventilation, *LOS* length of stay

*p<0.05, COPD versus non COPD patients

#### A602 Driving pressure automatically selected by INTELLiVENT-ASV in ICU patients

##### J.-M. Arnal^1,2^, M. Saoli^1^, D. Novotni^2^, A. Garnero^1^

###### ^1^Hopital Sainte Musse, Toulon, France; ^2^Hamilton Medical, Research and Development, Bonaduz, Switzerland

####### **Correspondence:** J.-M. Arnal - Hamilton Medical, Research and Development, Bonaduz, Switzerland

**Introduction:** Mechanical ventilation is associated with ventilation induced lung injuries (VILI) due to lung stress and lung strain. VILI can be prevented by limiting driving pressure (ΔP) (1), plateau pressure (P_PLAT_), and tidal volume (V_T_). INTELLiVENT-ASV is a full closed loop ventilation solution that combines a ventilation and an oxygenation controller (2). The ventilation controller adjust minute volume to reach a target end tidal CO2 set by the user. Adaptive Support Ventilation (ASV) algorithm determines for a given minute volume the respiratory rate (RR) - V_T_ combination associated with the minimal work of breathing (3). Recently, ASV algorithm has been modified in order to limit driving pressure (ΔP) (4).

**Objectives:** In order to determine if INTELLiVENT-ASV selects a lung protective ventilation, this prospective observational study reports on the applied ventilation parameters and blood gas results in passive ICU patients ventilated with INTELLiVENT-ASV in different lung conditions.

**Methods:** Passive patients intubated and mechanically ventilated between June 2015 and March 2016 were included. Excluded were patients with bronchopleural fistula and brain dead patients. All patients were ventilated using a Hamilton Medical S1 ventilator in INTELLiVENT-ASV mode. Measurements were collected once for each patient during the first day of mechanical ventilation at the time of blood gas analysis. Airway pressure and flow were measured by the proximal flow sensor of the ventilator. P_PLAT_ and total PEEP (PEEP_TOT_) were measured by a 5 seconds end-inspiratory and end-expiratory occlusion, respectively. ΔP was calculated as the difference between P_PLAT_ and PEEP_TOT_.

At inclusion patients were classified into one of the three predefined lung conditions (normal lung, acute respiratory distress syndrome (ARDS), or chronic obstructive pulmonary disease (COPD)). Data are presented as median (25^th^ - 75^th^ quartiles). An analysis of variance (ANOVA) on ranks was used to compare values between each lung conditions. Statistical significance was assumed for P value below 0.05.

**Results:** 120 patients were included (80 males, age: 67 (56–75) years; ideal body weight (IBW): 65 (55–70) Kg; SAPS II: 60 (49–68)).

**Conclusions:** INTELLiVENT-ASV is able to select a lung protective ventilation with limited driving pressure in passive ICU patients with different lung conditions.

**References**

1) Amato. N Engl J Med 2015;

2) Arnal. Intensive Care Med 2012;

3) Otis. J Appl Physiol 1950;

4) Mead. J Appl Physiol 1960.

**Table 43 (abstract A602).** Ventilation selected and blood gas results

	Normal lung	Mild ARDS	Moderate ARDS	Severe ARDS	COPD	p (ANOVA)
n	49	17	32	13	9	
ΔP (cmH20)	9 (8–10)	10 (9–11)	10 (8–13)	10 (8–11)	11 (6–12)	0.127
VT/IBW (mL/Kg)	7.5 (6.6–8.1)	6.8 (6.3–7.5)	6.6 (6.1–7.0)	6.6 (5.7–7.3)	9.3 (6.9–11.1)	0.001
PPLAT (cmH20)	16 (14–18)	21 (17–24)	22 (20–24)	24 (23–26)	20 (18–22)	<0.001
PEEP (cmH20)	6 (5–7)	10 (8–12)	10 (8–15)	12 (10–18)	7 (6–10)	<0.001
PaCO2 (mm Hg)	38 (36–42)	41 (38–49)	41 (38–46)	46 (42–52)	49 (45–56)	<0.001
PaO2 (mm Hg)	88 (79–94)	81 (75–120)	81 (73–96)	74 (68–95)	76 (64–89)	0.159

#### A603 Electrical impedance tomography-based algorithm for personalized adjustment of mechanical ventilation in ards patients

##### T. Becher, V. Buchholz, D. Schädler, I. Frerichs, N. Weiler

###### University Medical Center Schleswig-Holstein, Campus Kiel, Anesthesiology and Intensive Care Medicine, Kiel, Germany

####### **Correspondence:** T. Becher - University Medical Center Schleswig-Holstein, Campus Kiel, Anesthesiology and Intensive Care Medicine, Kiel, Germany

**Introduction:** Electrical impedance tomography (EIT) can detect deleterious side effects of mechanical ventilation like alveolar cycling and overdistension ^1, 2^ that may lead to the development of ventilator-induced lung injury (VILI). The early recognition of these effects at the bedside may accelerate the necessary modification of the ventilator settings. We developed an EIT-based algorithm for personalized adjustment of positive end-expiratory pressure (PEEP) and tidal volume (V_T_) with the aim of reducing alveolar cycling and overdistension and inducing lung recruitment.

**Objectives:** To apply the EIT-based algorithm in patients with acute respiratory distress syndrome (ARDS) and to assess its feasibility for personalized adjustment of ventilator settings.

**Methods:** We conduct a pilot clinical study in n = 20 patients requiring mechanical ventilation for early ARDS. EIT measurements are performed using the PulmoVista 500 device (Dräger, Lübeck, Germany). Patients are initially ventilated according to the ARDS network algorithm ^3^ using a low PEEP strategy and V_T_ of 6 ml/kg predicted body weight (phase ARDSNet). The patients are then ventilated according to the EIT-based algorithm during a four-hour period (phase EIT). During the phase EIT, ventilation maneuvers for overdistension and alveolar cycling detection are performed every 30 minutes and PEEP and V_T_ are adjusted according to the EIT results. After the four-hour period, the resulting V_T_, PEEP, ratio of arterial partial pressure of O_2_ and fraction of inspired O_2_ (PaO_2_/FiO_2_), respiratory system compliance (C_rs_), driving pressure (ΔP ^4^), standard deviation of regional ventilation delay (SDRVD40) ^2^ and other parameters are compared to the values measured at the end of the phase ARDSNet. All results are presented as mean values ± standard deviation with p-values calculated by two-sided paired t-test.

**Results:** To date, 8/20 patients (4 men, 4 women, age 62 ± 17 years, height 171 ± 7 cm) were included. Comparing the phases ARDSNet and EIT, the interim analysis revealed that V_T_ and PEEP changed from 398 ± 54 to 388 ± 117 ml (p = 0.81) and from 8.6 ± 1.1 to 14.3 ± 2.6 mbar (p < 0.01), respectively. PaO_2_/FiO_2_ improved from 159 ± 31 to 233 ± 56 mmHg (p = 0.01). We found no significant changes in C_rs_ (from 37 ± 7 to 40 ± 13 ml/mbar, p = 0.48), ΔP (from 10.8 ± 1.6 to 9.8 ± 1.5 mbar, p = 0.14) and SDRVD40 (from 11.2 ± 4.1 to 8.6 ± 2.5 %, p = 0.07).

**Conclusions:** Our preliminary results indicate that adjustment of mechanical ventilation according to the EIT-based algorithm is feasible and leads to ventilator settings that are associated with improved oxygenation.

**References**

1. Zick G, et al. PLoS One. 2013;8(8):e72675.

2. Muders T, et al. Crit Care Med. 2012;40(3):903–11.

3. Brower RG, et al. N Engl J Med. 2004;351(4):327–36.

4. Amato MB, et al. N Engl J Med. 2015;372(8):747–55.

**Grant acknowledgement**

This work was supported by Dräger Medical, Lübeck, Germany.

#### A604 Selecting positive end expiratory pressure (PEEP) by electrical impedance tomography (EIT): a feasibility clinical study

##### N. Eronia^1^, T. Mauri^2^, S. Gatti^1^, E. Maffezzini^3^, A. Bronco^3^, L. Alban^2^, T. Sasso^2^, C. Marenghi^2^, G. Grasselli^2^, A. Pesenti^2^, G. Bellani^1,3^

###### ^1^San Gerardo Hospital, Monza, Italy; ^2^Fondazione IRCCS Ca' Granda Ospedale Maggiore Policlinico, Milan, Italy; ^3^University of Milan-Bicocca, Health Sciences, Monza, Italy

####### **Correspondence:** N. Eronia - San Gerardo Hospital, Monza, Italy

**Introduction:** PEEP is a key element of protective mechanical ventilation. Nevertheless, the identification of feasible bedside methods for "optimal" PEEP selection is still controversial.

EIT allows real time monitoring of changes in End Expiratory Lung Impedance (ΔEELI), which corresponds to end-expiratory volume. We reasoned that ΔEELI decrease after a Recruitment Maneuver (RM) might indicate alveolar de-recruitment and inadequate PEEP. Thus, we aimed at identifying PEEP level able to minimize decrease of ΔEELI.

**Fig. 66 (abstract A604).**
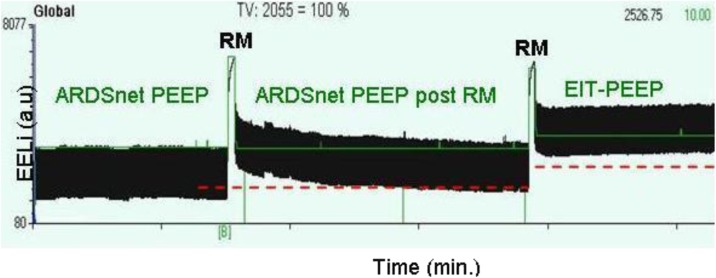
EIT PEEP setting

**Methods:** We enrolled 15 intubated sedated paralyzed hypoxemic patients undergoing controlled ventilation. The study consisted of 3 steps during which protective tidal volume and FiO_2_ were kept constant and ΔEELI was continuously monitored:

- Step 1: PEEP was selected according to the ARDSnet protocol^1^ .

- Step 2: a) a RM was performed, applying pressure of 40 cmH_2_O for 40 seconds; b) ΔEELI was measured within 30 seconds (ΔEELI_30sec_) and after 10 minutes (ΔEELI_10min_); c) if we observed that (ΔEELI_30sec_-ΔEELI_10min_) ≥10 % of ΔEELI_30sec_, a new RM was performed followed by PEEP increase by 2 cmH_2_O. Steps b) and c) were repeated until PEEP level associated with (ΔEELI_30sec_ - ΔEELI_10min_) < 10 % of ΔEELI_30sec_ (EIT-PEEP) (Fig. 66).

- Step 3: a new RM was performed, and PEEP was increased by 2 cmH_2_O (EIT-PEEP + 2).

**Results:** PEEP selection according to EIT was feasible in 13/15 patients. EIT-PEEP was significantly higher than ARDSnet PEEP (13 ± 2 cmH_2_O vs. 9 ± 2, p < 0.001); PaO_2_/FiO_2_ improved, both at EIT-PEEP and EIT-PEEP + 2 (190 ± 85 and 205 ± 93 vs. 161 ± 67, p < 0.05) but respiratory system compliance didn't change (p = 0.114). However, EIT waveforms showed that at higher PEEP regional compliance of the ventral lung was reduced (6 ± 5 ml/cmH_2_O and 5 ± 5 ml/cmH_2_O vs. 8 ± 5 ml/cmH_2_O, p < 0.01), likely indicating relative hyperdistension, and improved in dorsal lung region (22 ± 8 ml/cmH_2_O and 23 ± 10 ml/cmH_2_O vs. 12 ± 5 ml/cmH_2_O, p < 0.001), possibly indicating recruitment; a consensual and significant increase of Cumulated Hypedistension^2^ was observed in non-dependent lung zones at EIT-PEEP + 2 (+18 ± 25 % vs. ARDSnet PEEP, p < 0.001).

**Conclusions:** PEEP setting by EIT appears feasible in a large fraction of patients, allowing a bedside disclosure of recruitment and hyperdistension.

**References**

1. Acute Respiratory Distress Syndrome Network. N Engl J Med. 2000.

2. Costa EL et al. Intensive Care Med. 2009

#### A605 Impact of tidal volume setting according to recipient or donor predicted body weight in patients following double lung transplantation

##### A. Al-Fares^1^, L. Del Sorbo^2^, S. Anwar^2^, F. Facchin^2^, S. Azad^3^, R. Zamel^3^, N. Ferguson^2^, M. Cypel^3^, S. Keshavjee^3^, E. Fan^2^

###### ^1^Adult Critical Care Medicine Residency Program, Interdepartmental Division of Critical Care Medicine, University of Toronto, Toronto, Canada; ^2^University of Toronto, Department Critical Care Medicine, Toronto, Canada; ^3^Toronto Lung Transplant Program, University of Toronto, Toronto, Canada

####### **Correspondence:** A. Al-Fares - Adult Critical Care Medicine Residency Program, Interdepartmental Division of Critical Care Medicine, University of Toronto, Toronto, Canada

**Background:** Standard ventilation strategies in patients undergoing lung transplantation have not been established. The use of lung protective ventilatory strategies during and immediately after lung transplantation could minimize the potential additional mechanical injury induced by increased pulmonary stress and strain. Targeting tidal volumes based on recipient size may lead to ventilator induced-lung injury (VILI) to the donor lungs, especially if the allograft is relatively undersized, contributing to the occurrence of primary graft dysfunction (PGD).

**Objective:** To evaluate the association between mechanical ventilation settings during the first 24 hours post bilateral lung transplantation with the diagnosis of ISHLT PGD grade 3 at 72 hours post reperfusion (PGD3-72 h) and the potential role of donor/recipient lung size mismatch.

**Methods:** Retrospective study of 381 consecutive patients who underwent bilateral lung transplantation between January 2010 and December 2014 at the Toronto General Hospital. The association of the post transplant ICU mechanical ventilation settings and outcomes was performed using logistic regression.

**Results:** 33 (8.7 %) out of 381 patients had PGD3-72 h. In the univariate analysis the following settings of mechanical ventilation at ICU admission were significantly associated with PGD3-72 h: positive end-expiratory pressure (OR 1.55, 95 % CI 1.31-1.84), driving pressure (OR 1.11, 95 % CI 1.01-1.24) and tidal volume according to donor predicted body weight (donor PBW) (OR 1.35, 95 % CI 1.01-1.81). Interestingly, PGD3-72 h was not significantly associated with tidal volume according to recipient predicted body weight (recipient PBW) (OR 1.19, 95 % CI 0.80-1.77). Donor/recipient lung size mismatch was calculated according to the ratio between donor and recipient predicted total lung capacity (pTLC ratio). Tidal volume set according to recipient PBW was 6.6 mL/kg ± 0.7 in undersized grafts (pTLC ratio < 1) compared to 6.7 ml/kg ± 0.7 in non-undersized grafts (pTLC ratio ≥ 1). When tidal volume was computed according to donor PBW, it was significantly higher in patients with undersized grafts (pTLC ratio < 1) compared to non-undersized grafts (pTLC ratio ≥ 1) (7.2 ml/kg ± 0.9 vs 5.9 ± 0.7, p < 0.001). The incidence of PGD3-72 h was higher in undersized compared to non-undersized grafts (12 % vs 6 %, p = 0.052), but not statistically significant.

**Conclusions:** Following double lung transplantation, setting of tidal volume according to recipient, instead of donor, PBW may underestimate the effect of mechanical ventilation on the development of PGD3-72 h. Future studies are required to confirm the potential contribution of VILI to the occurrence of PGD.

**References**

1. Diamond JM et al. Clinical risk factors for primary graft dysfunction after lung transplantation. *AJRCCM* 2013;187:527

2. Dezube R et al. The effect of lung-size mismatch on mechanical ventilation tidal volumes after bilateral lung transplantation. *ICVTS* 2013;16:275

#### A606 Hyperoxia: at what level of SpO_2_ is a patient safe? A prospective study in mechanically ventilated ICU patients

##### E. Durlinger^1^, A. Spoelstra-de Man^1^, B. Smit^1^, H.-J. de Grooth^1^, A. Girbes^1^, H. Oudemans-van Straaten^1^, Y. Smulders^2^

###### ^1^VU University Medical Center Amsterdam, Intensive Care, Amsterdam, Netherlands; ^2^VU University Medical Center Amsterdam, Internal Medicine, Amsterdam, Netherlands

####### **Correspondence:** E. Durlinger - VU University Medical Center Amsterdam, Intensive Care, Amsterdam, Netherlands

**Purpose:** Concerns have been expressed regarding a possible association between arterial hyperoxia and adverse outcomes in critically ill patients, including those with cardiac ischemia, stroke or post-resuscitation. Oxygen status is commonly monitored noninvasively by peripheral saturation monitoring (SpO_2_). However, the risk of hyperoxia above specific SpO_2_ in critically ill patients levels is unknown. The purpose of this study was to determine a threshold value of SpO_2_ above which the prevalence of arterial hyperoxia distinctly increases.

**Design:** A prospective observational study in mechanically ventilated intensive care patients in a tertiary referral center.

**Materials and methods:** In 100 patients, we collected 200 arterial blood gases (ABG) and simultaneously registered SpO_2_ levels, as well as hemodynamic and ventilation parameters, vasoactive medication and clinically assessed peripheral perfusion. Patients under therapeutic hypothermia were excluded.

**Results:** The risk of arterial hyperoxia, defined as PaO_2_ > 100 mmHg or >125 mmHg, was negligible when SpO_2_ was ≤95 % or ≤96 %, respectively. The majority (89 % and 54 %, respectively for PaO_2_ > 100 mmHg and 125 mmHg) of ICU patients with SpO_2_ of 100 % had arterial hyperoxia. The relation between SpO2 and PaO_2_ was not clealry affected by hemodynamic or other clinical variables (pH, pCO_2_, body temperature, recent blood transfusion).

**Conclusions:** In critically ill patients, the risk of arterial hyperoxia increases when SpO_2_ is >95 %. Above this saturation level, supplemental oxygen should be administered with caution in patients potentially susceptible to adverse effects of hyperoxia.

#### A607 Effects of “express” settings in respiratory system mechanics and gas exchange in ARDS patients

##### M.A. Alfaro^1^, F. Parrilla^1^, A. Meli^2^, M. Pellegrini^2^, N. Rodriguez^1^, J.M. Goyeneche^1^, I. Morán^1^, H. Aguirre^1^, J. Mancebo^1^

###### ^1^Hospital de la Santa Creu i Sant Pau, Servei de Medicina Intensiva, Barcelona, Spain; ^2^AOU Cittá della Salute e della Scienza di Torino, Department of Anesthesia and Critical Care, Torino, Italy

####### **Correspondence:** M.A. Alfaro - Hospital de la Santa Creu i Sant Pau, Servei de Medicina Intensiva, Barcelona, Spain

**Introduction:** The “Express” strategy is a method for PEEP titration, where PEEP is set to reach an airway plateau pressure (Pplat) of 28 to 30 cmH_2_O in ARDS patients^1^.

**Objectives:** To compare the changes between the baseline ventilator settings and the “Express” strategy in terms of respiratory system mechanics and gas exchange.

**Methods:** Patients with early (<48 hours) moderate ARDS were included (Berlin definition).

Baseline settings were adjusted according to our routine clinical practice and thereafter PEEP level was titrated to reach a Plat 28–30 cmH_2_O (“Express” strategy). FiO_2_, respiratory rate (RR) and tidal volume (Vt) were kept unchanged. Measurements of respiratory system mechanics and gas exchange were done at baseline and after 2 hours of “Express” settings. Patients were classified as *responders* or *non responders* according to their change in PaO_2_/FiO_2_ (>25 mmHg) as described ^2^.

**Results:** We prospectively analysed 12 consecutive patients (9 males, aged 56 ± 19 years). A comparison of respiratory system mechanics and gas exchange between baseline and “Express” is detailed in Table 44 As expected, the overall comparisons showed significant changes in PEEP and Pplat and a significant improvement in oxygenation. The main differences between *PEEP responders* (n = 6) and *PEEP non responders* (n = 6) are shown in Table 45.

**Conclusions:** Overall, patients with moderate ARDS significantly improved oxygenation when the PEEP “Express” strategy was implemented. However, half of our patients did not improve oxygenation and parameters of respiratory mechanics also worsened.

**References**

1. Mercat A et al. Positive end-expiratory pressure setting in adults with acute lung injury and acute respiratory distress syndrome: a randomized controlled trial. JAMA. 2008 Feb 13; 299 (6): 646–55.

2. Goligher EC et al. Oxygenation Response to Positive End-Expiratory Pressure predicts mortality in Acute Respiratory Distress Syndrome. A secondary analysis of the LOVS and ExPress Trials. Am J Respir Crit Care Med 2014 Jul 1;190(1): 70–6

**Table 44 (abstract A607).** Mechanical and gasometrical variables

Variable (n=12)	Baseline	“Express”	p=
*Vt (ml/kg PBW)*	6.0 ± 0.3	6.0 ± 0.2	0.81
*Vt (ml)*	382 ± 56	385 ± 63	0.54
*RR (b.p.m.)*	23 ± 4	22 ± 4	0.39
*PEEP (cmH*_*2*_ *O)*	9 ± 3	15 ± 1	<0.001
*Pplat (cmH*_*2*_ *O)*	20 ± 3	27 ± 1	<0.001
*Respiratory system compliance (ml/cmH*_*2*_ *O)*	37 ± 11	35 ± 12	0.35
*Driving Pressure (cmH*_*2*_ *O)*	11 ± 2	11 ± 2	0.31
*PaO*_*2*_ */FiO*_*2*_ *(mmHg)*	150 ± 31	198 ± 58	0.03
*PaCO*_*2*_ *(mmHg)*	46 ± 5	52 ± 11	0.06

**Table 45 (abstract A607).** Analysis between responders and not responders

Variable	PEEP responders (n=6)	PEEP non responders (n=6)	p=
*PEEP baseline (cmH*_*2*_ *O)*	8 ± 3	10 ± 1	0.21
*PEEP "Express" (cmH*_*2*_ *O)*	15 ± 1*	15 ± 2*	0.27
*DP baseline (cmH*_*2*_ *O)*	11 ± 2	11 ± 3	0.13
*DP "Express" (cmH*_*2*_ *O)*	10 ± 2	13 ± 1	0.04
*C*_*RS*_ *baseline (ml/cmH*_*2*_ *O)*	39 ± 12	35 ± 11	0.58
*C*_*RS*_ *"Express" (ml/cmH*_*2*_ *O)*	40 ± 15	30 ± 6*	0.15
*PaO*_*2*_ */FiO*_*2*_ *baseline (mmHg)*	141 ± 38	159 ± 22	0.34
*PaO*_*2*_ */FiO*_*2*_ *"Express" (mmHg)*	243 ± 44*	154 ± 25	0.003

*DP* driving pressure, *C*_*RS*_ respiratory system compliance

*p<0.05 vs. baseline

#### A608 The global inhomogeneity index is not suitable to determine the best PEEP

##### S.J.H. Heines, U. Strauch, D.C.J.J. Bergmans

###### Maastricht University Medical Centre+, Maastricht, Netherlands

####### **Correspondence:** S.J.H. Heines - Maastricht University Medical Centre+, Maastricht, Netherlands

**Introduction:** Best dynamic compliance (Cdyn) has been proposed to titrate PEEP(1). Since electrical impedance tomography (EIT) gives regional information on ventilation distribution it may be superior for optimizing PEEP. To support the decision making of PEEP setting, guided by EIT, the global inhomogeneity index (GI) was developed. The GI is a global value which quantifies the homogeneity of tidal volume distribution. The smaller the GI, the more homogeneous tidal volume is distributed within the ventilated area. It has been argued whether best PEEP is established when the air is most homogeneously distributed in the lungs(2).

**Objectives:** We hypothesize that the GI is not suitable to determine best PEEP and EIT guided PEEP is not identical to PEEP set at the best Cdyn.

**Methods:** In our institution best PEEP guided by EIT is determined as the best compromise between alveolar overdistension and alveolar collapse during an incremental and decremental PEEP trial. Data were analysed with offline software (EITdiag; Dräger Medical) that calculates overdistension and collapse. In a retrospective analysis of 71 patients with respiratory failure due to different reasons we compared the best PEEP according to EIT with Cdyn and according to the GI with Cdyn. A PEEP difference greater than or equal to 4 cmH_2_O is defined as a clinical relevant difference. All patients were ventilated with Evita Dräger ventilators in a BIPAP mode.

**Fig. 67 (abstract A608).**
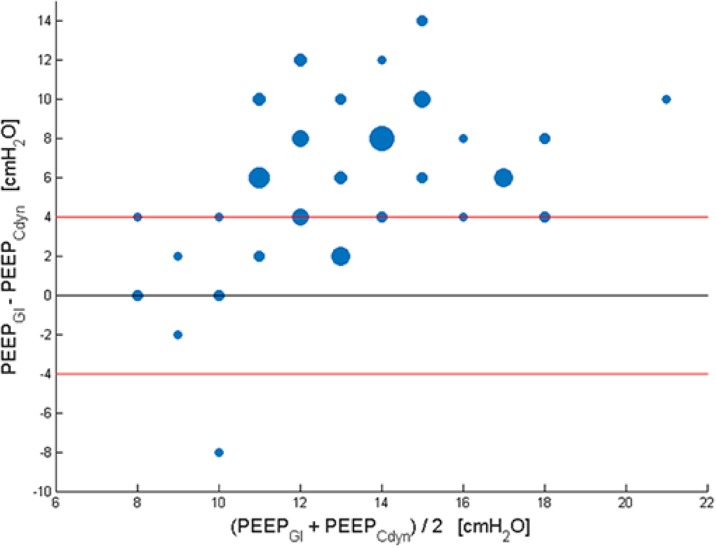
ᅟ

**Fig. 68 (abstract A608).**
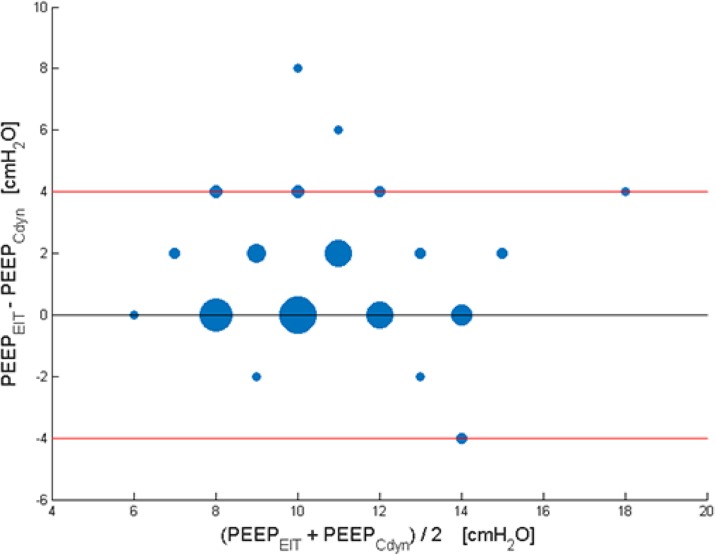
Bland Altman plot of differences in PEEP between EIT and Cdyn

**Results:** In 82 % (58/71) of the cases GI PEEP differs 4 cmH_2_O or more compared to Cdyn and in 18 % (13/71) of the cases between EIT and Cdyn. The individual differences are shown in Figs. 67 and 68.

**Conclusions:** The GI is not useful to determine best PEEP in the above mentioned manner. Best global Cdyn may be influenced by the effects of overdistension as well as atelectasis(3). EIT can visualize this, enhancing the decision on the best compromise between alveolar overdistension and collapse. Whether EIT guided PEEP is better than Cdyn guided PEEP remains to be determined.

**References**

1. Suarez-Sipmann F, Bohm SH, Tusman G, Pesch T, Thamm O, Reissmann H, et al. Use of dynamic compliance for open lung positive end-expiratory pressure titration in an experimental study. Crit Care Med. 2007;35(1):214–21.

2. Zhao Z, Steinmann D, Frerichs I, Guttmann J, Moller K. PEEP titration guided by ventilation homogeneity: a feasibility study using electrical impedance tomography. Crit Care. 2010;14(1):R8.

3. Karsten J, Grusnick C, Paarmann H, Heringlake M, Heinze H. Positive end-expiratory pressure titration at bedside using electrical impedance tomography in post-operative cardiac surgery patients. Acta Anaesthesiol Scand. 2015;59(6):723–32.

**Grant acknowledgement**

No grant was received for this study.

#### A609 Regional overdistention measured by electrical impedance tomography

##### P. Blankman, A. Shono, D. Hasan, D. Gommers

###### Erasmus Medical Center, Adult Intensive Care, Rotterdam, Netherlands

####### **Correspondence:** P. Blankman - Erasmus Medical Center, Adult Intensive Care, Rotterdam, Netherlands

**Introduction:** In order to avoid Ventilator Induced Lung Injury (VILI) it is important to improve ventilation homogeneity. Homogeneous ventilation reduces lung stress and strain diminishing lung damage. To open up atelectatic tissue recruitment maneuvers can be performed. However, it is remains unclear whether fast or slow PEEP steps should be applied.

**Objectives:** Ventilation homogeneity, as measured by Electrical Impedance Tomography (EIT), during fast and slow PEEP trials in healthy and ARDS lungs.

**Methods:** In eight pigs EIT measurements were performed before and after induction of ARDS with oleic acid. A fast PEEP trial was performed from 0–26 cmH_2_O in steps of 2 cmH_2_O. The slow PEEP trial was performed with PEEP levels from 0–25 cmH_2_O in steps of 5 cmH_2_O. During the fast PEEP trial each PEEP step was applied for 1 minute, whereas during the long PEEP trial each PEEP level was applied for 5 minutes. From the EIT data the intratidal gas distribution (ITV) was calculated. The ITV divides the impedance curve of the inspiration in eight equal-volume sections, from which the contribution of the dependent and non-dependent region during a breath can be calculated.

**Results:** For the fast PEEP trial homogeneous ventilation was found at 6 and 14 PEEP in non-ARDS and ARDS lungs respectively. For the slow PEEP trial this was 5 and 15 cmH_2_O.

**Conclusions:** ARDS lungs need significantly higher PEEP levels for homogeneous ventilation as compared to healthy lungs. However, no difference in optimal PEEP could be found between fast and slow PEEP steps. Therefore, we advice to apply fast PEEP steps in order to avoid high forces acting on lung tissue for a longer period.

#### A610 Application of adaptive support ventilation in korean patients with acute lung injury

##### W.Y. Chung^1^, K.S. Lee^1^, Y.J. Jung^1^, J.H. Park^2^, S.S. Sheen^1^, K.J. Park^1^

###### ^1^Ajou University, Pulmonology, Suwon, Republic of Korea; ^2^Ajou University, Suwon, Republic of Korea

####### **Correspondence:** W.Y. Chung - Ajou University, Pulmonology, Suwon, Republic of Korea

**Introduction:** Adaptive support ventilation (ASV) is a novel electronic ventilator protocol that incorporates the recent and sophisticated measurement tools and algorithms.

The target tidal volume and respiratory rate are continually adapted to patient's respiratory physics and varying medical conditions. In injured lung, the ASV should actively adjust ventilatory parameters achieving minimal work of breathing to meet the lung protective strategies.

**Objectives:** We applicated ASV to patients with lung injury to verify its efficacy in Korean population.

**Methods:** We observed initial mechanical ventilation parameters in 114 patients receiving ASV due to various causes (48 lung injuries including 27 community acquired pneumonia, 9 hospital acquired pneumonia, 5 interstitial lung diseases, 4 pulmonary tuberculosis and 3 idiopathic cases; 66 without lung injury which comprise 33 trauma cases, 18 strokes, 9 suicidal attempts and 6 other cases). The mean age of studied population was 57.5 years (male: female = 42:15). The data were collected within the first 12 hours of mechanical ventilation.

**Results:** Mean age of lung injury group was 63 years (53.3 for normal lung group; *p* < 0.05), PaO_2_ per inspired fraction of O_2_ (PF ratio) was 207.7 ± 61.2 (388 ± 103 for normal lung group, p < 0.05), minute volume was 7.6 ± 2.21 l (7.1 ± 1.83 l for normal lung group, *p* > 0.05), inspiratory flow was 43.0 ± 8.2 l/min (39.2 ± 10.3 l/min for normal lung group, *p* > 0.05), expiratory flow was 41.8 ± 11.4 L/min (39.2 ± 12.2 l/min for normal lung group, *p* > 0.05), peak pressure and plateau pressure were 26.8 ± 10.2 cmH_2_O and 23.8 ± 6.0 cmH_2_O (20.3 ± 4.8 cmH_2_O, 16.2 ± 3.9 cmH_2_O for normal lung group, *p* > 0.05), inspiratory resistance was 13.5 ± 5.6 cmH_2_O/s/l (12.4 ± 5.3 cmH_2_O/s/l for normal lung group, *p* > 0.05). Static compliance was measured at 26.7 ± 7.9 ml/cmH_2_O in lung injury group (60.7 ± 12.2 ml/cmH_2_O in normal lungs; *p* < 0.05), and inspiratory to expiratory time ratio in lung injuries was 0.5 (0.55 in normal lungs, *p* > 0.05). Expiratory time constant (RCexp) in lung injuries was 0.54 ± 1.7 s (0.79 ± 2.2 s in normal lungs). In lung injury patients, the tidal volume was smaller (8.35 ± 2.38 ml/Kg vs 6.20 ± 1.89 ml/Kg in normal lung group, *p* < 0.05) and respiratory rate was higher (19.8 breaths/min vs 15.2 breaths/min for normal lung group, *p* < 0.05).

**Conclusions:** As expected, adaptive support ventilation delivered smaller tidal volume and higher respiratory rates for injured lungs.

ASV efficiently operated in Korean ALI patients without any serious drawbacks and favorably adjusted the tidal volume and respiratory rates combination in relation with RCexp to meet lung protective strategies.

**Note:** This abstract has been previously published and is available at [1]. It is included here as a complete record of the abstracts from the conference.

**References**

1. Intensive Care Med (2009) 35: 13. doi:10.1007/s00134-009-1593-2

#### A611 An approach to improve uniformity in the delivery of lung protective ventilation in the intensive care unit

##### R. Worral, S. Denham, P. Isherwood

###### University Hospital Birmingham, Critical Care Medicine, Birmingham, UK

####### **Correspondence:** R. Worral - University Hospital Birmingham, Critical Care Medicine, Birmingham, UK

**Introduction:** LPV is the gold standard for mechanically ventilated patients with features of, or at risk of ARDS^1^. Uptake of ARDSnet guidelines into clinical practice has been variable^3^; this service improvement project identifies some of the barriers to changing practice and demonstrates that improvement is possible.

**Objectives:** We identified the variation in practice in mechanical ventilation across an 78 bedded mixed Intensive Care Unit (ICU) in a tertiary referral centre and implemented a strategy to promote LPV. We aimed to identify and address the barriers to the use of LPV through an ongoing education program.

**Methods:** Audits have been performed comparing unit practice to the guidelines for LPV issued by ARDSnet. All staff on the unit were invited to respond to an electronic survey regarding the use of LPV in our unit, Unit guidelines have been written to guide all staff in the use of LPV and bedside laminates supporting LPV decision making have been attached to all the ventilators in our unit.

**Results:** There has been an improvement in the percentage of patients compliant with lung protective ventilation from 14.9 % to 29.0 %, a decrease in peak airway pressures greater than 30 cmH20 and an increase in those ventilated with tidal volumes less than 6 ml/kg (26 % to 64 %). There is an ongoing downward trend in tidal volumes with 64 % of the final cohort ventilated at < 6 ml/kg compared with a majority of 6–8 mls/kg in the previous two cohorts. Key barriers to the use of LPV were identified.

**Conclusions:** We have demonstrated that with guideline implementation, education, promotion and decision support tools LPV performance can be improved. Whilst the tide is changing, there is still a significant performance gap; the implementation of change into healthcare practice faces challenges of information dissemination, unit culture, perceived role understanding, authority hierarchy and variation between multiple autonomous practitioners.

**References**

1. Petrucci N, De Feo C. Lung protective ventilation strategy for the acute respiratory distress syndrome. Cochrane Database of Systematic Reviews 2013, Issue 2. Art. No.: CD003844. DOI: 10.1002/14651858.CD003844.pub4.

2. The Acute Respiratory Distress Syndrome Network. Ventilation with lower tidal volumes as compared with traditional tidal volumes for acute lung injury and the acute respiratory distress syndrome. New England Journal of Medicine 2000; 342: 1301–1308.

3. Dickson RP. Mechanical ventilation of patients with and without ARDS: how far have we come? Respiratory Care 2013; 58(4): 712–714

**Grant acknowledgement**

Nil

#### A612 Typical patterns of expiratory flow and carbon dioxide in mechanically ventilated patients with spontaneous breathing

##### S.E. Rees^1^, S. Larraza^1^, N. Dey^2^, S. Spadaro^3^, J.B. Brohus^4^, R.W. Winding^2^, C.A. Volta^3^, D.S. Karbing^1^

###### ^1^Aalborg University, Respiratory and Critical Care Group (Rcare), Department Health Science and Technology, Aalborg, Denmark; ^2^Herning Hospital, Department of Anesthesiology, Herning, Denmark; ^3^University of Ferrara, Department of Morphology, Experimental Medicine and Surgery. Section of Anaesthesia and Intensive Care. Arcispedale Sant' Anna, Ferrara, Italy; ^4^Mermaid Care A/S, Aalborg, Denmark

####### **Correspondence:** S.E. Rees - Aalborg University, Respiratory and Critical Care Group (Rcare), Department Health Science and Technology, Aalborg, Denmark

**Introduction:** Inappropriate mechanical ventilation can lead to dynamic hyperinflation and autoPEEP. In the absence of an esophageal catheter, dynamic hyperinflation is typically evaluated from the expiratory flow profile with the absence of zero flow at end expiration illustrating incomplete expiration. In patients with spontaneous breathing, expiratory profiles can vary breath to breath, making interpretation of flow profiles difficult. Additional information may be present in the capnography signal. As incomplete expirations will result in early termination of the expiratory CO2 signal and low end-tidal CO2 (ETCO2), a combination of complete and incomplete expirations will result in variability in ETCO2.

**Objectives:** This study investigates whether systematic patterns of expiratory flow and ETCO2 exist in mechanically ventilated patients with spontaneous breathing.

**Methods:** Respiratory flow and capnography signals were analysed in eleven patients on pressure support ventilation, each ventilated at 3–5 pressure support levels. Part of these data have been published previously (1). Flow signals were analysed to calculate the average number of incomplete expirations over a 20 breath window. CO2 signals were analysed to calculate the variability (2*SD) in ETCO2 over the same window. Data including 2350 sets of 20 breaths were categorized according to the analysis of flow into 3 groups, few incomplete expirations (<2 in 20), some incomplete expirations (2–18 in 20) and many incomplete expirations (>18 in 20); and the variability of ETCO2 was calculated for each of these groups. A Wilcoxon rank sum test was used to compare median ETCO2 variability between groups.

**Results:** Significant differences were found for median ETCO2 variability between the three groups. For few (<2) and many (>18) incomplete expirations, ETCO2 variability was low (0.13 kPa and 0.19 kPa, respectively) indicating consistently complete or incomplete expirations. For the group of some incomplete expirations (2–18) ETCO2 variability was significantly higher (p < 0.05), at 0.44 kPa, with great variability observed in both expiratory flow and CO2 signals.

**Conclusions:** These results indicate that systematic patterns can be seen in expiratory flow and capnography signals. Combining these signals may provide a useful source of information for easier evaluation of dynamic hyperinflation.

**References**

1. A mathematical model approach quantifying patients´ response to changes in mechanical ventilation: Evaluation in pressure support. Larraza S, Dey N, Karbing DS, Jensen JB, Nygaard M, Winding R, Rees SE. J Crit Care. 2015 Oct;30(5):1008–15.

**Grant acknowledgement**

DSK and SER are minor shareholders and perform consultancy for Mermaid Care.

### THE IMMUNOCOMPROMIZED PATIENT IN THE ICU

#### A613 Postoperative morbidity and mortality in cardiac surgery patients with a history of malignancy

##### F. Ampatzidou^1^, A. Vlachou^2^, G. Kehagioglou^2^, T. Karaiskos^2^, A. Madesis^2^, C. Mauromanolis^2^, N. Michail^2^, G. Drossos^2^

###### ^1^G. Papanikolaou Hospital, Cardiac Surgery ICU, Thessaloniki, Greece; ^2^G. Papanikolaou Hospital, Cardiac Surgery, Thessaloniki, Greece

####### **Correspondence:** F. Ampatzidou - G. Papanikolaou Hospital, Cardiac Surgery ICU, Thessaloniki, Greece

**Introduction:** Cancer survival rates are improving over time. Co-existence of cardiac disease requiring cardiac surgery and a history of malignancy is not unusual. Whether malignancy history impairs short-term outcomes after cardiac surgery has not been well studied.

**Objectives:** Aim of this study is to describe the characteristics and outcomes of patients with a history of solid tumor or hematological malignancy who underwent cardiac surgery procedure under the use of cardiopulmonary bypass.

**Methods:** A total of 1618 consecutive patients underwent cardiac surgery from May 2012 to March 2016. Our clinic's electronic database was searched for patients with a history of solid tumor or hematological malignancies (Group A) The following factors were compared between group A and group B (control): Age, Euroscore II, total red blood transfusions > 3units, post-op low cardiac output syndrome (LCOS), prolonged mechanical ventilation (>24 hours), post-op non-invasive ventilation(NIV) because of respiratory failure, acute kidney injury(AKI-RIFLE criteria), post-op atrial fibrillation and mortality. Statistical analysis was based on x square test.

**Results:** History of malignancy was found in 47 (9 females) patients (2,9 %, group A): 9 prostate, 8 breast, 5 colon/rectum,5 lung,4 stomach,3 laryngeal,2 bladder, 1 kidney,1 skin,1 salivary gland cancer,

7 lymphomas (2 Hodgin) and 1 sarcoma. In all patients, cancer was in remission. Performed procedures were (6 emergency):41 CABG ± valve, 5 thoracic aorta surgery and 1 resection of heart sarcoma. Cancer patients were older- mean age 68,6 ± 7,6 vs 65,1 ± 10,6- and had slightly higher Euroscore II :2,4 ± 2,4 vs 2,1 ± 3,3. Median ICU days were 2 for both groups. Median mechanical ventilation time was 8 hours for group A vs 7 hours for group B. No re-intubations, no strokes, no sternal wound infections were recorded for group A while mortality was 0 %. Comparing the 2 groups for transfusions with > 3RBCs (9/47, p = 0,43), post-op atrial fibrillation (19/47, p = 0,39), AKI(7/47, p = 0,85), NIV(5/47, p = 0,94), prolonged ventilation (3/47, p = 0,8) and LCOS (1/47, p = 0,58) no statistical difference was found.

**Conclusions:** History of cancer has no impact on postoperative mortality and morbidity in patients after cardiac surgery under the use of cardiopulmonary bypass.

**References**

1. Yolanda Carrascal Cardiac Surgery With Extracorporeal Circulation in Cancer Patients: Influence on Surgical Morbidity and Mortality and on Survival. Rev Esp Cardiol. 2008;61:369–75

2. J. Chan Cardiac Surgery in Patients with a History of Malignancy: Increased Complication Rate but Similar Mortality Heart Lung and Circ 2012; 21(5) :255–259

#### A614 Did the outcome of critically ill immunocompromised patients change over the last decade?

##### N. Saraj, S. Rijkenberg, H.M. Feijen, H. Endeman

###### OLVG, ICU, Amsterdam, Netherlands

####### **Correspondence:** S. Rijkenberg - OLVG, ICU, Amsterdam, Netherlands

**Introduction:** The outcomes of HIV-infected patients, hematologic transplant recipients and cancer patients have gradually improved over the last 30 years outside the ICU. But research on the outcomes of these and other groups of critically ill immunocompromised patients is scarce. It is unclear if these patients show a comparable improvement in outcome and, if so, whether this is due to a change in patients' characteristics such as severity of illness.

**Objectives:** Determine the outcomes and characteristics of critically ill immunocompromised patients over the last decade.

**Methods:** Retrospective cohort study in a Dutch Intensive Care Unit (ICU) between 2005 and 2015. All adult patients fulfilling the definition of the APACHE II comorbidity immunologic insufficiency were eligible for the analysis. The outcomes were analyzed in two cohorts: 2005–2010 (I) and 2011–2015 (II).

**Fig. 69 (abstract A614).**
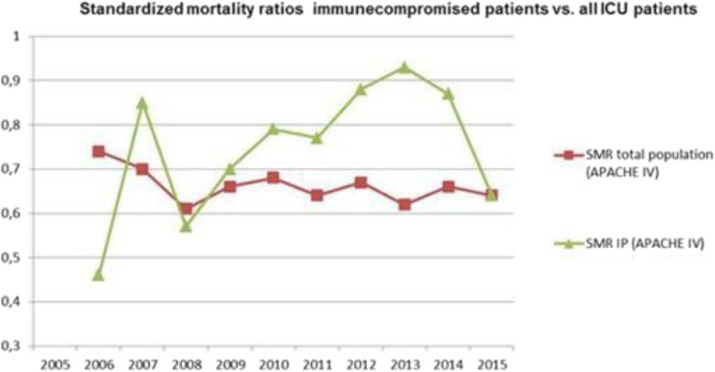
SMR

**Results:** A total of 861 immunocompromised patients were admitted to the ICU between 2005 and 2015 (cohort I: n = 457 and cohort II: n = 404). After correction with the APACHE IV pm, there was no significant difference in ICU mortality (I:16 % vs. II:21 %) and hospital mortality (I:24 % vs. II:30 %) between the cohorts (adjusted odds ratio (“adj. OR”): 1.5, 95%CI [0.92- 2.36] and adj. OR: 1.3, 95%CI [0.89-1.93], respectively). Patients in cohort II had 11 % more medical admissions (p = 0.001), used 6 % more oral anti-diabetic medication (p = 0.002) and suffered 5 % more from chronic kidney insufficiency (p = 0.012) compared to cohort I. The standardized mortality ratio (SMR) of cohort I was 0.71 95%CI [0.61-0.79] vs. 0.82 95%CI 0.74-0.87] in cohort II. See Fig. 69.

The largest immunocompromised group consisted of patients using corticosteroids (n = 206 cohort I, n = 140 cohort II). The ICU mortality in this group showed an increase of 9 % in cohort II (15 % vs. 24 %, p = 0.047) and the hospital mortality showed an increase of 8 % (23 % vs. 31 %, p = 0.158). The mortality rates corrected for the APACHE IV pm did not significantly differ between the cohorts (adj. OR: 1.35 95%CI [0.74-2.45] and adj. OR: 2.1 95%CI [0.995-4.51]). Patients in cohort II had 13 % more medical admissions (p = 0.02), used 10 % more oral anti-diabetic medication (p = 0.01) and suffered 6 % more from chronic kidney insufficiency (p = 0.03) compared to cohort I.

**Conclusions:** Survival of immunocompromised critically ill patients, including of the largest subgroup, did not change over the last decade, however, patient characteristics did. Patients admitted during the last five years have more co-morbidities and had more medical admissions. These results suggest that recently admitted critically ill immonocompromised patients are more seriously ill than in the past.

**Grant acknowledgement**

We have no interests to declare.

#### A615 An audit of HIV testing in a general adult critical care unit - is a universal opt out policy the way forward?

##### A.A.J. Donnelly, E. Morgan, H. Garrard, H. Buckley

###### Royal Bolton NHS Trust, Anaesthesia and Intensive Care, Bolton, UK

####### **Correspondence:** A.A.J. Donnelly – Royal Bolton NHS Trust, Anaesthesia and Intensive Care, Bolton, UK

**Introduction:** 25 % of UK HIV patients remain undiagnosed.A large proportion present with a late diagnosis1.Late diagnosis is linked with HIV related sequelae,often culminating in critical care admission.Early diagnosis in critically ill patients has a marked impact on outcomes and costs.Testing based on traditional HIV risk factors lacks sensitivity.The UK National Guideline (UKNG) suggests using certain HIV indicator illnesses to prompt testing2.This may provide a framework for HIV testing in critical care.No formal critical care specific guideline exists in the UK. Less than 10 % of UK critical care units follow a local HIV testing policy and the minority using UKNG report poor compliance3.

**Objectives:** We aimed to audit all patients admitted to our critical care unit against UKNG for HIV testing and to assess HIV prevalence.UKNG recommends where HIV prevalence exceeds 2/1000 of the population,all medical admissions should be tested.Public Health England data places our prevalence at 1.8/1000 of the population.Local expert opinion suggests the prevalence is over 2/1000 when accounting for local catchment area.We aimed to evaluate our critical care unit's HIV prevalence and proportion of patients presenting with late diagnosis.

**Methods:** A retrospective audit of all admissions to Bolton NHS Trust's critical care unit from 1/1/15 to 31/12/15 was undertaken using the critical care database.During this period no HIV testing protocol was in place.Critical care consultants requested HIV tests based on personal knowledge or after consultation with an HIV specialist.An HIV indicator illness had a defined aetiology other than HIV.The virology lab database was assessed for all HIV tests sent within the Trust and cross-referenced to the critical care database.

**Results:** 388 records were identified(382 patients, 6 re-admissions).68 % were medical patients and 32 % were acute or elective surgical.No patients were known to be HIV positive before admission.28 patients (7 %) were tested for HIV. 71 % of these had an acute respiratory illness as the HIV indicator illness.96 patients met UKNG criteria for HIV testing. 80 % of these had an acute respiratory illness.6/28 patients tested positive for HIV equating to a prevalence 15/1000 population.4/6 HIV positive patients presented with late diagnosis.

**Conclusions:** Based on national guidelines,25 % of patients admitted to our critical care unit,had indicators to test for HIV.Ad hoc testing only lead to 7 % of admissions being tested.The positive rate was high,leading us to assume missed diagnosis in the interested population. Based on this data,we are changing our policy to routine testing for all admissions with an opt-out for some patient groups.Other UK critical care units have adopted a similar strategy3.

**References**

1. Public Health England. HIV in the United Kingdom 2013. Colindale

2. BHIVA,BASHH,BIS (2008) UK National Guidelines for HIV Testing

3. Nardone A et al.HIV in the UK:test,test, and test again.Lancet 2013;382:1687–88.

#### A616 Bleeding, thrombosis and thromboelastography in intensive care patients with haematological malignancy and severe sepsis

##### L. Russell^1,2^, N. Haase^1,2^, A. Perner^1^

###### ^1^Rigshospitalet, Copenhagen University Hospital, Copenhagen, Denmark; ^2^Hvidovre Hospital, Copenhagen, Denmark

####### **Correspondence:** L. Russell - Hvidovre Hospital, Copenhagen, Denmark

**Introduction:** Intensive care unit (ICU) patients with haematological malignancy have an increased risk of bleeding and thrombotic complications^1^, adding to complications such as sepsis, respiratory failure and renal failure and thereby increasing the mortality^2–5^.

Traditionally conventional methods (such as platelet count, APTT, INR and fibrinogen) remain the standard approach, but more recently global haemostatic methods such as thromboelastography (TEG) have gained impact^6^.

**Objective:** The aim of this study was to observe whether TEG could predict bleeding and thrombosis in haematological ICU patients.

**Methods:** Post-hoc analysis of haematological patients included in a single ICU centre in the 6S trial (ClinicalTrials.gov NCT00962156)^7^. Clinical characteristics, TEG measurements within 24-hours of randomisation and bleeding complications according to WHO criteria^8^ were retrieved from the 6S trial database. The patient's electronic files were reviewed for thrombotic events. We used receiver operating characteristic (ROC) curves to analyse the ability of TEG to predict bleeding events.

**Results:** 202 patients with severe sepsis were admitted to the ICU of Rigshospitalet, Copenhagen, and included in the 6S trial within 6 (IQR 1–16) hours of admission. Of these, 41 had haematological malignancy and were analyzed in the present study. Median age, SAPS II and SOFA score were 66 (IQR 61–69), 64 (IQR 52–74 and 10 (IQR 9–11), respectively (Table 46). During the ICU stay 19 patients (46 %) had a bleeding episode. Nine (22 %) of these patients bled within the first five ICU-days. Two (5 %) patients had a thrombosis during the first five ICU-days. None of the 41 patients had a “normal” TEG with all parameters within the reference ranges (Table 47). There was no obvious relationship between TEG values and the risk of thrombosis. All baseline TEG variables were poor predictors of both bleeding within 5 days of inclusion and during the ICU admission (Table 48).

**Conclusion** Baseline TEG did not predict bleeding in our heterogeneous cohort of haematological ICU patients with severe sepsis. Thrombotic events were few.

**References**

1. Cook et al. *Haematologica*. 2006;91:1530–1537.

2. Azoulay É et al. *Medicine (Baltimore).* 2004;83:360–370.

3. Park HY et al. *Leuk Lymphoma*. 2008;49:1929–34.

4. Lenglin E, et al. *Leuk Lymphoma*.2012;53:1352–1359.

5. Schellongowski P et al. *Haematologica*. 2011;96:231–7.

6. Ågren A et al. *Scand J Clin Lab Invest.* 2013;73:214–20.

7. Perner A et al. *N Engl J Med*. 2012;367:124–34.

8. Miller A et al. *Am Cancer Soc.* 1981;47:207–214

**Table 46 (abstract A616).** Patient characteristics

Haematological disease:	
- Acute myeloid leukaemia	15
- Non-hodgkin's lymphoma	9
- Multiple myeloma	5
- Chronic lymphatic leukemia	5
- Hodgkin's disease	3
- Myelodysplastic syndrome	2
- Acute lymphoblastic leukaemia	2
Shock - no.(%)	38/41 (93%)
Mortality within 90 days - no. (%)	24/41 (59%)

**Table 47 (abstract A616).** Thromboelastography at ICU-admission

	No.	R-time Median (IQR)	Angle Median (IQR)	MA Median (IQR)	FF-MA Median (IQR)	Minor/mild blood loss (WHO grade 1–2) No.	Severe/Debilitating blood loss (WHO grade 3–4) No.
All patients	41	10 (8–11)	54 (41–66)	54 (46–69)	24 (16–32)	16	11
Bleeding within 5 days	9	11 (8–11)	61 (44–67)	59 (47–67)	24 (17–31)	6	2
Bleeding during ICU-stay	19	10 (8–12)	54 (47–61)	53 (46–66)	24 (18–31)	10	9
Non-bleeding patients	22	9 (8–11)	51 (37–68)	55 (45–71)	23 (15–32)	0	0
Thrombosis (Patient no1 and 2)	2	18 and 9	19 and 82	69 and 52	43 and 18	0	0

**Table 48 (abstract A616).** Area under the ROC curve

	Bleeding within 5 days	Any bleeding in the ICU
R-time, AUC (95%-CI)	0.53 (0.28–0.77)	0.50 (0.31–0.68)
Angle, AUC (95%-CI)	0.56 (0.34–0.78)	0.49 (0.30–0.68)
MA, AUC (95%-CI)	0.54 (0.34–0.74)	0.58 (0.40–0.76)
FF-MA, AUC (95%-CI)	0.50 (0.29–0.71)	0.54 (0.36–0.72)

#### A617 HIV testing in intensive care: a year's experience of opt-out testing

##### C. Goh^1,2^, K. Mouyis^1^, C.L.N. Woodward^3^, J. Halliday^1^

###### ^1^Milton Keynes University Hospital NHS Foundation Trust, Department of Critical Care, Milton Keynes, UK; ^2^University of Oxford, Wellcome Trust Centre for Human Genetics, Oxford, UK; ^3^Milton Keynes University Hospital NHS Foundation Trust, Blood Borne Virus Clinic, Milton Keynes, UK

####### **Correspondence:** C. Goh - University of Oxford, Wellcome Trust Centre for Human Genetics, Oxford, UK

**Introduction:** Undiagnosed HIV infection poses a significant public health burden in the UK; a fifth of infected individuals remain unaware.^1^ Our ICU is situated in Milton Keynes, a city with an unexpectedly high HIV prevalence and one of the highest late diagnosis rates in the UK.^2^ This is relevant to critical care as HIV infection may influence a patient's outcome and since ICU admission provides a unique opportunity to offer testing.

UK Guidelines for HIV testing^3^ do not specifically address ICU patients. However, consideration of universal testing is recommended for medical inpatients when local prevalence exceeds 2 per 1000, as in Milton Keynes. Our previous practice of HIV testing was based on informal assessment of risk, with correspondingly low testing rates. Evidence shows that testing rates increase when opt-out policies are adopted in other settings^3^ and we elected to implement this in our ICU. In patients with capacity, we sought consent prior to testing. In those lacking capacity, testing was performed in their best interests. There is limited UK data on the cost-effectiveness of expanded HIV testing but US data suggests it is cost-effective if 1 new diagnosis is made per 1,000 tests.^4^ We report our experiences following a year of opt-out testing.

**Objectives:** To compare HIV testing rates before and after the implementation of an opt-out policy.

**Methods:** All unplanned admissions to our ICU were included and pathology records interrogated to determine if HIV testing was performed during ICU admission or in the 3-month preceding period. We compared testing rates prior to (August 2014-January 2015; 6 months) and after (April 2015-March 2016; 12 months) the introduction of opt-out testing.

**Fig. 70 (abstract A617).**
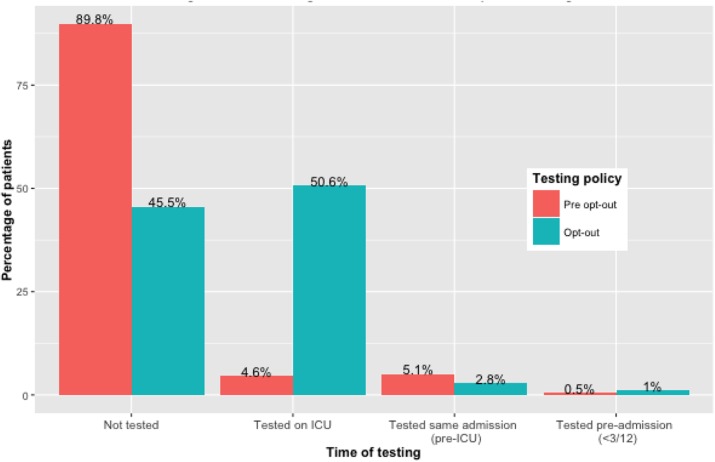
HIV testing rates before and after opt-out testing

**Results:** Our initial 6-month analysis (n = 196) showed that the majority of patients (89.8 %, n = 176) were not tested during the study period. Of the 10.2 % (n = 20) tested, 4.6 % (n = 9) were tested on ICU. We admitted 391 patients in the first year of opt-out testing. The percentage of untested patients halved from 89.8 % to 45.5 % [Fig. 70] due to a 10-fold increase in ICU testing rate with 50.6 % of patients (n = 198) receiving testing on ICU. The number of patients tested prior to ICU admission remained low. Of the 207 tests performed on ICU, one new diagnosis was made and the patient integrated into local specialist services. Of the patients able to consent, none declined testing.

**Conclusions:** Implementation of an opt-out testing policy improved our rates of HIV testing and yielded one new diagnosis. We have found this new policy to be feasible and cost-effective.

**References**

1 www.gov.uk/government/uploads/system/uploads/attachment_data/file/477702/HIV_in_the_UK_2015_report.pdf*(accessed 6/4/2016)*

2 www.gov.uk/government/uploads/system/uploads/attachment_data/file/469327/LA_UTLA_Prevalence_tables20102015.xls*(accessed 6/4/2016)*

3 www.bhiva.org/documents/Guidelines/Testing/GlinesHIVTest08.pdf*(accessed 6/4/2016)*

4 Walensky *et al.*, Am J Med 2005; 118(3):292–300

#### A618 Characteristics and 14-day mortality risk factors of oncological critically ill patients

##### G.B. Encina^1^, J. Ros^2^, L. Lagunes L^1^, J. Tabernero^3^, F. Bosch^4^, J. Rello^5^

###### ^1^Vall d´Hebrón University Hospital, Intensive Care Unit, Barcelona, Spain; ^2^Hospital Vall d'Hebron, Clinical Oncology Department, Barcelona, Spain; ^3^Vall d´Hebrón University Hospital, Clinical Oncology Department, Barcelona, Spain; ^4^Vall d´Hebrón University Hospital, Clinical Haematology Department, Barcelona, Spain; ^5^Vall d´Hebrón Research Institute, Barcelona, Spain

####### **Correspondence:** G.B. Encina - Vall d´Hebrón University Hospital, Intensive Care Unit, Barcelona, Spain

**Introduction:** Over the last decade, survival rates in critically ill cancer patients have improved dramatically. [1] Earlier admission to the intensive care unit (ICU) has resulted in better survival rates for critically ill cancer patients. [2] Selecting patients likely to benefit from ICU admission is based on complex criteria, and some ICU specific therapies such as mechanical ventilation; have been associated with an increase in mortality. [3]

**Objectives:** To identify clinical characteristics of oncological patients admitted to our ICU and to identify risk factors associated with 14 day mortality in this specific population.

**Methods:** Five - year retrospective observational analysis was performed including all patients with a personal history of active oncological malignancies admitted to the ICU of a tertiary care University Hospital in Spain. Patients´ demographic, clinical features and outcome were recorded. Data were expressed as frequency (percentage) and median (interquartile range IQR). Multivariate logistic regression with significant covariables at 0,05 was performed to identify specific risk factors for 14 day mortality.

**Results:** One hundred and thirty six patients were registered in this period. Age (median 60,IQR 2575: 5270), ICU Length of stay (LOS) (median 5 (IQR 2575:28) Principal reason to admission to ICU was respiratory failure (41,9 %), followed by septic shock (33,8 %) and low conscious level (8,1 %). Top three solid malignancies were lung (27.2 %), followed by breast (12.5 %) and Bowel (11.8 %). No differences in co morbidities between Survivors and nonsurvivors at 14 day were assessed. APACHE II score was higher in non survivors median 25 (IQR 2575:18,731,5; p = 0,002) versus survivors and this group had more frequently mechanical ventilation and vasopressors. Multivariate logistic regression for 14 day mortality assessed mechanical ventilation OR:3.59 (CI95%:1.438.97, p = 0,006) and APACHE II increases in 1score OR 1.06 (CI95%:1.011.23, p = 0,021) as independent risk factors for 14 day mortality in ICU.

**Conclusions:** Oncological critically ill patients remain as a high mortality population. Mechanical ventilation and increases in one point in APACHE II score at admission to ICU are independent risk factor for 14 day mortality.

**References**

1. Thiéry G, Azoulay E, Darmon M et al. Outcome of cancer patients considered for intensive care unit admission: a hospitalwide prospective study. J Clin Oncol. 2005 Jul 1;23(19):440613.

2. Larche J, Azoulay E, Fieux F, et al.: Improved survival of critically ill cancer patients with septic shock. Intensive Care Med 29:1688 1695, 2003

3. Azoulay E, Alberti C, Bornstain C, et al.: Improved survival in cancer patients requiring mechanical ventilatory support: Impact of noninvasive mechanical ventilatory support. Crit Care Med 29:519525, 2001

#### A619 Hospital mortality in patients with haematological diseases on mechanical ventilation in intensive care unit

##### D. García Huertas, F. Manzano, E. Morente-Constantin, B. Rivera-Ginés, M. Colmenero-Ruiz

###### Complejo Hospitalario de Granada, Granada, Spain

####### **Correspondence:** D. García Huertas - Complejo Hospitalario de Granada, Granada, Spain

**Background:** Patients diagnosed with hematological diseases may require admission to the hospital Intensive Care Unit (ICU) because of severe complications from the disease or treatment received.

**Objective:** To evaluate hospital mortality of haematological patients requiring mechanical ventilation over a period of nine years in a medical-surgical ICU.

**Methods:** A prospective, descriptive and single-center study with haematological patients admitted to the ICU in the period 2006–2015. Baseline demographic data were: age, sex, APACHE II score, haematological diagnosis, time of admission and discharge from ICU and hospital, duration of invasive mechanical ventilation and non invasive, history of surgery and mortality (ICU, hospital, one year post-discharge). Statistical analysis: descriptive, bivariate (chi ^2^ and t-Student) and multivariate logistic regression analysis.

**Results:** We included 121 patients. The percentage of male was 64.5 %, and average age of 54 ± 16 years. History of surgery 9.9 %. APACHE II score was 27.1 ± 8 points. The percentage of different hematological diseases were: 21.8 % Acute Myeloblastic Leukaemia, 27.7 % of Non Hodgkin Lymphoma, Hodgkin Lymphoma 10.1 %, Acute Lymphoblastic Leukaemia: 5.8 %; 21.5 %, Chronic Myeloid Leukaemia 5.9 %, and Chronic Lymphoid Leukaemia: 5.9 %, Multiple Myeloma: 9.2 %, Aplastic anaemia: 3.4 %, etc. ICU mortality was 68.6 %, the hospital mortality 76.9 % and the mortality one year post-discharge was 86.7 %.The average length of stay in the ICU was 13.6 ± 16 days, compared to the average hospital stay: 22.9 ± 27 days.

The average number of days of mechanical ventilation was 9.4 ± 8.7, and noninvasive mechanical ventilation: 2.36 ± 3.2.Hospital mortality of acute Myeloblastic Leukaemia and non-Hodgkin lymphoma were 69 and 76 % respectively.

**Conclusion** Mortality in ICU of haematological patients is very high, being higher in patients diagnosed with acute myelogenous leukemia and non-Hodgkin lymphoma.

#### A620 Evolution of ICU and six-month mortality in hematopoietic stem cell transplantation recipients admitted to an ICU

##### A. Naharro Abellán, L. Pérez Pérez, A. Pérez Lucendo, P. Matía Almudévar, J. Palamidessi Domínguez, P. Rodríguez Villamizar, J. García Sanz, I. Fernandez Simon, B. Lobo Valbuena, S. Alcantara Carmona

###### Hospital Universitario Puerta de Hierro Majadahonda, Majadahonda, Spain

####### **Correspondence:** A. Naharro Abellán - Hospital Universitario Puerta de Hierro Majadahonda, Majadahonda, Spain

**Introduction:** Although hematopoietic stem cell transplantation recipients (HSCTR) admitted to an ICU have a high mortality (52 %-83 %^1^), limited data exists regarding six-month mortality rates. The aim of our study was to investigate the outcomes and explore non-respiratory predictors of ICU and six-month mortality, and to compare our result with a previous publication^2^ done in our center, in order to learn if outcome has improved.

**Methods:** Retrospective study. We analyzed all HSCTR who were admitted to our ICU from January 2009 to March 2015. We recovered demographic data, reason for ICU admission and all the possible variables related with mortality. Statistical analysis was done using the Chi-square test for categorical variables and the Mann–Whitney test for continuous non-parametric variables.

**Results:** During the study period a total of 25 HSCTR required ICU care, and this accounted for 33 admissions (7 of them were readmitted). General patient characteristics are shown in Table 49.

The most frequent cause for ICU admission was acute respiratory failure (56 %) followed by non-respiratory sepsis (7 patients; 28 %) and by acute coronary syndrome, brain stroke, hemophagocytic syndrome and veno-occlusive disease (one case each; 4 %). Twenty patients had 2 or more organ failures: respiratory (64 %), hemodynamic (64 %) and renal (72 %; use of renal replacement techniques in 32 %). All patients received antibiotic treatment, and 9 of them (36 %) had positive microbiological results. Mean ICU stay was 9 days (2–35).

In our study, ICU mortality was 48 % (12 of 25 patients), in-hospital mortality 60 % (15/25) and six-month mortality was 72 % (18 of 25 patients). Compared to mortalities reported 30 years ago for our center the rates that we found were lower (ICU-mortality 48 vs 76 %; in-hospital mortality 60 vs 88 %)^2^. Factors related to ICU and six-month mortality are listed in Table 50. Non-respiratory laboratory parameters (lactic, hemoglobin, INR, aPTT, fibrinogen) and isolated respiratory (p = 0,053) and hemodynamic (p = 0,386) failures together with the use of vasoactive drugs (p = 0,819) renal replacement therapies (p = 0,236) or positive cultures (p = 0,656) did not reach statistical significance.

**Conclusions:** Pre-ICU hospital days, 24 h SOFA, number of organ failures and isolated renal failure are factors associated with ICU mortality. The presence of lower counts in the first 24 h of platelets, leukocytes and neutrophils together with pre-ICU hospital days were related with six-month mortality. Although mortality rates in HSCTR admitted to ICU have decreased, ICU, in-hospital and six-month survival rates are still low.

**References**

1 Afessa, Bekele, and Elie Azoulay. "Critical care of the hematopoietic stem cell transplant recipient." *Critical care clinics* 26.1 (2010): 133–150.

2 Torrecilla, C et al. "Prognostic assessment of the acute complications of bone marrow transplantation requiring intensive therapy." *Intensive care medicine* 14.4 (1988): 393–398.

**Table 49 (abstract A620).** General patient characteristics

	Overall	ICU mortality			6 month mortality		
		Survivors	Exitus	p	Survivors	Exitus	p
Sex (% of women)	28%	30,77%	25%	0,748	28,57%	27,78%	0,968
Age (years)	44,72±16,32	49,85±15,42	39,17±16,32	0.0969	49,43±18,27	42,89±16,32	0,2498
Underlying disorder							
Lymphoblastic leukemia	20%	1	4	0,181	1	4	0,353
Non-Hodgkin lymphoma	4%	1	0		0	1	
Multiple myeloma	20%	4	1		3	2	
Marrow aplasia	20%	1	4		0	5	
Lymphoblastic lymphoma	4%	1	0		1	0	
AML	16%	2	2		1	3	
Chronic Lymphocytic Leukemia	4%	1	0		0	1	
Myelodysplastic syndrome	8%	2	0		1	1	
Panmyelosis	4%	0	1		0	1	
Type of HSTC							
Allogeneic	79,17%	7 (36,84%)	12 (63,16%)	0,043*	3 (15,79%)	16 (84,21%)	0,0401*
Autologous	16,67%	4 (100%)	0		3 (75%)	1 (25%)	
Microtransplant	4,17%	1 (100%)	0		0	1 (100%)	
GHCD	40,00%	38,46%	41,67%	0,87	42,86%	38,89%	0,856
CMV	35%	20%	50%	0,16	0,00%	46,67%	0,058
APACHE II	20,75±5,64	19,62±5,08	22,09±5,64	0,257	18,71±3,73	21,59±5,64	0,2514
SOFA	8,38±3,98	6,54±3,89	10,55±3,98	0,0061*	6,86±1,95	9±3,98	0,0787

Continuous variables are shown as mean ± sd

P<0,005

**Table 50 (abstract A620).** Factors related to sixmonth or ICU morta

	Overall	ICU mortality			6 month mortality		
		Survivors	Exitus p	p	Survivors	Exitus	p
Pre-ICU hospital days	17,2±20,46	7±10,12	28,25±20,46	0,0058*	3±5,1	22,72±20,46	0,0068*
SOFA	8,38±3,98	6,54±3,89	10,55±3,98	0,0061*	6,86±1,95	9±3,98	0,0787
Number of organ failures	2,84±1,34	2,31±1,18	3,42±1,34	0,0475*	2,43±1,27	3±1,34	0,3846
Renal failure^a^	72%	71%	72%	0,968	54%	92%	0,035
Platelets count	90960±97849	133615±120817	44750±97849	0,1737	174571±132024	58444±97849	0,0341*
Leucocytes count	7205±10144	9863±12978	4326±10144	0,1573	15191±15888	4100±10144	0,0065*
Nutrophils count	5204±5730	6949±6831	3313±5730	0,1572	10389±7479	3187±5730	0,0092*

P<0.005

^a^Renal failure is the only qualitative variable. Show as frequency

#### A621 Assessing the impact of the new british committee of standards in haematology guideline on the outcome of critically ill patients with haematological disease

##### M. Pais, S. Ramalingam

###### Heart of England NHS Foundation Trust, Intensive Care, Birmingham, UK

####### **Correspondence:** M. Pais - Heart of England NHS Foundation Trust, Intensive Care, Birmingham, UK

**Introduction:** Outcomes for haematology patients admitted to intensive care have improved in the last decade and this has thought to be due to a collection of factors rather than single treatment. In 2015, the British Committee of Standards in Haematology published an evidence based guideline outlining the major considerations in managing critically ill patients with haeamtological disease, for haematologists and intensivists.

**Objectives:** To evaluate the impact of the guideline on mortality and use of ICU resources, for adult haematology patients at our institution.

**Methods:** We performed a retrospective analysis of 71 patients with a haematological disease, admitted to our 19 bed ICU between 2013–2016. Information was collated from the Intensive care National Audit and research database and from patient records. Data was then organised into 2 groups (pre and post guideline) and analysed using SPSS.

**Results:** Patients in the study were separated into 2 groups, 40 in the pre-guideline group vs 30 in the post guideline group. A statistically significant improvement in mortality was found in the group after the guideline had been implemented (43 % vs. 68 % p < 0.05) however there was no difference between the groups in duration of intensive care stay or number of organs supported. Admission APACHE-II scores were significantly better after the guideline had been introduced (22.3 ± 7.11 vs. 25.5 ± 6.3) as were measures of functional status and premorbid state. Of the patients who had respiratory failure there was no significant difference between the groups regarding use of NIV or early intubation that could account for our mortality results.

**Conclusions:** The implementation of the new guideline has resulted in reduced mortality for patients admitted to ICU with a haematological disease. While aspects of the guideline have made no difference to outcome, our results demonstrate that early referrals by haematologists prior to developing multiorgan failure in individuals who have good-moderate functional capacity, have a positive effect on mortality. In our institution this was achieved with increased reliance on early warning scoring systems, critical care outreach teams and consultant to consultant referrals.

**References**

1. Wise MP et al. Guidelines on the management and admission to intensive care of critically ill adult patients with haematological malignancy in the UK; British Committee for Standards in Haematology. British Journal Haematology. 2015.

#### A622 Receptors of a hematopoietic stem cell transplantation admitted to an intensive care unit. Description and analysis of factors related to survival

##### C. Díaz^1^, L. Fox^2^, M. Santafe^1^, P. Barba^2^, M. García^1^, S. Leal^1^, M. Pérez^1^

###### ^1^Vall d'Hebron University Hospital, Intensive Care, Barcelona, Spain; ^2^Vall d'Hebron University Hospital, Hematology, Barcelona, Spain

####### **Correspondence:** C. Díaz - Vall d'Hebron University Hospital, Intensive Care, Barcelona, Spain

**Introduction:** Receptors of a hematopoietic stem cell transplantation (HSCT) are a population with a high risk of developing severe complications that require admission to intensive care units (ICU), which is related to a high mortality. So, efforts are needed in order to improve their prognostic.

**Objectives:** Our aim was to describe the cohort of receptors of an HSCT admitted to a tertiary hospital ICU and analyze factors that are likely to affect their survival.

**Methods:** Retrospective study including adult (+18 years) patients who received an HSCT and required admission to ICU between 01 January 2010 and 29 February 2016. Quantitative variables are presented as median (IQR) and categorical as N (%).

**Results:** Fifty patients were included, 30 (65 %) men, with a median age of 52 (34–60) years. The underlying leading conditions were leukemia, with 23 (46 %) patients, and lymphoma, with 17 (34 %). Reduced intensity chemotherapy was employed in 24 (47 %). Allogenic transplantation was done in 42 (84 %) cases. Twenty-two (44 %) suffered GVHD, that was acute in 18 (36 %) and chronic in 7 (14 %). The patients presented on ICU admission a median of 3 (2–4) organ failures, distributed as follow: respiratory 32 (64 %), hemodynamic 25 (50 %), renal 29 (58 %), liver 13 (26 %), hematological 37 (74 %), metabolic 22 (44 %). Initial mechanical ventilation (MV) was required in 15 (30 %), 21 (42 %) high flow nasal cannula (HFNC), only one (2 %) non invasive mechanical ventilation and 11 (27 %) low flow systems. Finally, 27 patients (54 %) received MV. Thirty five (70 %) patients needed vasoactive drugs and 14 (28 %) renal replacement therapy (RRT). The time from the transplantation to ICU admission was 33 (12–122) days. APACHE score was 22 (15–27). Neutropenia on admission was present in 20 (43 %). The HCT-CI was 3 (2–4).Twenty-two (46.8 %) patients were discharged to the hematology ward and 14 (29.8 %) were discharged home. Factors related to mortality: number of organ failures (3.5 vs 2.2; p = 0.016), need of MV (OR = 4.4, IC95%:1.2-16.9, p = 0.024) and renal failure (OR = 10.6, IC95%:2.4-47.6, p = 0.002). Patients who initially were treated with HFNC and needed MV had a similar mortality than patients initially on MV. None of the 14 patients on RRT survived. We did not find differences in mortality related to APACHE score, type of HSCT, GVHD, neutropenia and need of vasoactive drugs. In the multivariate analysis only renal failure maintained statistical significance (OR: 9.1, IC95%: 1.9-42.4, p = 0.005).

**Conclusions:** Receptors of a HSCT admitted to an ICU have a great hospital mortality (70.2 %). Patients who need MV or suffer renal failure have the worse prognostic. Patients initially on HFNC who required MV did not have a different outcome compared to patients initially on MV.

#### A623 Evolution of hematopoietic stem cell transplant patients with acute respiratory failure admitted to an ICU

##### M.L. Pérez Pérez^1^, A. Naharro Abellán^1^, A. Pérez Lucendo^1^, P. Matia Almudevar^1^, J. Palamidessi Domínguez^1^, P. Rodríguez Villamizar^1^, J. Veganzones^1^, I. Fernandez Simón^1^, B. Lobo Valbuena^1^, N. Martínez^2^, S. Alcántara Carmona^1^

###### ^1^Hospital Universitario Puerta de Hierro Majadahonda, Intensive Care, Madrid, Spain; ^2^Hospital Universitario Puerta de Hierro Majadahonda, Madrid, Spain

####### **Correspondence:** M.L. Pérez Pérez - Hospital Universitario Puerta de Hierro Majadahonda, Intensive Care, Madrid, Spain

**Introduction:** From March 1981 to June 1987, 25 patients (40 %) with a previous Hematopoietic Stem Cell Transplant (HSCT) needed ICU support in our tertiary care hospital. The main cause of admission was acute respiratory failure (ARF)(18 patients). Sixteen (64 %) required mechanical ventilation (MV). Mortality amongst this group was 94 % and more than 7 days of MV was a strong factor associated with it^**1**^.

**Objectives:** To study the evolution of HSCT patients with ARF that require ICU care, the factors associated with mortality, and to compare them to the previous data recorded in our center. Statistical analysis was done using the Chi-square and the Mann–Whitney tests.

**Methods:** Retrospective study from January 2009 to March 2015. All patients admitted to the ICU with a previous history of HSCT were included. We collected demographic data, reason for admission, respiratory support applied and factors associated with mortality.

**Results:** During the period studied, 235 patients received a HSCT and 25 (10.63 %) needed ICU care. There were 33 admissions in total.

Median time between the HSCT and admission was 86 days (54–1685). Main reason for admission was ARF (14 patients; 56 %), followed by non-respiratory sepsis (7 patients; 28 %) and by acute coronary syndrome, brain stroke, hemophagocytic syndrome and veno-occlusive disease (one case each; 4 %). Median number of organs that had failed upon admission was 3 (2–4): respiratory (64 %), hemodynamic (64 %) and renal (72 %).

In the ARF group we accounted for 11 pneumonias, 2 alveolar hemorrhages and 1 pulmonary graft-versus-host disease. Seven patients met Acute Respiratory Distress Syndrome (ARDS) criteria. The most common radiographic finding was an interstitial infiltrate (14). Twenty-three patients required respiratory support: 4 high-flow nasal cannula (16 %), 8 non-invasive MV (32 %) and 11 MV (44 %; 5 after unsuccessful non-invasive MV). Median duration of MV was10 days (5–16). Only one patient needed prone position. Three patients underwent a tracheostomy.

Overall ICU mortality was 48 % (12) and 6-month survival dropped to 28 % (7). The variables associated with mortality were: failure of 3 or more organs (p = 0.047), the presence of ARDS (85.71 % vs. 38.46 %; p = 0.043), MV (72.73 % vs. 25 %; p = 0.022), pO_2_/FiO_2_ ratio < 120 in the first 24 hours (p = 0.011) and ICU readmission (6 out of 7 patients: 85,7 %).

**Conclusions:** Although nowadays fewer patients required ICU care after a HSCT, the main reason for admission hasn´t changed and the mortality amongst these patients is still high.The presence of MV, ARDS, low pO_2_/FiO_2_ ratio, three or more organ failures and ICU readmission is associated with mortality. The need for MV in our study was lower that in the previous one probably due to an improvement in other ways of respiratory support.

**References**

1. Torrecilla et al.Prognostic assessment of the acute complications of bone marrow transplantation requiring intensive therapy.Intensive Care Med (1988) 14; 393–398.

**Fig. 71 (abstract A623).**
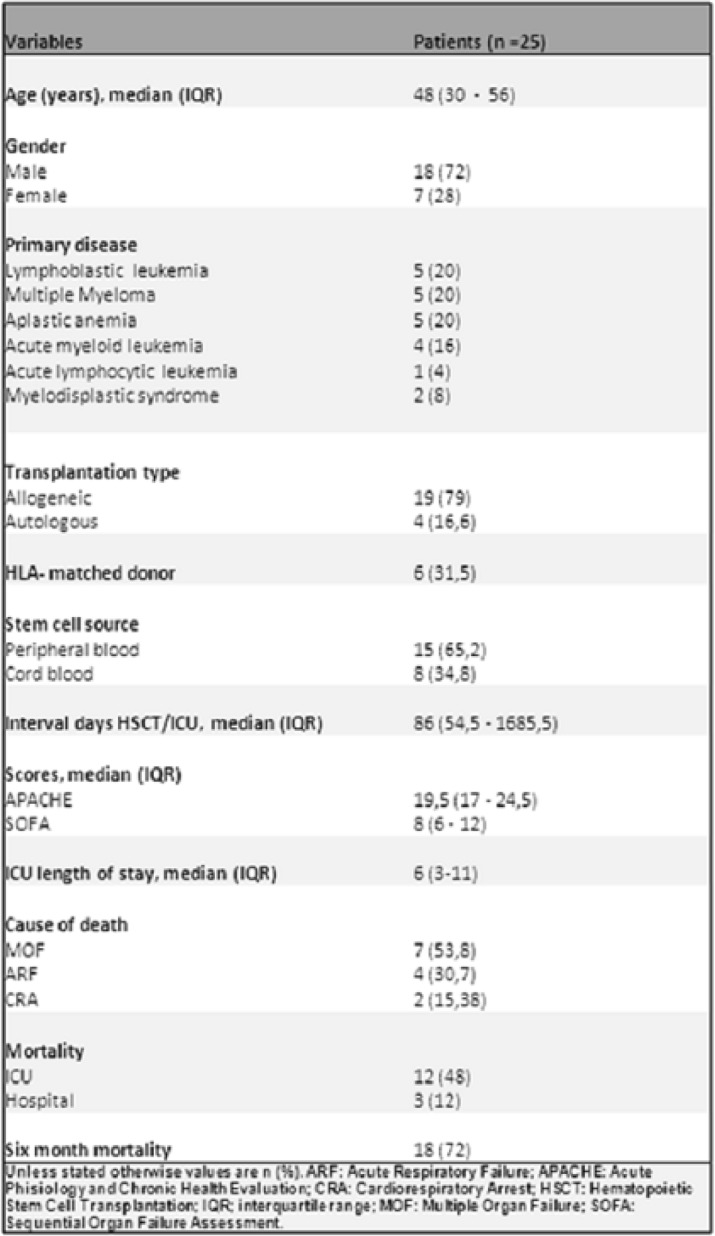
Demographic characteristics

#### A624 Early versus late ICU admission in haematological patients receiving chemotherapy: impact on outcome and prognostic indicators

##### I. Moors^1^, D. Mokart^2^, F. Pène^3^, J. Lambert^4^, A. Kouatchet^5^, J. Mayaux^6^, F. Vincent^7^, M. Nyunga^8^, F. Bruneel^9^, L. Laisne^4^, A. Rabbat^3^, C. Lebert^10^, P. Perez^11^, M. Chaize^4^, A. Renault^12^, A.-P. Meert^13^, R. Hamidfar^14^, M. Jourdain^15^, M. Darmon^4^, B. Schlemmer^4^, S. Chevret^16^, V. Lemiale^4^, E. Azoulay^4^, D. Benoit^17^

###### ^1^Ghent University Hospital, Hematology, Ghent, Belgium; ^2^Institut Paoli Calmettes, Marseilles, France; ^3^Hôpital Cochin, Paris, France; ^4^Hôpital Saint Louis, Paris, France; ^5^Centre Hospitalier Universitaire d'Angers, Angers, France; ^6^Groupe Hospitalier Pitié-Salpêtrière Charles Foix, Paris, France; ^7^Hôpital d'Avicenne, Bobigny, France; ^8^Centre Hospitalier de Roubaix, Roubaix, France; ^9^Hôpital Mignot, Versailles, France, ^10^Centre Hospitalier Départemental site de Montaigu, La Roche Sur Yon, France; ^11^Hôpital Brabois, Nancy, France; ^12^Hôpital de la Cavale Blanche, Brest, France; ^13^Institut Jules Bordet, ULB, Brussels, Belgium; ^14^Hôpital Albert Michallon, Grenoble, France; ^15^Hôpital Roger Salengro, Lille, France; ^16^Paris Diderot Sorbonne University, Paris, France; ^17^Ghent University Hospital, Ghent, Belgium

####### **Correspondence:** I. Moors - Ghent University Hospital, Hematology, Ghent, Belgium

**Introduction:** Delayed admission in the ICU (defined as time from hospital to ICU > 24 hours) has been associated with poorer outcome in critically ill patients with haematological malignancies (1), however no data exists concerning the subgroup of patients receiving chemotherapy (CT) in the ICU. Moreover, only a few small studies have assessed outcome in this subgroup.(2)

**Objectives:** The aim was to assess whether CT was associated with outcome, overall and within the subgroups of patients admitted to the ICU ≤ 24 hours vs. > 24 hours after hospitalisation. Furthermore, we wanted to identify prognostic indicators for hospital mortality within the CT subgroup.

**Methods:** Retrospective analysis of prospectively collected data on 1011 patients included between January 1^st^ 2010 and May 1^st^ 2011 in 17 French and Belgian centres.(1) Clinically important variables and variables associated with CT and mortality in univariate analysis (P < 0.2) were included in the multivariate logistic regression analysis.

**Results:** Of the 1011 patients, 928 (91 %) had complete data, and 231 of them received CT in the ICU. Overall 51.9 % of the patients were newly diagnosed and 50.6 % suffered from AML. Sixty-nine percent needed organ support (NIV, MV, vasopressors or dialysis). Hospital mortality was similar between CT and non-CT patients (38.5 %, vs 39.9 %, P = 0.715). However, CT patients were younger (median 58y vs 61y, P = 0.003), had better performance status (86.1 % vs 79.5 % with ECOG 0 to 2, P = 0.025) and a lower SOFA score (5 vs 6, P = 0.002) at admission. After adjustment for all potential confounders, CT was associated with increased hospital mortality (OR 1.52, P = 0.036) as compared to non CT patients, however only when admitted in the ICU > 24 hrs after hospital admission (OR 2.99, P < 0.001). This was not the case for CT patients admitted ≤ 24 hrs after hospital admission (OR 1.32, P = 0.396). Within the CT subgroup SOFA score (OR 1.19, P < 0.001), number of previous lines of CT (OR 1.33, P = 0.039) and comorbidity (CCI) (OR 1.15, P = 0.071) were associated with increased mortality in multivariate analysis, while admission ≤ 24 hrs (OR 0.44, P = 0.012) was protective.

**Conclusions:** Outcome of patients receiving chemotherapy in the ICU was similar to the general population with haematological malignancies, however only when admitted within 24 hrs after hospital admission. Earlier referral to the ICU may be a key factor to reduce mortality in high risk patients requiring chemotherapy.

**References**

1. Azoulay E. et al. Outcomes of critically ill patients with hematologic malignancies: prospective multicenter data from France and Belgium - A groupe de recherche respiratoire en réanimation onco-hématologique study. J Clin Oncol 2013; 31(22): 2810–18

2. Moors I et al. Urgent chemotherapy in hematological patients in the ICU. Curr Opin Crit Care, 2015; 21(6): 560–567

**Grant acknowledgement**

IM: clinical research fund Ghent University Hospital. DB: senior clinical grant FWO Flanders.

#### A625 Prognostic factors in adult cancer patients with sepsis or septic shock at admission to an intensive care unit: a single-centre retrospective cohort study

##### D. Martins-Branco^1^, M. Sousa^2^, S. Marum^2^, M.J. Bouw^2^

###### ^1^Instituto Português de Oncologia de Lisboa FG, EPE, Oncologia Médica, Lisboa, Portugal; ^2^Instituto Português de Oncologia de Lisboa FG, EPE, Unidade de Cuidados Intensivos e Intermédios, Lisboa, Portugal

####### **Correspondence:** D. Martins-Branco - Instituto Português de Oncologia de Lisboa FG, EPE, Oncologia Médica, Lisboa, Portugal

**Introduction:** Sepsis is a common complication in cancer patients and it is associated with high mortality rates. Clinical approach in critically ill cancer patients should take into account the disease course and cancer-related determinants.

**Objectives:** To characterise cancer patients admitted to an oncological intensive care unit with sepsis/septic shock and to determine baseline predictors of in-hospital mortality.

**Methods:** This is a retrospective cohort study of all the adult cancer patients admitted to a 6-bed Oncological Intensive Care Unit (ICU) with the diagnosis of severe sepsis or septic shock, between 1 January 2012 and 31 December 2015. Solid and haematological conditions were analysed separately. The analysis was performed with multivariate logistic regression models, using a stepwise backward elimination with complete case analysis. All the analysis was performed with the software Stata 12.1®. P-value was considered significant at < 0.05.

**Results:** We identified 210 patients: the mean age was 60.8 (CI 58.8-62.8) and 65 % were male (n = 136). About 19 % (n = 40) were postoperative and the remaining 81 % (n = 170) were medical septic complications; 61 % were admitted with septic shock (n = 129). The majority of cancer conditions were solid tumours - 71 %(n = 149) - and among the haematological cancers - 29 %(n = 61) - 16 % (n = 10) were admitted after bone marrow transplantation. The median ECOG performance status score was 2. Only 6 % (n = 12) were in complete response and 65 % (n = 137) were at an initial phase of the treatment. Data regarding comorbidities, tumour staging, site of infection, culture-positive sepsis and organ support at admission were included in the multivariate analysis. ICU mortality and in-hospital mortality were 34 % (n = 71) and 50 % (n = 104), respectively. Among solid tumour patients, higher scores of Simplified Acute Physiology Score II (SAPS II) and Eastern Cooperative Oncology Group (ECOG) Performance Status showed a statistically significant association with in-hospital mortality, as expected; postoperative admission, septic shock at admission tend to be associated with worst prognosis. Regarding haematological conditions, only Acute Physiology and Chronic Health Evaluation (APACHE) II score was a statistically significant predictor of in-hospital mortality.

**Conclusions:** This study highlights ECOG as a predictor of in-hospital sepsis-related mortality in patients with solid tumours and raises the awareness around the search of oncological baseline characteristics that may be determinants of sepsis-related mortality. This Oncological ICU mortality and respective in-hospital mortality rates are similar to mortality rates of sepsis/septic shock in general population. These results suggest SAPS II and APACHE II as a good predictor of in-hospital sepsis-related mortality in patients with solid tumours and haematological patients, respectively. Larger retrospective cohorts or prospective studies might strengthen these conclusions.

**Funding**

None.

#### A626 Administration of multipotent mesenchymal stromal cells (MSC) improves short term but not long term survival in oncohematological neutropenic patients (PTS) with septic shock (SS)

##### G. Galstyan^1^, P. Makarova^1^, E. Parovichnikova^2^, L. Kuzmina^3^, V. Troitskaya^2^, N. Drize^4^, E. Gemdzhian^5^, V. Savchenko^6^

###### ^1^National Research Center for Hematology, ICU, Moscow, Russian Federation; ^2^National Research Center for Hematology, Hematology Department, Moscow, Russian Federation; ^3^National Research Center for Hematology, BMT Department, Moscow, Russian Federation; ^4^National Research Center for Hematology, Physiology of Hematopoiesis Lab., Moscow, Russian Federation; ^5^National Research Center for Hematology, Biostatistics Department, Moscow, Russian Federation; ^6^National Research Center for Hematology, Moscow, Russian Federation

####### **Correspondence:** G. Galstyan - National Research Center for Hematology, ICU, Moscow, Russian Federation

**Introduction:** Sepsis and SS are one of the most serious complications in neutropenic pts. MSCs reprogram of monocytes and macrophages by releasing prostaglandin E2, IL-10 and decrease the production of TNF-α and IL-6. Injection of MSCs to septic animals reduced mortality and improved organ function [2]. No study has investigated the efficacy of MSC therapy in pts with SS, especially neutropenic pts.

**Objectives:** The aim of the study was to investigate the efficacy of MSC administration on survival and organ dysfunction in neutropenic pts with SS.

**Methods:** In prospective single center randomized Russian clinical trial of MMSCs in severe neutropenic pts with SS (RUMCESS) (NCT 01849237) 30 neutropenic pts (WBC < 0.5x10^9^/l) were enrolled. The pts were randomly assigned to receive either conventional therapy (CT) of SS (CT group) (15 pts) or CT plus donor MMSCs within the first 10 hours of SS (CT + MMSCs group) (15 pts). The SOFA score was calculated at onset of SS and in 2, 3, 7, 14, 21 and 28 days. Fisher´s exact test, Kaplan-Meier method with the log-rank test & Cox proportional hazard regression model were used.

**Results:** There were no significant differences between groups in demographic parameters. Positive blood cultures were documented in 8/15 patients in CT group and in 11/15 patients in CT + MSCs group. Baseline APACHE II scores (30.6 [95 % CI 27.0-40.1] and 30.9 [95 % CI 26.1-36.7] in the CT and MSC groups, respectively) and SOFA scores (15.9 [95 % CI 13.9-20.2] and 16.1 [95 % CI 12.1-19.0], respectively) were similar in both groups. During the study period, total SOFA score did not differ between the groups. However, since second day cardiovascular SOFA score and respiratory SOFA score were lower (p < 0.05) in MSCs-CT group, than in CT-group. 28-days survival rates were 20 % (3 out of 15 pts) in CT group and 60 % (9 out of 15 pts) in CT + MSCs group (P < 0.05).

Despite higher 28-days survival rates in MSCs + CT group only 4 of 9 pts survived 28 days remained alive in 3 months, 5 pts died due to sepsis related organ dysfunctions. In 3 months there were no differences in survival between the groups.

**Conclusions:** Administration of MSCs in first hours of SS improves organ dysfunction and short-term survival in neutropenic pts, but doesn´t prevent death from sepsis related organ dysfunctions in long term period. Perhaps, it is required repeated administration of MSCs.

**References**

1. Namendys-Silva S.A., et al. Outcome of critically ill patients with hematological malignancies Ann Hematol. 2013; 92: 699–705. 2. Németh K, et al. Bone marrow stromal cells attenuate sepsis via prostaglandin E(2)-dependent reprogramming of host macrophages to increase their interleukin-10 production. Nat Med. 2009; 15: 42–9

**Grant acknowledgement**

None

**Fig. 72 (abstract A626).**
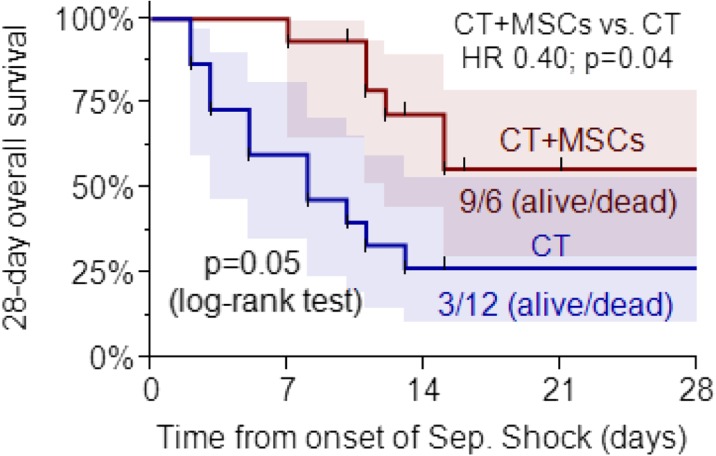
28-day overall survival

### Critical care outcomes I

#### A627 Interventional physical therapy to reduce intensive care unit ventilator-dependent patient days

##### H.-C. Chao

###### Chi Mei Medical Center, Intensive Care Medicine, Tainan, Taiwan, Province of China

**Introduction:** Patients with acute respiratory failure on ventilators constitute the majorities of patients in intensive care units (ICU). Early extubation has always been one goal that medical teams want to achieve.

**Objectives:** Rehabilitation invention has already been administered in ICUs in other countries and has successfully helped achieve the goal of early extubation. It is therefore hoped that physical therapy as intervention can help create a safe environment that facilitates early extubation for patients.

**Methods:** The participating patients with acute respiratory failure were all in the conditions of stable hemodynamics, normal nutritional status and no infectious complications and first underwent joint assessment by the medical teams based on their progresses in recovery after treatment for their diseases before receiving rehabilitation consultation, which directed them to cardiopulmonary rehabilitation that included T-tube training. Upon the confirmation of their ventilator weaning indications, their endotracheal tubes were removed. They were then engaged in early ambulation activities ranging from Level II to Level IV according to their abilities. During this period, the medical teams conducted 3 sessions of education training led by physical therapists, followed by exercises and group testing.

**Results:** Days of ventilator use were reduced from 6.3 (days) previously to 4.1 (days) after improvement. The secondary goal, i.e. days in ICUs, were reduced from 10 (days) to 6.1 (days). Hospitalization costs were also reduced from NT$329,000 before improvement to NT$241,000 during improvement and NT$186,000 after improvement. During the improvement, no tube slippage or patient falls occurred. This shows that this innovative proposal has achieved its purpose and also ensured patient safety.

**Conclusions:** It is suggested that medical teams for ventilation in severe cases can develop weaning protocols that have shared goals and integrate multiple technologies and find weaning solutions for ventilator dependent patients by exploring literature and relevance of content in protocols. This provides possible indicators for assessing healthcare for ventilator dependent patients and serves as a reference for healthcare facilities in developing patient care plans.

**References**

1. Zanni JM, Korupolu R, Fan E, et al. Rehabilitation therapy and outcomes in acute respiratory failure: an observational pilot project. J Crit Care 2010; 25: 254–262.

2. Carpenè N, Vagheggini G, Panait E, et al. A proposal of a new model for long-term weaning: respiratory intensive care unit and weaning center. Respir Med 2010; 104: 1505–1511.

3. Thomsen GE, Snow GL, Rodriguez L, et al. Patients with respiratory failure increase ambulation after transfer to an intensive care unit where early activity is a priority. Crit Care Med 2008; 36: 1119–1124.

#### A628 Retrospective evaluation of intensive care patients affected by civil war in Syria

##### E. Kılıc^1^, B. Demiriz^2^, M.L. Uygur^1^, M. Sürücü^1^, K. Cınar^3^, A.E. Yıldırım^4^

###### ^1^Sehitkamil State Hospital, Anesthesia and Reanimation, Gaziantep, Turkey; ^2^Sehitkamil State Hospital, General Surgery, Gaziantep, Turkey; ^3^Sehitkamil State Hospital, Brain Surgery, Gaziantep, Turkey; ^4^Sehitkamil State Hospital, Internal Medicine, Gaziantep, Turkey

####### **Correspondence:** E. Kılıc ^_^ Sehitkamil State Hospital, Anesthesia and Reanimation, Gaziantep, Turkey

**Introduction:** Retrospective Evalution of the Intensive Care Unit Patients Who Were Affected by Syrian Civil War.

**Objective:** The aim of this study is to examine the clinical status of the patients who were requiring intensive care as the victims of the Syrian civil war.

**Methods:** Clinical characteristics, health state at admission into General Intensive Care Unit (GICU), treatments in GICU, expected, and actual mortality rates estimated based on APACHE II scores, number of patients rejected because of full bed occupancy were evaluated retrospectively among Syrian civil war victims who applied to the GCI between 01.01.2013 - 12.31.2013.

**Results:** 159 (21.4 %) of 740 patients who were monitored in GICU belonged to a Syrian nationality who were the victims of the Syrian civil war. A total of 795 patients were requested for hospitalization in GICU, while GICU stay of 55 (6.9 %) patients were rejected because of full bed occupancy. Mean age (34.3 ± 12.4 years), gender distribution (women, n = 36; 22.6 %), and men, n = 123; 77.4 %), and the mean APACHE II score (24.2 ± 13.7) of the patients were recorded as indicated. The most common indications for hospitalization were multisitel shrapnel injuries (42.7 %), subarachnoidal bleeding (SAK) (42.1 %) caused by firearm injuries, and chest gunshot wounds (5.6 %). Total number of days of GICU hospitalization were 4300 days, while patients with Syrian nationality stayed in the GICU a total of 1558 (36.2 %) days. Mean duration of hospitalization was 9.7 days. Average mortality rate among all GICU patients in 2013 (26 %) was lower than that of Syrian citizens (n = 49 deaths, mortality rate 30.8 %; n = 12 women; 24.4 %). The standardized mortality ratio [observed mortality (49/159), according to the APACHE II score of expected mortality rate (48 /159) ratio] was 1.02

**Conclusions:** Because of higher APACHE II score, increased incidence of gunshot hwounds, and transportation of the patients from a distance of over 100 kilometer under unfavourable conditions increased mortality rates were observed.

**References**

1. Aras M, Ataş M, Yılmaz A, et al. Being a neigbor to Syria: a retropective analysis of patients brought to our clinic for cranial gunshot wounds in the Syrian civil war. Clin Neurol Neurosurg 2014;125:222–8.

2. Thanapaisal C1, Saksaen P. A comparison of the Acute Physiology and Chronic Health Evaluation (APACHE) II score and the Trauma-Injury Severity Score (TRISS) 34 for outcome assessment in Srinagarind Intensive Care Unit trauma patients. J Med Assoc Thai 2012;95 Suppl 11:S25-33.

#### A629 Different levels and variances of melatonin (MT) production observed in septic and non-septic ICU patients

##### K. Kiss^1^, B. Köves^2^, V. Csernus^3^, Z. Molnár^1^

###### ^1^University of Szeged, Szeged, Hungary; ^2^Jahn Ferenc Teaching Hospital, Budapest, Hungary; ^3^University of Pécs, Department of Anatomy, Pécs, Hungary

####### **Correspondence:** K. Kiss - University of Szeged, Szeged, Hungary

**Introduction:** The massively increasing knowledge of recent years about the importance of circadian rhythms is orientating the researchers from laboratories towards the bedside (1).

**Objectives:** The daily pattern of MT production is the main regulator of circadian rhythms, with the strongest known signal of its release being the daily change of light and darkness. The aim of this prospective study was to determine the MT production in septic and non-septic groups of ICU patients suffering from severe night light pollution.

**Methods:** Mechanically ventilated patients over 18 years, without CNS damage were enrolled in the study. Illuminance levels (IL) were measured by a lux meter. Serum MT levels were determined from arterial blood samples. Sampling and IL measurements were performed after admission at 01:00, 07:00, 13:00 and 19:00 constitutively for 96 hours. Blood samples were kept in total darkness before being centrifuged, then serum samples were placed into −80 °C until MT measurement was performed. Repeated correlation analysis was performed to investigate the correlation between illuminance and MT levels. The equality of MT variances was tested by fitting two nested models and comparing their likelihoods.

**Results:** Out of the 28 enrolled patients 15 were able to take over the study. 8 patients proved to be septic and 7 non-septic, which was sufficient to form subgroups. Out of the 16 measurements/patient, 240 MT samples were analysed. Although IL were the lowest at 01:00 [19(3–101) lux], estimated MT peaks were missing, the median (IQR) was 30(24–51) pg/ml at this time point. Although repeated correlation analysis between MT and IL showed a non-significant between-subject correlation (r = 0.438, P = 0.103), the MT variances of septic subgroup were 70 % of the non-septic variances. In spite of the low sample size the fixed effect of subgroup was statistically highly significant (P < 0.0001), in general higher MT levels were observed in the septic subgroup.

**Conclusions:** Our results indicate that circadian rhythm is desynchronized in ICU patients, as indicated by the missing MT peaks during the night, the absence of the normal 24 h rhythm of MT release and by the poor correlation between illumination and MT levels. This first observation suggests that absence of darkness at night and the missing contrast of day and night light levels can rapidly destruct the rhythm of MT release. The cause(s) of our second observation, namely that patients in septic subgroup showed lower MT variances and higher MT levels in general needs to be elucidated by studies focusing on this phenomenon, conducted in larger patient population. Based on these observations, future trials on light/dark conditions and patient outcomes could lead to redesigned healthcare environments, which may help restore circadian rhythmicity and reach health benefits.

**References**

1. MA Oldham et al. Crit Care Med 2016; 44:207–217.

#### A630 Burnout of Greek ICU healthcare workers. A cross-sectional, multi-centered study

##### A. Ntantana^1^, D. Matamis^1^, S. Savvidou^1^, M. Giannakou^2^, M. Gouva^3^, G. Nakos^4^, V. Koulouras^4^

###### ^1^Papageorgiou General Hospital, ICU, Thessaloniki, Greece; ^2^AHEPA University Hospital, ICU, Thessaloniki, Greece; ^3^Technological Educational Institutes of Ipeirus, Ioannina, Greece; ^4^University Hospital of Ioannina, ICU, Ioannina, Greece

####### **Correspondence:** A. Ntantana - Papageorgiou General Hospital, ICU, Thessaloniki, Greece

**Introduction:** Healthcare workers in the Intensive Care Unit (ICU) environment present very commonly with burnout worldwide^1^.

**Objectives:** Aim of this study was to assess burnout in ICU physicians and nurses in a national level and to identify possible contributing factors.

**Methods:** A cross-sectional, multicenter study in 18 Greek ICUs was conducted between June and December 2015. Scores of Exhaustion, Depersonalization and Personal Accomplishment from the Maslash Burnout Inventory Questionnaire^2^ were recorded in the 469 study participants. Univariate and multivariate logistic regression analysis was performed in order to identify predicting factors for burnout.

**Results:** A high participation rate was recorded (65.7 %). Baseline participant characteristics, scores of burnout, as well as statistical comparisons between the medical and nursing staff are shown in (Table 51 and 52).

ICU nurses compared to doctor participants were mainly females (80.8 % vs. 55.6 %, chi-square test, p < 0.001), almost a decade younger (p < 0.001), had less working experience (p < 0.001), and declared lower job satisfaction (p = 0.001). Exhaustion scores were relatively low but statistically different between doctors and nurses (22.8 % and 37.5 % respectively, Mann–Whitney U test, p < 0.001), while differences in scores of depersonalization and personal accomplishment did not reach statistical significant levels. Multivariate logistic regression analysis (Table 53) identified two independent predicting factors for high exhaustion: to be a nurse (Odds ratio 1.6, 95 % CI 1.1-2.7, p = 0.05) and to have low job satisfaction (Odds Ratio 0.2, 95 % CI 0.1-0.4, p < 0.001). Other factors like gender, age, working experience, working in university hospitals or having personal experience of losing a close family member were found not to be associated with burnout.

**Conclusions:** Our results demonstrate low burnout levels in Greek ICU personnel. Nurses are more exhausted compared to doctors. The major contributing factor for burnout is job satisfaction.

**References**

1. Curtis RJ, Puntillo K. Is there an epidemic of burnout and post-traumatic stress in critical care clinicians. Am J Respir Crit Care Med. 2007;175:634–635.

2. Maslash C, Jackson SE, Leiter MP. Maslash burnout inventory manual, 3^rd^ ed. Palo Alto, CA: Consulting Psychologists Press:1996.

**Table 51 (abstract A630).** Baseline participant characteristics

	Physicians n=149	Nurses n=320	p values
Male gender	63 (44.4%)	58 (19.2%)	<0.001
Age > 40 years	101 (67.8%)	119 (37.4%)	<0.001
Working experience >10years	66 (44.3%)	129 (40.9%)	<0.001
University hospital	82 (55.0%)	142 (44.4%)	0.031
Personal loss	90 (60.4%)	172 (55.1%)	NS
Job satisfaction:			0.001
Low	3 (2.1%)	22 (6.9%)	
Moderate	25 (17.1%)	95 (29.7%)	
High	118 (80.8%)	203 (63.4%)

**Table 52 (abstract A630).** Participant scores of burnout

	Physicians n=149	Nurses n=320	p values
Exhaustion (total score)	17 (13–26)	23 (17–30)	<0.001
Exhaustion:			<0.001
Low	67 (45.0%)	77 (24.1%)	
Moderate	48 (32.2%)	123 (38.4%)	
High	34 (22.8%)	120 (37.5%)	
Depersonalization	8 (4–12)	7 (3–13)	NS
Personal accomplishment	33 (29–37)	32 (28–37)	NS

**Table 53 (abstract A630).** Univariate and multivariate logistic regression

	Univariate analysis			Multivariate analysis		
Variables	Odds Ratio	95% CI	p values	Odds Ratio	95% CI	p values
Females	1.474	0.930–2.336	NS			
Age < 40y	1.194	0.810–1.759	NS			
Nurses	2.029	1.301–3.165	0.002	1.611	1.061–2.699	0.050
Working >10y	1.089	0.736–1.611	NS			
University hospital	1.443	0.980–2.124	NS			
Personal loss	0.874	0.591–1.292	NS			
Job satisfaction	0.193	0.126–0.295	<0.001	0.222	0.139–0.355	<0.001

#### A631 Ulnar length and clinician estimation of height in tidal volume calculations for lung-protective ventilation

##### S. Gaffney, E. Black, R. Docking

###### Queen Elizabeth University Hospital, NHS GG&C, Department of Critical Care, Glasgow, UK

####### **Correspondence:** S. Gaffney - Queen Elizabeth University Hospital, NHS GG&C, Department of Critical Care, Glasgow, UK

**Introduction:** Despite their importance in informing care interventions, significant barriers exist in Critical Care in regards to the measurement of height and weight. Common interventions based on weight include lung-protective ventilation in ARDS management[1], renal replacement therapy, and antibiotic dosing. Studies have investigated the use of a calibrated ulnar length (UL) tape in tidal volume (VT) measurements [2], whilst our institution uses a formula converting actual height (AH) to ideal body weight. We have demonstrated in previous audits poor compliance with measurement of actual height and wished to investigate the performance of UL to improve compliance.

**Objectives:** We performed a three-way comparison comprising height derived from UL versus AH, and clinician estimation (CE) versus AH. We sought to simulate the clinical effects of applying measured and derived values into the common clinical parameter of 6 ml/kg VT lung-protective ventilation.

**Methods:** We sampled 16 Critical Care patients and 4 healthy volunteers. Participants were measured supine, semi-recumbent or sitting. UL was measured between olecranon process and ulnar styloid. AH was measured in the position the participant was approached in, using a summation of heel to knee, knee to greater trochanter (GT), and GT to vertex lengths. CE was estimated by a Consultant Intensivist, blinded to actual measurements. Results were analysed using Excel, with correlation of results performed using Bland-Altman plots and regression analysis.

**Results:** A significant correlation between performance of UL and AH was obtained (P < 0.01). There was no significant correlation between CE and either UL or AH. Similarly, when converted to 6 ml/kg VT, a statistically significant correlation was obtained between UL and AH and (P < 0.01) but not CE.

Estimates of height ranged between -16 cm and +5 cm, most commonly underestimating height. VT extrapolated from CE gave a range between −41.4 ml and +68.4 ml.

**Conclusions:** Significant barriers to measuring height in Critical Care exist, including lack of pre-morbid measurement, immobility, and lack of suitable equipment. Anecdotally there may have been a tendency for clinicians to rely on estimation of height and weight which can erroneously form the basis for regimes in a range of interventions. Our study confirms UL validity as a proxy of formal height measurement, and the poor performance of estimation compared to AH and UL. We propose UL measurement as an acceptable standard of care.

**References**

[1] - The Acute Respiratory Distress Syndrome Network. Ventilation with lower tidal volumes as compared with traditional tidal volumes for acute lung injury and the acute respiratory distress syndrome. N Engl J Med, 2000. 342(18): 1301–8

[2] - Rivers J, Brown J, Dolphin K and Squire Y. A calibrated measuring tape accurately predicts tidal volumes from ulna length. Journal of the Intensive Care Society. 2015, 16(4): 302–305

#### A632 Development of an automated data extraction process for use in retrospective pharmacokinetic analyses: initial application to vancomycin dose and concentration data in patients using citrate and heparin CVVHDF anticoagulation modalities

##### C. Judge^1^, T. Drew^2^, H. Misran^2^, R. Munshi^3^, L. McGovern^2^, M. Coyle^2^, L. Dunne^2^, E. Deasy^3,4^, P. Lavin^1^, A. Fahy^2^, D.M. Darcy^3^, M. Donnelly^2^

###### ^1^AMNCH Tallaght Hospital, Nephrology, Dublin, Ireland; ^2^AMNCH Tallaght Hospital, Intensive Care, Dublin, Ireland; ^3^Trinity College, School of Pharmacy and Pharmaceutical Sciences, Dublin, Ireland; ^4^AMNCH Tallaght Hospital, Pharmacy, Dublin, Ireland

####### **Correspondence:** T. Drew - AMNCH Tallaght Hospital, Intensive Care, Dublin, Ireland

**Introduction:** Critically ill patients have altered antibiotic pharmacokinetic (PK) characteristics, which are further affected by continuous venovenous haemodiafiltration (CVVHDF) (1). Regional citrate anticoagulation (RCA) use is increasing and effects of RCA-CVVHDF settings on antibiotic PK parameters need quantification for dose individualisation.

Electronic prescribing and recording systems have potential to improve data availability and accuracy for PK analyses. Data extraction and amalgamation from multiple sources requires a systematic approach, considering data quality assurance and research objectives.

**Objectives:** The aim was to develop a reliable methodology to extract and amalgamate retrospective data from dialysis machines (Prismaflex), the clinical information system (ICIP) in the intensive care unit, and electronic laboratory systems, for use in future PK studies.

Using this methodology, we compared vancomycin doses and levels in patients receiving heparin anticoagulation or RCA on CVVHDF.

**Methods:** All patients on vancomycin and CVVHDF in 2014 were included. Vancomycin data were extracted from the laboratory information system; timing of dialysis and anticoagulation mode from ICIP (Philips); and dialysis flow data from the Prismaflex (Gambro) machines. Extracted data reliability was categorised as high or low risk in terms of time-sensitivity and patient-specificity for internal validation of data extraction and amalgamation. High risk data had a two part validation system to match variables across databases. Risk assessment was undertaken by a multidisciplinary team. Open source technologies were utilised including Perl (perl.org), REDCap (projectredcap.org) and MySQL (mysql.com); and SPSS V17 (IBM Corp.) was used for data analysis. Ethical approval was obtained.

**Results:** A fully coded-anonymised database for retrospective analysis was created.

58 patients were included, representing 323 patient days with an average of 5.57 (SD 2.14) days per patient. This resulted in 255, 56, 8 and 4 dialysis days on heparin, citrate, both and no anticoagulation, respectively. The average vancomycin dose was 1162.22 mg (+/−437 mg) and 1306.12 mg (+/−343 mg) on heparin and RCA respectively (p = 0.032). Trough vancomycin levels were within range in 37.9 % of levels in heparinised patients and 29.72 % of levels in patients on RCA. There was no significant association between anticoagulation and vancomycin trough levels in target range, χ2(1) = 1.563, p = .668.

**Conclusions:** This methodology represents a promising approach for retrospective data analysis. Our initial results indicate that anticoagulation modality used is associated with vancomycin dose, but not the proportion of trough levels in target range. Work is ongoing to further validate data amalgamation processes and explore vancomycin PK in these patients.

**References**

1. Trotman, Williamson, Shoemaker et al., Clin Infect Dis. (2005) 41 (8):1159–1166.doi: 10.1086/444500

#### A633 Evaluation of clinical pharmacist interventions in surgical & medical intensive care unit

##### N.H. Ismail

###### King Fhad University Hospital, Department of Pharmacy, Al-Khobar, Saudi Arabia

**Introduction:** The impact of clinical pharmacist on preventing drug related problems in critically ill patient has been well reported in several randomized control trials. The critical care pharmacist impact is greatly essential in therapeutic drug monitoring, renal dosing, drug-drug interaction, and de-escalation of antibiotic therapy and others in this setting.

**Objective:** The aim of this study was to evaluate the types of interventions, clinical significance and cost saving involved with each intervention.

**Methods:** A prospective observational study was conducted in a 13 bed critical care unit at tertiary care teaching hospital in AL-Khobar, Saudi Arabia. All Patient greater or equal to 18 yrs of age, who were admitted to our intensive care units were included in the study. Pharmacist interventions were recorded electronically on daily bases. The electronic documentation system collected the following information, patient demographics and (age, gender, and weight, past medical history, home medication, allergies, type of intervention, and outcome.

**Results:** The study group consisted of 181 patients. Three hundred and three interventions were recorded during a period of 12 months. Therapeutic drug monitoring accounted for 66 (36 %) of total pharmacist interventions, while 65 (35 %) was related to review & reconciling of medication history. Furthermore (17 %) of these interventions were related to unnecessary medications mostly antibiotics. Interventions related to renal dosing adjustment accounted for 50 (28 %) of interventions.

**Conclusions:** The overall interventions identified in this study well demonstrate the impact of critical care pharmacist in improving patient outcome and preventing drug related problems.

**References**

1. Wong T. et al. Effect of critical care pharmacist´s intervention on medication errors: A systematic review and meta-analysis of observational studies.J Crit Care. 2015 Oct;30(5):1101–6. doi: 10.1016/j.jcrc.2015.06.018. Epub 2015 Jun 24.

2. Kopp BJ, et al. Cost implications of and potential adverse events prevented by interventions of a critical care pharmacist.Am J Health Syst Pharm. 2007 Dec 1;64(23):2483–7.

3. Leape LL, et al. Pharmacist participation on physician rounds and adverse drug events in the intensive care unit.JAMA. 1999 Jul 21;282(3):267–70. Erratum in: JAMA 2000 Mar 8;283(10):1293.

#### A634 Are daily blood tests on icu necessary? How can we reduce them?

##### T. Hall, K. Wykes, J. Jack, W.C. Ngu, P. Morgan

###### East Surrey Hospital, ICU, Redhill, UK

####### **Correspondence:** T. Hall - East Surrey Hospital, ICU, Redhill, UK

**Introduction:** Daily blood tests are a long running feature of ICU care, and are regarded as an essential diagnostic tool by medical staff, however they aren't without their drawbacks. The risk of iatrogenic anaemia is a very real one and there are significant costs involved in both equipment and staff time.1 With this in mind guidelines were drawn up to limit excessive blood tests in otherwise stable patients.

**Objectives:** We audited adherence to these guidelines in 2015 finding 292 unnecessary vials of blood taken in a 20 bed unit. Guidelines were clarified and advertised on posters and then we re-audited.

**Methods:** In order to establish trends and to exclude stable post surgical patients we limited eligible patients to those who were on the unit for 3 days or more. A simple proforma was designed to record which common blood tests had been performed, daily haematocrit and haemoglobin (if tested), and any major change in overall condition or events such as transfusions or surgery. On six randomly assigned days over a 2 month period, all eligible patients on the unit had a proforma filled retrospectively for all days up until their admission to the unit, or up to seven days previously, whichever sooner. We also recorded if the patient had arterial or central access, as these facilitate obtaining samples for testing.

**Results:** 70 patient days were audited across 10 individuals, with 339 tests ordered, 41 % of which were unnecessary, down from 46 % before our intervention.

**Conclusions:** While the bedspace posters had a modest effect, we are now enforcing the guidelines through the weekly medical and nursing teaching sessions, and also ensuring all requests for clinical reasons are agreed with the registrar or nurse in charge.

We are also recommending removal or alteration of the “Critical Care” test set that is currently available from our electronic requesting system in the hope that it will encourage more critical thinking about which tests are appropriate for the patient they are caring for.

We aim to re-audit our practice within the next 6 months to ascertain the impact of these changes.

**References**

1: Goddard K, Austin SJ. Appropriate regulation of routine laboratory testing can reduce the costs of associated with patient stay in the intensive care unit. Critical Care [Internet].2011[cited 2015 Nov 8];15(Suppl 1):P133. Available from:http://ccforum.com/content/15/S1/P133

2: R Gray* and F Baldwin. Targeting blood tests in the ICU may lead to a significant cost reduction. Critical Care 2014, 18(Suppl 1):P15 doi:10.1186/cc13205

#### A635 Withdrawn

#### A636 Antimicrobial stewardship programme interventions over critical hematologic patients

##### J. Ruiz-Ramos^1^, P. Ramirez^1^, M. Gordon^1^, E. Villarreal^1^, J. Frasquet^2^, J.L. Poveda-Andrés^3^, A. Castellanos^1^

###### ^1^Hospital Universitari i Politècnic la Fe, Intensive Care Unit, Valencia, Spain; ^2^Hospital Universitari i Politècnic la Fe, Microbiology, Valencia, Spain; ^3^Hospital Universitari i Politècnic la Fe, Pharmacy, Valencia, Spain

####### **Correspondence:** P. Ramirez - Hospital Universitari i Politècnic la Fe, Intensive Care Unit, Valencia, Spain

**Introduction:** Hospital Antimicrobial Stewardship (AMS) programs have achieved savings and more rational use of antimicrobial treatments. However, intervention over antimicrobial treatment of critical patients with hematologic disease is highly complicated

**Objectives:** To evaluate a two years experience of an AMS program intervention in patients with hematologic disease admitted in an intensive care unit (ICU).

**Methods:** We designed a quasi-experimental study. AMS strategies were put into practice during three years (January 2014-January 2016) in collaboration with the ICU nosocomial infection control team. Antimicrobial treatment was revised twice a week. If any antimicrobial treatment modification was recommended in the AMS meeting, a face-to-face interview was held between AMS team and the attending physician. ICU length of stay and ICU mortality rate were compared with the same time period previous to AMS implementation. Continuous and categorical variables were compared using T-student and Fischer exact test (Stata v.13.0).

**Results:** 190 antimicrobials of 64 patients were evaluated in 69 AS team meetings [Age: Mean = 51.0 (SD = 13.8) years; APACHE II: Median (RIQ) = 22 (19–26)]. After ASM revision, there was 71 changes in antimicrobial prescriptions. Recommended amendments by the AMS team were: stop antimicrobial treatment (32; 45.1 %), change in dose (17; 23.9 %), change to a narrower spectrum antimicrobial (10; 14.1 %), monitoring drug concentrations (10; 14.1 %), and start a new antimicrobial (2; 3.0 %). Most of these recommendations (84.5 %) were accepted by the prescribing physician.

No significant changes in ICU length of stay [Pre-intervention: 6.42 (IC95%: 4.39-8.45) vs AMS intervention: 6.48 (5.23-7.96); p = 0.887), mortality (39.0 % vs 40,6 %; p = 0.827) or percentage of ICU readmission (16.0 % vs 6.6 %; p = 0.051) were observed.

**Conclusions:** AMS program focus in critical haematological patients has great potential to optimize and reduce antimicrobial consumption in the ICU.

#### A637 Caregivers' perceptions towards communication with mechanically ventilated patients: the results of a multicentre survey

##### C.E. Ijssennagger^1^, S. ten Hoorn^1^, A. van Wijk^1^, J.M. van den Broek^2^, P.R. Tuinman^1^

###### ^1^VU University Medical Center, Intensive Care, Amsterdam, Netherlands; ^2^Zaans Medisch Centrum, Intensive Care, Zaandam, Netherlands

####### **Correspondence:** C.E. Ijssennagger - VU University Medical Center, Intensive Care, Amsterdam, Netherlands

**Introduction:** Mechanically ventilated (MV) patients often experience difficulties with communication. It is well-studied that being non-vocal is experienced as distressing by most critically ill patients and could result in severe emotional reactions (1). However, the opinions of health care professionals on this subject remain unknown. Moreover, communication with ICU patients is becoming increasingly important since ICU guidelines aim to reduce the use of sedatives, thus it is expected that more patients will be awake and able to communicate (2).

**Objectives:** To investigate communication issues with MV patients as experienced by ICU health care professionals. The primary aim was to quantify the extent of the problem and to determine its effect on patient care and job satisfaction. The secondary aim was to identify patient and health care factors associated with communication difficulties and to evaluate the preference and effectiveness of currently available communication methods.

**Methods:** A multicentre survey study was conducted from December 2015 till January 2016. An online questionnaire consisting of 37 questions was sent to nurses, residents and intensivists of seven ICUs in the Netherlands.

**Results:** Out of 961 caregivers, 297 participated (30.9 % response rate), of which 267 responses were included. Communication difficulties are experienced in 50.0 % of the interactions with MV patients, resulting in 42.5 % of the professionals to lose valuable time on daily basis. More than 55 % of the participants indicated to lose between 30–60 minutes during an average shift. Consequently, 44.0 % indicated that their job satisfaction is negatively affected, primarily with feelings of unfulfillment (76.3 %) and frustration (70.8 %). To facilitate communication, 32.6 % reported to regularly use augmentative and alternative communication methods, but the most effective and preferred method remains the use of basic gestures. In addition, almost 50 % indicated to be dissatisfied with their personal communication skills with MV patients. Over 80 % of the caregivers have not been trained in communication with MV patients, while 72.0 % would like to receive training in the future.

**Conclusions:** In half of the interactions with MV patients, health care professionals experience communication difficulties. These difficulties frequently lead to negative effects on patient care and job satisfaction of ICU professionals. This further emphasizes the need for improvements such as the development of communication protocols, skills training and continued research into new communication methods.

**References**

1. Happ MB et al. Nurse-patient communication interactions in the intensive care unit. *Am J Crit Care* 2011;20(2):e28-40.

2. Barr J et al. Critical Care Medicine Clinical Practice Guidelines for the Management of Pain, Agitation, and Delirium in Adult Patients in the ICU. *Crit Care Med* 2013;41:263–306.

#### A638 Expanding icu beds and increasing its occupancy rate in a safe way in a developing country; 2 year experience

##### A.M. Elmenshawy

###### Alexandria University, Critical Care Medicine, Alexandria, Egypt

**Introduction:** ICU is a high demand area in the hospital, with a large demand supply gap especially in limited resource countries. In addition, it requires a higher staffing ratio than rest of the hospital.

**Objectives:** To explore the impact of multiple factors for increasing bed turnover during expanding newly developed ICU.

**Methods:** Observation study during gradual opening of 18 beds medical/surgical ICU from June 2014 till March 2016. It is a part of Alexandria University (primary and tertiary) hospitals covering 2 governorates receiving a lot of emergency patients. In September 2015, outreach critical care services were provided for early discharge of ICU patients but was inactive since April 2015 due to financial problem. In September 2015 post-operative ICU was opened but the nursing staff was recruited from general ICU. In January 2016, proactive ICU project was implemented which included reducing the time of delay of investigation for ICU patients, daily care plan for rapid stabilization, implementing end of life care decisions, and avoiding inappropriate antibiotic or medication use. In Feburary 2016, administration increased ICU capacity with same number of nursing staff.

**Results:** 347 patients were admitted to Smouha University ICU, from which 147 patients died (42.4 %). The mean mortality rate was 43.9 % (28–84.6 %), with mean APACHE II score of 23 (16–30), and mean expected mortality 46.5 % (23.5-70.3). The relative mortality (actual/expected) ranged from 0.35 to 1.25 with mean 0.88 %. Mean Bed occupancy ranged from 4 till 13 beds. After failure of outreach service, bed turnover was similar (2.29 to 2.3). Before and after opening of post-operative ICU, The mortality rate of post-operative cases was higher in post-operative ICU than in general ICU (9.5 vs 2 %, p = 0.019), with lower occupancy rate (4.5 vs 10 patients per month, p = 0.277), and higher mean ICU stay (3.5 vs 1.2 days, p = 0.02). Before and after implementation of proactive ICU, the number of admissions significantly increased from 18 to 33 (p = 0.003), insignificantly higher occupancy rate from 1.8 to 2.8 (p = 0.345), and significantly decreased waiting time for ICU admission from 38 to 6 hours (p < 0.001). However ICU complication increased; unexpected cardiac arrest from 7.5 to 18.5/1000 patient days (p = 0.002), unplanned extubation from 21 to 35/1000 ventilator days (p = 0.305), deep vein thrombosis from 0 to 4.7 % (p = 0.259) and pneumothorax from 3 to 2 % together with increased mortality and relative mortality insignificantly (45.7 to 59 % and 0.89 to 1.13 respectively)

**Conclusions:** From multiple efforts to increase ICU turnover, end of life care decisions and proactive ICU seems most effective, however the expense of increasing nursing and doctor load must be weighted to avoid iatrogenic complications. Also diverting post-operative patients to specialized ICU needed more nurses without better patients outcome.

#### A639 Better “guesstimates” - defining, standardising and documenting safe, accurate and consistent values for actual and ideal body weight in a uk university teaching hospital critical care service

##### B.D. Hammond^1^, G. Gibbon^2^, T. Belcham^2^, K. Burton^2^

###### ^1^Nottingham University Hospitals NHS Trust, Department of Anaesthesia and Critical Care, Nottingham, UK; ^2^Notingham University Hospitals NHS Trust, Department of Anaesthesia and Critical Care, Nottingham, UK

####### **Correspondence:** B.D. Hammond - Nottingham University Hospitals NHS Trust, Department of Anaesthesia and Critical Care, Nottingham, UK

**Introduction:** Knowledge of the critically ill patient's Actual Body Weight (ABW) and Ideal Body Weight (IBW) guide safe treatment in Critical Care (1, 2). When it is not safe or practical to measure these at admission it is necessary to estimate. Our dietician service attends within working hours and uses a systematic method to estimate values if these are unknown. We suspected that ABW and height was inconsistently estimated and documented by various teams.

**Objectives:** We present data from the first project chosen by the NUHCC Better Collaboration, a multi-disciplinary team (MDT) set up to measure and improve the care we offer (3). We aim to define, standardise and improve documentation of safe, accurate and consistent values of ABW and IBW.

**METHOD.**

- Phase 1: Survey current documentation of ABW, IBW and height.
- Phase 2: Survey our MDT for opinion and feedback on a proposed standardised approach to better systematic documentation and estimation of ABW and IBW.
- Phase 3: Reflect on this feedback to present a definition for optimal standard practice in sourcing and documenting safe values.
- Phase 4: Survey performance against the approved standards regularly with planned interventions to improve performance.

**Results:** The initial notes review showed that 32 % of heights and 24 % weights were documented. The majority of respondents to our internal survey agreed that using accurate values was important, that we should define and standardise how we do this and that there was a definite need to improve performance (4). A small MDT group collaborated to define best practice and it was agreed that a standard protocol for measurement and documentation of ABW and IBW would be implemented.

**Conclusion** There is an evident problem with consistency of documentation for safe values of ABW and IBW within NUHCC. We have defined our standard and are excited about implementing and embedding excellent practice as a collaborative effort. We look forward to sharing the data from the first steps of our journey to the ESICM conference.

**References**

1. Kishen R, Blakeley S, Bray K. Standards and Recommendations for the Provision of Renal Replacement Therapy on Intensive Care Units in the United Kingdom. Intensive Care Society; 2009.

2 Grounds M, Snelson C, Whitehouse T, Wilson J, Tulloch L, Linhartova L, et al. Intensive Care Society Review of Best Practice for Analgesia and Sedation in the Critical Care. Intensive Care Society.

3. Burton S, et al. Establishing a Multi-Disciplinary Team to Measure and Deliver Excellent Intensive Care Medicine Within a University Teaching Hospital. To be submitted to Health Education England in the East Midlands Quality Improvement Forum, 2016.

4. Venner C, et al. A Survey of a University Hospital Critical Care Multi-Disciplinary Team Views on Height and Weight Documentation and Systematic Estimation. To be submitted to Mid-Trent Critical Care Network Annual Meeting, November 2016.

### Scoring & prognostication IN ICU

#### A640 Sabadell score as a predictor of outcome after discharge from the intensive care: a prospective observational study

##### L.U. Taniguchi^1,2^, F.J.S. Ramos^3^, A.K. Momma^3^, A.P.R. Martins-Filho^3^, J.J. Bartocci^3^, M.F.D. Lopes^3^, M.H. Sad^3^, C.M. Rodrigues^3^, E.M.C. Pires^3^, J.M. Vieira Jr^3^

###### ^1^Education and Research Institute, Hospital Sírio-Libanês, Intensive Care Unit, São Paulo, Brazil; ^2^Hospital das Clínicas, Universidade de São Paulo, Emergency Medicine Discipline, São Paulo, Brazil; ^3^Education and Research Institute, Hospital Sírio-Libanês, Intensive Care Unit, Sao Paulo, Brazil

####### **Correspondence:** L.U. Taniguchi - Hospital das Clínicas, Universidade de São Paulo, Emergency Medicine Discipline, São Paulo, Brazil

**Introduction:** Despite initial recovery, many critically ill patients discharged from the intensive care unit (ICU) may experience deterioration^1^. Early identification of patients at risk might facilitate improvements in quality of care.

**Objective:** To determine risk factors associated with short-term hospital outcome (unplanned ICU readmission and unexpected death on the ward).

**Methods:** Prospective cohort study from August 2014 to May 2015 performed at Hospital Sírio Libanês (São Paulo, Brazil). During this period, we analyzed 527 patients who were admitted in our ICU and discharged alive. We used univariate and multivariate analysis to identify risk factors associated with latter ICU readmission or unexpected ward death in the same hospitalization period.

**Results:** Forty seven patients (8.9 %) were readmitted after ICU discharge and further forty seven died in the ward. Patients who had unexpected outcomes were older compared to those with successful outcomes (age 72 ± 17 vs 66 ± 18 respectively, p = 0.003), were sicker (SAPS 3 of 53 [IQR 43–60] vs 38 [29–49] respectively, p < 0.001), male sex (62.8 % vs 47.1 % respectively, p = 0.006), had a non-surgical reason for hospitalization (75.5 % vs 45 % respectively, p < 0.001), more frequently came from the ward (21.3 % vs 7.6 % respectively, p < 0.001), were less frequently independent for daily activities (54.3 % vs 76.7 % respectively, p < 0.001), had sepsis (39.4 % vs 11.8 % respectively, p < 0.001), required mechanical ventilation (44.7 % vs 14.1 % respectively, p < 0.001), vasoactive drugs (54.3 % vs 24.2 % respectively, p < 0.001) and dialysis (16 % vs 1.2 % respectively, p < 0.001), had higher SOFA score both at ICU admission (4 [2–7] vs 1 [0–3] respectively, p < 0.001) and discharge (2 [1–4] vs 1 [0–2] respectively, p < 0.001), had higher C-reactive protein values at admission (5.8 mg/dL [1.9 - 15.6] vs 3.6 [0.8 - 8.6] respectively, p < 0.001) and lactate values (1.9 mmoll/L [1.2 - 3.7] vs 1.7 [1.1 - 2.5] respectively, p = 0.03), and higher Sabadell score at discharge (1 [1–2] vs 0 [0–1] respectively, p < 0.001). On multivariate analysis, only sepsis and Sabadell score were independent risk factors for worse outcomes (AUC 0.73; p < 0.001).

**Conclusions:** Sabadell score at ICU discharge might be a promising subjective tool for hospital outcomes.

**References**

1. Rodrigues CM, Pires EMC, Feliciano JPO, et al. Admission factors associated with intensive care unit readmission in critically ill oncohematological patients: a retrospective cohort study. Rev Bras Ter Intensiva 2016;28:33–39.

#### A641 Six years' experience of a brazilian multiprofessional post-icu follow-up clinic

##### M.A. Leite, L.D. Murbach, E.F. Osaku, J. Barreto, S.T. Duarte, S. Taba, D. Miglioranza, D.P. Gund, C.F. Lordani, C.R.L.M. Costa, S.M. Ogasawara, A.C. Jorge, P.A.D. Duarte

###### Western Parana State University Hospital, Cascavel, Brazil

####### **Correspondence:** M.A. Leite - Western Parana State University Hospital, Cascavel, Brazil

**Introduction:** Survivors of ICU admission can develop several complications, such as physical impairment, weight loss, psychological and social disorders, with potential impact on quality of life. Little has been described on this evaluation in low-income countries.

**Objectives:** To describe the results of six years from a follow-up outpatient Clinic evaluating adult patients that survived to admission to ICU, three months after discharge.

**Methods:** Prospective cohort study evaluating adult patients that survived after admission to a general ICU of a University Hospital in Southern Brazil. It was included patients that stayed > 24 h in ICU and > 18 years old. All survivors were contacted by Clinic, and only were excluded that patients that did not attend to the Clinic. Socio-demographic and medical data were collected by searching the medical record. For the psychological evaluation it was used the *Hospital Anxiety and Depression Scale* - (HADS) and the *Impact of Event Scale-Revised -* (IES-R). It was analyzed weight and height on hospital admission and discharge, as well as at the office visit. Muscle strength was measured by *Medical Research Council* (MRC) scale. Spirometry was performed by using One-flow device, with standard procedures.

**Results:** During the period of the study (jul-2008 to dec-2014), 2615 patients were admitted to adult ICU, and 1845 were discharged alive from hospital. Of them, 696 were evaluated at the outpatient multiprofessional Post-ICU Clinic three months after discharge. 62.9 % of the patients were male, mean age 43.1 years, mean APACHE 20.4. The ICU admission was due to trauma (42.1 %), medical conditions (26.2 %) and postoperative (31.7 %). Mean ICU length of stay was 10.3 days. The most of the patients (83.3 %) required mechanical ventilation. In outpatient evaluation, 94.6 % were on oral feeding, mean weight loss of 11.7 % during hospital length of stay, and a mean weight gain of 9.1 % after discharge. 31.8 % had reduced muscle strength, with 4.9 % hemiparesis/hemiplegia and 5.7 % moderate/severe tetraparesia or tetraplegia. Spirometry was abnormal in 49 %, being more common obstructive disorders (37.8 %). At psychological evaluation, 45.2 % showed emotional disorders: anxiety (30.6 %); depression (21.4 %) and PTSD (19.5 %). 47,7 % had a family income lesser than US$550.00/month. It was found significant social and economical consequences for the most of the survivors and their families.

**Conclusions:** A large proportion of patients had physical, social and psychological complications three months after ICU discharge. Thus, early identification and appropriate treatment of these complications may be beneficial. The social and economical impact of ICU stay is to be determined in low-income populations.

#### A642 Examining fatigue in ICU survivors: validity and reliability of a modified version of FACIT-F scale

##### S. Spadaro, M. Capuzzo, F. Dalla Corte, S. Terranova, G. Scaramuzzo, A. Fogagnolo, S. Bertacchini, A. Bellonzi, R. Ragazzi, C.A. Volta

###### University of Ferrara, Department of Morphology, Surgery and Experimental Medicine, Ferrara, Italy

####### **Correspondence:** S. Spadaro - University of Ferrara, Department of Morphology, Surgery and Experimental Medicine, Ferrara, Italy

**Introduction:** A recent survey on the long-term complications reported by the General Practitioners of 1-year ICU survivors showed that chronic fatigue was among the complications most often reported [1]. One of the instruments proposed to assess fatigue is the Functional Assessment Chronic Illness Therapy-Fatigue (FACIT-F), a 13-item scale that has been validated in a sample of the general USA population [2]. Al-shair et al. [3] who used FACIT-F (13-FACIT-F) questionnaire in 2107 COPD patients, recently proposed a modified 9-item FACIT-F (9-FACIT-F) for these patients.

**Objectives:** The aim of this study was to compare reliability and validity of 13-FACIT-F and 9-FACIT-F in a group of ICU survivors assessed one year after hospital discharge.

**Methods:** The study was performed in 56 consecutive adult ICU patients staying in ICU for at least 72 hours, and able to co-operate at the time of ICU discharge. We excluded patients with pre-existing cognitive deficits, or resident more than 30 km from the hospital. One year after hospital discharge, the patients who consented came to the hospital. An investigator administered by direct interview the following instruments to the patients who consented: 13-FACIT-F, modified Medical Research Council (mMRC) Dyspnea scale and the Medical Outcomes Survey Short Form-36 (SF-36). An additional question asked whether the patient's fatigue was the same, improved, or worsened in comparison with the pre-ICU time. Statistical analysis was performed using Cronbach alpha for reliability, Spearman correlation between FACIT-F scales and both mMRC and Vitality of SF-36 for validity, and Mann–Whitney test in patients with same/improved, or worsened fatigue for discriminative ability of both 13-FACIT-F and 9-FACIT-F scales.

**Results:** 13-FACIT-F had higher internal consistency (α = 0.813) compared to 9-FACIT-F (α = 0.654). The correlation between the original and the modified scales was excellent (Table 54). The correlations between both scales and mMRC, and Vitality of SF-36 were all statistically significant (p < 0.001) (Table 54). Both 13-FACIT-F and 9-FACIT-F values were statistically significantly different in patients with improved/not changed and worsened fatigue in comparison with one year before (Table 55).

**Conclusions:** Both 13-item and 9-item FACIT-F scales demonstrate convergence with other conceptually relevant patient-reported outcomes, namely mMRC and Vitality of SF-36. The present study demonstrates that 13-FACIT-F and 9-FACIT-F have a similar ability in discriminating between ICU survivors with improved/not changed and worsened fatigue at one year. However, the original 13-item FACIT-F scale may have the advantage to allow comparison with the general population.

**References**

1. Steenbergen S et al. BMC Anesthesiol 15:142, 2015.

2. Cella D et al. Cancer 94:528–38, 2002.

3. Al-shair K et al. Health Qual Life Outcomes 10:100, 2012.

**Table 54 (abstract A642).** **ᅟ**

Cronbach alpha	Present study	Al-shair et al. [3]
13-FACIT-F	0.813	0.92
9-FACIT-F	0.654	0.91
Spearman CC	Present study	Al-shair et al. [3]
13-FACIT-F vs 9-FACIT-F	0.990	0.99
13-FACIT-F vs mMRC	−0.595	−0.48
9-FACIT-F vs mMRC	−0.606	−0.47
13-FACIT-F vs VT	0.531	
9-FACIT-F vs VT	0.525

**Table 55 (abstract A642).** ᅟ

Fatigue comparison at 1-y	Improved/not changed	Worsened	p Mann-Whitney
13-FACIT-F	47.50 [38.50–50.00]	38.00 [29.75–45.75]	0.001
9-FACIT-F	32.50 [27.00–34.00]	25.00 [19.75–31.00]	0.001

#### A643 What is the goal of the follow up outpatient clinic after intensive care?

##### C. Cruz, A. Nunes, F. Seabra Pereira, I. Aragão, A.F. Cardoso, C. Santos, M.J. Malheiro, H. Castro, T. Cardoso

###### UCIP, Hospital Santo António, Porto, Portugal

####### **Correspondence:** C. Cruz - UCIP, Hospital Santo António, Porto, Portugal

**Introduction:** Follow up outpatient clinic after intensive care (ICU) for has been pointed as a quality indicator of ICU care.

**Objectives:** The objective of this study is to describe the outcomes of the ICU outpatient clinic in terms of clinical management.

**Methods:** Prospective, cohort study, over 4 years (2011–2014), at a 12-bed mixed ICU, form a tertiary care, university affiliated hospital.

**Results:** During the study period 1482 patients were admitted into the ICU and 566 were observed at the outpatient clinic.

555 patients were able to answer to the screening questionnaires and are analyzed. Of these 50 % (277) reported new complains and 30 % (168) had to be oriented for specific interventions and 6 % (33) to more than one. Of these 168 patients: 29 % (n = 48) were oriented to Psychiatry; 15 % (n = 26) to Internal Medicine; 10 % (n = 16) to the Primary Care Physician; 8 % (n = 14) to General Surgery; 8 % (n = 13) to Cardiology; 7 % (n = 12) to Physiotherapy, and 23 % (39) others.

When observed at the outpatient clinic only 230 (44 %) had resumed previous normal activity (34 did not answer the question). Of the 291 that did not, 197 (71 %) assumed to be related to the ICU. No significant differences were observed concerning the timing of the outpatient clinic observation (<2 months, 2–6 months and > 6 months after hospital discharge, p = 0.147) and resuming previous activity.

Of those that were still not able to return to previous activity (n = 291), 194 (67 %) reported new complain and 85 (44 %) needed referral for specific therapeutic approaches.

**Conclusions:** The importance of the ICU follow up clinic is reinforced by the significant proportion of patients in whom new problems are identified requiring specific referral for treatment. The impact of the outpatient clinic in the subsequent quality of life of those patients remains to be determined. We propose for those in whom new clinical problems are addressed a second observation to evaluate its efficacy and impact.

**References**

1. Modrykamien A: The ICU Follow-Up Clinic: A New Paradigm for Intensivists. Respir Care 2012;57(5):764–772

2. Prinjha S, Field K, Rowan K: What patients think about ICU follow-up services: a qualitative Study. *Crit Care* 2009; 13(2): R46

3. Schandl A, Brattström O, Svensson-Raskh A, Hellgren E, Falkenhav M, Sackey P: Screening and treatment of problems after intensive care: A descriptive study of multidisciplinary follow-up. Intensive Crit Care Nurs. 2011 Apr;27(2):94–101

**Fig. 73 (abstract A643).**
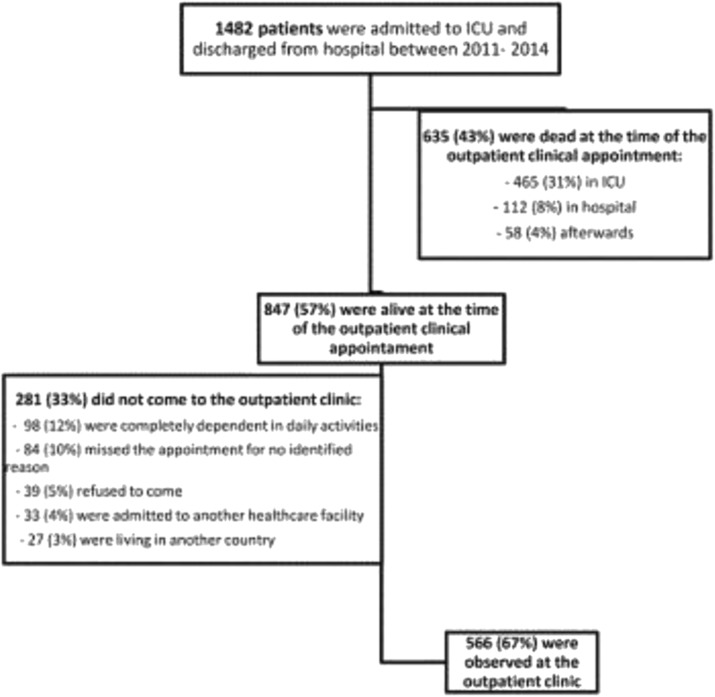
Flowchart of included patients

#### A644 A follow up clinic for sepsis survivors - preliminary results and feasibility

##### J. Paratz^1,2^, J. Kenardy^1^, T. Comans^2^, F. Coyer^3^, P. Thomas^4^, R. Boots^1^

###### ^1^University of Queensland, Brisbane, Australia; ^2^Griffith University, Brisbane, Australia; ^3^Queensland University of Technology, Brisbane, Australia; ^4^Royal Brisbane & Womens Hospital, Brisbane, Australia

####### **Correspondence:** J. Paratz - Griffith University, Brisbane, Australia

**Introduction:** Patients who survive an episode of post admission to intensive care for sepsis syndromes have been found to have poor quality of life, physical and psychological problems and a high rate of recurring illness or mortality in the first year. Follow-up clinics have been instituted for general intensive care patients but the evidence as to efficacy is sparse at present and there has been no clinic specifically for survivors of sepsis.

**Objectives:** The aim of this trial is to investigate if targeted screening and appropriate intervention to these subjects post hospital discharge can result in an improved quality of life, decreased mortality, decreased readmission to hospital and/or decreased use of health resources.

**Methods:** Patients post sepsis syndrome in intensive care and receiving respiratory support for greater than 48 hours will be randomized to one of two groups. The intervention group will attend an outpatient clinic two monthly for six months and receive screening and targeted intervention. Screening will include physical measures (strength, balance) psychosocial measures (depression, anxiety, PTSD), cognitive measures, pain, nutrition or sleep problems. The usual care group will remain under the care of their physician. The primary outcome will be short form (36) health survey (SF36v2Ô), readmission to hospital, mortality in the first 12 months, and use of health resources in the first year. A sub group in a rural setting will be assessed by telemedicine.

**Results:** Twenty patients (9 intervention, 11 usual care) have been randomized. Patients randomised to the intervention group report a number of issues including altered and unpleasant memories from intensive care and confusion about scars. Patients were referred to agencies including physical rehabilitation (3), a chronic pain clinic (1), sleep study (1) and a psychologist (3). All patients have given expressed satisfaction with the content and explanations of the clinic visit. Six subjects have reached the 6 month mark and we have had 100 % attendance in the intervention group and 100 % completion for SF36.

**Conclusions:** We have shown that this clinic is feasible and there is a need for this service.

**References**

1. Paratz JD et al. IMPOSE (IMProving Outcomes after Sepsis)-the effect of a multidisciplinary follow-up service on health-related quality of life in patients post sepsis syndromes-a double-blinded randomised controlled trial: protocol. BMJ Open. 2014;4(5):e004966. doi: 10.1136/bmjopen-2014-004966

**Grant acknowledgement**

Queensland Government Health and Medical Research Unit

#### A645 Identifying risk factors for post-intensive care syndrome

##### N. Pereira, A. Vilas-Boas, E. Gomes, C. Dias, J. Torres, D. Carvalho, E. Molinos, C. Vales, R. Araújo

###### Hospital Pedro Hispano, Matosinhos, Portugal

####### **Correspondence:** N. Pereira - Hospital Pedro Hispano, Matosinhos, Portugal

**Introduction:** Surviving the ICU is only the beginning of getting life back. The majority of ICU survivors experience immediate and frequently persistent physical, mental and cognitive impairments that compose post-intensive care syndrome (PICS) ^[1–3]^.

The exact prevalence of PICS is unrevealed, but it is estimated that more than half of critical care survivors develop at least one symptom throughout their recovery. Understanding who are the patients more likely to develop PICS is a key issue.

**Objectives:** This study intends to determine the prevalence of PICS in one Portuguese ICU, to characterise this population and identify risk factors to the development of this syndrome.

**Methods:** We performed a prospective cohort study from January 2013 to February 2015. All ICU survivors that were admitted to our unit during two or more days were included. In the first 24 hours of admission patients were screened for the risk to develop physical and non-physical components of PICS. The presence of PICS was assessed at four time points: at ICU discharge, one week after, at hospital discharge and three months after ICU discharge. Additionally, we recorded clinical data on: patients' demographics, type of admission, simplified acute physiology score (SAPS II), length of ICU and total hospital stay, length of sedation and mechanical ventilation.

**Results:** We enrolled 355 patients with a median age of 63 years and a predominance of males (62 %). At ICU admission 87.6 % of the patients had some risk factor for PICS. Throughout time PICS was identified in 186 (54 %) patients at ICU discharge, 148 (58 %) one week after discharge, 78 (55 %) at hospital discharge and on 106 (43 %) patients three months after ICU. At ICU discharge, physical components of PICS were more frequent, and at three month follow up non-physical components were more common. Patients with higher SAPS score, longer length of ICU and hospital stay and more ventilation and sedation days were more prone to have PICS at ICU discharge. These variables were independent risk actors to the development of PICS. At three months follow-up, these same variables, except SAPS II score, were still risk factors of PICS.

**Conclusions:** PICS is a major concern in nowadays intensive care units (ICU), reflecting a drift from the original focus on organ support to patient support. Interventions in reducing the absolute number of ventilation and sedation days might contribute to reduce the burden of PICS.

**References**

[1] Needham D., Davidson J., Cohen H., et al. (2012). Improving long-term outcomes after discharge from intensive care unit: Report from stakeholders' conference. Critical Care Medicine, vol 40, n°2, 502–509

[2] Damm T., Patel J., (2015), Long-term outcomes after critical illness. PulmMCC journal.

[3] Hoffman L., Guttendorf J., (2015), Post Intensive Care Syndrome: Risk factors and prevention strategies. ACHMedia

#### A646 Depression after intensive care: who to blame?depression after intensive care: who to blame?

##### C. Cruz, A. Nunes, F. Seabra Pereira, A.F. Cardoso, C. Santos, M.J. Malheiro, H. Castro, T. Cardoso

###### UCIP, Hospital Santo António, Porto, Portugal

####### **Correspondence:** C. Cruz - UCIP, Hospital Santo António, Porto, Portugal

**Introduction:** Depression has been associated to ICU stay in a fair proportion of patients. But most studies rely solely on the outpatient clinic screening after ICU discharge, without considering previous psychopathology of the patients or specific risk factors, like ICU memories.

**Objectives:** To determine the prevalence of depression after ICU discharge in patients with and without a previous diagnosis of psychopathology and its association with memories from the ICU admission.

**Methods:** Prospective cohort study of all patients seen at the outpatient clinic, discharged between January 2011 and December 2014, from a 12-bed, mixed ICU, at a tertiary care university affiliated hospital. The ICU memory questionnaire and Beck Depression Inventory (BDI) were the tools used, depression was assumed if the BDI score was higher than 16. With depression has the dependent variable: age, sex, previous psychopathy, acute psychiatric event, previous neurologic disorder, acute neurologic event and the presence of memories were studied as risk factors, through logistic regression.

**Results:** During the study period 1482 patients were discharged from the ICU, 635(43 %) died before the outpatient clinic appointment and 566(67 %) attended the clinic, of these 555 filled the BDI and 530 the ICU memory questionnaire. These patients (n = 555) had a mean ± SD age of 58 ± 17 years; 341(61 %) were male; 157(28 %) had a previous diagnose of a psychopathology disorder : 79(14 %) alcoholism, 55(35 %) depression, 16(10 %) psychosis and 7(4 %) drug abuse were the more frequent, 13(8 %) had more than one; 32(6 %) were admitted to ICU due to an acute psychiatric event, only 2 patients without a previous diagnose were admitted with an acute psychiatric event. The prevalence of depression in the group with a previous history of psychopathology was significantly higher than in the group without: 26 %(n = 40) vs 16 %(n = 63), p = 0.008. In the univariate analysis variables significantly associated with depression after ICU were: previous psychopathology (OR1.82), acute psychiatric event (OR3.79) and presence of memories from ICU admission (OR0.66). In the final model those retained has independent risk factors were: acute psychiatric event [adjusted OR(95 % CI) = 4.30(2.01-9.21)] and presence of memories from the ICU admission [adjusted OR(95 % CI) = 0.63(0.40-0.98)].

**Conclusions:** Depression after ICU is more prevalent among patients that have previous psychopathology, recalling that the ICU stay is associated with a reduced risk for after ICU depression. We suggest that when patients are admitted to ICU the relatives fill in a questionnaire about the previous cognitive, psychologic and functional status of the patient in order to allow clinicians to determine the true impact of ICU care. The elaboration of a diary to decrease post-traumatic stress disorder following ICU has already been study, but it would be interesting to see if that would have a positive impact in the prevalence of depression after ICU care.

#### A647 Emotional and cognitive sequelae of medical-surgical ICU care

##### L. Karnatovskaia, K. Philbrick, G. Ognjen, M. Clark

###### Mayo Clinic, Rochester, USA

####### **Correspondence:** L. Karnatovskaia - Mayo Clinic, Rochester, USA

**Introduction:** Many intensive care unit (ICU) patients experience symptoms of depression, anxiety, and posttraumatic stress along with cognitive impairment and physical debility. This combination of symptoms has been classified as post-intensive care syndrome (PICS). Over half of ICU survivors are reported to have significant psychiatric symptoms which appear to diminish little over time. Post-ICU intervention efforts may fail due to delayed initiation, i.e., following hospital discharge after patients' perceptions of their ICU experience has potentially solidified. Currently work is being done to prevent physical aspects of PICS via early physical rehabilitation while still in the ICU. However, there are no evidence based practical interventions to prevent or treat the psychological and cognitive consequences of the ICU stay. It is also not known if there may be categories of patients who are more susceptible to PICS, and would therefore benefit most from tailored treatments.

**Objectives:** The aim of this study was to assess the prevalence of psychocognitive morbidity among patients across six adult ICUs.

**Methods:** Within 96 hours of dismissal from the ICU, eligible patients completed the Hospital Anxiety and Depression Scale (HADS; scores ≥ 8 indicating significant symptoms of anxiety or depression), Impact of Events Scale-Revised (IES-R; scores ≥ 1.6 indicating significant posttraumatic stress disorder (PTSD) symptoms), and Montreal Cognitive Assessment-Blind (MoCA-blind; scores < 18 indicating cognitive impairment). Inclusion: >18 years old; ICU stay of >48 hours; GCS > 13, CAM-ICU negative, ≤ 2 errors on the 6-item Cognitive Screener. Exclusion: admitted to the ICU for suicide attempt; known prior cognitive impairment/dementia; prior diagnosis of PTSD; non-English speaking.

**Results:** 107 patients completed the assessment. Table 56 illustrates prevalence of psychocognitive symptoms by the ICU type.

**Conclusions:** There was a high prevalence of symptoms of depression (range 17-69 %), anxiety (range 18-58 %) and PTSD (range 18-58 %). In terms of predictors of distress, patients in the heme-onc/transplant ICU had a much lower prevalence of symptoms of depression, anxiety and PTSD compared to patients in the other ICU units. Whether these patients experience the ICU differently by virtue of the chronicity of their diseases and the contextual circumstances of their ICU admissions invites further study. There was also a high level of cognitive impairment across the ICUs (range 45-83 %). Clearly more needs to be learned about patient and unit factors that are related to psychological difficulties, and tailored interventions will need to be appropriate for individuals with cognitive impairments.

**References**

1. Bienvenu OJ et al. Cooccurrence of and remission from general anxiety, depression, and posttraumatic stress disorder symptoms after acute lung injury: a 2-year longitudinal study. Crit Care Med. 2015;43(3):642–53.

**Table 56 (abstract A647).** Psychocognitive data by ICU

ICU type	N patients	HADS-D≥8 (%)	HADS-A≥8 (%)	IES-R≥1.6 (%)	MOCA-blind<18 (%)
Heme-onc/ transplant MICU	11	18	18	18	45
Cardiac MICU	21	38	57	19	75
Cardiothoracic SICU	19	33	58	53	74
MICU	31	33	42	39	68
Trauma SICU	12	17	58	58	83
Cardiovascular SICU	13	69	54	46	51

#### A648 Provent score validation in intra-ICU mortality in patients with prolonged admission

##### R. Molina Montero, J. Luján Varas, L. Alcázar Sánchez-Elvira, C. Pintado Delgado, P. Villa Díaz, B. Llorente Ruiz, A. Pardo Guerrero, J.A. Cambronero Galache

###### Hospital Universitario Príncipe de Asturias, Alcalá de Henares, Madrid, Spain

####### **Correspondence:** R. Molina Montero - Hospital Universitario Príncipe de Asturias, Alcalá de Henares, Madrid, Spain

**Introduction:** Prolonged ICU admission is associated with high mortality. Provent score at day 21 has been proven effective as a prognostic index of mortality one year after prolonged admission in ICU in patients requiring prolonged mechanical ventilation (over 20 days).

**Objective:** Assesment of Provent Score as prognostic score in ICU mortality in patients with prolonged ICU stay (over 20 days).

**Methods:** Prospective descriptive study. During two years, we included all patients with more than 20 days of stay in a medical-surgical ICU. Previous informed consent, we collected demographics data, diagnosis, severity index (APACHE II and SOFA scores), Provent score variables at day 21 of ICU admission (age, platelets < 150.000/mm^3,^, need of vasoactive support and renal replacement therapy), PaO2/FiO2 at day 21, mortality and ICU stay.

Data is expressed as number and percentage; statistical analysis was performed with Chi-square test.

**Results:** A total of 58 patients with prolonged ICU admission were included. We did not find differences in age, sex, comorbidity or severity of illness at admission between survivors and deceased in ICU. At day 21, 33 % of the patients needed vasoactive support (72 % of deceased and 15 % of the survivors, p < 0.001), 22 % had thrombocytopenia (50 % of deceased and 10 % survivors, p = 0.002), 22 % needed renal replacement therapy (33 % of deceased and 17 % survivors, p = 0.195) and 29.3 % presented PaO 2 / FiO 2 less or equal to 200 mmHg (55.6 % of deceased and 16.7 % survivors, p = 0.003). ICU mortality was 31 %.

**Conclusions:** High Provent Score (4–5) or PaO 2 / FiO 2 below 200 mmHg.

at day 21 of admission is associated with increased intra-ICU mortality in patients with prolonged admission .

**References**

- Carson SS, Kahn JM, Hough CL et al. A multicenter mortality prediction model for patients receiving prolonged mechanical ventilation. Crit Care Med. 2012;40:1171–1176.

- Hortiguela-Martin VA, Sanchez-Casado M, Rodriguez-Villar S et al. [Post-Intensive Care Unit mortality and related prognostic factors in a cohort of critically ill patients with multi-organ dysfunction]. Med Clin (Barc). 2013;140:479–486.

- Laupland KB, Kirkpatrick AW, Kortbeek JB, Zuege DJ. Long-term mortality outcome associated with prolonged admission to the ICU. Chest. 2006;129:954–959. 013;140:479–486.

**Table 57 (abstract A648).** ICU mortality and Provent

	Survivors	Deceased	p
ICU mortality	36	18	
Provent			
0	5 (13.5%)	1(5.6%)	0.651
1	5 (13.5%)	1(5.6%)	0.651
2	16 (44.4%)	3 (16.7%)	0.044
3	9 (25%)	6(33.3%)	0.519
4-5	1 (2.8%)	7 (38.9%)	0.001
PaO2/FiO2 < 200	6 (18.8%)	10 (55.6%)	0.007

#### A649 Evaluation of acute physiology and chronic health evaluation II and simplified acute physiology score II prediction of hospital mortality in chronically critically ill patients

##### R. Jiménez, S. Rebollo, O. Alejandro, A. Fernández, S. Moreno, L. Herrera, A. Ojados, M. Galindo, J. Murcia, M. Contreras, S. Sánchez-Argente, Y. Bonilla, M.D. Rodríguez, J.M. Allegue

###### Hospital Universitario Santa Lucía, Intensive Care Unit, Cartagena, Spain

####### **Correspondence:** R. Jiménez - Hospital Universitario Santa Lucía, Intensive Care Unit, Cartagena, Spain

**Introduction:** Patients surviving the acute phases of critical illness, complications as polyneuropathy and prolonged mechanical ventilation can appear. A high level of uncertainty exists regarding to prognostication of outcomes in these patients. Chronical critical illness is usually considered when invasive mechanical ventilation is prolonged for at least 21 days after acute illness.

**Objectives:** To study the ability of Acute Physiology and Chronic Health Evaluation (APACHE) II and Simplified Acute Physiology Score (SAPS) II to predict outcome in our chronically critically ill patients.

**Methods:** We performed a retrospective analysis of patients admitted to our medical-surgical ICU in the last 5 years. Differences in basal characteristics and outcomes and data regarding APACHE II and SAPS II predictive value were studied in two cohort of patients according to the need for more than 21 days of mechanical. For univariate analysis Chi-square test, t-Student and U-Mann–Whitney test was used. Receiver Operating Characteristic (COR) curves was used to study predictive ability of scores.

**Results:** A total of 2671 patients were admitted. Of them, 85 required more than 21 days of mechanical ventilation (chronic).

Chronic patients were younger (65 (51.5-73) vs 68 (54–77); p 0.036), had higher APACHE II (21 (15.25-25) vs 16 (12–22); p < 0.001) and SAPS II (48.5 (37–56) vs 40 (30–53); p < 0.001), same Glasgow scale at admission and longer ICU stay (38.5 (30–52.5) vs 4 (2–6); p < 0.001) and hospital stay (56.5 (37.25-80.5) vs 10 (5–18); p < 0.001) Mortality was higher in prolonged mechanical ventilation patients (33.3 vs 18.7; p 0.002). Chronic patients had higher incidence of inmunodepression, COPD, neoplasia and malnutrition at admission (statiscally significant). Required urgent surgery in higher proportion (22.4 vs 11.6 %; p 0.004), more renal support (30.6 vs 5.7 %; p < 0.001) and more parenteral nutrition (34.1 vs 7.3 %; p < 0.001) Admission from ward was more frequent in chronic patients (56.5 vs 41.1 %; p < 0.001). Table 58 shows ROC curves data. AUC differences resulted slightly lower in medical patients [AUC 0.724 (0.588-0.860; p 0–006) vs 0.816 (0.793-0.838; p < 0.001)] and were more pronounced in surgical patients [AUC 0.641 (0.415-0.867; p 0.270) in chronic vs 0.833 (0.789-0.876; p < 0.001)].

**Conclusions:** APACHE II and SAPS II showed worse predictive accuracy for hospital mortality in patients requiring prolonged mechanical ventilation after the acute phase, especially in surgical admissions.

**References**

- Nelson et al. Am J Respir Crit Care Med Vol 182. pp 446–454, 2010

- Dematte D'Amico. Chest 2003;124: 1039–1045.

**Table 58 (abstract A649).** ROC curves for APACHE II and SAPS II

		AUC	CI 95%	p
APACHE II	Chronicl	0.702	0.586–0.817	0.003
	Non-chronic	0.820	0.800–0.839	<0.001
SAPS II	Chronic	0.641	0.415–0.867	0.348
	Non-Chronic	0.828	0.782–0.875	<0.001

#### A650 The prognostic role of scoring systems following liver transplantation

##### Ö. Cakin, H. Parlak, H. Kirca, F. Mutlu, B. Aydınlı, M. Cengiz, A. Ramazanoglu

###### Akdeniz University, Antalya, Turkey

####### **Correspondence:** Ö. Cakin - Akdeniz University, Antalya, Turkey

**Introduction:** Liver transplantation patients generally are followed up in intensive care unit (ICU) for postoperative period. Prognosis of those patients can be predicted with some scoring systems.

**Objectives:** The aim of the study was to evaluate the prognostic efficacy of physiological scoring systems obtained at first postoperative day after liver transplantation on ICU stay, mortality and mechanical ventilation(MV) duration.

**Methods:** Postoperative first day acute physiology and chronic health evaluation II (APACHE II), Sequential Organ Failure Assessment (SOFA), Model of End Stage Liver Disease (MELD) scores, ICU stay and early (28 days) of the liver transplant patients admitted between the years 2010–2016 to Akdeniz University ICU were evaluated, retrospectively. Ethic consent was obtained from local ethic committee.

**Results:** Record files of 119 liver transplant patients admitted to ICU during study period were evaluated. Postoperative early ICU mortality was 15.1 %. Mean MV duration, APACHE II score, SOFA score, MELD score were found 5 hours (1–168), 9(3–29), 6(2–16) and 18(5–31), respectively. APACHE and SOFA scores of the patients whose ICU stay were longer than 35 hours were significantly higher (p < 0.05). APACHE and SOFA scores were inversely correlated with early ICU mortality (p < 0.001). MELD score did not have significant prognostic value on ICU stay and early ICU mortality. Independent risk factors effective on mortality were evaluated by logistic regression test indicating APACHE and blood urea nitrogen(BUN) value as independent risk factors in terms of mortality.

**Conclusions:** There are conflicting results in terms of availability of generally used physiological scoring systems for predicting the prognosis after liver transplantation(3). Significant number of studies has results confirming the prognostic value of SOFA, APACHE II and MELD scores in ICU stay, MV duration and mortality. In our study, early mortality and ICU stay were higher in the liver transplant patients who have higher APACHE and SOFA scores obtained at first day of ICU admission, postoperatively(2,3). APACHE II and SOFA scoring systems were found to have prognostic efficacy on ICU stay and early mortality after liver transplantation(3). However, we found no correlation between MELD score and mortality(1). The discrepancy may result from the quality of liver graft as 70 % of the donation was performed from live organ donors in our study.

**References**

1- Oberkofler CE, Dutkowski P, et al. Model of end stage liver disease (MELD) score greater than 23 predicts length of stay in the ICU but not mortality in liver transplant recipients. Crit Care 2010;14:117

2- Santoyo J, Suarez MA, et al. True impact of the indication of cirrhosis and the MELD on the results of liver transplantation. Transplant Proc 2006;38:2462–4.

3- Pan H-C, Jenq C-C, et al. Scoring Systems for Predicting Mortality after Liver Transplantation. PLoS One 2014; 9: e107138

#### A651 Comparison of apache IV with other scoring systems in the ICU in predicting in-hospital mortality after liver transplantation

##### E.-J. Jung^1^, S.-Y. Oh^2^, H. Lee^1^

###### ^1^Seoul National University Hospital, Anesthesiology and Critical Care, Seoul, Republic of Korea; ^2^Seoul National University Hospital, Surgery, Seoul, Republic of Korea

####### **Correspondence:** E.-J. Jung - Seoul National University Hospital, Anesthesiology and Critical Care, Seoul, Republic of Korea

**Introduction:** Several prognostic systems have been tested to predict early in-hospital mortality after liver transplantation, showing moderate performance. The recently developed Acute Physiology and Chronic Health Evaluation (APACHE) IV score and Simplified Acute Physiology Score (SAPS) 3 now include liver transplantation as a diagnostic category.

**Objectives:** The aim of this study was to compare the performance of APACHE IV, SAPS 3, APACHEII, MELD, MELD-Na, and CTP scores in predicting early in-hospital mortality in liver transplant patients.

**Methods:** We studied 590 adult (age ≥ 18 years) patients who had undergone living donor or deceased donor liver transplantation between October 2010 and September 2014. APACHE II, APACHE IV and SAPS 3 were recorded on ICU admission after liver transplantation. MELD, MELD-Na, and CTP scores were calculated using values before liver transplantation.

**Results:** The overall in-hospital mortality rate was 2.9 %. Hosmer-Lemeshow statistics showed good calibration for all 6 prognostic models. The AUC was 0.83 for APACHE IV, 0.78 for SAPS 3, 0.81 for APACHE II, 0.72 for MELD-Na, 0.76 for MELD, and 0.68 for CTP score.

**Conclusions:** The APACHE IV score and SAPS 3 in predicting in-hospital mortality after liver transplantation showed revealed generally good discrimination and calibration. The overall discrimination and calibration of APACHE IV were similar to those of all other models except CTP score for predicting hospital mortality.

**References**

1. Moreno RP, Metnitz PG, Almeida E, Jordan B, Bauer P, Campos RA et al. SAPS 3--From evaluation of the patient to evaluation of the intensive care unit. Part 2: Development of a prognostic model for hospital mortality at ICU admission. Intensive Care Med 2005; 31: 1345–1355.

2. Wan P, Yu X, Xia Q. Operative outcomes of adult living donor liver transplantation and deceased donor liver transplantation: a systematic review and meta-analysis. Liver Transpl 2014; 20: 425–436.

#### A652 Validation of SAPS 3 and SOFA in the prognosis of cardiac patients admitted to Brazilian intensive care unit

##### N.M. Filgueiras Filho^1,2^, E.F. Ricaldi^1,2^, S.S. Gomes^1,2^, B.B. Ramos^1,2^, C.V. De Lucia^1,2^, C.S. Ballalai^1,2^, J.C.A. Oliveira^1^, G.P. Araponga^1^, L.N. Veiga^1^, C.S. Silva^1,2^, M.E. Garrido^1^, GEMINI

###### ^1^Núcleo de Ensino e Pesquisa Hospital da Cidade, Intensive Care, Salvador, Brazil; ^2^Universidade Salvador - UNIFACS, Núcleo de Pesquisa Clínica, Salvador, Brazil

####### **Correspondence:** N.M. Filgueiras Filho - Universidade Salvador - UNIFACS, Núcleo de Pesquisa Clínica, Salvador, Brazil

**Introduction:** The scores have been widely used in the Intensive Care and Cardiac Units. The SOFA initially used for the daily assessment of organ dysfunction in septic patients, was subsequently validated for serious patients in general, as a way of assessing daily dysfunction. The prognosis system SAPS3 (Simplified Acute Physiology Score 3) aims to establish a predictive mortality rate for patients admitted to Intensive Care Units (ICU).

**Objectives:** To evaluate the accuracy of the SAPS 3 and SOFA scores in the prognosis of cardiac patients admitted to the ICU of a general hospital in northeast region of Brazil.

**Methods:** Prospective cohort study placed in a general ICU. In the study were included 54 patients with cardiac disease of 314 patients admitted patients to general ICU, from August 2015 and March 2016. All the patients were assessed using the SAPS 3 (Global Equation and Central & South American Equation) and SOFA scoring systems. Logistic regressions analysis was used to calculate the sensitivity, specificity and accuracy as well as the OR probability of mortality. The ability to predict mortality was performed with ROC curves. The differences between observed-to-predicted mortality were analyzed with the Hosmer-Lemeshow test.

**Results:** The study population had the following general characteristics: age 67 ± 15.3 years, men 51.9 %, with a median time of 6 days of hospitalization (IQR = 2.5-10.5). The admission reason was clinical in 85.2 % of patients (15.4 % NSTEMI, STEMI 3.9 %, 7.7 % UA, 23.1 % Heart Failure, Acute lung edema 11.5 %). The median (in points) in SAPS 3 score was 43 (CI = 38.0-49.8) and SOFA was 0 (IQR = 0–3.0) and the area under the ROC curve was 0.93 (CI 95 % = 0.85-1.00) and 0.95 (CI 95 % = 0.89-1.00), respectively. Calibration was confirmed by the Hosmer-Lemeshow test for SAPS 3-X2 = 0.1565 df: 8 (p = 0.99). Sensitivity to the SAPS 3 score and SOFA was 81.82 % and 100 %, and the specificity of the scores was 94.12 % and 79.41 % respectively. The corresponding negative predictive value to scores SAPS 3 and SOFA was 94.12 % (CI 95 % = 76.81-99.28) and 100 % (CI 95 % = 86.03-100.00). The corresponding positive predictive value to scores SAPS 3 was 84.12 % (CI 95 % = 53.45-97.72).

**Conclusions:** The results showed that the scores can be effective in the prognostic assessment of cardiac patients admitted to a general ICU, with good calibration and adequate discrimination value.

#### A653 SAPS 3 score and grace index. Mortality calibration analysis in acute coronary sindrome patients admitted in ICU. Correlation between both scores

##### J. Cebrián Domenech, A. Pinos Montalvo, T. Ciges Chornet, P. Concha Martinez, M. Piñol Ribas, R. Gimeno Costa, A. Castellanos Ortega

###### Hospital Universitario y Politécnico La Fe, ICU, Valencia, Spain

####### **Correspondence:** J. Cebrián Domenech - Hospital Universitario y Politécnico La Fe, ICU, Valencia, Spain

**Introduction:** Saps 3 is widely used in intensive care units (ICU). However their use among the acute coronary patiens remains controversial.

**Objectives:** To compare, in mortality calibration terms, a general ICU severity index (Saps 3) with a specific one (Grace) designed for coronary patients in a population of acute coronary syndromes (ACS) admitted in our ICU. In addition to build a correlation equation between both scores.

**Methods:** During the period 1/1/2015 to 30/9/2015 a total of 287 patients with ACS were admitted in our ICU. Predicted Saps 3 mortality is systematically recorded in our electronic clinical record. Grace score was obtained by retrospective carefully revision of medical records according to their authors recommendations. Saps 3 death risk was derived by Mediterranean country index equation while Grace death risk was obtained from original score variables by using Granger's regression coefficients. We compared in-hospital observed mortality with theoretical predicted mortality for both scores. We have used a goodness of fit test. We used Pearson's R analysis for correlation purposes.

**Results:** Mean age was 63.2 years (SD 13.6) and 24 % were women. Mean Saps 3 score was 44.1 (SD 7.8) while mean Grace index was 152.8 (DE 37.6) Estimated mortality by Saps 3 and Grace were 12.0 % and 5.1 % respectively. Observed in-ICU mortality was 3.1 % (95 % CI 1.7-5.9) and in-hospital 5.2 % (95 % CI 3.2-8.4) Regarding correlation analysis Grace and Saps 3 scores are linked by the following equation: **Grace = 27.01 + 2.86 x Saps 3 (R**^**2**^ **= 0,34; p < 0.001)**.

**Conclusions:** Mortality of ACS patients in our ICU is clearly overestimated by Saps 3 score. It seems more appropriate to use the Grace specific index in this subgroup of our case-mix. It ist feasible the use of a correlation equation between both scores.

### Airway management in ICU

#### A654 “Airway aware” measuring and improving multi-disciplinary team awareness of a safe response to airway and tracheostomy crises across critical care units in two British university teaching hospitals

##### C. Forbes, H. Prescott

###### Nottingham University Hospitals NHS Trust, Adult Critical Care, Nottingham, UK

####### **Correspondence:** C. Forbes - Nottingham University Hospitals NHS Trust, Adult Critical Care, Nottingham, UK

**Introduction:** The use of temporary tracheostomies to avoid complications of prolonged intubation and facilitate respiratory weaning is a well recognised cornerstone of critical care medicine, but their insertion and subsequent management is not without risk. Tracheostomy displacement is one of the most serious and frequent of all airway events in critical care and is associated with 50 % of airway related deaths in this setting^1,2^. As such a timely emergency action plan is a necessity for critical care areas^1,2,3^.

**Objectives:** We aimed to carry out a service improvement project to improve timely escalation of patients with dislodged tracheostomies to senior airway trained doctors. The project was carried out at two sites: Queen´s Medical Centre (QMC) and Nottingham City Hospital (NCH) of Nottingham University Hospitals NHS Trust, UK.

**Methods:** A rapid cycle audit was undertaken by the authors whereby staff awareness of the local emergency airway protocol was audited on a shift-by-shift basis. All non-airway trained clinical staff were asked how best to get an airway expert to urgently attend a patient with a dislodged tracheostomy. Answers were recorded and at the same time staff were reminded of the local protocol. Other education measures undertaken concurrently included departmental emails, posters and reminders during resuscitation training. A Statistical Process Control chart of percentage correct answers per shift was plotted.

**Results:** Over both sites an improvement in compliance was observed over a 4 month period. Mean compliance for the first 3 audit cycles at QMC and NCH was 38.47 % and 24.2 % respectively versus mean compliance for the final 3 audit cycles of 97.23 % and 50.53 % respectively. At QMC, use of posters after 3 months saw a marked improvement in compliance with all 8 audit cycles undertaken after distribution of the posters exceeding the upper control limit of the preceding 3 months' worth of data and 4 of those 8 cycles seeing greater than 90 % compliance.

**Conclusions:** Improved awareness of the escalation protocol over the 4 month audit period was reflected in its increased utilisation. Use of various educational tools achieved varying results, the most effective being distribution of posters. There is ongoing scope for staff education in order to consistently achieve greater than 90 % compliance at both sites and we would recommend re-audit within 6 months.

**References**

1. Wilkinson K, Freeth H, Kelly K. ´On the right Trach?´ A review of the care received by patients who undergo tracheostomy. Br J Hosp Med (Lond) 2015;76(3):163–5.

2. Cook TM, Woodall N, Harper J, Benger J. Major complications of airway management in the UK: results of the Fourth National Audit Project of the Royal College of Anaesthetists and the Difficult Airway Society. Part 2: Intensive care and emergency departments. Br J Anaesth 2011;106(5):232–42

3. UK National Tracheostomy Safety Project. www.tracheostomy.org.uk

**Grant acknowledgement**

No financial support was received.

#### A655 Ideal length of oral endo-tracheal tube (ETT) for intubated patients in Asian population: comparison to current Western standards

##### A. Lal^1^, F.A. Khan^1^, E.G. Dela Pena^1^, J.S. Dizon^1^, P.P.P. Perez^1^, C.M.J. Wong^2^

###### ^1^Ng Teng Fong General Hospital (Jurong Healthcare), Intensive Care, Singapore, Singapore; ^2^Ng Teng Fong General Hospital (Jurong Healthcare), Clinical Research Unit, Singapore, Singapore

####### **Correspondence:** A. Lal - Ng Teng Fong General Hospital (Jurong Healthcare), Intensive Care, Singapore, Singapore

**Introduction:** The length of oral ETT in males and females has been recommended as 23 cm and 21 cm respectively from corner of mouth to above carina, standards described for Western population. Caucasian population tends to be taller than Asian population. We, thus retrospectively analysed whether it is true in Asian population of smaller stature and whether there is any correlation between height of the patient to the ideal length of oral ETT.

**Objectives:** To validate the ideal length of oral ETT in Asian Population compared to Western standards.

**Methods:** Retrospective analysis of the data for patient episodes, who had oral ETT inserted in the Intensive care unit of Jurong Healthcare, Singapore (Alexandra Hospital) from April 2011 till June 2015. The variables analysed were demographics, height and ideal body weight of the patient, length of oral ETT and chest X rays (for confirmation of position of ETT).

**Results:** There were 923 incidences of oral cuffed ETT insertion above the age of 18 years in 750 patients and their intubation records were analysed.

The median length of oral ETT was 22.2 cm in 600 males(SD 1.26)and mean length in 323 females was 21 cm (SD = 1.46).The mean length in all three ethnic groups of Chinese, Malay and Indians was 22 cm.

The median ETT depth for ICU male patients with acceptable distance from carina (2–5 cm) was 22 cm, (p < 0.0001) different from 23–24 cm but was similar in females at 21 cm (p < 0.66).

**Conclusions:** The local population in Singapore is representative of 3 major ethnic groups (Malay, Chinese and Indians) which covers almost 2/3 population of Asia. They are generally of shorter stature in comparison to western population especially Caucasians. It has been well documented that the ETT moves downwards towards carina by at least 2 cm in flexion of neck and upwards by 2 cm in extension. This plays an important role while securing ETTs especially in Intensive care patients, as they tend to be intubated for an average of 3–5 days and require lot of movements during day to day care. Either securing the tube too low or too high can cause either right sided endo-bronchial intubation or high risk of accidental extubation respectively. These inadvertent impediments predispose already critically ill patients to significant risks and complications. We suggest that females and males in Asian population especially in South East Asia should have their endo-tracheal tubes secured at corner of mouth by at least one cm less in comparison to western population.

**References**

1. Chen-Hwan Cherng, et al.Airway length in adults: estimation of the optimal ETT length for orotracheal intubation. Journal of Clinical Anaesth. Volume 14, Issue 4, June 2002, Pages 271–274

2. Anu Varshney et al.Appropriate depth of placement of oral ETT and its possible determinants in Indian adult patients. Indian J Anaesth. 2011 Sep-Oct; 55(5): 488–493

**Table 59 (abstract A655).** Demographics 1

VARIABLES	MALE (N = 491)	FEMALE (N = 281)
Mean Age in Years (SD)	62.9 (16.4)	67.4 (15.6)
Mean Height in cm (SD)	166.9 (8.7)	154.5 (7.6)

**Table 60 (abstract A655).** Demographics 2

Ethnicity	Male (N = 491)	Female (N = 281)
Chinese	340 (69%)	178 (63%)
Indian	45 (9%)	31 (11%)
Malay	60 (12%)	45 (16%)
Others	46 (9%)	27 (10%)

**Table 61 (abstract A655).** Depth of ETT versus indication of intubation

Distance of Tip of ETT from Carina	Respiratory failure (N = 313)	Collapse (N = 105)	Post operative (N = 285)	Others (N = 195)
Less than 2 CM	37 (12%)	15 (14%)	33 (12%)	27 (14%)
2-5 CM (acceptable range)	192 (61%)	64 (61%)	164 (58%)	112 (57%)
More than 5 CM	76 (24%)	23 (22%)	83 (29%)	52 (27%)
Right or left main bronchus	8 (3%)	3 (3%)	5 (2%)	4 (2%)

**Fig. 74 (abstract A655):**
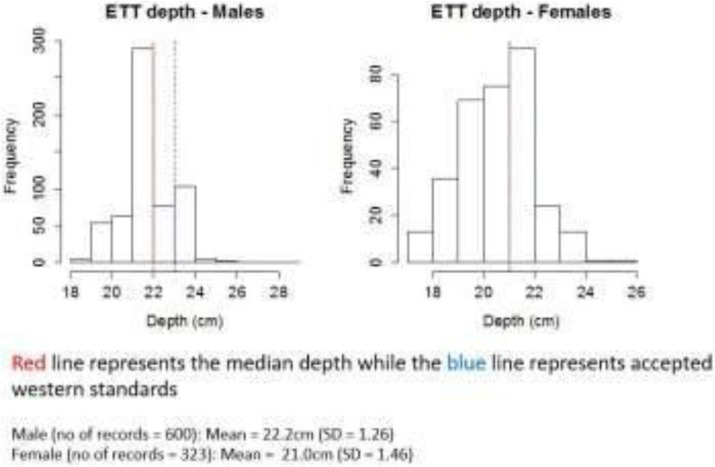
Median depth of ETT

**Fig. 75 (abstract A655).**
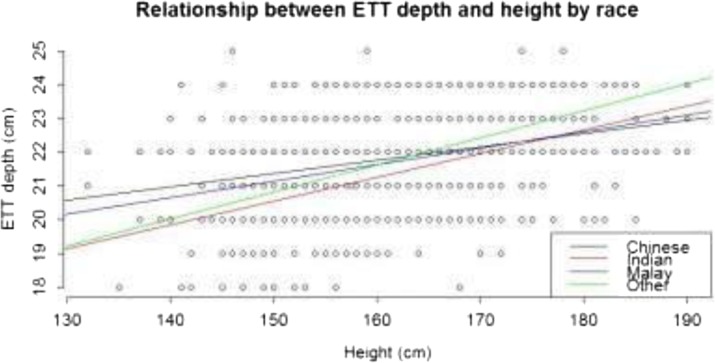
ETT depth relation with Ethnic group

**Fig. 76 (abstract A655).**
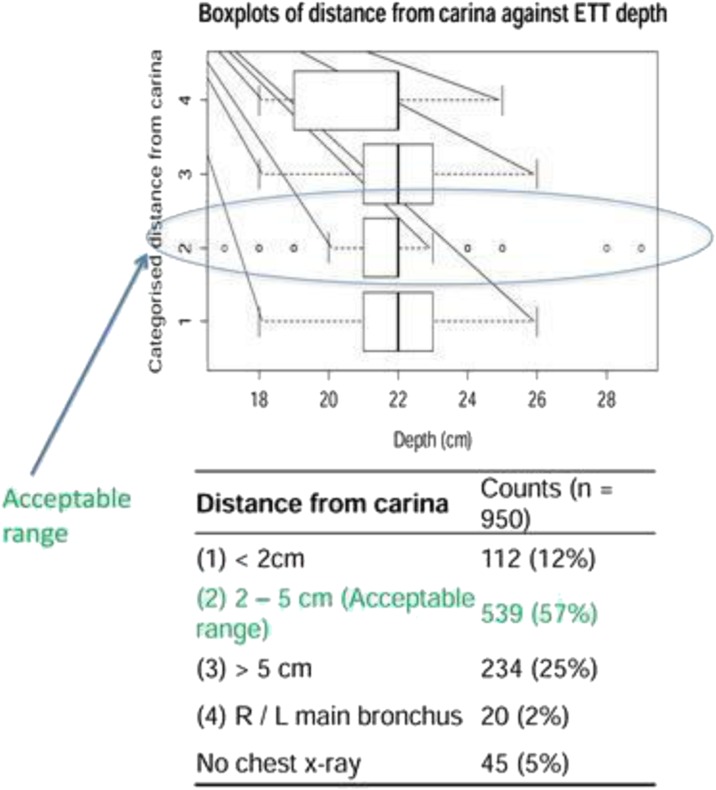
Boxplot carina distance to ETT depth

#### A656 Accurancy of extended evan's blue dye testing in predicting artificial airway dysphagia

##### M. Muñoz Garach, O. Moreno Romero, R. Ramirez Puerta, F. Acosta Diaz, A.M. Perez Bailon, A. Carranza Pinel, L. Peñas Maldonado

###### Complejo Hospitalario de Granada, ICU, Granada, Spain

####### **Correspondence:** M. Muñoz Garach - Complejo Hospitalario de Granada, ICU, Granada, Spain

**Introduction:** The patient with a tracheostomy tube may experience dysphagia. The physiologic factors that may contribute to the dysphagia include reduced laryngeal elevation, reduced pharyngeal sensation, reduced cough response, and disuse atrophy o laryngeal musculature. A tracheostomy tube with a cuff may be placed to prevent aspiration of secretions, food, and gastric contents. However, a patient may still aspirate some food with a fully inflated cuff due to severe dysphagia or a tracheostomy site placed too high. The identification and management of aspiration in the patient with a tracheostomy is important and often implies difficulties and challenging clinical decisions.

**Objetive:** Comparation of the Extended Evan's Blue Dye (EEBD) test in predicting artifical airway dysphagia versus Fiberoptic Endoscopic Evaluation of Swallowing (FEES) as the gold standard.

**Fig. 77 (abstract A656).**
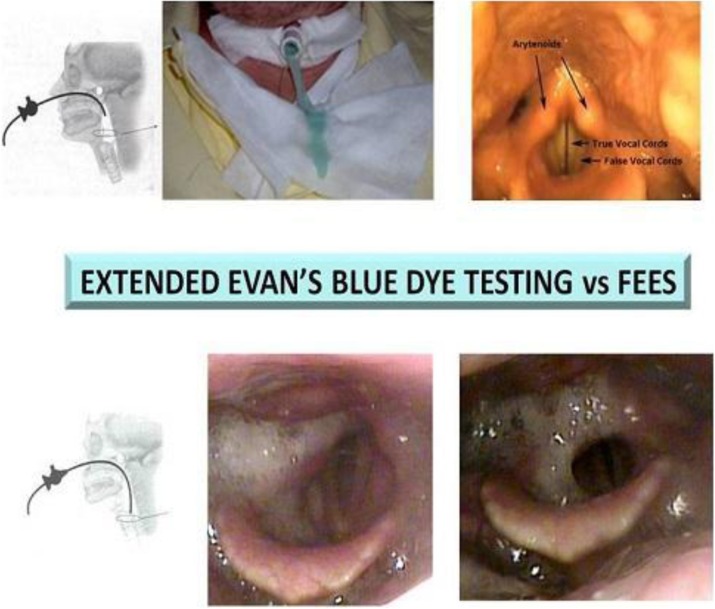
**ᅟ**

**Methods:** We prospectively registered 26 tracheotomized patients due to difficulty in weaning. First we used the EEBD test (2 ml blue Evans administered with a spoon in middle third of the tongue), then we observed wheather aspiration signs appear (early/late cough, symptoms of asphyxia, or more than 3 percentage points basal pulse oximetry desaturations), and the posible blue stained secretions suctioned through the tracheostomy tube. Simultaneously the FEES was performed, watching directely all the posibilities of penetration and or blue Evans aspiration (Fig. 77). The validity and performance enhancement were calculated, and the ROC curve was made with 95 % CI. The study was approved by the hospital ethics committee.

**Fig. 78 (abstract A656).**
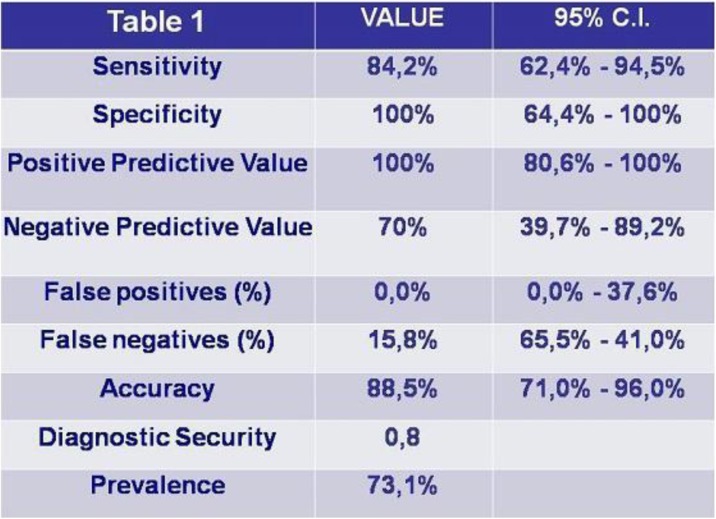
**ᅟ**

**Fig. 79 (abstract A656).**
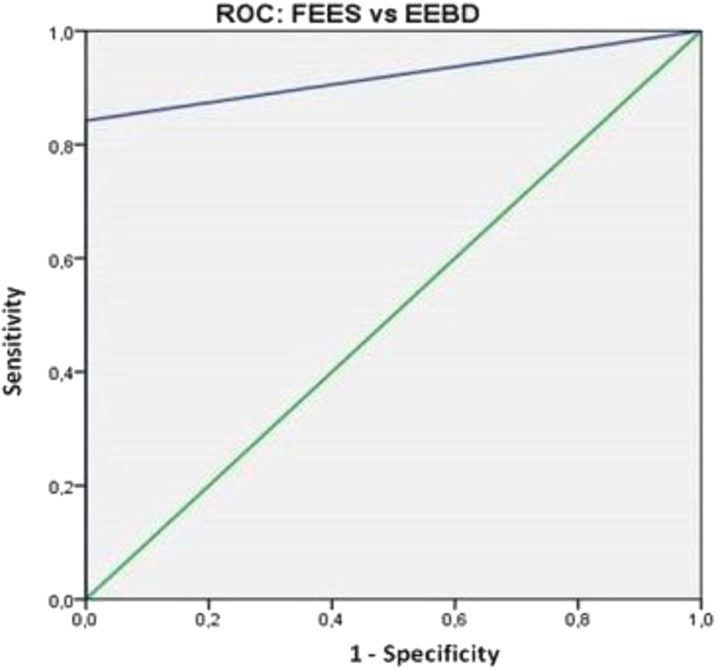
**ᅟ**

**Results:** 26 patients underwent FEES + EEBD: 7 had FEES without aspiration and a negative EEBD. 19 had FEES with aspiration, 16 were positive and 3 negative EEBD. The validity and performance enhancement indexes are described in Fig. 78, with an area under curve of 0.92 ± 0.54 (95%CI: 0.82-1.00) and p = 0.006 (Fig. 79).

**Conclusions**

1- The EEBD is a significant and simple diagnostic screening tool in dysphagia secondarily to artificial airway.

2- In our opinion if the EEBD is negative and there is high clinic suspicion, FEES should be considered to confirm, or not, dysphagia.

#### A657 Prospective analysis of indications and complications of endotracheal intubation in critically ill patients

##### M.S. Kalaiselvan^1^, R.L. Siva kumar^1^, M.K. Renuka^2^, A.S. Arun Kumar^1^

###### ^1^Sri Ramachandra University, Department of Critical Care Medicine, Chennai, India; ^2^Sri Ramachandra University, Department of Anaesthesiology, Chennai, India

####### **Correspondence:** M.S. Kalaiselvan - Sri Ramachandra University, Department of Critical Care Medicine, Chennai, India

**Introduction:** Endotracheal intubation(ETI)is a commonly performed procedure in the intensive care unit (ICU). Many life-threatening complications occur during endotracheal intubation and are associated with poor patient outcomes.

**Objectives:** To analyze the indications, complications and outcomes of patients who underwent Endotracheal intubation in ICU.

**Methods:** Prospective observational study, done in a university hospital ICU over a period of 6 months. Adult patients admitted to ICU requiring endotracheal intubation during ICU stay for more than 24 hours were included. Data collection included demographic profile, vital parameters, APACHE II and SOFA scores, oxygenation and ventilation status and indications for endotracheal intubation. Outcome data was collected on complications of Endotracheal intubation, mortality, ICU LOS, hospital LOS and ventilator free days.

**Results:** 171 patients fit into inclusion criteria. Mean age was 55.48(±15.86) years, majority of patients were males(n = 112[65.4 %]). The mean APACHE II and SOFA scores were 19.82(±4.547) and 5.64(±2.619)respectively. Respiratory failure (51.46 %) was the most common reason for intubation followed by neurological deterioration (21.63 %).

Majority of Endotracheal intubations were elective (81.28 %) and were performed by residents (43.85 %) with ICU experience of more than 3 years. 15.7 % of patients required muscle relaxant for facilitation of ETI. Most common complications were hypotension (39.18 %) and hypoxia (27.48 %) at the time of intubation.

5 (2.9 %) patients had cardiac arrest at the time of intubation, 18 (10.52 %) patients required vasopressor support post intubation. Mortality rate was 67.25 %(n = 115) with an ICU ALOS 8.27(±6.32) days and hospital LOS 12.54(±9.1) days. Average ventilator days were 7.27(±5.06)days. 7 % of patients developed VAP and 17 % of patients required tracheostomy. Risk factors for mortality included higher age, high APACHE and SOFA scores, hypoxia and hypotension at the time of intubation, requirement of vasopressor support at the time of intubation.

**Conclusions:** Endotracheal intubation was associated with high complications and poor outcomes.

**References**

1. Simpson GD, Ross MJ, McKeown DW, Ray DC. Tracheal intubation in the critically ill: a multi-centre national study of practice and complications. Br J Anaesth. 2012;108:792–9.

2. Jaber S, Amraoui J, Lefrant JY, et al. Clinical practice and risk factors for immediate complications of endotracheal intubation in the intensive care unit: a prospective, multiple-center study. Crit Care Med 2006; 34: 2355–61

**Grant acknowledgement**

None.

**Table 62 (abstract A657).** Indications for ETI

Indications for intubation	n-171(%)
Respiratory failure	88(51.4)
Neurological deterioration	37(21.6)
Hemodynamic instability	22(12.8)
Extubation failure	9(5.2)
Others	16(9.3)

**Fig. 80 (abstract A657).**
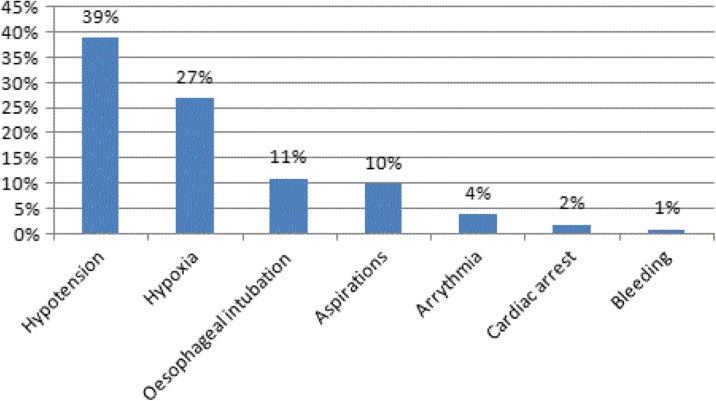
Complications during ETI

**Table 63 (abstract A657).** Comparison of survivors and non survivors

Parameters	Survivors(N=56)	Non survivors(N=115)	P value
Age (mean±SD)	50.14(±17.5)	58.08(±14.33)	0.002
Admission APACHE II (mean±SD)	16.48(±4.41)	21.45(±3.64)	0.000
Admission SOFA (mean±SD)	3.95(±1.28)	6.46(±1.25)	0.000
Requirement of vasopressor during ETI (%)	1.75	8.77	0.000
Hypoxia during ETI (%)	4.67	34.5	0.002
Hypotension during ETI (%)	4.09	23.39	0.001

#### A658 Airway management with Fastrach laryngeal mask versus Spritztube: a prospective randomised manikin-based study

##### S. De Rosa^1,2^, F. Ferrari^1,3^, S. Carboni Checcacci^3^, A. Rigobello^3^, M. Joannidis^4^, F. Politi^3^, A. Pellizzari^3^, R. Bonato^3^

###### ^1^International Renal Research Institute of Vicenza (IRRIV), Department of Nephrology, Dialysis and Transplantation, Vicenza, Italy; ^2^San Bortolo Hospital, Department of Anesthesiology and Intensive Care, San Bortolo Hospital, Vicenza, Italy; ^3^San Bortolo Hospital, Department of Anesthesiology and Intensive Care, San Bortolo Hospital, Vicenza, Italy; ^4^Medical University Innsbruck, Division of Intensive Care and Emergency Medicine, Department of Internal Medicine, Innsbruck, Austria

####### **Correspondence:** S. De Rosa - San Bortolo Hospital, Department of Anesthesiology and Intensive Care, San Bortolo Hospital, Vicenza, Italy

**Introduction:** Supraglottic airway devices are used with increasing frequency following routine anesthesia and for emergency airway management. Unfortunately, not all devices allow a possibility to perform a secondary endotracheal intubation (ETI) through the device. A new promising device, the Spritztube (ST), has been developed combining the ability to perform both supraglottic ventilation and orotracheal fibreoptic intubation using the same device. Particularly, it is able to perform a supraglottic ventilation during positioning as tracheal cannula during fibroscopy.

**Objectives:** To compare the ease, the time of insertion, and the success rates of use between the intubating laryngeal mask (Fastrach™) and the novel tracheal tube (Spritztube ®).

**Methods:** We evaluated the novel Spritztube and compared it to the Fastrach using a manikin-based study. Each partecipant received a verbal instruction and practical demonstration of technique of insertion for both devices on patient simulation manikins. Each participants used both devices in a randomised order. Time of placement (T1), time of inflation (T2), the elapsed procedural time (T3), ease of insertion, time of exchange maneouver for intubation (T4), success rates and number of attempts were recorded for each supraglottic device. After a maximum of 3 attempts airway-management was defined as a failure.

**Results:** A total of 47 participants were enrolled: 36 % without previous experience with Fibroscopy and 17 % without previous experiences with ETI. There was no difference in number of attempts for insertion between both devices(p = 0.68).Fastrach was applied 11 s faster than the Spritztube (median T3 Fastrach:13 s.,Spritztube:24 s., p = 0.000). The exchange manoevrue for ETI was 11 s faster for Fastrach than the Spritztube (median T3 Fastrach:13 s.,Spritztube:24 s., p = 0.000). Number of attempts for exchange to ETI were significantly more for Fastrach (p = 0.000). Participants judged Spritztube as easier insertion than Fastrach (p = 0.000). Spritztube had a significant higher success rate than Fastrach (p = 0.000). However the success rate did not depend on previous experience in ETI or in fibroscopy.

**Conclusions:** The Spritztube performed similarly to the Fastrach in insertion and intubation times in a manikin setting.

**References**

1. Sethi P, Samra T, Gupta N. Comparison of supraglottic devices i-gel(®) and LMAFastrach(®) as conduit for endotracheal intubation. Indian J Anaesth. 2014 Nov-Dec.58(6):790.

2. Halwagi AE, Massicotte N, Lallo A, Gauthier A, Boudreault D, Ruel M, Girard F. Tracheal intubation through the I-gel™ supraglottic airway versus the LMA Fastrach™: a randomized controlled trial. Anesth Analg. 2012 Jan;114(1):152–6.

**Grant acknowledgement**

None

#### A659 Dysphagia following prolonged mechanical ventilation and tracheostomy in critical ill patients. Results of edisval study after first year

##### A. Fernandez-Carmona^1^, I. Macias-Guarasa^2^, R. Gutierrez-Rodriguez^2^, P. Martinez-Lopez^3^, M.A. Diaz-Castellanos^4^, EDISVAL Group

###### ^1^Hospital Virgen de las Nieves, Intensive Care, Granada, Spain; ^2^Hospital Regional, Intensive Care, Malaga, Spain; ^3^Hospital Virgen de la Victoria, Malaga, Spain, ^4^Hospital Santa Ana, Motril, Spain

####### **Correspondence:** A. Fernandez-Carmona - Hospital Virgen de las Nieves, Intensive Care, Granada, Spain

**Introduction:** Available data shows that dysphagia and swallowing disorder rate secondary to artificial airway and prolonged mechanical ventilation in critical tracheostomized patients is high (50-83 %), nevertheless it real incidence is not yet well established. Dysphagia is directly related to bronchial aspirations and respiratory infections. The rate of respiratory infections on tracheostomized patients is also very high (Some series next 100 %). The re-establishment of airway using speaking valves allow the rehabilitation and post-recovery of those disorders, as well as deglutition and phonatory system rehabilitation.

The aim of EDISVAL Study is to determine the usefulness of speaking valve in preventing respiratory nosocomial infections in critical tracheostomized patients diagnosed of dysphagia secondary to artificial airway, for what it was done a screening test of dysphagia to the critical tracheostomized patients.

**Objectives:** To describe the incidence of swallowing disorder in critical patients who require tracheostomy secondary to artificial airway and prolonged mechanical ventilation.

**Methods:** From September 2014 until December 2015, in all patients over 18 years, during mechanical ventilation weaning-decannulation phase, without neurological or surgical disease which could contribute to the appearance of dysphagia, there was realized the Modified Evans Blue Dye Test (MBDT) as dysphagia screening test. This study was carried out of simultaneous form in 7 intensive care units including first, second and third level centers.

**Results:** Mean age average of the patients was 67,6 years, the initial APACHE´S average was 23,6 points. Mean time of mechanical ventilation was 27,6 days. During the study period we studied 37 patients by MEBDT, 34 patients were diagnosed of dysphagia, and included to the EDISVAL study; we did not carry out other specifics test of dysphagia to discriminate a possible MEBDT false negative.

In the patients with negative MEBDT, not diagnosed of dysphagia, respiratory infections were not registered.

In the patients diagnosed of dysfagia included at EDISVAL study, in spite of strict measures for it prevention, including absolute oral diet, 15 respiratory infections were registered (40.54 %), 5 catalogued as tracheobronchitis and 10 as pneumonia. 3 of the patients who suffered respiratory serious infections died during hospital stay.

**Conclusions:** The dysphagia secondary to artificial airway incidence in our series was 91,9 % according to the Modified Evans Blue Dye Test. Respiratory infections were a frequent complication in the patients diagnosed of dysphagia, with a probable repercussion in the mortality of the patients.

**References**

1. Romero CM. Swallowing dysfunction in nonneurologic critically ill patients who require percutaneous dilatational techeostomy. Chest 2010;137

2. Fernández A. Exploration and approach to artificial airway dysphagia. Med Intensiva 2012;36

#### A660 Use of speaking valve on preventing respiratory infections, in critical traqueostomized patients diagnosed of dysphagia secondary to artificial airway. Edisval study first 12 months

##### A. Fernandez-Carmona^1^, M. Arias-Diaz^2^, E. Aguilar-Alonso^3^, I. Macias-Guarasa^4^, P. Martinez-Lopez^5^, M.A. Diaz-Castellanos^2^, EDISVAL Group

###### ^1^Hospital Virgen de las Nieves, Intensive Care, Granada, Spain; ^2^Hospital Santa Ana, Motril, Spain; ^3^Hospital Infanta Margarita, Intensive Care, Cabra, Spain; ^4^Hospital Regional, Intensive Care, Malaga, Spain, ^5^Hospital Virgen de la Victoria, Malaga, Spain

####### **Correspondence:** A. Fernandez-Carmona - Hospital Virgen de las Nieves, Intensive Care, Granada, Spain

**Introduction:** Available data shows that dysphagia rate, secondary to artificial airway and prolonged mechanical ventilation, in critical tracheostomized patients is high (50-83 %). Dysphagia is directly related to bronchial aspirations and respiratory infections. The rate of respiratory infections on tracheostomized patients is also very high (Some series next 100 %). The re-establishment of airway using speaking valves allow the rehabilitation and post-recovery of those disorders, as well as deglutition and phonatory system rehabilitation.

**Objectives:** Determine the usefulness of speaking valve in preventing respiratory nosocomial infections in critical tracheostomized patients diagnosed of dysphagia secondary to artificial ariway.

**Methods:** Randomized multicenter controlled clinical trial. From December 2014 until February 2016, the use of speaking valve was randomized during mechanical ventilation weaning-decannulation phase in critical patients over 18 years, tracheostomized at ICU, diagnosed of dysphagia secondary to artificial airway. Neurological patients were excluded. In all cases it was contraindicated oral diet intake. This study was carried out of simultaneous form in 5 units of intensive care including centers of the first, second and third level.

**Results:** 32 patients were included, 72,7 % were men, mean age of 63,21 ± 15,13 years, mean APACHE-II 22,45 ± 8,75 points. Mean time of mechanical ventilation was 28,82 ± 14,23 days. The most common reason for ICU admission was respiratory 39.1 % of patients, followed by surgery and shock.

After ramdomisation 19 patients were treated with speaking valve (SVgroup), and 13 by identical protocol without speaking valve (decannulation protocol, DPgroup). The incident of infections in the SVgroup was 26,3 % (5/19), 2 pneumonia and 3 cases of tracheobronquitis; and 61,5 % (8/13) in the DPgroup, 5 pneumonía and 3 tracheobronquitis. Some cases of this group were more than one respiratory infection process. The mortality was 0 in the SVgroup and 3 in DPgroup.

Both groups had a mean mechanical ventilation time and reasos of ICU admission similar, APACHE-II was higher in PDgroup (19.00 vs 26.6 points) and age (61.71 vs 66.71 years). SVgroup had a higher ICU stay than the DPgroup (51.00 vs 39.10 days) and greater hospital stay (69.44 vs 60.75 days). Patients who died were not those with higher APACHEII or older in DPgroup.

**Conclusions:** The incidence of infectious complications and mortality was lower in SVgroup. In the following months will continue this research in order to know if these differences are attributable to SV use

#### A661 Endotracheal tube cuff pressures on admission to intensive care unit

##### R.N. Nikandish

###### Shiraz University Of Medical Sciences, Shiraz, Islamic Republic of Iran

**Introduction:** High endotracheal tube(ETT) cuff pressure could damage tracheal mucosa and result in significant airway complications. We hypothesized that high cuff pressure on intensive care unit (ICU) admission is very common. So we designed this study as a quality measurement audit to gauge ETT cuff pressure on ICU arrival.

**Methods:** In this prospective observational study we included all intubated who were admitted from different clinical area to ICU. We measured ETT cuff pressure on ICU admission using an aneroid cuff pressure gauge during a 6 month period.

**Results:** We measured ETT cuff pressure in 159 patients on their arrival to ICU. The Mean cuff pressure on ICU admission was 63.94 (SD 31.52) cm H2O. There was no statistically significant difference in ETT cuff pressure between the patients admitted from different clinical areas. Duration between tracheal intubation and patient handover to ICU was 9.5 (SD 8.0) hours.

**Conclusion** High ETT cuff pressure is very common on ICU admission and this can set the stage for airway complications. Using cuff pressure gauge is a simple and effective way to measure and readjust ETT cuff pressure immediately after intubation.

**References**

1. Andrews MJ, Pearson FG. Incidence and pathogenesis of tracheal injury following cuffed tube tracheostomy with assisted ventilation: Analysis of a two-year prospective study. Ann Surg 1971;173(2):249–263

2. Seegobin RD, van Hasselt GL. Endotracheal cuff pressure and tracheal mucosal blood flow: Endoscopic study of effects of four large volume cuffs. BMJ 1984;288(6422):965–968.

3. Quemby D, Bennett M, Robbins P.An audit of endotracheal cuff pressures on admission to cardiac surgical intensive care. Electronic poster. 0621.ESICM, Berlin 2011

4. Chapman J, Pallin D, Ferrara L, et al. Endotracheal tube cuff pressures in patients intubated before transport. Am J Emerg Med 2009;27(8):980–982.

5. Galinski M, Treoux V, Garrigue B, Lapostolle F, Borron SW, Adnet F. Intracuff pressures of endotracheal tubes in the management of airway emergencies: The need for pressure monitoring. Ann Emerg Med 2006;47(6):545–547

6. Bernon JK, McGuire CI, Carrara H, Lubbe DE Endotracheal tube cuff pressures - the worrying reality: a comparative audit of intra-operative versus emergency intubations. S Afr Med J. 2013 5;103(9):641–3.

#### A662 Efficacy of conduction anaesthesia of nervus mandibularis and videolaryngoscope “tepro” in patients with inflammatory lockjaw

##### V. Artemenko^1^, A. Budnyuk^2^

###### ^1^Odessa National Medical University, ICU, Odessa, Ukraine; ^2^Odessa National Medical University, Odessa, Ukraine

####### **Correspondence:** V. Artemenko - Odessa National Medical University, ICU, Odessa, Ukraine

**Introduction:** In most cases, endotracheal intubation in patients with the neck phlegmon may not be feasible due to inflammatory lockjaw and mouth opening limitation[1,3]. Conductive anaesthesia of nervus mandibularis can improve condition for intubations in patients with inflammatory lockjaw [2].

The goal of work: to study the efficacy of conductive anaesthesia nervus mandibularis with videolaryngoscopy “Tepro” and to provide optimal conditions for intubation in patients with inflammatory lockjaw.

**Materials and methods:** We analysed medical cases of 47 pts. with the surgical treatment of the neck phlegmon and inflammatory lockjaw. The control group (n = 23) tracheal intubation was proved by conventional laryngoscope *Macintosh*. In the main group (n = 24) patients have limitation of mouth opening. In those patients conduction anesthesia of nervus mandibularis was performed and trachea intubation by videolaryngoscope “Tepro”; (Table 64).

**Results:** In control group, rate of difficult laryngoscopy and failed tracheal intubation was 91.3 % due to limitation of mouth opening by lockjaw.

In main group we performed conduction anesthesia of nervus mandibulus and videolaryngoscopy, that significantly reduced the rate of difficult and failed intubation to 3.3 % in comparison with patients in the control group (χ2 = 32.36; p = 0.0000).

Conclusions. Conduction anaesthesia of nervus mandibulus with videolaryngoscopy “Tepro” increased the amplitude of mouth opening, reduced the rate of difficult laryngoscopy and failed intubation, improved the larynx visualization, therefore provided the best conditions for intubation in patients with inflammatory lockjaw.

**References**

1. Can't intubate, can't ventilate! A survey of khowledge and skills in a large teaching hospital / L. Green // Eur J Anesthesiol. - 2009. - Vol. 26 - P. 480–483.2. Guideline for maxillofacial and dentistry Surgery/ Timofeev A.A.-Kyiv, 2002.- P. 112.3. Algorithm of difficult intubations trachea/ Chuev P.N. - Kiev, 2007. - p 52.

**Table 64 (abstract A662).** Comparative characteristics of different methods o

The groups of pts.	Trachea intubation, tot (%)			
	Difficult	Easy	χ2	P
Control (n=23)	21(91,3)	2 (8,3)		
Main (n=24)	2 (8,7)	22(91,7)	32,36	0,0000*
Total (n=47)	23(100)	24 (100)		

#### A663 Preliminary evaluation of a novel strategy to aspirate subglottic secretions following intubation with standard endotracheal tubes

##### G. Li Bassi^1^, T. Senussi^2^, F. Idone^2^, E. Aguilera Xiol^2^, C. Travierso^3^, C. Chiurazzi^3^, A. Motos^2^, R. Amaro^2^, Y. Hua^2^, L. Fernández-Barat^2^, O.T. Ranzani^2^, Q. Bobi^2^, M. Rigol^2^, A. Torres^2^

###### ^1^Hospital Clinic, Pulmonary and Critical Care Medicine, Barcelona, Spain; ^2^Hospital Clinic, Barcelona, Spain; ^3^University of Milan, Fisiopatologia Medico-Chirurgica e dei Trapianti, Milano, Italy

####### **Correspondence:** G. Li Bassi - Hospital Clinic, Pulmonary and Critical Care Medicine, Barcelona, Spain

**Introduction:** Endotracheal tubes (ETTs) that allow subglottic secretions aspiration (SSA) reduce ventilator-associated pneumonia (1). An innovative device has been recently developed to insert an aspiration catheter into the subglottic region, post intubation with standard ETTs.

**Objectives:** In animals on mechanical ventilation up to 76 hours, we evaluated safety and efficacy of the novel SSA strategy, in comparison with standard ETTs comprising SSA.

**Fig. 81 (abstract A663).**
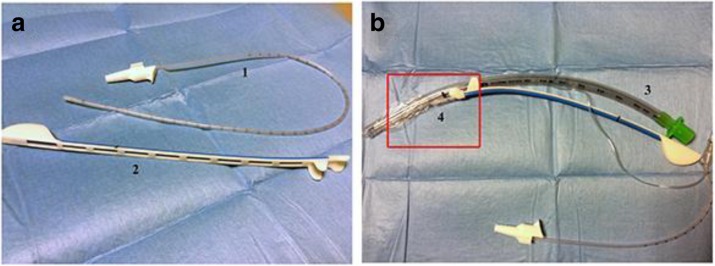
Novel device to place SSA catheter

**Methods:** Thirteen pigs (31.7 ± 2.6 Kg) on mechanical ventilation (MV) for 76 hours. Pigs were randomized to be intubated with a standard SSA ETT (Control). Upon aspiration, the patency of the suction lumen was tested; then, secretions were aspirated and the volume quantified. Whereas, in the treatment group, a novel delivery device was employed to advance a 12-Fr suction catheter into the subglottic space and perform SSA as reported above (Fig. 81).

A: 1, Subglottic secretions aspiration catheter; 2, Delivery device to place the aspiration catheter into the subglottic region. B: 3, ETT. Upon insertion, the delivery device was placed along the concave surface of the endotracheal tube and advanced up to the larynx. 4, Then, the catheter was inserted into the internal channel of the delivery device and advanced up to the subglottic region.

Upon autopsy, lesions of the 1) epiglottis, 2) corniculate cartilages, 3) true vocal cords, and 5) subglottic region were grossly scored: 0: no injury; 1: mucosal erythema and/or edema; 2: mucosal erosion; 3: exposed cartilage.

**Results:** Six animals (4 controls and 2 treatment) were kept in the lateral-Trendelenburg position and aspiration was performed every 4 hours. Whereas, 7 animals (2 controls and 5 treatments) were kept in semi-recumbent position and SSA was performed every 8 hours. Among animals in lateral-Trendelenburg position, SSA was performed 18.2 ± 0.9 and 18.5 ± 0.7 times in the control and treatment groups, respectively (p = 0.972). Overall, the mean volume of aspiration was 0.05 ± 0.07 (range 0–0.25 mL) and 0.03 ± 0.07 mL (0–0.25 mL) of secretions/hour in the control and treatment group, respectively (p = 1.000). The median [IR] level of injury was 1[0] and 1[0] in the control and treatment group, respectively (p = 0.258). Whereas, among animals in semi-recumbent position, SSA was performed 5.5 ± 0.7 and 7.6 ± 2.7 times in the control and treatment groups, respectively (p = 0.378). Overall, the median [IR] volume of aspiration was 0.14 ± 0.12 (range 0–0.41 mL) and 0.17 ± 0.30 mL (0–1.33 mL) of secretions/hour in the control and treatment group, respectively (p = 0.324). The median [IR] level of injury was 1[0] and 1[0] in the control and treatment group, respectively (p = 0.734).

**Conclusions:** In preliminary *in-vivo* evaluations, the novel SSA strategy is safe and not inferior to standard ETTs comprising SSA.

**References**

1. Muscedere J et al. *Crit Care Med*. 39:1985–91

**Grant acknowledgement**

Ciel Medical, the manufacturer of the novel delivery device.

#### A664 Comparison of the clinical performance of air-Q and i-gel during positive pressure ventilation

##### A. Youn, J. Gyung Hwang

###### Chungnam National University Hospital, Anesthesiology and Pain Medicine, Daejon, Republic of Korea

####### **Correspondence:** J. Gyung Hwang - Chungnam National University Hospital, Anesthesiology and Pain Medicine, Daejon, Republic of Korea

**Introduction:** Successful airway management is crucial in emergency care especially when endotracheal intubation is difficult. The air-Q is a new supraglottic airway device used as a conduit for endotracheal intubation. This study compared the performance of air-Q with i-gel regarding airway seal pressures and ease of intubation for better function.

**Objectives:** We hypothesized that the air-Q would have better airway sealing than the i-gel due to its larger cuff design and flexibility and compared it between patients undergoing positive pressure ventilation for general anesthesia.

**Methods:** 50 patients classified as ASA 1 or 2 undergoing general anesthesia were randomly allocated into two groups, group A (Air-Q) or group I (i-gel). Anesthesia was induced with propofol 2 mg/kg and rocuronium 0.5/kg. We compared the ease of intubation, vital signs, airway sealing pressures, and adverse events upon and during insertion. Acceptable peak inspiratory pressures were limited to 40 cmH2O, in which cases exceeded, endotracheal intubation was performed.

**Results:** Airway leak pressures were lower in group A than group I. Hemodynamic and respiratory conditions were statistically insignificant between the two groups. Adverse events occurred in 1 patient from group A and 4 patients from group I in the post-anesthetic care unit.

**Conclusions:** The Air-Q provides an adequate airway seal and can be efficiently used in patients undergoing positive pressure ventilation.

**References**

1. Bamgbade OA, Macnab WR, Khalaf WM. Evaluation of the i-gel airway in 300 patients. European Journal of Anaesthesiology 2008; 25: 865–6.

2. Bakker EJ, Valkenburg M, Galvin EM. Pilot study of the air-Q intubating laryngeal airway in clinical use. Anaesthesia and Intensive Care 2010; 38: 346–48.

#### A665 Utility of direct laryngoscopy after negative cuff-leak test

##### M. Muñoz Garach, O. Moreno Romero, M.E. Yuste Ossorio, F. Acosta Diaz, A.M. Perez Bailon, A. Carranza Pinel, L. Peñas Maldonado

###### Complejo Hospitalario de Granada, ICU, Granada, Spain

####### **Correspondence:** M. Muñoz Garach - Complejo Hospitalario de Granada, ICU, Granada, Spain

**Introduction:** Endotracheal intubation is a common procedure in the treatment of respiratory failure in the Intesive Care Unit (ICU). Sometimes generates local complications that might difficult or even make it imposible to remove the artificial airway (AA). If this complications are not detected before the AA removal, stridor, laryngeal dyspnea or spasms might apear requiring emergency reintubation/recannulation in rather difficult circumstances with severe increase in morbimortality. Therefore is essential to follow a clinical guideline in the removal of the AA once overcome weaning and espontaneous breathing test.

**Objetive:** To describe the incidence of treatable causes for negative cuff-leak test in our critical care unit.

**Methods:** We prospectively registered all patients admitted in our ICU (18 beds) who underwent artificial airway procedure (endotracheal tube or tracheotomy) during 24 months (from January 2014 to December 2015). In every patient who overcome the weaning guideline process (including spontaneous breathing test), we implemented also an aritificial airway removal guideline, containing the cuff-leak test, in Volume Controlled Ventilation (CMV) and with the cuff deflated. If leak rate was greater than 20 % of tidal volumen, “positive test”, we could safely remove the artificial airway. If leak rate was less than 20 %, “negative test”, an upper airway revision by direct laryngoscopy was performed to evaluate treatable causes.

**Fig. 82 (abstract A665).**
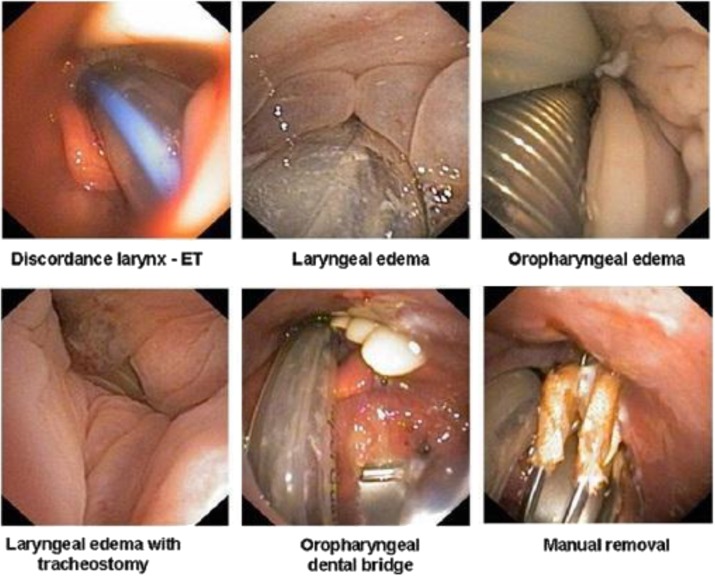
ᅟ

**Results:** We registered 407 patients in ICU with artificial airway procedures (endotracheal tuve, tracheotomy) during the study period. Mean age was 62 years (48–76 CI 95 %), 67,8 % were male and the mean APACHE was 23 (33–13 CI 95 %). Before extubation/decanulation, cuff-leak test was performed. 374 (91.9 %) had “positive test” and 33 (8.1 %) “negative test”. In these negative test a direct laryngoscopy was performed and the following treatable causes were found: 23 laryngeal edema, 2 dental bridge impacted in the pharynx, 1 supraostial laryngeal edema plus granuloma, 1 edema plus decubitus, 1 laryngeal granuloma, 1 distal tube obstruction, 1 laryngeal granuloma in intercricotiroidea tracheotomy, 1 with a bloodclot in the larynx and finally 1 with a tumor in vocal cordv (Fig. 82).

**Conclusions**

- The inclusion of an upper airway direct laryngoscopy exam after negative cuff-leak test, ensures specific diagnosis and manage of the treatable causes.
- In all our patients, the artificial airway was safely removed after treating the cause, avoiding the increase of morbimortality due to reintubation/recanulation.

#### A666 How do we deal with the airway: assessment in the emergency department (ED) and intensive care unit (ICU)

##### C. Teixeira^1,2^, H. Figueira^1^, R. Oliveira^1^, A. Mota^1^, I. Aragão^1^

###### ^1^Centro Hospitalar do Porto, Hospital de Santo António, UCIP-Departamento de Anestesiologia, Cuidados Intensivos e Emergência, Porto, Portugal; ^2^Universidade do Porto, Instituto de Ciências Biomédicas Dr. Abel Salazar, Porto, Portugal

####### **Correspondence:** C. Teixeira - Universidade do Porto, Instituto de Ciências Biomédicas Dr. Abel Salazar, Porto, Portugal

**Introduction:** Airway management is a commonly performed procedure in the ED and in the ICU. The management of the airway is crucial in that setting, being tracheal intubation (TI) the gold standard. Several studies identify higher rates of complications in these settings.

**Objectives:** Assess the practice of airway approach in the ED and ICU in a university hospital, and identify risk factors for difficult intubation and its main complications.

**Methods:** Prospective observational study. Data collection regarding the operator, the patient, the technique and the outcome were completed at the time of intubation. Attempted laryngeal visualization to facilitate TI was used as the definition of an attempted intubation (ATI).

**Results:** 182 records filled, 257 ATI during this period. The leading cause was acute respiratory failure (26 %). 122 patients were intubated at the first attempt and 60 needed 2 or more attempts (min1, max4). The success rate of TI at the first attempt was 67 %. Intubation success rate at first ATI was similar during day or night (p = 0.53). Regarding the operator: 18 % of the missed TI at first ATI were performed by anesthetists, while that number rose to 75 % in non-anesthetists (p = 0.013). 88 % of missed TI at first ATI were performed by residents, while senior doctors just missed 12 % (p < 0.033). Considering the operator reported own experience: 65 % with few airway experience didn´t intubate at first attempt, doctors with good and excellent airway expertise just missed first ATI in 20 % and 3 % of the times respectively (p < 0.001). Predictive signs of difficult airway were identified in 95 patients. Being obese and neck deformity the most reported ones (Respectively 25 % and 21 %). 70 % of patients who have not been intubated at the first ATI had predictive signs of difficult airway (p < 0.001). Complications were reported in 30 % of the ATI, namely: failed intubation 18 %, esophageal intubation 9 %, aspiration of gastric contents 2 %, and cardiac arrest 1 %. Main contributory factors reported for failure to IT at first ATI: operator's inexperience 13 % and patient-related 11 % (both attained statistical significance p < 0.001). Lack of equipment and lack of strategy delineation were only reported in 2 % each. No surgical or rescue airway supraglottic devices were required.

**Conclusions:** Failed TI and its complication are largely due to the operator's inexperience. As our hospital is a teaching one, TI in our ED and ICU is performed mostly at first by trainee doctors. Patient-related factors also play a role. Risk factors assessment for difficult intubation should be a routine in ICU patients Training in recognition and planning the approach of the difficult airway and expertise immediately available are crucial in the airway management in these settings.

**References**

1. De Jong et al. Critical Care 2014, 18:209 http://ccforum.com/content

#### A667 Percutaneous dilatational tracheostomy in ankylosing spondylitis (Bechterew`s disease) is feasible and not associated with higher complication rates

##### O. Kamp^1^, O. Cruciger^1^, M. Aach^2^, C. Kaczmarek^1^, C. Waydhas^1^, T.A. Schildhauer^1^, U. Hamsen^1^

###### ^1^Berufsgenossenschaftliches Universitätsklinikum Bergmannsheil, Surgical Clinic and Polyclinic, Bochum, Germany; ^2^Berufsgenossenschaftliches Universitätsklinikum Bergmannsheil, Surgical Clinic - Department of Spinal Cord Injuries, Bochum, Germany

####### **Correspondence:** O. Kamp - Berufsgenossenschaftliches Universitätsklinikum Bergmannsheil, Surgical Clinic and Polyclinic, Bochum, Germany

**Introduction:** Spondylitis ankylosans (Bechterew's disease) is a common disease with an incidence of about 0.5 % in Europe. Percutaneous dilatational tracheostomy is a common procedure in Intensive Care Units. . Intubation and tracheostomy can be complicated by deformities of the cervical spine and the temporomandibular joints and cervical spine. A combination of cervical kyphosis and osteoporosis also leads to complexities in patient positioning.

**Objectives:** These fact could suggest that patients suffering spondylitis ankylosans are not suitable for percutaneous dilatational tracheostomy but no study or case report ever reported about feasibility of this procedure in Bechterew`s disease.

**Methods:** Retrospective analysis at one Level 1 trauma center of patient records from 2002–2016, identifying all patients with Spondylitis ankylosans (SA) and percutaneous dilatational tracheostomy (PDT).

**Results:** 31 patients underwent PDT. All PDTs were performed using the Ciaglia single step dilation technique. No cardiopulmonary or surgical complication occurred during the procedure, especially no airway loss, no desaturation, no hemodynamic abnormalty or bleeding. One patient received airway switch from a small nasal tube to a laryngeal mask before starting the PDT procedure.

**Conclusions:** A percutaneous dilatational tracheostomy in a patient with Bechterew' s disease is a safe procedure in an experienced centre.

**References**

Dean L.E., Jones G.T., MacDonald A.G., Downham C., Sturrock R.D., Macfarlane G.J. Global prevalence of ankylosing spondylitis. Rheumatology (Oxford) 2014;53(4):650–657.

Cheung NH, Napolitano LM. (2014) Tracheostomy: epidemiology, indications, timing, technique, and outcomes. Respir Care;59(6):895–915; discussion 916–9

Romero CM, Cornejo RA; Ruiz MH; Galvez LR, Llanos OP, Tobar EA et al.(2009) Fiberoptic bronchoscopy-assisted percutaneous tracheostomy is safe in obese critically ill patients: a prospective and comparative study J crit care; 24(4) 494–500

Romero J, Vari A, Gambarrutta C, Oliviero A. (2009) Tracheostomy timing in traumatic spinal cord injury. Eur Spine J 18(10):1452–1457.

Binder H, Lang N, Tiefenboeck TM, Bukaty A, Hajdu S, Sarahrudi K. (2015) Tracheostomy following anterior cervical spine fusion in trauma patients. Int Orthop. 2015 Jul 21.

Romero-Ganuza J1, Gambarrutta C, Merlo-Gonzalez VE, Marin-Ruiz MÁ, Diez De La Lastra-Buigues E, Oliviero A. (2011) Complications of tracheostomy after anterior cervical spine fixation surgery. Am J Otolaryngol.;32(5):408–11.

### Experimental studies in acute lung injury

#### A668 Heparin effect in alveolar macrophages in acute lung injury model

##### M. Camprubí-Rimblas^1^, L. Chimenti^1^, R. Guillamat-Prats^2^, T. Lebouvier^3^, J. Bringué^1^, J. Tijero^1^, M.N. Gómez^1^, L. Blanch^1,2^, A. Artigas^2,4^

###### ^1^Fundació Parc Taulí, Sabadell, Spain; ^2^CIBERES, Sabadell, Spain; ^3^Surgical Intensive Care Unit Ponchaillou University Hospital, Rennes, France; ^4^Critical Care Center - Corporació Sanitària i Universitària Parc Taulí, Sabadell, Spain

####### **Correspondence:** J. Bringué - Fundació Parc Taulí, Sabadell, Spain

**Introduction:** Acute Lung Injury (ALI) and Acute Respiratory Distress Syndrome (ARDS) are characterized by a promptly release of proinflammatory mediators that downregulate natural anticoagulant mechanisms, initiate the coagulation system, impair fibrinolysis and produce the rupture of the endothelial and epithelial monolayer (1). Proinflammatory activated Alveolar Macrophages (AM) initiate and regulate ALI/ARDS inflammation and contribute to the propagation of the coagulant response. Currently there is no effective treatment for this disease. Previous studies have presented the beneficial effect of anticoagulants for their anticoagulation and anti-inflammatory action (2–3).

**Objectives:** Evaluate the effect of local heparin administration in AM in ALI model.

**Methods:** Sprague–Dawley rats (~300 g) underwent intratracheal administration of Lipopolysaccharide (LPS 10 μg/g body weight) or Saline (0.9 %) in control animals. Saline or heparin (1000 IU/kg body weight) was nebulized through Aeroneb system (Philips Healthcare) at constant oxygen flow (2 L/min) before and after LPS administration. Animals were sacrificed 24 h after injury and AM from all groups were isolated with a bronchoalveolar lavage. The pro and anti-inflammatory pathways involved in AM activity were evaluated by qRT-PCR. Neutrophil and monocytic chemoattractant activity of AM was evaluated by CXCL1 and CCL2, respectively. Data expressed as media ± SEM, relative to GAPDH and fold over saline group (n = 8 for all the study groups). Statistics: One-Way-ANOVA and Newman Keuls post-hoc test (Statistical significance: p ≤ 0.05).

**Fig. 83 (abstract A668).**
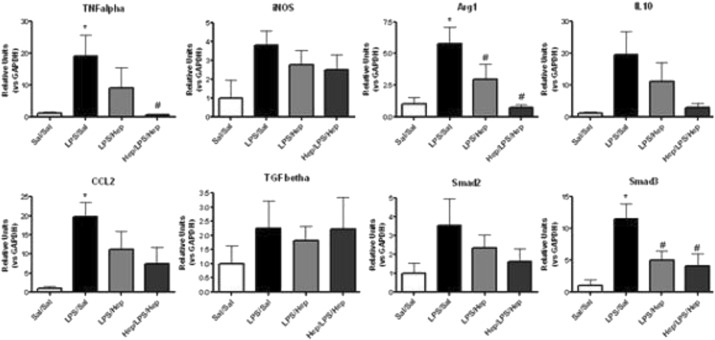
Gene expression of pro and anti-inflammatory markers and chemoattractant action of AM. (n=8); *p≤0.05 vs Sal/Sal; # p≤0.05 vs LPS/Sal

**Results:** In ALI model there was a significant increase in TNFα, Arginase-1, Smad/TGFβ effectors and CCL2. Heparin reduced significantly TNFα, Arginase-1 and Smad 3. Further, changes were observed in iNOS, IL10, and Smad 2. Although no changes were detected on neutrophil chemoattractant activity of AM, monocytic chemotattractant activity was diminished by heparin (Fig. 83).

**Conclusions:** Altogether indicate that AM have a major role in the ALI development and resolution. During ALI, AM present more activity and the treatment with heparin is able to attenuate this response diminishing the lung injury.

**References**

1. Ware LB *et al.* The acute respiratory distress syndrome. N Engl J Med 2000; 342:1334–1349

2. Mu E *et al.* Heparin attenuates lipopolysaccharide-induced acute lung injry by inhitibiting nitric oxide synthase and TGF-β/Smad signaling pathway. Thromb Res. 2012;129(4):479–85

3. Li Y *et al.* The effect of heparin administration in animal models of sepsis: A prospective study in *Escherichia coli*-challenged mice and a systematic review and metaregression analysis of published studies. Crit Care Med 2011; 39(5): 1104–1112

**Grant acknowledgement**

This Project has been supported by the FIS-PI12/02548 project integrated to the *Plan Nacional de I + D + I* and jointly financed by *ISCIII-Subdirección General de Evaluación y el Fondo Europeo FEDER*, the CIBERES and the Fundació Parc Taulí.

#### A669 Action of costal and crural diaphragm during hypercapnic stimulation in dogs

##### G. Tagliabue, M. Ji, J.V. Suneby Jagers, P.A. Easton

###### University of Calgary, Critical Care Medicine, Calgary, Canada

####### **Correspondence:** G. Tagliabue - University of Calgary, Critical Care Medicine, Calgary, Canada

**Introduction:** Classically, with increasing CO_2_ there is a linear increase in minute ventilation and diaphragm electromyogram (EMG) (1). Several lines of evidence suggest that the Costal and Crural portions of the diaphragm are two distinct muscles (2,3). Although both muscles would be expected to increase in parallel with minute ventilation during CO_2_ stimulation, there is no a priori reason that Costal and Crural would have identical neuro-mechanical recruitment.

**Objectives:** To determine if the two portions of the diaphragm, Costal (COS) and Crural CRU) diaphragm, are recruited identically during CO_2_ stimulated ventilation.

**Methods:** Sonomicrometry transducers and EMG wire electrodes were implanted in the left COS and CRU of 21 mongrel dogs. After full recovery from diaphragm segmental shortening, the animals were studied awake, breathing through a mask. Airflow, EtCO_2_, heart rate, muscle length and moving averaged EMG (mavgEMG) were recorded during room air and CO_2_ stimulated breathing (3). Output included breath-by-breath breathing pattern, muscle Shortening (% of change in Length from baseline), Peak EMG (ΔPeakEMG, % of baseline-to-peak difference in mavgEMG), EtCO_2_ and heart rate.

**Results:** For N = 21 (mean wgt 31.1 kg) studied after 16 days post-implantation (range 10–23 days) of Sonomicrometry transducers and EMG electrodes, minute ventilation (Vi) and tidal volume increased significantly from room air with CO_2_ stimulated breathing [8.8 and 0.3, 10.2 and 0.4, 14.6 and 0.6, and 18.2 and 0.7 and 22.9 and 0.8 at room air, low (47), medium-low(53), medium-high (58) and high (63 Torr) CO_2_, respectively]. Respiratory rate did not significantly change (range 25–29). As CO_2_ increased, ΔPeakEMG of COS and CRU increased conjointly in a linear fashion tracking changes in Vi,. however, there was a significantly (p < 0.05) greater increasing in ShortCOS compared to ShortCRU at medium-low, medium-high and high levels of CO_2_.

**Conclusions:** With CO_2_ stimulation, neural drive of COS and CRU increased together in lock-step with ventilation. However, there was distinctive differential action of the diaphragm segments. Relative mechanical action of COS was significantly higher than CRU at equivalent increasing of CO_2_. This results suggest a predominant contribution of the Costal portion of the diaphragm in order to generate the tidal volume during hypercapnic stimulation.

**References**

**(1)** Lopata M. Journal of Applied Physiology, 1977, 43(2):262–70

**(2)** DeTroyer A. Science, 1981 Jul 10;213(4504):237–8.

**(3)** Easton PA. Journal of Applied Physiology, 1993, 74(3):1406–18

**Grant acknowledgement**

Excellent laboratory assistance and support from colleagues : Jenny V. Suneby Jagers and Michael Ji.

**Fig. 84 (abstract A669).**
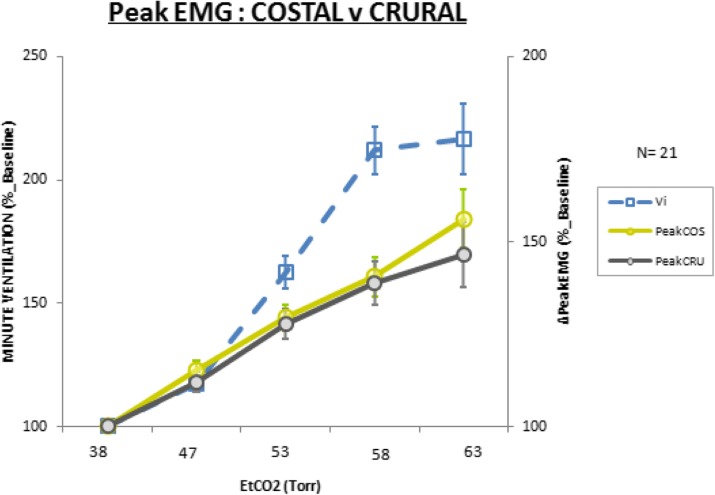
Peak EMG : COSTAL v CRURAL

**Fig. 85 (abstract A669).**
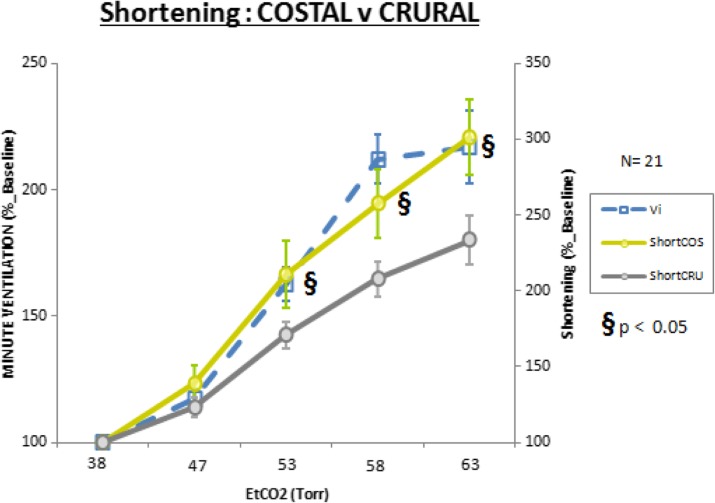
Shortening : COSTAL v CRURAL

#### A670 Pulmonary dysfunction pattern in sepsis survivors.an experimental study

##### R.B. Souza^1^, A.M.A. Liberatore^2^, A.M.C.R.P.F. Martins^3^, J.C.F. Vieira^2^, Y.R. Kang^2^, M.N. Nakamae^2^, I.H.J. Koh^2^

###### ^1^Federal University of São Paulo, Morphology and Genetics, São Paulo, Brazil; ^2^Federal University of São Paulo, Surgery, São Paulo, Brazil; ^3^Biological Institute of São Paulo, São Paulo, Brazil

####### **Correspondence:** R.B. Souza - Federal University of São Paulo, Morphology and Genetics, São Paulo, Brazil

Sepsis is frequently complicated by organ dysfunction and the lungs seem to be particularly vulnerable to the inflammatory response. The disabling fatigue and the higher susceptibility to developing a viral respiratory infection that many survivors experience in post-sepsis syndrome cannot yet be explained. Herein, we sought to evaluate the impact of sepsis on lung structures of the post-sepsis survivors.

**Methods:** Adult Wistar rats (200 g) were submitted to sepsis [iv. 2 mL *E. coli* 10^8^ (S8) or 10^9^ CFU/ mL (S9), DL60 and DL80, respectively, in 26 hours].Under general anesthesia, the lung architecture was monitored by histology at 6 hours after sepsis (T6h, n = 3/group) and 30^th^ day survivals (T30d, n = 3/group). The lungtissue wasexaminedafter staining with H.E.

**Fig. 86 (abstract A670).**
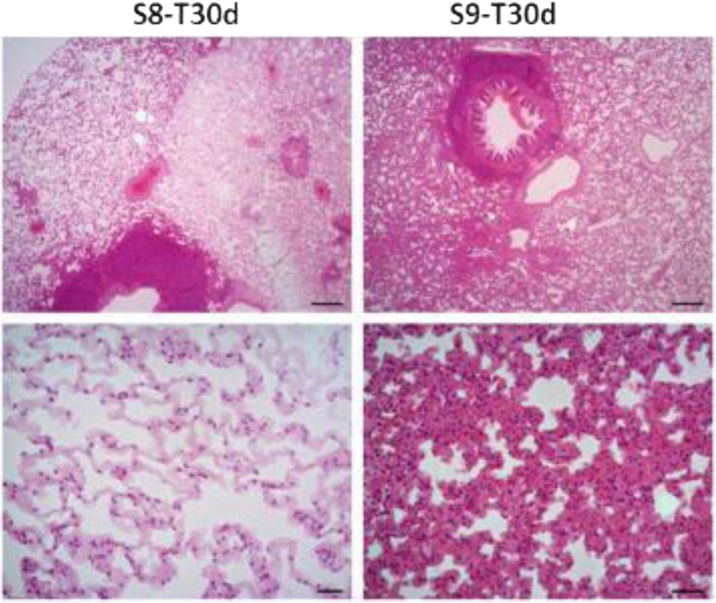
ᅟ

**Results:** The S8-6 h group showed a vascular congestion of the alveolar wall, alveolar wall thickening and neutrophils infiltration.It was also observed an increase in cellularity in the bronchial lumen and BALT hyperplasia (Fig. 86). After 30 days was observed a reduction of the alveolar wall thickening, complete reduction of alveoli vascular congestion in the entire lung, however, there was a persistence of the hyperplasia of BALT. In animals submitted to more severe sepsis (S9-30d), the alveolar wall thickening remained more intense, there was decreased of inflammatory cells infiltrates, but persisted the alveoli wall congestion and BALT hyperplasia and hypertrophy (Fig. 2). These findings showed that depending on the severity of sepsis, the parenchyma and pulmonary microcirculation injuries may have significant recovery (S8) or minimal (S9), even 30 days after the clinical recovery from sepsis. In addition, presence of histological signs suggestive of persistence of a state of the activated BALT, even in less aggressive sepsis (S8-30d), shows that still exist a continuous stimulating inflammatory factor even in the absence of clinical signs of infection. Thus, the overall data demonstrate that clinical recovery is not synonymous of a normal physiological state, on the contrary, histology showed an ongoing severe lung disease process at post-sepsis period. How long last these eventsis unknown, but the data suggest that post-sepsis physiological state is far from the standard health.

**Grant acknowledgement**

FAPESP 2011/20401-4.

#### A671 Inhibition of EphA2/Ephrinea1 signal attenuates lipopolysaccharide-induced lung injury

##### J.Y. Hong^1^, M.H. Shin^2^, M.S. Park^2^

###### ^1^Hallym University Medical Center, Chuncheon, Republic of Korea; ^2^Yonsei University College of Medicine, Seoul, Republic of Korea

####### **Correspondence:** J.Y. Hong - Hallym University Medical Center, Chuncheon, Republic of Korea

**Introduction:** Eph-Ephrin signaling mediates various cellular processes, including vascular permeability, inflammation, angiogenesis, cell migration, axon guidance, fluid homeostasis, and repair after injury. The detailed mechanisms of EphA2 signaling in lung injury are unknown.

**Objectives:** To evaluate the role of EphA2 signaling, as well as related signaling pathways, in the lipopolysaccharide (LPS)-induced lung injury model.

**Methods:** Three experimental mouse groups were formed: PBS + IgG (IgG instillation after PBS exposure), LPS + IgG (IgG instillation after LPS exposure), and LPS+ EphA2 mAb (EphA2 monoclonal antibody instillation posttreatment after LPS exposure). Cell numbers and protein concentration in the bronchoalveolar lavage fluid (BALF), changes in histopathology and the expression of several signaling pathway proteins—including PI3K-Akt-NF-kB, Src, Erk, E-cadherin and mTOR—were compared among the three groups.

**Results:** Acute LPS exposure significantly upregulated EphA2 and EphrinA1 expression. Inhibition of the EphA2 receptor by intranasal EphA2 mAb administration attenuated lung injury and reduced BALF cell counts and protein concentration (all, P < 0.05). EphA2 mAb posttreatment downregulated the expression of PI3K 110γ, phospho-Akt, phospho-NF-kB, Erk1/Erk2, phospho-Src, and phospho-S6K. In addition, inhibiting the EphA2 receptor augmented the expression of E- cadherin, which is involved in cell-cell adhesion.

**Conclusions:** The present data suggest that the EphA2 receptor may be an unrecognized modulator of several signaling pathways—including PI3K-Akt-NF-kB, Src-NF-kB, E-cadherin, and mTOR—in LPS-induced lung injury. Further studies are needed to verify the potential of EphA2 receptor inhibitors as novel therapeutic agents for LPS-induced lung injury.

**References**

1. Coulthard MG, Morgan M, Woodruff TM, Arumugam TV, Taylor SM, Carpenter TC, Lackmann M, Boyd AW. Eph/Ephrin signaling in injury and inflammation. *The American journal of pathology* 2012; 181: 1493–1503.

2. Carpenter TC, Schroeder W, Stenmark KR, Schmidt EP. Eph-A2 promotes permeability and inflammatory responses to bleomycin-induced lung injury. American journal of respiratory cell and molecular biology 2012; 46: 40–47.

3. Cercone MA, Schroeder W, Schomberg S, Carpenter TC. EphA2 receptor mediates increased vascular permeability in lung injury due to viral infection and hypoxia. American journal of physiology Lung cellular and molecular physiology 2009; 297: L856-863.

4. Ivanov AI, Steiner AA, Scheck AC, Romanovsky AA. Expression of Eph receptors and their ligands, ephrins, during lipopolysaccharide fever in rats. Physiological genomics 2005; 21: 152–160.

**Grant acknowledgement**

This study was financially supported by the "Kiturami" Faculty Research Assistance Program of Yonsei University College of Medicine for 2012(6-2012-0149)

#### A672 Minimal arterial blood gas analysis based on the average carbon dioxide elimination during automatic ventilation therapy using the ARDSNet protocol

##### A. Pomprapa^1^, P.A. Pickerodt^2^, M.B.T. Hofferberth^2^, M. Russ^2^, W. Braun^3^, M. Walter^1^, R. Francis^2^, B. Lachmann^2^, S. Leonhardt^1^

###### ^1^Chair for Medical Information Technology, RWTH Aachen University, Aachen, Germany; ^2^Department of Anesthesiology and Intensive Care Medicine, Campus Charité Mitte and Campus Virchow-Klinikum, Charité-University Medicine, Berlin, Germany; ^3^Fritz Stephan GmbH, Gackenbach, Germany

####### **Correspondence:** A. Pomprapa - Chair for Medical Information Technology, RWTH Aachen University, Aachen, Germany

**Introduction:** One of the therapeutic ventilation goals of the ARDS Network protocol is to control blood pH in a certain range (7.30-7.45) [1]. Only a strict and frequent schedule of arterial blood gas analysis can result in a good performance for such control, which mainly relies on personal efforts.

**Objective:** This preliminary work was to analyze the possibility to control blood pH via the average carbon dioxide elimination (*VCO*_*2*_) that can be noninvasively measured from the exhaled air during the closed-loop ventilation therapy [2].

**METHOD.** Approved by the local animal ethical committee, a male pig (45.4 kg) was anesthetized, intubated and ventilated in supine position for 1 h. After induction of ARDS by repetitive lung lavages, automatic ventilation therapy was continued for 3 h. All vital parameters including the CO_2_ data from a capnography (CO_2_SMO+) were continuously recorded by a medical panel PC. Based on the ARDSNet protocol, higher PEEP/lower FiO_2_ settings [1] were automatically applied to the pig. After the onset of the automatic ventilation, the average *VCO*_*2*_ was computed for every 30 min.

**Results:** All goals of the ARDSNet protocol were satisfied in terms of oxygenation, pH value and plateau pressure. The figure shows the experimental results of the pH value compared to the computed average V*CO*_*2*_ during the ventilation therapy. It should also be noted that a new setting of PEEP value influences the dynamics of *VCO*_*2*_.

**Conclusions:** The carbon dioxide elimination is a possible intermediate parameter for the use of noninvasive indirect control of blood pH that could fulfil a fully closed-loop ventilation therapy based on the ARDSNet protocol.

**References**

[1] Brower RG, et al., Higher versus lower positive end-expiratory pressures in patients with the acute respiratory distress syndrome. N Engl J Med. 2004; 351(4):327–36.

[2] Pomprapa A, et al., Automatic protective ventilation using the ARDSNet protocol with the additional monitoring of electrical impedance tomography. Critical Care; 18:R128.

**Grant acknowledgement**

German Federal Ministry of Economics and Technology (BMWi-ZIM)

**Fig. 87 (abstract A672).**
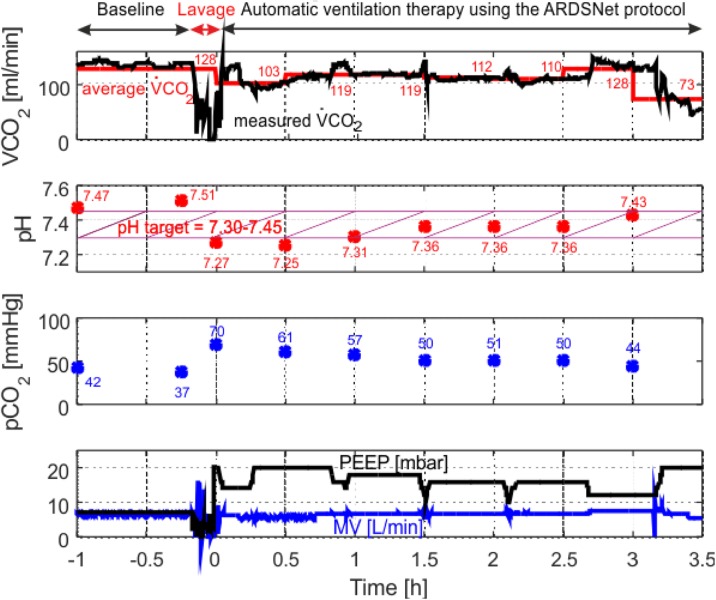
Automatic ARDSNet ventilation therapy

#### A673 Early aggressive-fluid therapy and lung: consequences in sepsis survivors. An experimental study

##### I.H.J. Koh^1^, R.B. Souza^2^, A.M.C.R.P.F. Martins^3^, J.C.F. Vieira^1^, A.M.A. Liberatore^1^

###### ^1^Federal University of São Paulo, Surgery, São Paulo, Brazil; ^2^Federal University of São Paulo, Morphology and Genetics, São Paulo, Brazil; ^3^Biological Institute of São Paulo, São Paulo, Brazil

####### **Correspondence:** I.H.J. Koh - Federal University of São Paulo, Surgery, São Paulo, Brazil

Sepsis is a leading cause of acute respiratory distress syndrome (ARDS), characterized by diffuse injury of the alveoli-capillary wall, increased pulmonary vascular permeability, and alveolar and interstitial edema. The aggressive fluid therapy is a controversial procedure by the possibility of increment the lung edema. Besides, due to the difficulties to study the lung, the consequences in the tissue injuries in sepsis patients in the post-sepsis phases are little known. The present study investigated the effects of aggressive-fluid therapy in lung tissues of the sepsis survivors.

**Fig. 88 (abstract A673).**
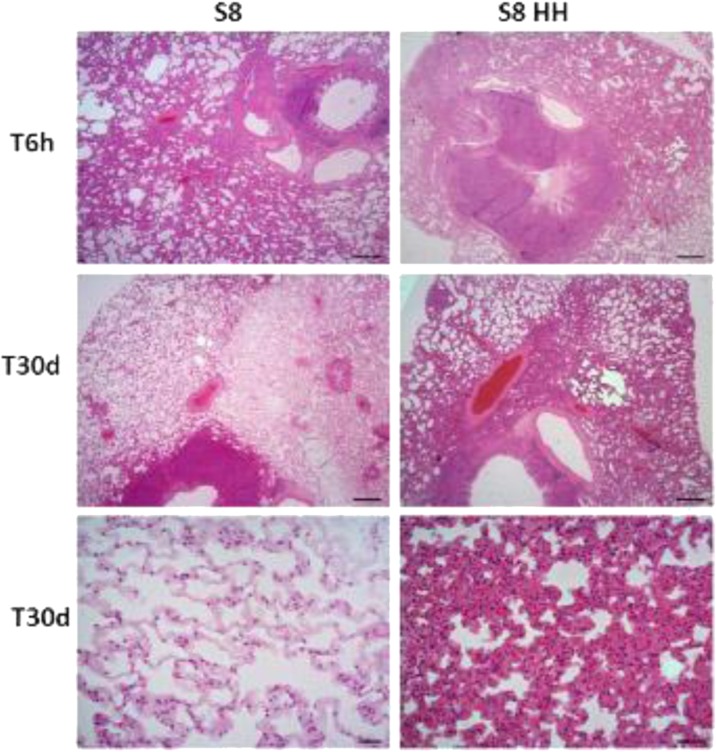
ᅟ

**Methods:** Adult Wistar rats (200 g) were submitted to sepsis (S8) (iv. 2 mL *E. coli* 10^8^ CFU/mL_−1_, DL60 in 26 hours), or sepsis and Ringer Lactate (S8HH) (iv. 30 mL/kg/20 min), 30 minutes after sepsis challenge. The tissue samples were evaluated by histology (T6h,T30d), using HE dyes.The S8-6 h group showed a vascular congestion on the alveoli walls, withintense thickening and infiltration of neutrophils, and increased cellular invasioninto the bronchus lumen. BALT showed intense hyperplasia. InS8-30d animals,there was a reduction in thickening of the alveoli wall, the absence of thecapillary congestion throughout the lung, but with cellular debris in the bronchial lumenand a persistent BALT hyperplasia (Fig. 88). The aggressive fluid therapy in S8HH-6 h group resulted in lesser vascular congestion in the peripheral alveolar walls. More pronounced lesions were seenin the central part of the organ (hilo); with venous congestion and thickening of the alveoli-wall, infiltration of mononuclearcells and neutrophils in intra epithelia's spaces of the bronchi; BALT hyperplasia was highly evident. The aggressive fluid didnot recovered the injuries of the S8HH-30d group. the vascular congestion and thickened parenchyma were observed in whole lung, although without inflammatory infiltrate. BALT showed less hypertrophy and hyperplasia. In summary, although there was a worsening of the alveoli injuries, the signs of non-activated BALT suggested low immune activation under aggressive-fluid therapy but with high pulmonary edema due to their high permeability characteristics. The overall data suggested that lung not have adequate physiology and anatomy to support a fluid aggressive therapy demanding a more careful maneuver in terms of volume and perfusion velocity. These results reinforce the doubts that inadequate management of sepsis may cause consequences in the future.

**References**

1. Koh Ivan HJ, et al.: Shock 2010, 34 (7 Suppl 1):27–33.

2. De Backer D, et al.: Intensive Care Med, 2010, 36:1813–1825.

3. Sheu CC, et al. Chest, 2010, 138(3):559–567

**Grant acknowledgement**

FAPESP 2011/20401-4.

#### A674 Usefulness of haematocrit as a predictor of failure in weaning from invasive mechanical ventilation

##### A. Landaverde-López, N.A. Canedo-Castillo, A. Esquivel-Chávez, P.C. Arvizu-Tachiquín, L.A. Sánchez-Hurtado, J.A. Baltazar-Torres

###### Hospital de Especialidades, Centro Médico La Raza, IMSS, Intensive Care Unit, Mexico, Mexico

####### **Correspondence:** N.A. Canedo-Castillo - Hospital de Especialidades, Centro Médico La Raza, IMSS, Intensive Care Unit, Mexico, Mexico

**Introduction:** During weaning from invasive mechanical ventilation (IMV), pulmonary oedema may develop due to cardiac failure, and it is a cause of failure in weaning from IMV. Cardiogenic pulmonary oedema produces hemoconcentration and haematocrit (Ht) is a good marker of hemoconcentration. It is not determined if the increase of Ht during weaning protocol may predict failure in weaning from IMV.

**Objective:** To determine if the increase of Ht during spontaneous breathing trial (SBT) is a predictor of failure in weaning from IMV.

**Methods:** We prospectively included patients with IMV >72 hours. A SBT was performed with a T-piece for two hours. Ht value at the start and at the end (or failure) of the SBT was measured and the delta was calculated. Logistic regression analysis was performed to determine the association between the increase of Ht and failure in weaning from IMV. Its performance as a predictor of failure was assessed by its discriminative capacity and calibration. A p value < 0.05 was considered statistically significant.

**Results:** Fifty-five patients were analysed, 58.2 % female, mean age 49.9 years. Thirty-two (58.2 %) patients had elevation of the Ht at the end of the SBT and 41.8 % failed in weaning from IMV. The increase of Ht had OR of 1.22 (95%CI 1.05-1.42, p = 0.011) and behaved as a good predictor of failure in weaning from IMV, with area under the receiver operative characteristics (ROC) curve of 0.719 (p = 0.006) and Hosmer-Lemeshow Chi^2^ of 41 (p = 0.142).

**Conclusions:** Patients who failed in weaning from IMV has significantly increased the value of Ht at the end of the SBT. The increase of Ht is a risk factor and has adequate discriminative capacity and calibration to predict failure in weaning from IMV.

Key words: Failure of weaning from mechanical ventilation, spontaneous breathing trial, haematocrit.

**References**

1) Boles JM, Bion J, Connors A, et al. Weaning from mechanical ventilation. Eur Respir J 2007;29(5):1033–56

2) McConville J. Kress, J. Weaning patients from the ventilator. N Engl J Med 2012;367(23):2233–9

3) Teboul JL. Weaning-induced cardiac dysfunction: where are we today? Intensive Care Med 2014;40(8):1069–79

4) Grasso S, Pisani L. Weaning and the heart: from art to science. Crit Care Med 2014;42(8):1954–5

5) Anguel N, Monnet X, Osman D, et al. Increase in plasma protein concentration for diagnosing weaning-induced pulmonary oedema. Intensive Care Med 2008;34:1231–38

6) Aman J, Van der Heijden M, Van Lingen A, et al. Plasma protein levels are markers of pulmonary vascular permeability and degree of lung injury in critically ill patients with or at risk for acute lung injury/acute respiratory distress syndrome. Crit Care Med 2011;39(1):89–97

7) Figueras J. Weil M. Increases in plasma oncotic pressure during acute cardiogenic pulmonary edema. Circulation 1977;55:195–199.

#### A675 Adherence to bundles and protocols may reduce VAP incidence

##### V. Cardoso^1^, A. Krystopchuk^2^, S. Castro^2^, L. Melão^2^, S. Firmino^2^, A. Marreiros^1^, C. Granja^1,2,3^

###### ^1^University of Algarve, Department of Biomedical Sciences and Medicine, Faro, Portugal; ^2^Centro Hospitalar do Algarve, Hospital de Faro, Emergency and Intensive Care Department, Faro, Portugal; ^3^Faculty of Medicine of Porto, CINTESIS, Porto, Portugal

####### **Correspondence:** V. Cardoso - University of Algarve, Department of Biomedical Sciences and Medicine, Faro, Portugal

**Introduction:** With a high prevalence in Intensive Care Units (ICU), the Ventilator-Associated pneumonia (VAP) has been responsible for increased mortality and morbidity rates, hospital stay, duration of mechanical ventilation (MV), costs and a set of other complications. Usually, VAP develops 48 h after the beginning of mechanical ventilation (MV) and can be classified as of early- or late-onset.

**Objectives:** This study aims to evaluate the incidence, length of hospital stay, number of days of MV, and treatment and prevention methods (bundles) of VAP during the year of 2015, in the ICU of CHA-Faro, as well as to compare these results with those from 2014.

**Methods:** Patients were evaluated according to their age, sex, type of VAP (early-/late-onset), identified microorganism, antibiotic treatment, admission type and severity scores (APACHE II and SAPS II), as well as the assessment of the likelihood of lung infection (CPIS).

**Results:** Of the 473 patients admitted to the ICU, 24 developed VAP. The incidence rate was 5.07 %, and the incidence density rate was 9.74 per 1000 ventilation days. The mean value of CPIS was 1.35 ± 7.5. Five cases were classified as early-onset (20.83 %) and 19 as late-onset (79.17 %). The mean values of both, hospital stay (7.12 ± 9.97 and 29.83 ± 21.35) and MV (5.21 ± 9.26 and 26.17 ± 23.05), were significantly different (p < 0.01) between patients without and with VAP, respectively. The most common isolated microorganism was P. aeruginosa (57.9 %) in late-onset VAP, with no microorganisms isolated (60 %) in the early-onset VAP. The adherence to VAP prevention bundles from January to October 2015 was 14,91 %.

**Conclusions:** Our data indicate that the incidence of VAP was reduced from 2014 to 2015. The increase on adherence to VAP bundles might explain this signigficant reduction. Decrease on VAP incidence was significantly asociated with reduction on days of mechanical ventilation and length of hospital stay.

#### A676 Correlation between antibiotic class and the incidence of ventilator associated pneumonia in an American hospital

##### S. Almaziad, A. Kubbara, W. Barnett, R. Nakity, W. Alamoudi, R. Altook, T. Tarazi, M. Fida, F. Safi, R. Assaly

###### University of Toledo Medical Center, Toledo, United States

####### **Correspondence:** S. Almaziad - University of Toledo Medical Center, Toledo, United States

**Introduction:** Patients who are on mechanical ventilation have an increased risk of developing Ventilator associated event (VAE) which is tiered into three definitions; Ventilator-Associated Condition (VAC), Infection-related Ventilator-Associated Complication (IVAC) and Ventilator- associated pneumonia (VAP). VAP is a rare yet serious complication that carries a high risk of morbidity and mortality. Current guidelines, published in 2005 by the American Thoracic Society in association with the Infectious Diseases Society of America, recommend the use of empiric antibiotics therapy.

**Objective:** This is an ongoing research with the aim of examining the trends in the management of VAC and minimizing the use of vancomycin in patients on mechanical ventilation due to the higher risk of developing multi-drug resistant organisms and as a consequence, deteriorating to IVAC and VAP as compared to other antibiotics.

**Methods:** A retrospective analysis of data collected from 858 patients admitted to the intensive care unit at the University of Toledo Medical Center and received empiric antibiotic therapy. Among the sample of patients, 152 met CDC-defined criteria for a ventilator-associated event. Additionally, those that met VAE criteria were stratified by VAE type, which yielded 82 VACs, 38 IVACs, and 32 PVAPS.

**Results:** Vancomycin was used frequently in patients on mechanical ventilation. Among 706 patients who did not develop VAE, it was used in 62.5 %. Furthermore, it was used in 80.5 %, 94.7 % and 81.3 % in patients who developed VAC, IVAC and VAP, respectively and 84.2 % collectively. Overall, the primary use of three antibiotic groups (Penicillin, cephalosporin and vancomycin) is consistent through each stage of VAE and likewise in the non-VAE group; cephalosporin was used in 63.2 % in the VAE group and 51.6 % in non-VAE while penicillin was used in 59.2 % in the VAE group and 43.3 % in the non-VAE group. In addition, the use of Vancomycin either alone or in combination with other antibiotics is associated with VAEs (OR = 3.2).

**Conclusion** By CDC definition, those that have met IVAC or PVAP criteria would have been on a minimum of 4 days of antimicrobial therapy. The extensive use of Vancomycin in critically-ill patients compared to other antibiotics can be a contributing factor in the development of resistant organisms leading to subsequent complications.

**Table 65 (abstract A676).** Antibiotics usage

Antibiotic	VAC (n=82)	IVAC (n=38)	PVAP (n=32)	VAE (n=152)	Non-VAE (n=706)
PCN	44 (53.7%)	22 (57.9%)	24 (75.0%)	90 (59.2%)	306 (43.3%)
Cephalosporin	52 (63.4%)	21 (55.3%)	23 (71.9%)	96 (63.2%)	364 (51.6%)
Vancomycin	66 (80.5%)	36 (94.7%)	26 (81.3%)	128 (84.2%)	441 (62.5%)
Macrolides	19 (23.2%)	6 (15.8%)	4 (12.5%)	29 (19.1%)	83 (11.8%)
Quinolones	31 (37.8%)	16 (42.1%)	17 (53.1%)	64 (42.1%)	172 (24.4%)
Carbapenems	40 (48.8%)	17 (44.7%)	16 (50.0%)	73 (48%)	99 (14.0%)
Metronidazole	37 (45.1%)	19 (50.0%)	17 (53.1%)	73 (48%)	147 (20.8%)

**Fig. 89 (abstract A676).**
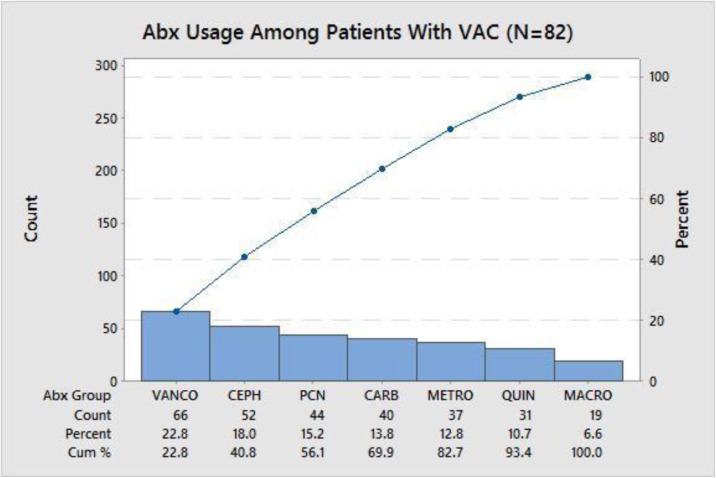
Antibiotics usage among patients with VAC

**Fig. 90 (abstract A676).**
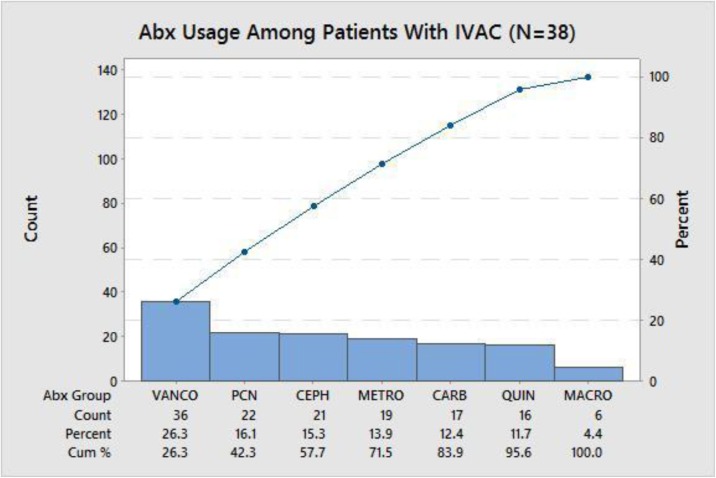
Antibiotics usage among patients with IVAC

**Fig. 91 (abstract A676).**
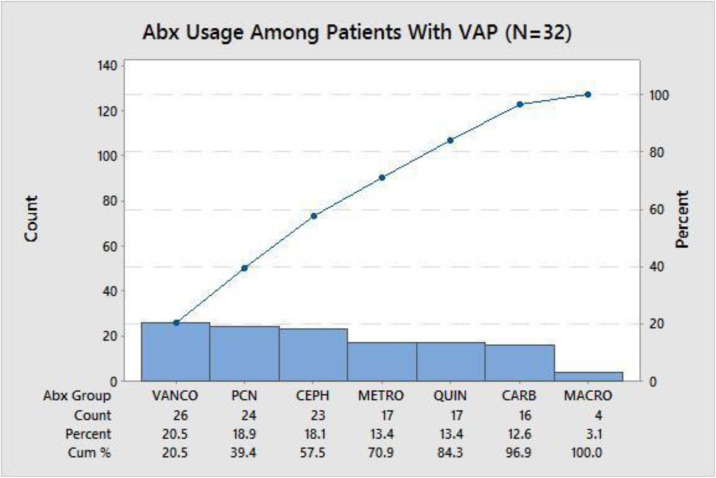
Antibiotics usage among patients with VAP

#### A677 Role of static and dynamic driving airway pressure in the development of ventilator-induced lung injury

##### A. Santini^1^, M. Milesi^2^, T. Maraffi^2^, P. Pugni^2^, D.T. Andreis^2^, M. Cavenago^3^, L. Gattinoni^1,2^, A. Protti^1^

###### ^1^Fondazione IRCCS Ca' Granda Ospedale Maggiore Policlinico, Dipartimento di Anestesia, Rianimazione ed Emergenza Urgenza, Milan, Italy; ^2^Università degli Studi di Milano, Dipartimento di Fisiopatologia Medico-Chirurgica e dei Trapianti, Milan, Italy; ^3^Fondazione IRCCS Ca' Granda Ospedale Maggiore Policlinico, Centro di Ricerche Chirurgiche Precliniche, Milan, Italy

####### **Correspondence:** A. Santini - Fondazione IRCCS Ca' Granda Ospedale Maggiore Policlinico, Dipartimento di Anestesia, Rianimazione ed Emergenza Urgenza, Milan, Italy

**Introduction:** Driving airway pressure, the difference between plateau (end of an inspiratory pause) and end-expiratory airway pressure, can be an independent predictor of ventilator-induced lung injury (VILI) [1]. However, during ongoing mechanical ventilation, (dynamic) end-inspiratory airway pressure exceeds (static) plateau airway pressure, especially when inspiratory flows are very high [2]. Thus, for the same *static* driving airway pressure, different *dynamic* driving airway pressures can be reached.

**Objectives:** To compare the role of static and dynamic driving airway pressures in the development of VILI.

**Methods:** Sixteen healthy piglets were anesthetized, paralyzed and mechanically ventilated for 54 hours with no positive end-expiratory pressure. Animals were divided into two groups that were matched for *static* driving airway pressure (ΔP_AW_,_stat_), defined as above, but not for *dynamic* driving airway pressure (ΔP_AW_,_dyn_), defined as the difference between airway pressure recorded when flow zeroed immediately after an end-inspiratory occlusion (P1) and end-expiratory airway pressure. Lower and higher dynamic driving airway pressures were obtained by using lower or higher inspiratory flows. Respiratory rate was always 15 breath-per-minute. VILI was diagnosed if final lung weight (measured with a balance) exceeded initial lung weight (measured with computed tomography), as for edema. Data were compared between the two groups with Student's *t* and Fisher's Exact tests.

**Results:** The two groups of animals were ventilated with similar static but different dynamic driving airway pressures (Table 66). Incidence of VILI was 25 % among animals ventilated with lower and 75 % among those ventilated with higher dynamic driving airway pressure (p = 0.13). On average, lung weight decreased over time (−56 ± 60 g) in the former group whereas it increased (98 ± 166 g) in the latter group (p = 0.04). Mortality at 54 hours was 13 % among animals ventilated with lower and 63 % among those ventilated with higher dynamic driving airway pressure (p = 0.12).

**Conclusions:** For the same static driving airway pressure, higher dynamic driving airway pressures may increase the incidence of (fatal) ventilator-induced lung injury.

**References**

1. Amato MBP, Meade MO, Slutsky AS, et al. (2015) Driving Pressure and Survival in the Acute Respiratory Distress Syndrome. N Engl J Med 372:747–755. doi: 10.1056/NEJMsa1410639

2. Protti A, Maraffi T, Milesi M, et al. (2016) Role of Strain Rate in the Pathogenesis of Ventilator-Induced Lung Edema. Critical Care Medicine. doi: 10.1097/CCM.0000000000001718

**Table 66 (abstract A677).** **ᅟ**

	Low ΔP_AW,dyn_ (n=8)	High ΔP_AW,dyn_ (n=8)	p
Tidal Volume, ml	633 ± 143	553 ± 158	0.31
Inspiratory flow, ml/sec	569 ± 161	1365 ± 268	<0.01
Static driving airway pressure, cmH_2_O	22.4 ± 2.7	22.6 ± 2.8	0.50
Dynamic driving airway pressure, cmH_2_O	25.6 ± 3.3	27.9 ± 5.1	0.03

#### A678 Study of alveolar kinetics by synchrotron radiation computed tomography

##### G. Perchiazzi^1,2^, J.B. Borges^1^, S. Bayat^3^, L. Porra^4^, L. Broche^1,5^, M. Pellegrini^1,2^, G. Scaramuzzo^6^, G. Hedenstierna^1^, A. Larsson^1^

###### ^1^Uppsala University, Department of Medical Sciences - Hedenstierna Laboratory, Uppsala, Sweden; ^2^Università di Bari Aldo Moro, Department of Emergency and Organ Transplant, Bari, Italy; ^3^Université de Picardie Jules Verne, Laboratoire Peritox EA -INERIS, Amiens, France; ^4^University of Helsinki, Department of Physics, Helsinki, Finland; ^5^European Synchrotron Radiation Facility, Grenoble, France; ^6^University of Ferrara, Morphology, Surgery and Experimental Medicine, Ferrara, Italy

####### **Correspondence:** G. Perchiazzi - Università di Bari Aldo Moro, Department of Emergency and Organ Transplant, Bari, Italy

**Introduction:** The sequence of events that at alveolar level determine lung inflation are only partially known. Methods that can provide simultaneously high-resolution, in-vivo conditions and information about the core region of the lungs are lacking. Ventilatory strategies to support lung function rely upon the idea that lung alveoli are isotropic balloons that accommodate gas progressively and that pressure/volume curves derive by the interplay of opening pressures and position of alveoli inside the lung. This simplistic idea has been challenged by subpleural microscopy^1^, magnetic resonance imaging^2^ and computed tomography (CT) studies^3,4^. Images of in-vivo lungs can be obtained from Synchrotron Radiation Computed Tomography (SRCT) at resolutions higher than conventional CT.

**Objective:** To evaluate kinetics of airspaces in healthy (HC) and Acute Respiratory Distress Syndrome (ARDS) conditions.

**Methods:** The study was conducted in seven anesthetized New Zealand rabbits. They underwent SRCT scans (resolution of 47.6 μm)of the lung at decreasing PEEP levels of 12, 9, 6, 3 and 0 cmH_2_O during end-expiratory holds. Pulmonary imaging was executed during HC and after induction of ARDS by repeated lung lavages and injurious ventilation (inspiratory pressure of 35 and PEEP = 0 cmH_2_O). Three concentric regions-of-interest (ROI) were studied: subpleural (SP), peripheral (PE) and core (CO). Images were enhanced by phase contrast algorithms. Airspaces Number (AN), covered area (AA) and dimensions (AD) were computed by using the Image Processing Toolbox for MatLab (Mathworks, Natick, USA). Student's T-test was used to assess any statistically significant difference produced by PEEP or ROI position.

**Results:** AN and AA decreased with decreasing PEEP, however in ARDS at PEEP lower than 6 cmH_2_O they remained stable. The three ROIs had similar courses but with different magnitudes. AD during HC showed a tendency to reduce progressively after 9 cmH_2_O; during ARDS this tendency was observed sharply after 6 cmH_2_O.

**Conclusions:** Alveolar kinetics is different between healthy and ARDS conditions. In HC and ARDS de-recruitment and isotropic deflation are both present, however their entity and proportion depend on the applied pressure. In HC, alveolar dimensions in the studied range support the idea of a continuous derecruitment simultaneous with isotropic deflation. In ARDS, passage from PEEP 12 to 6 cmH_2_O is characterized by a sharp derecruitment (alveoli do not change appreciably dimensions but can be either open or closed). In ARDS lungs this may be consequence of their high critical closing pressures and elastance.

**References**

1. Dirocco JD, et al.: *Intensive Care Med* 2007;33:1204–1211

2. Hajari AJ, et al.: *J Appl Physiol* 2011;112 :937–943

3. Kaczka DW et al.:*Ann Biomed Eng* 2011;39:1112–24

4. Perchiazzi G et al.: *Respir Physiol Neurobiol* 2014;201:60–70

**Grants**

Bari University: Master in Critical Care; The Swedish Research Council; The Heart Lung Fund; The ESRF.

#### A679 Spontaneous breathing compared with mechanical ventilation improves lung aeration and reduces lung collapse in experimental mild acute respiratory distress syndrome

##### M. Pellegrini^1,2^, G. Hedenstierna^3^, A. Roneus^1^, M. Segelsjö^4^, M.C. Vestito^2^, A. Larsson^1^, G. Perchiazzi^1,2^

###### ^1^Uppsala University, Hedenstierna Laboratory, Department of Surgical Sciences, Uppsala, Sweden; ^2^Bari University, Department of Emergency and Organ Transplant, Bari, Italy; ^3^Uppsala University, Hedenstierna Laboratory, Department of Medical Sciences, Uppsala, Sweden; ^4^Uppsala University, Section of Radiology, Department of Surgical Sciences, Uppsala, Sweden

####### **Correspondence:** M. Pellegrini - Bari University, Department of Emergency and Organ Transplant, Bari, Italy

**Introduction:** We have previously found that during spontaneous breathing (SB) the diaphragm exerts an expiratory breaking effect, maintaining lung volume. In this experimental study in a porcine mild acute respiratory distress (ARDS) model we compared the effect on lung aeration between full mechanical ventilation (MV) and SB and our hypothesis was that SB, due to the breaking activity by the diaphragm, would improve intraparenchymal distribution of gas during expiration and reduce atelectasis formation.

**Methods:** A mild acute respiratory distress syndrome was induced in five anesthetized, tracheostomized pigs by repeated lung lavages, targeting a PaO_2_/FiO_2_ of 250 mmHg. After stabilization, the animals were allowed to breathe spontaneously and underwent a decremental continuous positive end-expiratory pressure (CPAP/PEEP) trial at 15, 12, 9, 6, 3 and 0 cmH_2_O. Then the same sequence was repeated during MV after muscle relaxation. Para-diaphragmatic expiratory dynamic CT-scans were collected four (L1) and one (L2) cm cranial to the diaphragm. The amount of gas (Vgas, [mL]) for each CT-image at half and end expiration was calculated in all the compartments from +100 to −1000 Hounsfield Units (HU). The amount of atelectasis (between −100 and +100 HU) for each selected CT-image was also measured. Paired samples Student t-test was applied to assess any statistically significant difference between study conditions.

**Results:** At CPAP/PEEP levels equal or lower than 6 cmH_2_O, Vgas was higher during SB than during MV at all distances from the diaphragmatic dome and, in addition, at both half and end-expiration. The increase in atelectasis with decreasing CPAP/PEEP was in all animals larger during MV than SB. Only during SB, and not during MV conditions, the lung near the diaphragmatic dome (L2) was less atelectatic than the lung further away from it (L1). More atelectasis during MV than SB was observed already at half-expiration.

**Conclusions:** SB maintains the amount of lung aeration significantly better than MV in a collapse-prone mild ARDS model. Furthermore, SB protected against atelectasis formation: this effect was visible already at half expiration.

Therefore we deduce that the improved redistribution of gas and the decreased atelectasis formation depend on the preserved diaphragmatic contraction. These findings have potential implications for the design of new ventilatory strategies.

**Grant acknowledgement**

The Swedish Heart Lung Fund; The School of Anesthesia of Bari University.

#### A680 Cerebral availability and usage of oxygen in pigs with sepsis: can lung protective ventilation protect the brain?

##### E. Gremo^1^, A. Nyberg^1,2^, M. Castegren^2,3^, A. Pikwer^1,2^

###### ^1^Mälarsjukhuset, Clinic for Anesthesiology and Intensive Care, Eskilstuna, Sweden; ^2^Uppsala University, Centre for Clinical Research Sörmland, Uppsala, Sweden; ^3^Karolinska University Hospital, Department of Anaesthesia, Intensive Care and Surgical Services, Stockholm, Sweden

####### **Correspondence:** E. Gremo - Mälarsjukhuset, Clinic for Anesthesiology and Intensive Care, Eskilstuna, Sweden

**Introduction:** Sepsis is a common cause for admission to the ICU and more than a quarter who suffer from septic shock dies (1). Severe sepsis and septic shock leads to failure of one or more organs. The brain can get affected by septic induced organ failure, a condition known as sepsis-associated encephalopathy (SAE).

Protective ventilation (PV), with tidal volumes (TV) of 6 ml/kg, have showed a decrease in mortality in patients with sepsis and acute respiratory distress syndrome, in comparison to routine ventilation (RV), TV of 10 ml/kg, and is the recommended approach for intensivists worldwide (2). However, it's not yet shown whether PV can affect hemodynamic adverse effects and impaired usage of oxygen in the brain of a septic patient.

**Objectives:** The purpose of this study is to examine brain oxygenation and hemodynamic changes in a sepsis porcine model and to investigate whether PV may affect cerebral usage and availability of oxygen, in comparison to RV.

**Methods:** Data was collected in an ongoing project concerning neuroinflammation in septic pigs, at the Hedenstierna Laboratory in Uppsala, Sweden.

Fourteen healthy pigs were randomized to two groups and anaesthetized. The groups were given an infusion of lipopolysaccharide to induce a septic like condition and was ventilated with RV (n = 6) respectively PV (n = 8). The animals received vascular catheters in order to draw arterial and venous blood gases before and after the blood has passed through the brain (sagittal sinus), and to monitor hemodynamic changes. A flow meter was applied to the left internal carotid artery. Hemodynamic data and blood samples were collected every hour for 6 hours.

**Results:** No difference in mean arterial pressure or arterial oxygen content was seen between the two groups. Protective ventilation enhanced oxygen delivery index to the brain (44.5 ± 14.1 to 30,3 ± 5.9 mlO2/min/m2, p = 0.018). Increased blood flow to the brain due to reduced brain vascular resistance was seen in the PV-group in comparison to the RV-group (19046.2 ± 7270.4 to 26637.3 ± 5678.5 dyn · s · cm − 5, p = 0.018).

**Conclusions:** Protective ventilation induces hemodynamic changes in cerebral blood vessels in the septic pig which reduces brain vascular resistance. These changes leads to improved oxygen delivery to the brain. Further studies will take place in order to clarify the cause behind these changes, with an emphasis on the role of inflammatory biomarkers and neuronal nitric oxide synthase.

**References** 1. Mouncey PR, Osborn TM, Power GS, Harrison DA, et al. Trial of early, goal-directed resuscitation for septic shock. N Engl J Med. 2015;372(14):1301–11.

2. Dellinger RP, Levy MM, Rhodes A, Annane D, et al. Surviving Sepsis Campaign: international guidelines for management of severe sepsis and septic shock, 2012. Intensive Care Med. 2013;39(2):165–228.

**Grant acknowledgement**

This research received no specific grant from any funding agency in the public, commercial, or not-for-profit sectors.

#### A681 Continuous negative abdominal pressure - augments PEEP, reduces lung injury

##### T. Yoshida^1,2,3^, D. Engelberts^1^, G. Otulakowski^1^, B. Katira^1,2,3^, M. Post^1^, N.D. Ferguson^3,4^, L. Brochard^3,5^, M.B.P. Amato^6^, B.P. Kavanagh^1,2,3^, PLUG Working group

###### ^1^Hospital for Sick Children, University of Toronto, Physiology and Experimental Medicine, Toronto, Canada; ^2^Hospital for Sick Children, University of Toronto, Departments of Critical Care Medicine and Anesthesia, Toronto, Canada; ^3^University of Toronto, Interdepartmental Division of Critical Care Medicine, Toronto, Canada; ^4^University Health Network and Mount Sinai Hospital, Division of Respirology, Department of Medicine, Toronto, Canada; ^5^University of Toronto Saint Michael's Hospital and Keenan Research Centre, Toronto, Canada; ^6^Heart Institute (Incor) Hospital das Clínicas da Faculdade de Medicina da Universidade de São Paulo, Laboratório de Pneumologia LIM-09, Disciplina de Pneumologia, São Paulo, Brazil

####### **Correspondence:** T. Yoshida - University of Toronto, Interdepartmental Division of Critical Care Medicine, Toronto, Canada

**Introduction:** In supine patients with ARDS, the lung usually partitions into dorsal atelectasis and ventral aeration. Conventional positive-pressure ventilation directs tidal volume (V_T_) preferentially to ventral aerated areas. Attempts to 'recruit' atelectasis with positive pressure often overinflate aerated regions. This occurs due to a large vertical gradient of pleural pressure (P_pl_) in ARDS. Prone positioning is the only effective means to lessen this P_pl_ gradient.

**Objectives:** To test 2 hypotheses - That Continuous Negative Abdominal Pressure (CNAP), by selectively recruiting basal atelectasis, would:

(1) improve lung function in a stable lung injury model;

(2) attenuate ventilator-induced lung injury in a model of progressive lung injury.

**Methods:** An established model of ventilator-induced lung injury (VILI) was used (anesthetized pig) and all animals were monitored using esophageal manometry (P_es_, pleural pressure), Electrical Impedance Tomography (EIT, regional ventilation) and pulmonary artery catheter. 2 series of experiments were performed.

***Series 1 - Stable Lung Injury***. 7 pigs were subject to maximal lung recruitment using PEEP (20 cmH_2_O) followed with progressive derecruitment by lowering PEEP in serial increments of 2 cmH_2_O; full physiologic assessment was performed at each decrement of PEEP. This series of assessments was performed twice in each animal: once with CNAP (−5 cmH_2_O) and once with no CNAP (the order was randomized).

***Series 2 - Progressive Lung Injury***. Animals were randomized to 'CNAP' or 'no CNAP' (5 per group) and both groups were exposed to standardized injurious ventilation: V_T_ 20 mL/kg, low PEEP, and the same expiratory transpulmonary pressure (P_L_, −3 cmH_2_O) for 4 h.

**Fig. 92 (abstract A681).**
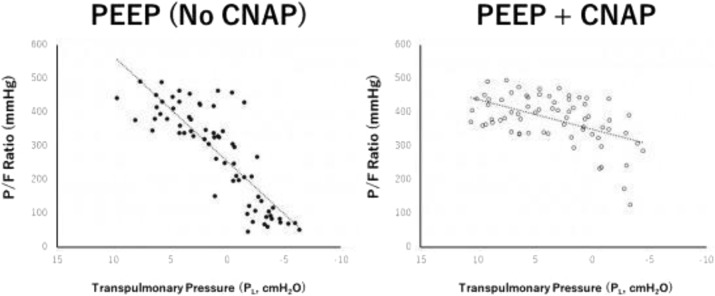
ᅟ

**Results:*Series 1 - Stable Lung Injury****.* CNAP considerably reduced the vertical gradient of P_pl_ (measured by direct instrumentation): at PEEP = 4: 11.3 ± 3.5 vs. 6.6 ± 2.5 cmH_2_O, PEEP vs. PEEP + CNAP (P < 0.01). Application of CNAP consistently resulted in better oxygenation, respiratory system compliance, and more homogeneous ventilation at descending PEEP values from 12 to 4 cmH_2_O. At PEEP = 4, the P/F ratio (mmHg) was 67 ± 5 (no CNAP) vs. 263 ± 36 (with CNAP, P < 0.01). At most levels of PEEP, oxygenation with added CNAP was greater despite a lower global P_L_ (Fig. 92).

. Dynamic CT demonstrated that addition of CNAP (−5 cmH2O) to a PEEP level of 10 cmH2O yielded the same amount of aeration as a PEEP of 18 cmH2O.

**Fig. 93 (abstract A681).**
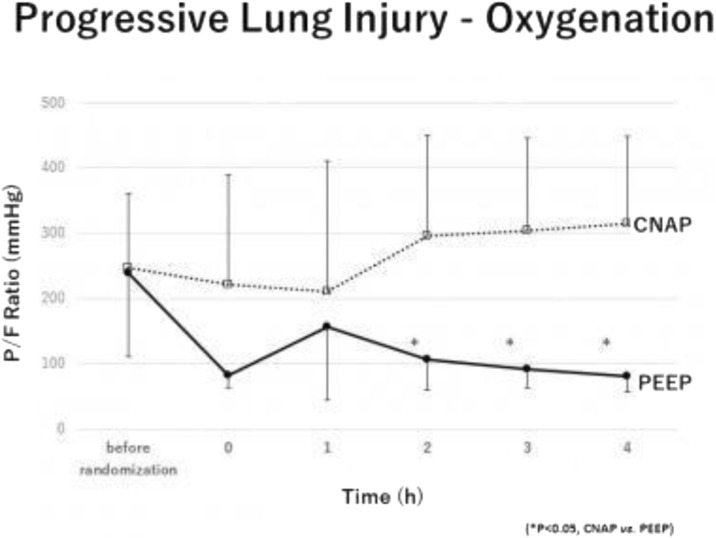
ᅟ

**Fig. 94 (abstract A681).**
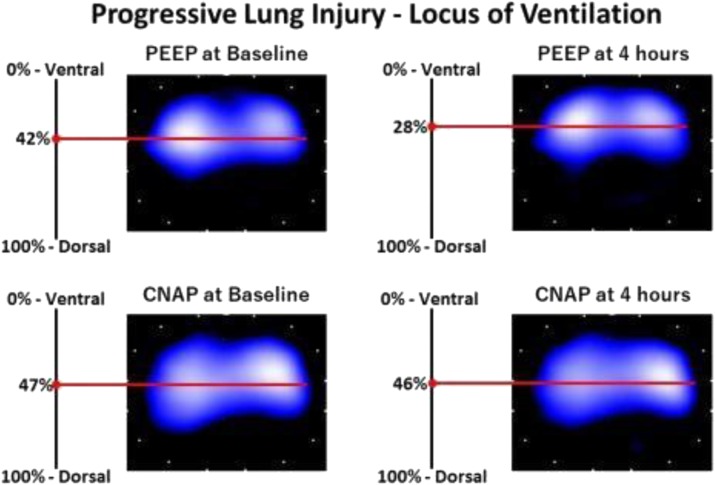
ᅟ

***Series 2 - Progressive Lung Injury***. Ventilation with no CNAP resulted in progressive lung injury; use of CNAP reduced the progression of lung injury resulting in greater oxygenation, respiratory system compliance, and homogeneity of ventilation (Figs. 93 and 94)

**Conclusion** CNAP selectively recruited basal atelectasis and homogenized ventilation by increasing regional P_L_ in dependent lung. CNAP is not only 'equal but opposite' to positive pressure; instead, at comparable P_L_ it improves lung function and lessens lung injury.

BPK, TY & DE - CNAP device patent applied

### Liver failure

#### A682 Racehorse: a randomized controlled trial on goal-directed therapy of hepatorenal syndrome

##### N. Koch, W. Huber, J. Hoellthaler, S. Mair, V. Phillip, R.M. Schmid, A. Beitz

###### ^1^Technische Universität München, II. Medizinische Klinik, Munich, Germany

####### **Correspondence:** W. Huber - Technische Universität München, II. Medizinische Klinik, Munich, Germany

**Introduction:** Hepatorenal syndrome (HRS) is a frequent complication of liver cirrhosis with poor prognosis. Pathophysiology of HRS comprises excessive splanchnic vasodilation, reduction of effective arterial volume and reduced renal perfusion. Several therapeutic approaches have been shown to improve prognosis of HRS, including volume expansion with albumin, terlipressin and transjugular porto-systemic shunt (TIPSS). While the specific haemodynamics of HRS and these therapeutic approaches have been thoroughly investigated using advanced haemodynamic monitoring, this technology is not yet recommended in recent guidelines.

**Objectives:** Therefore, it was the aim of our randomized controlled trial to compare the outcome of patients with HRS treated according to a pre-defined algorithm based on transpulmonary thermodilution (TPTD) to standard treatment.

**Methods:** 25 patients were randomized with a ratio of 1:2 to standard care or to the RACEHORSE-protocol (Early goal-directed volume resuscitation in hepato-renal syndrome). This protocol was based on three sequential algorithms including global end-diastolic volume index GEDVI, extravascular lung water index EVLWI, cell count in the ascites and pO2/FiO2. In summary these algorithms aimed at GEDVI-guided volume expansion within the first 48 h, followed by a TPTD-guided strategy for fluid support using the PiCCO-2-device (Pulsion Medical Systems SE, Feldkirchen, Germany). Primary endpoint: Combination of complete reversal of HRS according to the Ascites-Club-criteria (2007) within 14d AND survival without renal replacement therapy (RRT) AND without liver transplantation for ≥28d. Statistics: SPSS 23.

**Results:** 9 female, 16 male patients with alcoholic (22), viral (2) or autoimmune (1) aetiology of cirrhosis. Patients of treatment group (n = 16) and controls (n = 9) were comparable regarding baseline serum creatinine (3.6 ± 1.5 vs. 3.1 ± 1.3 mg/dL), BUN (66 ± 27vs. 54 ± 22 mg/dL), cystatin C (2.5 ± 0.8 vs. 2.5 ± 0.4 ng/dL), indocyanine green plasma disappearance rate (4.4 ± 1.2 vs. 5.5 ± 2.3 %/min), sodium, ADH, aldosterone, adrenalin and noradrenalin pevels (all p-values >0.05). The primary endpoint was met in 0/9 (0 %) of the controls and 6/15 (40 %; p = 0.028) of the treatment group. Several other endpoints gave hints for a better outcome in the treatment group compared to controls, although statistical significance was failed: Complete response within 14d without RRT (9/16 (56 % vs. 2/9 (22 %); p = 0.072), changes in serum creatinine after 7d (−1.0 ± 1.9 vs. -0.3 ± 1.7 mg/dl; n.s.), requirement of RRT (6/15 (40 %) vs. 5/9 (56 %); n.s.)and days without RRT within 28d (19 ± 11 vs. 15 ± 11; n.s.).

**Conclusions:** Goal-directed therapy based on TPTD-guided substitution of albumin and crystalloids improves short term outcome of patients with HRS. Due to the limited statistical power of this mono-centric study larger multi-centric trials also including targets for vasopressor therapy are required to confirm these data.

#### A683 Can we predict early liver failure with indocyanine green liver clearance?

##### V. Baladrón^1^, F.J. Redondo Calvo^2^, D. Padilla^3^, P. Villarejo^4^, R. Villazala^1^, A.S. Yuste^1^, N. Bejarano^5^

###### ^1^Hospital General Universitario de Ciudad Real, Anestesiologia y Reanimación, Ciudad Real, Spain; ^2^Facultad Medicina Ciudad Real, Hospital General Universitario de Ciudad Real, Anestesiologia y Reanimacion, Ciudad Real, Spain; ^3^Facultad de Medicina Ciudad Real, Hospital General Universitario de Ciudad Real, Ciudad Real, Spain; ^4^Facultad de Medicina Ciudad Real, Hospital General Universitario de Ciudad Real, Cirugía Hepatobiliar, Ciudad Real, Spain; ^5^Facultad de Medicina Ciudad Real, Hospital General Universitario de Ciudad Real, Cuidados Criticos Pediatricos, Ciudad Real, Spain

####### **Correspondence:** Facultad Medicina Ciudad Real, Hospital General Universitario de Ciudad Real, Anestesiologia y Reanimacion, Ciudad Real, Spain

**Introduction:** A non-invasive liver function monitoring system, the LIMON®, has been developed to measure indocyanine green (ICG) elimination by pulse spectrophotometry. The aim is to assess the relationship between pre and post-operative ICG plasma disappearance rate (ICG PDR %/min) values and the onset of post-hepatectomy liver dysfunction. Postoperative liver failure after hepatectomy has been identified by the association of prothrombin time < 50 % and serum bilirubin >50 umol/L (the “50-50” criteria).

**Methods:** We collected 30 patients scheduled for major liver resections. Nine had chronic liver disease. Prothrombin time and serum bilirubin was determined on day 3 and day 5 in critical care unit after hepatectomy. IGC PDR was measured preoperatively and 24 hours postoperative.

**Results:** We submit the values obtained for the variables of the ICG PDR pre and postoperative to an analysis of ROC curves, we found a very good predictive capacity for postoperative PDR showing an area under the curve of 0.96 but not well for preoperative PDR AUC = 0,667 for determined early diagnosis of postoperative liver failure. Bilirrubin and prothrombin time show an area under the curve of 0,922 and 1.

Significant correlation was found between ICG PDRpost measurement taken on postoperative day 1 and bilirubin level on day 5 (p < 0,001) and prothrombin time on day 5 (p,001).

The most sensible and specific value calculated for PDR post was 7.8.

**Discussion:** LiMON ICG PDR measured by pulse spectrophotometry is a non-invasive and reliable liver function test for patients undergoing liver resection that aids in the prediction and early detection of postoperative liver failure.

**References**

· Paugam-Burtz D, Janny S, Delefosse D, Dahmani S, Dondero F, Mantz J et al. Prospective validation of that “fifty-fifty” criteria as an early and accurate predictor of death after liver resection in intensive care unit patients. Ann Surg 2009;249:124–128

· Sugimoto H, Okochi O, Hirota M, Kanazumi N, Nomoto S, Inoue S et al. Early detection of liver failure after hepatectomy by indocyanine Green elimination rate measured by pulse dye-densitometry. J Hepatobiliary Pancreat Surg 2006;13: 543–548.

· Okochi O, Kaneko T, Hiroyuki S, Inoue S, Takeda S, Nakao A. ICG pulse spectrophotometry for perioperative liver function in hepatectomy. J Surg Res 2002;103:109–13.

**Grant acknowledgement**

We express our gratitude to Mutua Madrileña Fundation (Madrid, Spain) for its grant collaboration by without which this work could not have been completed

#### A684 Lactate and lactate clearance as early predictors of postoperative liver function after liver resection

##### R.J. Steenstra^1^, H. Banierink^2^, J. Hof^3^, I.C. van der Horst^2^, M.W. Nijsten^2^, M. Hoekstra^2^

###### ^1^University Medical Center Groningen, University of Groningen, Department of Anesthesiology, Groningen, Netherlands; ^2^University Medical Center Groningen, University of Groningen, Department of Intensive Care, Groningen, Netherlands; ^3^University Medical Center Groningen, University of Groningen, Department of Surgery, Groningen, Netherlands

####### **Correspondence:** R.J. Steenstra -University Medical Center Groningen, University of Groningen, Department of Anesthesiology, Groningen, Netherlands

**Introduction:** Post-operative liver failure (PLF) is a major complication after extended liver resection with a high mortality rate. Early identification of patients at risk for PLF could improve prognosis.

**Objectives:** To determine the time course of lactate, lactate clearance and the relationship of lactate with other markers of liver function and mortality.

**Methods:** All consecutive patients (2008–2014) who were postoperatively admitted to the intensive care unit (ICU) after liver resection were included. Patients were classified as “minor” or “major” liver resection based on a functional liver remnant (FLR) of ≥70 % and < 70 % respectively. Lactate clearance (%) was defined as: (lactate at ICU admission - lactate at 6 h) / lactate at ICU admission *100. The 50–50 criteria, a validated predictor of liver failure and death after liver resection, was defined as PT >21'' and serum bilirubin >50 umol/L on postoperative day 3 [1]. Numbers are expressed as median (interquartile range).

**Results:** A total of 382 patients admitted to the ICU after liver resections were included (80 % major resection). The main indication was colorectal liver metastasis (63 %). At ICU admission lactate was 2.4 (1.7-3.4) mmol/L. Maximum lactate level was 2.7 (2–4) mmol/L and occurred at 1.2 (0.3-7.9) hours after ICU admission. There was a significant difference between the lactate clearance at 6 h between the minor and major resections (25 % versus 14 %, P = 0.005). Highest PT was observed 30 hours after ICU admission. Only 3 patients met the “50-50 criteria” at day 3 and mortality at 30-days was 3.1 %. After multivariate analysis, lactate at ICU admission and perioperative blood loss, but not FLR, lactate clearance at 6 h or the “50-50 criteria”, were independent predictors of 30-day mortality.

**Conclusions:** Lactate levels at ICU admission, but not lactate clearance during the first 6 hours, is a strong independent predictor for 30-day mortality. In addition, lactate at ICU admission is of more prognostic value than the often used “50-50 criteria” (day 3) and also available much earlier.

**References**

[1] Paugam-Burtz C, Janny S, Delefosse D et al. Prospective validation of the “fifty-fifty” criteria as an early and accurate predictor of death after liver resection in intensive care unit patients. Ann Surg 2009; 249(1): 124–8.

**Grant acknowledgement**

None

#### A685 Incidence and outcome of hypoxic hepatitis in patients after out-of-hospital cardiac arrest

##### K. Roedl^1^, F. Sterz^2^, T. Horvatits^1^, K. Horvatits^1^, A. Drolz^1^, H. Herkner^2^, V. Fuhrmann^1^

###### ^1^Medical University Center Hamburg-Eppendorf, Department for Intensive Care Medicine, Hamburg, Germany; ^2^Medical University of Vienna, Department of Emergency Medicine, Vienna, Austria

####### **Correspondence:** K. Roedl - Medical University Center Hamburg-Eppendorf, Department for Intensive Care Medicine, Hamburg, Germany

**Introduction:** Sudden cardiac arrest (CA) is one of the leading causes of death in adults in many parts of the world. Every year estimated 350.000 to 700.000 people in Europe are suffering CA and receive cardiopulmonary resuscitation (CPR). Hypoxic hepatitis (HH) can be found in up to 10 % of critically ill patients. To date, data on incidence and outcome of HH after out-of-hospital CA and CPR is scarce.

**Objectives:** Aim of the study was to determine incidence and outcome of HH in patients after out-of-hospital CA.

**Methods:** Assessment of incidence of HH in a cohort of 798 consecutive patients with out-of-hospital CA and successful CPR that were treated at the Medical University Vienna, 168 (21 %) patients with HH could be identified. Patient characteristics, admission diagnosis, severity of disease, course of the disease and 28d mortality were assessed.

**Results:** Overall, 168 patients representing 21 % of the total cohort (128 male, age 56 (47–66) years) developed HH after CA with successful CPR were assessed. CA was witnessed in 136 (81 %) cases. Initial rhythm was shockable (VT/VF) in 90 (54 %), non-shockable (PEA/Asystole) in 75 (44 %) and unknown in 3 (2 %) patients. Time to ROSC was 27 (13,25 - 46,50) minutes. Cardiac events leading to CA were observed in 108 (64 %) of patients. SOFA-Score on admission was 11 (9–12), SAPS II was 80 (75–86).149 (89 %) patients needed mechanical ventilation on admission and 123 (73 %) needed vasopressor support. Mild therapeutic hypothermia was applied in 125 (74 %) patients. 28d-survival was 45 % in patients with HH and 60 % in the total cohort (p < 0,05). 107 (64 %) patients with HH were dead or had bad neurological outcome (CPC III/IV) after 28d.

**Conclusions:** New onset of HH is a frequent finding in patients following successful CPR out-of-hospital and is associated with worse outcome.

#### A686 Effect of acetaminophen on hepatocellular procalcitonin biosynthesis in a human hepatoma cell line (HepaRG©)

##### M. Kott^1^, K. Zitta^1^, B. Brandt^2^, C. Schildhauer^1^, G. Elke^1^, L. Hummitzsch^1^, I. Frerichs^1^, N. Weiler^1^, M. Albrecht^1^

###### ^1^UKSH, Campus Kiel, Department of Anaesthesiology and Intensive Care Medicine, Kiel, Germany; ^2^UKSH, Campus Kiel, Institute for Clinical Chemistry, Kiel, Germany

####### **Correspondence:** M. Kott - UKSH, Campus Kiel, Department of Anaesthesiology and Intensive Care Medicine, Kiel, Germany

**Introduction:** Acetaminophen (APAP) overdose is the most frequent cause of acute liver failure in many countries. Following cell necrosis, the release of intracellular damage-associated molecular patterns, like high-mobility group-box protein-1 or DNA fragments, induces a sterile inflammation mediated through interleukin- (IL) 1, IL-6 and tumor necrosis factor-α (TNF-α), amongst others^1^. Procalcitonin (PCT) that is frequently used to monitor the course of systemic infections and inflammation is also up-regulated through these pro-inflammatory mediators^2^. Therefore, PCT could be a potential biomarker to monitor the course of APAP overdose patients.

**Objectives:** This pilot study sought to assess whether APAP administration induced PCT synthesis in an in vitro model of human hepatoma cells (HepaRG©) and to explore possible dose-dependent relationships.

**Methods:** Human hepatoma cells (HepaRG©, Biopredic International, Rennes, France) were cultured according to the instructions of the manufacturer. Cells were exposed to increasing concentrations of APAP (20 μg/ml; 200 μg/ml, 500 μg/ml). Cell supernatants were collected after 24 hr (time/concentration: 24/20, 24/200, 24/500) and after 48 hr (48/20, 48/200, 48/500). To assess if HepaRG cells are capable of producing PCT, lipopolysaccharide stimulated cells were used as positive controls (24/LPS and 48/LPS). PCT was measured using the BRAHMS PCT LIA-Test (Thermo Fisher Scientific, Waltham, USA). Lactate dehydrogenase (LDH), gamma-lactate dehydrogenase (GLDH), aspartate aminotransferase (AST), and alanine aminotransferase (ALT) were measured as markers of hepatocellular injury. All analyses were carried out in duplicate.

**Results:** At both time points, no increase in PCT (median: 0.088 ng/ml, 25 %/75 % percentile: 0.0815 ng/ml / 0.097 ng/ml, P = > 0.05) was found when compared to control (0.082 ng/ml). LDH concentrations (with control as 100 %) were 24/20: 106 %, 24/200: 98 %, 24/500: 51 %, and 48/20: 98 %, 48/200: 98 %, 48/500: 51 %. GLDH was 24/20: 98 %, 24/200: 119 %, 24/500: 143 %, and 48/20: 98 %, 48/200: 98 %, 48/500: 51 %. AST was 24/20: 100 %, 24/200: 152 %, 24/500: 193 %, and 48/20: 104 %, 48/200: 144 %, 48/500: 185 %. ALT was 24/20: 95 %, 24/200: 111 %, 24/500: 157 %, and 48/20: 90 %, 48/200: 131 %, 48/500: 95 %. LPS administration did not result in a statistically significant increase either in PCT or the assessed liver markers when compared to control.

**Conclusions:** APAP and LPS administration did not result in a significant increase in PCT in this in vitro model of APAP toxicity. APAP concentrations might have been too low to induce enough cell necrosis and consecutive inflammation, although our experiments were based on APAP concentrations that are clinically relevant and found in APAP overdose patients.

**References**

1) Jaeschke H et al., Liver Int 2012

2) Matwiyoff GN et al., Inflamm Res 2012

#### A687 Usefulness of score "life" in the prognosis of patients with liver disease requiring icu admission

##### L. Rey González, D. Cabestrero Alonso, A. Blandino Ortiz, R. de Pablo Sánchez, J. Higuera Lucas

###### Hospital Ramón y Cajal, Madrid, Spain

####### **Correspondence:** A. Blandino Ortiz - Hospital Ramón y Cajal, Madrid, Spain

**Introduction:** Recently, it has been proposed "LiFe" score as a quick, easy and useful tool for evaluating patients with severe liver disease admitted at ICU area.

**Objectives:** To assess the validity and usefulness of the score "LiFe" in patients with liver disease requiring admission in Intensive Care Medicine of our environment.

**Methods:** Retrospective analysis of patients with liver disease admitted from May 2013 to October 2015. The following variables were analyzed: origin of liver disease, cause of decompensation, SOFA, APACHE II, SAPS II, MELD, lactate, bilirubin, INR (calculating "LiFe" score), mortality and mortality at 30 days.

**Results:** 113 patients, 73.5 % men and 26.5 % women, mean age 57.42 ± 10.21 (34–85) were included. The severity scores: APACHE II 21.18 ± 9.38 (5–50), SAPS 51.55 ± 19.7 (10–116), 10.28 ± 6.8 SOFA (1–24), MELD at admision18.82 ± 9.63 (6–47). The mean values of lactic acid, bilirubin and INR: lactate 3.88, bilirubin 4,90, INR 1.82. The score "LiFe" of the sample is 2.75 ± 2.62 (0–10), stratifying at: 20.4 % low risk, 45.1 % intermediate risk, 24.8 % high risk and 3.5 % very high risk . The mortality of patients in ICU: alive (71.7 %), dead (28.3 %); at 30 days: alive (56.6 %), dead (43.4 %).

Applying a Chi square test the score "LiFe" is significant in order to assess the mortality of patients both in ICU and at 30 days (p < 0.002 and p < 0.003 respectively).

The Pearson correlation coefficient between the different scores and the score "LiFe" is: APACHE II 0.51, SAPS II 0.41, SOFA 0.52 and MELD 0.74.

**Conclusions:** The score "LiFe" is a quick and useful tool in our environment for assessing the prognosis of patients with liver disease admitted to ICU. The score "LiFe" is correlated with MELD values.

**References**

1. Edmark C, et al. LiFe: a liver injury score to predict outcome in critically ill patients. *Intensive Care Med* (2016) 42:361–369 DOI 10.1007/s00134-015-4203-5.

#### A688 Hypoxic hepatitis after in-hospital cardiac arrest

##### K. Roedl^1^, F. Sterz^2^, A. Drolz^1^, K. Horvatits^1^, T. Horvatits^1^, H. Herkner^2^, V. Fuhrmann^1^

###### ^1^Medical University Center Hamburg-Eppendorf, Department for Intensive Care Medicine, Hamburg, Germany; ^2^Medical University of Vienna, Department of Emergency Medicine, Vienna, Austria

####### **Correspondence:** K. Roedl - Medical University Center Hamburg-Eppendorf, Department for Intensive Care Medicine, Hamburg, Germany

**Introduction:** Hypoxic hepatitis (HH) is a frequent found condition in critically ill patients and is associated with a high morbidity and mortality. Up to 10 % of critically ill patients suffer from HH during their stay at the ICU. Literature of HH after cardiac arrest is scarce and only available in out-of-hospital cardiac arrest (CA). To date no study evaluated the incidence and outcome of HH in patients after in-hospital CA.

**Objectives:** Aim of the study was to determine incidence and outcome of HH in patients after in-hospital CA.

**Methods:** Assessment of incidence of HH in a cohort of 270 consecutive patients with in-hospital CA and successful CPR that were treated at the Medical University Vienna. Patient characteristics, admission diagnosis, severity of disease, course of the disease and 28d mortality were assessed.

**Results:** Overall, 51 patients representing 19 % of the total cohort (33 male, age 67 (54,5 - 76,5) years) developed HH after CA with successful CPR. CA was witnessed in 48 (94 %) cases. Initial rhythm was shockable (VT/VF) in 16 (31 %), non-shockable (PEA/Asystole) in 35 (69 %). Time to ROSC was 8,5 (2–18,5) minutes. Cardiac events leading to CA were observed in 34 (67 %) of patients. SOFA-Score on admission was 11 (8–13,5), SAPS II was 88 (78,5 - 97). Mild therapeutic hypothermia was applied in 24 (47 %) patients. In 6 (12 %) of patients with HH was a cardiac re-arrest observed. 28d-survival was 37 % in patients who developed HH compared to 63 % in patients without HH (p < 0,05). 19 patients, who developed HH, survived longer than one month, of these 16 (84 %) had good neurological outcome (CPC I/II).

**Conclusions:** New onset of HH is a frequent finding in patients following successful CPR in-hospital and is associated with worse outcome.

#### A689 Circulating bile acids predicting hepatic decompensation and acute-on-chronic liver failure

##### T. Horvatits^1^, A. Drolz^1^, K. Roedl^1^, K. Rutter^1^, A. Ferlitsch^2^, G. Fauler^3^, M. Trauner^2^, V. Fuhrmann^1^

###### ^1^University Medical Center Hamburg-Eppendorf, Hamburg, Germany; ^2^Medical University of Vienna, Vienna, Austria; ^3^Medical University of Graz, Graz, Austria

####### **Correspondence:** T. Horvatits - University Medical Center Hamburg-Eppendorf, Hamburg, Germany

**Introduction:** Accumulation of serum bile acids (BAs) plays a central role in hepatic damage and disturbed BA signalling in liver disease. However, there is lack of data regarding the impact of serum BAs on clinical complications, acute decompensation (AD) and acute-on-chronic liver failure (ACLF).

**Objectives:** Aim of this study was to evaluate the impact of circulating serum BAs on clinical complications in patients with liver cirrhosis.

**Methods:** 143 patients with liver cirrhosis were included in this prospective observational study. Total serum BAs and individual BA composition were assessed in all patients on admission. BAs were analyzed via high performance liquid chromatography. Clinical complications such as AD, ACLF and transplant-free survival were recorded.

**Results:** Circulating total and individual BAs were significantly higher in patients with bacterial infection, AD and ACLF (p < 0.001). BAs furthermore correlated significantly with model of endstage liver disease (MELD) and hepatic venous pressure gradient (p < 0.001). Total serum BAs were identified as independent predictor of decompensation on admission (independently of sex, age and severity of liver disease assessed via MELD score) (OR 1.017, 95%CI: 1.01-1.025, p < 0.001) and furthermore predicted new onset of decompensation/ACLF during follow-up (OR1.025, 95%CI:1.012-1.038, p < 0.001).

Best cut-off predicting new onset of AD/ACLF and survival during course of time was total BAs **≥**36.9 μmol/l.

**Conclusions:** Circulating total and individual BAs are associated with AD and ACLF in patients with liver cirrhosis. Measurement of serum BAs could represent an additional marker for risk stratification in cirrhotic patients with respect to new onset of decompensation and ACLF.

#### A690 Long-term follow-up of liver transplant-patients with renal dysfunction pre transplantation

##### T. Horvatits, S. Pischke, L. Fischer, F. Thaiss, M. Koch, K. Bangert, V. Fuhrmann, S. Kluge, A.W. Lohse, B. Nashan, M. Sterneck

###### University Medical Center Hamburg-Eppendorf, Hamburg, Germany

####### **Correspondence:** T. Horvatits - University Medical Center Hamburg-Eppendorf, Hamburg, Germany

**Introduction:** Since 2011 there is the option of kidney-after-liver transplantation (KALT) for patients with renal insufficiency via receiving extra points in the kidney allocation system during a period of 90 to 360 days after liver-only transplantation (LT).

**Methods:** In this retrospective analysis we included patients listed for LT with KALT bonus at the University Medical Centre Hamburg-Eppendorf, during a period of 05/2011 - 05/2015.

**Results:** 78 patients (female: 38 %, age: median 59 yrs, IQR 51–65) were included in the study. Median length of stay at intensive care unit was 16 days (IQR 7–37).

28 patients (36 %) needed renal replacement therapy (RRT) pre-LT. Median serum creatinin of those without need for RRT was 2,5 mg/dl (IQR 1,3-3,8 mg/dl). Post-LT 46 patients (59 %) required RRT. Only 7 patients (9 %) remained with terminal dialysis-dependent kidney insufficiency - 4 due to hepatorenal syndrome, 3 due to preexisting chronic renal insufficiency of other causes.

2 patients (3 %) received a kidney transplant (KT) during the predetermined period of KALT bonus of 360 days. Another 5 patients remained with need for RRT after expiry of the KALT option. One of them underwent RT in the Eurotransplant-senior-program. 4 (57 %) of these 7 patients had already been on RRT pre-LT.

Overall mortality rate of those without necessity of RRT pre-LT was 36 %, and 39 % in those with requirement of RRT pre-LT.

In total 29 (37 %) of 78 patients died during follow-up. 38 % of these patients required RRT pre-LT, whereas 80 % needed RRT post-LT.

**Conclusions:** 9 % (7/78) of patients undergoing LT with an option for KALT remained long-lasting with need for RRT. Only 3 % (2/78) received a kidney graft during the predetermined period of KALT option, whereas 5 (6 %) patients remained with necessity for RRT and subsequent need for KT. In these 6 % of LT-patients KALT allocation system was not effective with respect to prioritize patients for early KT.

#### A691 Bilirubin removal with a new adsorbent system: in vitro and in vivo study

##### S. Faenza^1^, A. Siniscalchi^1^, E. Pierucci^1^, E. Mancini^2^, D. Ricci^2^, C. Gemelli^3^, A. Cuoghi^3^, S. Magnani^4^, M. Atti^4^

###### ^1^Teaching Hospital Policlinico S.Orsola-Malpighi, Department of Surgery, Intensive Care and Transplantation, Bologna, Italy; ^2^Teaching Hospital Policlinico S.Orsola-Malpighi, Nephrology, Dialysis, Hypertension, Bologna, Italy; ^3^Science and Technology Park for Medicine, Mirandola, Italy; ^4^Aferetica s.r.l, Bologna, Italy

####### **Correspondence:** S. Faenza - Teaching Hospital Policlinico S.Orsola-Malpighi, Department of Surgery, Intensive Care and Transplantation, Bologna, Italy

**Introduction:** A new sorbent (*Cytosorb, Cytosorbents*), able to adsorb hydrophobic molecules, could be a valid artificial support in many conditions of organ failure through the reduction of cytokines and other toxic molecules directly from blood. At present, extracorporeal systems for this purpose are based instead on plasma-adsorption.

**Objectives:** We designed an in vitro and in vivo study. The in vitro study evaluated the system's adsorption capacity of bilirubin and the ability to remove protein-bound solutes, while the in vivo study verified the congruence between in vitro and in vivo results and clinical outcomes.

**Methods:** The in vitro study included 3 tests. Two tests were carried out with equimolar solution of Albumin(Alb)-Bilirubin(Bil), containing unconjugated Bil, strongly Alb-bound[1], to verify removal of protein-bound solutes.Test 3, 24 h long, was performed with higher concentration of Bil and lower of Alb, to study the kinetics of adsorption. The in vivo study is currently enrolling patients with severe hepatic dysfunction with hyperbilirubinemia. Primary outcome is to evaluate in vivo Bil removal, whereas secondary outcomes are the hemodynamic stabilization, the reduction of inotropes, ithe mprovement of hepatic function and inflammatory status.

**Results:** The in vitro experiments showed the adsorption capacity of the system concerning Bil, also Alb-bound (Table 67), with a minimal Alb loss. We could not demonstrate any release of the adsorbed Bil. For the in vivo study, we enrolled until today, 3 patients (Table 68). Bil Mass Balance (MB) in vivo was higher than in vitro and showed the system's capacity of Bil removal, whereas removal rate (RR) is not significant for that purpose, being affected by many factors, first at all Bil production. In patient 3, Cytosorb was continuously used for 4 days and it would seemed to favour reduction of inflammation, help the recovery of hepatic function, and the hemodynamic stabilization, with inotropic support interrupted already after 2^nd^ treatment.

**Conclusions:** The in vitro study shows the effectiveness in removing Bil, also Alb-bound, for 24 h, without significant Alb loss. In vivo, Cytosorb is able to remove Bil in combination with cytokine removal, as reported in literature. Clinical effects suggest to be linked to the precocity of treatment and the adequate dose. Its use might help in restoring organs function. Cytosorb might represent a simple aid in organ dysfunctions, that may work alone or in combination with CRRT. Other experiences are ongoing to confirm these data.

**References**

[1] Weber et al. Biomacromolecules 2008, 9 1322–1328.

**Table 67 (abstract A691).** In vitro data

	1^st^	2^nd^	3^rd^
Bil. (mM)	0,4	0,8	0,8
Alb. (mM)	0,4	0,8	0,4
MB Bil. (g)	0,94	1,02	2,49

**Table 68 (abstract A691).** In vivo data

	Tot. h treatment	Bil. Conc t0 (mg/dl)	MB Bil. (g)	RR %	Other
1/Cirrhosis post-transplantation	28	45,1	2,25	36	Gram - Sepsis
2/Cirrhosis post-transplantation	24	36,7	6,00	0	Gram - Sepsis
3/Hyperbilirubinemia post-transplantation	96	53,06	20,88	64.1	Gram - Sepsis

#### A692 Mortality of cirrhotic patients admitted to a polyvalent ICU

##### F. Sotos, J. Cánovas, A. López, A. Burruezo, D. Torres

###### Morales Meseguer Hospital, Murcia, Spain

####### **Correspondence:** F. Sotos - Morales Meseguer Hospital, Murcia, Spain

**Introduction:** Due to the severity of cirrhotic patient, ICU admission remains a problem in terms of results.

**Objectives:** To analyze the clinical characteristics, complications and hospital and a year evolution of patients admitted with a diagnosis of cirrhosis in an Intensive Care Unit.

**Methods:** Retrospective observational study on a prospective database of 10 years duration. All patients admitted consecutively with primary or secondary diagnosis of cirrhosis. Sociodemographic, clinical and developmental variables are analyzed. The variables are expressed as means ± standard deviation, and absolute and relative frequencies. Comparisons between variables using the Student *t* test and Pearson Chi2. Multivariate analysis using logistic regression. Analysis of survival by Kaplan Meier curves and compared with the log rank test.

**Results:** 229 patients were analyzed, mean age 58.6 ± 13.1 years, and 78.6 % males. The most common etiologies were alcoholic in 149 patients (65.1 %) and hepatitis C virus in 36 patients (15.7 %). The most common reason for admission was upper gastrointestinal bleeding in 33.6 % followed by acute respiratory failure in 22.3 %. The most common cause of respiratory infection was 51 cases (22.3 %) and the most common cause of bleeding was esophageal varices in 65 cases (28.4 %). Do not intubation order was established in 33 cases (14.4 %). The SAPS II 49 ± 21 at admission, and the MELD index 21 ± 37.1 % presented 9. Child-Pugh C. The SOFA rate and maximum income was 7 ± 4 and 11 ± 4, respectively. Hospital and year mortality was 47.6 % and 60.7 % respectively. 137 patients required mechanical ventilation (59.8 %). By multivariate analysis, predictors of hospital mortality were SAPS II (OR = 1.065; 95 % CI = 1.039 to 1.091; p < 0.001), MELD (OR = 1.083; 95 % CI = 1.032 to 1.137; p = 0.001) and maximum SOFA (OR = 1.287; 95 % CI = 1.143 to 1.448; p < 0.001). After one year of admission, the Child-Pugh classification was clearly related to survival (p < 0.001).

**Conclusions:** Cirrhotic patient admitted to ICU has a high mortality. The severity of the patient´s disease and the development of multiple organ failure are the main determinants of hospital evolution.

#### A693 Indocyanine green for the initial evaluation of graft function after liver trasplant

##### M.E. Herrera-Gutierrez, J. Barrueco-Francioni, D. Arias-Verdú, R. Lozano-Saez, G. Quesada-Garcia, G. Seller-Pérez

###### Complejo Universitario Carlos Haya, Málaga, Spain

####### **Correspondence:** D. Arias-Verdú - Complejo Universitario Carlos Haya, Málaga, Spain

**Introduction:** Evaluation of grafty function after liver trasplant (OLT) is a dificult task because diferent parameters must be taken into acount. Determination of clearance of indocyanine green (ICG) has been proposed as a good method to measure liver function in diferent scenarios.

**Objective:** To evaluate the capability of ICG elimination as a marker of development of graft dysfunction, kidney dysfunction or mortality.

Material: Protective observational study. Series of cases. In our patients we measured PDR and R-15 after a dose of 0,5 mgr/Kg IV of ICG by means of the Limon monitor (Pulsion®) the first day after OLT.

We looked the relationship with AKIN-III, graft dysfunction (modified Toronto criteria) and in-hospital mortality. Analysis was performed by chi-square, Student *t*-test and area under curve (AuC).

**Results:** 39 patients, age 55.7 ± 11.3 years, 66,7%men. Mean hospital stay was 18.8 ± 16 days. APACHE II at admission 13.8 ± 3.6, SOFA 7.1 ± 3.4, lactate 1.8 ± 1.2 mm/L and PDR 19.5 ± 14.5 %.

In 15.4%patients a mild dysfunction was detected, without associated mortality and 20.5 % developed severe dysfunction with a mortality of 37.55. In 15,4 % patients AKIN-3 was detected with a mortality of 7.7 %.

None of the variables analyzed showed a good relationship with severe liver dysfunction [SOFA AuC 0.69(IC 0.52-0.86, ns) and ICG 0.58(0.40-0.76, ns)]. For AKIN-3, SOFA predicted its development with an AuC 0.92(0.83-1, p 0,001) vs PDR 0.65(0.48-0.82, ns). Regarding mortality, SOFA AuC was 0.92(0.79-1, p 0.01), lactate 0.85(0.70-0.88, p0.048) but ICG only 0.64(0.39-0.88, ns).

**Conclusions:** In our series, SOFA at admission and lactate levels seem the best predictors for outcome, clearly surpassing ICG clearance.

**Fig. 95 (abstract A693).**
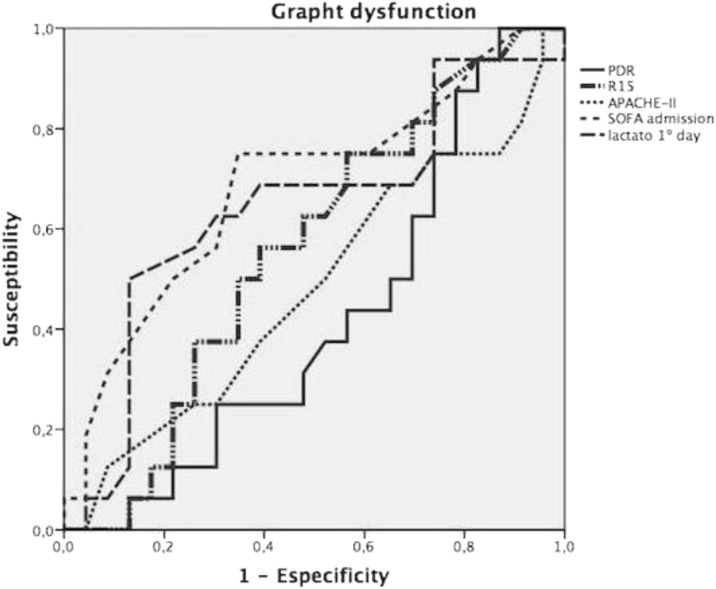
ᅟ

#### A694 Plasmatic ionised calcium concentrations associated with worse outcomes in post-surgical ICU liver transplant patients

##### A. Figueiredo, Y. Anzola, R. Pereira, L. Bento

###### Hospital Curry Cabral, CHLC, UCIP7, Lisboa, Portugal

####### **Correspondence:** R. Pereira - Hospital Curry Cabral, CHLC, UCIP7, Lisboa, Portugal

**Introduction:** Plasmatic ionised calcium is vital for organic function and homeostasis of the human body.

Plasmatic ionised calcium concentration (PICC) is afected by multiple clinical conditions such as endocrinopathies, organ disfunction, pH and albumin concentration.

In the critically ill patient adequate PICC is even more important in the presence of neuro-muscular or cardio-vascular disfunction, coagulopathy, bleeding and massive transfusion.

In adult critically ill patients calcium supplementation has been reported to improve 28-day survival [1]. Conversely, it has been shown that in septic patients calcium administration increases inflamatory signaling, multiorgan dysfunction and mortality [2].

The critical post liver transplant patient admitted in ICU can present with these conditions leading to altered PICC.

**Objectives:** We analysed PICC of liver transplant patients and its association with ICU outcomes.

**Methods:** Retrospective single-center study in liver transplant patients admitted in ICU at a reference liver transplant center.

PICC was analysed at admission and its trend in percentual diference from baseline (deltaPICC) reflected the initial 12 hours evolution period. Univariable analysis was performed to explore association with ICU lenght of stay (ILOS) and UCI vital outcome.

**Results:** 140 cases were included in this study.

In the ICU survivor group (n = 166) the mean PICC was 1.11 mmol/L (SD 0.12) and deltaPICC −3.04 % (SD 15.2 %). While in non-survivor the mean PICC (n = 9) was 1.17 mmol/L (SD 0.09) and deltaPICC (n = 7) was −16.1 % (SD 10.1 %). The diference between survivor and non-survivor was non significative for PICC (p = 0.15; IC95% 0.021-0.14) and significative for deltaPICC (p = 0.03; IC95% -24.5; −1.49).

In the 3 or less days ILOS group (n = 60) the mean PICC was 1.10 mmol/L (SD 0.09); in the 4 or more days ILOS group (n = 67) the PICC was 1.15 mmol/L (SD 0.15). There was a significant PICC difference between ILOS groups (p = 0.03; IC95% 0.003-0.09). Differences between deltaPICC between 3 or less days ILOS group (mean −1.15 %, SD 14.4 %) and 4 or more days ILOS (mean −1.16 %, SD 10.0 %) were not significative (p = 0.99; 0.-4.31; 4.33).

**Conclusions:** Univariable analysis in liver transplant post-surgical patients revealed longer ILOS significantly associated with higher PICC at ICU admission and a negative deltaPICC significantly associated with higher ICU mortality.

Multivariate analysis will assess the independent value of calcemia as a risk factor in this ICU population.

**References**

1. Zhang Z et al. Calcium supplementation improves clinical outcome in intensive care unit patients: a propensity score matched analysis of a large clinical database MIMIC-II. Springerplus. 2015

2. Collage RD et al. Calcium supplementation during sepsis exacerbates organ failure and mortality via calcium/calmodulin-dependent protein kinase kinase signaling. Crit Care Med. 2013

#### A695 Changes in the early postoperative morbidity after orthotropic liver transplant in recent years

##### D. Arias-Verdú^1^, M. Lai^2^, M. Deiana^2^, J. Barrueco-Francioni^1^, M.E. Herrera-Gutierrez^1^, G. Seller-Perez^1^

###### ^1^Complejo Universitario Carlos Haya, Málaga, Spain; ^2^University of Perugia, Department of Clinical and Experimental Medicine, Perugia, Italy

####### **Correspondence:** D. Arias-Verdú - Complejo Universitario Carlos Haya, Málaga, Spain

**Introduction:** Liver transplant (OLT) is the only definitive therapy for patients with cirrhosis and also for those patients on acute liver failure that do not regain liver function. It is a highly demanding procedure that carries a high number of complications, but outcome is regarded nowadays as excellent. We analyzed prospectively ten years ago the complications in our patients and wanted to evaluate if management and results have changes in this ten years.

**Objective:** To define the profile of early complications after OLT and its related variables and to compare them with our population from ten years ago.

Material y methods: Observational prospective cohorts recruiting all patients admitted to our unit after OLT during 2013–2015 (13–15) and retrospective comparison to a prospective cohort recruited from 2003–2005 (03–05). The transplantation team has not changed but a revision of the protocol had done during this interval (restriction in transfusion and volume infusion, immunosuppression based on CysA and TAC and use of basiliximab for patients with high risk for AKI). Definition of complications was the same but for AKI that was recalculated in the group 03–05 according to AKIN criteria. Fir the analysis we performed chi-square y Student t-test.

**Results:** Group 13–15, 180 patients; group 03–05, 131 patients. Equal gender, age and SOFA at admission but higher Child-C (28.9 % vs 13 %, p 0.004), higher percentage of arterial hypertension (32 vs 9.2, P 0,001) and previous renal disease (14.4 vs 6.1, p 0.032) for 13–15.

Transfusion during surgery was significantly lower for 13–15 as well as volume infused (11.34 L vs 5.9 L for the first three postoperative days, p 0.054).

We detected less reperfusion syndrome (19.4 vs 8.9 %, p 0.01) and coagulopathy (23.4 vs 9.4 %, p 0.001) during surgery for 13–15, an also a lower percentage of hypothermia (61.8 vs 17.2, p 0.0001), pulmonary edema (4.6 vs 0.6, p 0.04) or ARDS (8.4 vs 2.2, p 0.015) during the early postoperative course. Contrariwise, hyperglycemia was detected more frequently (78.5 vs 91.1 %, p 0.03).

No changes were detected in AKI, need for RRT, encephalopathy, hemorrhages, infections or primary graph dysfunction. Mortality was 11.5 % for 03–05 vs 6 % for 13–15 (p ns).

**Conclusion** Patients that opt for an OLT are sicker than before but this fact does not suppose a higher morbidity and we even detect a trend toward lower mortality. Changes in their management has supposed a lessening of intraoperative and respiratory complications.

### Sepsis biomarkers

#### A696 Glucocorticoid receptor expression is higher in patients with sepsis and non-survivors and is related to cortisol heat shock proteins and cytokines

##### K. Vardas^1^, S. Ilia^2^, A. Sertedaki^3^, E. Charmadari^3^, C.A. Stratakis^4^, E. Briassouli^5^, D. Goukos^5^, K. Psarra^6^, E. Botoula^7^, S. Tsagarakis^7^, E. Mageira^1^, C. Routsi^1^, S. Nanas^1^, G. Briassoulis^2^

###### ^1^National and Kapodistrian University of Athens, First Critical Care Department, Athens, Greece; ^2^University Hospital, University of Crete, Pediatric Intensive Care Unit, Heraklion, Greece; ^3^National and Kapodistrian University of Athens, Division of Endocrinology, Metabolism and Diabetes, First Department of Pediatrics, Athens, Greece; ^4^Eunice Kennedy Shriver National Institute of Child Health and Human Development (NICHD), National Institutes of Health, Section on Endocrinology and Genetics, Bethesda, USA; ^5^National and Kapodistrian University of Athens, 1st Department of Internal-Medicine - Propaedeutic, Athens, Greece; ^6^Evangelismos Hospital, Immunology-Histocompatibility Department, Athens, Greece; ^7^Evangelismos Hospital, Department of Endocrinology - Diabetes, Athens, Greece

####### **Correspondence:** S. Ilia - University Hospital, University of Crete, Pediatric Intensive Care Unit, Heraklion, Greece

**Introduction:** The glucocorticoid receptor (GR) is ubiquitously expressed on nearly all cell types, but tissue-specific deletion of this receptor can produce dramatic whole organism phenotypes. Recent data provide evidence that GR expression is progressively decreased in experimental sepsis and that dexamethasone has a decreased ability to translocate into the cell nucleus^1^.

**Objectives:** To evaluate the protein expression of the GR in peripheral blood monocytes (m) and neutrophils (n) and the GR isoform a (GRa) mRNA in whole blood in Intensive Care Unit (ICU) patients with early-onset severe sepsis (SS) or systemic inflammatory response syndrome (SIRS) compared to healthy control subjects (H). To relate their expression with serum levels of prolactin, cortisol and interleukins, plasma levels of ACTH, and to the expression of intracellular heat shock proteins 72 (HSP72) and HSP90α.

**Methods:** Consecutively admitted patients with early (first 48 hours) SS (n = 48) or SIRS (n = 40) were admitted to a University Hospital ICU and enrolled in the study. 35 H were included. Total RNA was isolated from peripheral blood samples and cDNA was prepared. RT-PCR was performed using Light Cycler 480 Probes Master kit, employing primers and Taqman probes specifically designed for NR3C1 (GRα) and RPLP0 (used as control gene), on Light Cycler 480 System (Roche). Monocytes and neutrophils protein expression was evaluated by flow cytometry. Mean Fluorescence Intensity (MFI) values for HSPs and GR were measured after staining surface antigens CD33 and CD45 with CD33-PE/Cy5 and CD45-PE/Cy7 followed by intracellular staining with HSP72-FITC, HSP90a-PE or GR-FITC using 4-colour flow cytometry. Prolactin and cortisol were measured using the ADVIA centaur system, ACTH using Immulite 2000. ELISA was used to evaluate interleukins.

**Results:** GR MFI was significantly higher in SS compared to SIRS and H (p < 0.01) and was related to mHSP90α (p < 0.0001), nHSP90α (p < 0.0001), mHSP72 (p < 0.02), cortisol (p < 0.03), and IL-6 (p < 0.005). ACTH, cortisol, prolactin, INFγ, IL-6, and IL-10 were also increased in SS compared to SIRS and/or H. ACTH and expression levels of NR3C1 (GRα) were significantly higher in non-survivors compared to survivors (p < 0.02) and were related to ACTH (p < 0.0001), INFγ (p < 0.002), and IL-10 (p < 0.04).

**Conclusions:** Sepsis and mortality are associated with early-onset increased GRa expression, which, apart from cortisol and ACTH, is also related to HSP90α, HSP72 expression, as well as to cytokines.

**References**

(1) Bergquist M, Nurkkala M, Rylander C, et al.Expression of the glucocorticoid receptor is decreased in experimental Staphylococcus aureus sepsis.J Infect. 2013;67(6):574–83.

**Grant acknowledgement**

This research has been co-financed by the European Union (European Social Fund (ESF)) and Greek national funds through the Operational Program "Education and Lifelong Learning" of the National Strategic Reference Framework (NSRF)-Research Funding Program: THALES.

#### A697 Microparticles and septic shock: new biomarkers of organ failure and AKI

##### A. Boscolo^1^, D. Bertini^1^, E. Campello^2^, V. Lucchetta^1^, E. Piasentini^1^, C.M. Radu^2^, L. Manesso^1^, P. Simioni^2^, C. Ori^3^

###### ^1^UOC Anesthesia and Intensive Care Unit, Hospital of Padova, Padova, Italy; ^2^Thrombotic and Hemorrhagic Diseases Unit, Department of Medicine, University of Padua, Padova, Italy; ^3^UO Anesthesia and Intensive Care Unit, Department of Medicine-DIMED, Padova, Italy

####### **Correspondence:** A. Boscolo - UOC Anesthesia and Intensive Care Unit, Hospital of Padova, Padova, Italy

**Introduction:** Microparticles (MPs) are cell membrane-derived particles promoting coagulation, inflammation and cell-to-cell communication. MPs have been assessed as possible biomarkers of septic vascular dysfunction and sepsis-induced AKI [1]. However, the role of MPs in the crosstalk between coagulopathy and inflammation in septic patients is still unclear.

**Objectives:** The aim of this case–control study was to clarify the possible role of MPs as biomarkers in Septic Shock (SS) measuring Annexin V+ and endothelial-derived MPs in consecutive patients.

**Methods:** 21 patients (43 % M, 57 % F, 66 y) admitted to our ICU with a diagnosis of SS were enrolled. Exclusion criteria were: end-stage heart failure and liver disease, severe active cancer, age < 18 years, pre-existent hematological disorders, ongoing anticoagulation and antiplatelet therapy.

21 healthy volunteers, age and sex matched, were recruited to establish references values for MPs.

Circulating MPs levels were measured at baseline (day 0) and 24 hours later (day 1) by size and Annexin V- FITC labelling, using flow-cytometer. Endothelial MP were identified using CD62E-PE monoclonal antibody [2]. The SOFA-score was determined over the first 24 hours and 28-day mortality was recorded. Data were expressed as medians, IQR or percentages. Correlations were assessed using Spearmann ρ. *T*-test was used for comparison between groups.

**Results:** In critically ill patients V^+^ Annexin and E selectin^+^ MPs were significantly higher than controls [4367 (IQR 2546–7419) vs 1728 (IQR 782.5-2122) MPs/ul at day 0; 367 (IQR 271–450) vs 97 (IQR 81–127.5) MPs/ul at day 1, p < 0.01]. Spearmann's correlation between SOFA-scores (11, IQR 8–14) and MPs revealed a moderate coefficient only with E selectin^+^ MPs (r = 0.43, p < 0.038). No differences were noticed based on outcome. Furthermore, 11 patients had a diagnosis of sepsis-induced-AKI [18 % with stage 1–2 and 82 % with stage 2–3,[1]) and once again their E selectin^+^ MPs were higher than non-AKI septic patinets [442 (IQR 409–604) vs 275 (IQR 274–352) MPs/ul, p < 0.03] at day 0.

**Conclusion** Our preliminary data showed that endothelial MPs and V Annexin^+^ MPs could be useful biomarkers of vascular damage. E selectin^+^ MPs were strongly correlated to disease severity and renal failure. Sepsis-induced AKI probably occurs in the setting of a more severe microvascular dysfuction. Our results confirmed an important endothelial alteration, probably due to an abnormal release of E selectin^+^ MPs, during sepsis-induced AKI. In conclusion, MPs may be important therapeutic targets to prevent or treat SS.

**References**

1. Uchino S, Kellum JA, Bellomo R, Doig GS, Morimatsu H,Morgera S, et al. Acute renal failure in critically ill patients: a multinational, multicenter study. JAMA '05;294:813e8.

2. Campello E, Spiezia L, Radu CM, et al. Circulating microparticles and the risk of thrombosis in inherited deficiencies of antithrombin, protein C and protein S. Thromb Haemost. 2015;115(1):81–8.

#### A698 Impact of initial lactate level on ed management of patients with sepsis: a multicenter retrospective cohort study

##### H. Su^1^, Y.M. Lam^1^, K. Willis^2^, V. Pullar^2^, R.P. Hubner^3^, J.L. Tsang^1,2,4^

###### ^1^McMaster University, Michael G. DeGroote School of Medicine, St. Catharines, Canada; ^2^Niagara Health System, St. Catharines, Canada; ^3^University of St. Andrews, Bute School of Medicine, St. Andrews, UK; ^4^McMaster University, Department of Medicine, Hamilton, Canada

####### **Correspondence:** H. Su - McMaster University, Michael G. DeGroote School of Medicine, St. Catharines, Canada

**Introduction:** Sepsis is “a life-threatening organ dysfunction caused by a dysregulated host response to infection.” Septic shock is “a subset of sepsis in which underlying circulatory and cellular/metabolic abnormalities are profound enough to substantially increase mortality.” (1) Lactate is a marker of organ hypoperfusion, thus organ dysfunction, and has been shown to be predictive of mortality (2, 3) and is a part of the new Sepsis-3 definition of septic shock.

**Objectives:** To examine the role of initial lactate level in the management of sepsis in the emergency department (ED).

**Methods**

**Study design:** A multi-centre retrospective cohort study

**Patient population:** All inpatients with sepsis, severe sepsis, or septic shock with initial lactate level drawn in the ED from July 2011 to July 2015.

**Study centres:** ED of three community acute care centres in the Niagara Region, Ontario, Canada.

**Data collection:** A dedicated Regional Sepsis Coordinator collected data on demographics, comorbidities, clinical symptoms, investigations and managements. Institutional research ethics board approval was obtained.

**Results:** A total of 2082 patients were included in the study. The median age was 72 years (interquartile range, 60–81), and the mean initial lactate level was 3.48 ± 2.63 mmol/L. Door-to-antibiotics time decreased from 194 ± 156 min in the normal lactate group to 138 ± 135 min in the high lactate group (p < 0.05). Total fluid administration in first 6 hours increased from 1.9 ± 1.3 L in the normal lactate group to 3.2 ± 1.9 L in the high lactate group (p < 0.05). Central line insertion rates increased from 8.8 % in normal lactate group to 47.8 % in the high lactate group (p < 0.05). Intensive Care Unit (ICU) admission rate increased from 25.4 % in normal lactate group to 54.5 % in high lactate group (p < 0.05). Unadjusted in-hospital mortality for normal, low, intermediate and high lactate group was 13.9 %, 21.7 %, 32.9 % and 57.2 %, respectively.

**Conclusions:** Higher initial lactate level correlates with more intensive ED management of sepsis including earlier antibiotics administration, more fluid resuscitation, increase chance of central line insertion and ICU admission. It also correlates with higher unadjusted in-hospital mortality rate.

**References**

1. Seymour CW, Liu VX, Iwashyna TJ, et al. Assessment of Clinical Criteria for Sepsis: For the Third International Consensus Definitions for Sepsis and Septic Shock (Sepsis-3). *JAMA.* 2016;315(8):762–774.

2. Mikkelsen ME, Miltiades AN, Gaieski DF, Goyal M, Fuchs BD, Shah CV, et al. Serum lactate is associated with mortality in severe sepsis independent of organ failure and shock. Crit Care Med 2009;37:1670–7.

3. Shapiro NI, Howell MD, Talmor D, Nathanson LA, Lisbon A, Wolfe RE, et al. Serum lactate as a predictor of mortality in emergency department patients with infection. Ann Emerg Med 2005;45:524–8.

**Grant acknowledgement**

Dr. J.L.Tsang is supported by the McMaster Internal Medicine Research Award

**Table 69 (abstract A698).** Summary of results

Lactate (mmol/L)	Normal (0–2.49)	Low (2.5-3.99)	Intermediate (4–5.99)	High (≥6)
Number of patients (total N=2082)	960 (46.1%)	508 (24.4%)	322 (15.5%)	292 (14.0%)
Time to antibiotics administration, mean in min ± SD	192 ± 157	158 ± 129	144 ± 124	138 ± 137
Amount of fluids given in first 6 hrs, mean in mL ± SD	1934 ± 1264	2304 ± 1306	2786 ± 1547	3209 ± 1939
Number of subsequent lactates drawn, mean ± SD	1.13 ± 2.38	2.45 ± 3.34	3.39 ± 3.84	3.98 ± 4.83
Mean time to repeat lactate, mean in min ± SD	586 ± 1482	844 ± 2555	605 ± 951	560 ± 1464
Central line insertion rates	8.8%	16.3%	25.8%	47.8%
ICU admission rates	25.4%	35.2%	47.2%	54.5%
Unadjusted in-hospital mortality	13.9%	21.7%	32.9%	57.2%

#### A699 Value of presepsin (SCD14-ST) as a diagnostic biomarker of sepsis in an emergency department. Comparison with procalcitonin and c-reactive protein

##### L. García de Guadiana-Romualdo^1^, S. Rebollo-Acebes^2^, P. Esteban-Torrella^1^, R. Jiménez-Sánchez^2^, E. Jiménez-Santos^1^, A. Ortín-Freire^2^, A. Hernando-Holgado^1^, M.D. Albaladejo-Otón^1^

###### ^1^Hospital Universitario Santa Lucía, Biochemistry Department, Cartagena, Spain; ^2^Hospital Universitario Santa Lucía, Critical Care Unit, Cartagena, Spain

####### **Correspondence:** L. García de Guadiana-Romualdo - Hospital Universitario Santa Lucía, Biochemistry Department, Cartagena, Spain

**Introduction:** Sepsis is a usual condition handled in the emergency department (ED) and, despite advances in antibiotic therapy and cardiovascular and respiratory support, represent a major cause of morbidity and mortality. Presepsin is an emergent early biomarker for sepsis.

Objective: To evaluate its diagnostic accuracy, compared to other traditional biomarkers, such as procalcitonin and C-reactive protein, in a population presenting to emergency department (ED) with suspected sepsis.

**Methods:***Study population*: 223 patients (median age: 69 years [(Interquartile range (IQR: 31)]; 131 (58.7 %) male) with suspicion of sepsis, according to 2001 SCCM/ESICM/ACCP/ATS/SIS International Sepsis Definitions Conference criteria presenting at the ED were recruited. Definitive diagnosis was made according to the Third International Consensus Definitions for Sepsis and Septic Shock (Sepsis-3).

*Laboratory methods*: Blood samples were collected at first medical evaluation and analyzed for sepsis biomarkers. Presepsin concentrations were measured by a chemiluminescent enzyme immunoassay on an automated immunoassay analyser (PATHFAST).

*Statistical analysis*: Receiver operating characteristic (ROC) curves and areas under the curves (AUCs) were calculated to evaluate the diagnostic performance of each biomarker and optimal cutoff values were derived from ROC curves. Values p < 0.05 were considered statistically significant. Statistical analysis was done using SPSS v. 20.0 and and MedCalc statistical software v.14.8.1.

**Results:** Sepsis was diagnosed in 70 (31.4 %) patients. According to the sepsis severity, septic patients were classified as sepsis [n = 57] and septic shock [n = 13]. C-reactive protein, procalcitonin and presepsin levels were higher in septic patients than in patients without sepsis [17.0 (21.3) mg/dL *vs.* 13.0 (14.1) mg/dL, p = 0.005; 4.05 (16.42) ng/mL *vs.* 0.29 (0.85) ng/mL, p < 0.001; 1115 (1723) pg/mL *vs.* 542 (374) pg/mL, p < 0.001, respectively]. The AUC ROC curves of procalcitonin (0.84 [CI95%: 0.78-0.89]) and presepsin (0.79 [CI95%: 0.73-0.84]) were similar and significantly greater than that of C-reactive protein (0.830 [95 % CI 0.55-0.68]). The corresponding procalcitonin and presepsin thresholds were 1.56 ng/mL (sensitivity 67 % and specificity 86 %) and 849 pg/mL (sensitivity 67 % and specificity 83 %).

**Conclusion** Presepsin was a valuable biomarker for diagnosis of sepsis in an ED, with a similar diagnostic accuracy than procalcitonin. Further research is needed to clarify the role of biomarkers after than sepsis definition has been updated.

**References**

1. Singer M, Deutschman CS, Seymour CW, Shankar-Hari M, Annane D, Bauer M, et al. The Third International Consensus Definitions for Sepsis and Septic Shock (Sepsis-3). JAMA 2016;315:801–10.

2. Pizzolato E, Ulla M, Galluzzo C, Lucchiari M, Manneta T, Lupia E, et al. Role of presepsin for the evaluation of sepsis in the emergency department. Clin Chem Lab Med 2014;5:1695–700.

#### A700 Value of C-reactive protein and procalcitonin in the discrimination between ventilator-associated tracheobronchitis and ventilator-associated pneumonia

##### L. Coelho^1^, L. Rabello^2^, J. Salluh^2^, I. Martin-Loeches^3^, A. Rodriguez^4^, S. Nseir^5^, P. Póvoa^1^, TAVeM study Group

###### ^1^Hospital São Francisco Xavier, Unidade de Cuidados Intensivos Polivalente, Lisboa, Portugal; ^2^D'Or Institute for Research and Education, Rio de Janeiro, Brazil; ^3^St James's Hospital, Department of Clinical Medicine, Dublin, Ireland; ^4^Hospital Joan XXIII, Tarragona, Spain; ^5^Centre Hospitalier Régional Universitaire de Lille, Lille, France

####### **Correspondence:** L. Coelho - Hospital São Francisco Xavier, Unidade de Cuidados Intensivos Polivalente, Lisboa, Portugal

**Introduction:** Ventilator-associated tracheobronchitis (VAT) has been suggested as an intermediate process between tracheobronchial colonization and ventilator-associated pneumonia (VAP) in patients receiving mechanical ventilation.

**Objectives:** The aim of this study was to evaluate the ability of C-reactive protein (CRP) and procalcitonin (PCT) to differentiate between VAT and VAP on the day of infection diagnosis.

**Methods:** Post-hoc analysis of the large prospective multinational TAVeM database, coming from 114 ICUs in Europe and Latin America (1). All consecutive patients receiving mechanical ventilation for >48 h were included in the TAVeM database. We compared VAT and VAP at the day of infection diagnosis. Only first episodes of VAT and VAP were taken into account.

**Results:** Four hundred and four patients (mean age 60 years, 238 men, ICU mortality 35 %) were studied, 207 with VAT and 197 with VAP. On the day of infection diagnosis, the median CRP was elevated in both groups but significantly higher in VAP (14 mg/dl and 18 mg/dl, p = .001). PCT was also significantly higher in VAP (0.64 ng/dl and 2.1 ng/dl, p < .001). However, roughly 25 % VAT patients and 16 % of VAP patients had PCT values < 0.25 ng/dl, while 14 % VAT patients and 7 % of VAP patients had CRP values < 4.0 mg/dl. Dividing the patients in sepsis, severe sepsis and septic shock, the levels of PCT were significantly different between the three groups (p = 0.008) and that was not the case for CRP (p = 0.097).

**Conclusions:** Both CRP and PCT in patients with suspected ventilator associated lower respiratory tract infection were higher in VAP than VAT. PCT presented a higher false negative rate. The level of PCT showed a good correlation with clinical severity.

**References**

1. Martin-Loeches I et al. Lancet Respir Med 2015, 3:859–68

#### A701 Evolution of blood lactate and 90-day mortality in critically ill patients with sepsis and septic shock. A posthoc analysis from the observational finnaki study

##### E. Varis^1,2^, V. Pettilä^3,4^, M. Poukkanen^3,5^, S. Jacob^6^, S. Karlsson^7^, A. Perner^8^, J. Takala^4^, E. Wilkman^3^, The FINNAKI Study Group

###### ^1^Helsinki University/Helsinki University Hospital, Division of Anesthesia, Intensive Care and Pain Medicine, Hus, Finland; ^2^Vaasa Central Hospital, Vaasa, Finland; ^3^Helsinki University/Helsinki University Hospital, Division of Anesthesia, Intensive Care and Pain Medicine, Helsinki, Finland; ^4^Inselspital, Bern University Hospital and University of Bern, Bern, Switzerland; ^5^Lapland Central Hospital, Rovaniemi, Finland; ^6^Inselspital, Bern University Hospital and University of Bern, Bern, Finland; ^7^Tampere University Hospital, Tampere, Finland; ^8^Rigshospitalet, Copenhagen University Hospital, Copenhagen, Denmark

####### **Correspondence:** E. Varis - Vaasa Central Hospital, Vaasa, Finland; ^3^Helsinki University/Helsinki University Hospital, Division of Anesthesia, Intensive Care and Pain Medicine, Helsinki, Finland

**Introduction:** Hyperlactatemia is known to predict mortality in patients with sepsis and septic shock. Elevated blood lactate concentrations are conventionally viewed as a surrogate of hypoperfusion and tissue hypoxia. Despite its limitations hyperlactatemia is an independent predictor of mortality and thus it was included in the most recent septic shock definition. However, both the evolution and the time to normalization of lactate in representative cohorts of septic patients receiving vasopressors are largely unknown.

**Objectives:** Our aim was to investigate the evolution of blood lactate level over time and its relationship to 90-day mortality in critically ill septic patients with hypotension and in patients with septic shock according to the recently revised definition.

**Methods:** We performed a post-hoc analysis from the prospective, observational, multicenter FINNAKI study. We analyzed 513 septic patients with hypotension, lactate recordings within 4 hours of ICU admission and repeat recordings of lactate during the ICU. 265 patients fulfilled the new definition for septic shock. The primary outcome was the association of time-weighted mean lactate during ICU stay with 90-day mortality.

**Results:** The 90-day mortality for all patients was 33.3 %. In patients with admission lactate of > 2 mmol/L the 90-day mortality was higher than in those with an admission lactate of ≤ 2 mmol/L (43.4 % vs 22.5 %, respectively p < 0.001). Patients with persistent lactatemia (>2 mmol/L) at 72 hours had higher 90-day mortality compared those with a lactate value of ≤ 2.0 mmol/L (52.0 % vs. 24.3 %, respectively, p < 0.001). Time-weighted mean lactate values were significantly higher in 90-day non-survivors than in survivors, 2.05 (1.38-4.22) mmol/L vs. 1.29 (0.98-1.77) mmol/L, p < 0.001. The median time to normalization of lactate was similar for 90-day survivors and non-survivors (15.0 (5.0-35.0) vs 17.0 (3.5-43.5) hours, p = 0.67). In separate backwards logistic regression analyses time-weighted mean lactate, the lactate value at 72 hours, and a lactate value over 2 mmol/L at 72 hours (dichotomous variable) were independently associated with 90-day mortality, but admission lactate or time to normalization of lactate were not.

**Conclusions:** In septic patients with hypotension, and in those who additionally had hyperlactatemia, time-weighted mean lactate, lactate values at 72 hours, and persisting hyperlactatemia at 72 hours were independently associated with 90-day mortality, but time to normalization of lactatemia and admission lactate were not.

**References**

1. Garcia-Alvarez M, Marik P, Bellomo R. Sepsis-associated hyperlactatemia. Crit Care. 2014;18(5):503.

2. Shankar-Hari M, Phillips GS, Levy ML, Seymour CW, Liu VX, Deutschman CS, et al. Developing a New Definition and Assessing New Clinical Criteria for Septic Shock: JAMA. 2016;315(8):775–87.

#### A702 Adrenomedullin and endothelin-1 are associated with myocardial injury and death in septic shock patients

##### O.H.M. Lundberg^1^, L. Bergenzaun^1^, J. Rydén^1^, M. Rosenqvist^2^, O. Melander^3^, M.S. Chew^4^

###### ^1^Skane University Hospital, Intensive and Perioperative Care, Malmö, Sweden; ^2^Skane University Hospital, Infectious Diseases, Malmö, Sweden; ^3^Lund University, Internal Medicine, Malmö, Sweden; ^4^Linkoping University, Anesthesiology and Intensive Care, Linkoping, Sweden

####### **Correspondence:** O.H.M. Lundberg - Skane University Hospital, Intensive and Perioperative Care, Malmö, Sweden

**Introduction:** Adrenomedullin and endothelin-1 are hormones with opposing effects on the cardiovascular system. Adrenomedullin acts as a vasodilator and seems to be important for the initiation and continuation of the hyperdynamic circulatory response in sepsis. Endothelin-1 is a vasoconstrictor and has been linked to decreased cardiac performance. Few studies have studied the relationship between adrenomedullin and endothelin-1 and morbidity and mortality in septic shock patients. High-sensitivity troponin T (hsTNT) is normally used to diagnose acute cardiac injury but is also prognostic for outcome in intensive care.

**Objectives:** We investigated the relationship between mid-regional pro-adrenomedullin (MR-proADM), c-terminal pro-endothelin-1 (CT-proET-1) and myocardial injury, measured using transthoracic echocardiography and hsTNT in septic shock patients. We were also interested in the development of different biomarkers throughout the ICU stay, and how early measurements were related to mortality. Further, we assessed if a positive biomarker panel, consisting of MR-proADM, CT-proET-1 and hsTNT changed the odds for mortality.

**Methods:** A cohort of 53 consecutive patients with septic shock had their levels of MR-proADM, CT-proET-1, hsTNT and left ventricular systolic functions prospectively measured over 7 days. The relationship between day 1 levels of MR-proADM/CT-proET-1 and myocardial injury was studied. We also investigated the relationship between biomarkers and early (7 day) as well as later (28 day) mortality. Likelihood ratios, pre-and posttest odds for mortality were calculated.

**Results:** Levels of MR-proADM and CT-proET-1 were significantly higher among patients with myocardial injury and were correlated with left ventricular systolic dysfunction. MR-proADM and hsTNT were significantly higher among 7 and 28 day non-survivors. CT-proET-1 was also significantly higher among 28 but not 7 day non-survivors. A positive biomarker panel consisting of the three biomarkers increased the odds for mortality 13-20-fold.

**Conclusions:** MR-proADM and CT-proET-1 are associated with myocardial injury. A biomarker panel combining MR-proADM, CT-proET-1 and hsTNT increases the odds ratio for death, and may improve currently available scoring systems in critical care.

**Grant acknowledgement**

Region Halland County Council

#### A703 Circulating levels of advanced glycation end products (AGES) are associated with 28-day mortality in septic patients

##### E. Rodriguez-Ruiz^1^, R. Hernández Vaquero^1^, A. Lopez Lago^1^, J.L. Garcia Allut^1^, A. Estany Gestal^2^, M.A. Garcia Gonzalez^3^

###### ^1^Hospital Clínico Universitario, Intensive Care Unit, Santiago de Compostela, Spain; ^2^Fundación Ramón Dominguez, Unidad de Epidemiología e Investigación Clínica, Santiago de Compostela, Spain; ^3^Instituto de Investigaciones Sanitarias, Grupo de genética y Biología del Desarrollo de las Enfermedades Renales, Santiago de Compostela, Spain

####### **Correspondence:** E. Rodriguez-Ruiz ^_^ Hospital Clínico Universitario, Intensive Care Unit, Santiago de Compostela, Spain

**Introduction:** Multiple biomarkers have been studied to assess sepsis severity and prognosis. The pathological role of the advanced glycation end products (AGEs) has become increasingly evident in numerous types of diseases. A large number of papers have shown that circulating levels of AGEs are elevated under inflammatory and/or diabetic conditions, and associated with cardiovascular disease. Also, accumulation of AGEs in various tissues, progresses at a physiological normal aging, and at an extremely accelerated rate under hyperglicemic, inflammatory, and/or oxidative stress conditions.

**Objectives:** Our aim was to study the dynamics of the plasma circulating levels of AGEs, and of the accumulated AGEs in the skin in septic patients during the first 5 days of the septic process, and their association with (i) APACHE II, (ii) SOFA on days 1 to 5, and (iii) mortality at day 28.

**Methods:** We assessed 48 consecutive patients admitted to our ICU with the diagnosis of sepsis. We determined for 5 consecutive days plasma circulating AGEs and skin AGEs. Plasma AGEs were measured by quantitative fluorescence spectroscopy analysis of plasma according to the method of Munch *et al.* (1) in a multi-mode microplate reader (Synergy 2, Biotek, Winooski, VT, USA), and results were expressed as arbitrary units (AU). Skin autofluorescence was measured with the AGE Reader mu (DiagnOptics, Groningen, The Netherlands) (2). We correlated these results with the APACHE II, daily SOFA, and mortality at day 28.

**Results:** There were 43 survivors and 5 non-survivors. We did not find association between plasma AGEs on days 1 to 5, and APACHE II or SOFA on days 1 to 5; neither between skin autofluorescence on days 1 to 5, and APACHE II or SOFA on days 1 to 5.

When analyzing mortality at day 28, we found that on day 1, plasma AGEs in non-survivors were significantly higher (37,4 ± 19,1) than in survivors (24,3 ± 11,2) (*p* = 0.027); the receiver operating characteristic analysis (AUC = 0.742, *p* = 0.079), determined a trend but not power enough to predict 28-day mortality. On days 2 to 5, we did not find differences in plasma AGEs between non-survivors and survivors. Skin autofluorescence on days 1 to 5, did not show association with 28-day mortality.

**Conclusion:** In our cohort of septic patients, increased plasma levels of AGEs on day 1, are associated with 28-day mortality. Circulating AGEs become an early biomarker in septic patients, and its prognostic efficacy needs to be determined in a larger study.

**References**

(1) Munch G. *et al*. (1997) Determination of advanced glycation end products in serum by fluorescence spectroscopy and competitive ELISA. Eur J Clin Chem Clin Biochem 35: 669–677.

(2) Meerwaldt R *et al*. (2004) Simple non-invasive assessment of advanced glycation endproducts accumulation. Diabetologia. 47:1324–1330.

#### A704 A study of patients with sepsis who have discrepancy between lactate levels and ScvO2

##### Y. Kishihara, H. Yasuda

###### Musashino Red Cross Hospital, Tokyo, Japan

####### **Correspondence:** Y. Kishihara - Musashino Red Cross Hospital, Tokyo, Japan

**Introduction:** The usefulness of lactate levels and ScvO2 is well known in treating patients with sepsis. We have encountered discrepancies between lactate levels and ScvO2, but the reason for this discrepancy is unknown.

**Objectives:** Our hospital joined the transpulmonary thermodilution (TPTD) study, which is a multicenter, prospective, randomized controlled trial. Inclusion criteria were ICU patients with sepsis undergoing mechanical ventilation. Various data were collected using TPTD and compared with CVP for transfusion. Primary outcome was ventilator-free days and secondary outcomes were ICU length of stay, 28-day mortality, and length of hospital stay. We used these study data to determine factors associated with the discrepancy between lactate clearance and ScvO2.

**Methods:** Based on TPTD study data, we subdivided patients into the following four groups: ScvO2 ≥ 70 % and lactate level ≥ 4 mmol/L (HH group), ScvO2 ≥ 70 % and lactate level < 4 mmol/L (HL group), ScvO2 < 70 % and lactate level ≥ 4 mmol/L (LH group), and ScvO2 < 70 % and lactate level < 4 mmol/L (LL group). Evaluations included the SOFA score, SAPS II score, lactate level, ScvO2, BNP, etc. We also analyzed mortality at 28 days, duration of mechanical ventilation, use of renal replacement therapy, duration of catecholamine use, and ICU length of stay.

**Results:** In total, 57 patients were included: HH group (n = 11), HL group (n = 24), LH group (n = 6), and LL group (n = 16). There were no differences in patients' characteristics or change in lactate levels, CVP, and ScvO2 from 24 h to 72 h. Mortality at 28 days was 3/6 (50 %) in the LH group, 3/11 (27.3 %) in the HH group, 3/16 (18.8 %) in the LL group, and 3/24 (12.5 %) in the HL group (p < 0.001). On multivariate analysis, the odds of 28-day mortality were 8.16 (95 % CI: 0.2-275) in the LH group, 1.46 (95 % CI: 0.05-26.8) in the HH group, and 1.34 (95 % CI: 0.17-11.1) in the LL group.

**Conclusions:** Mortality at 28 days was highest in the LH group and lowest in the HL group. These data are not conflicted our experience and past studies. Because 28-day mortality was higher in the HH group than in the LL group, it is possible that lactate levels are more predictive of mortality.

**References**

1. Crit Care Med. 2010;38:367–374.

2. JAMA. 2010;303:739–746.

3. Am J Respir Crit Care Med. 2010;182:752–761.

#### A705 N-terminal pro-brain natriuretic peptide for the prognostic utility in patients with severe sepsis or septic shock in intensive care unit

##### S. Rebollo^1^, L. García de Guadiana-Romualdo^2^, R. Jimenez^3^, P. Esteban Torrella^2^, A. Fernandez^1^, S. Sanchez^1^, A. Ortin^1^

###### ^1^Hospital General Universitario Santa Lucia, Intensive Care, Cartagena, Spain; ^2^Hospital General Universitario Santa Lucia, Biochemistry Department, Cartagena, Spain; ^3^Hospital General Universitario Santa Lucia, Cartagena, Spain

####### **Correspondence:** S. Rebollo - Hospital General Universitario Santa Lucia, Intensive Care, Cartagena, Spain

**Introduction:** Sepsis is a leading cause of death in critically ill patients despite improvements in antimicrobial therapy and supportive care. Early identification of patients at high risk of dying after intensive care unit (ICU) admission may help determine therapeutic interventions.

Objective: The aim of this study was to evaluate the predictive value of N-terminal pro- brain natriuretic peptide (NT-proBNP) for in-hospital mortality in a cohort of patients admitted to ICU with sepsis ands and septic shock.

**Methods:** Patients: 47 patients [mean age: 64.3 years (SD:17.2); male: 25 (53.2 %)] with diagnosis of sepsis and septic shock, according to the Third International Consensus Definitions for Sepsis and Septic Shock (Sepsis-3), admitted to ICU between October 2013 and March 2014 were enrolled.

Measurements: Blood tests for C-reactive protein (CRP), procalcitonin (PCT) and NT-proBNP analyses were drawn on the day of admission and 48 hours later. Patients' demographic data and severity scores (APACHE II, SAPS and SOFA) were collected. In-hospital mortality was defined as outcome. For measurement of NT-proBNP, a chemiluminescent inmunoassay (NT-proBNP Dimension Vista Siemens Heathcare) and an ECLIA assay (NT-proBNP Cobas 8000 Roche Diagnostic) were used.

Statistical analysis: Receiver operating characteristic (ROC) curves and areas under the curves (AUCs) were calculated to evaluate the prognostic value for in-hospital mortality of NT-proBNP of each biomarker and optimal cutoff value was derived from ROC curves. Value p < 0.05 was considered statistically significant. Statistical analysis was done using SPSS v. 20.0 and and MedCalc statistical software v.14.8.1.

**Results:** In-hospital mortality rate was 34 % (16 of 47). NT-proBNP levels at admission were significantly higher in non-survivors (median: 22546 pg/mL [IQR: 29958]) compared with survivors (median: 2310 pg/mL [IQR: 9219]; p < 0.001), and the difference remained after 48 hrs (p = 0.001). The ROC of admission and 48-hours NT-proBNP levels for in-hospital mortality resulted in AUC values of 0.88 (95 % confidence interval (CI): 0.77-0.99; p < 0.001) and 0.82 (95 % CI: 0.66-0.99; p < 0.001), respectively, with a similar performance than that of SOFA (0.84 (95%CI: 0.7-0.95; p < 0.001), APACHE II (0.86 (95%CI: 0.76-0.97; p < 0.001) and SAPS II (0.88; 95%CI:0.78-0.98; p < 0.001). There were not differences among survivors and non-survivors for PCT and CRP on admission and 48-hours PCT and CRP.

**Conclusions:** NT-proBNP values are frequently increased in sepsis and septic shock. Values are significantly higher in non-survivors than survivors. Although NT-proBNP on admission and at 48 hours showed a good performance for prognosis of in-hospital mortality, with AUC ROC > 0.8 for both, they did not improve the value of severity scores usually used in ICUs.

#### A706 Diagnostic value of systemic interleukin-1 receptor antagonist for ventilator associated pneumonia in a porcine model of mechanical ventilation

##### G. Li Bassi^1^, R. Guillamat Prats^2^, A. Artigas^2^, E. Aguilera^3^, D. Marti^3^, O.T. Ranzani^3^, M. Rigol^3^, L. Fernandez^3^, M. Ferrer^3^, I. Martin-Loeches^4^, A. Torres^3^

###### ^1^Hospital Clinic, Pulmonary and Critical Care Medicine, Barcelona, Spain; ^2^Corporació Parc Taulí, Universitat Autonoma de Barcelona, Parc Taulí, Sabadell, Spain; ^3^Hospital Clinic, Barcelona, Spain; ^4^Trinity College, Dublin, Ireland

####### **Correspondence:** G. Li Bassi - Hospital Clinic, Pulmonary and Critical Care Medicine, Barcelona, Spain

**Introduction:** We previously investigated efficacy of a new ventilatory strategy - aimed at promoting mucus clearance and preventing pulmonary aspiration - or the Trendelenburg position for the prevention of ventilator-associated pneumonia (VAP) (1).

**Objectives:** We now appraise patterns of systemic inflammation to identify blood inflammatory biomarkers that could detect VAP or ventilator-associated tracheobronquitis (VAT). Also, we evaluated the effects of the Trendelenburg position on systemic inflammation.

**Methods:** Twenty-one pigs were intubated and on mechanical ventilation (MV) for 72 hours. Following surgical preparation, pigs were randomized to be positioned: 1) in semirecumbent/prone position, ventilated with a duty cycle (T_I_T_TOT_) of 0.33 and without PEEP (control); 2) as in the control group, PEEP of 5 cm H_2_O, and T_I_T_TOT_ to achieve a mean expiratory-inspiratory flow bias of 10 L/min (treatment); 3) in Trendelenburg/prone position and ventilated as in the control group (Trendelenburg). Following randomization, *P. aeruginosa* was instilled into the oropharynx. Prior to bacterial challenge, and every 24 hours thereafter, blood was drawn for quantification of serum interferon (INF)-Gamma; interleukin (IL)-1α; IL-1β; IL-1 receptor antagonist (RA); IL-2; IL-4; IL-6; IL-8; IL-10; IL-12; IL-18, tumor necrosis factor (TNF)-α, tissue factor; angiotensin-2; adrenomedullin and protein C reactive. VAP and VAT were clinically suspected and confirmed by histological/microbiological studies.

**Results:** Overall, ten animals developed VAP (4 controls, 6 treatments and 0 Trendelenburg), four animals VAT (1 control, 0 treatments, 3 Trendelenburg) and 6 did not develop any pulmonary infection (1 control, 1 treatment and 4 Trendelenburg). Median [IR] tracheal aspirate quantitative culture of *P.aeruginosa* was 6 [1.2], 6.1 [6.3] and 6.5 [0.4] log10 cfu/mL (p = 0.018) and *P.aeruginosa* pulmonary burden was 0 [1.6], 0 [0.9] and 2.2 [4.3] in animals without infection or that developed VAT and VAP, respectively (p < 0.001). Study treatments significantly changed INF-γ, IL-1Ra, IL-4, IL-8 and IL-18 concentrations (p < 0.05 for all comparisons). Multivariate analyses showed that cytokines did not predict VAT; yet, IL-1Ra was strongly associated with VAP (odds ratio 64.941 (CI 1.638 - ≻ 999), area under the ROC curve 0.85 (0.64-1.00). A value of 1.21 log pg/mL appeared to diagnose VAP most accurately (sensitivity 90 %, specificity 80 %).

**Conclusions:** In a reliable animal model of MV and acquired pulmonary infections, IL-1Ra predicts *P.aeruginosa* VAP with good diagnostic accuracy. Consistently with previous findings, the Trendelenburg position reduced incidence of VAP, and decreased inflammation during MV. Further clinical studies are warranted to confirm these preliminary findings.

**References**

1) Li Bassi G et al. *Crit Care Med* 2014; 42: e620-7

**Grant acknowledgement**

ECCRN ALAIN HARF AWARD; MINECO Plan nacional I + D (SAF2012-33744)

#### A707 Patterns of c-reactive protein ratio response to antibiotics in pediatric sepsis: a prospective cohort study

##### V.S. Lanziotti^1,2^, P. Póvoa^3^, L. Pulcheri^4^, M.O. Ribeiro^1^, A.P. Barbosa^5,6^, J.R. Lapa e Silva^7^, M. Soares^2^, J.I.F. Salluh^2^

###### ^1^Federal University of Rio de Janeiro, Paediatric Intensive Care Unit, Rio de Janeiro, Brazil; ^2^D'Or Institute for Research and Education, Intensive Care Research, Rio de Janeiro, Brazil; ^3^Nova Medical School (Universidade Nova de Lisboa), Intensive Care Unit, Lisboa, Portugal; ^4^Rede D'Or São Luiz, Paediatric Intensive Care Unit - Rio's D'Or Hospital, Rio de Janeiro, Brazil; ^5^D'Or Institute for Research and Education, Paediatric Research, Rio de Janeiro, Brazil; ^6^Federal University of Rio de Janeiro, Paediatric Intensive Care Unit and Paediatric Department, Rio de Janeiro, Brazil; ^7^Federal University of Rio de Janeiro, Internal Medicine Department, Medical School, Rio de Janeiro, Brazil

####### **Correspondence:** V.S. Lanziotti - D'Or Institute for Research and Education, Intensive Care Research, Rio de Janeiro, Brazil

**Introduction:** Sepsis is a leading cause of mortality in children. The mortality remains unchanged, despite advances in treatment and the clinical judgement is still insufficient to an early recognition of outcomes in children with sepsis.

**Objective:** To evaluate the patterns of C-Reactive Protein (CRP) ratio response to antibiotic therapy during the first 7 days in the Paediatric Intensive Care Unit (PICU) in children admitted with sepsis.

**Methods:** This is a multicenter, prospective and descriptive cohort study of children (1 month to 18 years old) admitted at PICUs of 3 tertiary hospitals in Rio de Janeiro, Brazil, with sepsis diagnosis (less than 72 hours). CRP was sampled every other day during the first week of antibiotic therapy (from D0 to D6). CRP-ratio was calculated in relation to D0 CRP value. Patients were classified according to an individual pattern of CRP-ratio response with the following criteria: fast response - when D4 CRP was ≤ 0.4 of D0 CRP concentration; slow response - when D4 CRP was > 0.4 and D6 ≤ 0.8 of D0 CRP concentration (continuous and slow response); nonresponse - when D6 CRP was > 0.8 of D0 CRP concentration; biphasic response - characterized by an initial CRP decrease to levels < 0.8 of the D0 CRP followed by a secondary rise > 0.8. We performed comparison between PICU survivors and non-survivors.

**Results:** This cohort included 99 patients (age median: 1.8; 52.5 % male); the most frequent sites of infection were lung (58.6 %), central nervous system (13.1 %), abdominal (7.1 %) and urinary (5.1 %). Out of the 99 patients, 88 could be classified according to a predefined pattern: 26.1 % fast response, 54.6 % slow response, 10.2 % nonresponse and 9.1 % biphasic response. The ICU mortality rate was significantly different (p < 0.001) according to the patterns of CRP-ratio response: fast response 4.3 %, slow response 2.1 %, nonresponse 44.4 % and biphasic response 37.5 %. Besides, the time dependent analysis of CRP-ratio and CRP course of the different patterns was significantly different (p < 0.001).

**Conclusions:** In paediatric sepsis, sequential evaluation of CRP-ratio was useful in the early identification of patients with poor outcome. The identification of CRP-ratio pattern of response to antibiotics during the first week of antibiotic therapy could be useful in the recognition of the individual clinical evolution, influencing clinical decision-making process at bedside.

#### A708 HIF-1 α levels and inflamation biomarkers in hypoxaemic septic shock patients

##### I. Palacios Garcia^1^, A. Diaz Martin^2^, M. Gil Marqués^2^, A. Puppo Moreno^2^, A. Gutierrez Pizarraya^2^, J. Pachón Diaz^2^, M. Pachón Ibañez^2^, Y. Smani^2^, M. Mc Connell^2^

###### ^1^Hospital Virgen del Rocio, Seville, Spain; ^2^Hospital Virgen del Rocio, Sevilla, Spain

####### **Correspondence:** I. Palacios Garcia - Hospital Virgen del Rocio, Seville, Spain

**Objectives:** The aim of this study is to analyse the relationship between tissue hypoxia, levels of hypoxia-inducible factor 1α (HIF-1α, a factor that activates gene expression in response to inflammatory and hypoxaemic conditions) and the innate immune response in patients with septic shock, and its relationship with mortality.

**Method:** Prospective observational study in patients with septic shock and healthy volunteers (controls) to analyse normal values of non-standard determinations (pyruvate, HIF-1α, and HLA-DR on circulating monocytes). Individuals signed an informed consent and the study was approved by the Ethics Committee. Tissue hypoxia was monitored by non-invasive transcutaneous spectroscopy (NIRS). At inclusion, 3rd and 7th day, venous blood samples were obtained for determinations above were obtained. Lactate was determined at baseline, every 6 hours the first day and daily until day 3. Quantitative variables expressed as median [IQR]. Statistical analysis: Mann–Whitney test ranges with Wilcoxon test and Spearman rho; it was considered significant a value of p < 0.05.

**Results:** Eleven patients with septic shock were included, 72.7 % males (8 cases), median age 67 years [58–73] and 10 healthy controls. The clinical severity APACHE II at ICU admission was 21 [17–25]. ICU mortality was 19 % (2 cases).

At inclusion all patients had respiratory failure (SOFA respiratory ≥2, SaO2 76.3 % [71.2-81]), venous lactate >4 mmol/l, peripheral tissue hypoxia data (NIRS median 58 % [49–70]), and SOFA 9 [7.5-11.5]. All patients had elevated levels of leukocytes, CRP, serum lactate and pyruvate. Pyruvate levels were higher in patients than in controls.

HIF-1α was higher in patients, reaching peak levels on day 3rd (2.06 times the inclusion day). In patients: a) levels of HLA-DR on monocytes circulating inclusion day were 2.86 times lower than in controls; b) IL-6 and IL-10 levels in patients were higher than in controls on the day of inclusion, with marked reduction at day 3rd (26.25 and 6.98 times respectively), IL6 was 1088.49 pg/ml and 0.17 pg/ml, respectively. IL10 values were 121.66 pg/ml in patients being undetectable in controls; c) there were no differences in HIF-1α, IL-6, IL-10 and tissue hypoxia between dead and survivors. In the follow up, an inverse relationship between pyruvate and HIF-1α levels was observed.

**Conclusions:** Patients with septic shock show peripheral tissue hypoxia. HIF-1α appears high and rising until the third day of monitoring, which despite a decrease in the levels of IL-6 and IL-10 in those days was observed.

#### A709 Identifying sepsis endotypes and time of onset from interleukin-6 trajectories in septic shock patients

##### L.A. Zhang^1^, R.S. Parker^1,2,3^, I. Banerjee^1,3,4^, G. Clermont^1,2,4^

###### ^1^University of Pittsburgh, Department of Chemical Engineering, Pittsburgh, USA; ^2^University of Pittsburgh, CRISMA Center, Department of Critical Care Medicine, Pittsburgh, USA; ^3^University of Pittsburgh, McGowan Institute for Regenerative Medicine, Pittsburgh, USA; ^4^University of Pittsburgh, Department of Bioengineering, Pittsburgh, USA

####### **Correspondence:** L.A. Zhang - University of Pittsburgh, Department of Chemical Engineering, Pittsburgh, USA

**Introduction:** The high inter- and intra-patient variability and confounding factors during early sepsis has made it difficult to diagnose and characterize. Despite this variability, serum cytokine levels such as interleukin-6 (IL-6) during sepsis appear to follow consistent and distinctive trajectories across patients.

**Objectives:** To classify septic patients into groups defining endotypes of sepsis using longitudinal IL-6 trajectories and to estimate time of sepsis onset.

**Methods:** The multicenter, randomized Protocol-Based Care for Early Septic Shock (ProCESS) trial enrolled 1341 patients with septic shock and showed a 16.3 % 14-day mortality. Patients with IL-6 measured at 0, 6, 24, and 72 hours after enrollment were selected for this analysis. Those with IL-6 values below the lower limit of detection or with missing measurements were excluded, leaving 164 patients for analysis.

Each IL-6 trajectory was modeled as a differential equation evolving in response to inflammation. 3 classes of differential equations were used to enforce classification into specific dynamic behaviors: over-damped response, under-damped response, and overshoot-type response. Patient classification was performed based on best data fit to one of the 3 possible responses.

Within each class of response, a Gaussian Mixture model on differential equation parameters was used to further classify each patient. The centers of each cluster generated a characteristic IL-6 response to sepsis (a master curve) for that sub-population. Aligning each patient along with their category master curve provided an estimate for disease time-zero with respect to onset of sepsis.

**Results:** Subgroup analysis revealed a total of 5 distinct IL-6 responses: 1-high response and fast decline; 2- slow and protracted response; 3- fast response and protracted decline; 4- small response and fast decline; 5- sustained response. Statistical testing of clinical biomarkers between clusters revealed significant differences across 12 hour bins following disease onset. There were notable differences in vital signs, bilirubin, lactate, and urine output, establishing a correspondence between trajectory endotypes and physiological variables. This suggests that endotypes can be informed from easy and minimally-invasive measurements. Finally, endotypes were associated with distinct low and high mortality outcome. Endotype 5 exhibited particularly high mortality.

**Conclusions:** A novel data-driven approach is proposed to generate sepsis endotypes and disease time-zero. The clusters provided physiological characterization of phenotypes. The knowledge of disease time as well as endotype risk can be used to guide treatments in order to shift sepsis patients towards better outcomes.

**Grant acknowledgement**

US DOE GAANN P200A120195 and US NIH R01-GM-105728

### Biomarkers of outcome in neurointensive care

#### A710 The impact of acute cardiac events on long-term mortality after subarachnoid hemorrhage

##### E. Norberg, J. Oras

###### University of Gothenburg, Institue of Clinical Sciences, Gothenburg, Sweden

####### **Correspondence:** J. Oras - University of Gothenburg, Institue of Clinical Sciences, Gothenburg, Sweden

**Introduction:** Acute cardiac events are common after subarachnoid hemorrhage (SAH) and are associated with an increased risk of poor outcome (1–3). However, the impact of cardiac events on long term mortality after SAH are barely studied (4).

**Objectives:** The aim was to evaluate the impact on acute cardiac events on long-term mortality after SAH. Our hypothesis was that these events are associated with an increased long-term mortality.

**Methods:** This is a retrospective study. Medical records from all patients admitted to our NICU from 2010 to 2015 with the diagnosis SAH were analysed. Admission variables obtained were age, medical history, WFNS score, modified fischer grade. Cardiac varibales obtained were troponin and NTproBNP levels, ECG, echocardiograpic evaluation. Kaplan-Meier curves and Cox-regression were used in the statistical analyses.

**Fig. 96 (abstract A710).**
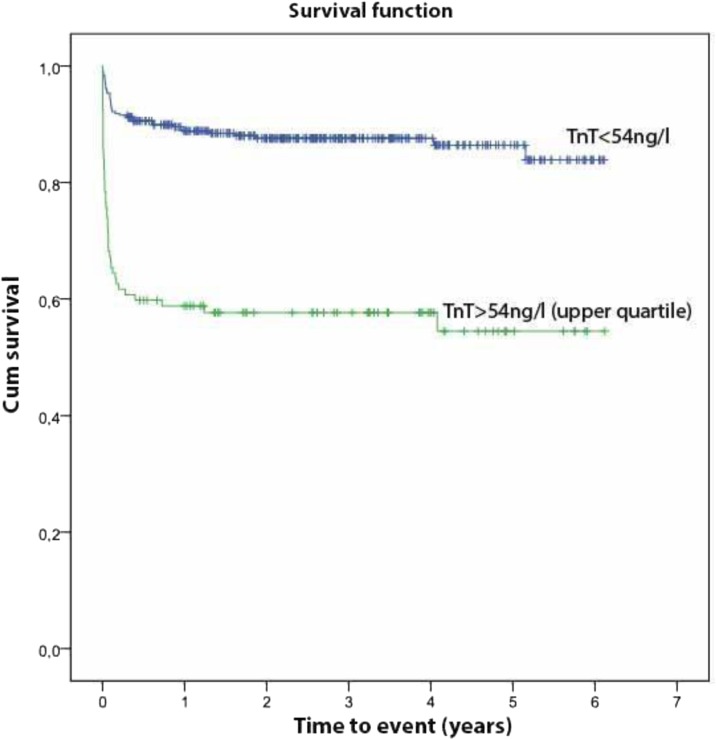
ᅟ

**Results:** A total of 708 patients were admitted with suspected/verified SAH during the study period. 118 patients did not fulfill SAH-diagnosis, 33 patients were not included due to long time to admission (>4 days). Cardiac variables were found in the following number of patients; troponin levels n = 428, NTproBNP levels n = 342, ECG n = 454, echocardiography n = 235. In 28 patients no ECG/echo/cardiac biomarkers were found. Of the 529 patients analysed, 109 patients had died at the time mortalily data was obtained (April 2016). In a univariate cox-regression analysis, WFNS grade 4–5, Fisher grade, age, history of hypertension, cardiovascular disease (CVD) or renal disease, levels of troponin and NTproBNP and ST-elevations or negative T-waves had a significant higher risk (hazard ratio) for death. Stress-cardiomyopathy was not significantly associated with an inreased risk of death. Fig. 96 shows a Kaplan Meier curve of cumulative survival in patients with troponin release, divided in upper or lower quartiles. In a multivariable cox-regression analysis, both troponin levels (Table 70) and NTproBNP levels were significantly associated with an increased risk for death when adjusting for the most important admission variables; WNFS grade 4–5, age and history of CVD or renal disease. However, these differences were only significant the first year after the hemorrhage.

**Conclusions:** Acute realease of the cardiac biomarkers troponin and NTproBNP after SAH is an omnious clinical sign which is associated with an increased risk of death, especially the first year after the hemorrhage. Further research is needed to address whether measures to optimize cardiac treatment after SAH might improve survival.

**References**

(1) Oras et al., Critical Care 2016.

(2) van der Bilt et al., Neurology 2014.

(3) Naidech et al., Circulation 2005.

(4) Zaroff et al., Neurocritical Care 2012

**Table 70 (abstract A710).** Multivariable Cox-regression analysis

	Hazard ratio	Lower 95% CI	Upper 95% CI	p-value
WFNS 4-5	3.69	2.34	5.82	<0.001
Age (per year)	1.05	1.03	1.07	<0.001
History of CVD or renal disease	2.06	1.21	3.48	0.007
Troponin, per 100ng/l	1.09	1.02	1.16	0.010

#### A711 Myocardial function at the early phase of traumatic brain injury: a prospective controlled study

##### A. Cuisinier^1^, C. Maufrais^2^, J.F. Payen^1,3,4^, S. Nottin^5^, G. Walther^5^, P. Bouzat^1,3,4^

###### ^1^Centre Hospitalier Universitaire de Grenoble, Pôle Anesthésie Réanimation, La Tronche, France; ^2^Department of Medicine, University of Fribourg, Laboratory of Integrative Cardiovascular and Metabolic Physiology, Fribourg, Switzerland; ^3^Grenoble Institut des Neurosciences, INSERM U1216, Grenoble, France; ^4^Grenoble Alpes Université, Grenoble, France; ^5^Avignon University, LAPEC EA4278, Avignon, France

####### **Correspondence:** A. Cuisinier - Centre Hospitalier Universitaire de Grenoble, Pôle Anesthésie Réanimation, La Tronche, France

**Introduction:** The concept of brain-heart interaction (1) has been described in several brain injuries. Traumatic brain injury (TBI) may also lead to cardiac dysfunction but evidences are mainly based upon experimental (2) and clinical retrospective studies (3).

**Objectives:** The present study aimed at evaluating myocardial function at the early phase of TBI using conventional 2D echocardiography and complementary STE analysis. We hypothesized that patients with TBI had altered cardiac function compared to the control group.

**Methods:** We conducted a prospective case–control study in a level I trauma center. Twenty consecutive adult patients with severe TBI were matched according to age and gender with twenty control patients. Control group included adult patients undergoing general anesthesia for peripheral trauma surgery. Conventional and Speckle Tracking Echocardiography (STE) was performed within the first 24 post-traumatic hours in the TBI group and PRE/PER-operative in the control group. Primary endpoint was left ventricle ejection fraction (LVEF) measured by the Simpson's method. Secondary endpoints included diastolic function and STE analysis.

**Results:** We found similar LVEF between the TBI group and the PER-operative control group (61 % [56–76]) vs. 62 % [52–70]). LV morphological parameters and systolic function were also similar between the two groups. Regarding diastolic function, isovolumic relaxation time was significantly higher in the TBI cohort (125 s [84–178] versus 107 s [83–141], p = 0.04), suggesting subclinical diastolic dysfunction. Using STE parameters, we observed a trend toward higher strains in the TBI group but only apical circumferential strain and basal rotation reached statistical significance. STE-derived parameters of diastolic function tended to be lower in TBI patients.

**Conclusions:** No systematic myocardial depression was found in a cohort of severe TBI patients. STE rather revealed correct adaptation of left systolic function, while diastolic function slightly impaired.

**References**

1 - Mertes P-M, Bruder N, Audibert G. Cardiac complications of acute brain injury. *Ann Fr Anesth Reanim* 2012;31:91-96.

2 - Shivalkar B, Van Loon J, Wieland W, *et al*. Variable effects of explosive or gradual increase of intracranial pressure on myocardial structure and function. *Circulation* 1993;87:230-239.

3 - Prathep S, Sharma D, Hallman M, *et al*. Preliminary report on cardiac dysfunction after isolated traumatic brain injury. *Crit Care Med* 2014;42:142-147.

**Grant acknowledgement**

The authors thank Carole Saunier, MD for providing ultrasound device.

**Fig. 97 (abstract A711).**
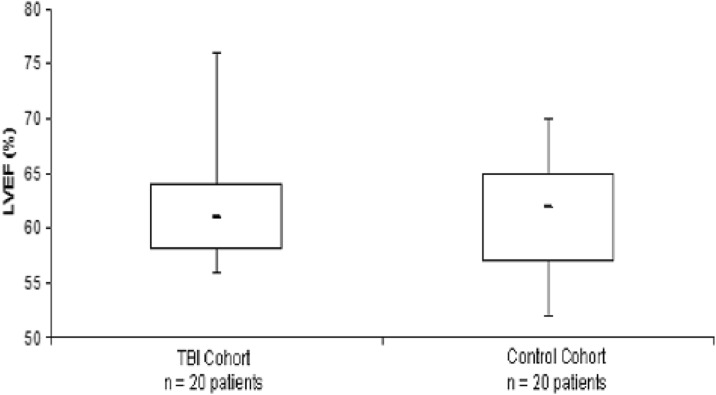
Primary outcome LVEF (%)

#### A712 Cerebro-spinal fluid glucose concentrations and insulin therapy after subarachnoid hemorrhage: a multicentre cohort study

##### S. Arib^1^, F. Bilotta^2^, R. Badenes^3^, F. Rubulotta^4^, S. Mirek^5^, I.A. Crippa^1^, B. Monfort^5^, E. Stazi^2^, A. Lozano Roig^3^, J. Creteur^1^, F.S. Taccone^1^

###### ^1^Hopital Erasme, Department of Intensive Care, Brussels, Belgium; ^2^Sapienza University, Department of Anesthesia and Intensive Care, Rome, Italy; ^3^Hospital Clinic Universitari, Department of Anesthesia and Intensive Care, Valencia, Spain; ^4^St Mary Hospital and Charing Cross Hospital Imperial College NHS Trust, Department of Anaesthesia and Intensive Care Medicine, London, UK; ^5^CHU Dijon, Department of Anesthesia and Intensive Care, Dijon, France

####### **Correspondence:** S. Arib - Hopital Erasme, Department of Intensive Care, Brussels, Belgium

**Introduction:** In patients with subarachnoid hemorrhage (SAH), monitoring brain metabolism through microdialysis catheters has revealed a decrease in brain glucose concentrations during intensive insulin therapy to control hyperglycemia. However, the use of such catheters is not routinely applied. No data are available on glucose concentrations into the cerebro-spinal fluid (CSF) of these patients during insulin therapy (IT).

**Methods:** We reviewed data in all patients admitted after a non-traumatic SAH over a 4-year period (2011–2014) in 5 ICUs. Inclusion criteria were: a) age > 18 years; b) presence of an external ventricular drain (EVD) for intracranial pressure monitoring; c) daily analysis of CSF including glucose concentrations for at least 4 consecutive days; d) concomitant analysis of glucose concentrations on the arterial blood gas analysis. Demographics and clinical characteristics were recorded on admission as well as the need for continuous IT (target glucose levels: 110–150 mg/dL) and 3-month neurological outcome (favourable outcome was defined as a Glasgow Outcome Scale of 4–5).

**Results:** Of a total of 724 patients admitted with SAH, 335 needed an EVD and 144 met the inclusion criteria for the final analysis (median age: 58 [49–66] years; male gender: 77/144). Median time from admission to EVD placement was 1 [0–3] days; EVD was maintained for a period of 7 [4–12] days. Of the 115 identified aneurysms, 83 were treated with endovascular therapy. Median Glasgow Coma Scale (GCS) on admission was 10 [7–13] and CT-scan Fisher scale was 4 [3–4]. A total of 46 (32 %) died during the ICU stay and 63 (44 %) had a favourable neurological outcome. Patients receiving IT on the first day of EVD monitoring (n = 72) had more frequently diabetes (27/72 vs. 4/72; p < 0.001) but similar GCS and Fisher scales than those without IT. Together with a higher CSF red blood cells count (65210 [11900–94192] vs. 24650 [6020–64188]/mm^3^; p = 0.006) and similar white blood cells and proteins levels, patients receiving IT had higher blood glucose (180 [151–222] vs. 140 [118–155] mg/dL; p < 0.001) and similar CSF glucose concentrations (89 [66–108] vs. 81 [72–92] mg/dL; p = 0.13) than those without IT. Thus, the ratio between CSF and blood glucose levels was significantly lower in patients receiving IT than others (0.51 [0.36-0.61] vs. 0.60 [0.49-0.70]; p = 0.006).

**Conclusions:** Insulin therapy resulted in lower proportion of glucose available in the CSF in patients with SAH. The impact of such findings on cerebral recovery and outcome needs to be further evaluated.

#### A713 Serial MRI for outcome prediction after aneurysmal subarachnoid haemorrhage

##### S. Magnoni^1^, M. Marando^2^, S. Pifferi^1^, V. Conte^1^, F. Ortolano^1^, M. Carbonara^1^, G. Bertani^1^, E. Scola^1^, M. Cadioli^3^, F. Triulzi^1^, A. Colombo^1^, N. Stocchetti^1,2^

###### ^1^Fondazione IRCCS Ca' Granda Ospedale Maggiore Policlinico, Milan, Italy; ^2^Milan University, Milan, Italy; ^3^Philips Healthcare, Milan, Italy

####### **Correspondence:** S. Magnoni - Fondazione IRCCS Ca' Granda Ospedale Maggiore Policlinico, Milan, Italy

**Introduction:** The immediate events associated with aneurysmal subarachnoid haemorrhage (SAH) result in early brain injury (EBI). Delayed cerebral ischemia (DCI) may also develop between 1–2 weeks after bleeding caused by vasospasm and other mechanisms. Characterizing both type of brain injury and standardising the diagnostic modalities is key to predicting and potentially modifying the outcome of SAH.

**Objectives:** Use serial MR imaging to determine the characteristics of early and late brain injury and investigate their role in determining outcome after SAH.

**Methods:** Patients' demographic and clinical data were collected. 6-months outcome was evaluated using the extended GOS (GOS-E). Serial MRI scans were performed on days 0–5 (early), and 7–14 (late) following the acute bleeding. The MRI protocol included 3D-T1, T2, T2*-weighted sequences, 3D-FLAIR, diffusion-weighted imaging (DWI) and apparent diffusion coefficient maps (ADC) were calculated. FLAIR, DWI/ ADC and T2 sequences were co-registered and volumes measured using the SPM and Analyze software. Early cerebral ischemia (ECI) and DCI were identified as hyper/hypointense lesions on the correspondent DWI/ADC maps. DWI were classified on the basis of the whole lesion volume: none (N), spotty (SP), ≤10 ml (S = small), and > 10 ml (L = large). Early cerebral haematomas (ECH) were identified on FLAIR and T2 and similarly classified as none (N), small (S) and large (L). All early lesions (excluding SP lesions) were named as EBI (ECI + ECH). Chi square test and logistic regression were used for univariable and multivariable analysis.

**Results:** 82 SAH patients (53 F and 29 M) were enrolled, with a median age of 57 (IQR 48–66). 54 subjects (66 %) had good grade (WFNSS 1–3) and 28 (34 %) poor grade SAH (WFNS 4–5) on admission. 67 patients (82 %) were coiled while 14 were surgically treated (17 %) and one was not treated. Vasospasm (defined as “late” clinical deterioration associated to instrumental finding of arterial narrowing) occurred in 22 patients (27 %). MRI abnormalities compatible with ECI were found in 34 subjects (42 %), 22 classified as S lesions (65 %) and 12 as L (35 %). 40 patients (49 %) had ECH (25 S and 15 L lesions), including 4 small subdural haematomas (SDH) and 2 with both SDH and ECH. DCI was observed in 38 % of the cases (N = 31), 27 classified as S lesions and 4 as L. The Table 71 shows the results of the univariable analysis for factors related to outcome.

Multivariable logistic regression analysis shows an association between worse outcome and poor WFNSS on admission (OR 6.3, 95 % CI, 2–20), the presence of DCI (OR 4.2, 95 % CI 1.4-13) and EBI (OR 1.3, 95 % CI 1.3-15).

**Conclusions:** These findings show that early and delayed brain injury are both independent predictors of worse outcome after SAH, and suggest that standard MRI protocols are useful diagnostic tools. Significant variance in outcome still remains, indicating that additional methods may be necessary to quantify brain damage after SAH.

**Table 71 (abstract A713).** Results of the univariable analysis

Variable	Favorable outcome	Unfavorable outcome	p-value
WFNSS (1–3) (4–5)	(41) (10)	(13) (18)	0,0004
Median age ( IQR)	57 (46–65)	61 (55–72)	0,0756
Clinical Vasospasm (Yes) (No)	(9) (42)	(13) (15)	0,0063
ECI (Yes) (No)	(17) (32)	(17) (14)	0,0758
ECH (Yes) (No)	(18) (31)	(22) (9)	0,0029
DCI (Yes) (No)	(13) (38)	(18) (13)	0,0032
EBI (ECI + ECH) (Yes) (No)	(7) (42)	(14) (17)	0,0022
ECI volume > 10 mL (Yes) (No)	(3) (46)	(9) (22)	0,0006
ECH volume > 10 mL (Yes) (No)	(6) (43)	(9) (22)	0,0609

#### A714 Brain injury biomarkers and inflammatory markers as prognostic factors in patients with spontaneous intracerebral hemorraghe

##### H.B. Rotzel, A. Serrano Lázaro, D. Aguillón Prada, M. Rodriguez Guimillo, C. Sanchís Piqueras, J. Romero Guia, M. García Simon, A. Mesejo Arizmendi, A. Carratalá

###### Hospital Clinic Universitari Valencia, Valencia, Spain

####### **Correspondence:** H.B. Rotzel - Hospital Clinic Universitari Valencia, Valencia, Spain

**Introduction:** Spontaneous intracerebral hemorrhage (ICH) is responsible for 9-27 % of cerebrovascular diseases worldwide, with 37–52 % of mortality at 30 days and 54 % a year. Brain injury biomarkers (BIB) and inflammatory markers (IM) have been studied to predict prognosis in this disease.

**Objectives:** To associated BIB and IM with mortality and functional outcome in patients with spontaneous ICH.

**Methods:** We performed a prospective observational study, with patients admitted in ICU with spontaneous ICH. BIB were determined (Enolase, S-100B, Dimer D (DD), BNP, CRP) at admission, 1,2, 3 and 7^th^ day, and IM (transferrin, albumin, prealbumin, Quick index (QI), leukocytes) at admission and 7^th^ day. APACHE II, SOFA, GCS, GRAEB were determined at admission and the modified Rankin Scale (mRS: poor outcome >2) and Glasgow Outcome Scale (GOS: poor outcome < 4) at ICU discharge. Variables were summarized using %, mean (SD) and median (minimal/ maximum). We used T-Student and χ2 (p < 0.05) for univariable analysis. We conducted a multivariable analysis for mortality with binary logistic regression (95 % CI, OR) p < 0,05. We obtained a ROC curve for those variables that were independent factors for mortality.

**Results:** We enrolled 120 patients, 68 % were men, mean age 62 years(±12.6). Global mortality was 34.2 %. 83.3 % were supratentorial and 16.7 % infratentorial. Mean APACHE II 14 (±6, 5) and GCS 10.4 (±4,1) and median SOFA 4 (0–14) and hematoma volume 21,35 cc3 (1–252). In the univariable analysis a worse outcome (with mRS and GOS) was significantly associated with APACHE II, SOFA, GCS at admission (p 0,000 each one), length of stay (LOS)-ICU and days of mechanical ventilation (p 0,000). The BIB and IM related with a worse mRS were DD at 1, 2 3^rd^ day (p 0,016, 0,002, 0,000 respectively), S-100B 1^st^ and 2^nd^ day (p 0,013 and 0,003) and CRP 1, 2 and 3^rd^ day (p 0,039, 0,001, 0,002); and with a worse GOS were DD 2 and 3^rd^ day (p 0,015 and 0,002), S-100B and CRP at 2^nd^ day (p 0,04 and 0,009). Otherwise, mortality was associated with BNP at admission, 1, 2, 3 and 7^th^ day (p 0.005, 0.006, 0.022, 0.032 and 0.010), DD 1, 2, 3 and 7^th^ day (p 0.037, 0.001, 0.000 and 0.001), CRP 2^nd^ day (p 0.004), QI at admission (p 0.004), albumin at admission and 7^th^ day (p 0.013 and 0.037), prealbumin at admission and 7^th^ day (p 0.007 and 0.014). After the multivariable analysis obtained the QI at admission as an independent factor with mortality OR 0.978 (95 % CI 0.962 to 0.994; p 0.009). We performed a ROC curve for QI with AUC 0.668 (p 0.003) an a cutoff point 80.50 %.

**Conclusions:** BIB as S-100B and DD (1, 2 and 3rd day) and CRP (first 72 hours) in an early stage were related to worse outcome at ICU discharge. QI at admission with cutoff point 80.50 % was associated with lower risk of mortality.

**Reference**

1. Potential Role of Blood Biomarkers in the Management of Nontraumatic Intracerebral Hemorrhage. Senn et al.Cerebrovasc Dis 2014;38:395–409

#### A715 Validation of copeptin as a prognostic marker in hemorrhagic and ischemic strokes

##### S. El Maraghi^1^, A. Yehia^2^, M. Bakry^1^, A. Shoman^3^

###### ^1^Faculty of Medicine - Beni Suef University, Critical Care Department, Cairo, Egypt; ^2^Faculty of Medicine Cairo University, Critical Care Department, Cairo, Egypt; ^3^Fever Hospital, Critical Care Department, Shebin Elkom, Menoufia, Egypt

####### **Correspondence:** S. El Maraghi - Faculty of Medicine - Beni Suef University, Critical Care Department, Cairo, Egypt

**Introduction:** Acute stroke is the second leading cause of death and disability in western world^1^**.** An early risk assessment with estimate of the severity of disease and prognosis is pivotal for optimized care and allocation of healthcare resources**.** Novel biomarkers have emerged to assist clinicians with decision making. Copeptin is a novel neuroendocrine peptide that is a strong and independent prognostic marker for functional outcome and death in patients with acute stroke^3^.

**Objectives:** To validate the usage of a single Copeptin value on admission as a short-term prognostic marker in patients with acute stroke.

**Methods:**Forty patients admitted to the Critical Care Department with acute ischemic or hemorrhagic stroke according to the AHA/ASA criteria^2^ were included in the study during the period from September 2013 to September 2014 together with ten volunteers who served as controls. Plasma Copeptin level was measured within 72 hours from onset of symptoms by ELISA technique. Copeptin level was correlated with APACHE II score, GCS, Hemphill score, ASPECTS score and Modifiesd Rankin scale.

**Results:** Copeptin level was statistically higher in studied group than in controls (140.98 ± 101.7 vs. 8.29 ± 2.1 with a p-value < 0.001). There was a statistically significant difference of Copeptin level between patients with recurrent stroke and those with no previous history of strokes (168.2 ± 21.83 vs. 84.53 ± 20.33; with a p- value of 0.035). Copeptin level was numerically higher in hemorrhagic stroke (150.4 ± 98.3) than in ischemic stroke (131.6 ± 50.7) although it didn't reach any statistical significant difference (p-value 0.73). Copeptin level was higher in convulsive patients than in non-convulsive patients (151.13 ± 78.3 vs. 69.96 ± 14.7; with a p value of 0.007). It was found that Copeptin level possess a positive linear correlation with APACHE II score, Hemphill score and Modified Rankin scale with a p-value of 0.011, 0.000 and < 0.001 respectively while it was negatively correlated with ASPECTS score and GCS with a p-value 0.001 and 0.005 respectively. Copeptin level was higher in survivors than in non-survivors (102.89 ± 30.8 vs. 255.24 ± 117.8 pmol/L with a p value 0.011. The optimal cut off value for copeptin as a prognostic marker was 54.5 pmol/l with a sensitivity of 100 % and a specificity of 97 %.

**Conclusions:** Copeptin could be a novel biomarker that correlates with poor outcome, dependence, disability and death in patients with cerebrovascular stroke.

**References**

1. **Donnan GA, Fisher M, Macleod M; et al. (2008):** Seminar: Stroke. The Lancet. 371:1612–23.

2. **An Updated Definition of Stroke for the 21st Century**: A Statement for Healthcare Professionals From the American Heart Association/American Stroke Association: *Stroke.* 2013;44:2064–2089;

3. **Katan M, Fluri F, Morgenthaler NG; et al. (2009):** Copeptin: a novel independent prognostic marker in patients with ischemic stroke. Ann Neurol 66:799–808.

#### A716 Blood biomarkers for acute stroke prognostics

##### F.N. Backes^1^, M.M. Bianchin^2^, S.R.R. Vieira^3^, A. de Souza^4^, A.N. Backes^4^, C. Klein^4^

###### ^1^Hospital de Clínicas de Porto Alegre, Intensive Care Medicine, Neurology, Porto Alegre, Brazil; ^2^Hospital de Clínicas de Porto Alegre, Neurology, Porto Alegre, Brazil; ^3^Hospital de Clínicas de Porto Alegre, Intensive Care Medicine, Porto Alegre, Brazil; ^4^Hospital de Clínicas de Porto Alegre, Porto Alegre, Brazil

####### **Correspondence:** F.N. Backes - Hospital de Clínicas de Porto Alegre, Intensive Care Medicine, Neurology, Porto Alegre, Brazil

**Background and purpose:** Stroke is an important cause of death worldwide, and the majority of stroke survivors suffer from some form of residual disability. This study aimed to investigate the association of blood biomarkers with stroke scales and their predictive value after acute stroke at the time of admission until hospital discharge.

**Design and methods:** We investigated 60 patients with acute stroke who were admitted within 24 h of event onset at the intensive care unit or neurovascular emergency unit of Clínicas Hospital. All patients provided venous blood samples for the measurement of neuron-specific enolase (NSE), S100ß protein (S100ß), interleukin-6 (IL-6), C-reactive protein (CRP) and brain-derived neurotrophic factor (BDNF) within 24 h of the acute event, on the third day and on the fifth day after the stroke. Neurological stroke severity and global disability were determined with the National Institutes of Health Stroke Scale (NIHSS) and modified Rankin Scale (mRS) at the same three times of blood collection and at the time of hospital discharge.

**Results:** The serum levels of the S100ß protein, IL-6 and CRP seem to constitute the best panel of biomarkers after acute stroke in this study. When patients were subdivided into two groups according to the NIHSS (NIHSS ≤ 6 and NIHSS > 6) and mRS (mRS ≤ 3 and mRS > 3) scores, which were used as neurological outcome measures, both neurologic scores for good outcome (NIHSS ≤ 6 and mRS ≤ 3) at hospital discharge were significantly related to the S100ß protein and IL-6 levels at all of the measured time points. Among the analyzed blood markers, S100ß, IL-6 and PCR levels significanttly correlated with the stroke scales and prognostic value.

**Conclusion:** Blood biomarkers may be useful in acute stroke either by suggesting stroke severity or providing a prognostic value. The addition of the S100ß protein, IL-6 and CRP to previously validated stroke scales slightly improves the ability of these scales to predict outcome.

**References**

1. Jickling GC, Sharp FR. Biomarker panels in ischemic stroke. Stroke. 2015 Mar; 46(3): 915–20.

2. Whiteley W, Wardlaw J, Dennis M, Lowe G, Rumley A, Sattar N, et al. The use of blood biomarkers to predict poor outcome after acute transient ischemic attack or ischemic stroke. Stroke. 2012 Jan; 43(1): 86–91.

3. Raman K, Paré G. Of stroke and biomarkers: the elusive quest for a clinical biomarker panel. Clin Biochem. 2013 Jun; 46(9): 705–6.

#### A717 Posterior reversible encephalopathy syndrome (PRES): clinical features and outcomes in ICU patients

##### M.S. Kalaiselvan^1^, M.K. Renuka^2^, A.S. Arunkumar^1^

###### ^1^Sri Ramachandra University, Department of Critical Care Medicine, Chennai, India, ^2^Sri Ramachandra University, Department of Anesthesiology, Chennai, India

####### **Correspondence:** M.S. Kalaiselvan - Sri Ramachandra University, Department of Critical Care Medicine, Chennai, India

**Introduction:** PRES is a clinicoradiological syndrome characterised by transient neurological symptoms with vasogenic edema involving the posterior cerebral region.

**Objectives:** To study the Clinical features and outcomes of patients with PRES admitted to our ICU.

**Methods:** This was a prospective observational study done over period of two years (2014–15). We included all adult patients admitted to our ICU with the following criteria (1) acute onset neurologic symptoms including headache, encephalopathy, seizure, visual disturbance, or focal deficit; (2) focal vasogenic edema on brain imaging and (3) clinical proof of reversibility. Data was collected on demography, co-existing illness, and admission severity of illness, neurological symptoms, systolic and diastolic blood pressure, treatments initiated and MRI findings. Outcome data collected included, mortality, ICU ALOS, no of ventilator days and neurological disability assessed by modified Rankin scale (MRS).

**Results:** 14 patients were admitted with PRES. 13 patients were females, their mean age was 31.5(**±**8.3) years.

Etiology of PRES include eclampsia (n = 9[64.2 %]), lupus nephritis (n = 3[21.4 %]), chronic kidney disease (n = 1[7.1 %]) and hypertension (n = 1[7.1 %]). Most common presenting symptom was seizure (92.8 %), followed by visual disturbance (42.8 %), headache (42.8 %), encephalopathy (14.2 %) and status epilepticus (14.2 %).

Mean APACHE II & SOFA scores were 8.5(±6.9)&2(±1.7) respectively and GCS on admission was 12.3(±2.9). High blood pressure was seen in 12 patients (85.7 %), their mean systolic and diastolic pressures were 173(±9.1) /110(±5.5) mmhg respectively. MRI showed parieto-occipital region was most commonly involved (92.8 %), followed by frontal lobe (42.8 %).

Blood pressure was controlled with IV antihypertensive agents and magnesium sulphate was given for eclamptic patients. Time taken for control of blood pressure 31.25(±12) hours and time taken for awakening was 1(median) hour (range1-60). 7(50 %) patients required ventilator support of which 3 patients were postoperative caesarean section for eclampsia and 4 patients had neurological deterioration and 3(21.4 %) patients required hemodialysis. ICU ALOS was 4.35(±2.4) days and mean ventilator days was 1.7 ± (2.0) days. One patient [1/14(7.4 %)] died of multiorgan failure and 13 patients were discharged with no residual neurological deficit (Modified Rankin Scale-0).

**Conclusions:** PRES is a potentially reversible disorder with prompt recognition and control of blood pressure.

**References**

1) Fugate JE, Claassen DO, Cloft HJ, Kallmes DF, Kozak OS, Rabinstein AA. Posterior reversible encephalopathy syndrome: associated clinical and radiologic findings. Mayo Clin Proc. 2010;85:427–32.

2) Fugate JE, Rabinstein AA. Posterior reversible encephalopathy syndrome: clinical and radiological manifestations, pathophysiology, and outstanding questions.

Lancet Neurol. 2015:14:914–25.

**Table 72 (abstract A717).** Patient characteristics

Demography	n=14
Age mean (±SD)	31.5(10)
Female	13(92.8)
Male	1(7.14)
Etiology of PRES	n=14(%)
Eclampsia	9(64.2)
Lupus nephritis	3(21.4)
Chronic kidney disease	1(7.1)
Hypertension	1(7.1)

**Fig. 98 (abstract A717).**
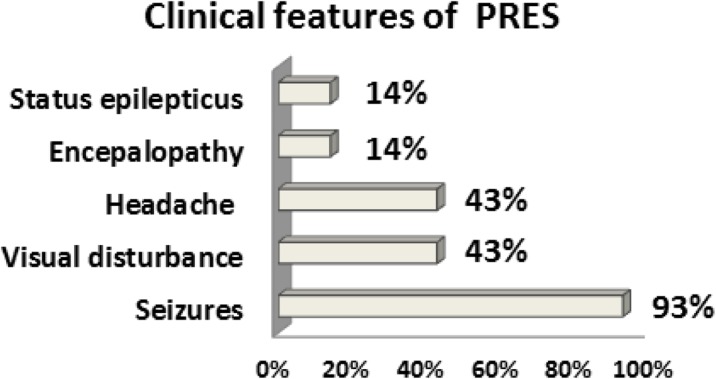
Clinical presentation of PRES

**Table 73 (abstract A717).** MRI findings in PRES(site of lesion)

Site of lesion	n(%)
Parieto-occipital lobes	13(92.8)
Frontal lobe	6(42.8)
Cerebellum	3(21.4)
Brainstem	3(21.4)
Deep white matter.(internal/external capsule)	3(21.4)
Temporal lobe	2(14.2)
Cortical	2(14.2)
Basal ganglia	2(14.2)
Focal hyperacute sub-arachnoid /intraparenchymal hemorrhage	2(14.2)

**Fig. 99 (abstract A717).**
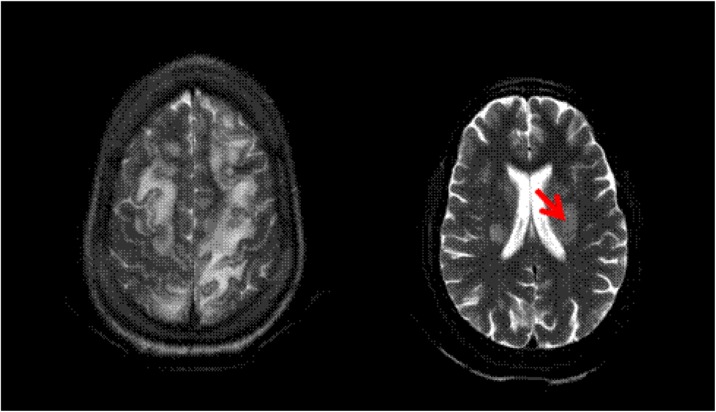
MRI - sites of lesion in PRES

**Fig. 100 (abstract A717).**
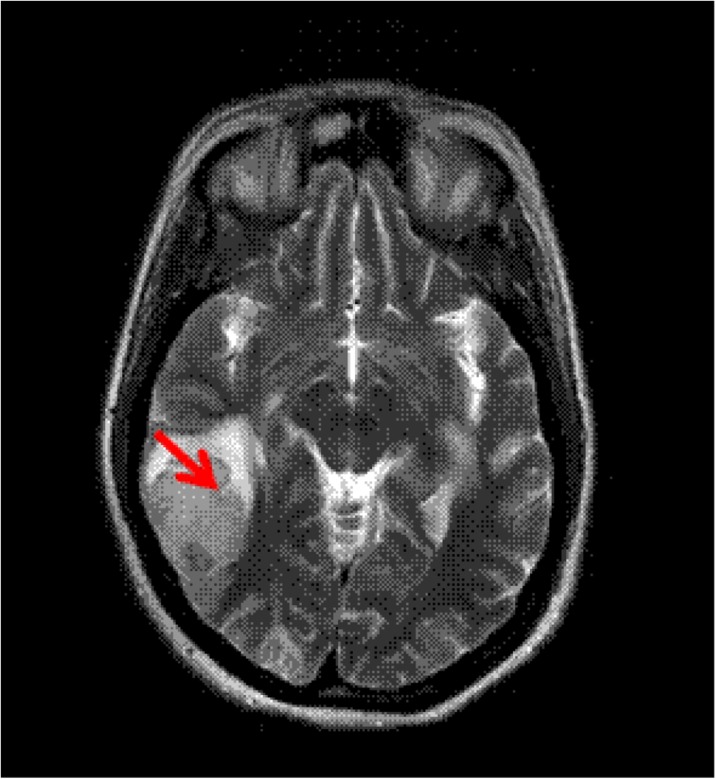
MRI- PRES with intraparenchymal hemorrhage

#### A718 Cerebrospinal fluid glucose and lactate concentrations after traumatic brain injury

##### A. Lozano^1^, O. Lheureux^1^, R. Badenes^2^, J.-L. Vincent^1^, J. Creteur^1^, F.S. Taccone^1^

###### ^1^Erasme Hospital, Intensive Care, Brussels, Belgium; ^2^Hospital Clínico de Valencia, Anesthesia and Intensive Care, Valencia, Spain

####### **Correspondence:** A. Lozano - Erasme Hospital, Intensive Care, Brussels, Belgium

**Introduction:** In patients with traumatic brain injury (TBI), monitoring of brain metabolism using micro dialysis revealed that a decrease in brain glucose concentrations and an increase in lactate levels were markers of cellular alterations. However, such catheters are not used routinely. Few data are available on glucose and lactate concentrations in the cerebrospinal fluid (CSF) of these patients.

**Objectives:** Determine the relationship between glucose and lactate concentrations in CSF and arterial blood and the CSF glucose/lactate ratio and its correlation with outcome in severe TBI.

**Methods:** We reviewed data from all patients admitted to a 35-bed medico-surgical ICU following TBI over a 4-year period (2011–2014). Inclusion criteria were: a) age >18 years; b) presence of an external ventricular drain (EVD) for intracranial pressure monitoring; c) daily analysis of CSF including glucose concentrations for at least 4 consecutive days; d) concomitant measurements of blood glucose/lactate concentrations. Demographics and clinical characteristics were recorded on admission as was the need for continuous insulin therapy (IT; target glucose levels: 110–150 mg/dL) and 3-month neurological outcome (unfavourable outcome was defined as an extended Glasgow Outcome Scale of 1–4).

**Results:** Of 151 patients TBI patients who needed an EVD, 56 met the inclusion criteria (median age: 37 [26–59] years; male: 40). Most EVDs were placed on the day of ICU admission and they were maintained for 10 [6–14] days. Median Glasgow Coma Scale (GCS) on admission was 7 [3–10] and most initial CT-scans were classified as V or VI in the Marshall score (54/57). Twenty (36 %) patients died during the ICU stay and 23 (41 %) had a favourable neurological outcome. Patients who received IT (n = 47) on the first day of EVD monitoring had similar blood and CSF glucose concentrations than those who did not (n = 9). CSF and blood glucose levels were similar in survivors and non-survivors, but non-survivors had higher CSF lactate levels (4.2 [2.9-6.0] vs. 2.4 [2.0-3.7] mmol/L; p = 0.003) and blood lactate levels (1.8 [1.1-2.8] vs. 1.1 [0.8-1.5] mmol/L; p = 0.03) on the first CSF analysis. The CSF glucose/lactate ratio was lower in the non-survivors than in the survivors (1.0 [0.8-1.7] vs. 1.9 [1.4-2.4]; p =0.008). Similar results were found when patients with an unfavourable neurological outcome were compared with the other patients.

**Conclusions:** Non-surviving patients after TBI have initially higher CSF lactate levels and a lower CSF glucose/lactate ratio than survivors. These observations suggest a more profound metabolic brain distress in the non-survivors, who may benefit from an alternative energetic substrate to glucose.

#### A719 In patients with acute spontaneous subarachnoid haemorrhage admitted to the intensive care unit, what proportion require vasopressor and/or inotropic support, and what proportion require central venous catheterization?

##### C. Gallaher^1^, S. Cattlin^2^, S. Gordon^2^, J. Picard^1^

###### ^1^Imperial College Healthcare NHS Trust, Anaesthesia, London, UK; ^2^Imperial College Healthcare NHS Trust, Critical Care, London, UK

####### **Correspondence:** C. Gallaher - Imperial College Healthcare NHS Trust, Anaesthesia, London, UK

**Introduction:** To prevent delayed cerebal ischaemia (DCI), patients with spontaneous SAH (s-SAH) are frequently given supra-normal blood pressure targets.^1^ If patients with DCI do not improve with hypertensive therapy (achieved using fluids and vasopressor drugs, e.g. noradrenaline), a trial of inotropic therapy (e.g. dobutamine) may be considered.^2^ Consequently, vasopressor and/or inotropic therapy is frequently required in s-SAH patients. Central venous catheters (CVCs) are required for the safe administration of many vasopressors and inotropes,^3,4^ but carry potentially serious morbidity and mortality risks,^5,6^ particularly as the femoral insertion site is frequently chosen to avoid the theoretical risk of impaired venous drainage from the head in association with internal jugular vein CVC placement.^7^ The authors observed that there was a difference in practice between consultants within the Department of Anaesthesia regarding the routine placement of CVCs in patients with s-SAH, prompting the study.

**Objectives:** The purpose of the study was to investgate what proportion of patients admitted to ICU with s-SAH required vasopressor and/or inotropic support, what proportion required CVCs, and to establish guidelines as to which s-SAH patients should have CVCs inserted routinely.

**Methods:** All consecutive patients admitted to Charing Cross Hospital ICU with s-SAH for a 1-year period were included retrospectively (n = 70).

**Results:** Forty patients (57.1 %) had vasopressors and/or inotropes administered. Duration of therapy was >24 hours in 38/40 (95 %); median duration was 5.5 days (IQR 6.25 [range 1–22]). Of 44 patients admitted to ICU with GCS ≥13 (NB World Federation of Neurological Surgeons (WFNS) grade 3 SAH = GCS 13–14, with motor deficit), 17 (38.6 %) had vasopressors/inotropes administered, and 10 (22.7 %) had a CVC inserted. Of 26 patients admitted to ICU with GCS ≤12 (NB WFNS grade 4 SAH = GCS 7–12, with or without motor deficit), 23 (88.5 %) had vasopressors/inotropes administered, and 23 (88.5 %) had a CVC inserted.

**Conclusions:** Central venous catheterization carries significant morbidity and mortality risks, and cannot be recommended routinely in all patients admitted to ICU with s-SAH. However, pateints with WFNS grade 4 or 5 SAH (equivalent to GCS ≤12) almost invariably (in 88.5 % of cases) require vasopressors/inotropes and central venous catheterization. CVC insertion should, therefore, form part of the routine anaesthetic care of patients with s-SAH who have GCS ≤12.

**References**

1. Suarez JL. Diagnosis and Management of Subarachnoid Hemorrhage. *Neurocritical Care* 2015; 5: 1263–87.

2. Diringer MN, Bleck TP, Hemphill III JC, et al. Critical Care Management of Patients Following Aneurysmal Subarachnoid Hemorrhage: Recommendations from the Neurocritical Care Society's Multidisciplinary Consensus Conference. *Neurocritical Care*, 2011; 15: 211–240.

3. -7. See full text version

#### A720 The prognostic value of red cell distribution width after subarachnoid haemorrhage

##### V. Fontana, O. Bond, L. Nobile, J.-L. Vincent, J. Creteur, F.S. Taccone

###### Erasme University Hospital, Université Libre de Bruxelles, Intensive Care, Brussels, Belgium

####### **Correspondence:** V. Fontana - Erasme University Hospital, Université Libre de Bruxelles, Intensive Care, Brussels, Belgium

**Introduction:** Red cell distribution width (RDW) is a quantitative measure of variability in the size of erythrocytes and it is used for the differential diagnosis of anaemia. High RDW has been associated with increased hospital mortality in some patients, but this has not been studied in subarachnoid haemorrhage (SAH).

**Objectives:** To investigate whether RDW is associated with outcome after SAH.

**Methods:** We reviewed our institutional database of all adult patients with non-traumatic SAH (2013–2015). We collected demographics, SAH-related data and results of routine daily blood analyses. The RDW (normal values: 10.9-13.4 %), was obtained from the day of admission to day 3. We recorded ICU mortality and long-term neurological outcome; a Glasgow Outcome scale of 4–5 at 3-months was used to define a good neurological outcome (GNO).

**Results:** A total of 174 patients (age 55 [45–66] years; male 76/174) were studied with a median Glasgow Coma Score of 14 [5–15] and a Fisher scale of 4 [3–4]. Sixty-three patients (36 %) developed delayed cerebral ischemia (DCI); overall ICU mortality was 56/174 (32 %) and GNO and 101 patients (58 %) had a GNO. The median RDW on admission was 13.9 [13.3-14.7]% and the highest value during the study period was 14.3 [13.6-15.1]%. A total of 115 patients (66 %) had a high RDW on admission. The RDW was significantly higher on admission in patients who developed DCI than in the other patients (14.0 [14.5-14.9]% vs. 13.6 [13.1-14.3]%, p < 0.001), in non-survivors than in survivors (14.4 [13.9-14.9]% vs. 13.7 [13.1-14.5]%, p < 0.001) and in patients with poor neurological outcome than in those with GNO (14.5 [13.9-14.9]% vs. 13.6 [13.1-14.2]%, p < 0.001).

**Conclusions:** RDW is higher in patients with a poor outcome after SAH. It could be used to better stratify the severity of these patients on admission.

#### A721 Are there cerebral biomarkers for secondary brain damages after traumatic brain injury? Experimental study in rat model

##### S. Mrozek^1,2^, L. Delamarre^1,2^, F. Capilla^3^, T. Al-Saati^3^, O. Fourcade^1,2^, T. Geeraerts^1,2^

###### ^1^University Hospital of Toulouse, Department of Anaesthesia and Intensive Care, Toulouse, France; ^2^University Toulouse 3 Paul Sabatier, Equipe “Modélisation de l'agression traumatique”, Toulouse, France; ^3^University Paul Sabatier, Unité INSERM/UPS US006, Toulouse, France

####### **Correspondence:** S. Mrozek - University Toulouse 3 Paul Sabatier, Equipe “Modélisation de l'agression traumatique”, Toulouse, France

**Introduction:** Secondary insults such as hypoxia-hypotension (HH) provide secondary brain damages and worsen neurologic outcome in patients with severe traumatic brain injury (TBI). Early identification of secondary insults with biomarkers could improve the characterization of TBI severity to better individualize treatment. Several biomarkers have been investigated in severe TBI; However, they have been poorly studied after secondary brain insults. Two of them appear to be particularly of interest^1^: *Glial fibrillary acidic protein* (GFAP) and *Ubiquitin carboxy-terminal hydrolase L1* (UCH-L1).

**Objective:** Our objective was to describe the effects of a standardized secondary insult including hypoxia and hypotension on GFAP and UCH-L1 expression in rat brain in a model of severe diffuse TBI.

**Methods:** Rats were randomly allocated to the following 4 groups: *Control (C)*, in which the entire procedure was performed except TBI and HH; *TBI*, in which TBI was performed alone; *HH*, in which HH phase was performed alone; *TBI + HH*, in which TBI was followed by HH. The TBI model was realized according to the adapted model of Marmarou and Foda reproducing diffuse TBI with acceleration-deceleration damage. Hypoxia was performed by mechanical ventilation with O_2_/N_2_ mixture of 10 %/90 % (decrease in arterial PaO_2_ to about 40 mmHg). Hypotension was obtained with a controlled hemorrhagic shock (mean arterial pressure (MAP) at 40 mmHg) during 15 minutes. Brains were collected after 4 hours of mechanical ventilation for immunohistochemistry of GFAP and UCH-L1 in 6 regions known as particular sensitive to hypoxic insults^2^ (neocortex, striatum, thalamus, hippocampus (CA-1 and dentate gyrus) and corpus callosum). Non-parametric data were compared using Kruskall-Wallis test (p < 0.05).

**Fig. 101 (abstract A721).**
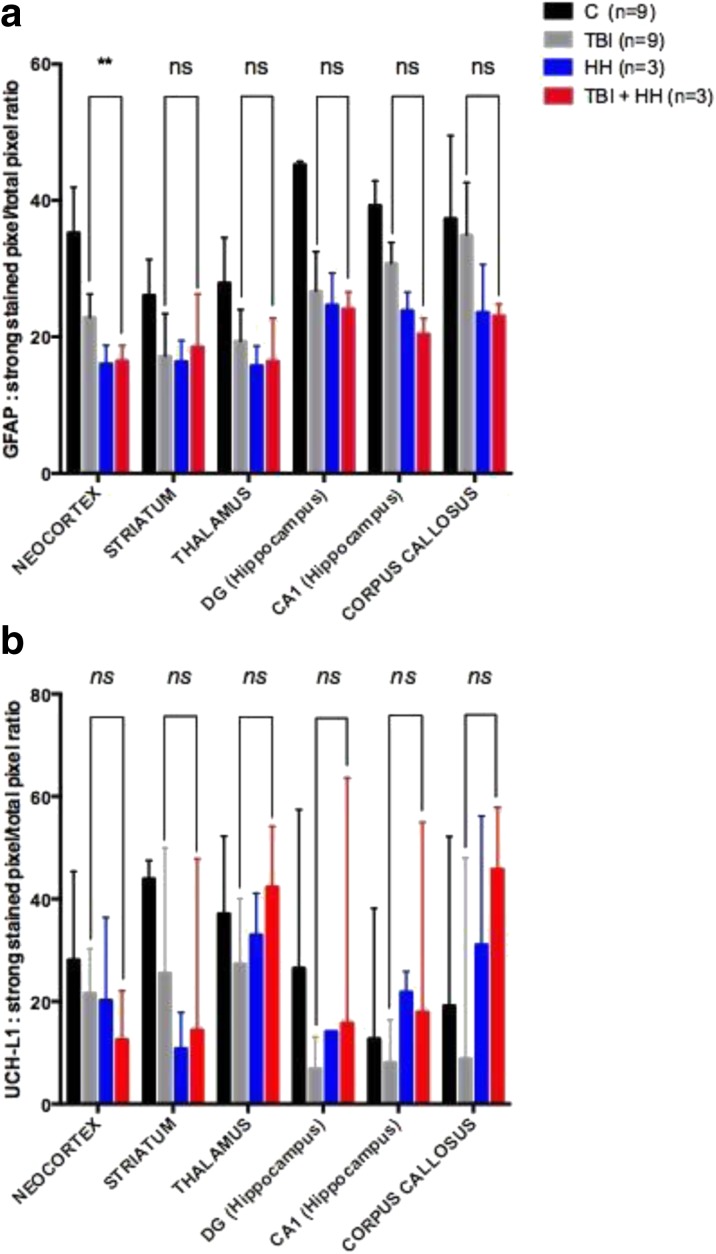
GFAP and UCH-L1 immunostaining in brain tissue according to cerebral regions. A: strong stained pixel/total pixel ratio for GFAP. B: strong stained pixel/ total pixel ratio for UCH-L1. ns: non significant, **: p<0,001. C: control group, TBI: traumatic brain injury group, HH: hypoxia/hypotension group, TBI+HH: traumatic brain injury and hypoxia/hypotension group

**Results:** Twenty-four rats were included (C, n = 9; TBI, n = 9; HH, n = 3; TBI + HH, n = 3). For GFAP, immunostaining revealed a decrease of strong staining (p < 0.001) in the neocortex for TBI + HH group compared to TBI group (Fig. 101). A similar trend was observed in the hippocampus without reaching statistical significance. In all regions, strong staining trended to decrease in TBI group compared to control group. For UCH-L1, strong staining tended to decrease for TBI + HH group compared to TBI group in the neocortex (NS). Moreover, a trend of strong staining decrease in all regions for TBI group compared to control group.

**Conclusion:** These preliminary results suggest that GFAP and UCH-L1 could be early markers of secondary insults reflecting glial and neuronal damages after severe TBI. Measurement of GFAP and UCH-L1 in plasma and cerebrospinal fluid are in progress.

**References**

1. *J Neurotrauma* 2015; 32(16) : 1179–89

2*. J Neurotrauma* 2012; 29(18) : 2782–90

**GRANT ACKNOWLEDGMENT**

La Fondation des gueules cassées

#### A722 Short and mid-term prognostic relevance of brain hypoxia (low PbtO2)

##### A.M. Dominguez-Berrot^1^, M. Gonzalez-Vaquero^2^, M.E. Vallejo-Pascual^3^

###### ^1^Complejo Asistencial Universitario de Leon, Intensive Care Unit, Leon, Spain; ^2^Hospital Rio Carrion, Intensive Care Unit, Palencia, Spain; ^3^Universidad de Leon, Economia y Estadistica, Leon, Spain

####### **Correspondence:** A.M. Dominguez-Berrot - Complejo Asistencial Universitario de Leon, Intensive Care Unit, Leon, Spain

**Introduction:** Intracranial pressure (ICP) monitoring (*MNT*) is widely used in intensive care, due to its therapeutic and prognostic implications. PbtO2 *MNT* is not that frequent, despite studies supporting its use. Given the link between hypoxia and a bad outcome, we accept that correcting brain hypoxia may improve the final result.

**Objectives:** To identify to what extent survival and level of dependence (analysed by GOS at 6 months, GOS-6) of neurocritical patients (NCP) are affected by the time under certain levels of PbtO2, with simultaneous normal ICP value.

**Methods:** Retrospective study. NCP admitted to our ICU (2011–15) underwent ICP + PbtO2 *MNT* . Registered data, among others, were: PbtO2, ICP (hourly), PaO2/8 h, GCS, GOS-6. NCP were divided into 3 groups (**gr**), depending on GOS-6: **gr** 0 (GOS-6 = 1: death); **gr** 1 (GOS-6: 2 & 3, highly dependent); **gr** 2 (GOS-6: 4 & 5; moderately/not dependent). 21 variables (VAR) were analysed, 5 of which reached statistical relevance. We focused on the following items: T1: Time (h) with ICP > 20; T2: Time (h) with PbtO2 < 5; and combined VAR: T10: Time (h) with ICP < 20 + PbtO2 < 10; T15: Time (h) with ICP < 20 + PbtO2 < 15; T20: Time (h) with ICP < 20 + PbtO2 < 20.

STATISTYCAL ANALYSIS. We conducted the study in two phases. FIRST: we compared NCP who survived with those who did not, focusing on critical values of PbtO2 and ICP. SECOND: we analysed differences between **gr** 1 and 2 for variables T10, T15 and T20. After obtaining data descriptive, for each one of them, we applied parametric statistic tests. The arithmetic mean was the starting point. To compare means we used t Student and Welch robust test. Then we conducted a logistic regression analysis to evaluate to what extent each VAR affects outcome or dependence. For **gr** 2 and 3 we performed Kaplan-Meier analysis, to compare the relationship between outcome and the time that each type of patient required to reach a specified goal of PbtO2 (10, 15 and 20).

**Results:** 49 NCP were included (63.3 % men); mean age: 50 (s.d.14,92). The main results of the 1st phase are shown in Table 74.

The risk of death vs no death increases 5.8 % with every h with high ICP. The risk of death vs no death increases 24 % with every h with PbtO2 < 5 and normal ICP. The results of T10, T15 and T20 VAR are shown in Table 75.

**Fig. 102 (abstract A722).**
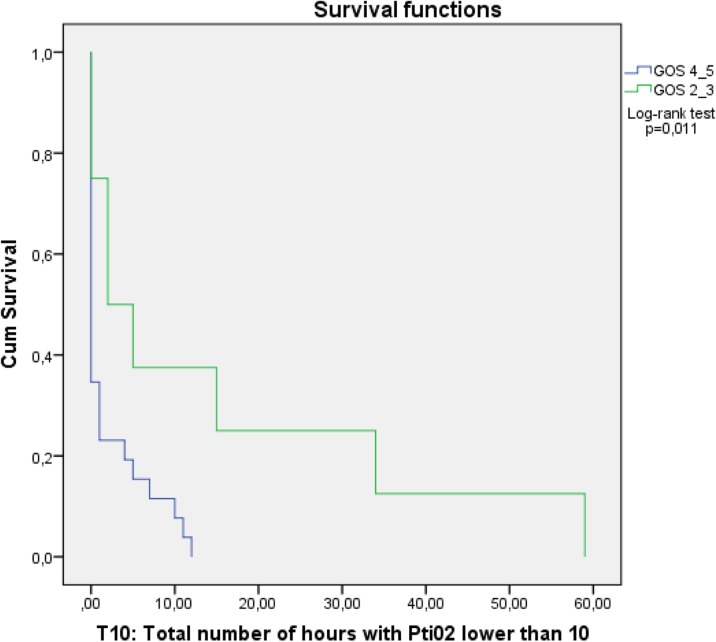
ᅟ

**Fig. 103 (abstract A722).**
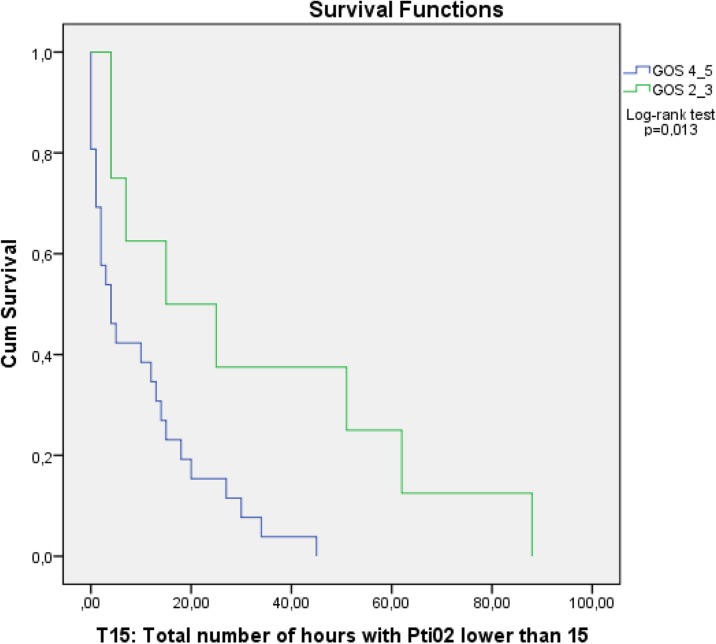
ᅟ

The probability of being highly vs low dependent increases 5.4 % every h with PbtO2 < 15, given normal ICP. For variable T10, differences are nearly but not definitely statistically significant. Finally, for VAR T10 and T15, Kaplan Meier estimator was applied, (results shown in Figs. 102 and 103.

**Conclusions:** Maintained high ICP and low PbtO2 are mortality determinants. Time with low PbtO2 increases the risk of death to a higher extent than time with high ICP. Low PbtO2 is in itself an independent prognostic factor. Even with moderate brain hypoxia, patients may reach an acceptable functional outcome. Not only are PbtO2 values crucial for outcome, but also time spent to achieve an acceptable value.

**Table 74 (abstract A722).** Survivors vs non survivors

	Group 0 (N=15)	Group 1+2 (N=34)	Welch	Logistic Regression	p
	Media	Media	p	Odds Ratio (CI:95%)	
T1	51,4667	14,9706	0,007	1,058 (1,020–1,097	0,003
T2	7,0000	1,4706	0,039	1,239 (1,064–1,443)	0,006

**Table 75 (abstract A722).** Severe vs low disability

	Group 1 (N=8)		Group 2 (N=26)		Welch (p)	Logistic regression	p
	Mean	s.d.	Mean	s.d.		Odds ratio (CI 95%)	
T10	14,6250	21,32696	2,0000	3,76298	0,139	1,134 (0,982–1,309)	0,086
T15	32,0000	31,45972	10,1154	12,31041	0,092	1,054 (1,006–1,105)	0,027
T20	62,6250	51,80165	41,0000	45,49989	0,312 (n.s.)

#### A723 Does cerebral glucose parallel serum glucose in severe TBI subjects: a cerebral microdialysis study from India

##### D. Gupta

###### All India Institute of Medical Sciences, Neurosciences Centre, Delhi, India

**Introduction:** The study hypothesis was hypoglycaemia is an independent outcome prognosticator in severe traumatic brain injured cases.

**Objectives:** The study aimed to see the role of glucose monitoring in the brain parenchyma region as independent outcome prognosticator and its association with plasma glucose . The aim of the study was to analyse the relationship of intracerebral glucose measured by intraparenchymal cerebral microdialysis and it's relationship to blood glucose. We also evaluated the relationship of these values to the outcome.

**Methods:** Prospective nonrandomised study conducted at a tertiary care trauma centre in India.Twenty-five patients with severe TBI who underwent decompressive craniectomy were prospectively monitored with intracerebral microdialysis catheters (MD).

**Results:** Fifteen patients (60 %) had a good outcome in terms of GOS at 3 months while the rest (10 patients) had poor GOS at 3 months. There was significant difference in the incidence of hyperglycemia (RBS > 10 mmol/L) between the two groups (p < 0.0001). The difference between the two groups while comparing episodes of hypoglycemia was significant (p = 0.0026). Good outcome group had fewer episodes of brain hypoglycemia during systemic hypoglycemia (p = 0.0026). Neither mean blood glucose values nor mean cerebral glucose values predicted outcome at 3 months.

**Conclusions:** After decompressive craniectomy in severe TBI, there was a poor correlation between plasma and MD glucose concentration. A high degree of variation was seen in the correlations for patients individually. Neither mean blood glucose values nor mean cerebral glucose values predicted outcome at 3 months. Good outcome group had fewer episodes of both hyperglycemia and hypoglycemia.

**References**

1. Vespa PM, McArthur D, O'Phelan K, Glenn T, Etchepare M, Kelly D, et al. Persistently low extracellular glucose correlates with poor outcome 6 months after human traumatic brain injury despite a lack of increased lactate: a microdialysis study. J Cereb Blood Flow Metab 2003;23:865–877.

2. Schlenk F, Nagel A, Graetz D, Sarrafzadeh AS. Hyperglycemia and cerebral glucose in aneurysmal subarachnoid hemorrhage. Intensive Care Med 2008;34:1200–7.

3. Abate MG, Trivedi M, Fryer TD, Smielewski P, Chatfield DA, Williams GB, et al. Early derangements in oxygen and glucose metabolism following head injury: the ischemic penumbra and pathophysiological heterogeneity. Neurocrit Care 2008;9:319–25.

**Grant acknowledgement**

Non funded institutional study.

### Education, training, staff

#### A724 Starting out: point of care critical care simulation training programme - experience in a UK university hospital intensive care unit

##### B.D. Ivory^1^, M. Chopra^2^

###### ^1^Torbay Hospital, Intensive Care, Torquay, UK; ^2^Derriford Hospital, Intensive Care, Plymouth, UK

####### **Correspondence:** M. Chopra - Derriford Hospital, Intensive Care, Plymouth, UK

**Introduction:** Simulation is increasingly recognised as a useful modality in post-graduate education in Intensive Care Medicine (ICM) but is often perceived as expensive and difficult to deliver. It is used for recruitment to ICM training posts and also forms part of assessment for the Faculty of Intensive Care Medicine exam, however its use in ICM education training lags behind anaesthesia.

**Objectives:** We aimed to establish and evaluate a programme of regular, integrated, curriculum-mapped point-of-care simulation training in a United Kingdom University Hospital Intensive Care Unit.

**Methods:** A 6 month pilot project was undertaken delivering dedicated, weekly inter-professional simulation sessions as part of the existing trainee education programme. This consists of weekly, 2-hour curriculum-mapped small group sessions attended by stage 1, 2 and 3 ICM trainees. During the pilot programme, 1 hour of each session was transferred to a simulation-based session, with scenario objectives designed to complement the teaching topic. At least 1 member of nursing staff was released from clinical duties to participate in the session. Other allied professionals were encouraged to attend. Each session required 1 confederate, 1 debriefer and 1 person to run the manikin. Scenarios were designed to explore both technical and non-technical aspects of the topic and were delivered in an ICU side-room that is permanently set up with a manikin. Debriefing was provided using the ´good judgement´ model. Quantitative and qualitative feedback was sought after each session^1^. After 6 sessions, interim feedback was analysed and changes were implemented for the remaining pilot sessions.

**Results:** Several common themes emerged from the initial feedback. Whilst broadly positive, participants and faculty all stated that the room was very crowded, with many feeling that it detracted from the learning experience. In an attempt to address this, we employed the low-cost iOS application Airbeam®. This allows live audio and video to be streamed over a secure wireless network between iOS devices (iPhones and a MacBook). This allowed observers to view the scenario from an adjacent room.

Final feedback was almost universally positive. Participants reported greater willingness to speak up in future and highlighted specific issues such as the accessibility of emergency guidelines. Quantitative feedback demonstrated statistically significantly higher scores in all areas for simulation versus didactic teaching.

**Conclusions:** We were able to provide a curriculum-mapped, in situ simulation-based training programme within our ICU that was highly rated by participants using low-cost, medium fidelity technology. We present our experiences as potential template for other programmes.

**References**

1. Rudolph, J W, et al., et al. There´s no such thing as a ´non-judgmental´ debriefing: a theory and method for deberiefing with good judgment. 2006, Simul Healthc 2006; 1: 49–55.

**Table 76 (abstract A724).** Comparison of scores awarded for didactic and simu

	Met objectives mean score (IQR)	Relevance mean score (IQR)	Effectiveness of teaching method mean score (IQR)	Standard of teaching mean (IQR)
Tutorial	8.632 (8–9.25)	8.97 (8–10)	8.8 (8–10)	8.995 (8–10)
Simulation	9.161 (9–10)	9.352 (9–10)	9.397 (9–10)	9.353 (9–10)
p value	<0.001	0.004	<0.001	0.004

#### A725 Less than full time training and intensive care medicine: unlikely bedfellows or a match made in heaven?

##### J. McCarthy, C.L. Felderhof, C. MacNeil

###### Queen Elizabeth University Hospital, NHS GG&C, Anaesthetics & ICM, Glasgow, UK

####### **Correspondence:** J. McCarthy - Queen Elizabeth University Hospital, NHS GG&C, Anaesthetics & ICM, Glasgow, UK

**Introduction:** A recent article written by The Faculty of Intensive Care Medicine Workforce Advisory Group (FICM WAG) highlighted the looming staffing crisis for ICM within the UK due to the increasing need for critical care related to the increasing age of the population. The Intensive Care National Audit and Research Centre (ICNARC) have advised FICM WAG to expect a 4 % rise per year in the demand for intensive care beds and the Centre for Workforce Intelligence (CfWI) is predicting a required increase in the number of WTE in anaesthesia and ICM from 6,100 to 11,800 by 2033.^1,2^ This, in conjunction with contract reforms and a change in workforce demographic, means that we need a multifaceted approach to increasing the number of intensivists over the coming years.

**Objectives:** Less than full time (LTFT) trainees are still very few in number within ICM and we wanted to assess how the experiences LTFT anaesthetic trainees had within the ICU setting affected their future career choices and whether this group could be a source of increasing the number of applicants to ICM dual training.

**Methods:** We used an anonymous web based survey distributed nationwide to LTFT anaesthetic trainees.

**Results:** We received 115 responses. The vast majority were LTFT due to childcare responsibilities (96.4 %). 7.2 % had been asked to complete their ICM training full time despite approval for LTFT training from their parent specialty. 40 % had considered dual training with ICM but ultimately decided against it. Only 3.5 % were planning to apply in the future. 78.3 % had encountered difficulties with ICM training related to their LTFT status. The most common issues were engaging in extra-curricular activities, negative attitudes to LTFT training and working patterns. 45.2 % felt they were perceived more negatively than their FT peers. 53.1 % felt they would consider applying for dual training if there was a more structured pathway for LTFT trainees. LTFT trainees expressed a wish to connect with others in order to allow peer support and mentorship.

**Conclusions:** Very low numbers of LTFT anaesthetic trainees are pursuing a career within ICM due to difficulties encountered with their training. Some of the highlighted issues may be due to the nature of ICM but the negative attitudes experienced by LTFTs can and should be addressed and more structured guidance for navigating LTFT training within ICM could be developed. Innovative approaches to allow equity of access to training opportunities would be welcomed. We propose that although ICM and LTFT training have not been the most natural bedfellows, there is a pressing need for this relationship to improve. LTFT trainees may be a relatively untapped resource which could contribute towards the necessitated expansion of the ICM workforce.

**References**

1. Rhodes A. FICM Workforce. Bulletin 95. 2016 Jan; 95: 12

2. Centre for Workforce Intelligence. In-Depth Review of the Anaesthetics and ICM Workforce - Final Report. 2015 Feb

#### A726 Factors associated with success in new format of oral part of European diploma of intensive care medicine (EDIC)

##### F. Rubulotta^1^, P. Waldauf^2^, M. Maggiorini^3,4^, F. Duska^2,4^, on behalf of Department of Professional Development, ESICM

###### ^1^Imperial College London, London, UK; ^2^3rd School of Medicine, Charles University in Prague, Anaesthesia and Critical Care, Prague, Czech Republic; ^3^UniversitätsSpital Zürich, Medical ICU, Zürich, Switzerland; ^4^Examinations Committee, ESICM, Brussels, Belgium

####### **Correspondence:** F. Rubulotta - Imperial College London, London, UK

**Introduction:** Oral part of EDIC diploma examinations has changed in 2013 into objective-structured clinical examination (OSCE) type exam [1]. All candidates face identical questions with pre-defined correct answers simultaneously in 7 high-throughput exam centres.

**Objectives:** To identify factors associated with success in part 2 EDIC exam.

**Methods:** We prospectively collected self-reported data from all candidates sitting EDIC Part 2 in 2015, namely demographics, professional background and attendance to an EDIC part 2 or generic OSCE exam preparatory courses. After testing association with success (with cut-off at p < 0.10) and co-linearity of these factors as independent variables, we performed a multivariate logistical analysis, with binary exam outcome (pass/fail) as the dependent variable. Structural equation modelling was used to gain further insight into relations among determinants of success in oral part of EDIC.

**Results:** Out of 427 candidates sitting the exam we completed data from 341 (80 %). Following candidates' factors were associated with increased chance of success: English as native language (Odds ratio 4.3 [95%CI 1.7; 10.7]), use of Patient-Centered Acute care Training e-learning program module (OR 2.0 [1.2; 3.3]) and working in EU country (OR 2.5 [1.5; 4.3]) and better results in written part of EDIC (for each additional SD of 6.1 points OR 1.9 [1.4; 2.4]). On the contrary, chance of success in EDIC 2 decreased with candidates´age (for each additional SD of 5.5 years OR 0.67 [0.51; 0.87]). Factors eliminated from the model because of lack of association with success or because of co-linearity included attendance in EDIC Part 2 prep courses (OR 0.95 [0.52-1.70]), English as study language, baseline medical specialty, the use of CoBaTriCE and actual working status (working or not). Exam centres could be grouped into 3 groups with similar success rates, and there were significant differences in exam outcomes among these groups even after adjustment to known candidates´ factors (G1 vs G2 OR 2.4 [1.4; 4.1]; G1 vs G3 OR 9.7 [4.0; 23.1]; and G2 vs G3 OR 3.9 [1.7; 9.2]). Short data collection period (only one year) and 20 % of missing candidates' data are the main limitations of this study. The ESICM examination committee and Executive Committee have taken a series of measures in order to optimise fairness and reproducibility of the exam, which will be meticulously monitored in upcoming series of exams.

**Conclusions:** Younger age, English as native language, working at a European country and the use of PACT for preparation were factors associated with success in oral part of EDIC exam. Despite the limitations of this study, the signal of differences in outcomes among the 7 exam centres will need further investigation.

**References**

1. R M Harden et al.: Assessment of clinical competence using objective structured examination. *Br Med J* 1975;1:447

#### A727 Moral distress and its contribution to the development of burnout syndrome among critical care providers

##### R.R.L. Fumis, J.M. Vieira Junior, G. Amarante

###### Hospital Sírio-Libanês, Intensive Care Unit, São Paulo, Brazil

####### **Correspondence:** R.R.L. Fumis - Hospital Sírio-Libanês, Intensive Care Unit, São Paulo, Brazil

**Introduction:** Burnout appears to be common among Critical Care providers. It is characterized by three components: emotional exhaustion or loss of passion for one's work; depersonalization or treating patients as objects and the sense that your work is no longer meaningful. Moral distress is the inability of a moral agent to act according to his or her core values and perceived obligations due to internal and external constraints.

**Objectives:** To assess both burnout syndrome and moral distress among all ICU and the Step Down Unit (SDU) providers (Physicians, Nurses, Nurse Technicians, and Respiratory Therapists) and to estimate the correlation between moral distress and burnout.

**Methods:** For data collection, a self-administered questionnaire for each critical care provider was used including basic demographic data, the Maslach Burnout Inventory (MBI) and the Moral Distress Scale (MDS).

**Results:** A total of 283 of the 389 (72.7 %) Critical Care providers (Nurses, Nurses Technicians and Respiratory Therapists), agreed to participate and the survey occurred from August and September 2015. Out of this total, 134 were from ICU and 116 from SDU. Regarding Physicians, the same team of intensivists attended both ICU and SDU and they were treated as an individual group and 67.3 % of them participated. Severe Burnout was identified in 18.2 % of Physicians. Considering all others Critical care providers (ICU + SDU) we identified that 22.5 % (95 % CI: 17.7-27.8 %) presented severe Burnout. There was not statistical difference between the units: 22.1 % of ICU respondents had severe Burnout and 23.7 % from SDU,p = 0.774. A total of 68 questionnaires of Moral Distress were incomplete and were excluded from analysis. All respondents (n = 215) reported a high level of moral distress overall. The mean MDS rate for all ICU respondents including both domains (frequency and intensity) was 111.5 and for all SDU respondents was 104.5, p = 0.446. We observed that those respondents with high MDS score (>100 points) were more likely to suffer from Burnout (28.9 % x 14.4 %, p = 0.010). Moreover, many questions from the MDS questionnaire were significantly associated with severe burnout.

**Conclusions:** Our data show that moral distress, resulting from therapeutic obstinacy and the provision of futile care was the great problem of the critical care providers' team and significantly associated with severe burnout.

**References**

1. Abbasi M, Nejadsarvari N, Kiani M, et al. Moral distress in physicians practicing in hospitals affiliated to medical sciences universities. Iran Red Crescent Med J. 2014;16(10):e18797.

2. Embriaco N, Papazian L, Kentish-Barnes N, Pochard F, Azoulay E. Burnout syndrome among critical care healthcare workers. Curr Opin Crit Care. 2007;13(5):482–8.

3. Rushton CH, Batcheller J, Schroeder K, Donohue P. Burnout and Resilience Among Nurses Practicing in High-Intensity Settings. Am J Crit Care. 2015 Sep;24(5):412–20.

#### A728 Short training intervention improves familiarity with Sengstaken-Blakemore tube management

##### A. Skorko, S. Sanders

###### Bristol Royal Infirmary, Bristol, UK

####### **Correspondence:** A. Skorko - Bristol Royal Infirmary, Bristol, UK

As the United Kingdom intensive care training curriculum develops and the role of the intensive care physician becomes more defined so procedural skills associated with this role will become refined. Some skills such as intubation and central access are well recognised. However, occasionally less common skills may be required and these deserve a place in the curriculum. One such skill is that of Sengstaken-Blakemore tube (SBT) insertion. Currently the curriculum contains domain 5.18: 'Describes Sengstaken tube (or equivalent) placement'.

A trainee survey was undertaken to quantify the degree of familiarity with this clinical tool in the Severn deanery in the South West of the UK. A mixed cohort of 24 trainees responded (17 intensive care, 7 anaesthetic trainees). Of those surveyed 3 had inserted SBT, 21 had not. 61 % had some previous experience with SBT ('seen one inserted by someone' or 'looked after a patient with an SBT in situ'). Little formal teaching existed on the topic with 57 % having received no training. 75 % felt they would benefit from a formal teaching session.

This survey demonstrated a gap in confidence and exposure to SBT use. As a result, a short teaching intervention was performed during a regional training day. This focused on the common cause of GI bleeds and situations where SBT may be of benefit, as well as practical aspects of insertion, along a low fidelity model demonstration.

**Fig. 104 (abstract A728).**
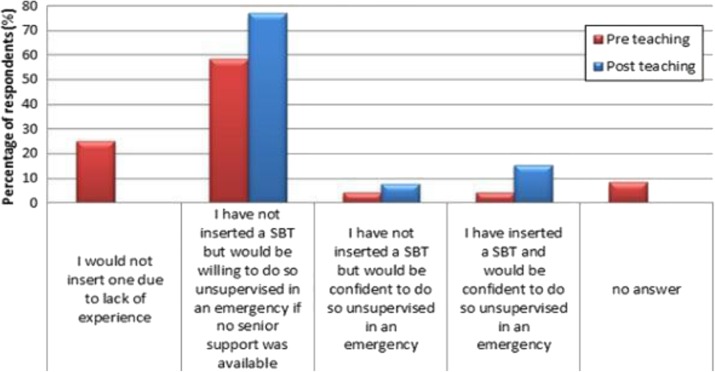
Pre versus post intervention confidence

After this session a repeat questionnaire was sent out. This demonstrated that confidence was improved (Fig. 104), with 61 % feeling that annual training would be of benefit.

**Summary:** As the Intensive Care curriculum continues to develops, trainees feel they would benefit from regular training sessions in less common skills such as the management of Sengstaken-Blakemore tubes. More broadly, involving trainees in the development of the curriculum will ensure it meets the learning needs of the future work force and guarantee high quality patient care.

#### A729 Creation of a critical care training program for midwives: part of a quality improvement project to create a maternal high dependency environment

##### J. Aron, R.J. Kroll, C. Redfearn, P. Krishnan

###### Medway Maritime Hospital, Anaesthesia and Intensive Care, Gillingham, UK

####### **Correspondence:** J. Aron - Medway Maritime Hospital, Anaesthesia and Intensive Care, Gillingham, UK

**Introduction:** National guidelines state that critically ill women should receive the same standard of care for their pregnancy-related and critical care needs. With changes to midwife training focusing on normal labour and delivery, nursing competences are reduced. A recent national audit identified that 43 % staff in maternity high dependency unit (HDU) areas had no specific critical care training (1).

**Objectives:**

- To identify the need to cerate a HDU on the labour ward to meet these national standards through local audit.

- Development of a training course for midwives.

**Methods:** A retrospective audit including all cases admitted to HDU over a one-year period was conducted. Information regarding demographics, diagnosis, maternal and neonatal morbidity and mortality and length of critical care stay was collected. Secondly, a prospective audit over a 2-week period surveyed all obstetric in-patients including demographics, physiology scoring, level of care required and staff providing this care.

**Results:** The retrospective phase identified 18 patients transferred to HDU, all post-partum. 6 of these patients could have been accommodated on a HDU on labour ward, had one existed.

The prospective phase identified 17 women required HDU according to physiology scoring. 88 % of these women were cared for by Band 6/7 midwives, and the remainder by band 5 midwives. None of the midwives had recent critical care training. Only 26 % of patients were reviewed by an anaesthetist and 37 % by a consultant obstetrician. 42 % did not receive the correct review prompted by their MMEOWS score.

**MECU development:** A 2-day course was created using a mixed-methods teaching program including theoretical, practical and simulation based sessions. The curriculum is designed to improve midwives´ confidence and skills in detecting, treating and stabilizing emerging or established critical illness.

The midwives who participated in the MMEC course highlighted that the practical elements, demonstrations and simulation-based teaching facilitated their learning were particularly effective. Feedback identified increased confidence in clinical management, increased awareness of when to escalate and recognition of their own limitations.

**Conclusions:** The MECU course, as part of a larger quality improvement project to create a labour ward HDU environment was created in response to local audit. This identified that such a unit could reduce admissions to HDU provide appropriate care for in-patients in need of a higher level of care. The course created has a curriculum designed to increase confidence and awareness of the issues surrounding the sick parturient and has received excellent feedback. Future plans include development of a regional training network.

**References**

1. What level of critical care are we providing?, NA Williams et al., IJOAA, 2015 24, S42

#### A730 Interhospital transfers in a non-tertiary hospital-does it improve with formal training?

##### J.E. Khalil^1^, F. Kovari^2^

###### ^1^North Middlesex University Hospital, Critical Care, London, UK; ^2^North Middlesex University Hospital, ICU, London, UK

####### **Correspondence:** F. Kovari - North Middlesex University Hospital, ICU, London, UK

The aim of this audit was to demonstrate any significant improvement in our inter-hospital transfer practice following the introduction of a new simulated all day training provided to junior doctors in the view of auditing our transfer data from 2014–2015.

A single centre retrospective audit in 21 bed mixed ICU of a non-tertiary university hospital. During the audit period (2014–15) 29 critical care transfers were made. We audited data of referring team, location, receiving team, transfer reason, decision-departure time, intubation details, arterial blood gases (ABG), comments of the teams, time of transfer, documentation and transferring doctor grade. Our primary aim was to analyse any effect of our newly introduced complex transfer training program.

In 2014, transfers were by SHO level doctors 38.5 % of cases, staff grade and registrar level doctors were 46.1 % each, **15.3 % were not documented**.

In 2015, transfers were by SHO level doctors 6.25 % of cases, staff grade and registrar level doctors were 93.7 % each. **All transfers were documented.**

Non-clinical reasons in 2014 were 24 % opposite zero non clinical transfers in 2015. In 2014 (53.8 %) of transfers occurred outside normal working hours (8 am to 17:00) versus 56.25 % in 2015, unlike the past where most of transfers usually happened outside the normal working hours.

Transfer- decision to departure time varied, with an average time of minutes In 2014 the average time in minutes was 229 minutes which was nearly double its peer value in 2015 **(133 minutes).**

Records of the doctor performing the transfer were well kept but those of the receiving team were generally absent but thanks to the new transfer form 24 out of 29 comments from the receiving team were documented (82.7 %) and were consistent with the expectations with one comment saying (Excellent handover).

The transfer of the critically ill patients remains a big challenge to juniors of all grades. A complex and holistic approach needed with a broad spectrum of knowledge regarding the possible complications.

Our all day transfer course introduced in 2015 gave the opportunity to learn and hands on simulate with a live ambulance what is it like to be on a hot transfer.

Our data shows significant improvement in the documentation, organization and quality of transfer without adverse events affecting patient´s safety with lack of incident reports.

The positive feed backs from trainees and the overall improvement of our transfer service proves that simulated training of this difficult and grey area is fruitful and worth carry on.

**References**

1. Intensive Care Society. Guidelines for transport of the Critically ill adult. London: Intensive Care Society, 2011.

2. Department of Health. Comprehensive Critical Care. A review of adult critical care services. London: Department of Health, 2000.

3. Transferring the critically ill patient: are we there yet?.Joep M, Smit M, Anthony R Jack JM et.al. Crit Care. 2015; 19(1): 62.

**Fig. 105 (abstract A730).**
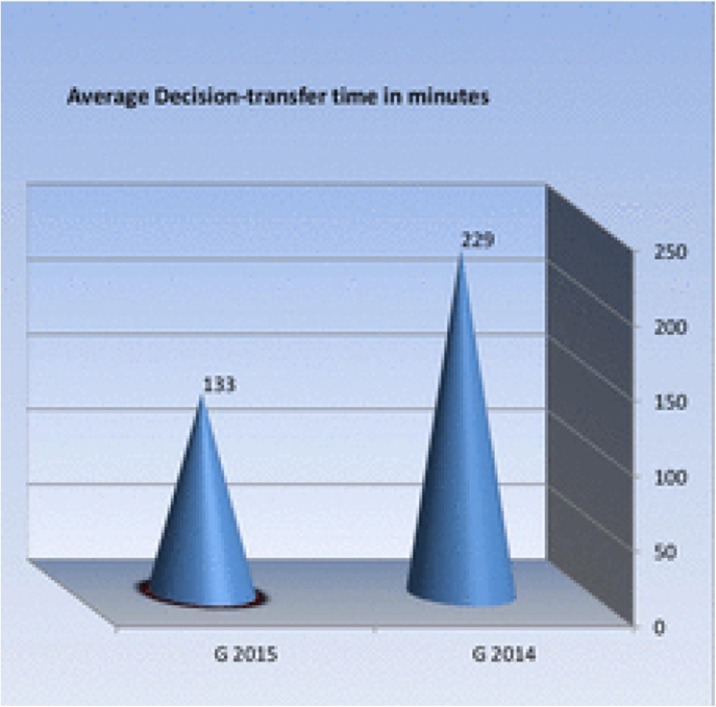
Average decision-transfer time in minutes

**Fig. 106 (abstract A730).**
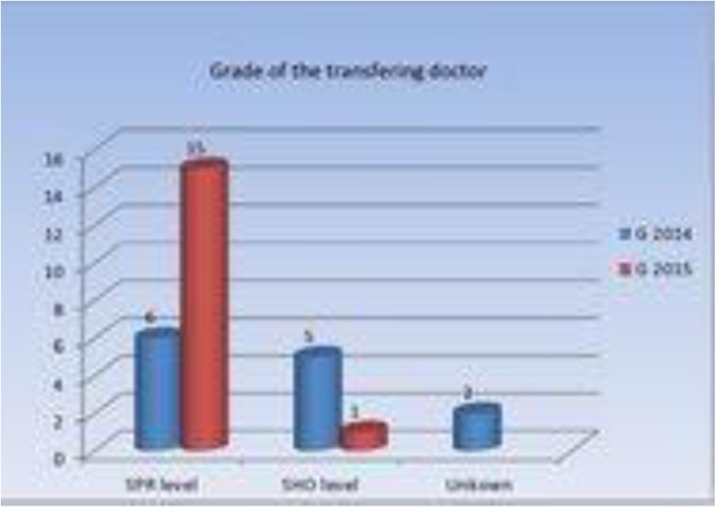
Grade of the transfering doctor

**Fig. 107 (abstract A730).**
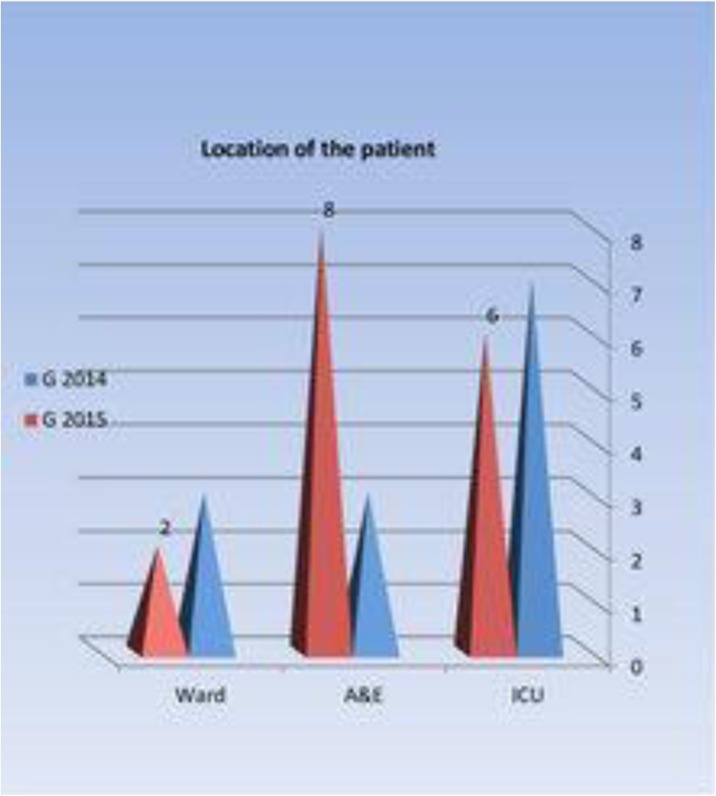
Location of the patient

#### A731 Critical care nurse competence in detecting delirium by the confusion assessment method for the intensive care unit (CAM-ICU)

##### N. Kongpolprom

###### ^1^King Chulalongkorn Memmorial Hospital, Thai Red Cross and Chulalonkorn University, Critical Care and Pulmonary Medicine, Bangkok, Thailand

**Introduction:** Delirium is one of the most common complications resulting in ICU morbidity and mortality. Despite its well-known condition, delirium is frequently unrecognized by critical care teams. Additionally, assessment of delirium in critically ill patients is not routinely performed in our country. The Confusion Assessment Method for the ICU (CAM-ICU)^1,2^ is a well-validated tool for delirium evaluation, however, it is the new tool for our team.

**Objectives:** We aimed to assess nurse competence in using the CAM-ICU for delirium evaluation.

**Methods:** Our hospital had implemented the CAM-ICU for delirium evaluation in 2 medical ICUs for a 4-month period and we subsequently assessed nurse competence in using the CAM-ICU by a paper-based examination. All medical ICU nurses were enrolled to perform the test which was composed of 5 case-scenarios. The nurses were allowed to use CAM-ICU sheets for Richmind Agitation Sedation Scale (RASS) scoring^3^ and delirium evaluation.

**Results:** Sixty-nine medical ICU nurses did the paper-based test. Approximately, 31 % of the examinees scored RASS inaccurately. Interestingly, 60 % of them did not understand how to interpret the mental status domain of the CAM-ICU, especially the fluctuating course of consciousness. In addition, only 29 % of them detected hypoactive delirium correctly. Furthermore, 80 % of the examinees got confused to make a diagnosis of delirium in partially sedated patients.

**Conclusions:** The study demonstrated the limited skill and knowledge of our intensive care nurses in detecting delirium by the CAM-ICU. According to these results, a structural training program would be provided to increase the nurse competence in delirium assessment.

**References**

1. Inouye SK, van Dyck CH, Alessi CA, Balkin S, Siegal AP, Horwitz RI. Clarifying confusion: the confusion assessment method. A new method for detection of delirium. Ann Intern Med.1990;113(12):941–948.

2. Ely EW, Margolin R, Francis J, et al. Evaluation of delirium in critically ill patients: validation of the Confusion Assessment Method for the Intensive Care Unit (CAM-ICU). Crit Care Med. 2001;29(7):1370–1379.

3. Sessler CN, Gosnell MS, Grap MJ, et al. The Richmond Agitation-Sedation Scale: validity and reliability in adult intensive care unit patients. Am J Respir Crit Care Med. 2002;166(10):1338–1344.

#### A732 A systematic review of the impact of simulation in human factors and non-technical skills (NTS) on the safety and quality in intensive care training, based on the lessons learnt from aviation and other high-risk industries

##### V. Gulia

###### Good Hope Hospital, Anaesthesia + Intensive care, Sutton Coldfield, United Kingdom

**Introduction:** In 2007, Martin Bromiley established the first clinical human factors group, which has both clinical and NTS specialists involved in safety in intensive care and anaesthesia.

Human factors and NTS contribute up to 40 % of the critical incidents as per the ***NAP 4*** anaesthesia audit which was a UK based national level audit on the adverse incidents in airway management during anaesthesia. The causes for critical incidents included lack of situation awareness, judgement and poor decision making with poor communication and teamwork. A mortality rate of 5.6 per million general anaesthetics (1:180 000) in a period of over a year in anaesthesia, ICU and the emergency department, was reported. The project findings suggest avoidable deaths due to airway complications occur in ICU and the emergency department.

**Objectives:** To analyse the rationale of a full-scale simulator called the ***Comprehensive Anesthesia Simulation Environment (CASE*)** to specifically study decision-making processes of anaesthetists during that were a take off from aviation ***Crew Resource Management (CRM***)in difficult Airway & Resuscitation situation.

Critical analysis of simulation as a valid and reliable tool to assess the ***competency based training (CBT).***

**Methods:** This study conducts a systematic literature search and an analytical review from the Cochrane database of reviews and other online sites such as Medline, Embase and Pubmed.

Results-Simulator enables faculty to provide structured simulation lab experiences instead of trying to find appropriate and/or rare patient care opportunities in a health care settingAny kind of environment can be created to train multi professional teams in primary and secondary care. Currently simulators are used much more for educational purposes (undergraduate: 77 %, postgraduate: 85 %) than for evaluation purposes (undergraduate: 14 %, postgraduate: 7 % (Girard, Ely 2008) and that proportion needs to change with the current needs of using simulation for evaluation of competency.

**Conclusions:** Human factor training develops leadership, coordination and shared understanding of roles leading to improved performance in anaesthesia, trauma and intensive care. *Simulation* is vital to validate ***CBT*** and it can be accepted as a comprehensive way to assess procedural skill. Behaviour and personality testing should be considered in the selection process to identify the prospective trainees in the subject along with the just use of ***NTS training*** to achieve that end. Simulation centres require important financial and human resources and hence the need for communication and collaboration among centres using simulation technology to improve quality of training.

**References**

1. COOK, T.M. et .al NAP 4 *British journal of anaesthesia,***106**(5), pp. 617–631

2. CRABTREE, N.A et.alFibreoptic airway training: correlation of simulator performance and clinical skill. Canadian journal of anaesthesia = Journal canadien d´anesthesie, 55(2), pp. 100–104

No Grants

#### A733 Living in ICU - watch out for burnout

##### E. Lourenço^1^, L. Melão^1^, C. Duro^1^, G. Baptista^1^, A. Alves^1^, B. Arminda^1^, M. Rodrigues^1^, A. Marreiros^2^, C. Granja^1,3,4^

###### ^1^Centro Hospitalar do Algarve, Hospital de Faro, Emergency and Intensive Care Department, Faro, Portugal; ^2^Biomedical Sciences and Medical Department, Algarve University, Faro, Portugal; ^3^University of Medicine of Porto, CIDES/CINTESIS, Porto, Portugal; ^4^University of Medicine of Faro, Faro, Portugal

####### **Correspondence:** E. Lourenço - Centro Hospitalar do Algarve, Hospital de Faro, Emergency and Intensive Care Department, Faro, Portugal

**Introduction:** The professional activity in the ICU departments has inherent a particularly high level of stress, which is a risk factor for development of burnout.

Studies carried out in this area have revealed high levels of burnout and how these were related with labor absents with its consequences for the functioning of units.

**Objectives:** To identify the possible existence of burnout and its risk and protective factors in health professionals of the ICU Department of Algarve Hospital Center - Faro.

**Methods:** Quantitative, cross-sectional, descriptive study in which we applied a self-fulfilled questionnaire containing three items: sociodemographic characterization of the study population, experiences lived in the workplace based on Embriaco questionnaire and Maslash Burnout Inventory (MBI).

**Results:** Out of 77 professionals, 75 agreed to answer to the questionnaire (response rate 97.4 %). 60 returned the complete questionnaire, 8 physicians and 52 nurses. 43.3 % of the professionals had less than 10 years of work experience and 29.3 % said that if they could they would change profession. We considered burn out if the professional presented high level on Emotional Exhaustion and Depersonalization and low level of Personal Accomplishment; high risk of developing burnout if high level was presented in Emotional Exhaustion or Depersonalization and low level in Personal Accomplishment; and moderate risk if Emotional Exhaustion or Depersonalization presented high level or Personal Accomplishment presented low level. Of the 60 professionals, 48.3 % presented moderate or high risk of developing burnout or were in burnout. Burn out levels were higher in women (26.7 %) versus men (13.3 %) and in physicians (25 %) versus nurses (23.1 %).

**Conclusions:** Burn out or high risk of developing burnout was presented in a high rate of health professionals of our unit. Being woman and physician is associated with a higher risk of developing burnout. Strategies aiming to reduce burnout should include coping strategies and specific training in this field.

**References**

- Pereira, S. Burnout em médicos e enfermeiros: estudo quantitativo e multicêntrico em unidades de cuidados paliativos em Portugal. Revista Enfermagem referência. 2014;IV(3): 55–64. 2

- Teixeira, C. Burnout in intensive care units- a consideration of the possible prevalence and frequency of new risk factors: a descriptive correlational multicenter study. BMC Anesthesiology. 2013;13(38): 25–40

- Nathalie E, Laurent P, Nancy KB, Frederic P, Ellie A. Burnout syndrome among critical care healthcare workers. Curr Opin Critical Care . 2007;13:482–8.

#### A734 Stress and anxiety in relatives of patients on ICU

##### J. Hayward, F. Baldwin, R. Gray

###### Brighton and Sussex University Trust, ICU, Brighton, UK

####### **Correspondence:** J. Hayward - Brighton and Sussex University Trust, ICU, Brighton, UK

**Introduction:** It is known that significant anxiety and depression is suffered by relatives during a patients ICU admission with up to 33 % going on to experience post-traumatic stress disorder (PTSD)^1, 2^. A better understanding of these symptoms and risk factors for developing them may help determine interventions to avoid these adverse psychological states.

**Objectives:** The aim of the study was twofold. The first aim was to quantify the number of relatives with clinically detectable anxiety and depression using a verified scoring system. The second aim was to see how access to information correlated with anxiety and depression scores.

**Methods:** A questionnaire of patients' relatives on the ICU over a six month period (February - August 2015) was carried out. Local ethical approval was granted. Relatives were selected at random to be part of the study and elective admissions and admissions of less than 48 hrs were excluded from the study. The questionnaire collated data on the 'hospital anxiety and depression score' (HADS), the perceived quality of communication delivered and the external support outside the ICU that was sought.

**Results:** The questionnaire was completed by 50 relatives. Clinically significant anxiety was found in 38 (76 %) relatives and 20 (40 %) had clinically significant depression. There was no correlation between length of stay and HADS. There was a correlation between high HADS and perceived high quality of communication on the ICU. This possibly indicates that these relatives sought or required more information to help allay anxieties.

External information sources relating to the ICU and patients medical conditions was actively sought in 20 (40 %) of relatives, with the internet and friends being the most popular sources. Many individuals said they felt isolated when they were at home and relied on friends for support.

**Conclusions:** The results demonstrate that a large proportion of our patients relatives suffer significant acute psychological symptoms. Relatives with higher HADS sought more information from ICU staff and 40 % of relatives also sought external support and information. Following the results of this we have developed a website specific for our ICU with general information, useful links and a contact email address for the ICU consultants. We plan to re audit relatives HADS 6 months after the introduction of the website.

**References**

1. Azoulay E et al. Risk of post-traumatic stress symptoms in family members of intensive care unit patients. American Journal Respiratory Critical Care Med. May 2005 1;171(9):987–94.

2. McAdam et al. Psychological Symptoms of Family Members of High-Risk Intensive Care Unit Patients. American Journal critical care November 2012 21:6; 386–394.

**Grant acknowledgement**

None.

#### A735 The effect of structural simulation based team training in the ICU

##### P.A. Katinakis^1^, M. Stijf^1^, M. Ten Kleij^1^, M. Jansen-Frederiks^1^, R. Broek^1^, M. de Bruijne^2^, P.E. Spronk^1^

###### ^1^Gelre Hospitals, Apeldoorn, Netherlands; ^2^VU University Medical Center, Department of Public and Occupational Health, Amsterdam, Netherlands

####### **Correspondence:** P.A. Katinakis - Gelre Hospitals, Apeldoorn, Netherlands

**Introduction:** The most common sources of clinical errors are not from failure of clinical knowledge or skill, but rather failure of team performance with deficiencies in communication, coordination and teamwork. Growing evidence suggests that team training based on crew resource management (CRM) principles may improve teamwork, patient safety, and optimization of patient care.

**Objectives:** We evaluated the impact of simulation based team training on acute ICU admissions and determined this improved the confidence of the ICU staff and the quality of teamwork.

**Methods:** The project comprised 2 parts. In the first part, all participants received a 2-hour theoretical video training covering the basics of CRM prior the beginning of the study. Subsequently, during a period of 3 months, the admitting physician and coordinating nurse were asked to fill in a survey covering the 4 domains of teamwork (Leadership, communication, stress and situational awareness) on all acute admissions in the ICU. Items were scored on a scale of 0 (not important or worst level) to 10 (very important or highest level).

The second part of the study consisted of structural trainings of all physician- and nursing staff in the ICU divided in groups of 3 nurses and 1 physician, using high fidelity MTW/CRM based simulation with a prefixed scenario. The groups had each to fill in surveys before and after training, and were debriefed by an experienced instructor using video analysis. Scenario trainings were scheduled to be repeated after 6 months.

**Results:** During 133 acute ICU admissions physicians and nurses had the perception that handing over information and communication was above average, however summaries were used poorly (median scores 4 and 6 for physicians and nurses).

After 2 structural trainings, physician and nurses felt they were more competent leaders (P = 0.001). Especially nurses became more confident in a leadership role (P = 0.001). Feedback loops, briefings and summaries were used more systematically (p = < 0.001) and everyone was more accustomed to standard terminology. Furthermore, nurses dared to speak up more (P < 0.001). Overall, stress levels were reduced (P = 0.015).

**Conclusions:** The entire team in the ICU has the perception that structural training in CRM improves confidence and performance. Future research is warranted to compare these results with behavioural observations of the entire team in the ICU.

**Grant acknowledgement**

This study was partially funded by Gelre Hospitals.

#### A736 Evaluation of intensive care doctors' activity outside of the ICU in an urban general hospital

##### K. Sinha, M. Luney, K. Palmer, L. Keating

###### Royal Berkshire Hospital, Reading, UK

####### **Correspondence:** K. Sinha - Royal Berkshire Hospital, Reading, UK

**Introduction:** The number of intensive care unit (ICU) beds in the UK per capita is proportionally one of the lowest in Europe^1^; the Royal Berkshire Hospital (RBH) has one of the lowest proportions compared to the rest of UK. Access to this resource is therefore limited and increasingly intensivists are being asked to bring intensive care to the wards^2^.

**Objectives:** To describe the work carried out by intensive care doctors outside the 17 bed ICU in the 745 bed Royal Berkshire Hospital serving a population of 600,000 people and identify any impact this has on patient outcome.

**Methods:** Contemporaneously recorded requests for intensivists to review patients on inpatient wards and in the emergency department during 1^st^ August 2014 - 31^st^ July 2015 were retrieved from the electronic ICU database. Hospital admission and discharge data were analysed for comparison of outcomes.

**Results:** 1108 patients were referred; paediatric patients (n = 108) and referrals with incomplete details (n = 105) were excluded from analysis. 895 patients were included in analysis, median age 66 years (interquartile range = 48-77 years). 26.6 % were admitted to ICU (n = 238), of which 76.1 % survived to discharge from RBH. 45.6 % referred patients were suitable for but not requiring an ICU admission at the time of referral (96.1 % survived), 17.6 % were at their ceiling of care (ward based care maximum; 19 % survived). 60.7 % of all referred patients not admitted to ICU survived to hospital discharge.

**Conclusions:** Intensivists spend considerable clinical time reviewing patients on general wards, however only a proportion (26.6 %) require admission to an intensive care unit. The outcome for patients in whom escalation in care was felt inappropriate was widely poor. Whereas those reviewed by intensivists and considered eligible for but not requiring of admission to ICU experienced good outcomes (survival rate 96.1 %). Any potential effect of an increased bed capacity on admission and survival is unknown.

**References**

1. Rhodes et al. The variability of critical bed numbers in Europe. Intensive Care Med (2012) 38:1647–1653

2. Hillman K Critical care without walls. Curr Opin Crit Care. 2002 Dec;8(6):594–9

**Grant acknowledgement**

Nil

#### A737 Integration of focussed sonography into undergraduate education in resuscitation medicine

##### M. Abu-Habsa^1,2,3^, R. Bahl^2^, N. Baskaralingam^4^, A. Ahmad^5^, L. Kanapeckaite^4^, P. Bhatti^4^, S. Glace^4^, S. Jeyabraba^4^

###### ^1^Barts Health NHS Trust, Critical Care, London, UK; ^2^Kings College Hospital NHS Foundation Trust, London, UK; ^3^Kent, Surrey and Sussex Air Ambulance, Surrey, UK; ^4^King's College London, London, UK; ^5^King’'s College London, London, UK

####### **Correspondence:** M. Abu-Habsa - Kent, Surrey and Sussex Air Ambulance, Surrey, UK

**Introduction:** Since the advent of Focussed Assessment with Sonography for Trauma (FAST), focussed sonography has become integral to clinical assessment in the early phase of critical illness with wide adoption in Emergency Departments and Critical Care.

Bedside ultrasound (US) guided assessment has been demonstrated to be superior to clinical assessment alone in examination of the chest, abdomen, renal and musculoskeletal systems as well as a fundamental patient safety tool for invasive procedures [1].

While focussed US can be considered an extension of basic clinical skills, most undergraduate programs globally are yet to address this deficit with some uptake in Europe and the United States and none in the UK at present.

This descriptive paper outlines our institutional experience integrating focussed ultrasound training into a 3-month Resuscitation Medicine module.

**Objective:** Explore feasibility, engagement, self perceived and objectively assessed competence following integration of US into undergraduate clinical education.

**Methods:** An elective program on Resuscitation medicine was designed covering undergraduate learning outcomes spanning early identification of critical illness to the concepts of organ support. Nationally adopted ultrasound competencies designed for senior trainees in Emergency Medicine and Critical Care were edited and aligned to each component of the undergraduate module.

Students received an average of 2 hours of formal US training weekly for an 8 week period and were each allocated a handheld device (GE VScan) and this was integrated into their routine clinical assessment. The following themes were prioritised:

- Enhanced Focussed Assessment with Sonography for Trauma (eFAST)
- Echocardiography [RV/LV ratio, gross LV dysfunction, pericardial effusion, major valvular dysfunction]
- Inferior Vena-Caval collapsibility assessment
- Thoracic assessment [pleural effusions and consolidation]

**Results:** Each candidate recorded an average of 35 satisfactory studies. Undergraduate candidates within this pilot were able to achieve an OSCE assessed competence level comparable to that of post graduate trainees approaching Royal Colleges´ defined Level-1 assessment. A high degree of satisfaction was observed in formal feedback. Follow up for skill retention remains ongoing and will be assessed again at 6 months and 12 months in OSCE format. No adverse impact observed on acquisition of set learning outcomes.

**Conclusions:** Integration of US training into undergraduate clinical education proved feasible with a high degree of satisfaction and measured competence. While wider integration would carry cost implications, this is an area that warrants careful review if the modern undergraduate curriculums are to remain fit for 21st century practice.

**References**

1. Kessler C, Bhandarkar S (2010). Ultrasound training for medical students and internal residents - a needs assessment. J Clin Ultrasound 38(8):401–408.

**Grant acknowledgement**

Equipment support by GE Medical

### ICU treatment for the critically ill trauma patient

#### A738 Identification of risk factors for ventilator associated pneumonia in trauma patients

##### H.F. Lewis^1^, A. Kostopoulos^2^, M. Raja^2^, A. West^2^, A. Ely^2^, L.M. Turkoglu^3^, P. Zolfaghari^2^

###### ^1^Barts and the London School of Anaesthesia, The Royal London, Intensive Care Medicine, London, UK; ^2^The Royal London Hospital, London, UK; ^3^GKT Medical School, London, UK

####### **Correspondence:** H.F. Lewis - Barts and the London School of Anaesthesia, The Royal London, Intensive Care Medicine, London, UK

**Introduction:** Ventilator associated pneumonia (VAP) is associated with a significant clinical and financial burden on intensive care units (ICU). Length of stay (LOS), duration of mechanical ventilation (MV) and mortality are all increased^1^. National Healthcare Safety Network's (NHSN) data shows VAP rates in trauma patients are up to 3 fold higher compared to the general ICU population. Centralisation of UK trauma services has created larger cohorts of trauma patients in dedicated tertiary units^1^.

**Objective:** We aimed to identify risk factors within the trauma population that put certain subgroups at higher risk of developing VAP.

**Method:** A retrospective review of all patients admitted to the tertiary adult ICU of the Major Trauma Centre (MTC) at The Royal London Hospital over a 1-year period (February 2014–2015). The diagnosis of VAP was based on the Clinical Pulmonary Infection Score (CPIS). Demographic, clinical and microbiological data were collected. Mortality, LOS and duration of MV differences were studied between VAP and non-VAP groups.

**Results:** Of the 1269 patients admitted to ICU, 925 required invasive ventilation for > 48 hours. Overall incidence of VAP was 11 % (104/925), with rates higher amongst the trauma population 19 % (61/315) compared to the general ICU population 7 % (43/610); p < 0.0001. Patients who developed VAP had increased length of ICU stay (19.5+/− 13.6 vs. 9.71 +/− 11.9 days; p < 0.0001) and duration of MV (15.2 +/− 11.3 vs. 6.9 +/− 9.5 days; p < 0.0001). There was no difference in APACHE II scores and mortality between VAP and non-VAP groups (p = 0.27 and p = 0.16 respectively). Predominant pathogens were H.Influenza, S.Aureus, enterobacter and klebsiella. Trauma patients had increased infection with H.Influenza (28 % vs. 12 %) and S.Aureus (36 % vs. 19 %). Trauma patients who developed VAP had higher mean injury severity scores (ISS) (34 vs. 27; p = 0.0004) and lower mean pre hospital Glasgow Coma Scores (GCS) (7 vs. 9; p = 0.03) compared to trauma patients without VAP. Trauma patients with VAP had a higher incidence of significant head injury compared to those trauma patients who did not get VAP (41/61 (67 %) vs. 134/254 (52 %); p = 0.04). Chest trauma rates were similar between patients who developed VAP and those who did not (18 % v. 14 %; p = 0.43).

**Conclusion:** The trauma population are at high risk for developing VAP. Traumatic brain injury (TBI) patients are the highest risk in this group. The ISS is a better predictor for developing VAP than traditional ICU scoring systems (APACHE II). Amongst the TBI population, ones with lower pre-hospital GCS are at higher risk, and this may reflect loss of protective airway reflexes and higher risk of aspiration. VAP is associated with longer LOS and MV, but crude mortality is unaffected. Further research should ascertain other risk factors for VAP with focussed preventative strategies for high-risk subgroups.

**References**

1. MajorJ et al.Nosocomial Infection in Trauma ICU.*JICS*.2015.16(3)

#### A739 Trauma, male gender and young age are risk factors to the presence of augmented renal clearance (ARC) in critical care ill patients

##### J.P. Baptista^1^, M.P. Marques^2^, P. Martins^1^, J. Pimentel^1^

###### ^1^Centro Hospitalar e Universitário de Coimbra (CHUC), Intensive Care Medicine, Coimbra, Portugal; ^2^Centro Hospitalar e Universitário de Coimbra (CHUC), Bioestatistics, Coimbra, Portugal

####### **Correspondence:** J.P. Baptista - Centro Hospitalar e Universitário de Coimbra (CHUC), Intensive Care Medicine, Coimbra, Portugal

**Introduction:** Augmented renal clearance (ARC) has emerged recently as a new concept and is being increasingly described in critically care setting. The true prevalence of ARC in these heterogeneous populations of critical ill patients is not well known; nevertheless, it must not be neglected: recent reports describe prevalence between 28 to 65 % within the critically ill population, varying accordingly to methodology and *case-mix*.

**Objectives:** We aimed to determine the prevalence of ARC and to identify the risk factors (RF) associated with ARC condition, in a large heterogeneous population of critically ill patients.

**Methods:** This was an observational retrospective single-center study, performed in a 20-bed tertiary level, university affiliated, adult intensive care unit (ICU), within the period of one year. Daily routine 8 h-creatinine clearance (8-CLC_R_) was measured to all admitted patients (23 pm to 07 am), excluding measurements where serum creatinine ≥1.2 mg/dL. ARC was defined as an 8-CLC_R_ ≥130 ml/min/1.73 m^2^. Descriptive demographic and epidemiological characterization of the studied population was performed. Statistical tests used in the analysis were all two-sided, and a p-value of < 0.05 was considered statistically significant. A multivariable binary logistic regression model adjusted for possible confounders (age, gender and cause of ICU admission) was carried out to define RF for ARC condition.

**Results:** For-hundred and seventy-seven patients (477) contributed data, corresponding to 4271 CLC_R_ evaluations (clearance-days). “Medical” was the leading cause of ICU admission (43.3 %), followed by “post-surgery” (31.9 %) and “trauma” (24.7 %). The average SAPS II was 42.3 (12.6). Male gender predominated (65 %) and 98 % of the patients were submitted to invasive mechanical ventilation. Overall, 33 % of the measurements showed ARC (n = 1409), 47 % showed values between 60 and 130 mL/min/1.73 m^2^ (n = 2006) and 20 % had values lower than 60 mL/min/1.73 m^2^ (n = 856). The average 8 h volume of urine was higher in patients showing ARC - 875 *versus* 780 mL; p < 0.001. Serum creatinine and BUN were lower in ARC clearance-days (0.59/0.67 mg/dL, 17/23 mg/dL, respectively; p < 0.01). Adjusted Odds Ratio for ARC was 2.9 (95 % IC: 2.4-3.4; p < 0.01) for gender, 1.7 (95 % IC: 1.4-1.9; p < 0.01) for trauma admission and 1.067 (95 % IC: 1.061-1.073; p < 0.01) for age (per year).

**Conclusions:** ARC was a frequent condition in this large population of critically ill patients with normal plasma creatinine concentrations, with an overall prevalence of 33 % of the days. Decreased renal function (lower than 60 mL/min/1.73 m^2^) was present in 20 % of the days. Male gender, trauma ICU admission and young age were significant risk factors for ARC.

#### A740 A correlative study of ischemic and cell breakdown markers in intraparenchymal traumatic brain injured patients: a cerebral microdialysis correlative study

##### D. Gupta

###### All India Institute of Medical Sciences, Neurosciences Centre, Delhi, India

**Introduction:** Bedside microdialysis monitoring has shown better results compared to CPP-targeted therapy in the treatment of comatose patients sustaining brain injury after severe traumatic brain injury.

**Objectives:** The study aimed to see the role of glycerol and LP ratio monitoring in the brain parenchyma region and its association with cerebral perfusion pressure for outcome prediction.

**Methods:** The study was conducted on 41 patients above 18 years of age with severe traumatic brain injury (GCS < =8) who presented to the emergency with a surgically treatable lesion. Patients who were pregnant, GCS =3 with fixed dilated pupils or hemodynamically unstable were not enrolled in this prospective non-randomised study. As part of routine protocol, patient underwent a non contrast computed tomography (NCCT) head at admission along with assessment of other systemic injuries. Informed consent and Institute ethics review board permission was obtained prior to inclusion in this study. The outcome was assessed using the Glasgow outcome scale (GOS).

**Results:** A total of 3,697 corresponding cerebral perfusion pressure and brain microdialysis readings were obtained. In our study, the mean CPP for the whole cohort was 74.27 mm Hg (SD 14.05).

**Glycerol:** When compared during the first two days of monitoring, the glycerol levels were higher in the unfavourable outcome group (Mean 220.86; SD 25.85) as compared to the favourable outcome group (Mean 191.76; SD 31.89)[NS].

**LP ratio:** In the poor outcome group, 1354 values were available for LP ratio with a mean of 80.16 (SD 320.26). In the good outcome group, 2047 values were obtained with a mean LP ratio of 45.77 (SD 169.82). The difference between the two groups was statistically significant (p = 0.000).

**Cerebral perfusion pressure** was seen to have an inverse correlation to LP ratio for the whole cohort as would be expected with increased perfusion (p = 0.029).

**Conclusions:** CPP monitoring is important after traumatic brain injury since cerebral blood flow is highly dependent on CPP below the lower limit of cerebral autoregulation (CPP < 50 mmHg). Higher CPP by its effect on metabolic parameters, such as LP ratio could be associated with a better outcome.

**References**

Balestreri M, Czosnyka M, Hutchinson P, et al. Impact of intracranial pressure and cerebral perfusion pressure on severe disability and mortality after head injury. *Neurocrit Care*. 2006;4(1):8–13. doi:10.1385/NCC:4:1:008.

Portella G, Cormio M, Citerio G, et al. Continuous cerebral compliance monitoring in severe head injury: its relationship with intracranial pressure and cerebral perfusion pressure. *Acta Neurochir (Wien)*. 2005;147(7):707–713;

**Grant acknowledgement**

Non funded institutional study from public sector setup from India.

#### A741 Increased risk of psychiatric diseases in patients with mild traumatic brain injury: a nationwide cohort study

##### Y.C. Su

###### Dalin Tzu Chi Hospital, Buddhist Tzu Chi Medical Foundation, Chiayi, Taiwan, Province of China

**Background:** It is known that psychiatric disorders after traumatic brain injury are frequent. However, the relationship between mild traumatic brain injury and psychiatric diseases has never been established. We conducted a study of patients with mild traumatic brain injury to evaluate if they had a higher risk of psychiatric diseases compared with the general population.

**Methods:** We utilized a sampled National Health Insurance claims database containing one million beneficiaries. We followed all beneficiaries from January 1, 2005 to December 31, 2013 to determine if they were diagnosed with psychiatric diseases. The definitions of psychiatric diseases in our study are schizophrenia, bipolar disorders and major depression. We further identified patients with mild traumatic brain injury and compared their risk of psychiatric diseases with the general population.

**Results:** We identified 76,991 patients with mild traumatic brain injury and 881,511 patients without mild traumatic brain injury. After controlling for age, gender, urbanization level, socioeconomic status, liver cirrhosis, chronic obstructive pulmonary disease, diabetes, hypertension, coronary artery disease, hyperlipidemia, history of alcohol intoxication, malignancies, smoking, obesity, chronic renal insufficiency and Charlson Comorbidity Index score, the adjusted hazard ratio for psychiatric diseases was 1.46 (95 % confidence interval, 1.29—1.64).

**Conclusion:** Mild traumatic brain injury may be associated with diagnoses of psychiatric diseases.

#### A742 Acute respiratory distress syndrome (ARDS) in children with pulmonary contusion

##### S. Villacres^1^, M.E. Stone^1^, A. Parsikia^1^, S. Medar^2^

###### ^1^Jacobi Medical Center/Albert Einstein College of Medicine, Pediatrics, Bronx, USA; ^2^Albert Einstein College of Medicine & Children's Hospital at Montefiore, Pediatrics, Division of Pediatric Critical Care, Bronx, USA

####### **Correspondence:** S. Medar - Albert Einstein College of Medicine & Children's Hospital at Montefiore, Pediatrics, Division of Pediatric Critical Care, Bronx, USA

**Introduction:** Thoracic injuries account for 4 % to 6 % of children hospitalized for trauma and 25 % of pediatric trauma deaths. Almost a third of patients with chest trauma are found to have pulmonary contusions (PC). There is paucity of data about prevalence of ARDS in children with PC.

**Objective:** To evaluate the incidence and prevalence of PC in children with chest trauma and the association between PC and ARDS.

**Methods:** This is a retrospective single center cohort study. Our institutional Trauma registry was queried for patients 0–18 years of age who were admitted between 5/2008 and 10/2015 with a diagnosis of PC. PC at our center was defined using radiological and clinical criteria. We included patients with trauma with a chest Abbreviated Injury Score (AIS) of 2 or greater. Patient demographic, clinical data including age-adjusted hypotension, initial Glasgow Coma Scale score (GCS) and injury of severity score (ISS) was collected. ARDS was defined using the Berlin Criteria (1) and Pediatric ARDS PALICC criteria (2). We excluded patients who were discharged home from the emergency department or were dead on arrival*.* Continuous Data is expressed as median with interquartile (IQR) ranges and compared with Mann–Whitney *U* test, Fisher's Exact test, and Chi-Square test.

**Results:** Fifty out of 1916 (2.6 %) patients less than 18 yrs. of age with trauma had PC. Of these, 58 % patients were males, and 70 % were of non-Hispanic origin (Black 42 %, White 8 % and other 50 %). Median age was 10.7 yrs (IQR 6.8, 14.3). Median hospital length of stay (HLOS) was 8.5 (4,18) days. Ninety-six percent (48/50) needed admission to the pediatric ICU. Median scores for GCS, AIS chest and ISS were 15 (8, 15), 3 (3, 3) and 22 (18, 29) respectively. Forty two percent (21/50) of the patients with PC needed invasive mechanical ventilation (IMV) with the median duration of ventilatory support being 3 (2, 6) days. Fifty-two percent (11/21) of patients who needed ventilatory support had ARDS with a median p/f ratio of 264 (106, 426)*.* Patients with PC who needed invasive ventilatory support had significantly longer length of ICU stay (7.5 (4, 14) vs. 3.25 (2, 5) days, p = 0.003), lower GCS score (8 (3, 11) vs. 15 (15, 15), p = 0.0001), higher ISS score (29 (22, 34) vs. 19 (14, 22), p = 0.02), and lower S/F ratios (99 (95, 178) Vs. 461 (353, 471) compared to those who did not require ventilatory support. All patients with mortality (4/50) were in the IMV group.

**Conclusions:** Our experience demonstrated that over a quarter of patients with PC will require ventilatory support and more than half of patients with PC requiring ventilatory support will develop ARDS. The need for IMV, higher ISS score and lower GCS are risk factors significantly associated with morbidity and thus help to identify high risk patients.

**References**

1. Ranieri VM et al. (2012) ARDS: the Berlin definition. JAMA 307:2526–2533.

2. Khemani RG et al. Pediatr Crit Care Med. 2015 Jun;16(5 Suppl 1):S23-40

#### A743 Elevated levels of circulating leukocyte-derived microvesicles are related to clinical outcome in major burns injury patients

##### K.P. O'Dea, J. Porter, N. Tirlapur, J.M. Jonathan, S. Singh, M. Takata, Critical Care Research Group

###### ^1^Imperial College London, London, UK

####### **Correspondence:** K.P. O'Dea - Imperial College London, London, UK

**Introduction:** Extracellular microvesicles (MVs) are subcellular plasma membrane-enclosed particles released from activated and apoptotic cells, emerging as key indicators and mediators of disease pathophysiology. Acute increases in levels of circulating MVs have been reported in mechanical trauma and sepsis patients (1), but the relationship with clinical severity is unclear, due potentially to the complex dynamics of acute inflammation. Burn injury is a unique form of trauma with very well defined aetiology, onset of insults and early clinical course, producing acute sterile SIRS in virtually all patients with a moderate to severe degree of injury. Severe burns may therefore represent an optimal study population to evaluate acute MV release and its relationship to the development of SIRS. In this pilot investigation, we measured circulating MV levels following severe burns, with severe sepsis patients as a comparator group.

**Objectives:** To determine the relationship between circulating MVs and early onset SIRS following severe burns injury.

**Methods:** Patients with acute thermal injury admitted to the burns Intensive Care Unit (ICU) were prospectively studied. Baseline demographic data collected included age, sex, Abbreviated Burn Score Index (ABSI) and Belgian Outcome in Burn Injury (BOBI). Blood was sampled within 24 hours of admission and centrifuged to remove cells. MVs derived from leukocytes (CD45+), granulocytes (CD11b+/CD66b+), monocytes (CD45+/CD14+), and endothelial cells (CD105+) were quantified in plasma by flow cytometry. MV levels were compared to samples taken from healthy volunteers and patients with severe sepsis recruited from the general ICU. Sepsis and burns patients that died while on the ICU were classified as non-survivors.

**Results:** All circulating MV subpopulations were elevated in burns patients on day of admission (day 0) compared to healthy volunteers (leukocyte-MVs: 3.5-fold, p = 0.005; granulocyte-MVs: 12.8-fold, p < 0.0001; monocyte-MVs: 20.4-fold, p < 0.0001; endothelial-MVs: 9.6-fold, p = 0.01), but decreased significantly by day 2. MV levels were increased with severe sepsis, but less consistently between patients. Leukocyte- and granulocyte-derived MVs on day 0 correlated with clinical assessment scores and were significantly higher in burns ICU non-survivors compared to survivors (leukocyte MVs 4.6 fold, p = 0.002; granulocyte MVs 4.8 fold, p = 0.003). Mortality prediction analysis of area under receiver operating characteristic curve was 0.92 (p = 0.01) for total leukocyte MVs and 0.85 (p = 0.04) for granulocyte MVs.

**Conclusions:** These findings demonstrate, for the first time, acute increases in circulating MVs following major burns injury and point to their potential role in propagation of sterile SIRS-related pathophysiology.

**References**

1) Reid VL, Webster NR*.* Role of microparticles in sepsis. Br J Anaesth. 2012 109(4):503–13.

**Grant acknowledgement**

Funded by the Chelsea and Westminster Health Charity.

#### A744 Association between hyper-acute hyperglycaemia and injury severity score (ISS) in patients attended by a helicopter emergency medical service (HEMS) in the United Kingdom

##### M. Abu-Habsa^1,2^, A. Ahmad^3^, E. McWhirter^4^, R. Lyon^2,5^

###### ^1^Barts Health NHS Trust, Critical Care, London, UK; ^2^Kent, Surrey and Sussex Air Ambulance, HEMS, Surrey, UK; ^3^King's College London, School of Medicine, London, UK; ^4^Kent, Surrey and Sussex Air Ambulance, Surrey, UK; ^5^Edinburgh Royal Infirmary, Emergency Department, Edinburgh, UK

####### **Correspondence:** M. Abu-Habsa - Kent, Surrey and Sussex Air Ambulance, HEMS, Surrey, UK

**Introduction:** Trauma remains the leading cause of death and disability in patients under 40 years of age [1–4]. It is now widely accepted that patients with higher injury severity benefit from Major Trauma Centre (MTC) care. This has resulted in increasing emphasis on pre-hospital triage decision tools currently based on a combination of physiological data, diagnosis and mechanism of injury.

Hyperglycaemia was previously observed to be a predictor of neurological outcome in Traumatic Brain Injury [1] as well as associated with elevated morbidity and mortality in patients with major trauma [2–3]. On-scene (Pre-Hospital) blood testing reflects the hyper-acute phase of trauma and no previous studies have looked at correlation between pre-hospital capillary glucose levels and subsequent Injury Severity Score (ISS). If such a correlation exists, it may suggest a role for integrating glucose measurements into pre-hospital triage models.

**Objective:** Investigate correlation between pre-hospital hyperglycaemia and subsequent confirmed ISS and 28 day mortality.

**Method:** Records of patients attended by our Helicopter Emergency Medical Service over a 9-month period were retrospectively examined. Clinical assessment and interventions on-scene were contemporaneously recorded electronically by the Physician-Paramedic team including capillary glucose measurements. In addition, physiological, injury and outcome data was prospectively recorded at all MTCs by dedicated staff on a standardised national database - Trauma Audit and Research Network [4]. Patients who did not undergo an on-scene glucose measurement were not included in the study.

**Results:** 166 patients were included in the study. Mean ISS score was 16.5 (0–66) and overall mortality was 7.8 %. The majority [>90 %] of cases were blunt trauma patients. Linear regression analysis suggested a strong correlation between hyperglycaemia > 6.5 mml/L and ISS (P = 0.038) with a larger effect on ISS at a hyperglycaemia cut-off > 7.8 (P = 0.021). There was no signification correlation between pre-hospital hyperglycaemia and 28-day mortality or Critical Care admission within our sample.

**Conclusions:** Our findings suggest that hyperacute elevation in blood glucose may provide an early indication of underlying severe injury and merits further investigation within a larger study. If our findings are replicated, glucose measurement may provide a new parameter in pre-hospital triage algorithms.

**References**

1. Rovlias A, Kotsou S, The influence of hyperglycemia on neurological outcome in patients with severe head injury. Neurosurgery. 2000 Feb;46(2):335–42; discussion 342–3.

2. Yendamuri S, Fulda GJ, Tinkoff GH, Admission hyperglycemia as a prognostic indicator in trauma. J Trauma. 2003 Jul;55(1):33–8.

3. Laird AM, Miller PR, Kilgo PD, Meredith JW, Chang MC, Relationship of early hyperglycemia to mortality in trauma patients. J Trauma. 2004 May;56(5):1058–62.

4. Trauma Audit and Research Network (TARN), United Kingdom, https://www.tarn.ac.uk/Home.aspx

#### A745 Trauma admissions to the intensive care unit at Bahrain Defence Force Hospital

##### M.L. Hariz^1^, E. Azmi^1^, J. Alkhan^2^

###### ^1^BDF Hospital, Anaesthesia ICU, Manama, Bahrain; ^2^BDF Hospital, Manama, Bahrain

####### **Correspondence:** M.L. Hariz - BDF Hospital, Anaesthesia ICU, Manama, Bahrain

**Introduction:** Major trauma has been reported to be a major cause of hospitalization and intensive care utilization worldwide and consumes a significant amount of the health care budget.

**Objectives:** The aim of this study was to describe the characteristics and treatment outcome of major trauma patients admitted into our ICU and to identify predictors of outcome.

**Methods:** Between January 2009 and October 2014, a descriptive retrospective study of all trauma admissions to a multidisciplinary intensive care unit (ICU) of the Bahrain Defence Force Hospital in the Kingdom of Bahrain.

**Results:** A total of 385 cases of major trauma were admitted in the ICU. Males outnumbered females by a ratio of 5.8:1. Their median age was 25 years. Trauma admissions came mainly from the Accident and Emergency (84.7 %). Motor Vehicle Collision (MVC) was the most common cause of injuries affecting 67.4 % of patients. The overall ICU length of stay (LOS) for all trauma patients ranged from 1 to 103 days (mean = 6.4 ± 10.29 days). Mortality rate was 10.4 %. According to multivariate logistic regression analysis, the surgical specialty, revised trauma score < 9, injury severity core >15, the need for ventilatory support and the use of vasopressors significantly influenced mortality (P < 0.05).

**Conclusions:** The impact of trauma on our icu has not been previously documented. This study assesses the pattern, profile, frequency, and outcome of trauma cases admitted to the multidisciplinary ten-bed ICU. The characteristics and evolution of trauma admission of this series of patients are similar to those described abroad. Mortality was in agreement with ISS and RTS scores.

**References**

1. Hofman K, Primack A, Keusch G, Hrynkow S. **Addressing the growing burden of trauma and injury in low- and middle-income countries.** Am J Public Health 2005, 95:13–7.

2. Park K. **Accidents.** In In Textbook of Social and Preventive Medicine.. 17edition. Edited by: Park K. Jabalpur: Banarsidas Co; 2000:304–5.

3. Museru LM, Leshabari MT. **Road traffic Accidents in Tanzania: A 10-year epidemiological Appraisal.** East Central Afr J Surg 2002, 7:23–26.

#### A746 The use of outcome prediction models in severe traumatic brain injury

##### S. Honeybul

###### Sir Charles Gairdner Hospital, Neurosurgery, Perth, Australia

**Introduction:** Predicting long-term neurological outcomes after severe traumatic brain (TBI) is important, but which prognostic model in the context of decompressive craniectomy has the best performance remains uncertain.

**Methods:** This prospective observational cohort study included all patients who had severe TBI requiring decompressive craniectomy between 2004 and 2014, in the two neurosurgical centres in Perth, Western Australia. Severe disability, vegetative state, or death were defined as unfavourable neurological outcomes. Area under the receiver-operating-characteristic curve (AUROC) and slope and intercept of the calibration curve were used to assess discrimination and calibration of the CRASH (Corticosteroid-Randomization-After-Significant-Head injury) and IMPACT (International-Mission-For-Prognosis-And-Clinical-Trial) models, respectively.

**Results:** Of the 319 patients included in the study, 118 (37 %) had unfavourable neurological outcomes at 18-month after decompressive craniectomy for severe TBI. Both CRASH (AUROC 0.86, 95 % confidence interval 0.81-0.90) and IMPACT full-model (AUROC 0.85, 95%CI 0.80-0.89) were similar in discriminating between favourable and unfavourable neurological outcome at 18-month after surgery (p = 0.690 for the difference in AUROC derived from the two models). Although both models tended to over-predict the risks of long-term unfavourable outcome, the IMPACT model had a slightly better calibration than the CRASH model (intercept of the calibration curve = −4.1 *vs*. -5.7, respectively), especially when the predicted risks of unfavourable outcome were < 80 %.

**Conclusions:** Both CRASH and IMPACT prognostic models were good in discriminating between favourable and unfavourable long-term neurological outcome for patients with severe TBI requiring decompressive craniectomy, but the calibration of the IMPACT full-model was better than the CRASH model.

#### A747 Passive verticalization in polyvalent intensive care Unitsklifosovsky Research Institute of Emergency Medicine of the Moscow Healthcare Department, Moscow, Russian Federation

##### V. Movsisyan^1^, S. Petrikov^2^, Z. Marutyan^2^, I. Aliev^2^, A. Evdokimov^2^

###### ^1^N.V. Sklifosovsky Research Institute of Emergency Medicine of the Moscow Healthcare Department, Moscow, Russian Federation; ^2^N.V. Sklifosovsky Research Institute of Emergency Medicine of the Moscow Healthcare Department, Moscow, Russian Federation

####### **Correspondence:** V. Movsisyan - N.V. Sklifosovsky Research Institute of Emergency Medicine of the Moscow Healthcare Department, Moscow, Russian Federation

**Introduction:** Early rehabilitation is one of the most important therapy issue in the Intensive care unit (ICU). One of the most essential part of early rehabilitation is passive verticalization provided by medical tilt table. However there is no information provided about verticatization in polyvalent intensive care unit.

**Objective:** To investigate was the passive verticalization (PV) possibility and safety in the polyvalent ICU.

**Material and methods:** We analyzed 38 episodes of PV in 14 patients of polyvalent ICU: polytrauma (n = 8), isolated, traumatic brain injury (TBI) (n = 8). Verticatization was provided by the medical tilt table. In 24 (63, 2 %) cases we verticalizated patients on artificial lung ventilation (ALV), in 14 (36,8 %) cases on spontaneous breathing. Verticatization was provided up to 20° during first session, up to 40° during the second and up to 50°-60° during the third session. Verticatization session was cared out gradually with the step of 10° in 5 minutes. As the angle of inclination was reached, patient was fixed in a final position during 15 minutes. If any signs of orthostatic failure occurred the patient was still fixed on the reached point till compensation, but not more than for 15 minutes. The session was continued only after orthostatic failure absolute regress. If no regress happed during 15 minutes the sessions was stopped.

**Results:** Patients on ALV successfully reached the target value during 22 (91, 7 %) sessions. Most of the sessions (19(86, 4 %)) were performed without complications; in 3 cases (13, 6 %) were noted some insignificant complications. Two of the sessions (8, 3 %) had to be stopped because of the orthostatic failure. Patients on spontaneous breathing reached a target angle in 12 (85.7 %) session with no complications. Two of the sessions (14, 3 %) were interrupted because of the complications. The most frequent of the verticalization are tachycardia (n = 4 (10, 5 %)), arterial hypotension (n = 5 (13, 2 %)), tachypnea (n = 6 (15, 8 %)).

**Conclusions:** In the polyvalent intensive care unit verticalization can be used safety in patients on ALV and on spontaneous breathing.

#### A748 Hyperoxia during porcine hemorrhage and resuscitation with pre-existing coronary artery disease - part i: effects on kidney function and injury

##### E. Antonucci^1^, T. Merz^2^, C. Hartmann^3^, P. Pelosi^1^, E. Calzia^2^, P. Radermacher^2^, B. Nußbaum^3^

###### ^1^University of Genoa, Department of Surgical Sciences and Integrated Diagnostics, IRCCS San Martino IST, Genoa, Italy; ^2^University Hospital Ulm, Institute of Anesthesiological Pathophysiology and Process Development, Ulm, Germany; ^3^University Hospital Ulm, Anesthesiology, Ulm, Germany

####### **Correspondence:** E. Antonucci - University of Genoa, Department of Surgical Sciences and Integrated Diagnostics, IRCCS San Martino IST, Genoa, Italy

**Introduction:** Hemorrhagic shock is characterised by a mismatch of O_2_ demand and supply resulting in tissue hypoxia, inflammation and subsequent multi-organ failure (MOF)^1^. Hyperoxia reduced inflammation and attenuated kidney dysfunction in hemorrhagic shock in healthy swine^2^. However, pre-existing cardiac disease increases mortality after trauma and hemorrhage^3^.

**Objectives:** To investigate the effect of hyperoxia on kidney function during porcine hemorrhage and resuscitation with pre-existing coronary artery disease (CAD).

**Methods:** Hemorrhagic shock was induced by removal of 30 % of the calculated blood volume (mean arterial pressure (MAP) 43 (40–45) mmHg) and maintained for 3 h in anesthetized and instrumented LDL-receptor^−/−^ pigs with high fat diet-induced hypercholesterolemia and CAD^4^. Post-shock resuscitation comprised re-transfusion of shed blood, crystalloids and infusion of noradrenaline (NA) titrated to maintain MAP at pre-shock values for 48 h. So far, n = 7 animals were subjected to hyperoxia (FiO_2_ 1.0) during the initial 24 h of resuscitation, n = 8 pigs received standard treatment (FiO_2_ 0.3, SaO_2_ ≥ 90 %). Before, at the end of, and every 12 hours after hemorrhage, we assessed systemic and renal hemodynamics (renal artery ultrasound flow probe), kidney function (creatinine clearance, urinary output, renal venous blood gas analysis) as well as mitochondrial activity (high resolution respirometry) of kidney specimens collected at the end of the experiment. Data are median (range).

**Results:** Survival time, NA requirements as well as systemic and renal hemodynamics were comparable in both groups. However, plasma creatinine levels were significantly lower after 36 (p < 0.01) and 48 h (p < 0.02) and creatinine clearance was significantly higher at day 2 of resuscitation (115 (100–132) ml/min vs. 64 (33–97) ml/min; p < 0.02) in the hyperoxic group. Moreover, maximal oxidative phosphorylation (OxPhos) (hyperoxia: 241 (227–286), control: 189 (164–231) pmol*mg^−1^*s^−1^; p < 0.03) and O_2_ flux related to ATP production (178 (171–201) vs. 128 (95–147) pmol*mg^−1^*s^−1^; p < 0.03) of renal mitochondria were significantly higher in the hyperoxic animals.

**Conclusion:** In our model of porcine, resuscitated hemorrhagic shock with pre-existing CAD, hyperoxia significantly improved kidney function due to increased mitochondrial respiratory capacity which is in good agreement with previous results from healthy swine^2^.

**References**

1. Angele, M. K. et al. Crit Care 2008; 12:218

2. Knöller, E. et al. Crit Care Med 2015; Epub ahead of print

3. Ferraris, V. et al. J Trauma 2010; 69:645–652

4. Thim, T. et al. EuroIntervention 2010; 6:261.

Supported by the DFG (CRC1149).

#### A749 The role of chronic obstructive lung disease in a combined trauma model of blunt chest trauma and hemorrhagic shock

##### C. Hartmann^1^, M. Huber-Lang^2^, M. Gröger^3^, P. Radermacher^3^, B. Nußbaum^1^

###### ^1^Department of Anesthesiology, University Hospital, Ulm, Germany; ^2^Department of Traumatology, Hand-, Plastic- and Reconstructive Surgery, University Hospital, Ulm, Germany; ^3^Institute of Anesthesiological Pathophysiology and Process Development, Ulm, Germany

####### **Correspondence:** C. Hartmann - Department of Anesthesiology, University Hospital, Ulm, Germany

**Introduction:** Hemorrhagic shock (HS) accounts for about 40 % of trauma-related deaths [1]. The severity of trauma can be further deteriorated by an additional blunt chest trauma (TxT), which independently contributes to mortality upon the development of acute lung injury (ALI) [2]. The development of ALI following a severe trauma is encouraged by both active and passive cigarette smoke (CS) exposure [3]. We previously showed that CS exposure prior to TxT enhanced post-traumatic inflammation thereby aggravating lung injury [4].

**Objectives:** We investigated the impact of pre-traumatic CS exposure in mice following HS alone or in combination with TxT.

**Methods:** After 3–4 weeks of CS-exposure, anesthetized and spontaneously breathing (C57BL/6) mice underwent TxT or sham surgery. After initiation of lung-protective mechanical ventilation via tracheostomy and instrumentation for vascular access, mice underwent 1 h of hemorrhage (mean arterial pressure (MAP) = 35 mmHg) followed by resuscitation comprising re-transfusion of shed blood, fluid administration and noradrenaline (NA) titrated to maintain MAP > 50 mmHg. Lung mechanics, gas exchange, hemodynamics, metabolism, and acid–base status were measured together with lung and plasma cytokine and chemokine levels. After 4 h, lung and kidney were harvested for immunohistochemistry and western blotting.

**Results:** Pulmonary compliance was increased in chronic obstructive lung disease (COPD) mice which had only received HS. Lung tissue levels of the inducible nitric oxide synthase (iNOS) were slightly elevated in nonCOPD mice which, due to more NO availability, is in line with increased NA requirements to maintain hemodynamic targets. Also, systemic and pulmonary cytokine and chemokine levels as well as lung tissue expression of heme oxygenase-1 (HO-1) were increased in COPD mice. Their mortality rates were similar, whereas in the combined trauma model COPD mice showed an increase in mortality and elevated NA requirements compared to nonCOPD mice. Lung tissue HO-1 expression was reduced in the combined trauma model.

**Conclusion:** CS exposure aggravated post-traumatic inflammation. CS exposure prior to TxT and subsequent HS was associated with increased mortality rates. As HO-1 has been reported to act protective in ALI [5], we presume its reduction strongly highlights the severity of trauma due to an exhausted ALI-associated anti-inflammatory defence system.

**References**

[1] Kauvar et al. J Trauma. 2006; 60:S3-11

[2] Shah et al. Crit Care Med. 2008; 36:2309–2315

[3] Calfee et al. Am J Respir Crit Care Med. 2011; 183:160–165

[4] Wagner et al. PLoS One. 2015, 10:e0132810

[5] Otterbein et al. Chest. 1999;116: 61S-63S.

**Grant acknowledgement**

Supported by the DFG (CRC1149).

#### A750 Hyperoxia during porcine hemorrhage and resuscitation with pre-existing coronary artery disease - part II: effects on left ventricular function

##### B. Nußbaum^1^, E. Antonucci^2^, E. Calzia^3^, P. Pelosi^2^, P. Radermacher^3^, C. Hartmann^1^

###### ^1^University Hospital Ulm, Anesthesiology, Ulm, Germany; ^2^University of Genoa, Department of Surgical Sciences and Integrated Diagnostics, IRCCS San Martino IST, Genoa, Italy; ^3^University Hospital Ulm, Institute of Anesthesiological Pathophysiology and Process Development, Ulm, Germany

####### **Correspondence:** B. Nußbaum - University Hospital Ulm, Anesthesiology, Ulm, Germany

**Introduction:** Multi-organ failure after hemorrhagic shock is triggered by tissue hypoxia and inflammation resulting from the mismatch between O_2_ demand and supply^1^. In healthy swine, hyperoxia (increased FiO_2_) reduced inflammation and organ dysfunction^2^ after hemorrhagic shock and did not adversely affect myocardial function upon critical hemodilution^3^. Pre-existing coronary artery disease (CAD) might increase the risk of cardiac injury during low flow states. However, the use of increased FiO_2_ is restricted in acute coronary syndrome due to vasoconstriction and subsequent myocardial damage^4^.

**Objectives:** To investigate the influence of hyperoxia on left ventricular (LV) function during hemorrhage and resuscitation in swine with pre-existing CAD.

**Methods:** Hemorrhagic shock was induced by removal of 30 % of the calculated blood volume (target mean arterial pressure (MAP) 40 mmHg) and maintained for 3 h in anesthetized LDL-receptor^−/−^ pigs with high fat diet-induced hypercholesterolemia and CAD^5^. Post-shock resuscitation comprised re-transfusion of shed blood, crystalloids and noradrenaline (NA) to maintain MAP at pre-shock values for 48 h. Amiodarone was used if needed to treat arrhythmia. So far, n = 7 pigs were subjected to hyperoxia (FiO_2_ 1.0) for the initial 24 h of resuscitation, n = 8 pigs received standard treatment (FiO_2_ 0.3, SaO2 ≥ 90 %). Before, at the end of, and every 12 hours after hemorrhage, we assessed hemodynamics and LV function (pressure-conductance catheter) and cardiac mitochondrial activity at the end of the experiment. Data are median (IQR).

**Results:** Survival time, hemodynamics and total doses of NA and amiodarone did not significantly differ between groups. Dp/dt_max_ transiently increased after resuscitation and decreased again in both groups, whereas dp/dt_min_ and the isovolumic relaxation constant tau remained largely unaffected throughout the experiment despite high doses of NA and tachycardia. Together with the mostly unchanged ejection fraction, the fall in stroke volume and the rise in filling pressure (pulmonary artery occlusion pressure) after re-transfusion indicate increased LV stiffness and dysfunction in both groups.

However, all cardiac parameters and mitochondrial respiratory capacity of the heart did not show any significant differences between hyperoxic and control animals.

**Conclusion:** In our porcine model of resuscitated hemorrhagic shock with pre-existing CAD, hyperoxia did not exert detrimental effects with respect to LV function. Therefore, hyperoxic treatment appears to be a safe approach for the treatment of O_2_ deficiency in hemorrhagic shock even under conditions with pre-existing cardiac comorbidity.

**References**

1. Angele, M. K. et al. Crit Care 2008; 12:218.

2. Knöller, E. et al. Crit Care Med 2015; Epub ahead of print.

3. Kemming, G. I. et al. Acta Anaesthesiol Scand 2004; 48:951–959.

4. Stub, D. et al. Circulation 2015; 131:2143.

5. Thim, T. et al. EuroIntervention 2010; 6:261.

Supported by the DFG (CRC1149).

**Table 77 (abstract A750).** Cardiac parameters

		Pre-shock	12h	24h	36h	48h
dp/dt max (mmHg/s)	normoxia hyperoxia	2618 (2502;3291) 2770 (2696;3047)	3883 (2754;5639) 3774 (2418;6591)	4218 (3347;5078) 4762 (2734;5196)	3001 (2274;5841) 2986 (2818;4596)	2870 (2641;3098) 2806 (2209;3310)
dp/dt min (mmHg/s)	normoxia hyperoxia	−2032 (−2474;-1830) -1971 (−2219;-1805)	−2484 (−2727;-2240) -2925 (−3442;-1939)	−2496 (−4294;-1683) -2662 (−3098;-2245)	−3006 (−4348;-1458) -2646 (−2926;-2454)	−3113 (−3270;-2956) -2615 (−2798;-2428)
Tau (ms)	normoxia hyperoxia	30 ( 22 ; 34 ) 26 ( 23 ; 31 )	18 ( 14 ; 28 ) 23 ( 12 ; 33 )	14 ( 11 ; 17 ) 15 ( 15 ; 20 )	18 ( 12 ; 20 ) 17 ( 15 ; 20 )	18 ( 18 ; 18 ) 21 ( 19 ; 23 )
Stroke volume (ml)	normoxia hyperoxia	72 ( 50 ; 85 ) 61 ( 59 ; 89 )	37 ( 24 ; 60 ) 33 ( 30 ; 72 )	37 ( 30 ; 74 ) 40 ( 34 ; 43 )	38 ( 28 ; 62 ) 51 ( 29 ; 61 )	47 ( 32 ; 60 ) 40 ( 28 ; 59 )
PAOP (mmHg)	normoxia hyperoxia	11 ( 09 ; 12 ) 11 ( 10 ; 14 )	17 ( 16 ; 20 ) 19 ( 16 ; 21 )	17 ( 16 ; 18 ) 20 ( 17 ; 21 )	20 ( 18 ; 22 ) 19 ( 16 ; 23 )	18 ( 17 ; 21 ) 19 ( 17 ; 23 )
EF (%)	normoxia hyperoxia	48 ( 44 ; 78 ) 46 ( 39 ; 65 )	46 ( 31 ; 52 ) 39 ( 21 ; 46 )	55 ( 50 ; 60 ) 64 ( 45 ; 67 )	60 ( 18 ; 67 ) 28 ( 20 ; 39 )	35 ( 30 ; 39 ) 43 ( 31 ; 63 )

### Perioperative care of cardiac surgery patients

#### A751 Genetic regulation of the host response to cardiac surgery and cardiopulmonary bypass

##### E. Svoren-Jabalera^1^, E.E. Davenport^2^, P. Humburg^2^, J. Knight^2^, C.J. Hinds^3^

###### ^1^Queen Mary University of London, Queen's Hospital University Hospital Trust, London, UK; ^2^Oxford University Hospitals NHS Trust, Wellcome Trust Centre for Human Genetics, Oxford, UK; ^3^Queen Mary University of London, Barts Hospital and The London School of Medicine, London, UK

####### **Correspondence:** E. Svoren-Jabalera - Queen Mary University of London, Queen's Hospital University Hospital Trust, London, UK

**Introduction:** Differential expression of specific genes has been associated with postoperative brain injury and individual variation in the severity of the host response following cardiac surgery _1,2_. We investigated the role of DNA sequence variation in modulating the observed changes in gene expression.

**Objectives:** Our aim was to reveal the most relevant regulatory genetic variants of the host response to elective cardiac surgery and the clinical relevance of the variants detected. To identify these regulatory variants we performed an Expression Quantitative Analysis (e-QTL).

**Methods:** The Ambion LeukoLOCK Total RNA Isolation System was used to purify leukocytes from whole blood isolated before induction of anaesthesia and 24 hours after surgery. Gene expression was quantified using Illumina HumanHT-12 v4 expression beadchip . The output of this analysis was filtered by fold expression and P-values were corrected to false discovery rate < 0.05. Genotyping was performed on OmniExpressed-12v1chip. e-QTLS analysis was peformed using Matrix e-QTL software. Pathways analysis was performed using Ingenuity Pathways Analysis (web based software).

**Results:** 46 patients scheduled for elective cardiac surgery involving cardiopulmonary bypass (CBP) were recruited. Postoperative recovery was uneventful with only minor complications.

Of the genes differentially regulated 24 hours after cardiac surgery 80 were up-regulated and 15 were down -regulated. The group of up-regulated genes are primarily related to the innate immune response and cell signalling. Of the 15 downregulated the majority are implicated in T lymphocyte activation.

We identified 659 single nucleotide polymorphisms (10.E-06) that are significantly associated with the cis regulation of 218 genes, 24 hours after cardiac surgery. Analysis of the 10 top enriched pathways showed three groups 1-Granulocyte-macrophage colony stimulating factor (GM-CSF) group. 2- Interferon and growth factors group 3- Leptin Pathway group .

**Conclusions:** The pathways associated most strongly with the post-operative response to surgery, are related to innate immune response. Interestingly, we detected genes/pathways not conventionally associated with the host response to cardiac surgery such as cell proliferation, cell differentiation and cell signalling. Pro-inflammatory and anti-inflammatory genes operate simultaneously rather than sequentially after cardiac surgery and CPB.

**References**

1. Ramlawi B et al. Genomic expression pathways associated with brain injury after cardiopulmonary bypass. J Thorac Cardiovasc Surg 2007;134:996–1005.

2. Tomic V et al. Transcriptomic and proteomic patterns of systemic inflammation in on-pump and off-pump coronary artery bypass grafting. Circulation 2005; 112:2912–20.

**Grant Acknowledgment**

Young Investigator Award Intensive Care Society (ICS), UK.

#### A752 Association between perioperative intravenous fluid administration strategy and renal outcomes in patients undergoing cardiovascular surgery: an observational study

##### I.-J. Jun, W.-J. Kim, E.-H. Lee

###### Asan Medical Center, Department of Anesthesia, Seoul, Republic of Korea

####### **Correspondence:** I.-J. Jun - Asan Medical Center, Department of Anesthesia, Seoul, Republic of Korea

**Introduction:** Saline-based and hydroxyethyl starch solutions are commonly used for volume resuscitation, but have been linked to an increased risk of renal dysfunction.

Objective: To assess whether balanced solutions and a limited volume of hydroxyethyl starch (renal-protective fluid management [RPF] strategy) during perioperative period could decrease the incidence of postoperative renal dysfunction and improve clinical outcomes.

**Method:** This retrospective study investigated 2613 patients undergoing cardiovascular surgery. A group of patients undergoing cardiovascular surgery from January 1, 2010 to July 4, 2012 were included in the control group and were given intravenous fluids with saline-based solutions and hydroxyethyl starch perioperatively. The other group of patients undergoing cardiovascular surgery from July 23, 2012 to December 31, 2013 formed the RPF group and were given balanced solution and a limited volume of hydroxyethyl starch solution. The primary outcome was the incidence of postoperative acute kidney injury (AKI) using serum creatinine. Secondary outcomes included the incidence of severe AKI, requirement for renal replacement therapy, renal outcome at the time of discharge. Propensity score and multivariable regression analyses were performed to assess the association of fluid management strategy with renal outcomes.

**Result:** In the entire cohorts, postoperative AKI, severe AKI, in-hospital renal replacement therapy, and persistent AKI at discharge occurred in 213 (21.2 %), 54 (5.4 %), 29 (2.9 %), and 52 (5.2 %) patients of RPF group compared to 696 (43.2 %), 254 (15.8 %), 79 (4.9 %), and 194 (12.1 %) patients of control group, respectively. In the propensity score matched cohort, the incidence of AKI, severe AKI, in-hospital RRT, and persistent AKI at discharge were also lower in the RPF group than in the control group. After adjusting by the multivariable analyses, the RPF group was independently associated with a lower risk of postoperative AKI, severe AKI, use of renal replacement therapy, shorter postoperative extubation time and intensive care unit and hospital stay.

**Conclusion:** The RPF strategy was associated with improved acute renal and clinical outcomes after cardiovascular surgery. These findings support the need for definitive clinical studies on RPF strategy.

**References**

1. Yunos NM, Bellomo R, Hegarty C, et al. Association between a chloride-liberal vs chloride-restrictive intravenous fluid administration strategy and kidney injury in critically ill adults. Jama 2012; 308(15): 1566–72.

2. Myburgh JA, Finfer S, Bellomo R, et al. Hydroxyethyl starch or saline for fluid resuscitation in intensive care. The New England journal of medicine 2012; 367(20): 1901–11.

3. Kim JY, Joung KW, Kim KM, et al. Relationship between a perioperative intravenous fluid administration strategy and acute kidney injury following off-pump coronary artery bypass surgery: an observational study. Critical care 2015; 19: 350.

#### A753 Safety and efficacy of intravenous exenatide in the management of stress hyperglycemia after coronary artery graft bypass surgery: the ExSTRESS phase II trial

##### G. Besch^1,2^, A. Perrotti^2,3^, M. Puyraveau^4^, L. Carteron^1,2^, M. Baltres^1^, E. Samain^1,2^, S. Chocron^2,3^, S. Pili-Floury^1,2^

###### ^1^University Hospital of Besancon, Anesthesiology and Critical Care Medicine, Besançon, France; ^2^University of Franche-Comté, Besançon, France; ^3^University Hospital of Besancon, Thoracic and Cardiovascular Surgery, Besançon, France; ^4^University Hospital of Besancon, Clinical Methodology Center, Besançon, France

####### **Correspondence:** G. Besch - University of Franche-Comté, Besançon, France

**Introduction:** Intravenous infusion of short-acting insulin remains the gold standard treatment of stress hyperglycemia after coronary artery graft bypass (CABG) surgery despite the risk of insulin-related hypoglycemia. Exenatide is an agonist of the glucagon-like peptide-1 receptor that stimulates pancreatic insulin secretion and inhibits pancreatic glucagon production. The risk of exenatide-related hypoglycemia is theoretically null as the effect of exenatide depends on the blood glucose level.

**Objectives:** The aim of the ExSTRESS study was to assess the efficacy and the safety of intravenous exenatide in the management of stress hyperglycemia after CABG surgery.

**Methods:** The ExSTRESS study (NCT01969149) was a one-center randomized phase II trial. Patients scheduled for CABG surgery between January and December, 2015 were eligible. Non-inclusion criteria were: insulin-requiring diabetes mellitus, creatinine clearance < 60 ml/min, and a medical history of pancreatectomy, acute or chronic pancreatitis. Intravenous exenatide (EXE group) or intravenous insulin (INS group) was randomly allocated to achieve a blood glucose target between 5.5 and 7.7 mmol/l. A validated insulin therapy protocol was used in the INS group [1]. The primary outcome was the percentage of patients achieving the blood glucose target during at least half of the first 48 postoperative hours. The secondary outcomes were: incidence of moderate (<3.3 mmol/l) and severe (<2.2 mmol/l) hypoglycemia during the first 48 postoperative hours, and the incidence of adverse events at Day 30. According to the study protocol (O'Brien and Fleming plan for analysis), no statistical test was used for intergroup comparison. Data are presented as mean (standard deviation) or number of patients (percentage).

**Results:** 54 and 52 patients were included respectively in the EXE and in the INS groups (age : 70 (9) vs 68 (11) years; male : 49 (91) vs 42 (81); diabetes mellitus : 12 (27) vs 11 (26); HbA1c : 6.1 (0.7) vs 5.8 (0.6) %; Euroscore : 6.4 (2.3) vs 6.1 (2.7) %; respectively in the EXE and in the INS groups). 39 (72) and 39 (75) achieved the blood glucose target during at least half of the first 48 postoperative hours respectively in the EXE and in the INS groups. The mean blood glucose value was 6.9 (0.5) mmol/l vs 6.9 (0.4) mmol/l respectively in the EXE and in the INS groups. 0 (0) and 0 (0) patient has presented at least one episode of severe hypoglycemia and 2 (4) vs 1 (2) patients suffered from at least one moderate hypoglycemia respectively in the EXE and in the INS groups. 6 (11) vs 3 (6) patients did not require intravenous insulin respectively in the EXE and in the INS groups. No exenatide-related adverse event has been reported at Day 30.

**Conclusions:** Intravenous exenatide allows a safe management of stress hyperglycemia after CABG surgery but requires intravenous insulin rescue therapy in most patients.

**References**

[1] Studer C et al. Diabetes Metab. 2010

#### A754 Pilot study to evaluate the use of an adsorption membrane (OXIRIS®) during cardiopulmonary bypass surgery

##### E.P. Plata-Menchaca^1^, J. Sabater-Riera^2,3^, M. Estruch^4^, E. Boza^5^, F. Sbraga^6^, J. Toscana-Fernández^6^, E. Bruguera-Pellicer^6^, J. Ordoñez-Llanos^7^, X.L. Pérez-Fernández^3,8^, SIRAKI group

###### ^1^Instituto Nacional de Ciencias Médicas y Nutrición Salvador Zubirán, Critical Care, México, Mexico; ^2^Hospital Universitario de Bellvitge, Critical Care, L´Hospitalet, Spain; ^3^Instituto de Investigacion Biomédica de Bellvitge, L´Hospitalet, Spain; ^4^Hospital de la Santa Creu i Sant Pau, Laboratori de Recerca Cardiovascular, Barcelona, Spain; ^5^Hospital Universitari de Bellvitge, Anesthesiology, L´Hospitalet, Spain; ^6^Hospital Universitari de Bellvitge, Cardiac Surgery, L´Hospitalet, Spain; ^7^Hospital de la Santa Creu i Sant Pau, Institut de Recerca Cardiovascular, Barcelona, Spain; ^8^Hospital Universitari de Bellvitge, Critical Care, L´Hospitalet, Spain

###### **Correspondence:** E.P. Plata-Menchaca - Instituto Nacional de Ciencias Médicas y Nutrición Salvador Zubirán, Critical Care, México, Mexico

**Introduction:** Cardiopulmonary Bypass Surgery (CPB) is associated with organ dysfunction as a consequence of a generalized inflammatory response due to blood contacting the synthetic surfaces of the bypass equipment.

**Objectives:** To evaluate the safety and feasibility of increased adsorption membranes during cardiopulmonary bypass surgery.

**Methods:** This is a pilot study that included 20 cardiac surgery patients. Ten patients were assigned to receive CPB + OXIRIS**®** during cardiac surgery and 10 patients were assigned to receive standard CPB. Blood samples were collected and clinical variables were recorded (just before connection to CPB (T0), just before ending CPB (T1), at intensive care unit (ICU) admission (T2), and 24 hours after ICU admission (T3)). Clinical variables were registered from CPB and up to 24 hours from ICU admission, including any adverse effect observed during the use of OXIRIS**®** membrane.

**Results:** Twelve patients underwent coronary artery bypass graft (CABG) surgery, 3 patients uderwent valve replacement surgery, and 5 patients underwent mixed surgery (CABG plus valve replacement). No safety events were registered in both groups. Mean age was 65.9 ± 9 years and 85 % of patients were male. Mean baseline serum creatinine was 89 ± 15 μmol/L, and mean CPB time was 113 ± 38 minutes. At T1, mean concentration of cytokines was not statistically different between groups. In CPB-OXIRIS® group, IL-1 and IL-6 levels were lower as compared with standard CBP. Interleukin-4 and IL-10 were increased in the CPB-OXIRIS® group, whereas tumor necrosis factor-α levels were not different between groups. At T2, mean serum levels of IL-1 and IL-6 were increased in both groups. Furthermore, no adverse effects were observed in CBP-OXIRIS® group during follow up.

**Conclusions:** No adverse effects were observed with the use of OXIRIS®, neither during CPB nor after 24 hours of surgery. Therefore, the use of an increased adsorption membrane connected to CPB is safe and feasible.

#### A755 Evolution of diaphragmatic dysfunction after cardiac surgery

##### P. Cavaleiro^1^, A. Tralhão^2^, M. Arrigo^3^, J.-P. Lopes^4^, M. Lebrun^4^, B. Cholley^4^

###### ^1^Algarve Hospital Center, Faro Unit, Faro, Portugal; ^2^Hospital de Santa Cruz / CHLO, Lisboa, Portugal; ^3^Zurich University Hospital, Zurich, Switzerland; ^4^Georges Pompidou European Hospital, Paris, France

####### **Correspondence:** P. Cavaleiro - Algarve Hospital Center, Faro Unit, Faro, Portugal

**Introduction:** Diaphragmatic dysfunction is a known complication of cardiac surgery associated with delayed weaning from mechanical ventilation. Its incidence and evolution has not been established in detail. Echographic evaluation of diaphragmatic excursion is a simple, non-invasive technique that can be used at the bedside to monitor diaphragmatic function.

**Objectives:** To evaluate the incidence and evolution of diaphragmatic dysfunction in patients undergoing cardiac surgery.

**Methods:** This prospective observational study was conducted from 16/10 to 23/12/2015 at a tertiary cardiac surgical center in France. Consecutive elective adult patients operated via a median sternotomy with cardiopulmonary bypass were enrolled. Demographic data was collected. Echographic measurements of right and left hemi-diaphragm excursions were obtained using anatomical M-Mode and averaged over 3 respiratory cycles before surgery (E1), within 48 h following extubation (E2) and between 96 and 144 h after extubation (E3). Values were compared using Friedman's analysis for repeated measures and Wilcoxon test for paired samples. Diaphragmatic dysfunction was defined as an excursion < 1 cm, as previously reported^1,2^. An inter-observer reproducibility evaluation was performed using Bland-Altman analysis.

**Results:** 143 patients were submitted to cardiac surgery during the study period, of which 106 fulfilled inclusion criteria. 26 patients were not included: 8 did not consent, machine or operator were unavailable in 19 patients at E1. 80 patients were included and 79 were analysed (1 patient withdrew consent). The evolution of right and left hemi-diaphragmatic excursion is shown in graph 1. After establishing pre-op baseline values (E1), we observed a shorter excursion after surgery (E2) with partial recovery at E3 (Table 78). Patients that did not develop dysfunction followed a similar pattern. At E2, unilateral dysfunction was observed in 25 patients (37 %) and bilateral dysfunction in 4 (5 %). At E3, unilateral dysfunction remained in 9 patients (13 %).

**Conclusions:** Diaphragmatic excursion was reduced after surgery but returned mostly to normal by day 5. 30 % of patients developed a diaphragmatic dysfunction but this was not associated with clinically relevant complications in this small cohort.

**References**

1. Boussuges A, Gole Y, Blanc P. Diaphragmatic motion studied by M-mode ultrasonography: methods, reproducibility, and normal values. Chest. 2009;135:391–400;

2. Pasero D, Koeltz A, Placido R, Fontes Lima M, Haun O, Rienzo M et al. (2015) Improving ultrasonic measurement of diaphragmatic excursion after cardiac surgery using the anatomical M-mode: a randomized crossover study. Intensive Care Med 41(4):650–656

**Table 78 (abstract A755).** Diaphragmatic excursion

Variable	Patient N	p value (Friedman analysis or Wilcoxon test)
Right Excursion - E1, E2 and E3	67	<0,001
Left Excursion - E1, E2 and E3	62	0,032
Right Excursion - E1 vs E2	72	<0,001
Right Excursion - E1 vs E3	72	0,020
Right Excursion - E2 vs E3	67	<0,001
Left Excursion - E1 vs E2	67	0,003
Left Excursion - E1 vs E3	71	0,323
Left Excursion - E2 vs E3	62	0,017

**Fig. 108 (abstract A755).**
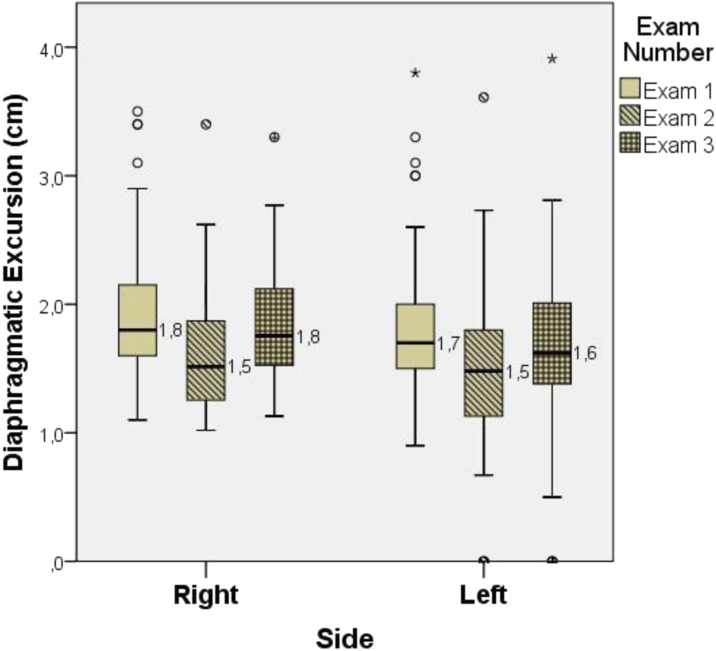
Diaphragmatic excursion – BoxPlot

#### A756 Low cardiac output syndrome and renal failure in postoperative cardiac surgery patients. Esbaga study

##### J.L. PerezVela^1^, H. MarinMateos^1^, J.J. Jimenez Rivera^2^, M.A. Alcala Llorente^3^, B. Gonzalez De Marcos^4^, F.J. Gonzalez Fernandez^5^, C. Garcia Laborda^6^, D. Fernandez Zamora^7^, Grupo ESBAGA

###### ^1^Hospital 12 de Octubre, Madrid, Spain; ^2^Complejo Universitario Canarias, Tenerife, Spain, ^3^Fundacion Jimenez Diaz, Madrid, Spain; ^4^Hospital 12 de la Princesa, Madrid, Spain; ^5^Hospital Virgen Macarena, Sevilla, Spain; ^6^Hospital Miguel Servet, Zaragoza, Spain; ^7^Hospital Carlos Haya, Malaga, Spain

####### **Correspondence:** J.L. PerezVela - Hospital 12 de Octubre, Madrid, Spain

**Introduction:** Low cardiac output syndrome (LCOS) is a potential complication in patients undergoing cardiac surgery. This clinical situation can be associated with increased morbidity (including acute deterioration of renal function) and mortality.

**Objetive:** Descriptive analysis of patients undergoing cardiac surgery and has a low cardiac output syndrome (LCOS), its relationship with renal failure and association with mortality.

**Methods:** Observational, prospective, multicenter study of patients admitted to the ICU during the post-surgical period and develops a LCOS (2012 Consensus definition). Preoperative data (type of intervention, cardiac function, risk scores), intraoperative variables (type of surgery, CEC, use of inotropic) and postoperative data to hospital discharge (including complications / postoperative mortality) are recorded. Descriptive statistics, ANOVA and Chi2 analysis, from June 2014 to June 2015. Data analyzed using SPSS-windows.

**Results:** 14 centers have provided 138 patients with LCOS (age 68.3 ± 9.3 years; 65 % male) operated mostly scheduled basis (59.4 %, 33.3 % urgency and 6.5 % emergency) with an Euroscore of 9.99 ± 13. Isolated myocardial revascularization (16.8 %) or accompanied by valve surgery (24.8 %) and aortic valve replacement (11.7 %) were the most frequent procedures. Most relevant antecedents: AMI (31.9 %; 22 cases in the pre-intervention 90 days), severe pulmonary hypertension (21.7 %), critical preoperative state (18.8 %), previous cardiac surgery (18.1 %), PTCA / stent (16.7 %), LVEF < 35 % (33.6 %), and NYHA III-IV (52.9 %). Prior to surgery, 21 patients (15.4 %) requiring vasoactive drugs. 70.1 % needed two (or more) vasoactive drugs for exit of the CPB (mean 147.4 ± 60 min). Postoperatively, 46.7 % of patients with LCOS (n = 63) develops acute deterioration of renal function (39 % AKI1, 23 % AKI2 and 37.7 % AKI3). The time to worst creatinine value was 41 ± 46 hours. 20 % required renal replacement technique; there is significant difference in the cardiogenic shock (CS) group (11.4 % LCOS, 14.6 % clinical group and 36.6 % CS, p = 0.007). Analyzing by subgroup of AKI (worst recorded value in the evolution), the AKI3 stage was associated with multiorgan failure (MOF) (17.4 % AKI1, 28.6 % AKI2, 68.2 % AKI3, p = 0.001), total days income (p = 0.03) and ICU mortality (16.7 % AKI1, 28.6 % AKI2 and 69.6 % AKI3, p = 0.001).

**Conclusions:** LCOS patients have a high incidence of acute deterioration of renal function and need for renal replacement technique. The AKI stadium is associated with increased development of MOF and mortality.

#### A757 Evaluation of inmature platelets fraction in patients undergoing cardiac surgery: a pilot study

##### J.C. Lopez Delgado^1^, C. Imperiali^2^, D. Berbel-Franco^1^, M. Dastis^2^, G. Moreno-Gonzalez^1^, J. Perez-Sanchez^1^, I. Romera-Peregrina^1^, R. Abellan-Lencina^1^, A. Martinez-Pascual^1^, V. Fuentes-Mila^1^, M. Gonzalez-Romero^1^

###### ^1^Hospital Universitari de Bellvitge, Intensive Care, L' Hospitalet de Llobregat, Spain; ^2^Hospital Universitari de Bellvitge, Laboratory, L' Hospitalet de Llobregat, Spain

####### **Correspondence:** J.C. Lopez Delgado ^_^ Hospital Universitari de Bellvitge, Intensive Care, L' Hospitalet de Llobregat, Spain

**Introduction:** The degree of severe inflammatory response syndrome (SIRS) after CS is related with the development of complications in cardiac surgery (CS). The Inmature Platelets Fraction (IPF) has been recently associated with inflammatory response after sepsis. However, its role related with the injury during CS is unknown.

**Objectives:** To evaluate the role and clinical usefulness of IPF blood levels as a tool to detect patients suffering clinically significant SIRS after CS.

**Methods:** Prospective, observational study in our Surgical ICU in a tertiary-level university hospital. IPF was measured on admission and 24 h after CS, together with clinical data and outcomes including in-hospital mortality. Clinically significant SIRS was defined as need of vasoconstrictors >24 h in the ICU for maintaining appropriate MAP and diuresis.

**Results:** 37 patients were included (62 % male; mean age:69.1 [59.3-78.6] years; BMI:28.1 ± 4.3Kg · m-2). 57 % were active smokers, 69 % suffer from Hypertension, 34 % were diabetic and 60 % dyslipidemia. 74.3 % was elective surgery. 37.8 % (n = 14) suffered SIRS. Univariate analysis showed that the variables associated with SIRS were: age (HR: 1.12;95 % IC: 1.01-1.24; P = 0.034), Cardiopulmonary bypass time (min) (HR:1.03; 95 % IC: 1.002-1.05; P = 0.029), ICU transfusion needs (Red Blood Cells units) (HR: 1.93; 95 % IC: 1.09-3.42; P = 0.024), maximum Urea (First 24 h after CS) (HR: 1.47; 95 % IC: 1.09-1.97; P = 0.011), Difference between IPF 0-24 h (ΔIPF) (HR:3.10; 95 % IC: 1.30-7.39; P = 0.010). Multivariable analysis showed that ΔIPF (HR: 3.78; 95 % IC: 1.099-13.002; P = 0.035) and age (HR: 1.23; 95 % IC: 1.001-1.508; P = 0.048) were factors associated with the presence of SIRS.

Receiver operating characteristic curve showed that ΔIPF had an area under the curve of 0.8224 to predict presence of SIRS. Patients who did not develop SIRS had a negative ΔIPF whereas it was positive in those who developed SIRS (P = 0.0005).

**Conclusions:** Patients who have a positive ΔIPF are prone to develop clinically significant SIRS in our pilot study. Despite our results seem promising, more research is still needed in order to know the role of IPF in SIRS after CS.

**References**

1. De Blasi R, Cardelli P, Costante A, Sandri M, Mercieri M, Arcioni R. Immature platelet fraction in predicting sepsis in critically ill patients. Intensive Care Med. 2013;39(4): 636–43.

2. Monteiro E, Hubert R, Veiga Rodrigues M, Dolci Andreguetto B, Santos T, Pereira Gilberti M, et al. Association of the immature platelet fraction with sepsis diagnosis and severity. Sci Rep. 2015;5:8019.

3. Park S, Ha S, Cho Y, Park C, Jang S, Hong S. Immature Platelet Fraction in Septic Patients: Clinical Relevance of Immature Platelet Fraction is Limited to the Sensitive and Accurate Discrimination of Septic Patients From Non-Septic Patients, not to the discrimination of sepsis severity. Ann Lab Med. 2015;36(1).

#### A758 Frequency of cardiovascular complications after vascular surgery and their impact on patient´s annual prognosis

##### J. Górka^1^, K. Górka^1^, T. Iwaniec^1^, M. Frołow^1^, K. Polok^2^, J. Fronczek^2^, M. Kózka^3^, J. Musiał^1^, W. Szczeklik^1^

###### ^1^Jagiellonian University Medical College, IInd Department of Internal Medicine, Kraków, Poland; ^2^Jagiellonian University Medical College, Kraków, Poland; ^3^St. John Grande Hospital, Vascular Surgery Department, Kraków, Poland

####### **Correspondence:** J. Górka - Jagiellonian University Medical College, IInd Department of Internal Medicine, Kraków, Poland

**Introduction:** Every year, 200 million patients undergo noncardiac surgery worldwide. More than one million of them die within 30 days as a result of perioperative complications. Over the years, venous thromboembolism (VTE) due to deep vein thrombosis (DVT) was considered as the most important and most dangerous complication after surgery, but recently phenomenon of myocardial injury after noncardiac surgery (MINS) definied as elevation in cardiac troponins level caught the attention of researchers.

**Objectives:** The aim of this study was to compare the frequencies of venous (DVT) and arterial (MINS) complications in vascular surgery patients, and to evaluate their impact on the short (30-days) and long-term (1-year) prognosis after surgery.

**Methods:** 167 patients operated due to peripheral arterial disease of lower limbs (PAD) or abdominal aortic aneurysm (AAA) in the Vascular Surgery Unit of a tertiary care hospital in Krakow were enrolled in the study. Every patient had high-sensitivity cardiac troponin T serum level measured before surgery and four times after procedure. In every patient lower extremities venous ultrasound with B-mode compression test was performed three times (before surgery and in the 4^th^ and 7th day after surgery) by experienced physician. All patients were monitored for cardiovascular complications during hospital stay, 30 days and one year follow-up after surgery.

**Results:** DVT was diagnosed in 4 patients after surgery (2.4 %). MINS defined as an increase in TnT after surgery by at least 30 % compared with baseline level and a level of > 0.03 ug/L (normal range 0–0,014 ug/L) was observed in 25 patients (15 %). Annual mortality in patients with MINS was clearly higher than in the group without MINS (6 (24 %) vs 12 (8.5 %); p = 0.02). Among patients with DVT we did not observed deaths in the year of follow-up.

**Conclusions:** Perioperative myocardial injury is more common complication than venous thromboembolism after noncardiac surgery and has a more significant impact on the patient's prognosis during annual observation.

**References**

1. Botto F, Alonso-Coello P, Chan MT et all. Myocardial injury after noncardiac surgery: a large, international, prospective cohort study establishing diagnostic criteria, characteristics, predictors, and 30-day outcomes. Anesthesiology. 2014 Mar;120(3):564–78.

2. Devereaux PJ, Sessler DI. Cardiac Complications and Major Noncardiac Surgery. N Engl J Med. 2016 Apr 7;374(14):1394–5.

**Grant acknowledgement**

The research project was funded by Polish Ministry of Science and Higher Education Diamond Grant DI 2011 023141

#### A759 Red blood cell transfusion and survival after cardiac surgery

##### A. González Pérez, P. Florez Ordoñez, A. Giribet, M.A. Alonso Cuervo, R. Alonso Cuervo, M.A. Rodriguez Esteban, L. Iglesias Fraile, C. Ponte Mittelbrum, G. Muñiz Albaiceta

###### HUCA, ICU, Oviedo, Spain

####### **Correspondence:** A. González Pérez - HUCA, ICU, Oviedo, Spain

**Introduction:** Red blood cell (RBC) transfusion has been associated with increased mortality in patients undergoing cardiac surgery according to various studies.

**Objectives:** The aim of this study is to analyze the impact of RBC transfusion in survival of the intensive care unit (ICU) after cardiac surgery.

**Methods:** Retrospective observational study of a series patients undergoing cardiac surgery between January 2006 and June 2014. A number of demographic and clinical variables taken from the Hospital database are analyzed. A univariate and multivariate logistic regression model was used to study the observed effect and was adjusted for potential confounders / mediators. The odds ratio (OR) with their respective confidence interval of 95 % was reported. P-values less than 0.05 were considered statistically significant.

**Results:** A total of 3449 patients were included in the study. Age: 66.88 +/− 11.9 years old; 65.2 % male.229 patients died during the ICU stay. In the univariate analysis, patients who survive: were more often male, younger: 66.81 +/− 12.64 vs 67.84 +/− 12.43 years (p < 0.05); They had lower ICU stay 6.63 +/− 14.24 vs 10.60 +/− 13.99 days (p < 0.01); less postoperative bleeding in the first 24 hours of ICU admission: 0.55 +/− 1.27 vs 1.1 +/− 1.29 liters (p < 0.01); higher preoperative hemoglobin: 13.64 +/− 5.8 vs 12.31 +/− 2.02 g / dl (p < 0.01); shorter time of cardiopulmonary bypass: 101.66 +/− 23.6 vs 139.17 +/− 66.09 minutes (p < 0.05); less Logistic EUROSCORE : 6.10 +/− 4.33 vs 10.27 +/− 8.58 (p < 0.01) and received fewer RBC transfusion: 1.59 +/− 2.25 vs 3.62 +/− 2.63 packed red blood cells (p < 0.01). In multivariate analysis, RBC transfusion is an independent factor that worse survival in the ICU: OR 1.39 CI (1.31-1.47) p < 0.01, adjusted for age, sex, length of ICU stay, postoperative bleeding, preoperative hemoglobin, CPB duration and EUROSCORE.

**Conclusions:** Transfusion of RBC decreases survival after cardiac surgery in patients admitted to the ICU.

**References**

1. Paone, G., Brewer, R., Theurer, P.F., Bell, G.F., Cogan, C.M., Prager, R.L. **Preoperative predicted risk does not fully explain the association between red blood cell transfusion and mortality in coronary bypass surgery.***J Thorac Cardiovasc Surg*. 2012;143:178–185

2. Paone, G., Likosky, D.S., Brewer, R. et al., **Transfusion of 1 and 2 units of red blood cells is associated with increased morbidity and mortality.***Ann Thorac Surg*. 2014;97:87–94.

3. Shander, A., Goodnough, L.T. **Can blood transfusion be not only ineffective, but also injurious?.***Ann Thorac Surg*. 2014;97:11–14.

**Grant acknowledgement**

Heart team Central of Asturias University Hospital

#### A760 ICU readmission after cardiac surgery: predictive factors, indications and outcome

##### F. Ampatzidou, M. Sileli, G. Kehagioglou, A. Madesis, T. Karaiskos, C. Moursia, H. Maleoglou, K. Leleki, G. Drossos

###### G. Papanikolaou Hospital, Thessaloniki, Greece

####### **Correspondence:** F. Ampatzidou - G. Papanikolaou Hospital, Thessaloniki, Greece

**Introduction:** Knowledge of predictive factors for ICU readmission may lead in earlier identification of deterioration signs in cardiac surgery patients.

**Objectives:** To identify perioperative factors and describe indications and outcome indices associated with ICU readmission after cardiac surgery performed under the use of cardiopulmonary bypass.

**Methods:** A total of 1334 consecutive patients who underwent cardiac surgery during a 3 years period were retrospectively analyzed. Patients who readmitted in ICU consisted group A and were compared with group B, the rest of the cohort. The following factors were compared between 2 groups: gender, severe obesity (BMI > 35), smoking habit, diabetes mellitus, COPD, pre-op pulmonary hypertension (SPAP > 30 mmHg), pre-op ejection fraction < 50 %, cardiopulmonary bypass time (CPB) > 120 min,prolonged mechanical ventilation- defined as > 24 hours (during initial ICU stay),transfusion with >3red blood cell (RBC) units, re-intubation, acute kidney injury(AKI- RIFLE criteria), need of renal replacement therapy, post-op atrial fibrillation and mortality. Statistical analysis was based on x square test.

**Results:** A total of 29 (2.17 %) patients readmitted in ICU. Group A patients were older (68,6 ± 9.8 vs 65,3 ± 10) with higher Euroscore II (4.05 ± 6 vs1.99 ± 2.1).Indications for readmission were : Low cardiac output syndrome (LCOS)(11), respiratory failure(8),cardiac arrest(4), stroke(3), pulmonary edema(2) and tamponade(1). Statistical significant factors for readmission were: severe obesity(10/29, p = < 0.01), COPD(11/29, p = < 0.01), red blood transfusion with > 3 units(15/29, p < 0.01) and prolonged initial mechanical ventilation(4/29, p = 0.018). Statistical significant outcome indices were: re-intubation(12/29, p = < 0.01), AKI(18/29, p = < 0.01), need of renal replacement therapy(8/29, p < 0.01) and post-op atrial fibrillation(19/29, p < 0.01). Mortality was 10.3 % (3/29) vs 2.84 % of group B.

**Conclusions:** COPD, severe obesity, prolonged ventilation and blood transfusions appear to be major factors for ICU readmission. LCOS is the most common indication. Incidence of re-intubation, AKI, need for renal replacement therapy, atrial fibrillation and mortality are higher in this group of patients

**References**

1. Sean van Diepen. Predicting cardiovascular intensive care unit readmission after cardiac surgery Crit Care. 2014; 18(6): 651

#### A761 Sublingual microcirculation post icu cardiac surgery ward identifying the success of furosemide induced recruitment of the microcirculation

##### Z. Uz^1^, Y. Ince^1^, R. Papatella^1^, E. Bulent^1^, P. Guerci^1^, C. Ince,^1^, B. De Mol^2^

###### ^1^Academic Medical Center, University of Amsterdam, Transaltional Physiology, Amsterdam, Netherlands; ^2^Academic Medical Center, University of Amsterdam, Cardio-Thoracic Surgery, Amsterdam, Netherlands

####### **Correspondence:** Z. Uz - Academic Medical Center, University of Amsterdam, Transaltional Physiology, Amsterdam, Netherlands

**Introduction:** The management of tissue perfusion in cardiac surgery with fluid therapy is a challenging task as the parameters used to assess it do not reflect microcirculatory dysfunction. Heterogeneity in blood flow perfusion and abnormalities in capillary density characterize microcirculation dysfunction and can be assessed with incident dark-field illumination (IDF) imaging of the sublingual tissue. The restoration of abnormalities for capillaries can become a target parameter for the administering of fluids in the future in addition to the restoration of the macro circulatory parameters solely used nowadays.

**Objectives:** Visualizing the sublingual microcirculation to assess systemic tissue perfusion and fluid overload in post-operative cardiac surgery patients, to propose a more accurate method to help assess tissue perfusion in patients who undergo surgical procedures.

**Methods:** In this prospective observational study videos of the sublingual microcirculation on post cardiac surgery patients were recorded with the use of the CytoCam (Braedius Medical, Amsterdam, The Netherlands). Data was obtained in day one after the patients left the intensive care unit (ICU). The data was recorded straight after surgery at T0 followed by a two day T1 and T2 measurement. Three videos were recorded at each time point. AVA software v. 3.2 (MicroVision Medical, Amsterdam, The Netherlands) was used to obtain the following microcirculatory parameters: total vessel density (TVD), microcirculatory flow index (MFI), proportion of perfused vessel (PPV), perfused vessel density (PVD). Macro circulatory parameters were also collected in those time points. The weight is measured before the operation (T0), second (T1)and third day(T2) in the post-operative cardiac surgery ward.

**Results:** 10 post-operative cardiac patients are included comprised of 3 females and 7 males. Varied group in cardiac surgery compromising of 5 patients which have undergone coronary bypass graft surgery and 5 aortic valve surgery. The patients came directly from the intensive care to the post-operative cardiac ward with a fluid overload which is currently expressed in a weight gain. All these patients have received daily furosemide and spironolactone to account for the fluid overload. Pre-operative weight T0(76 ± 12.6), T1(78 ± 12) T2 (77 ± 11.7) in 8 patients. There was a significant increase in the weight at T1 compared to baseline (P: 0.0065), and a significant decrease in the weight at T2 compared to T1(P: 0.0193).There was a significant increase in the TVD when comparing the T1 to T0 (24 ± 3.2, 20 ± 3.3 p = 0.0410), and T2 to T0 (26 ± 3.3 20 ± 2.7 P = 0.0005).

**Conclusions:** The TVD increased during the three days whilst the weight decreased possible due to the diuretics. The cytocam is a potential tool to monitor the microcirculation and can indicate and asses fluid overload.

**References**

1. Tanaka S et al. . Crit Care. 2015 Nov 6;19:388.

2. Kara A et al. Curr Opin Anaesthesiol. 2016 Feb;29(1):85–93

**Fig. 109 (abstract A761).**
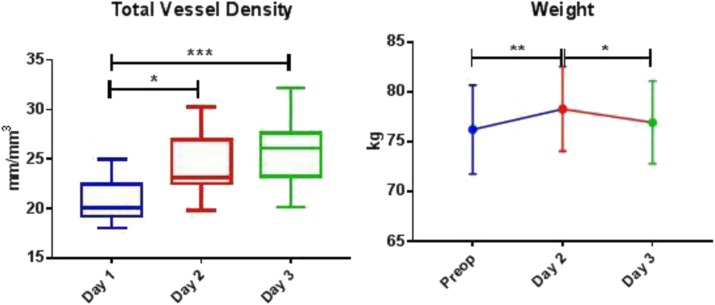
Trends of total vessel density and weight

**Fig. 110 (abstract A761).**
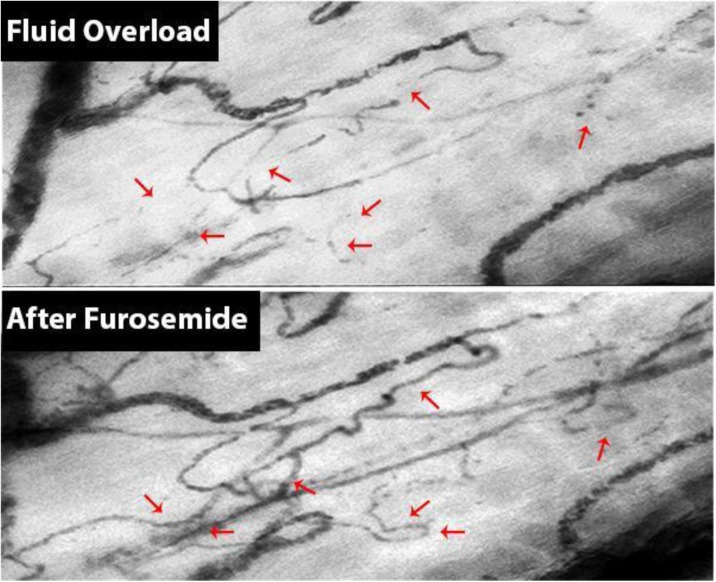
Single spot microcirculation after diuretics

#### A762 Perioperative changes of sodium in cardiac surgery - where to draw the line?

##### V. Vicka, D. Gineityte, D. Ringaitiene, I. Norkiene, J. Sipylaite

###### Vilnius University, Vilnius, Lithuania

####### **Correspondence:** V. Vicka - Vilnius University, Vilnius, Lithuania

**Introduction:** Despite being widely studied, origins of impaired neurologic status after cardiac surgery are not fully understood. One of the commonly reported predisposing factor is the perioperative change in sodium concentration.

**Objectives:** The aim of this study is to determine whether perioperative change of sodium is linked to impaired postoperative neurological status.

**Methods:** The observational study was conducted in a tertiary reference university hospital. Data of cardiac surgery patients were gathered and analyzed. The sodium concentrations were measured before the surgery, upon admission to the ICU and 24 hours later. The mean values and the changes of sodium during these periods were evaluated. The neurological status of the patients was evaluated for one day after the surgery. Impaired postoperative neurological status (INS) was defined as delirium, seizures or impaired consciousness after withdrawal of sedation and extubation of the patient in the first 24 postoperative hours. The perioperative sedation and analgesia was executed according to the routine protocol of the hospital. To determine the most potent predictor of INS a regression analysis of sodium measures was performed. The most potent sodium measurement was further analyzed in a multivariate regression analysis of INS risk factors adjusted for demographic variables, preoperative comorbidities, operative characteristics and early postoperative complications.

**Results:** The data of 262 patients were gathered. Most of the patients were men 65.6 % (N = 172), had a CABG procedure 62.6 % (N = 164) with a low operative risk of median Euroscore II value of 1.62 [0.97-2.35]. The rate of INS in the group was 9.9 % (N = 26). The mean value of preoperative sodium was 139.57 ± 3.00 mmol/L, upon admission to ICU 135.76 ± 3.40 mmol/L and 24 h later 137.76 ± 2.65 mmol/L, resulting in an operative change of 2.1 [0.0-4.4] mmol/L, and 24 h postoperative change of (−) 1.85 [(−) 4.5-0.90] mmol/L. The most potent predictor of INS was the 24 h postoperative change of sodium (OR = 0.818 CI95%: 0.733 - 0.911 p < 0.001). The multivariate regression analysis of INS risk factors revealed the 24 h postoperative change of sodium (OR = 0.80 CI 95 %: 0.71-0.90 p < 0.001) and presence of rethoracotomy as risk factors (OR = 5.28 CI95%: 1.40-19.09 p = 0.014) of INS. The results are presented in the chart below.

**Conclusions:** The INS after cardiac surgery is present in a substantial part of the patients. The most potent predictor of an INS is the 24 h postoperative change of sodium concentration, resulting in a seven-fold risk increase per every drop of 5 mmol/L of sodium.

**Fig. 111 (abstract A762).**
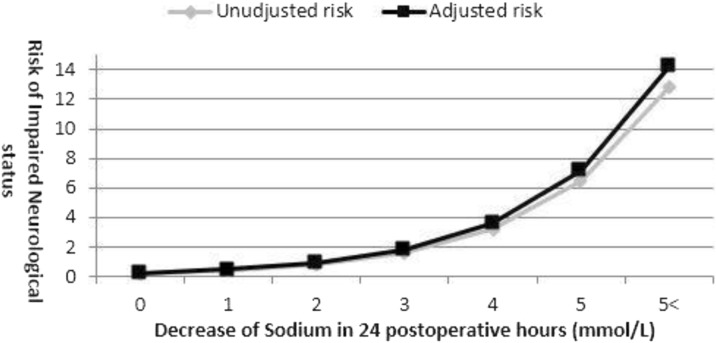
Decrease of sodium in 24 postoperative hours

#### A763 How safe is gelatin? A systematic review and meta-analysis of gelatin-containing plasma expanders versus crystalloids and albumin

##### C. Möller^1^, C. Fleischmann^1,2^, D.O. Thomas-Rueddel^1,2^, V. Vlasakov^1^, B. Rochwerg^3^, P. Theurer^1^, L. Gattinoni^4^, K. Reinhart^1,2^, C.S. Hartog^1,2^

###### ^1^Jena University Hospital, Department for Anesthesiology and Intensive Care, Jena, Germany; ^2^Jena University Hospital, Center for Sepsis Control and Care, Jena, Germany; ^3^McMaster University, Department of Medicine (Division of Critical Care) & Department of Clinical Epidemiology & Biostatistics, Hamilton, Canada; ^4^Università degli Studi di Milano, Dipartimento di Fisiopatologica Medico-Chirurgica e dei Trapianti, Milan, Italy

####### **Correspondence:** C. Möller - Jena University Hospital, Department for Anesthesiology and Intensive Care, Jena, Germany

**Introduction:** Gelatin is a synthetic colloid resuscitation fluid for treatment of hypovolemia. Gelatin efficacy and safety are poorly evaluated. Despite lack of evidence, gelatin use is widespread and increasing in parts of the world. We wanted to systematically review efficacy and adverse outcomes. Given the paucity of data from RCTs, we also included data from non-randomised trials.

**Methods:** Systematic review and meta-analysis of randomised and non-randomised studies comparing gelatin with crystalloid or albumin for treatment of hypovolemia. Adverse outcomes were defined as mortality, allogeneic blood transfusion, acute kidney injury, anaphylaxis and extravascular uptake. Multiple databases were searched systematically without language restrictions until August 2015 (Cochrane Central, Medline, Embase, Indmed, MedCarib, AJO, AIM, IMEMR, WHOLIS, LILACS, WPRIM, IMSEAR, Google Scholar, Grey Literature, German National Library, German Pharmacovigilance Database and Clinical Trials Registries). Risk of bias was assessed using the Cochrane tool for RCTs and the Ottawa Newcastle scale for observational studies. Certainty of evidence was assessed using the GRADE methodology.

**Results:** 60 studies were included, consisting of 30 RCTs with 3629 patients, 8 non-randomised studies with 10827 patients and 22 animal studies. For those receiving gelatin, the relative risks (RR) were as follows: for mortality (RR 1.18, 95 % CI 0.98-1.41, 16 RCTs, 2525 patients; low certainty in evidence), requiring allogeneic blood transfusion (RR 1.25, 95 % CI 0.95-1.64, 8 RCTs, 744 patients; low certainty in evidence), acute kidney injury (RR 1.32, 95 % CI [0.61, 2.87], 3 RCTs, 212 patients, very low certainty in evidence), anaphylactic adverse effects (RR 2.18, 95 % CI 0.87-5.44, 3 RCTs 872 patients, very low certainty of evidence). Mean crystalloid-to-colloid ratio was 1.44 (±0.31, 6 RCTs). Four observational controlled studies (9403 patients, low risk of bias) all found an increased risk for acute kidney injury (AKI) or need for renal replacement therapy (RRT). Between 17 to 31 % of administered gelatin was taken up extravascularly. The mean crystalloid-to-colloid ratio was 1.4.

**Conclusions:** Despite the poor quality of published evidence, this systematic review shows that Gelatin solutions increase the risk of anaphylaxis and may be harmful by increasing mortality, renal failure and bleeding, possibly due to extravascular uptake and coagulation impairment. Plasma expansion with gelatin is not associated with a relevant fluid-sparing benefit. Until well-designed RCTs show gelatin is safe we caution against the use of gelatins since cheaper and safer fluid alternatives are available.

**Grant acknowledgement**

Partially funded by the Federal Ministry of Education and Research (BMBF), Germany, grant no: 01EO1002

#### A764 Impact of red blood cell transfusion in health-related quality of life after cardiac surgery

##### A. González Pérez^1^, J. Zanabili Al Sibai^2^, P. Martinez Camblor^3^, P. Alvarez Fernandez^4^, J.M. García Gala^2^, J. Silba Guisasola^5^, G. Muñiz Albaiceta^1^

###### ^1^Hospital Universitario Central de Asturias, Intensive Care Unit 1–2, Oviedo, Spain; ^2^Hospital Universitario Central de Asturias, Hematology Department, Oviedo, Spain; ^3^University of Oviedo, Oviedo, Spain; ^4^Hospital Universitario Central de Asturias, Intensive Care Unit 3, Oviedo, Spain; ^5^Hospital Universitario Central de Asturias, Cardiac Surgery, Oviedo, Spain

####### **Correspondence:** A. González Pérez - Hospital Universitario Central de Asturias, Intensive Care Unit 1–2, Oviedo, Spain

**Introduction:** Red blood cell (RBC) transfusion has been associated with increased in-hospital morbidity and mortality after cardiac surgery. However, the impact on quality of life has not been fully clarified.

**Objectives:** The aim of this study is to analyze whether transfusion of RBC has an impact on health-related quality of life (HRQOL).

**Methods:** Prospective observational study of 205 patients undergoing cardiac surgery (valve replacement and / or coronary revascularization) between January and April of 2015 at a University Hospital. Clinical data relating to patient comorbidities, surgery and postoperative course were collected, including hemoglobin pre and post-surgery and the number of blood products transfused perioperatively. At 6 months after discharge, was applied EUROQUOL- 5D questionnaire for assessing the HRQOL through telephone interview. Significant variables in the univariate analysis were entered into a multivariate logistic regression model to calculate the Odds Ratio (OR) with confidence interval 95 %.

**Results:** Questionnaire response 178 patients (61.2 % males, age 70.7 ± 9.8). They were divided into two groups: group 1: problems in some dimension of HRQOL of EUROQOL 5D and group 2: without problems. In the Univariate analysis,group 2 patients (with problems) presented the following characteristics: were older (p < 0.01); were mostly males (p < 0.01); lower preoperative hemoglobin (p < 0.05), received more RBC (p < 0.01) as well as a higher Hospitality stay (p < 0.01). Patients receiving more RBC have a further deterioration in HRQOL (OR 1.23, 95 % CI 1.07 to 1.41, p = 0.004) .In multivariate analysis, transfusion of more RBC causes more deterioration in HRQOL after adjusting for age and gender OR (1.18, 95 % CI 1.04 to 1.34), p = 0.012); by preoperative and postoperative hemoglobin (OR 1.19, 95 % CI 1.04 to 1.37, p = 0.013) but not by stay

(OR 1.11, 95 % CI 0.95 to 1.28), p = 0.184) considering stay a mediator in the causal chain.

**Conclusions**

Transfusion of packed red blood cells reduces HRQOL after cardiac surgery.

**References**

1. Cladellas M, Bruguera J, Comin J, Vila J, de Jaime E, Marti J, et al. Is pre-operative anaemia a risk marker for in-hospital mortality and morbidity after valve replacement?. Eur Heart J. 2006; 27: 1093–9.

2. Persistent effect of red cell transfusion on health-related quality of life after cardiac surgery. Koch CG^1^, Khandwala F, Li L, Estafanous FG, Loop FD, Blackstone EH.

Ann Thorac Surg. 2006 Jul;82(1):13–20.

**Grant acknowledgement**

All contributors.

### Systemic diseases and metabolism

#### A765 Energy expenditure measured using indirect calorimetry after cardiovascular surgery in ventilated postoperative patients

##### T. Tamura, T. Yatabe, I. Miyajima, K. Yamashita, M. Yokoyama

###### Kochi Medical School, Department of Anesthesiology and Intensive Care, Nankoku, Japan

####### **Correspondence:** T. Tamura - Kochi Medical School, Department of Anesthesiology and Intensive Care, Nankoku, Japan

**Introduction:** Given that surgical procedures have become minimally invasive, we thought that postoperative energy expenditure would be decreased. In fact, the resting energy expenditure (REE) measured by an indirect calorimeter after minimally invasive esophagectomy in the intensive care unit (ICU) was significantly lower than the basal energy expenditure (BEE) estimated using the Harris-Benedict equation (HBE) [1]. However, postoperative REE after cardiovascular surgery remains unclear.

**Objectives:** We hypothesized that the postoperative REE would be lower than the BEE in patients in the ICU who had undergone cardiovascular surgery and conducted a prospective observational study to investigate it.

**Methods:** The ethics committee from our hospital approved this prospective study. This study included patients who underwent cardiovascular surgery in our hospital from September 2013 to March 2015. After surgery, the REE was measured using an Engström device (GE Healthcare Japan, Tokyo, Japan), which consists of a ventilator and an indirect calorimeter, and was measured from the time from ICU admission to extubation or at 9 AM on postoperative day 1. The patients received pressure-controlled ventilation (tidal volume: 6–10 mL/kg) in the ICU under dexmedetomidine and propofol sedation. The ventilation setting and the doses of these sedative agents were adjusted by each physician in accordance with the Richmond Agitation-Sedation Scale (RASS). Patients received an infusion of a 10 % glucose solution at a rate of 192 kcal/day after admission into the ICU. We maintained the blood glucose level between 110 to 180 mg/dL using an artificial pancreas (STG-55, Nikkiso, Tokyo, Japan). The following data were obtained: age, body weight, height, RASS, and REE for each hour during ventilation. The REE and the BEE estimated using the HBE were compared using the paired *t*-test.

**Results:** Forty-seven patients (23 female and 24 male) were enrolled in this study. The mean age, body weight, and height were 73 ± 10 years (mean ± standard deviation), 55 ± 10 kg, and 155 ± 10 cm, respectively. Seventy three percent of patients received a cardiopulmonary bypass (CBP). The average temperature and RASS during ventilation were 38.2 ± 3.3 °C and −3 ± 1, respectively. The average REE during ventilation was 1314 ± 148 kcal/day (23.9 ± 6.8 kcal/kg/day). The average BEE was 1147 ± 148 kcal/day. The average REE during ventilation was significantly higher than the BEE (114 ± 35 % of the BEE, p = 0.02). In addition, the REE was not significantly different between patients who received a CBP and patients who did not receive a CBP.

**Conclusions:** The REE after cardiovascular surgery was significantly higher than the BEE regardless of whether a CBP was performed. In postoperative patients, it is important to note the difference in surgical procedures when estimating the optimal target calorie level for nutrition therapy.

**References**

[1] Asia Pac J Clin Nutr. 2014;23:555–9.

**Grant acknowledgement**

None.

#### A766 Outcome of cardiac surgery patients with a history of connective tissue disease

##### F. Ampatzidou^1^, G. Kehagioglou^2^, E. Dalampini^2^, M. Nastou^2^, A. Baddour^2^, A. Ignatiadis^2^, T. Asteri^2^, G. Drossos^2^

###### ^1^G. Papanikolaou Hospital, Cardiac Surgery ICU, Thessaloniki, Greece; ^2^G. Papanikolaou Hospital, Cardiac Surgery, Thessaloniki, Greece

####### **Correspondence:** F. Ampatzidou - G. Papanikolaou Hospital, Cardiac Surgery ICU, Thessaloniki, Greece

**Introduction:** Connective tissue diseases represent a rare, heterogeneous group of diseases that commonly impair the function of many organs.

**Objectives:** Aim of this study was to determine whether a history of connective tissue disease has any impact on postoperative morbidity and mortality in patients who undergo cardiac surgery under the use of cardiopulmonary bypass.

**Methods:** We retrospectively reviewed our electronic database of 1618 consecutive cardiac surgery patients from May 2012 to March 2016. We analyzed the postoperative outcome indices of cardiac surgical patients with a history of connective tissue disease.

**Results:** A total of 15 (5 females) patients were identified (group A): 12 rheumatoid arthritis, 2 ankylosing spondylitis, 1 psoriatic arthritis. Oral steroids were used in 6 and methotrexate in 3 patients. The following operations were performed (1 emergency): 8 valve surgery, 5 coronary artery bypass and 2 operations on thoracic aorta. Comparing with the rest of the cohort (group B), the study group patients: were older (69,6 ± 7 vs 65,2 ± 10), their average pre-op mean ejection fraction was 57,3 ± 10 % vs 55,6 ± 11 % and their pre-op eGFR(MDRD) was 76,2 ± 3 vs 73,7 ± 23 mL/min/1.73 m^2^. Pulmonary hypertension- defined as pre-op doppler systolic pulmonary pressure > 30 mmHg- was found in 5/15 patients (36,6 % vs 22,49 %, p = 0,34). Median duration of mechanical ventilation was 11 hours for group A vs 7 hours for group B. Transfusion with > 3 red blood units was required in 5/15 (36,6 % vs 23,4 %, p = 0,36). Post-op low cardiac output syndrome developed in 2/15 (13,3 % vs 5,49 %, p = 0,43). Acute kidney injury defined by RIFLE criteria complicated 2/15 patients (13,3 % vs 15,6 %, p = 0,78). Post-op atrial fibrillation developed in 7/15 (46,6 % vs 33,6 %, p = 0,31). One patient required non invasive ventilation for post-op respiratory failure. Median ICU days were 2 in both groups. No patient of the study group developed sternal wound infection or had prolonged (>24 hours) mechanical ventilation while hospital mortality was 0 %.

**Conclusions:** According to the results of this single-center retrospective study, cardiac surgery procedures could be performed safely in patients with a history of connective tissue disease.

**References:** Lee J. Outcome of patients with connective tissue disease requiring intensive care for respiratory failure. Rheumatol Int. 2012;32(11):3353–8

#### A767 Red cell distribution width at hospital discharge and post-hospital outcomes in ICU survivors with chronic liver disease

##### K.E. Hathorn^1^, S.W. Purtle^2^, C.M. Horkan^1^, F.K. Gibbons^3^, K.B. Christopher^4,5^

###### ^1^Brigham and Women's Hospital, Department of Medicine, Boston, USA; ^2^University of Colorado, Division of Pulmonary Sciences and Critical Care Medicine, Boulder, USA; ^3^Massachusetts General Hospital, Pulmonary and Critical Care Medicine, Boston, USA; ^4^Brigham and Women's Hospital, Renal Division, Boston, USA; ^5^Brigham and Women's Hospital, Channing Division of Network Medicine, Boston, USA

####### **Correspondence:** K.E. Hathorn - Brigham and Women's Hospital, Department of Medicine, Boston, USA

**Introduction:** Red Cell Distribution width (RDW) is associated with mortality and bloodstream infection risk in the critically ill. In survivors of critical care with chronic liver disease (CLD) it is not know if RDW can predict subsequent risk of all-cause mortality following hospital discharge.

**Objectives:** We hypothesized that an increase in RDW at hospital discharge in CLD patients who received critical care would be associated with increased mortality following hospital discharge.

**Methods:** We performed a two center observational study of patients treated in medical and surgical intensive care units in Boston, Massachusetts. We studied 4,442 patients, age ≥ 18 years, who had chronic liver disease, received critical care between 1998 and 2012 and survived hospitalization. The exposure of interest was RDW within 24 hours of hospital discharge and categorized *a priori* as ≤13.3 %, 13.3-14.0 %, 14.0-14.7 %, 14.7-15.8 %, 15.8-17.0 % and >17.0 %. The primary outcome was all cause mortality in the 90 days following hospital discharge determined using the US Social Security Administration Death Master File. Adjusted odds ratios were estimated by multivariable logistic regression models with inclusion of covariate terms for age, race, gender, Deyo-Charlson Index, patient type (medical versus surgical), sepsis and number of organs with acute failure.

**Results:** The cohort patients were 62.6 % male, 21.4 % nonwhite and 35.7 % surgical. 14.9 % of the cohort had sepsis and the mean age was 59 years. Mean(SD) MELD score was 15.0(8.0). 90-day post discharge mortality was 10.3 %. RDW at hospital discharge was a robust predictor of all cause post discharge mortality and remained so following multivariable adjustment. Patients with a discharge RDW 14.7-15.8 %, 15.8-17.0 % or >17.0 % have an adjusted OR of 90-day post discharge mortality of 2.31 (95%CI, 1.16-4.62; P = 0.017), 3.24 (95%CI, 1.65-6.38; P = 0.001) or 6.06 (95%CI, 3.13-11.71; P < 0.001) relative to patients with a discharge RDW ≤13.3 %. Estimates were similar with additional adjustment for MELD score. Discharge RDW has moderate discrimination for 90-day post discharge mortality (AUC = 0.76; 95 % CI 0.74-0.78). Additionally, patients with a discharge RDW 15.8-17.0 % or >17.0 % have an adjusted OR of 30-day readmission of 1.60 (95%CI, 1.13-2.26) or 1.41 (95%CI, 1.01-1.98) relative to a discharge RDW ≤13.3 %. Finally, patients with a discharge RDW 15.8-17.0 % or >17.0 % have an adjusted OR of care facility placement of 1.50 (95%CI, 1.11-2.02) or 1.45 (95%CI, 1.08-1.94) relative a discharge RDW ≤13.3 %.

**Conclusions:** In CLD patients treated with critical care who survive hospitalization, an elevated RDW at discharge is a robust predictor of subsequent mortality, hospital readmission and placement in a care facility. Increased RDW at discharge likely reflects the presence of ongoing inflammation or oxidative stress which may explain the observed impact on CLD patient outcomes following hospital discharge.

#### A768 Early increase in protein intake reduces mortality in underweight critically ill patients

##### M.V. Viana^1,2^, T.A. Tonietto^1,2^, L.A. Gross^3^, V.L. Costa^3^, A.L.J. Tavares^3^, B.O. Lisboa^3^, R.B. Moraes^1^, S.R. Vieira^4^, L.V. Viana^5^, M.J. Azevedo^6^

###### ^1^Hospital de Clínicas de Porto Alegre, Intensive Care Unit, Porto Alegre, Brazil; ^2^Hospital Nossa Senhora Conceição, Intensive Care Unit, Porto Alegre, Brazil; ^3^Universidade Federal do Rio Grande do Sul, Faculdade de Medicina, Porto Alegre, Brazil; ^4^Universidade Federal do Rio Grande do Sul, Faculdade de Medicina - Department of Intensive Care, Porto Alegre, Brazil; ^5^Universidade Federal do Rio Grande do Sul, Faculdade de Medicina - Department of Medical Nutrition, Porto Alegre, Brazil; ^6^Universidade Federal do Rio Grande do Sul, Faculdade de Medicina - Department of Endocrinology, Porto Alegre, Brazil

####### **Correspondence:** M.V. Viana - Hospital Nossa Senhora Conceição, Intensive Care Unit, Porto Alegre, Brazil

**Introduction:** Critically ill patients with body mass index (BMI) lower than 20 kg/m^2^ have worse outcomes than normal or overweight patients possibly because underweight is a marker of malnutrition. It has been suggested that in malnourished critically ill patients an early increased intake of calories and protein might improve their prognosis.

**Objectives:** To evaluate the impact of nutritional support, especially calories and protein, on specific outcomes - need of tracheostomy, ICU readmission, and intra-hospital mortality - in underweight critically ill patients.

**Methods:** Prospective, two-center, observational study, was designed to asses the effect of nutritional intake in underweight critically ill patients. All patients consecutively admitted (November 2015 to March 2016) to general intensive care units (ICU) with IMC < 20 kg/m^2^ were included. Exclusion criteria were: palliative care, exclusively oral nutrition, pregnancy, life expectancy < 24 h, and ICU readmission. Patients had their nutritional intake evaluated after ICU admission between days 2 and 3 (1^st^ evaluation). Another evaluation was performed between days 5 and 7 (2^nd^ evaluation) if patient had not been discharged from ICU, was not on exclusively oral nutrition, or on palliative care. Patients were divided into groups according calorie intake (group A:< 20 kcal/kg/day; group B:≥20 kcal/kg/dia) and protein intake (group C:< 1 g protein/g/day; group D:≥ 1 g protein/kg/day). Patients were followed in the hospital until their death or discharge.

**Results:** The hospital mortality rate of 83 included patients was 55.4 % after 17 (10–32) days of follow-up. There was an increment in calories (19.6 ± 9.7 to ± 27.6 ± 11.2; p < 0.001) and protein (0.9 ± 0.6 to 1.33 ± 0.7; p < 0.001) intakes between 1^st^ and 2^nd^ evaluation. The caloric intake (Kcal/day) both in the 1^st^ (19.2 ± 9.2 vs. 16.3 ± 10; p = 0.189) and 2^nd^ evaluation (27.8 ± 10 vs. 27.5 ± 11.9; p = 0.916) did not differ between survivors and no survivors; there was a trend for a higher protein intake (g/Kg/day) in survivors in the 1^st^ evaluation (0.96 ± 0.56 vs. 0.69 ± 0.62; p = 0.051). Mortality did not differ according caloric intake in the 1^st^ (Table 79) and 2^nd^ evaluation. Patients who received >1 g of protein/day (Group D) in the 1^st^ evaluation (Table 79) had lower mortality than those who received less protein (Group C), but not in 2^nd^ evaluation. Frequency of tracheostomies was higher in patients who received more calories (group B) and protein(Group D) only in the 1^st^ evaluation. Multivariate logistic models confirmed (OR, 95%CI) that protein intake was negatively associated with mortality [protein intake 0.43(0.18-0.99); SAPS3 1.07(1.02-1.11)] and with need of tracheotomy [protein intake 3.06(1.03-9.07); SAPS3 0.94(0.90-9.99)] even after adjustment for illness severity. ICU readmission did not differ among groups.

**Conclusion:** In underweight critically ill patients an early high protein intake at ICU admission has a protective role for mortality.

**Grant acknowledgement**

FIPE - HCPA

**Table 79 (abstract A768).** Clinical characteristics and nutritional support

	Caloric intake			Protein intake		
	Group A (<20kcal/kg) n=50	Group B (≥20kcal/kg) n=33	P	Group C (<1g protein/kg) n=48	Group D (≥1g protein/kg) n=35	P
Age (ys)	55.7±17.6	54.8±18.6	0.839	54 ± 18.4	57.3±17.3	0.409
Male (%)	32(64)	18(54.5)	0.386	32 (66.7)	18 (51.4)	0.161
SAPS 3	70.6±13.2	68.2±9	0.471	71±14.9	66±14.3	0.121
Nutric ≥ 5 (%)	25(50)	16 (48.5)	0.893	25 (52.1)	16 (39)	0.567
Tracheostomy (%)	5(10)	12(37.5)	**0.030**	5 (10.4)	12 (35.3)	**0.006**
ICU readmission (%)	6(12)	3(9.4)	0.711	6(12.5)	3(8.8)	0.729
Mortality (%)	31(62)	15(45.5)	0.138	31 (64.6)	15(42.9)	**0.049**

#### A769 Quality indicators of nutritional therapy measured by dieticians may be associate with the outcome of the critical care patient

##### G.D. Ceniccola^1,2^, R.S.F. Pequeno^1^, T.P. Holanda^2^, V.S. Mendonça^1^, W.M.C. Araújo^2^, L.S.F. Carvalho^3^

###### ^1^Hospital de Base do Distrito Federal, Residência em Nutrição Clínica, Brasília, Brazil; ^2^Universidade de Brasília, Departamento de Nutrição, Brasília, Brazil; ^3^Universidade Estadual de Campinas, Universidade de Ciências Médicas, Campinas, Brazil

####### **Correspondence:** G.D. Ceniccola - Universidade de Brasília, Departamento de Nutrição, Brasília, Brazil

**Introduction:** Nutritional risk and malnutrition must be screened at admission in intensive care units (ICU) because these patients usually benefit from early nutritional therapy^1^. Nutritional parameters should be monitored by quality indicators (QI), but their goals and association with patient outcome are still unknown.

**Objectives:** To estimate the impact of malnutrition, nutritional risk and high caloric deficit on mortality at a 60 days follow-up in a general ICU population.

**Methods:** Consecutive adult patients who remained in the ICU for greater then 48 hours were included and prospectively followed. Data were abstracted from electronic health records (EHR) by trained ICU dietitians on their sex, age, APACHE II and baseline nutrition assessment at admission^2^. Daily nutrition information was collected on the type (i.e. EN, PN, oral, none) and amount of nutrition received (total calories) from ICU admission for a maximum of the first 7 days (EN only) unless death or discharge occurred sooner. Heavy caloric deficit occurred when < 25 % of the caloric needs was achieved in the first ICU week. Patients were followed while in hospital and their outcomes determined at 60 days after ICU admission. Online forms were elaborated to enter abstracted data with a secure web-based tool (GoogleDocs, © 2012).

**Results**

A total of 236 EHR were verify from 2014 to 2015 in 4 this audit, 85.6 % were screened for Nutritional risk of that 91.1 % had nutritional risk according to NRS 2002 tool^2^, 85.2 % were accessed with ASPEN-ADA^3^ tool, diagnosing 23.4 % of malnutrition. 91.1 % of the EHR had information on the nutrients received, heavy caloric deficit was verif in 27.4 % of the EHR with this information. Binary logistic regression evidenced risk of mortality at 60 days 8.01 times higher (95%CI 1.86 - 34.7, Wald = 7.8, p = 0.005) in patients malnourished and with heavy caloric deficit in the first ICU week at the same time when compared with patients without these two risk factors (controlled by sex, age and APACHE II). Either malnutrition or heavy caloric deficit when present increases the mortality risk in 5.83 times (95%CI 1,33 - 25.4, Wald = 5.5, p = 0.019). Nutritional risk was not related with mortality at 60 days.

**Conclusion:** The QI presented are potentially useful nutritional markers and could implement ICU routine, the presence of a dietitian in the multidisciplinary team can facilitate that task. The combination of malnutrition and heavy caloric deficit were relevant nutritional QI associated with ICU mortality regardless of sex, age and Apache II. More studies are needed to establish goals for these QI and to develop this field.

**References**

1- Doig GS, et al. Injury;2011; 42(1):50

2- Kondrup J. *Clin Nutri.* 2003;22(3):321

3- White JV, et al. JPEN 2012;36(3):275

**Grant acknowledgement**

This project used own funding.

**Table 80 (abstract A769).** Demographics

Characteristic	Non survivors (mean, 95%CI)	Survivors (mean, 95%CI)	P - value (Mann-Whitney test)
Age (n= 236)	59.31; 55.69−62.93	50.76; 47.99−53.54	0.001
Body Mass index (n= 217)	26.51; 25.05−27.96	25.84; 25.12−26.56	0.865
Apache II (n= 232)	23.22; 21.40−25.03	17.03; 15.69−18.36	0.0001
Nutritional Risk Screening 2002 (n= 202)	3.82; 3.55−4.08	3.49; 3.30−3.68	0.031
Length of ICU stay (n= 234)	19.31 15.46–23.17	31.11; 25.92–36.30	0.01
Length of hospital stay (n= 227)	25.91; 22.06–29.76	60.31; 53.69–66.94	0.0001

**Fig. 112 (abstract A769).**
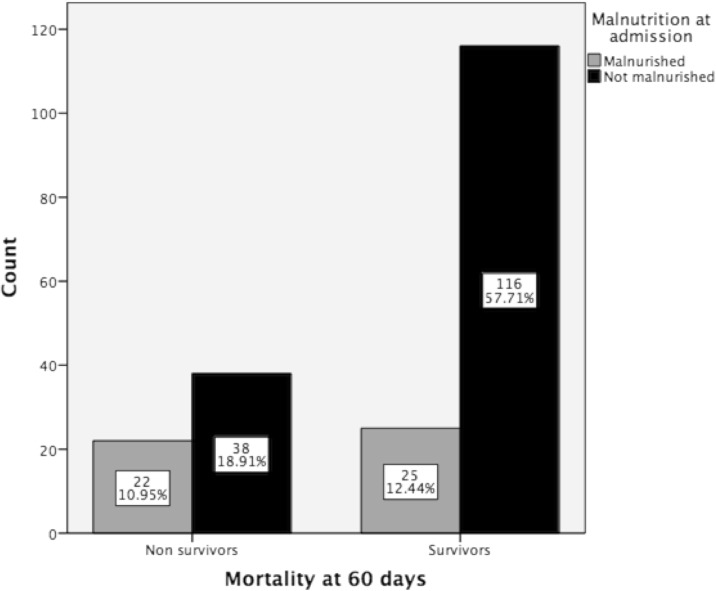
Malnutrition at admission

**Fig. 113 (abstract A769).**
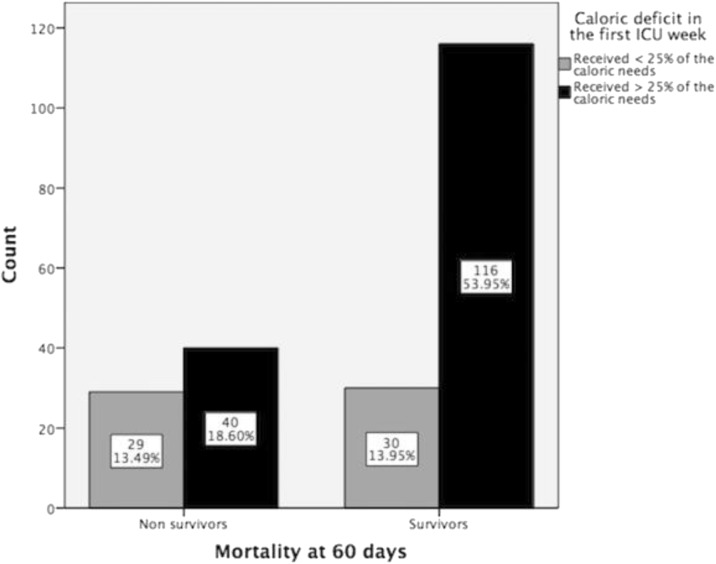
Caloric deficit in the first week of ICU

#### A770 Comparison of fasting practices in three critical care units

##### E. Segaran, L. Vickers, K. Brinchmann, I. Wignall, F. Rubulotta

###### Imperial College Healthcare NHS Trust, Adult Critical Care, London, UK

####### **Correspondence:** E. Segaran - Imperial College Healthcare NHS Trust, Adult Critical Care, London, UK

**Introduction:** Enteral nutrition (EN) is currently the route of choice for feeding critically ill patients (1); however, due to the unpredictable nature of critical illness, the delivery is frequently disrupted. Common reasons for inadequate delivery are gastrointestinal intolerance, fasting for diagnostic procedures, surgery and airway management (2). Cumulative calorie deficits contribute to malnutrition (3) and poor outcome including increased infections, length of ICU /hospital stay and mortality (4). There is no recognised guidance on the length of time that should elapse between stopping EN and commencing anaesthetic procedures. We developed and implemented our own fasting guidelines to address the EN interruptions on our units. In our trust, only radiologists or ICU consultants are allowed to confirm placement. We believe that this is negatively influencing tube confirmation times and contributing to EN under delivery.

**Objectives:** To compare specific interruptions to EN in 3 different adult intensive care units (ICU), assessing impact on nutrition delivery·

- To examine compliance with ICU fasting guidelines on stopping and restarting enteral nutrition prior to operative and non-operative procedures

**Methods:** An audit over 1 month in 3 adult different ICU's in a large London trust, each with its own unique specialities.

**Results:** Data was collected on 59 patients. See Table 81 for demographic details. The most frequent reasons for stopping EN were removal and confirmation of nasogastric tubes, extubation and cancelations. Each was associated with an average deficit of 800kcals and 35 g protein per episode. The nutritional deficits relating to each episode of fasting are detailed in Tables 82 and 83.

**Conclusions:** Although the case mix was different, each unit experienced frequent fasting impacting on enteral nutrition delivery. The reasons were similar for all units. Each fasting period was associated with considerable calorie and protein deficit. Despite having ICU specific fasting guidelines in place, violations were observed for all procedures especially extubation and tracheostomy, where the fast far exceeded 4 hours. Restarting EN was also extended waiting on NGT confirmation after the procedure. Prolonged fasting and delays in restarting EN suggests limited appreciation of nutritional deficits and associated negative impact of fasting by ICU staff.Further education to our ICU staff is recommended on fasting guidance to increase awareness and compliance. In addition, the current practice for NGT confirmation should be reviewed with the view to allowing ICU trainees to confirm position following appropriate training.

**References**

1. McClave S et al. JPEN 2016;4 159–211

2. De Jonghe B et al. Crit care med 2001;29(1):8–12

3. Alberda C et al. Inten Care Med 2009;35 (10):1728–37

4. Villet S et al. Clinical Nut 2005;24(4):502–9

**Grant Acknowledgement**

No funding was sought for this project

**Table 81 (abstract A770):** Patient demographics

	St Mary's Hospital	Hammersmith Hospital	Charing Cross Hospital
No. of patients Total of 59	21/59	14/59	24/59
Age years- (median)	57	59	61
Gender -number *Female, + Male	4 F*, 17M+	2F, 12M	12F, 12M
Diagnosis:	Number (%)	Number (%)	Number (%)
Trauma/ neurosurgery	11 (52)	0	3 (13)
Medical / Sepsis	6 (29)	9 (64)	13 (54)
Surgery (GI, pancreatic, cardiac, vascular)	2 (10)	4 (29)	6 (25)
Renal or Liver failure	2 (10)	1 (7)	2 (8)

**Table 82 (abstract A770):** Nutrition deficits per episode of fasting

Procedure	Total time off feed Hours Median (IQR)	Energy deficit per episode Kcal Median (IQR)	Protein deficit per episode Grams Median (IQR)
St Mary´s Hospital			
NGT insertion & confirmation	15 (11–17)	1164 (1078–1503)	62 (42–70)
Extubation	14 (12–31)	1008 (983–1980)	47 (46–72)
Tracheostomy (Percutaneous & surgical)	12 (10–20)	892 (832–1476)	54 (47–69)
Hammersmith Hospital			
NGT insertion & confirmation	9 (8–13)	800 (655–1400)	36 (25–67)
Extubation	12 (6–14)	825 (482–966)	30 (20–42)
Tracheostomy (Percutaneous & surgical)	11 (11–14)	1100 (958–1206)	46 (42–51)

**Table 83 (abstract A770).** Nutrition deficits per episode of fasting

Procedure	Total time off feed Hours Median (IQR)	Energy deficit per episode Kcal Median (IQR)	Protein deficit per episode Grams Median (IQR)
Charing Cross Hospital			
NGT insertion & confirmation	12 (4–15)	591 (274–1091)	27 (13–45)
Extubation	10 (8–12)	647 (504–741)	30 (26–34)
Tracheostomy (Percutaneous & surgical)	11 (9–12)	874 (705–934)	40 (32–43)

#### A771 How much fibre should we give to the critically ill patients?

##### I. De Brito-Ashurst

###### Royal Brompton and Harefield NHS Foundation Trust, London, UK

**Introduction:** Diarrhoea is a common complication in patients receiving enteral nutrition in the intensive care unit (ICU). International organizations[1]'[2] recommend fibre-based formula to control diarrhoea. Nevertheless, there are reported inconsistencies on the benefits of fibre in reducing diarrhoea. [3] Furthermore, the recommended amount of fibre for the ICU patient group remains controversial.

**Objective:** To establish the optimum amount of dietary fibre for regular bowel function in the ICU population.

**Methods:** A retrospective review of all patients on ETF for > 5 days between January and December/2015. Inclusion criteria was ETF with fibre-based formula and exclusion was non-fibre ETF. Feeding formula contained mixed fibre with fructooligosaccharides. A quantitative score based on stool volume and consistency was used for daily assessment of diarrhoea; diarrhoea was defined as a score ≥15. [4] Patients were split into two groups: Group D (GD) - diarrhoea and Group N (GN) - normal bowel function. Parametric tests were used to assess the difference between the mean faecal scores and fibre intake of fibre between the groups.

**Results:** Two hundred and fifty one patients fitted the inclusion criteria. Patients were male (153/251) and age 54 ± 17 years. Data are presented as mean and standard deviation (SD). Faecal scores were 9.9 (1.6) and 17.4 (1.8) (P < 0.001) for GN (216/251) and GD (35/251) respectively. Fibre intake was significantly higher on GD at 21.64 g/day (7.2) compared to GN at 11.8 (6.6) (P < 0.002). Flatulence and abdominal pain was only reported in the group D, on patients with a fibre intake >25 g/day. Nutritional intake was similar for both groups that received >75 % of caloric and protein prescription. Both groups were on prokinectics; GD was on metoclopramide (36) and erythromycin (8) and GN on 39/6 for both prokinectics.

**Conclusion:** Fibre intake has an impact on bowel function. A high fibre intake is associated with diarrhoea and flatulence. The higher rate of flatulence is likely to result from fermentation of the soluble fibre in the colon. The optimum fibre intake for the ICU group is < 20 g/day.

**References**

[1] Kreymann, K.G., et al., *ESPEN Guidelines on Enteral Nutrition: Intensive care.* Clin Nutr, 2006. 25(2): p. 210–23.

[2] McClave SA, Taylor BE, Martindale RG, et al. Guidelines for the Provision and Assessment of Nutrition Support Therapy in the Adult Critically Ill Patient: Society of Critical Care Medicine (SCCM) and American Society for Parenteral and Enteral Nutrition (A.S.P.E.N.). JPEN J Parenter Enteral Nutr. 2016;40(2):159–211.

[3] Zaman MK, Chin K-F, Rai V, et al. Fiber and prebiotic supplementation in enteral nutrition: a

 systematic review and meta-analysis. *World J Gastroenterol* 2015 7; 21(17): 5372–5381

[4] Whelan K, Judd PA, Taylor MA. Assessment of fecal output in patients receiving enteral tube feeding: validation of a novel chart. Eur J Clin Nutr. 2004;58(7):1030–1037.

#### A772 Effects of lipid emulsions based on n-3 polyunsaturated fatty acids on metabolic and infectious complications compared with MCT/LCT lipid emulsion in critically ill patients receiving parenteral nutrition

##### R. del Olmo^1^, M.J. Esteban^2^, C. Vaquerizo^3^, R. Carreño^3^, V. Gálvez^3^, G. Kaminsky^3^, B. Nieto^3^, M. Fuentes^3^, M.A. De la Torre^3^, E. Torres^3^, A. Alonso^3^, C. Velayos^3^, T. Saldaña^3^, A. Escribá^3^

###### ^1^Fuenlabrada University Hospital, Intensive Care Unit, Madrid, Spain; ^2^Fuenlabrada University Hospital, Pharmacology Department, Madrid, Spain; ^3^Fuenlabrada University Hospital, Madrid, Spain

####### **Correspondence:** R. del Olmo - Fuenlabrada University Hospital, Intensive Care Unit, Madrid, Spain

**Introduction**: Several clinical trials have shown beneficial effects of parenteral lipid emulsions based on n-3 polyunsaturated fatty acids (PUFAs) (derived from fish oil) in intensive care unit (ICU) patients receiving parenteral nutrition (PN). Many of these studies have been criticized for the heterogeneity of the patient´s population. Recently, controversy has arisen due to conflicting results on clinical outcomes.

**Objectives:** To assess the effects of a lipid emulsion based on n-3 PUFAs compared with a MCT (medium-chain triglycerides)/LCT (long-chain triglycerides) lipid emulsion on liver dysfunction, infectious complications and other clinical outcomes in a cohort of critically ill patients with Apache II > 17 and length of ICU stay of at least 5 days.

**Methods:** Observational retrospective single-centre cohort study in a general ICU from September 2014 to July 2015. Patients in the fish oil cohort (n 14) received n-3 PUFAs-enriched PN and patients in the standard PN cohort (n 10), a MCT/LCT lipid emulsion. Baseline characteristics of patients, energy and fat intake, laboratory parameters (including liver function tests), new infections, insulin requirements, days on mechanical ventilation, length of ICU and hospital stay and mortality were recorded. Significance level was defined at a p value of less than 0.05.

**Results: B**oth groups had similar baseline characteristics : age (63 ± 17 vs 63 ± 12), Apache II (18 ± 6 vs 18 ± 7), SAPS 3 (66 ± 12 vs 61 ± 15), prealbumin at admission (11 mg/dl ± 10 vs 9 ± 6). The variable results are shown in Tables 84 and 85.

**Conclusions:** The use of a fish oil based emulsion in PN had no effect on new infections, insulin requirements, hospital length of stay and hospital mortality when compared with MCT/LCT based emulsion in ICU patients with Apache II score > 17. Bilirubin concentrations and ICU length of stay were lower in the MCT/LCT group. There is also a non-significant trend toward less ventilator-days per patient in the MCT/LCT group. More strong evidence is required before giving a recommendation on lipid emulsions in critically ill patients requiring PN.

**References**

1. Grau-Carmona T, Bonet-Saris A, García-de-Lorenzo A, et al. Influence of n-3 polyunsaturated fatty acids enriched lipid emulsions on nosocomial infections and clinical outcomes in critically ill patients: ICU lipids study. Crit Care Med 2015;43:31–9

**Table 84 (abstract A772).** metabolic variables

	MCT/LCT	Fish oil	p
Average daily caloric intake (Kcal/day) SD: Standard Deviation	1445 (SD 306)	1479 (SD 281)	0,8
Average daily lipid intake (g/day)	54 (SD 11)	47 (SD 14)	0,08
Days on PN	13 (SD 10)	12 (SD 8)	0,57
Bilirubin on 7th day of PN (mg/dl)	0,55 (SD 0,07)	1,26 (SD 0,8)	0,04
Daily intravenous insulin requirements (median, IQR) IQR: interquartile range	12 (0–26)	18 (8–38)	0,76

**Table 85 (abstract A772).** Clinical outcome

	MCT/LCT	Fish oil	p
Days on mechanical ventilation (median)	7 (0,75−17)	15 (9–31)	0,05
ICU length of stay (days) (median, IQR)	11 (4–20)	19 (15–34)	0,03
Hospital length of stay (days) (median, IQR)	44 (23–88)	44 (30–68)	0,06
New infections in ICU (n,%)	4 (40%)	9 (64%)	0,76
Hospital mortality (n,%)	4 (40%)	4 (28%)	0,28

#### A773 Comparison of two methods for non-invasive regional blood flow measurements in ICU patients

##### J. GRIP^1^, R. Kölegård^2^, P. Sundblad^2^, O. Rooyackers^1^

###### ^1^Karolinska University Hospital / Karolinska Institutet, Department of Anesthesiology and Intensive Care, Huddinge, Sweden; ^2^School of Technology and Health, Royal Institute of Technology, Department of Environmental Physiology, Swedish Aerospace Physiology Center, Stockholm, Sweden

####### **Correspondence:** J. GRIP - Karolinska University Hospital / Karolinska Institutet, Department of Anesthesiology and Intensive Care, Huddinge, Sweden

**Introduction:** In metabolic ICU research it is often desirable to measure local blood flow to quantify production/utilization of various substrates. The gold standard is to do this through dilution techniques of e.g. cold water or dye. These methods are however invasive and may cause pain or risk of injury of the patient. It is therefore desirable to use noninvasive techniques, such as Strain Gauge Venous Occlusion Plethysmography (SGVOP) and Doppler Ultra Sound (DUS). Both of these are used today but they measure blood flow through different principles and gives results in different units. These methods have previously been compared in healthy subjects but never in ICU Patients.

**Objectives:** We wanted to investigate the correlation between SGVOP and DUS and how they are able to detect changes in blood flow in ICU patients.

**Methods:** ICU patients (n = 10) with an expected stay of >24 hours were recruited after informed consent. All patients underwent simultaneous blood flow measurements of both legs at two separate time points (5–26 hours) and medical data was collected from charts. SGVOP was performed on the left side with the cuff placed on the medial thigh and a strain gauge placed on the thickest part of the calf, which was hanging free as the foot was supported by pillows. Blood flow was estimated as a mean of five measurements. DUS was performed on the right leg by measuring the diameter of the superficial femoral artery (SFA) thrice and assess blood flow was at angle >60^0^ as a mean of five heart strokes. A mean of three such measurements was calculated.

**Results:** The mean values at the two time points were 36.3 ± 27.4 and 28.6 ± 21.0 ml x min^−1^ x 100 ml tissue^−1^ (with 10 % variability) for SGVOP and for DUS 189 ± 126 and 128 ± 60 ml/min (7.7 % variability). The *r* value for comparison between the methods in all measurements was 0.684 (p < 0.001) and for the change in blood flow between the time points 0.423 (p = 0.223). However 3 patients had higher doses of noradrenaline (>0.075 μg/kg/min) and changes in noradrenaline between the time points. When these were excluded from the analysis, the *r* value for the remaining 7 patients were 0.710 (p = 0.004) for all measurements and 0.913 (p = 0.004) to assess the change between time points.

**Conclusions:** SGVOP measures blood flow in the lower leg and does not correlate exactly with DUS of SFA. The methods however correlate fairly good and approximates changes in blood flow very good in stable hemodynamic conditions, similarly as in previous studies in healthy subjects, but fails to do so in situations with higher doses and changes in noradrenaline. This might be because noradrenaline alters the portion of blood going to skin and muscle respectively.

#### A774 Lung damage in systemic sclerodermam

##### Ben Naser, F. Jaziri, A. Ben Jazia, M.Barghouth O .Hentati, W. Skouri, M. El Euch,M. Mahfoudhi, S. Turki, K. Ben Abdelghni, Ben Abdallah, B.N.M. Maha

###### ^1^Charles Nicolles Hospital, Systemic Disease, Tunis, Tunisia

####### **Correspondence:** B.N.M. Maha - Charles Nicolles Hospital, Systemic Disease, Tunis, Tunisia

**Introduction:** The Diffuse infiltrative lung disease (PID) is a relatively common complication of Scleroderma (ScS) and its evolution is highly variable and difficult to predict.

**Patients and methods:** It is a retrospective study including 54 files of patients with a systemic Scleroderma and followed up by our service, for a period of 29 years from January 1985 until December 2013. The diagnosis of CBS has been selected according to the criteria of the ACR as defined in 1980.

**Results:** Lung damage was objectified in 35 patients (64, 81 %), 28 women and 7 men, middle-aged 39.96 years ± 14.96. The average timeappearance of lung damage was 34, 20 ± 27, 31 months. Lung damage was revealed by a Dyspnea on exertion in 20 cases (37.03 %) associated with a dry cough in 18 (33.33 %). Lung damage was asymptomatic in 10 cases (14.41 %).Chest x-ray performed in all patients was normal in 25 cases (46.30 %). She showed reticular opacities in 9 patients (16.67 %), reticulonodularin 15 cases (27.77 %) and honeycomb images in 5 cases (9.26 %). The EFR practiced in 52 patients revealed a restrictive syndrome in 28 cases. Thoracic CT confirmed the diagnosis of diffuse interstitial Pneumonitis. It showed: a glass aspect frosted (n = 8), reticular intra-lobulaires images (n = 12), images in honeycomb (n = 9) and pulmonary micronodules (n = 6).Fifteen patients had benefited from monthly cures of cyclophosphamide. The evolution was marked by the stabilization of lesions in 15 cas(42,85 %) improvement in 10 cases (28.57 %) and worsening of lung damage in 10 (18.8 %) patients.

**Conclusion:** The lung damage is currently the leading cause of mortality in Systemic Scleroderma.

#### A775 Severity indexes as predictors of hospital and annual mortality in cirrhotic patients

##### J. Cánovas, F. Sotos, A. López, M. Lorente, A. Burruezo, D. Torres

###### Morales Meseguer Hospital, Murcia, Spain

####### **Correspondence:** J. Cánovas - Morales Meseguer Hospital, Murcia, Spain

**Introduction:** Mortality of cirrhotic patients with extrahepatic complications is high due to associated comorbidities. For these reasons it is important to individualize each case in severity and severity of the cause of ICU admission.

**Objectives:** To analyze the ability of differents severity indexes (both general and specific) in predicting hospital and annual mortality of liver cirrhosis patients admitted to an Intensive Care Unit.

**Methods:** Retrospective observational study on a prospective database of 10 years duration. All patients were admitted consecutively with primary or secondary diagnosis of cirrhosis. The mortality prediction is performed by constructing ROC curves calculating the area under the curve (AUC) with confidence intervals at 95 % (95 % CI).

**Results:** 229 patients were analyzed (mean age 58.6 ± 13.1 years, and 78.6 % males) . The APACHE II index admission was 25 ± 10 SAPS II 49 ± 21 and 21 ± MELD index 9. The SOFA rate and maximum income was 7 ± 4 and 11 ± 4, respectively. Hospital mortality and the year was 47.6 % and 60.7 % respectively. ABC and 95 % CI for hospital mortality and per year is presented in Table 86. ABC.

**Conclusions:** Cirrhotic patients admitted to ICU have a high mortality. The severity indexes both general and the specific MELD cirrhosis well predict hospital mortality. However, that best mortality predictor is the maximum SOFA index, with high capacity to predict mortality per year.

**Table 86 (abstract A775).** Desciption of SOFA abstractions

SOFA abstractions used as endpoint	Description
Early-SOFA	Between-group difference in mean SOFA score on study days 2, 3, 4 or 5
Late-SOFA	Between-group difference in mean SOFA score on study days 7, 10 or 14
Delta-SOFA	Delta SOFA as defined by RCT authors: Between-group difference in SOFA maximum during ICU stay minus admission SOFA, or; Between-group difference in SOFA on a fixed day minus admission SOFA, with the last observation carried forward in case of discharge or death

#### A776 Clinical characteristic and prognosis of patients with primary systemic vasculitides (PSV) and other autoimmune diseases treated in ICU

##### K. Polok^1^, A. Włudarczyk^2^, J. Górka^2^, A. Hałek^2^, J. Musiał^2^, W. Szczeklik^2^

###### ^1^Jagiellonian University Medical College, Kraków, Poland; ^2^Jagiellonian University Medical College, IInd Department of Internal Medicine, Kraków, Poland

####### **Correspondence:** W. Szczeklik - Jagiellonian University Medical College, IInd Department of Internal Medicine, Kraków, Poland

**Introduction:** Autoimmune diseases are significant challenge for Intensive Care physicians. They are often complicated by multiple-organ dysfunction and side effects of long-term immunosuppressive therapy. Moreover, treatment of infections is particularly difficult concerning constant immunosuppression. Primary systemic vasculitides (PSV) are subgroup of autoimmune diseases associated with especially problematic clinical course often complicated by diffuse alveolar hemorrhage leading to respiratory failure.

**Objectives:** The aim of the study was to compare patients suffering from systemic autoimmune diseases with those suffering from primary systemic vasculitides in terms of clinical condition severity on admission, treatment requirements and in-hospital mortality in ICU setting.

**Methods:** Medical records of 74 patients with systemic autoimmune diseases (SAD) including primary systemic vasculitides admitted to Intensive Care Unit of the Jagiellonian University Medical College, Krakow, Poland between 2001 and 2014 were analyzed in terms of demographic, clinical and laboratory data. Severity of patients' clinical condition was assessed using SAPS II, SAPS III, APACHE II, APACHE III and SOFA scales.

**Fig. 114 (abstract A776).**
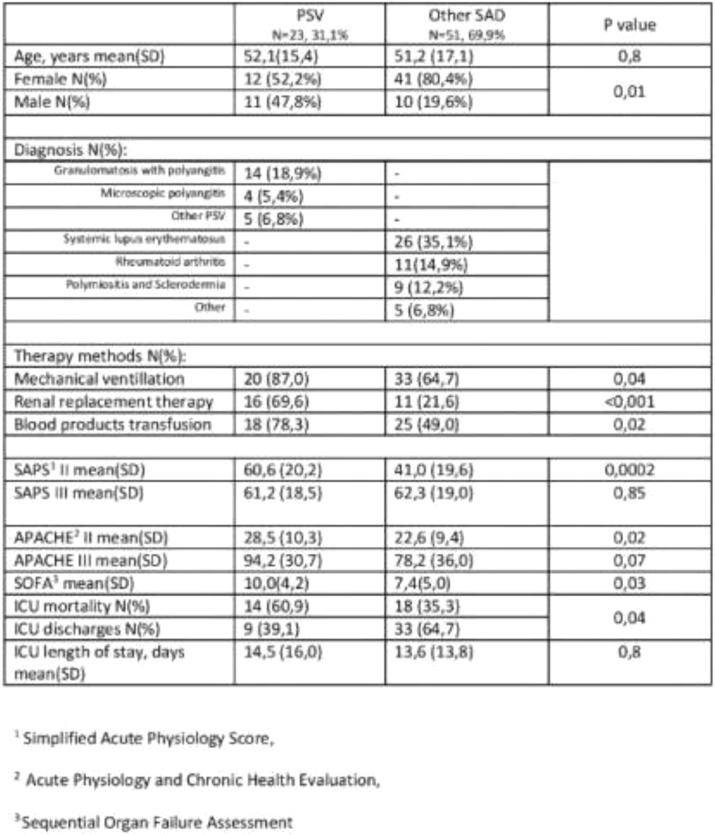
Demographic and clinical characteristic

**Results:** Demographic and clinical data are presented in Fig. 114. 23 patients with PSV and 51 patients with SAD were enrolled in the study. Patients with PSV had significantly higher scores in SAPS II, APACHE II and SOFA scales and required mechanical ventilation (87,0 % vs 64,7 %, p = 0,04), renal replacement therapy (69,6 % vs 21,6 %, p < 0,001) and blood products transfusions (78,3 % vs 49,0 %, p = 0,02) more often than patients with SAD. Also mortality was significantly higher among PSV group (60,9 % vs 35,3 %, p = 0,04).

**Conclusions:** Patients with PSV treated in the ICU are in more severe condition, require more complex intensive therapy and have poorer prognosis than patients with other SAD.

#### A777 Characteristics of complicated pulmonary embolism-deep venous thrombosis systemic diseases service

##### A. Ben Jazia, F. Jaziri, M. Bargouth, M. Bennasr, S. Turki, K. Ben Abdelghani, T. Ben Abdallah

###### Charles Nicolles Hospital, Systemic Disease, Tunis, Tunisia

####### **Correspondence:** A. Ben Jazia - Charles Nicolles Hospital, Systemic Disease, Tunis, Tunisia

**Introduction:** Deep vein thrombosis (DVT) is a common clinical situation causing morbidity and an important mortality . Because it can be a complicated pulmonary embolism can be life-threatening.

**Objectives:** Our goal is to study clinical features, Para clinical and scalable DVT complicated pulmonary embolism (DVT/PE).

**Materials. And methods:** We conducted a retrospective and descriptive study of the records of patients hospitalized for DVT/PE in the service of internal medicine, CHU Charles NICOLLES over a period of 30 years (1985–2015). We compared the patients with a DVT/PE (Group 1 G1) versus those who did not have pulmonary embolism complicating PST (Group 2 G2).

**Results:** We collected 120 cases of DVT. The mean age of patients was 41.76 ans [15–78]. The DVT/PE (Group1 G1) were observed in 28 patients either in 23.33 % of cases.. It was 14 women and 14 men. DVT non complicated with pulmonary embolism (Group 2 G2) were observed among 92 patients (76.66 %) . It was 36 women and 56 men. Elderly patients were significantly fewer in the group G1 (3 patients) versus 10 older subjects in the Group G2 (p = 0.01). Regarding thrombotic risk factors, there was no significant difference between the 2 groups. The frequency of recurrence weren't similar between the 2 groups: 3 in the group G1 and 26 in the Group G2. There were also significant etiological difference between the 2 groups. Indeed, neoplasia was observed in 2 patients group G1 and 9 patients in Group G2 (p = 0.02). Thrombophilia, Becket's disease,Lupus and hyperhomocystéinemia are kept respectively in 2,2,2 and 0 for patients of Group G 1 versus 6,24,3 and 4 patients of the Group G2. The cause of the DVT was idiopathic 17(60) patients of the group G1 and51(55) patients of the Group G2 (p =0.6).

**Conclusions:** Pulmonary embolism is a common and serious complication that can jeopardize the vital prognosis of the patient where the interests of well know the risk factors predisposing to this complication.

### Outcome analysis II

#### A778 The sequential organ failure assessment score (SOFA) as a surrogate endpoint for mortality in randomized controlled trials, a systematic review

##### H.-J. de Grooth^1^, I.L. Geenen^1^, J.-J. Parienti^2^, H.M. Oudemans-van Straaten^1^

###### ^1^VU University Medical Center, Department of Intensive Care, Amsterdam, Netherlands; ^2^Centre Hospitalier Universitaire de Caen, Unité de Biostatistique et de Recherche Clinique, Caen, France

####### **Correspondence:** H.-J. de Grooth - VU University Medical Center, Department of Intensive Care, Amsterdam, Netherlands

**Introduction:** The Sequential Organ Failure Assessment score (SOFA) is increasingly used as a surrogate endpoint in intensive care Randomized Controlled Trials (RCTs). Serially measured SOFA is independently associated with mortality in observational cohorts [1], but the association between RCT treatment effects on SOFA vs. mortality has not yet been quantified. It is currently unclear which SOFA abstraction is the most *responsive* and *consistent* surrogate endpoint for mortality in RCTs.

**Objectives:** Using study-level data from published RCTs that report both SOFA and mortality, our objective was to quantify the relation between SOFA endpoints and mortality and to identify which SOFA abstraction best reflects between-group mortality differences.

**Methods:** RCTs in adult intensive care patients reporting both SOFA and mortality endpoints were identified using a PubMed and Embase query. Reported SOFA abstractions were classified as *early, late* and *delta* according to the definition used to compute the SOFA endpoint (Table 87). Treatment effects on SOFA were calculated as the between-group SOFA standardized difference. Treatment effects on mortality were calculated as log odds ratio (OR) of treatment vs. control group mortality. We used random-effects meta-regression to: (i) quantify the linear relationship between RCT treatment effects on mortality (logOR) and SOFA; (ii) quantify residual heterogeneity (expressed as I^2^ and *tau*). The review protocol was prospectively registered (CRD42016034014).

**Fig. 115 (abstract A778).**
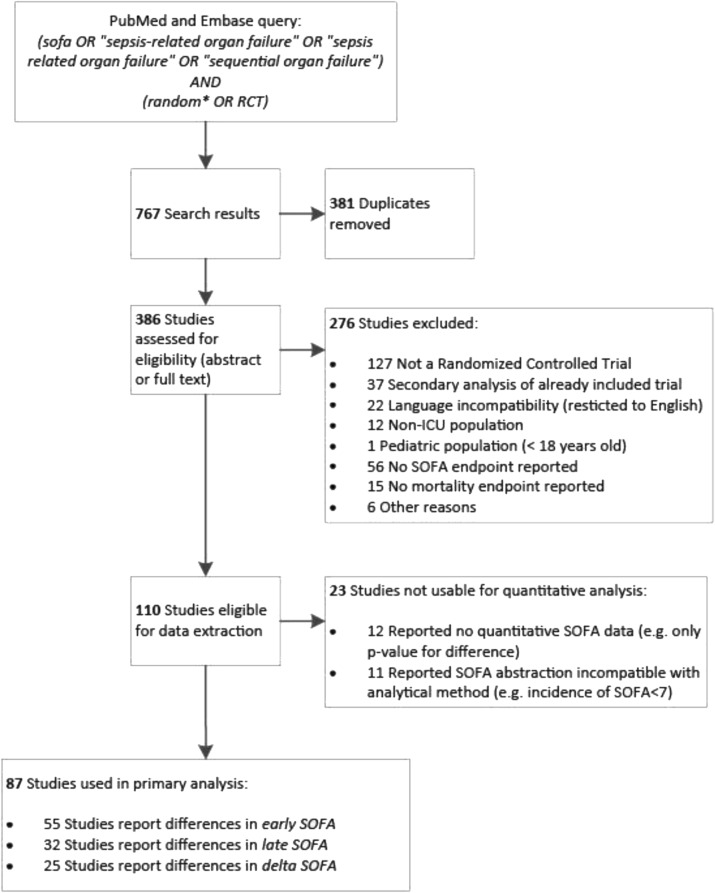
Flowdiagram of the systematic search

**Results:** Data from 87 studies were included in the analysis (Fig. 115). Fifty-five studies used Early-SOFA, 32 studies used Late-SOFA and 25 studies used Delta-SOFA as an endpoint. Studies reporting different SOFA abstractions had comparable characteristics regarding sample size, year of publication and study population.

**Fig. 116 (abstract A778).**
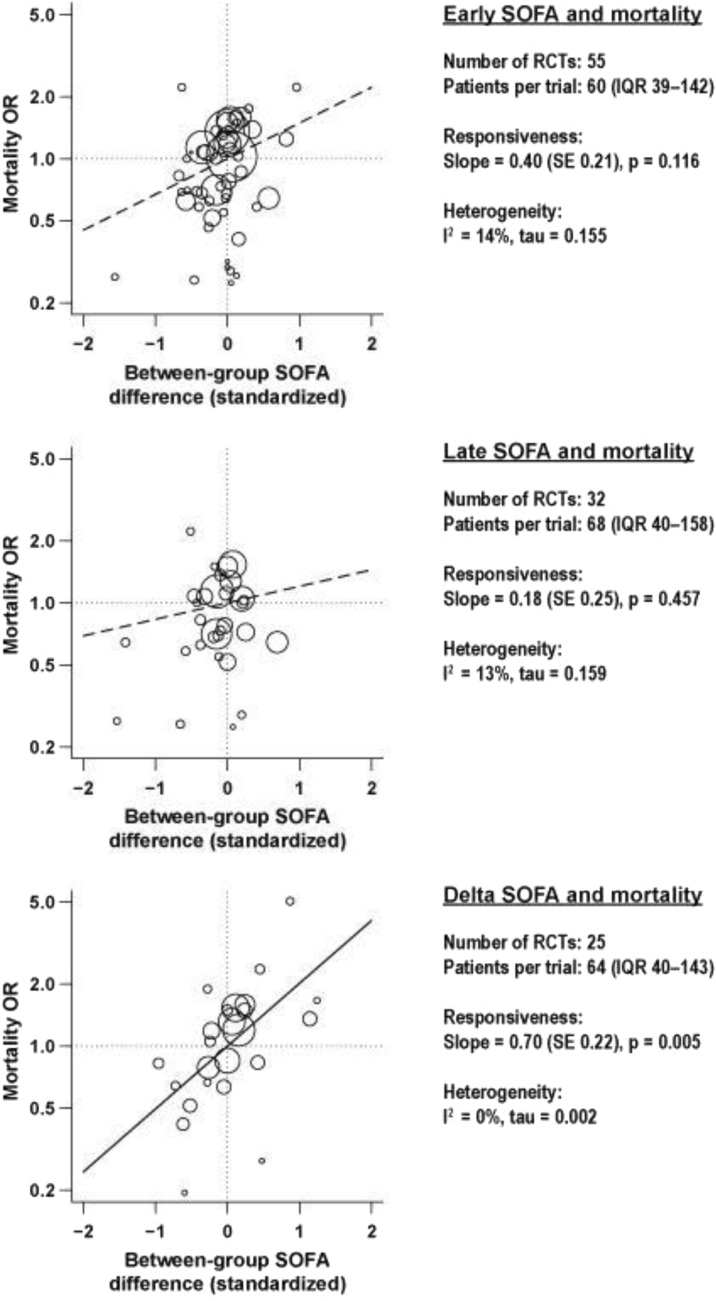
Meta-regression analyses

Between-group differences in Early-SOFA and Late-SOFA were not significantly associated with between-group differences in mortality (slope = 0.40 (SE 0.21), p = 0.116 and Slope = 0.18 (SE 0.25), p = 0.457, respectively), but difference in Delta-SOFA was (slope = 0.70 (SE 0.22), p = 0.005) (Fig. 116). The heterogeneity of the association was lowest for Delta-SOFA (I^2^ = 0 % vs. 14 % and 13 %, p < 0.01 against both using F-test on tau^2^).

**Conclusions:** Time-fixed Early- and Late-SOFA were the most frequently reported outcome measures among the reviewed RCTs. However, based on study level data aggregated in this systematic review, Delta-SOFA appears to be the most responsive and consistent surrogate endpoint for mortality.

**References**

[1] Ferreira et al. Serial evaluation of the SOFA score to predict outcome in critically ill patients. JAMA 286, 1754–1758 (2001).

**Table 87 (abstract A778).** Values of the different analyzed index

Hospital Mortality	Year mortality					
ABC CI-95 %	ABC CI-95 %					
APACHE II 0	836 0	782-0	882 0	769 0	709-0	822
SAPS II 0	853 0	801-0	896 0	796 0	738-0	846
MELD 0	871 0	820-0	911 0	799 0	741-0	841
initial SOFA 0	827 0	772-0	874 0	744 0	715-0	827
delta SOFA 0	727 0	664-0	774 0	723 0	660-0	779
maximum SOFA 0	912 0	868-0	945 0	856 0	803-0	898

#### A779 Clinical characteristics and outcome of those > =90 years receiving intensive care in a regional hospital: a retrospective observational study

##### H.P. Shum^1^, H.S. King^1^, K.C. Chan^2^, W.W. Yan^1^

###### ^1^Pamela Youde Nethersole Eastern Hospital, Hong Kong, China; ^2^Tuen Mun Hospital, Hong Kong, China

####### **Correspondence:** H.P. Shum - Pamela Youde Nethersole Eastern Hospital, Hong Kong, China

**Introduction:** As the prognosis of very elderly patients is generally limited, admissions to intensive care among these patients are often restricted. Therefore, only very few information is available on their prognosis.

**Objectives:** To evaluate the clinical characteristics and outcome of critically ill patients > =90 years old and compared with those between 70 and 79 years old.

**Methods:** Retrospective analysis of administrative data of patients admitted between 1/1/2009 and 31/12/2013 to an ICU of a regional hospital.

**Results:** Over 5 years, 109 patients aged ≥90 years old were admitted (1.4 % total ICU admission). Their median age was 92 and predominantly female (62.4 %). The majority of patients (96.3 %) were emergency admission with 36.7 % for postoperative care. Compared with those aged 70–79, those aged > =90 years old had similar prevalence of comorbidities (except metastatic carcinoma), comparable chance to receive mechanical ventilation but less likely to have renal replacement therapy (RRT) (16.2 % vs. 4.6 %). Despite having similar disease severity as assessed by Acute Physiology and Chronic Health Evaluation (APACHE) IV minus age score, they have higher ICU, hospital, 90-day, 180-day and 2-year mortality. After adjustment of disease severity, co-morbidities and the use of RRT, their 2-year mortality differed by 1.9 times. Around 60 % of patients aged > =90 years old could be discharged home but only 41.3 % survived 2 years after ICU admission.

**Conclusions:** Despite the fact that two-third of them could be discharged home following treatment in ICU, only around 40 % survived 2 years from ICU admission. These findings provided useful information for ICU triage purpose.

#### A780 Admission of critical octogenarian patients to intensive care (ICU). Short and long term results

##### J. Gonzalez Londoño, C. Lorencio Cardenas, M. Morales Pedrosa, C. Murcia Gubianas, C. Fuster Bertolin, N. Vila Batllori, J.M. Sirvent

###### ^1^Dr Josep Trueta University Hospital, Intensive Care Unit, Girona, Spain

####### **Correspondence:** J. Gonzalez Londoño - Dr Josep Trueta University Hospital, Intensive Care Unit, Girona, Spain

**Objectives:** The ICU admission of patients over 80 years of age has been questioned because of the high mortality and low life expectancy of these patients. However, with the growing increase of the life expectancy of the population together with the improvement of diagnostic and therapeutic techniques, more and more, age is not a limiting factor for ICU admission. Our goal is to analyze the short and long term the morbidity and mortality of octogenarian patients admitted to the ICU, as well as functional status after ICU discharge.

**Materials and methods:** Retrospective observational study in which all patients over 80 years old admitted to a polyvalent ICU of a tertiary hospital during the years 2012–2014 were included. Demographic variables, severity indices (APACHE II), initial lactate, mortality and length of stay were registered. Patients were followed during one year after ICU discharge. 6 months and one year mortality as well as the value of the Karnofsky scale after 1 year of discharge were registered.

**Results:** During the study period, 178 octogenarian patients were included [2012 = 52 (29.2 %); 2013 = 62 (34.8 %) and 2014 = 64 (36 %)]. 56.7 % were male with a median age of 82.7 years of age (±2.4) and an APACHE II of 21.8 (±7.1). The initial lactate was 25.1 mg/dL (±22). Reasons for admission were non-respiratory sepsis (38.8 %), post-operative cardiac surgery (16.9 %), cardiac arrest (5.6 %), polytrauma (4.5 %), pneumonia (4.5 %), and cerebral hemorrhage (3.9 %). The average intake was 6.7dies (±7.7) in the ICU and 17.7dies (±15) in the hospital. ICU mortality was 30.3 % and hospital mortality was 41 %. Mortality at 6 months of discharge from ICU was 48.3 % and at one year of discharge 51.1 %. The Karnofsky scale after one year of ICU discharge was 73.2 (±16.2).

The mortality for octogenarian patients was 10 % higher than the global mortality of ICU admitted patients during the same period (30.3 % vs. 21 %). Both the initial APACHE II and lactate were significantly higher in patients eventually died (20.4 ± 6.3 vs 25.3 ± 7.8; p < 7.20 vs 12:05 ± 30.8 and 22.7 ± 24, p < 0.05). ICU mortality was significantly higher in patients with pneumonia (75 %). However, mortality was significantly lower in postoperative cardiac surgery (6.7 %; p < 0.05).

**Conclusions:** The number of octogenarian patients admitted to ICUs has been increasing in recent years. Mortality in patients over 80 years of age admitted to our ICU is higher than the overall mortality of our ICU. However, it varies significantly depending on the diagnosis. The initial lactate and APACHE II are also prognostic in this group of patients. The mortality of octogenarians patients admitted to our ICU reaches the 50 % after one year of discharge, and those who survive cannot perform a normal daily activity.

#### A781 Outcomes of patients admitted to intensive care unit with acute variceal haemorrhage

##### K. Wykes, J. Jack, P. Morgan

###### East Surrey Hospital, Intensive Care Unit, London, UK

####### **Correspondence:** K. Wykes - East Surrey Hospital, Intensive Care Unit, London, UK

**Background:** Acute variceal haemorrhage (AVH) presenting to Intensive Care Unit (ICU) is associated with significant mortality.[1]

**Objectives:** To compare outcomes and factors associated with hospital mortality (HM) in patients admitted with AVH to a busy district general hospital ICU.

**Methods:** A retrospective study of adult patients (N = 82) admitted to ICU with a primary admission diagnosis of AVH from 1993–2016 was performed by searching the electronic medical records database. We applied statistical analysis (Pearson chi-square) to assess the differences in outcomes for patients receiving different interventions.

**Results:** The median age of our patient population was 56.3 years, 62 % were male, and there were 8 transfers to tertiary centres.

The percentage HM for patients admitted to ICU with a variceal bleed was found to be 43 %, with mortality rising to 59 % if that patient was intubated and ventilated, and 92 % if that patient received inotropic support during their admission. Inotropic support (OR, 11.9; 95 % CI, 1.5 to 97.3; chi-square 7.9 P = 0.0048) and an APACHEII score above 40 (OR, 3.98; 95 % CI, 1.0-15.5; chi-square 4.4 P = 0.0459) were both significant independent predictors for mortality. Intubation and ventilation alone was not a significant mortality predictor.

**Conclusions:** A patient presenting to ICU with an AVH is predicted to have a significantly worse outcome if they are commenced on inotropic support, with mortality rising to 92 % from 43 %. Intubation and ventilation alone was not a significant predictor of a worse outcome. This result should prompt a consideration of outcomes when starting inotropic support in a patient on ICU who presents with a variceal bleed.

**References**

[1] Al-Freah MA1, Gera A, Martini S, McPhail MJ, Devlin J, Harrison PM,Shawcross D, Abeles RD, Taylor NJ, Auzinger G, Bernal W, Heneghan MA, Wendon JA **Comparison of scoring systems and outcome of patients admitted to a liver intensive care unit of a tertiary referral centre with severe variceal bleeding.** Aliment Pharmacol Ther. 2014 Jun;39(11):1286–300. doi: 10.1111/apt.12744. Epub 2014 Apr 16.

[2] García-Pagán JC1, Reverter E, Abraldes JG, Bosch J. **Acute variceal bleeding**. Semin Respir Crit Care Med. 2012 Feb;33(1):46–54. doi: 10.1055/s-0032-1301734. Epub 2012 Mar 23.

[3] Turon F1, Casu S, Hernández-Gea V, Garcia-Pagán JC. **Variceal and other portal hypertension related bleeding.** Best Pract Res Clin Gastroenterol. 2013 Oct;27(5):649–64. doi: 10.1016/j.bpg.2013.08.004. Epub 2013 Sep 5.

**Grant acknowledgement**

nil

#### A782 Loss of muscle in critically ill patients admitted to medical intensive care unit

##### A. Mukhopadhyay^1^, H.Y. Chan^1^, Y. Kowitlawakul^1^, D. Remani^1^, C.S.-F. Leong^2^, C.J. Henry^2^, Z.A. Puthucheary^1^

###### ^1^National University Health System, Singapore, Singapore; ^2^Clinical Nutrition Research Centre,Singapore Institute for Clinical Sciences, Singapore, Singapore

####### **Correspondence:** A. Mukhopadhyay - National University Health System, Singapore, Singapore

**Introduction:** Loss of muscle mass in critically ill patients admitted to Intensive Care Unit (ICU) is common^1^ and contributes to ICU acquired weakness. Loss of rectus femoris muscle has been shown to be related to length of stay in ICU^2^; however previous studies have not investigated the relationship between mortality and muscle loss.

**Objectives:** To seek an association between muscle loss and 28-day mortality in medical ICU (MICU) patients and compare rates of muscle loss in patients dead or alive at 28-day.

**Methods:** This was an observational study in the MICU of a university affiliated academic medical centre. Serial measurements of rectus femoris thickness (RFT) by ultrasound were performed on admission, day 3,7 and 10.Other parameters including demographics, body mass index (BMI), Acute Physiology and Chronic Health Evaluation II (APACHE II) and Charlson co-morbidly index were collected. Analysis was performed with a generalised linear mixed model.

**Results:** We included 45 patients (65 % male, mean age 63 ± 11 years, mean BMI 25.2 ± 4.8 kg/m^2^, mean APACHE II score 28 ± 8),median (IQR) ICU and hospital stay of 6(3–10) and 14(5–55) days respectively with a 24.4 % 28-day mortality.Both patient group lost significant muscle thickness (mean loss of right RFT, day 3: −7.9 ± 7 %,day 10: −11.3 ± 9 %, p < 0.001) from 0–10 days but no difference was observed between the groups (p = 0.45).Loss of RFT was significantly associated with length of stay in the hospital (p = 0.048,R^2^ = 0.1).Each 1 % loss in the RFT was associated with 28 % increased relative risk for mortality (p < 0.001,OR 1.28,95 % CI 1.14-1.45).

**Conclusions:** Critically ill patients uniformly lose muscle during their hospital stay and muscle loss was significantly associated with 28-day mortality.

**References**

1. Mechanisms of Chronic Muscle Wasting and Dysfunction After an Intensive Care Unit Stay: A Pilot Study. Dos Santos C, Hussain SN, Mathur S et al. Am J Respir Crit Care Med. 2016 Apr 8. [Epub]

2. Acute skeletal muscle wasting in critical illness. Puthucheary ZA, Rawal J, McPhail M et al. JAMA. 2013 Oct 16;310(15):1591–600

**Grant acknowledgement**

This study was done with National University Health System Clinician grant 2013, Singapore

**Fig. 117 (abstract A782).**
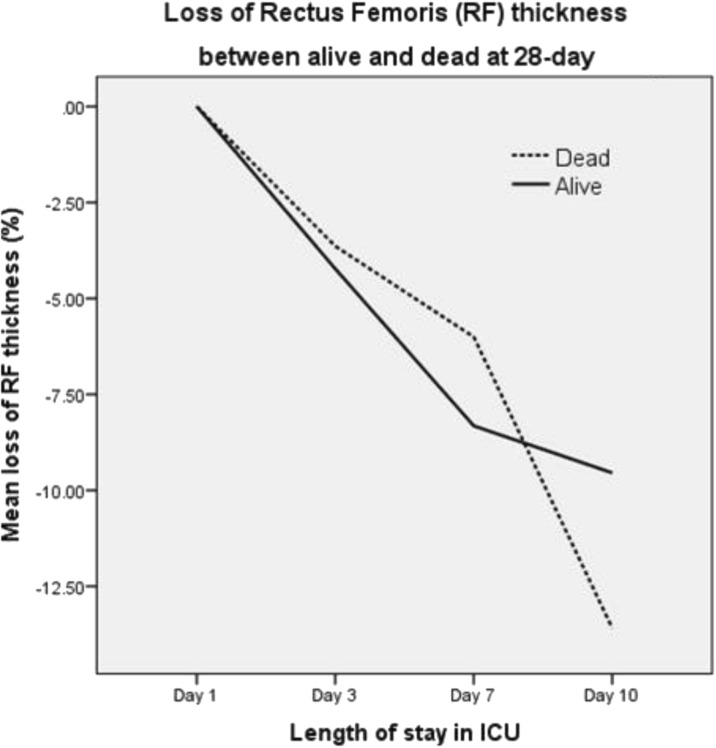
Loss of Rectus Femoris Dead vs Alive

#### A783 The epidemiology and outcome of critical illness in Mongolia

##### N. Mendsaikhan^1^, T. Begzjav^1^, G. Lundeg^2^, M. Dünser^3^

###### ^1^Intermed Hospital, Intensive Care Department, Ulaanbaatar, Mongolia; ^2^Health Sciences University of Mongolia, Division of Emergency Medicine and Anesthesia, Ulaanbaatar, Mongolia; ^3^Salzburg General Hospital and Paracelsus Private, Salzburg, Austria

####### **Correspondence:** N. Mendsaikhan - Intermed Hospital, Intensive Care Department, Ulaanbaatar, Mongolia

**Introduction:** Little is known about the epidemiology and outcome of critical illness in low- and middle-income countries (1). In these settings, intensive care medicine is challenged by delicate resource restrictions which are likely to affect patient outcome (2).

**Objective:** To evaluate the epidemiology and outcome of critical illness in Mongolia, a lower-middle income country in Central Asia.

**Methods:** In this prospective observational study, demographic, clinical and outcome data of all critically ill patients admitted to the intensive care units (ICU) of five tertiary university teaching hospitals and five secondary hospitals in the capital city of Ulaanbaatar as well as nine secondary province hospitals were collected during a six month period in 2014/15. The study protocol was approved by the Ethics Committee of the Mongolian Medical University.

**Results:** During the observation period, 2,032 patients (53.6 % male) with a median age of 49 years (interquartile range, 36–62 years) were admitted to the study ICUs. The ten most frequent ICU admission diagnoses were stroke (ischemic and hemorrhagic) (n = 354; 17.4 %), decompensated liver cirrhosis (n = 187; 9.2 %), acute or acute-on-chronic heart failure (n = 182; 9 %), infection (n = 169; 8.3 %), trauma (n = 153; 7.5 %), traumatic brain injury (n = 145; 7.1 %), acute abdomen (n = 143; 7 %), pre-eclampsia/eclampsia (n = 117; 5.8 %), acute or acute-on-chronic renal failure (n = 80; 3.9 %) and postoperative care following elective and emergency surgery (n = 66; 3.2 %). 100 %, 31.7 % and 13 % of patients had access to mechanical ventilation, renal replacement therapy and invasive hemodynamic monitoring, respectively. ICU mortality was 23.5 % in the total population and 26.6 % when maternal cases were excluded. Multiple organ dysfunction syndrome (38.1 %), coma (36.6 %) and shock (15.6 %) were the three most common causes of death. The five ICU admission diagnoses with the highest case fatality rate were lung tuberculosis (51.9 %), traumatic brain injury (42.1 %), decompensated liver cirrhosis (33.7 %), stroke (31.9 %) and infection (30.8 %). The five ICU admission diagnoses causing most death cases were stroke (n = 113), decompensated liver cirrhosis (n = 63), traumatic brain injury (n = 61), infection (n = 52) and acute abdomen (n = 38).

**Conclusions:** Critical illness in Mongolia affects younger patients than reported from high-income countries. ICU admission diagnoses are similar with a particularly high incidence of stroke and decompensated liver cirrhosis. Total ICU mortality is approximately 25 % with most ICU deaths caused by stroke, decompensated liver cirrhosis and traumatic brain injury.

**References**

(1) Dondorp AM, Iyer SS, Schultz MJ. Critical care in resource-restricted settings. JAMA 2016; 315:753–754.

(2) Baker T. Critical care in low-income countries. Trop Med Int Health 2009; 14:143–148.

#### A784 Association of hypochloremia with mortality in critically ill patients

##### E.D. Valenzuela Espinoza, S.P. Welsh, M.F. Motta, E. Guerra, M.C.l. Zerpa, F. Zechner, M. Furche, F. Berdaguer, P.N. Rubatto Birri, A. Risso-Vazquez, A. Dubin, F.D. Masevicius

###### Sanatorio Otamendi y Miroli, Buenos Aires, Argentina

####### **Correspondence:** E.D. Valenzuela Espinoza - Sanatorio Otamendi y Miroli, Buenos Aires, Argentina

**Introduction:** Chloride alterations play a major physiologic role in the development of acid–base disorders. Most of the studies related to abnormalities in chloride concentration have been focused on the hyperchloremic metabolic acidosis. Although hypochloremic metabolic alkalosis has been suggested as a predictor of mortality, this issue remains insufficiently studied.

**Objective:** To assess the prognostic value of hypochloremia in a large cohort of critically ill patients.

**Methods:** We conducted a single-center retrospective observational study that included all patients admitted at the ICU between 2007 and 2015. Chloride serum concentration adjusted to blood sodium level, acid–base status, routine laboratory, and clinical variables were measured at admission. The primary outcome was defined as the all-cause hospital death according to quintiles of serum chloride levels. We performed a multivariate logistic regression, in which quintiles of chloride levels were analyzed as dummy variables.

**Fig. 118 (abstract A784).**
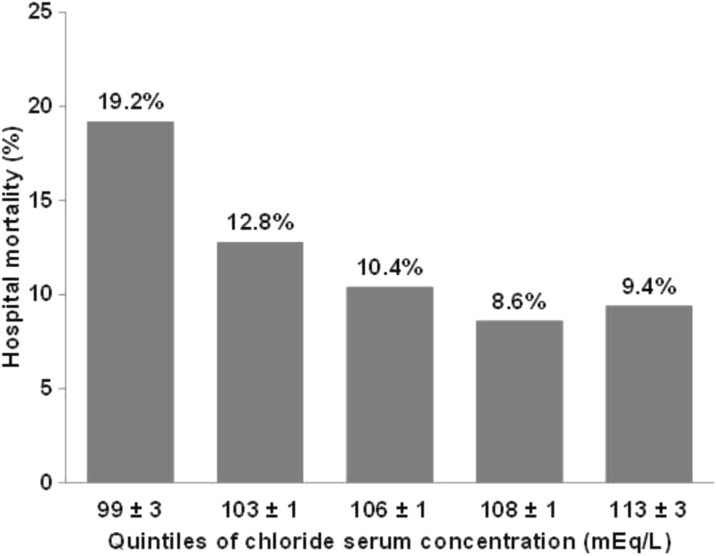
ᅟ

**Results:** We included 4898 patients. APACHE II score was 13 ± 7. Hospital mortality was 12 %. Fig. 118 shows the hospital mortality in the different quintiles of chloride concentration.

In the multivariate analysis, the quintile of chloride concentration of 99 ± 3 mEq/L was independently associated with a worse outcome (OR = 2.00, 95 % CI = 1.46-2.76, *P* = 0.001) after adjusting to APACHE II score, vasopressor requirements, mechanical ventilation, and lactate levels.

**Conclusions:** In this large series of critically ill patients, the presence of hypochloremia at ICU admission was an independent predictor of mortality. In contrast, hyperchloremia was not associated with increased mortality.

#### A785 Long-term outcomes post CRRT in an Irish ICU setting

##### D. Greaney^1^, A. Magee^1^, G. Fitzpatrick^2^

###### ^1^AMNCH, Critical Care, Dublin, Ireland; ^2^AMNCH, Dublin, Ireland

####### **Correspondence:** D. Greaney - AMNCH, Critical Care, Dublin, Ireland

**Introduction:** Continuous renal replacement therapy (CRRT) is commonly employed in ICU for patients with acute kidney injury and is associated with increased morbidity and mortality. However, renal outcomes at in the longer term are not widely described in the literature and have never been described in Ireland.

**Objectives:** We analysed all patients admitted to our tertiary ICU who were commenced on CRRT over a 2 year period. Our primary aim was to calculate the mortality of this cohort and estimate the likelihood of survivors requiring long-term dialysis. Our secondary aim was to analyse the characteristics of those requiring long-term dialysis.

**Method:** We conducted a retrospective cohort study of all patients requiring CRRT admitted to our ICU between 2013 and 2015. From the Clinical Information System, we analysed patient age, sex, diagnosis, illness severity scores, pre-CRRT creatinine levels, AKIN scores, length of CRRT and ICU admission. Survivors with new onset renal failure were followed up at 6 and 12 months to determine survival, dialysis dependency and serum creatinine level. This study received ethical committee waiver.

**Results:** Of 686 ICU admissions, 202 patients (29.4 %) received CRRT. 50 % of these patients died prior to hospital discharge. 85 patients had new onset renal failure and survived to hospital discharge. The 5 patients dialysed for toxin removal were excluded from further analysis. 11 patients (13.8 %) required ongoing CRRT at hospital discharge whereas 69 patients (86.2 %) did not. Of those requiring ongoing CRRT, 1 patient had died at 6 months, 2 further patients had died at 12 months, and 1 patient was lost to follow-up. Of the remaining 7 patients, 4 (57 %) were dialysis dependent at 12 months. Of the 69 patients who did not require RRT at hospital discharge, 12 had died at 6 months and 3 further patients had died at 12 month follow-up. 4 patients were lost to follow-up and a further 5 patients did not have blood results available. Of the remaining 45 patients, 100 % were not dialysis dependent at 12 months.

**Conclusions:** The mortality rate for patients admitted to ICU and commenced on CRRT in our cohort was consistent with that in the international literature. The likelihood of requiring long-term dialysis for survivors was similarly consistent. This novel information is of value in the management and prognostication of patients requiring CRRT in an Irish ICU setting.

**References**

1. Allegretti AS, Steele DJ, David-Kasdan JA, Bajwa E, Niles JL, Bhan I. Continuous renal replacement therapy outcomes in acute kidney injury and end-stage renal disease: a cohort study. *Critical Care*. 2013;17(3):R109

2. Rimes-Stigare C, Frumento P, Bottai M, Mårtensson J, Martling C-R, Bell M. Long-term mortality and risk factors for development of end-stage renal disease in critically ill patients with and without chronic kidney disease. *Critical Care*. 2015;19:383.

#### A786 Predictive value of serum estradiol for mortality in critically ill patients

##### R.G. Lugo-Cob, L.A. Sánchez-Hurtado, P.C. Arvizu-Tachiquín, B.C. Tejeda-Huezo, A.A. Cano-Oviedo, J.A. Baltazar-Torres

###### Hospital de Especialidades, Centro Médico La Raza, IMSS, Intensive Care Unit, Mexico, Mexico

####### **Correspondence:** P.C. Arvizu-Tachiquín - Hospital de Especialidades, Centro Médico La Raza, IMSS, Intensive Care Unit, Mexico, Mexico

**Introduction:** During acute illness, the risk of death depends mainly on the severity of the disease, the previous health status of the patient, and how he (she) responds to injury or disease. In the last decade, several studies have evaluated the impact of sex on mortality of patients with different acute problems and they have found that hormones may be a factor that also influences the outcome of critically ill patients.

**Objective:** To evaluate the capacity of serum estradiol levels to predict mortality in critically ill patients.

**Patients and methods:** A prospective study was performed in critically ill patients admitted to the intensive care unit (ICU). Serum estradiol level was measured at admission to the ICU, its discriminative capacity to predict mortality was determined by analysis of the receiver operative characteristics (ROC) curve, and its association with mortality by logistic regression analysis. A p value < 0.05 was considered statistically significant.

**Results:** We included 131 patients with a mean age of 48.9 years, 57.3 % men. The serum estradiol was higher in non-survivors compared with survivors: 116 vs 67.2 pg/mL, respectively (p < 0.0001). The area under the ROC curve of serum estradiol to predict mortality was 0.74 (p < 0.0001). The serum estradiol >97.9 pg/mL had sensitivity 60 %, specificity 90 %, positive predictive value 64 %, negative predictive value 88 %, positive likelihood 6, and negative likelihood 0.44 to identify mortality. In the multivariate analysis, it had OR of 6.47 (p = 0.002) for ICU mortality.

**Conclusions:** Serum estradiol is elevated in critically ill patients, especially in those who die, it has good discriminative ability for mortality and is an independent risk factor for death in this group of patients.

**Keywords:** Critically ill patients, serum estradiol, mortality.

**References**

1) Dossett L, Swenson B, Evans H. Serum estradiol concentration as a predictor of death in critically ill and injured adults. Surg Infect 2008;9(1):41–8.

2) Sakr Y, Elia C, Mascia L, Barberis B. The influence of gender on the epidemiology of and outcome from severe sepsis. Crit Care 2013;17(2):50–9.

3) Kauffmann R, Norris P, Jenkins J. Estradiol during critical illness are associated with mortality independent of admission estradiol. J Am Coll Surg 2011;212(4):703–12.

4) Romo H, Kajdacsy-Balla A, Vincent J. Effect of patient sex on intensive care unit survival. Arch Intern Med 2004;164:61–5.

5) Feng JY, Liu KT, Abraham E. Serum estradiol levels predict survival and acute kidney injury in patients with septic shock: a prospective study. PLoS ONE 9;(6) e97967. Doi:10.1371/journal.pone.0097967.

6) May A, Dossett L, Norris P. Estradiol is associated with mortality in critically ill trauma and surgical patients. Crit Care Med 2008;36(1):62–8.

7) Dosset L, Swenson B, Heffernan D, et al. High levels of estrogens are associated with death in the critically injured adult. J Trauma 2008;64:580–5.

#### A787 The effects of secondhand smoke on outcomes in ICU after scoliosis surgery

##### M.S. Aydogan, T. Togal

###### Inonu University, Intensive Care, Malatya, Turkey

####### **Correspondence:** T. Togal - Inonu University, Intensive Care, Malatya, Turkey

**Introduction:** Secondhand smoke (SHS) exposure is harmful and a major problem worldwide that is associated with ill health and mortality (1,2).

**Objectives:** The purpose of this study was to investigate the association between exposure to secondhand smoke (SHS) on outcomes in intensive care unit (ICU) after scoliosis surgery.

**Methods:** This prospective observational study was conducted in 103 patients, 12–18 years, with idiopathic scoliosis for elective posterior spinal fusion. All patients underwent standard total intravenous anesthesia and epidural morphine with PCA. Quality of pain relief and sedation were assessed using the Numeric Visual Analog Scale (NVAS), Richmond Agitation Sedation Scale (RASS). The patients were divided into three groups: Group S; consisted of smokers, Group SHS; consisted of exposure smokers, and Group NS; consisted of individuals who did not have any history of smoking and were not exposed to smoke. Information was reported in questionnaires and took blood samples for analysis of serum cotinine obtained at the time of study enrollment. Demographic and clinical characteristics included morphine usage, blood transfusion, duration of mechanical ventilator support and length of ICU stay were recorded.

**Results:** The patient characteristics did not differ between the groups. There was a statistically significant difference among groups in terms of serum cotinine levels. There were no significant differences in NVAS and RASS scores between the groups. The HR and MAP were not differ between the three groups of patients at all the evaluation time. Morphine consumption was significantly less in Group SHS compared with that of Group S at all time points (P < 0.05). There were no significant differences regarding the number of transfused blood units between the three groups of patients. Duration of MV support was significantly higher in group SHS than in group S (P < 0.05). The median length of ICU stay was significantly longer in group SHS than in group S (P < 0.05). The occurrence of pneumonia in this population was also similar between the three groups of patients. there were no significant differences between the groups with regard to postoperative side effects.

**Conclusions:** We found that SHS was associated with increased length of ICU stay compared to nonsmokers among scoliosis surgery patient. We concluded that it is important to avoid SHS before at ICU admission when possible.

**References**

1 -Homa DM, Neff LJ, King BA, Caraballo RS, Bunnell RE, Babb SD, et al. Vital signs: disparities in nonsmokers' exposure to secondhand smoke United States, 1999–2012. MMWR Morb Mortal Wkly Rep 2015;64:103–8.

2 -Mannino DM, Moorman JE, Kingsley B, Rose D, Repace J. Health effects related to environmental tobacco smoke exposure in children in the United States: data from the Third National Health and Nutrition Examination Survey. Arch Pediatr Adolesc Med 2001; 155:36–41.

#### A788 Patients with acute kidney injury from severe sepsis requiring continuous renal replacement therapy, are outcomes improving?

##### A. Taha^1^, H.Z. Chai^1^, C. Kam^2^, S.S. Yang Razali^2^, V. Sivasamy^2^, L.Y. Kuan^2^, V. Poulose^2^

###### ^1^Changi General Hospital, Respiratory and Critical Care Medicine, Singapore, Singapore; ^2^Changi General Hospital, Singapore, Singapore

####### **Correspondence:** A. Taha - Changi General Hospital, Respiratory and Critical Care Medicine, Singapore, Singapore

**Introduction:** Acute kidney injury (AKI) occurs in 20 - 50 % in patients with severe sepsis, with more than half of these patients needing continuous renal replacement therapy (CRRT)^1^. The mortality among the patients needing CRRT generally exceeds 60 %^2,3^.

**Objectives:** To evaluate the outcome of patients in the medical intensive care unit (MICU) with a diagnosis of severe sepsis with AKI and who required CRRT.

**Methods:** We conducted a retrospective analysis of patients admitted to Changi General Hospital MICU with a diagnosis of severe sepsis with AKI who required CRRT from June 2013 to January 2015.

**Results:** A total of 213 patients were screened and 33 were excluded because of incomplete data. One hundred and eighty patient records were analyzed. Of these, 105 (58.3 %) were male. The mean APACHE II score was 28.13 ± 9.64. The median length of MICU stay was 3 days (interquartile range 1–7) and median hospital length of stay was 16 days (interquartile range 9–33). The median duration of dialysis was 1 day (interquartile range 1–2). The MICU mortality was 38.9 % and hospital mortality was 39.4 %.

**Conclusions:** Mortality rates in our study were lower than what is previously stated in the literature. It may be worthwhile to conduct a multicentre study which includes other institutions in Singapore.

**References**

1. Rangel-Frausto MS, Pittet D, Costigan M, Hwang T, Davis CS, Wenzel RP. The natural history of the Systemic Inflammatory Response Syndrome (SIRS): A prospective study. JAMA. 1995;273:117–23.

2. Hussain S, Piering W, Mohyuddin T, Saleh M, Zhu YR, Hannan M, Cohen E. Outcome among patients with acute renal failure needing continuous renal replacement therapy: A single center study. Hemodial Int. 2009 Apr;13(2):205–14.

3. Walcher A, Faubel S, Keniston A, Dennen P. In critically ill patients requiring CRRT, AKI is associated with increased respiratory failure and death versus ESRD. Ren Fail. 2011;33(10):935–42

#### A789 Differences in critical care and hospital length of stay between two cohorts of patients admitted with traumatic brain injury (TBI) and hypoxic brain injury (HBI) between January 2011 and March 2016 to a large London teaching hospital

##### M.A. Lopez Morales

##### St Georges's Foundation Trust, Neuro Intensive Care Unit, London, UK

**Introduction:** Patients admitted to Adult Critical Care following Brain Injury who survive their hospital episode but do not regain 'normal' functionality (sufficient to return home) remain dependent upon healthcare for substantial periods of time. This poster compares the pathways experienced by two groups of patients with Brain Injuries (Traumatic vs Hypoxic) with the intention of influencing future planning strategies.

**Objectives**

- To identify the number of admissions and length of stay (LOS) in Critical Care/On the ward of patients with TBI/HBI;
- To identify the hospital discharge destination of survivors following TBI/HBI, where discharge home was not possible;
- Review of currents pathways for on-going management of TBI/HBI patients following cessation of acute care.

**Methods:** Retrospective analysis of data collected via Ward Watcher software (Critical Care Audit Ltd) of all TBI/HBI admissions to the Adult Critical Care Directorate of a large London teaching hospital between 01/01/2011 up to the 20/03/2016.

**Results:** Between 01/01/2011 and 20/03/2016, 205 patients were admitted to Adult Critical Care with TBI, versus 41 patients with HBI.

The average LOS in Adult Critical Care units for patients suffering TBI is 9.7 days versus 23.4 days following HBI. Following Critical Care discharge, hospital (ward) stay is 29.9 days for TBI patients and 48.9 days for HBI patients.

TBI patients Adult Critical Care Stay during this period was 1980 days versus 938 days for patients with HBI. Following discharge from this London teaching hospital, those patients unsuitable for discharge home received on-going care in other 'centres' .

Centre discharged to

- **TBI** 19 % Rehabilitation Centres, 78 % Other Hospitals, 20 % Nursing Home.
- **HBI** 19 % Rehabilitation Centres, 51 % Other Hospitals, 20%Other Hospitals,10 % Still awaiting discharge.

Analysis of care provision for these patients identified a clear structure and pathway for patients with TBI, supported by a multiprofessional team with structured, frequent patient reviews. In comparison, patients with HBI had no clear pathway or review frequency, although were supported by several therapeutic disciplines.

**Conclusion:** It is clear that patients experiencing severe Brain Injury (regardless of type) do experience prolonged hospital stays and uncertain recovery pathways. Although larger in number, patients with HBI tend to occupy both Critical Care and Ward Beds for lengthier periods than patients with TBI. This may lead to delays in accessing appropriate ongoing care and/or inappropriate use of resources in specialised hospital beds.

Further work is needed to unpick the layers of consistency in service provision for both groups.

#### A790 Studying mortality in critically ill patients - what does it teach us?

##### S. Castro^1^, T. Pires^2^, L. Melão^1^, A. Krystopchuk^1^, I. Pereira^1^, C. Granja^1,2,3^

###### ^1^Centro Hospitalar do Algarve, Serviço de Medicina Intensiva, Emergencia, Urgência e Cuidados Intensivos, Faro, Portugal; ^2^Universidade do Algarve, Ciências Biomédicas e Medicina, Faro, Portugal; ^3^Universidade do Porto, Faculdade de Medicina, CINTESIS, Porto, Portugal

####### **Correspondence:** S. Castro - Centro Hospitalar do Algarve, Serviço de Medicina Intensiva, Emergencia, Urgência e Cuidados Intensivos, Faro, Portugal

**Introduction:** Mortality in critically ill patients is high either while in the ICU but also after discharge from the Intensive Care Unit (ICU). The mortality rate must be understood and integrated into the hospital reality where the ICU is inserted.

**Objectives:** In the present study we aim to understand factors associated with ICU and hospital mortality and the trajectories and burden of functional limitations of ICU survivors.

**Methods:** Mortality was characterized at the time of discharge from ICU, in the first 48 hours of ICU and after ICU. Studied variables included type of admission (scheduled surgery, urgent surgery, medical and trauma), age, gender, SAPS II, main diagnosis, median length of stay (LS) before ICU, in ICU and after ICU.

Student test was performed to compare continuous values and chi-square for categorical variables. Man-Whitney *U* test was performed to study continuous variables nonparametric. Statistical significance was defined as p-values less than 0,05.

**Results:** A total of 446 patients were discharged from ICU in 2015. 118 (26.45 %) died in the ICU. 38 (8.5 %) patients died in the remainder time of their hospitalization. Hospital mortality was 34.9 %. Of the patients who died in the ICU, 59 (50 %) died in the first 48 hours.

Of the patients who died from septic shock, 36 were medical and 35 urgent surgical.

Septic shock was the main cause of death in medical and, in particular, in urgent surgical population. Medical patients had a early ICU mortality (<48 h) that was higher than in surgical patients. Higher age and higher severity of disease was also associated with higher mortality. Mortality was significantly higher in those patients who had a longer lenght of stay in hospital before ICU admission (Table 88).

Those who died after ICU had a median lenght of stay in hospital of 49 days; of these, a median of 8 days were spent in the ICU, a median of 13 days were spent in the ward before ICU admission and a median of 28 days were spent after ICU discharge until death occurs in the ward.

**Conclusions:** The perception of the conditions under which death occurs can help to rethink the organization of the critical patient circuit in the hospital, in particular to prompt earlier ICU admissions and to adress more aproppriately those patients who might or not benefit from ICU admission.

**References**

1. Capuzzo et al. Hospital mortality of adults admitted to Intensive Care Units in hospitals with and without Intermediate Care Units: a multicentre European cohort study. Critical Care 2014, 18:551

**Table 88 (abstract A791).** Characteristics of patients who suffered hospital mortality

Study variables	Patients discharged from hospital alive (n= 290)	Patients discharged from hospital dead (n=156 )	p
Admission type: urgent surgery;scheduled surgery;Medical;Trauma	72;62;130;26	50;9;83;14	<0,001*
Age in years (median)	59,58	64,9	0,001**
SAPS II (median)	46,46	64,9	<0,001**
Diagnosis (top 5):Septic shock;cardiogenic shock; Hypovolemic shock;Neurological emergency;Respiratory failure	93;13;11;20;24	79;5;6;21;9	0,127**;0,127**;0,127**;0,127**;0,127**
length of hospital stay (LS) in days (median)	32,2	22	-
LS before ICU	4	8	0,039***
LS in ICU	6,78	7,09	-
LS after ICU	21	7,0	-

(*chi-square ;**t-student test; *** Mann-Whitney U test)

#### A791 QuickSOFA and hospital mortality prediction in a Brazilian cohort of non-infected critically ill patients: a retrospective observational study

##### L.U. Taniguchi^1,2^, E.M.C. Pires^1^, J.M. Vieira Jr^1^, L.C.P. Azevedo^1,2^

###### ^1^Education and Research Institute, Hospital Sírio-Libanês, Intensive Care Unit, Sao Paulo, Brazil; ^2^Hospital das Clínicas, Universidade de São Paulo, Emergency Medicine Discipline, Sao Paulo, Brazil

####### **Correspondence:** L.U. Taniguchi - Hospital das Clínicas, Universidade de São Paulo, Emergency Medicine Discipline, Sao Paulo, Brazil

**Introduction:** QuickSOFA (qSOFA) was recently suggested to identify patients with suspected infection at higher risk of worse outcomes. However, qSOFA in non-infected patients has not been described. The objective of this study was to evaluate if qSOFA might predict hospital mortality in a Brazilian cohort of non-infected critically ill patients.

**Methods:** This is a retrospective cohort study conducted at a private tertiary hospital (Hospital Sirio-Libanes) at São Paulo, Brazil. We extracted relevant information from the adult intensive care unit (ICU) database (sistema Epimed™). A comparison was performed between SAPS 3 score model and qSOFA model at ICU admission both as a dichotomous variable (≥2 criteria - “qSOFA positive” versus 0–1 criterion - “qSOFA negative”) and as an ordinal variable from 0 to 3 (according to the number of qSOFA criteria met) for predicting hospital mortality in patients admitted to the ICU without infection. Models discriminations were compared using area under the ROC curves.

**Results:** Between January 2012 to December 2014 we studied 1,150 patients (hospital mortality of 12 %). Patients who died were more severely ill compared to patients those who survived (SAPS 3 of 56 [47–63] versus 36 [29–45] respectively; p < 0.001), were older, more frequently came from the ward with longer hospital length of stay before ICU admission, had more comorbidities, more frequently required mechanical ventilation and vasoactive drugs as well as dialysis, had higher lactate levels at admission (2.1 [1.3 - 3.5] versus 1.6 [1.1 - 2.5] respectively; p < 0.001), and more frequently were “qSOFA positive” (30.4 % vs 9.1 % respectively, p < 0.001). However, area under ROC curve for SAPS 3 was higher (0.84 [95 % CI 0.82 - 0.86]) than for qSOFA both as a dichotomous variable (0.61 [95 % CI 0.58 - 0.64]) as an ordinal variable (0.67 [95 % CI 0.65 - 0.70]; p < 0.001 for comparisons between SAPS 3 and both qSOFA models).

**Conclusion:** The utility of qSOFA to recognize severity of illness and predict mortality in our cohort of ICU patients is similar to the previous description in suspected infection, but less accurate than SAPS 3.

